# Supplementary material for: Crosstalk between Host Genome and Metabolome among People with HIV in South Africa
Source: Metabolites. 2022 Jul 6;12(7):624. doi: 10.3390/metabo12070624 (PMC9316179; doi:10.3390/metabo12070624)
Supplement: Supplementary file 1 [file metabolites-12-00624-s001.zip › SuppFigS1_QQ_plots.pdf]

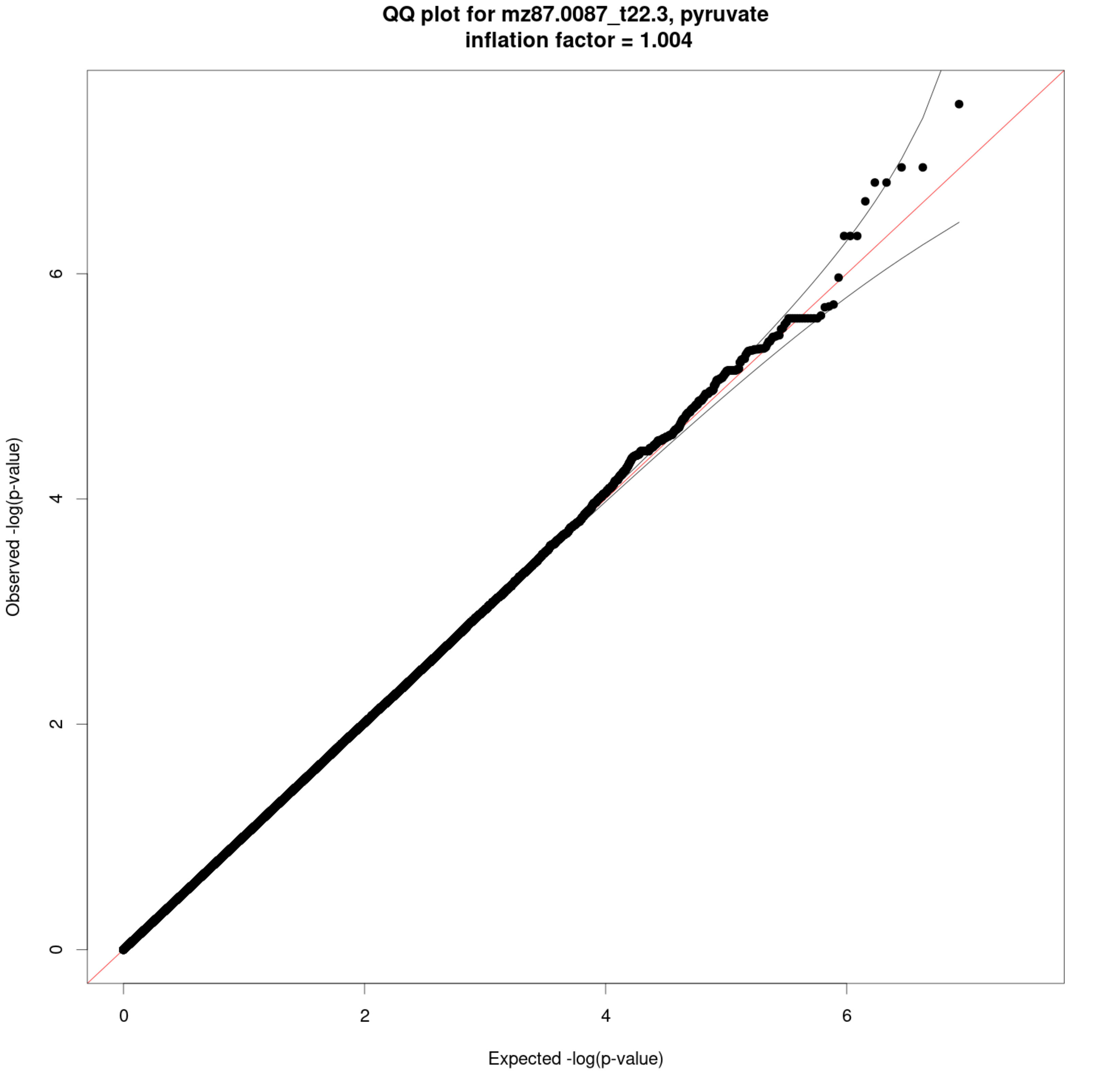
QQ plot for mz87.0087_t22.3, pyruvate
inflation factor = 1.004
Observed -log(p-value)
Expected -log(p-value)
0
2
4
6
0
2
4
6

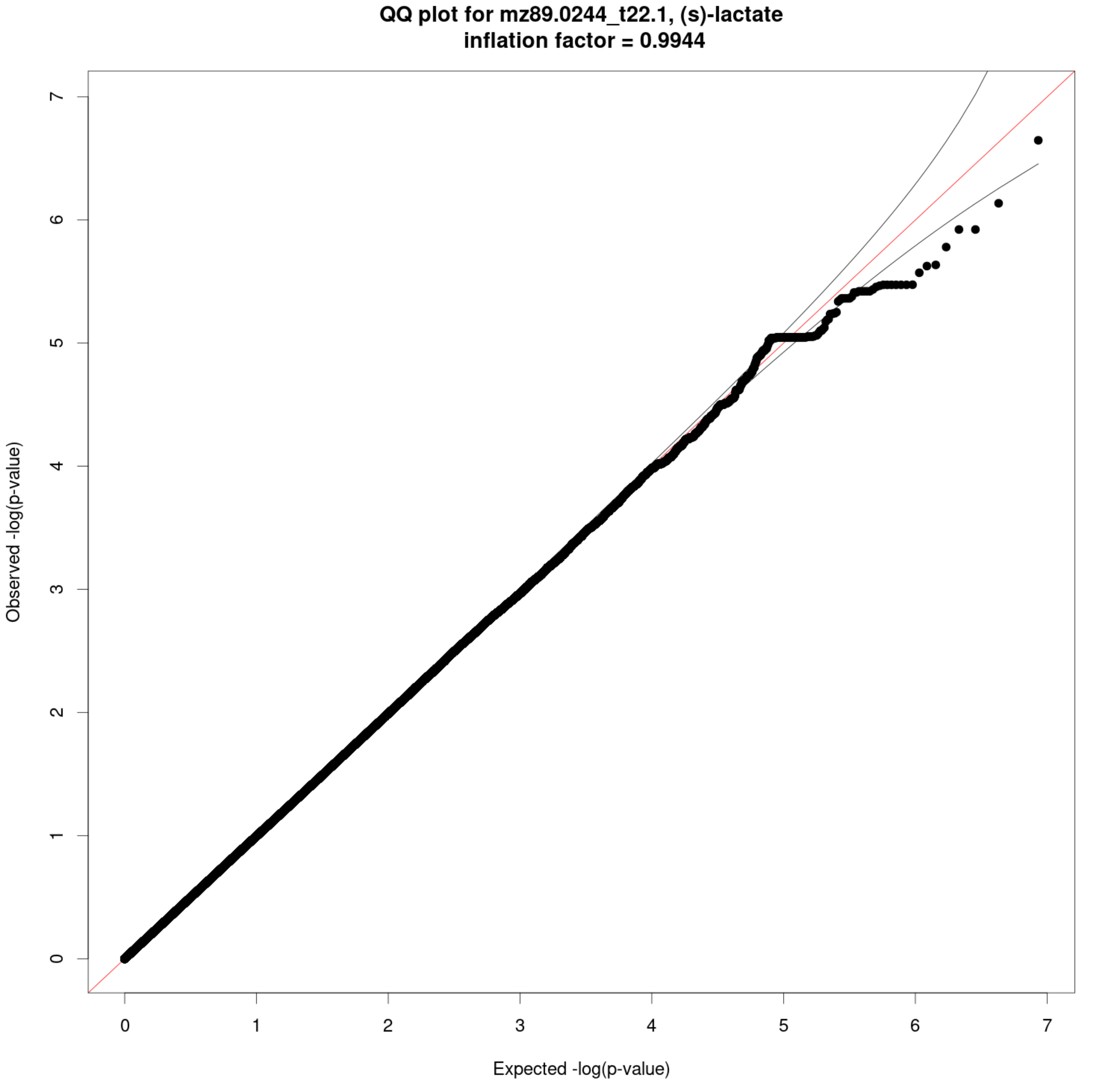
QQ plot for mz89.0244_t22.1, (s)-lactate
inflation factor = 0.9944
Observed -log(p-value)
Expected -log(p-value)
0
1
2
3
4
5
6
7

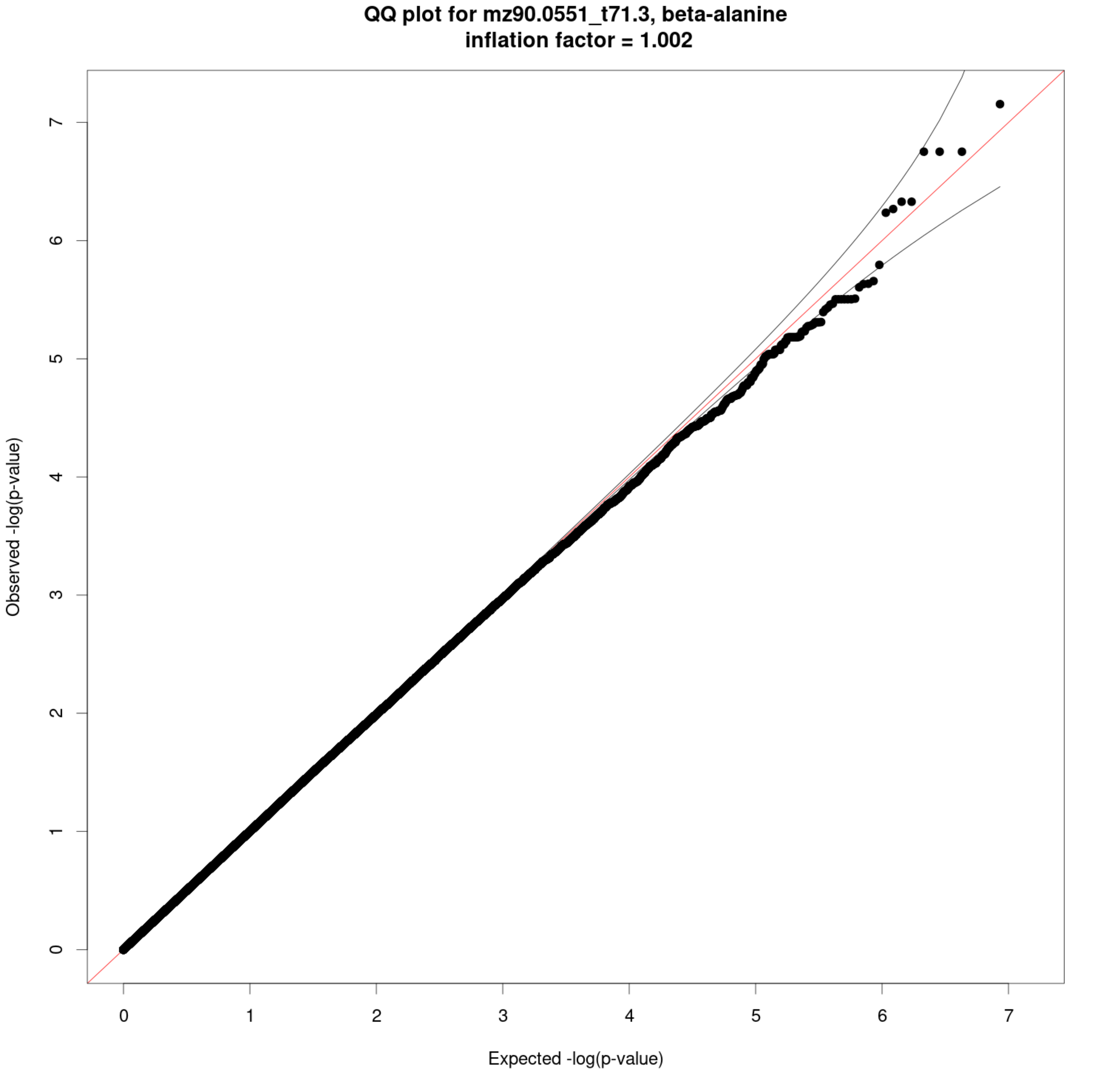
QQ plot for mz90.0551_t71.3, beta-alanine
inflation factor = 1.002
Observed -log(p-value)
Expected -log(p-value)
0
1
2
3
4
5
6
7

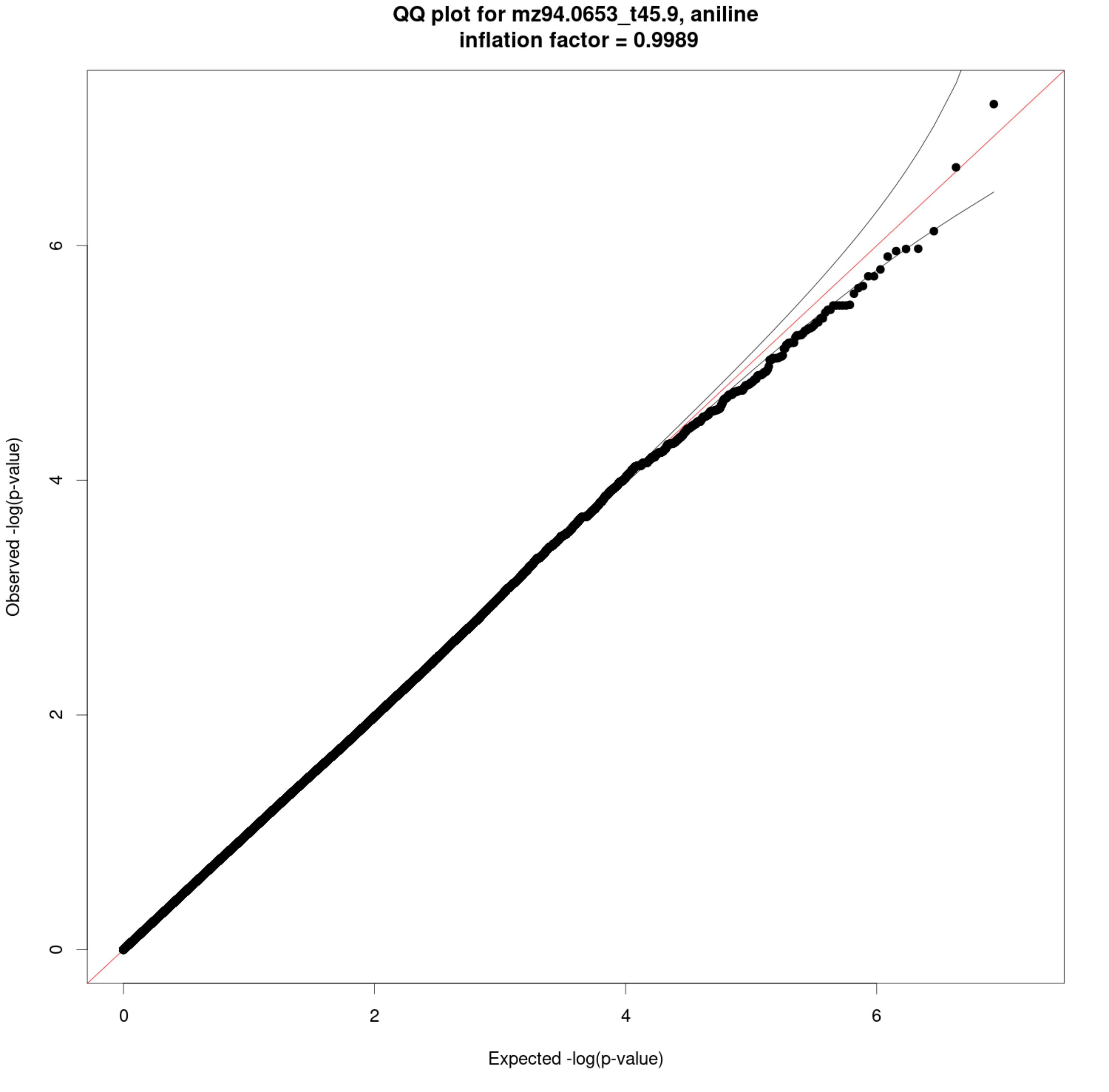
QQ plot for mz94.0653_t45.9, aniline
inflation factor = 0.9989
Observed -log(p-value)
Expected -log(p-value)
0
2
4
6
0
2
4
6

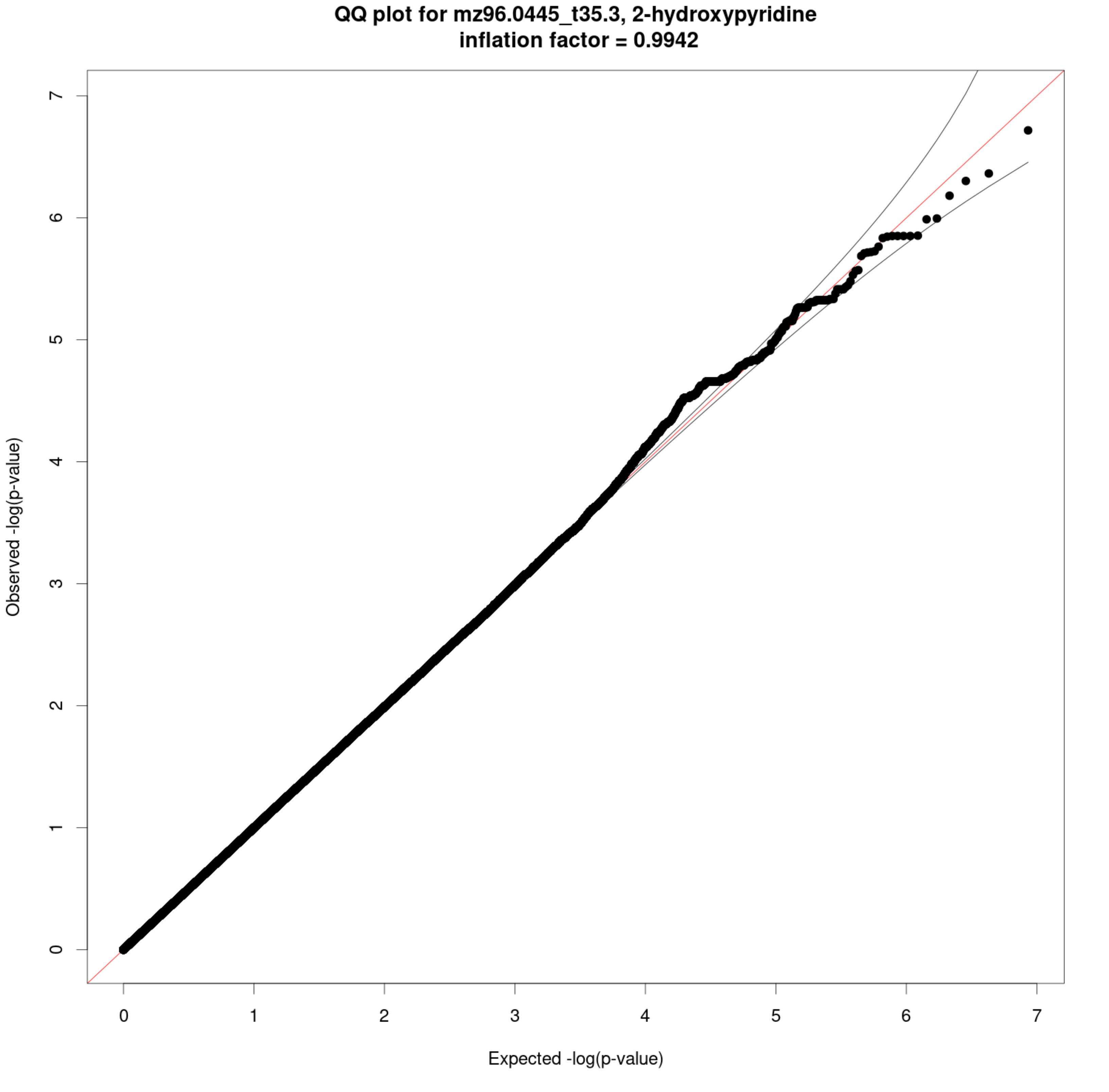

QQ plot for mz96.0445_t35.3, 2-hydroxypyridine
inflation factor = 0.9942
Observed -log(p-value)
Expected -log(p-value)
0
1
2
3
4
5
6
7

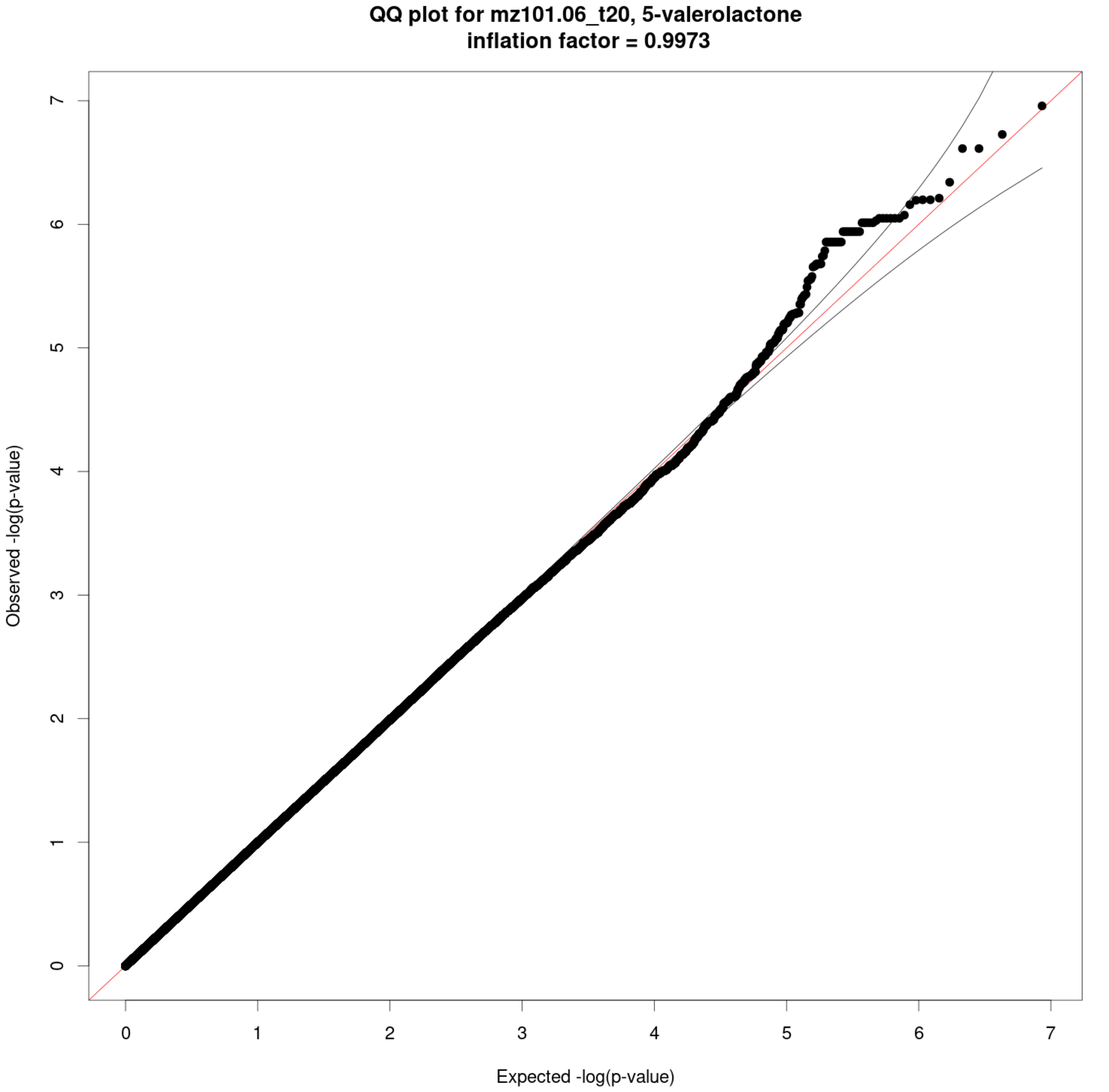
QQ plot for mz101.06_t20, 5-valerolactone
inflation factor = 0.9973
Observed -log(p-value)
Expected -log(p-value)
0
1
2
3
4
5
6
7
0
1
2
3
4
5
6
7

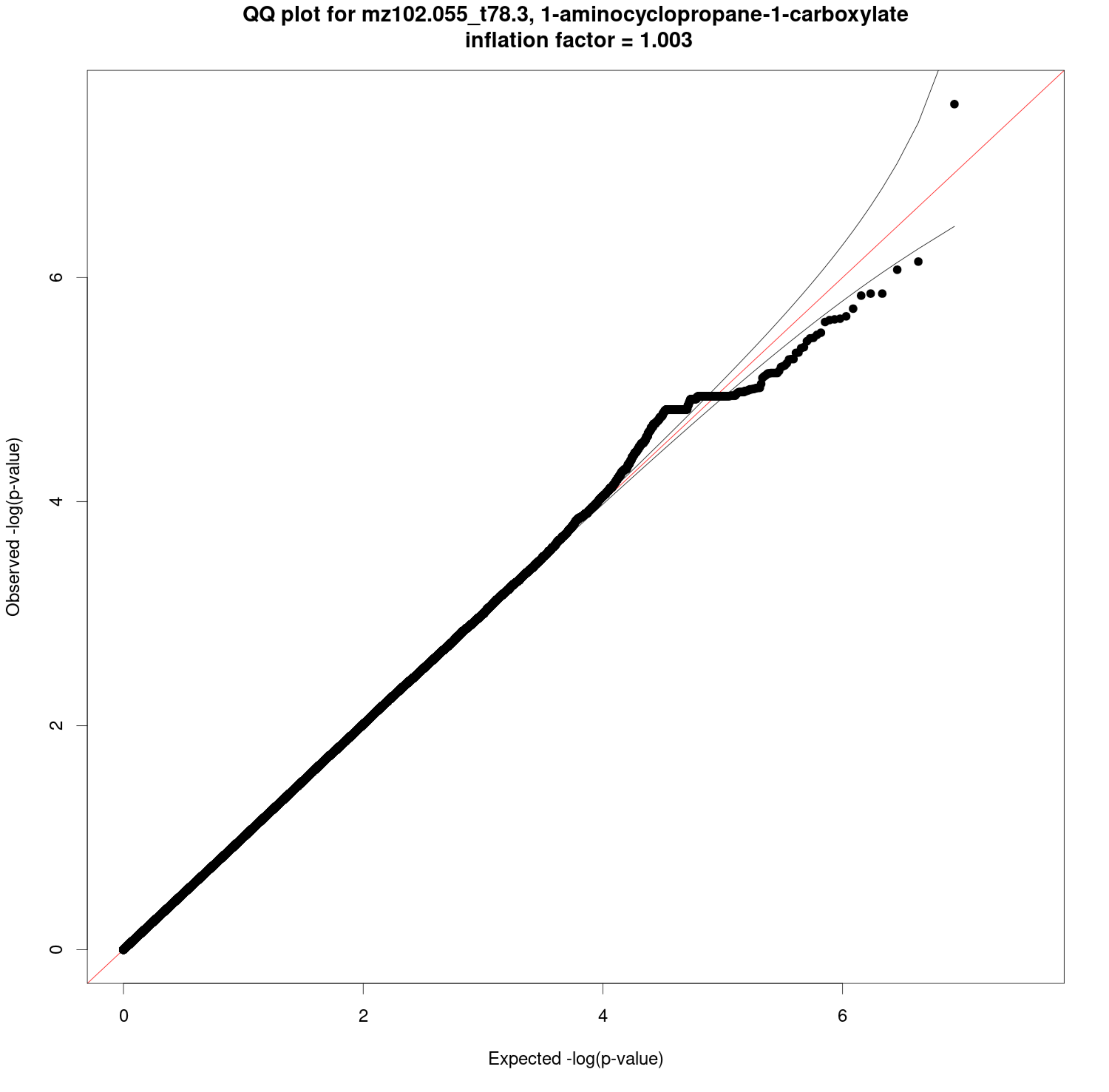
QQ plot for mz102.055_t78.3, 1-aminocyclopropane-1-carboxylate
inflation factor = 1.003
Observed -log(p-value)
Expected -log(p-value)
0
2
4
6
0
2
4
6

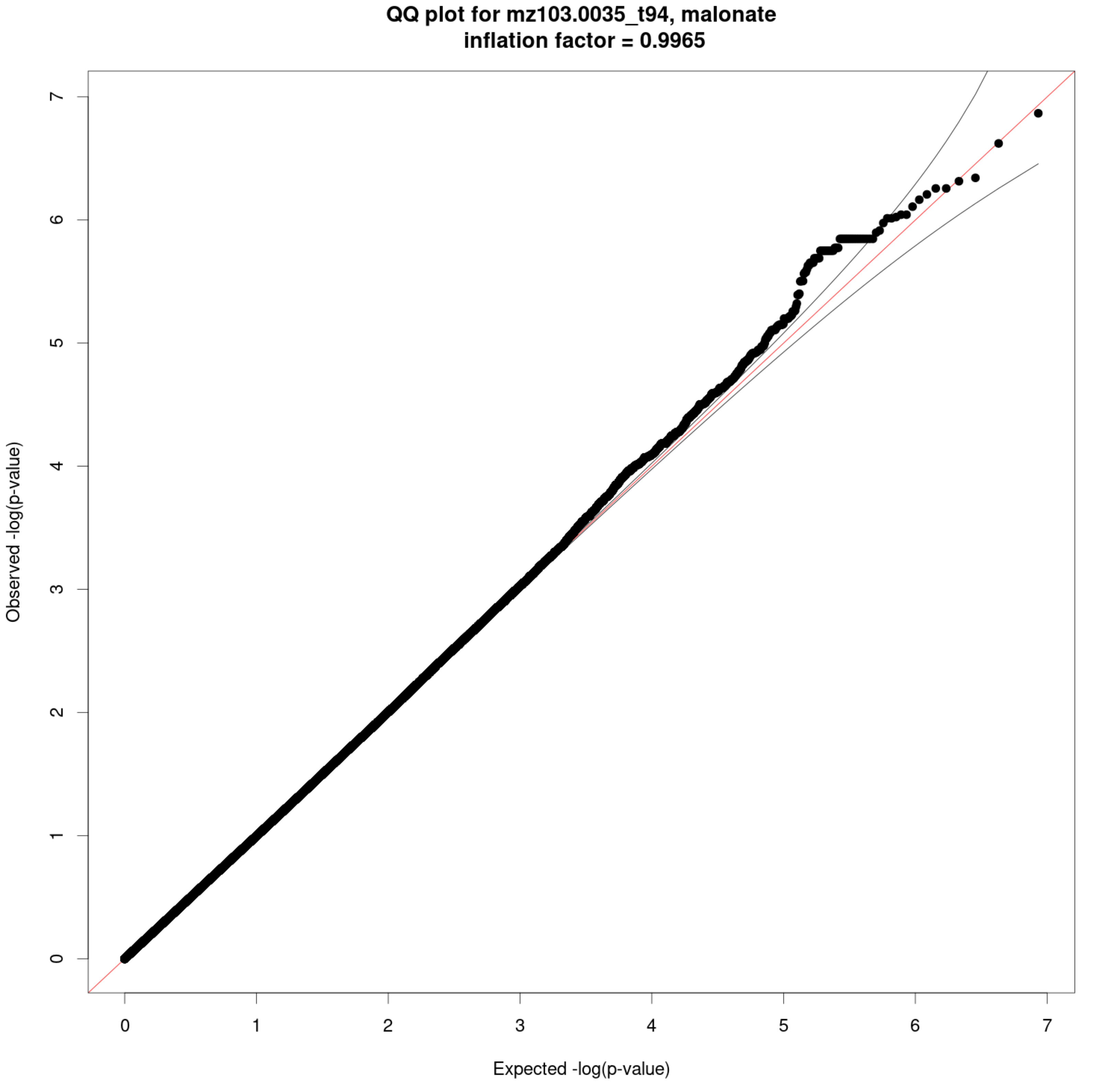
QQ plot for mz103.0035_t94, malonate
inflation factor = 0.9965
Observed -log(p-value)
Expected -log(p-value)
0
1
2
3
4
5
6
7

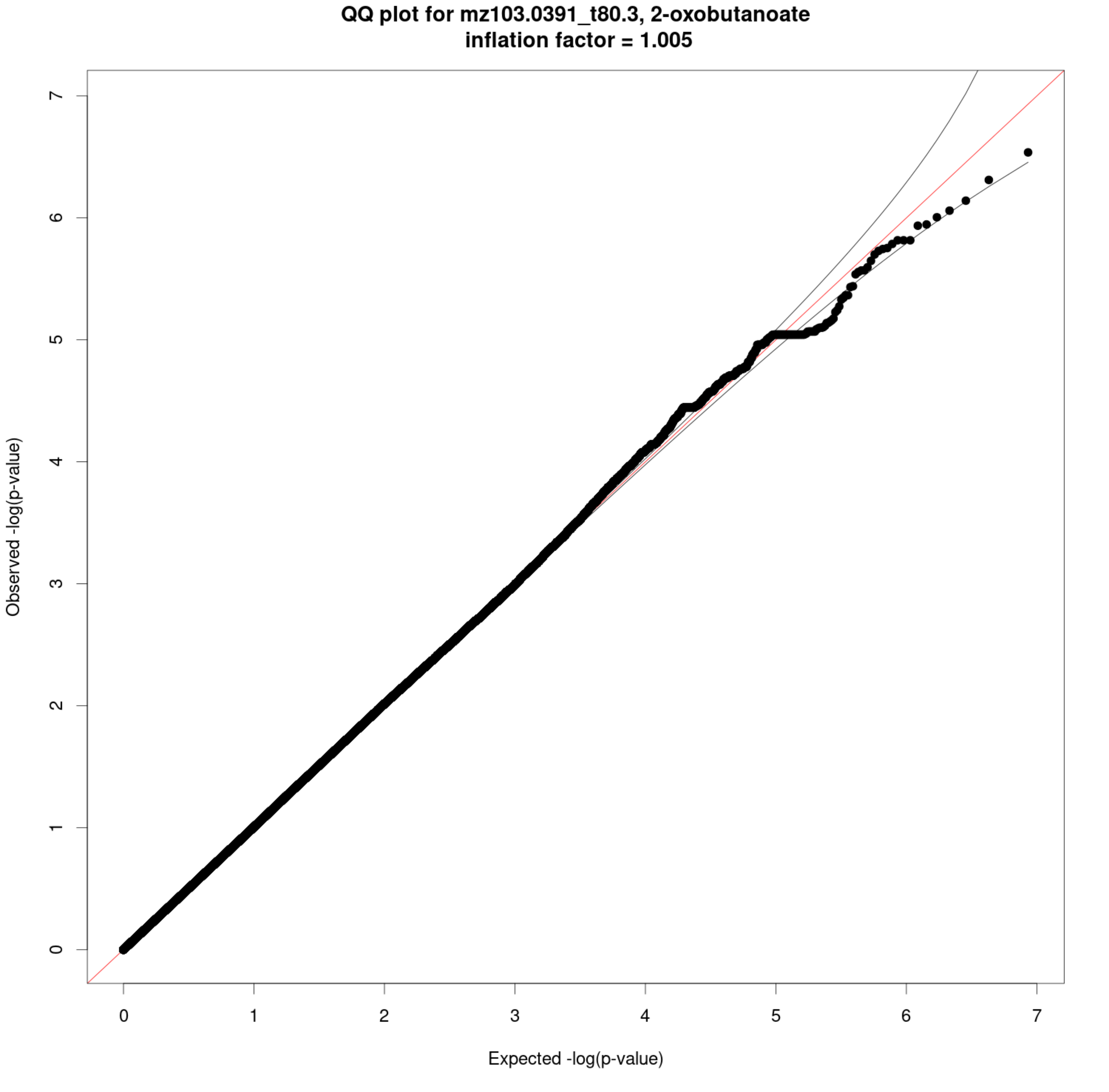
QQ plot for mz103.0391_t80.3, 2-oxobutanoate
inflation factor = 1.005
Observed -log(p-value)
Expected -log(p-value)
0
1
2
3
4
5
6
7

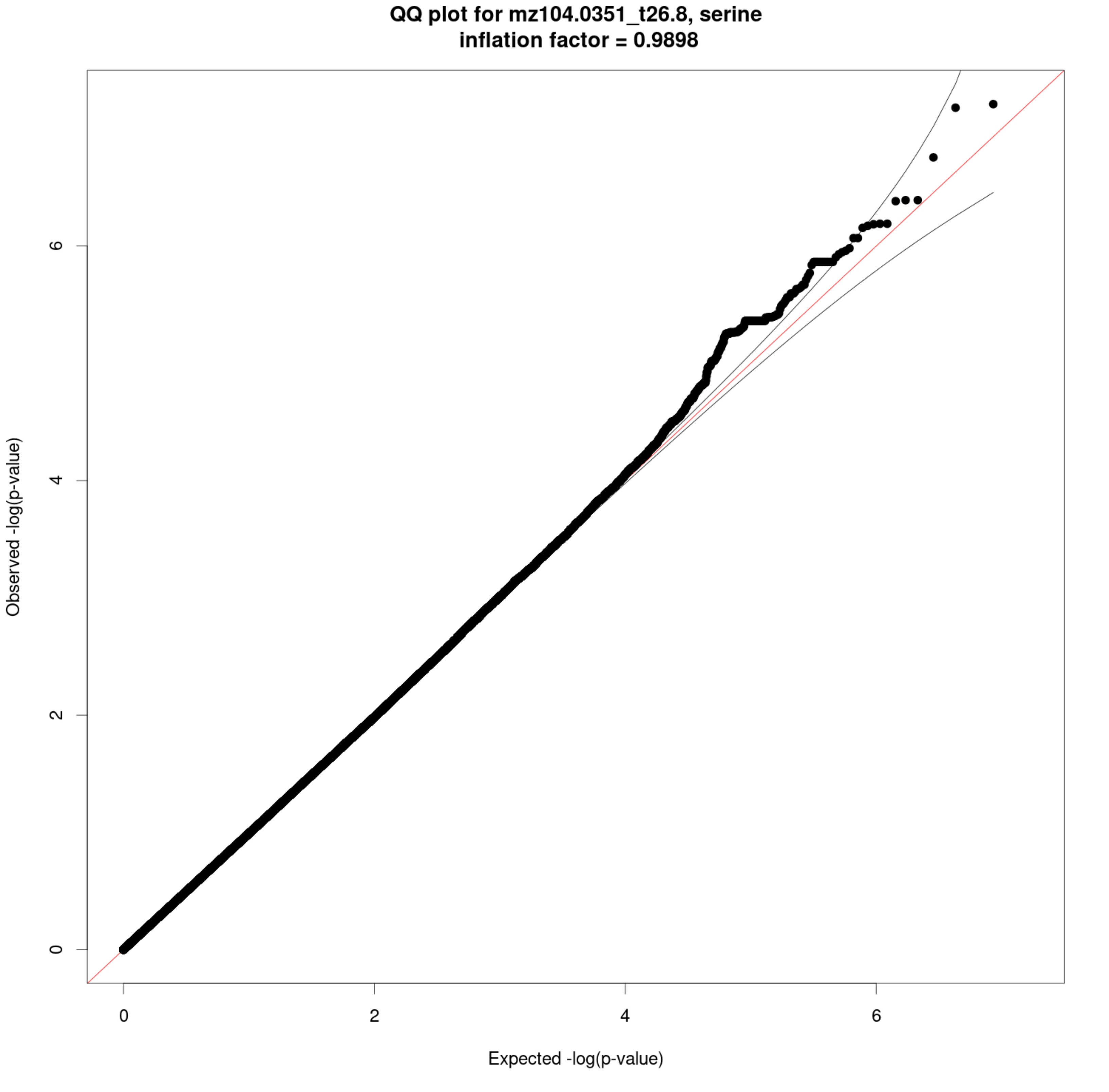
QQ plot for mz104.0351_t26.8, serine
inflation factor = 0.9898
Observed -log(p-value)
Expected -log(p-value)
0
2
4
6
0
2
4
6

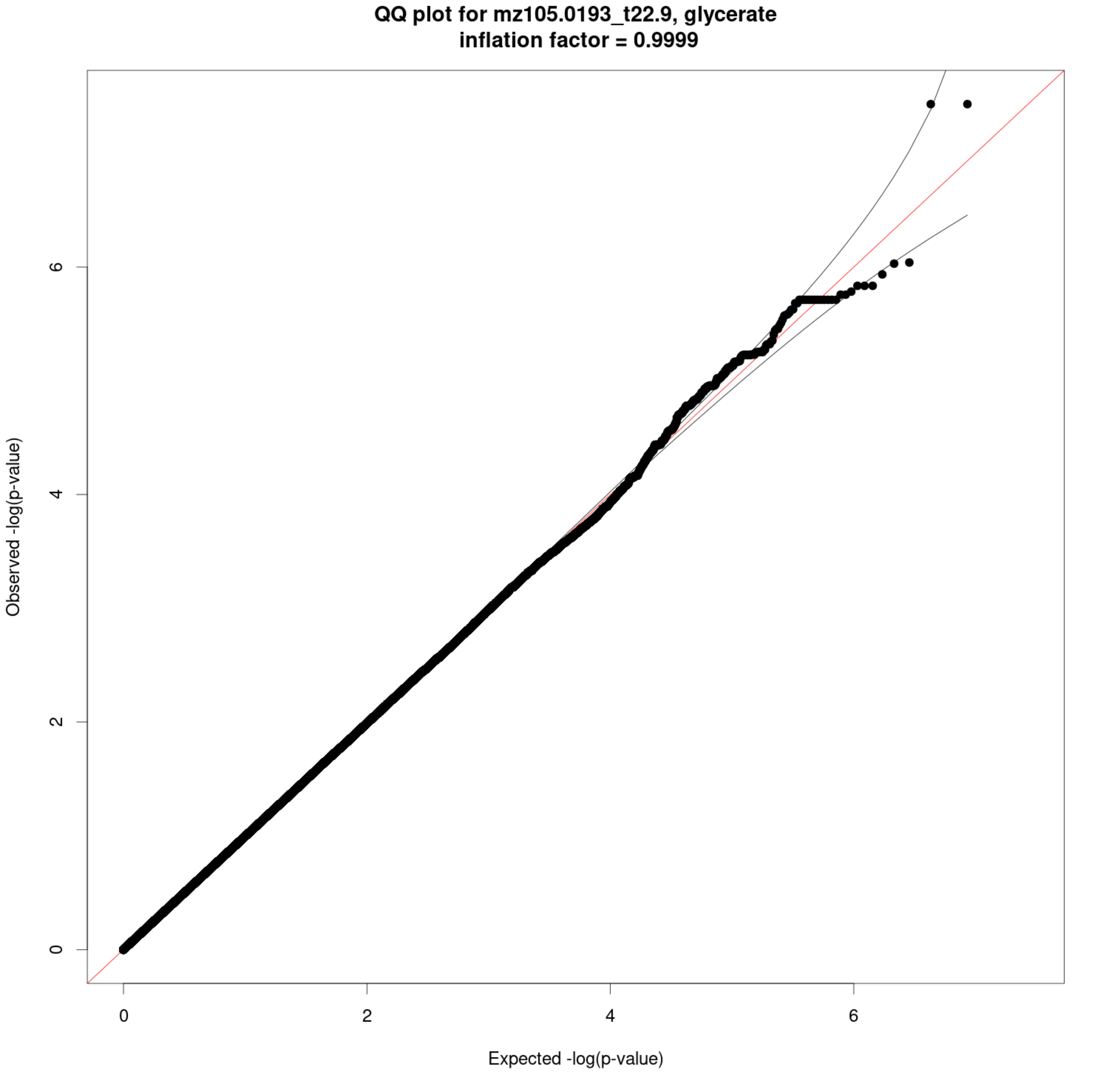
QQ plot for mz105.0193_t22.9, glycerate
inflation factor = 0.9999
Observed -log(p-value)
Expected -log(p-value)
0
2
4
6

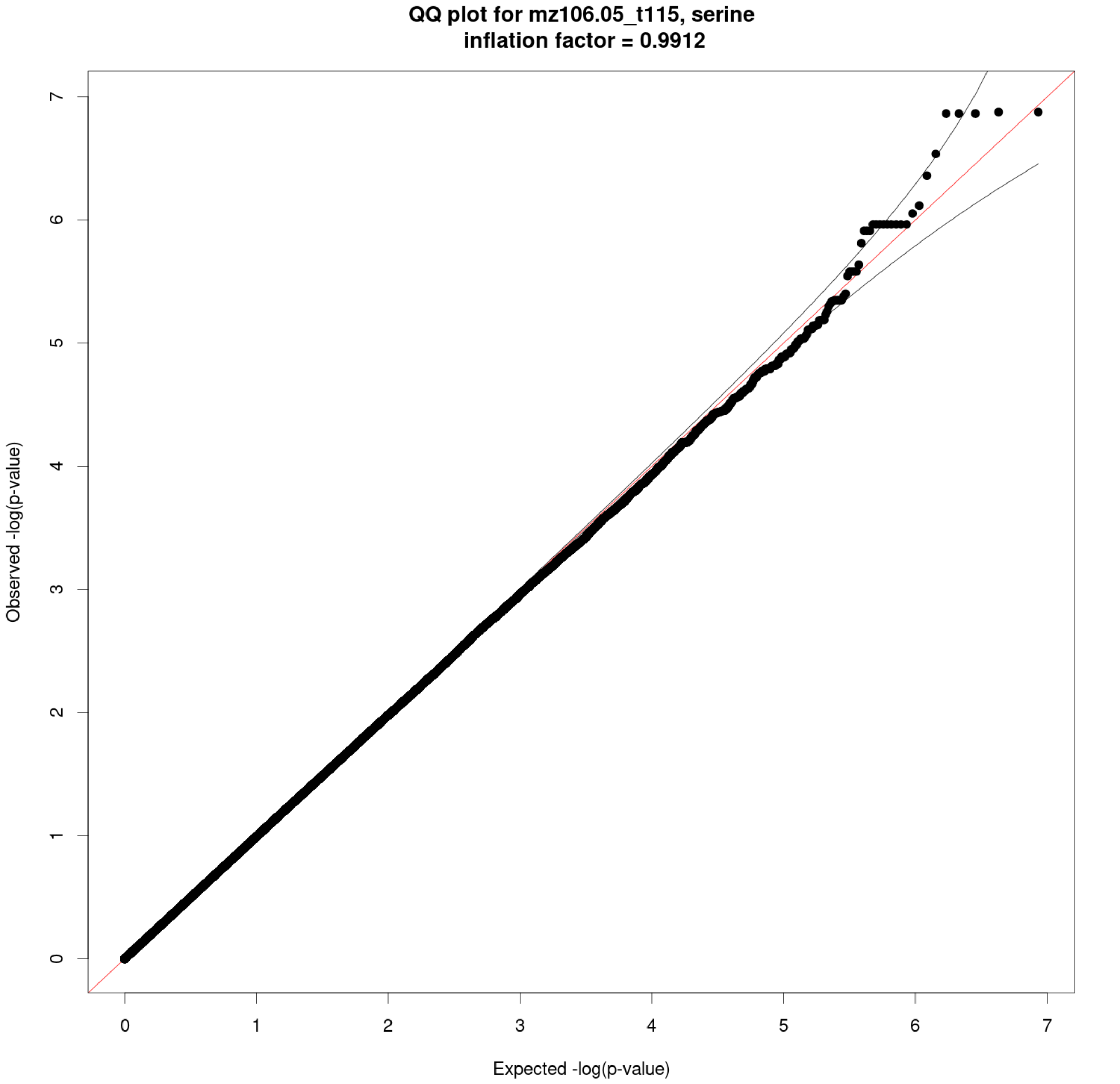

QQ plot for mz106.05_t115, serine
inflation factor = 0.9912
Observed -log(p-value)
Expected -log(p-value)
0
1
2
3
4
5
6
7

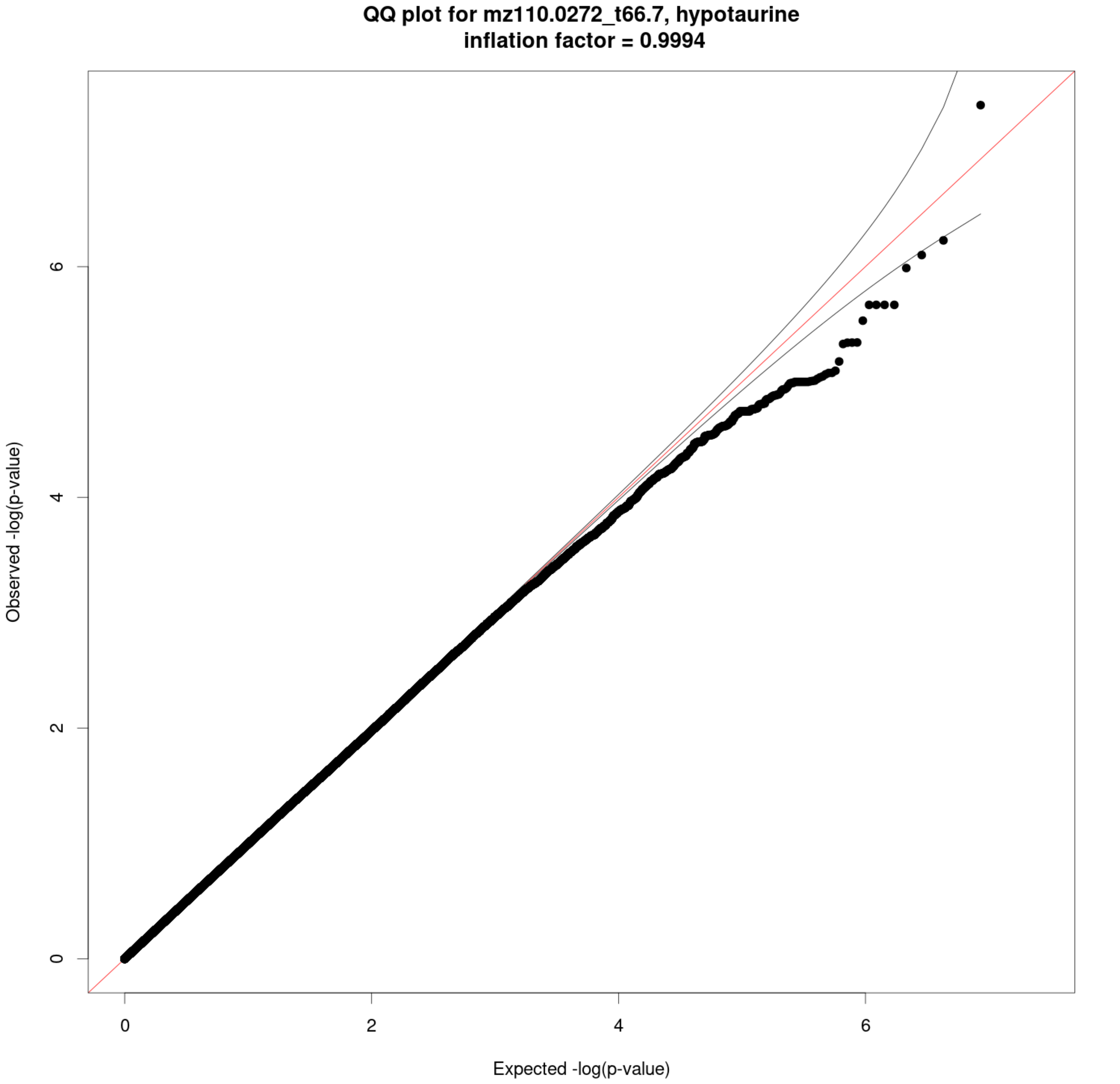
QQ plot for mz110.0272_t66.7, hypotaurine
inflation factor = 0.9994
Observed -log(p-value)
Expected -log(p-value)
0
2
4
6

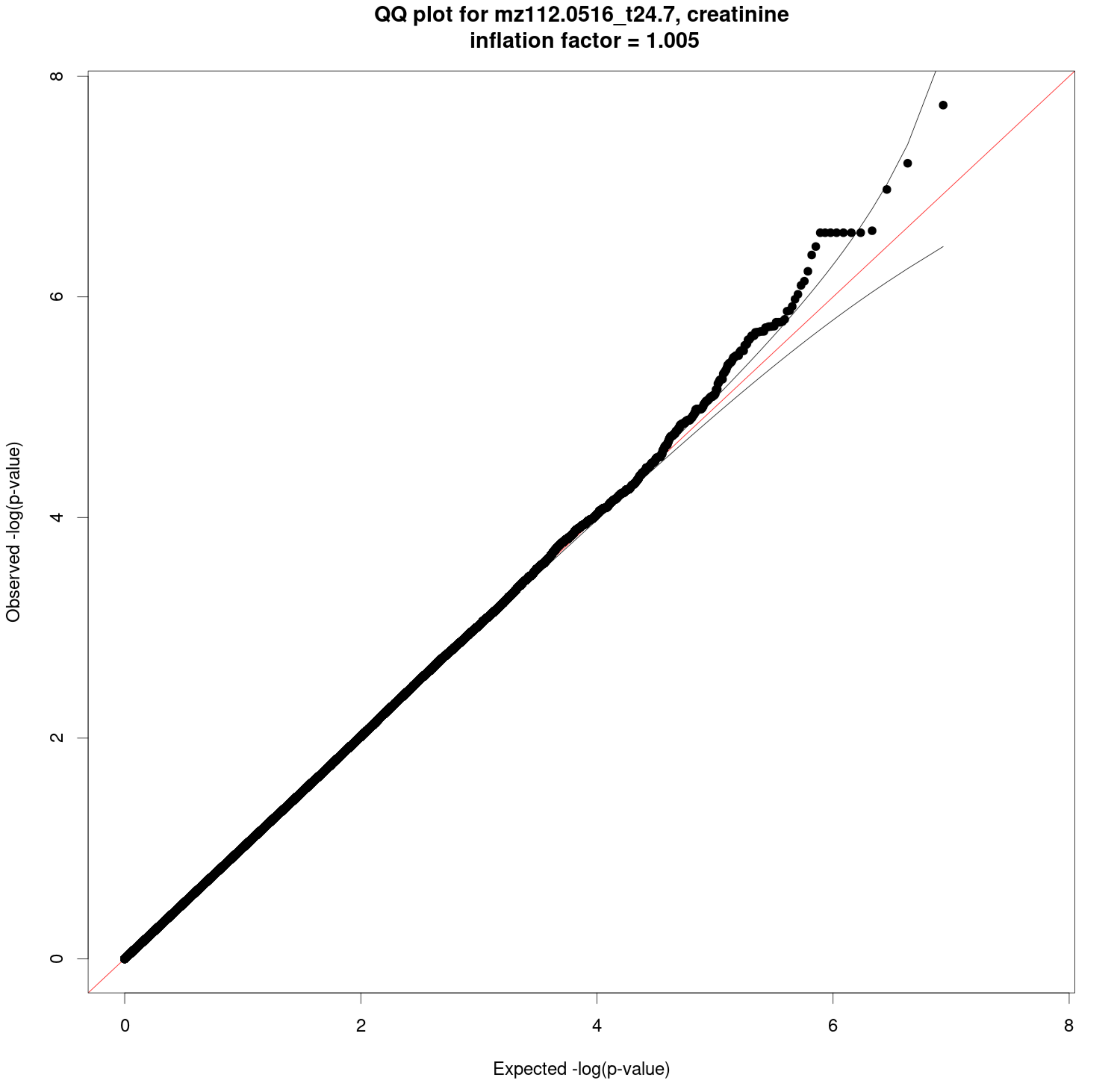
QQ plot for mz112.0516_t24.7, creatinine
inflation factor = 1.005
Observed -log(p-value)
Expected -log(p-value)
0
2
4
6
8

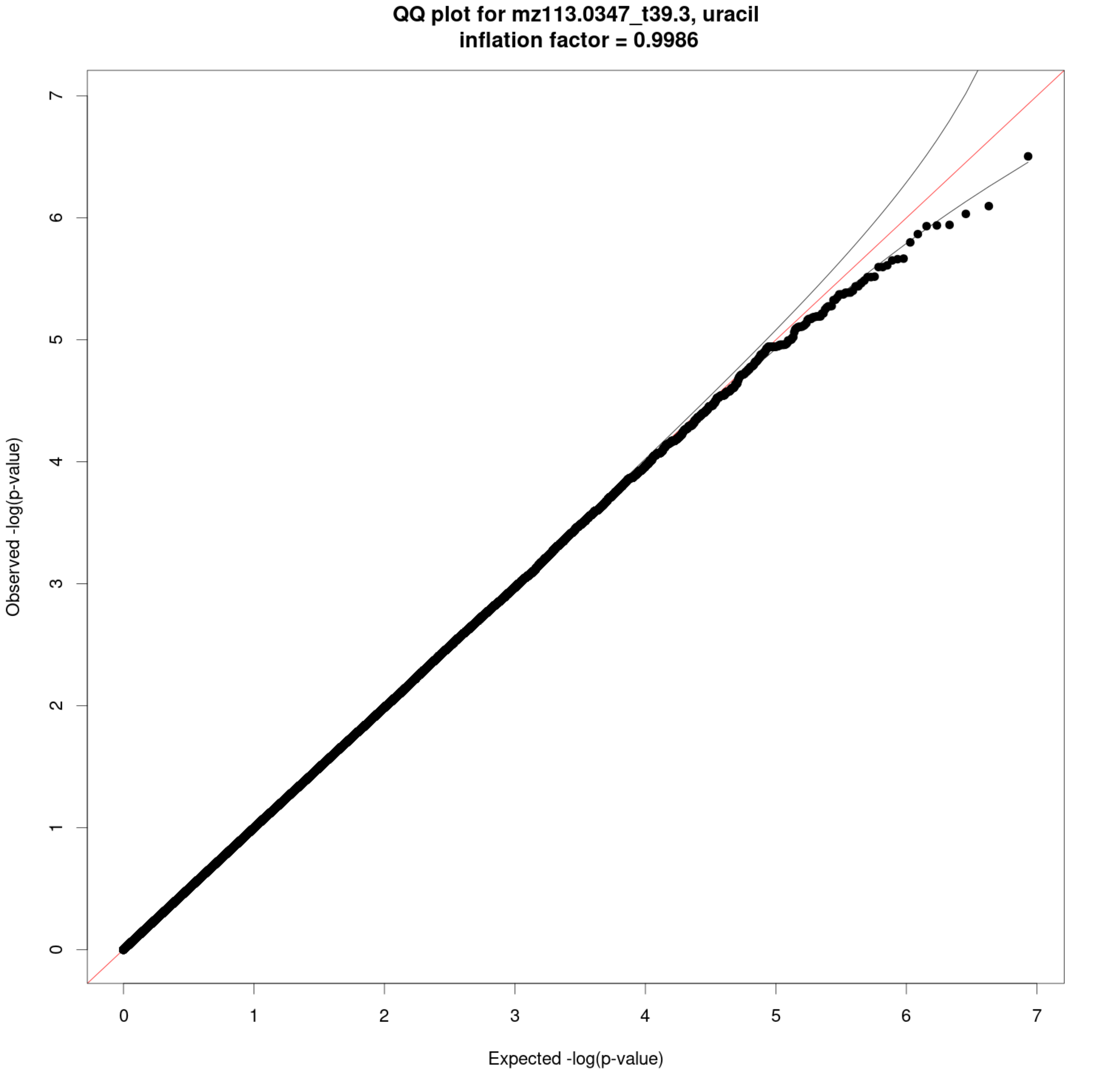
QQ plot for mz113.0347_t39.3, uracil
inflation factor = 0.9986
Observed -log(p-value)
Expected -log(p-value)
0
1
2
3
4
5
6
7

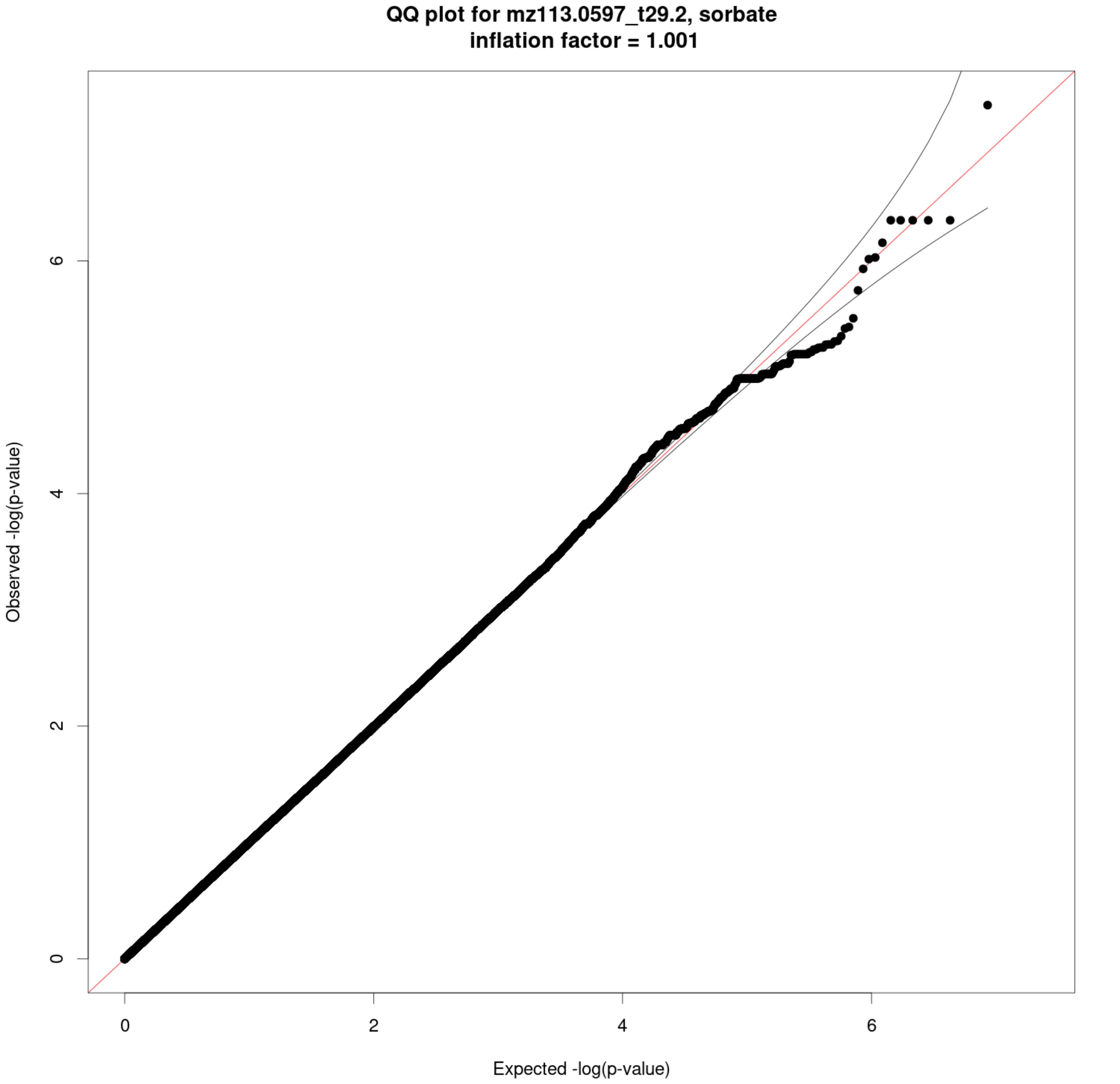
QQ plot for mz113.0597_t29.2, sorbate
inflation factor = 1.001
Observed -log(p-value)
Expected -log(p-value)
0
2
4
6

QQ plot for mz114.0661_t40.7, creatinine
inflation factor = 1.002

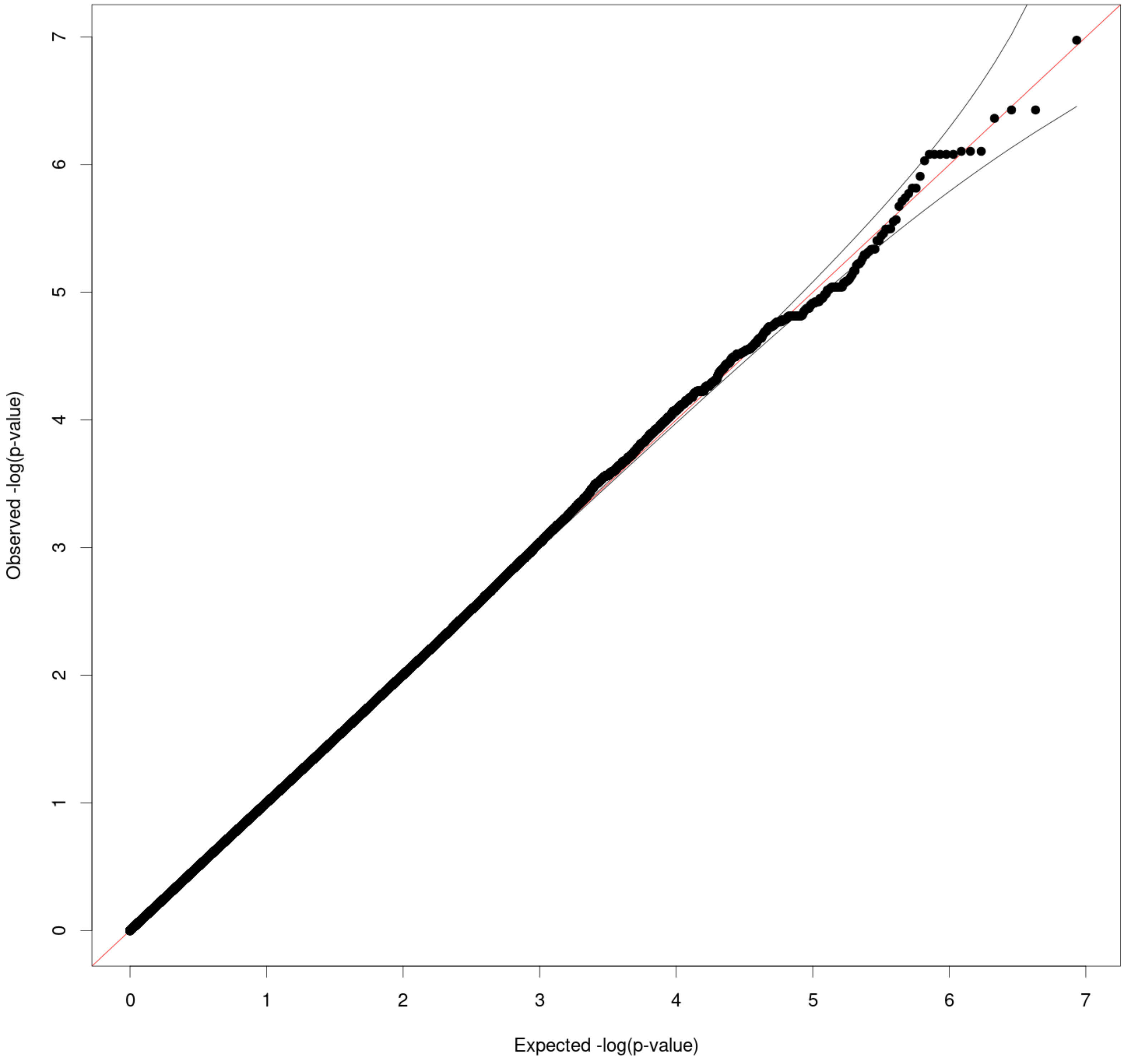

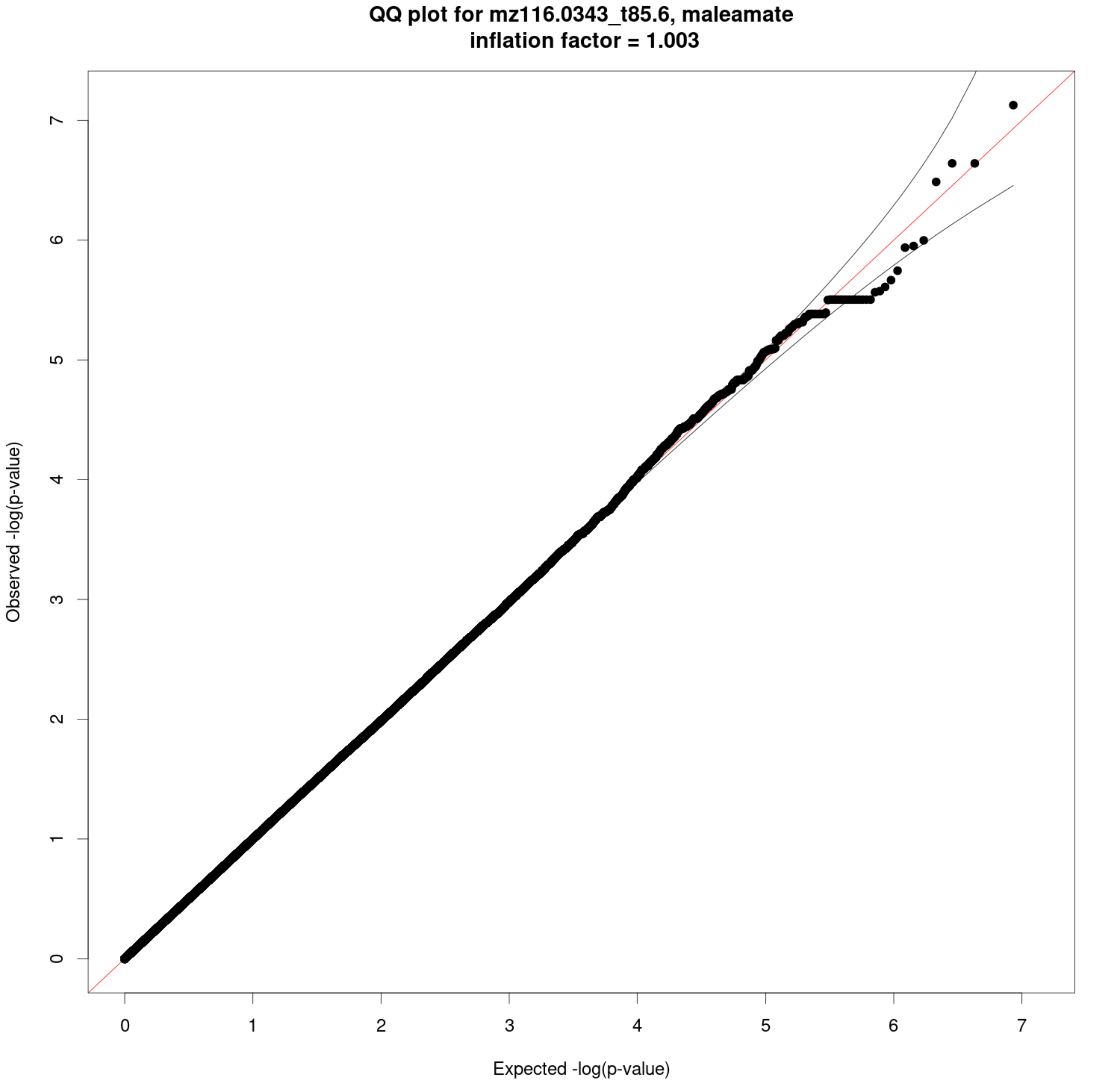
QQ plot for mz116.0343_t85.6, maleamate
inflation factor = 1.003
Observed -log(p-value)
Expected -log(p-value)
0
1
2
3
4
5
6
7

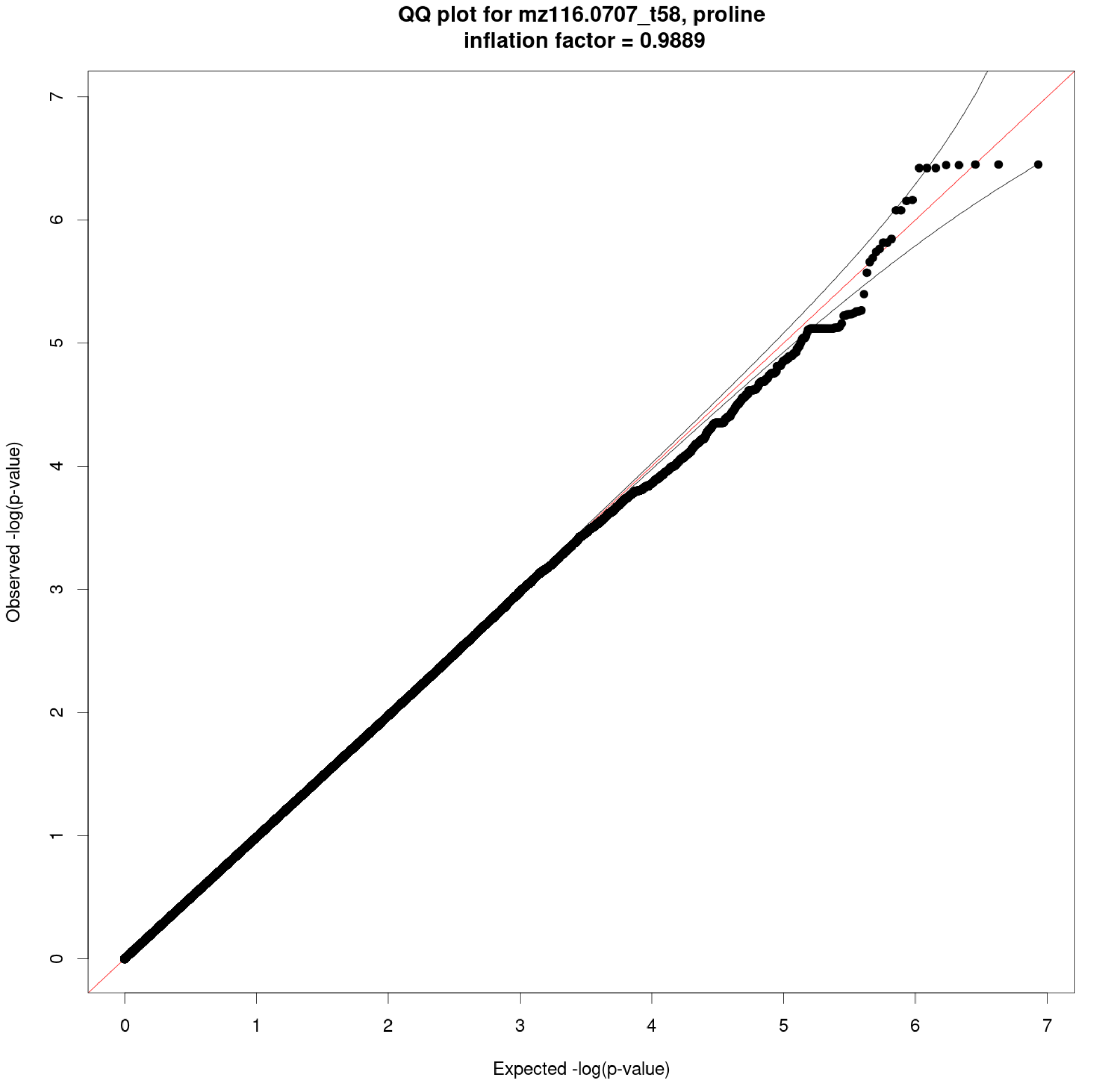
QQ plot for mz116.0707_t58, proline
inflation factor = 0.9889
Observed -log(p-value)
Expected -log(p-value)
0
1
2
3
4
5
6
7

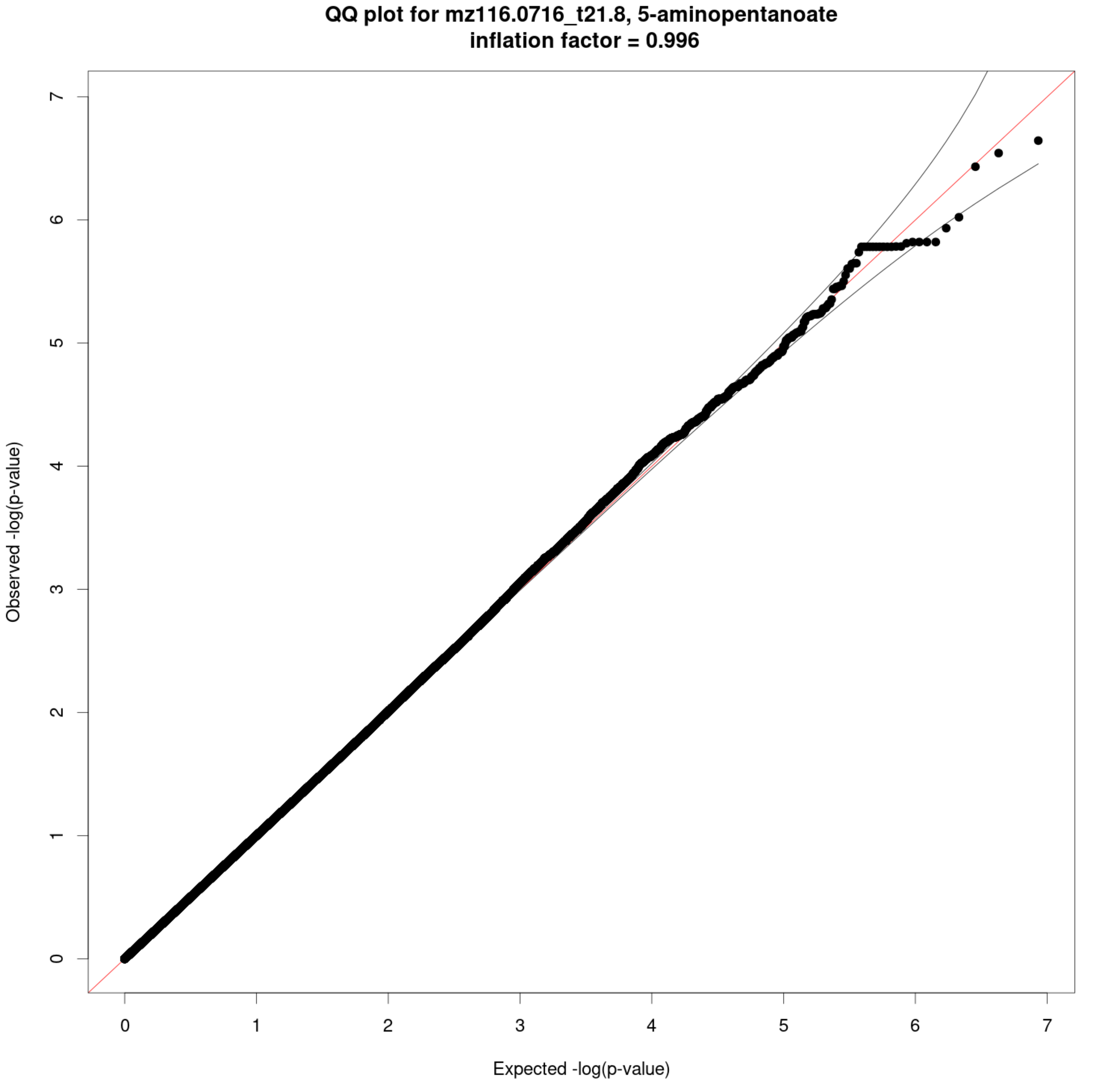
QQ plot for mz116.0716_t21.8, 5-aminopentanoate
inflation factor = 0.996
Observed -log(p-value)
Expected -log(p-value)
0
1
2
3
4
5
6
7

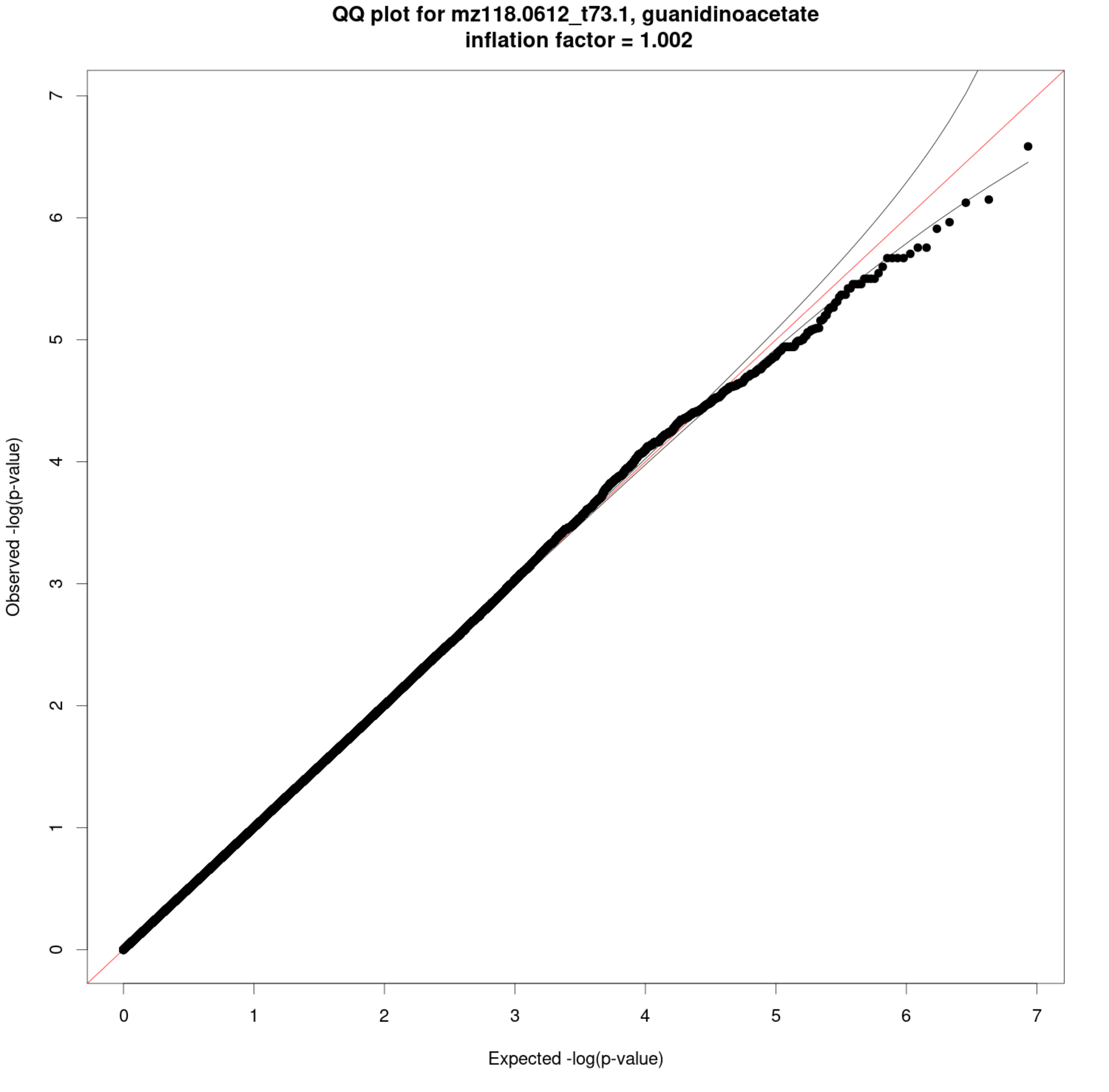
QQ plot for mz118.0612_t73.1, guanidinoacetate
inflation factor = 1.002
Observed -log(p-value)
Expected -log(p-value)
0
1
2
3
4
5
6
7

QQ plot for mz123.045_t27.4, 3-hydroxybenzyl alcohol
inflation factor = 1.011

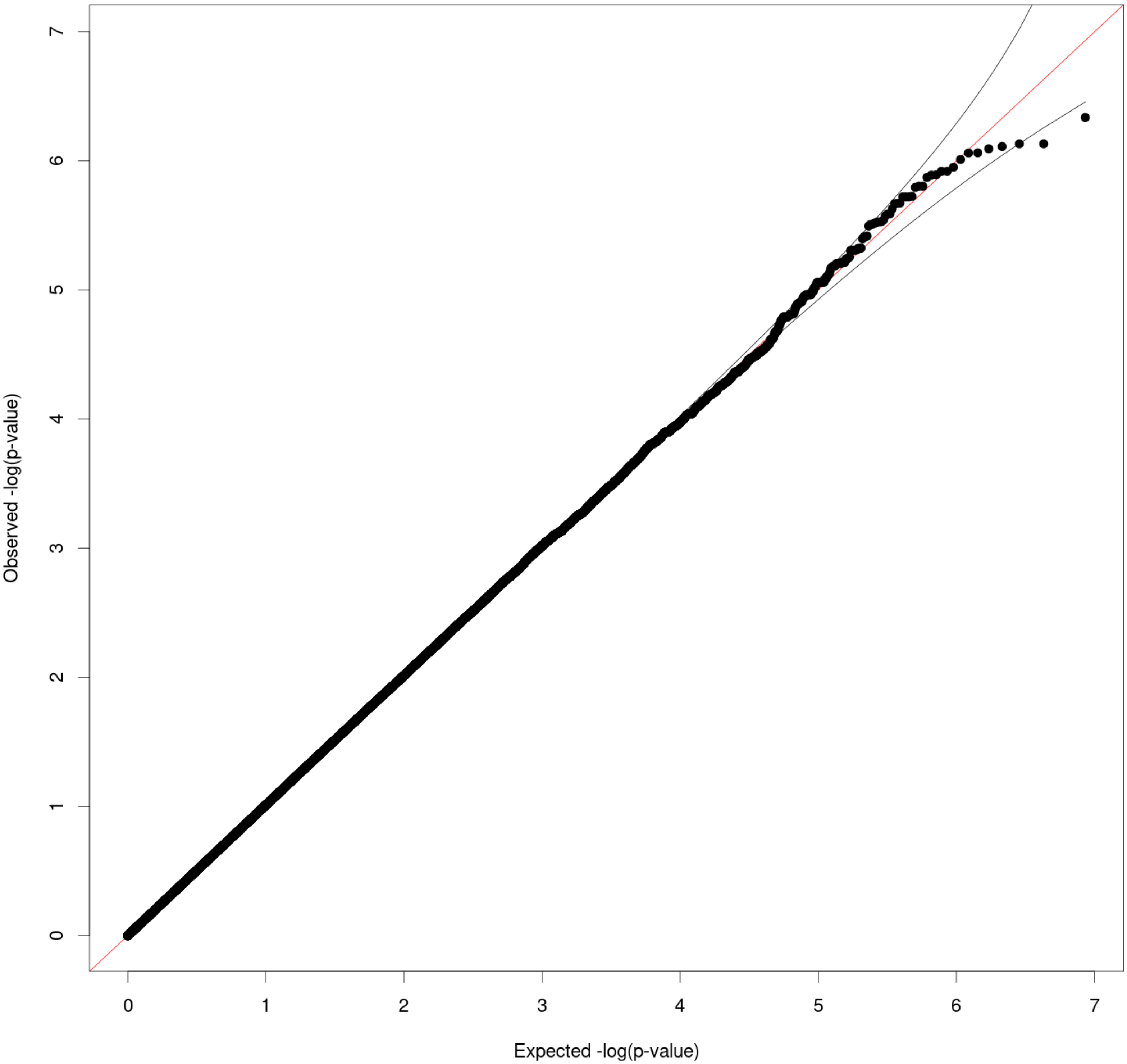

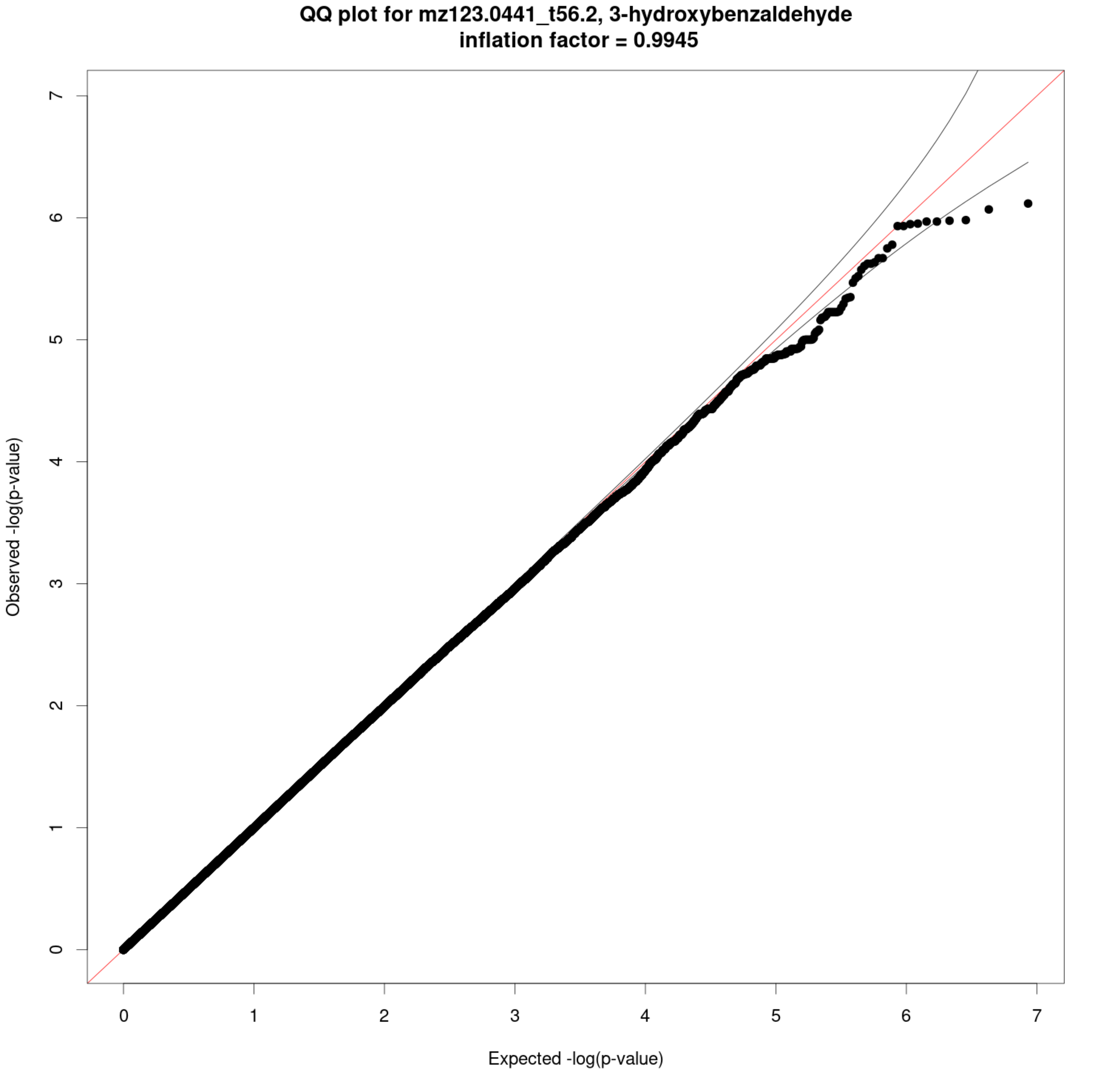
QQ plot for mz123.0441_t56.2, 3-hydroxybenzaldehyde
inflation factor = 0.9945
Observed -log(p-value)
Expected -log(p-value)
0
1
2
3
4
5
6
7

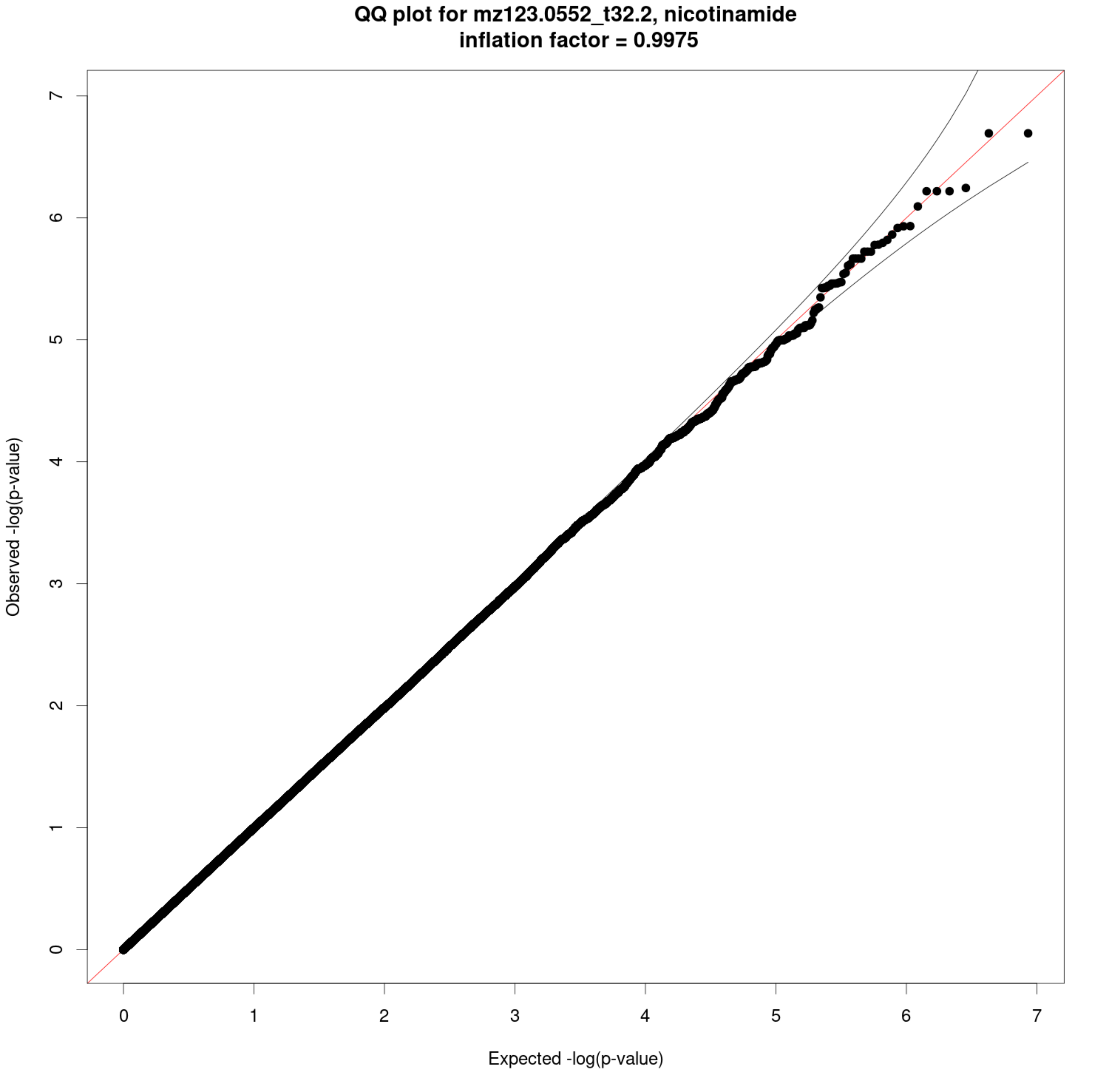
QQ plot for mz123.0552_t32.2, nicotinamide
inflation factor = 0.9975
Observed -log(p-value)
Expected -log(p-value)
0
1
2
3
4
5
6
7

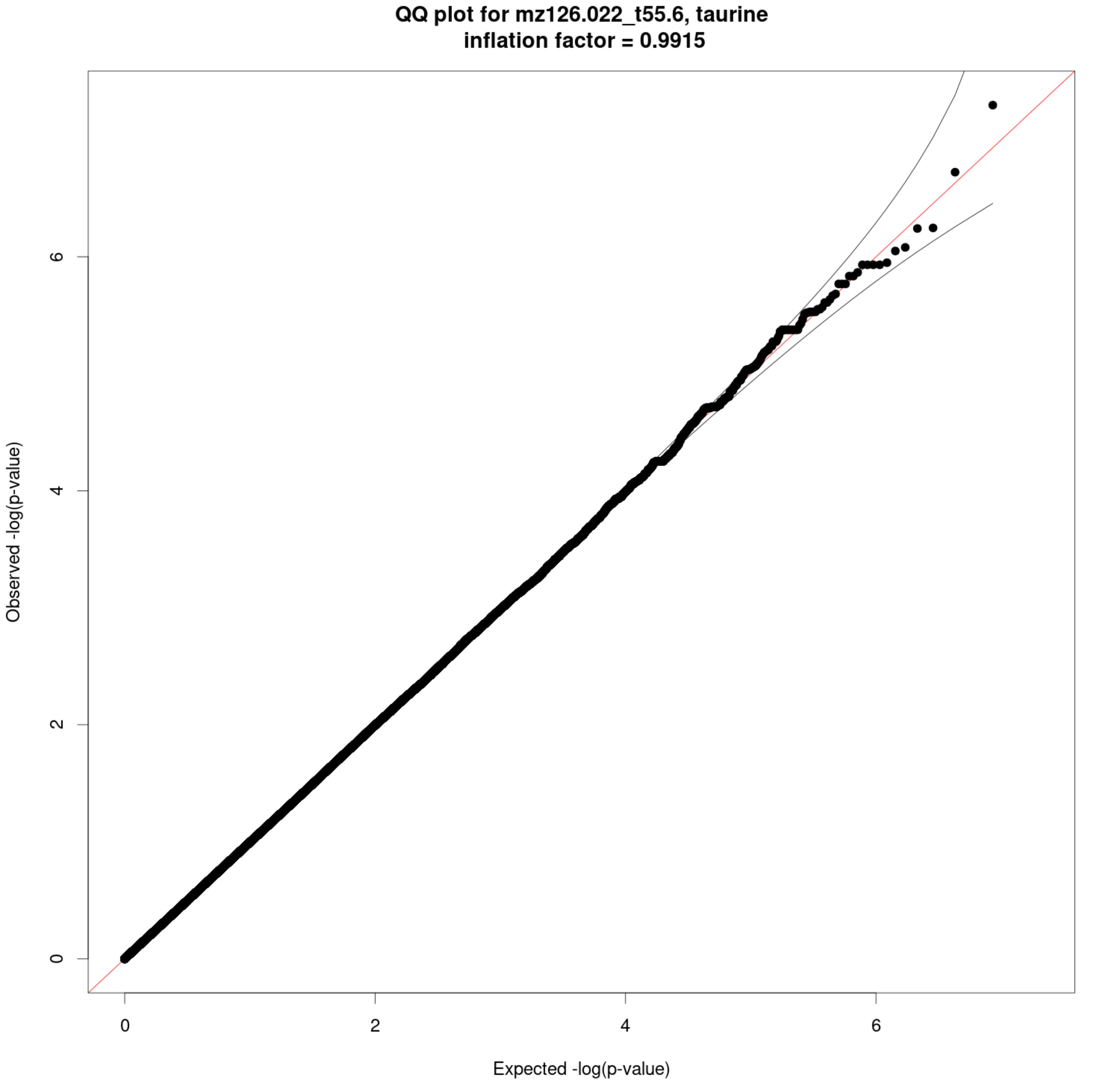
QQ plot for mz126.022_t55.6, taurine
inflation factor = 0.9915
Observed -log(p-value)
Expected -log(p-value)
0
2
4
6
0
2
4
6

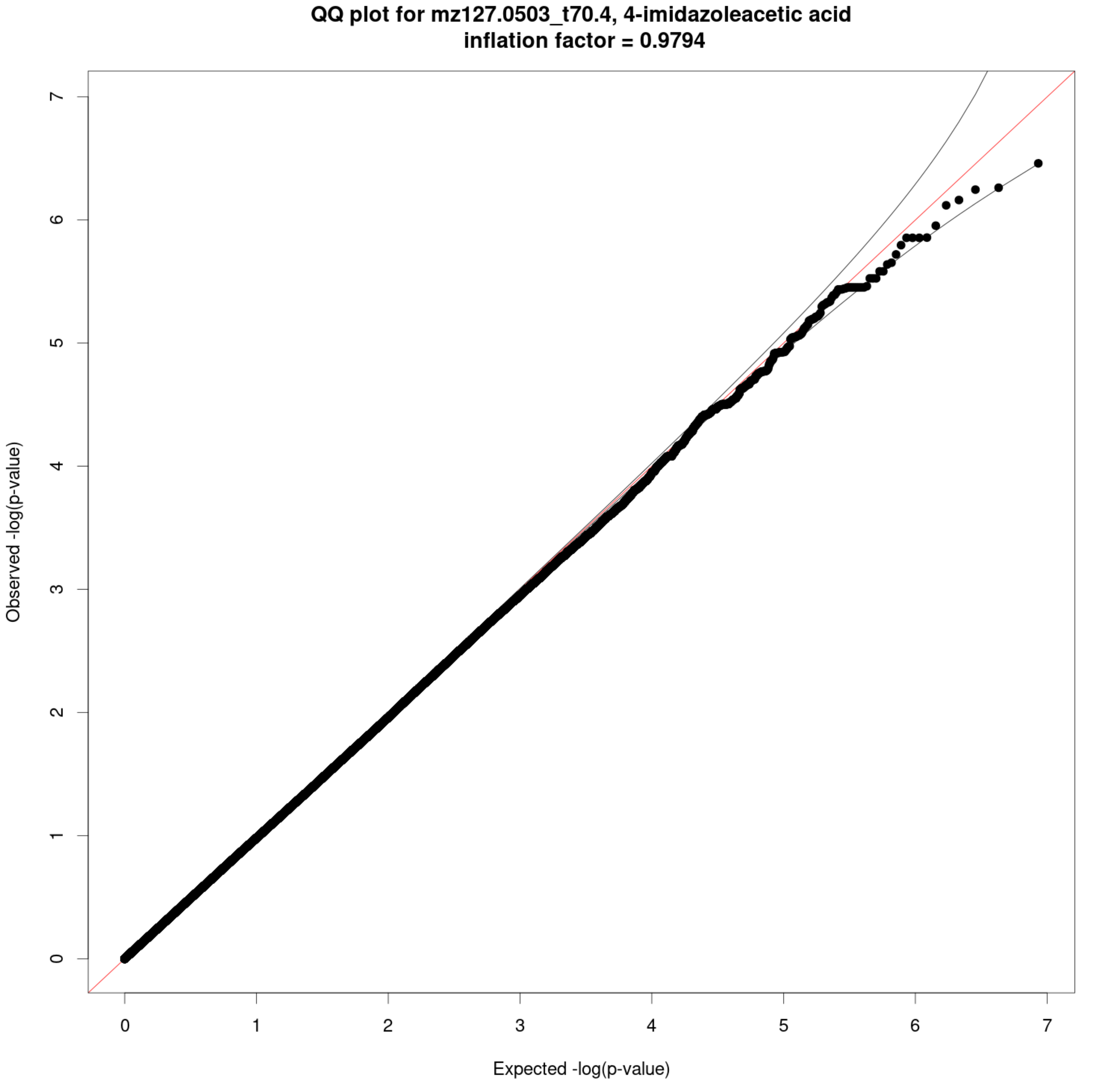
QQ plot for mz127.0503_t70.4, 4-imidazoleacetic acid
inflation factor = 0.9794
Observed -log(p-value)
Expected -log(p-value)
0
1
2
3
4
5
6
7

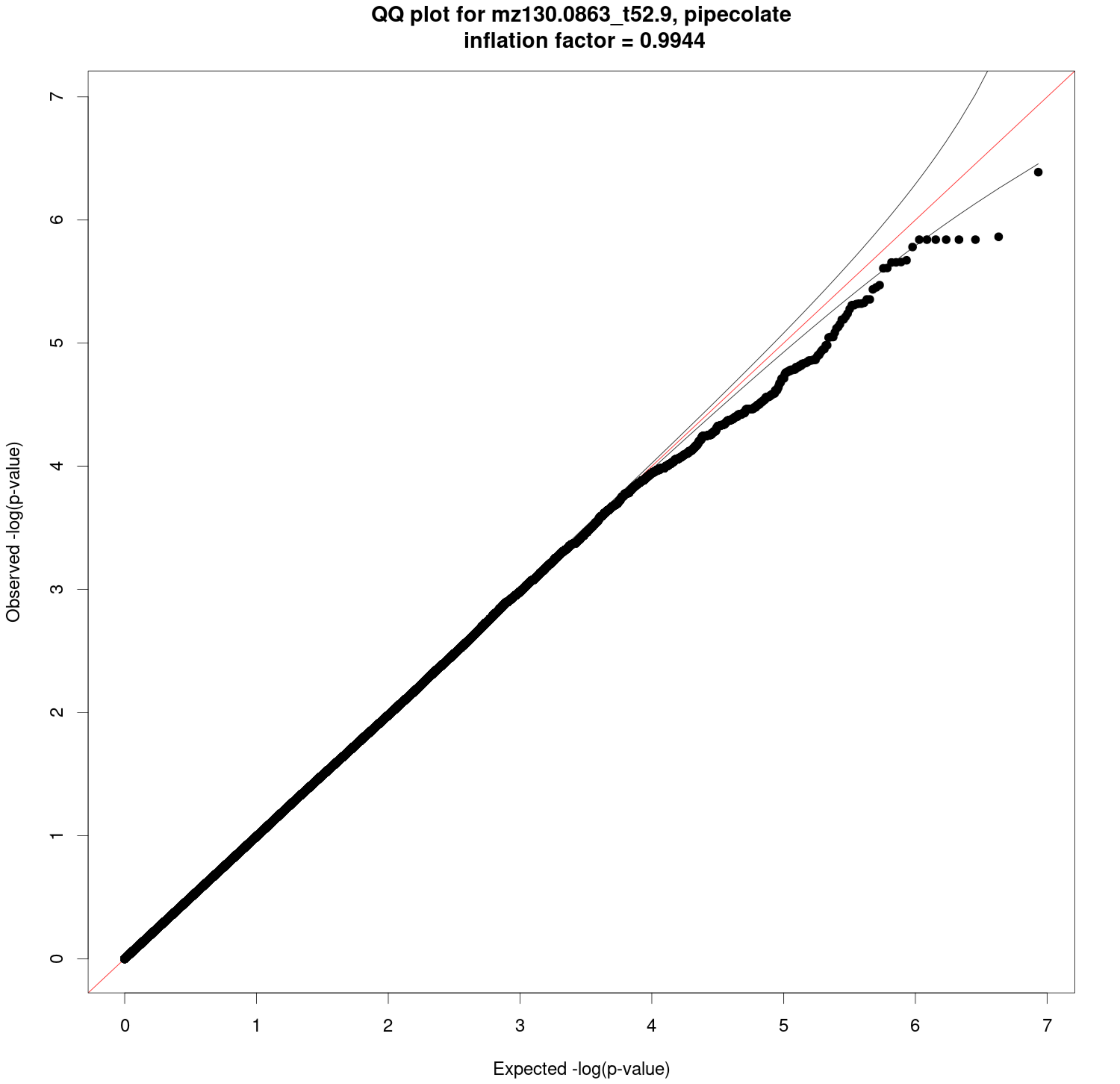
QQ plot for mz130.0863_t52.9, pipecolate
inflation factor = 0.9944
Observed -log(p-value)
Expected -log(p-value)
0
1
2
3
4
5
6
7

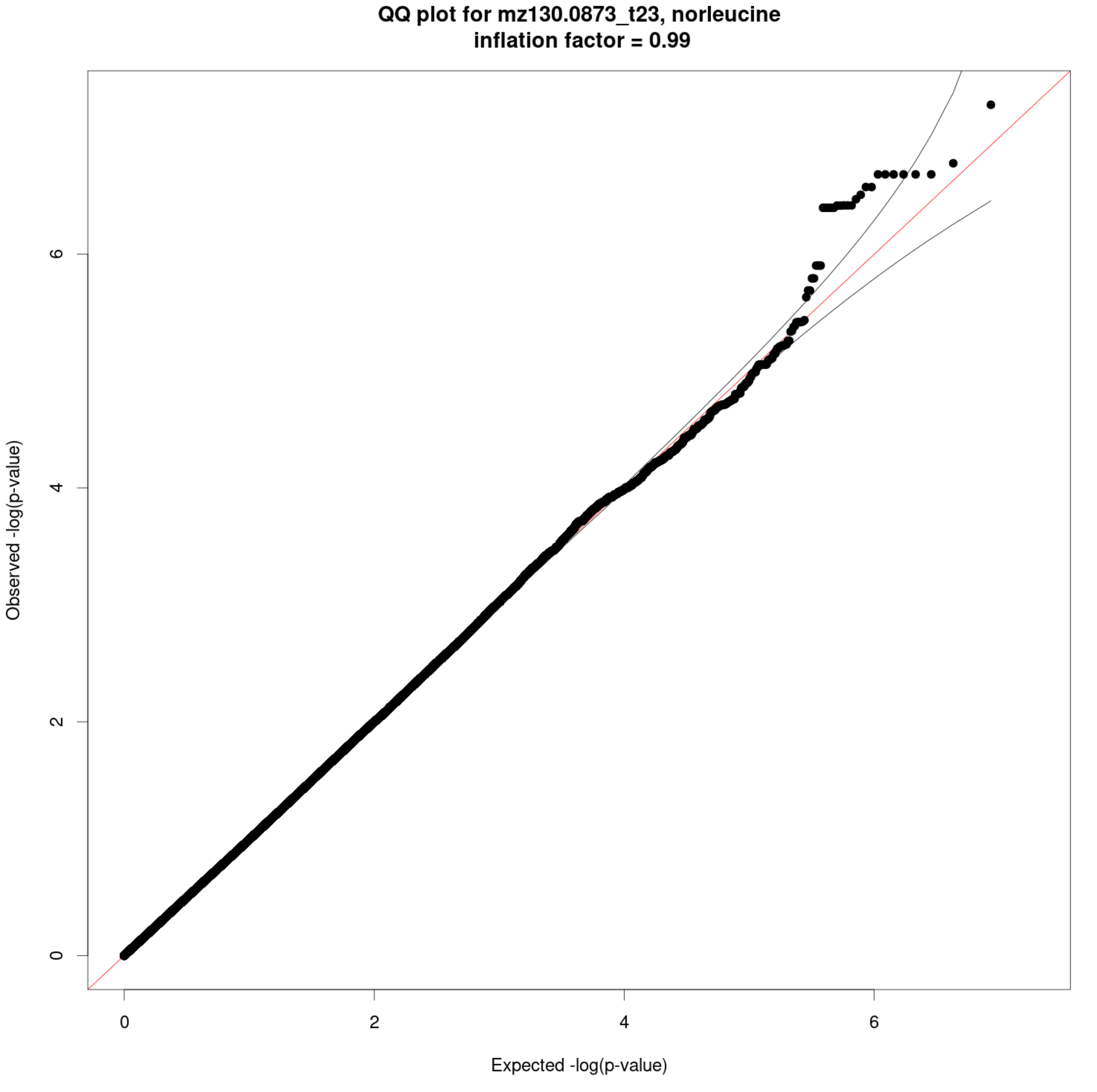
QQ plot for mz130.0873_t23, norleucine
inflation factor = 0.99
Observed -log(p-value)
Expected -log(p-value)
0
2
4
6
0
2
4
6

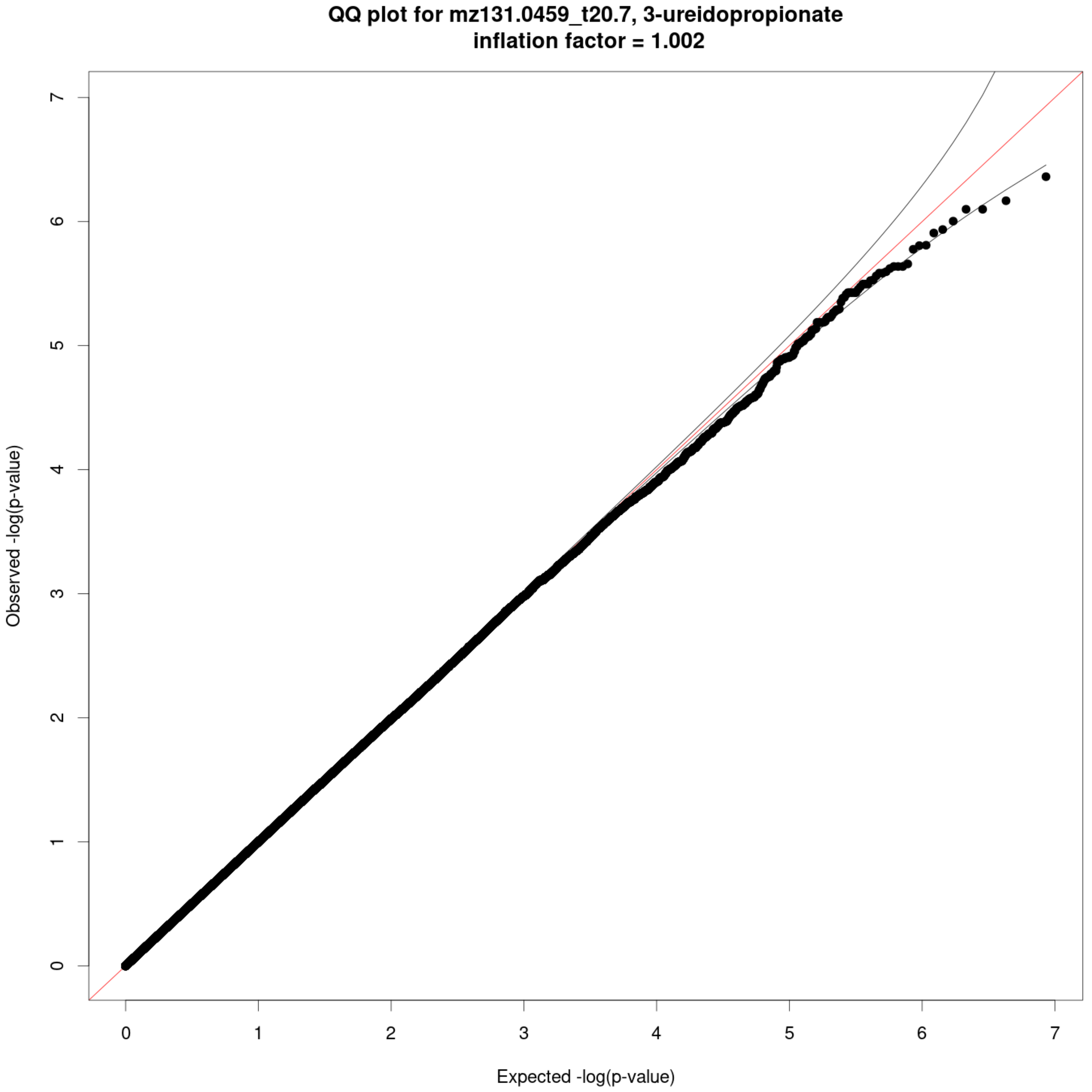
QQ plot for mz131.0459_t20.7, 3-ureidopropionate
inflation factor = 1.002
Observed -log(p-value)
Expected -log(p-value)
0
1
2
3
4
5
6
7

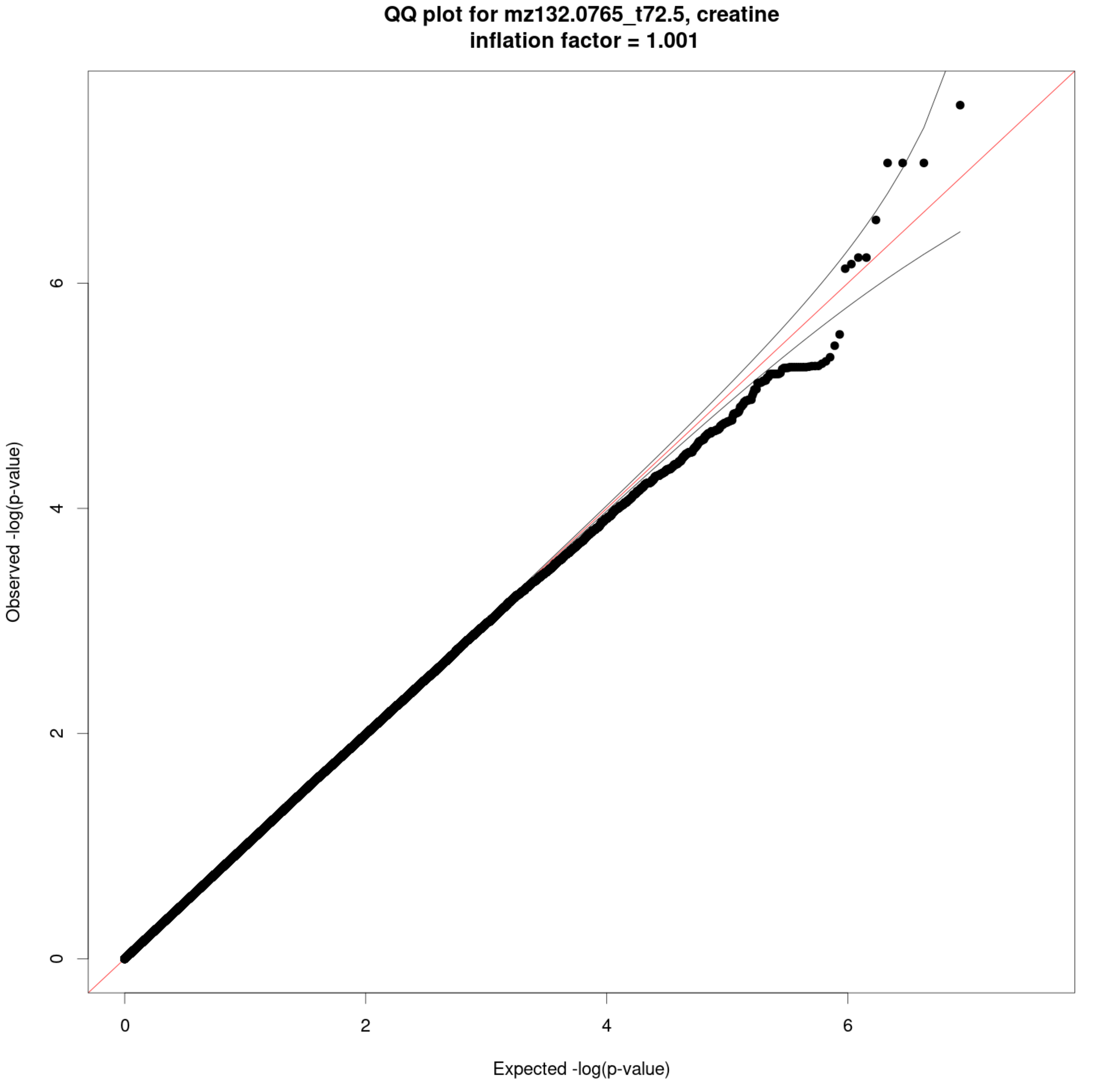
QQ plot for mz132.0765_t72.5, creatine
inflation factor = 1.001
Observed -log(p-value)
Expected -log(p-value)
0
2
4
6
0
2
4
6

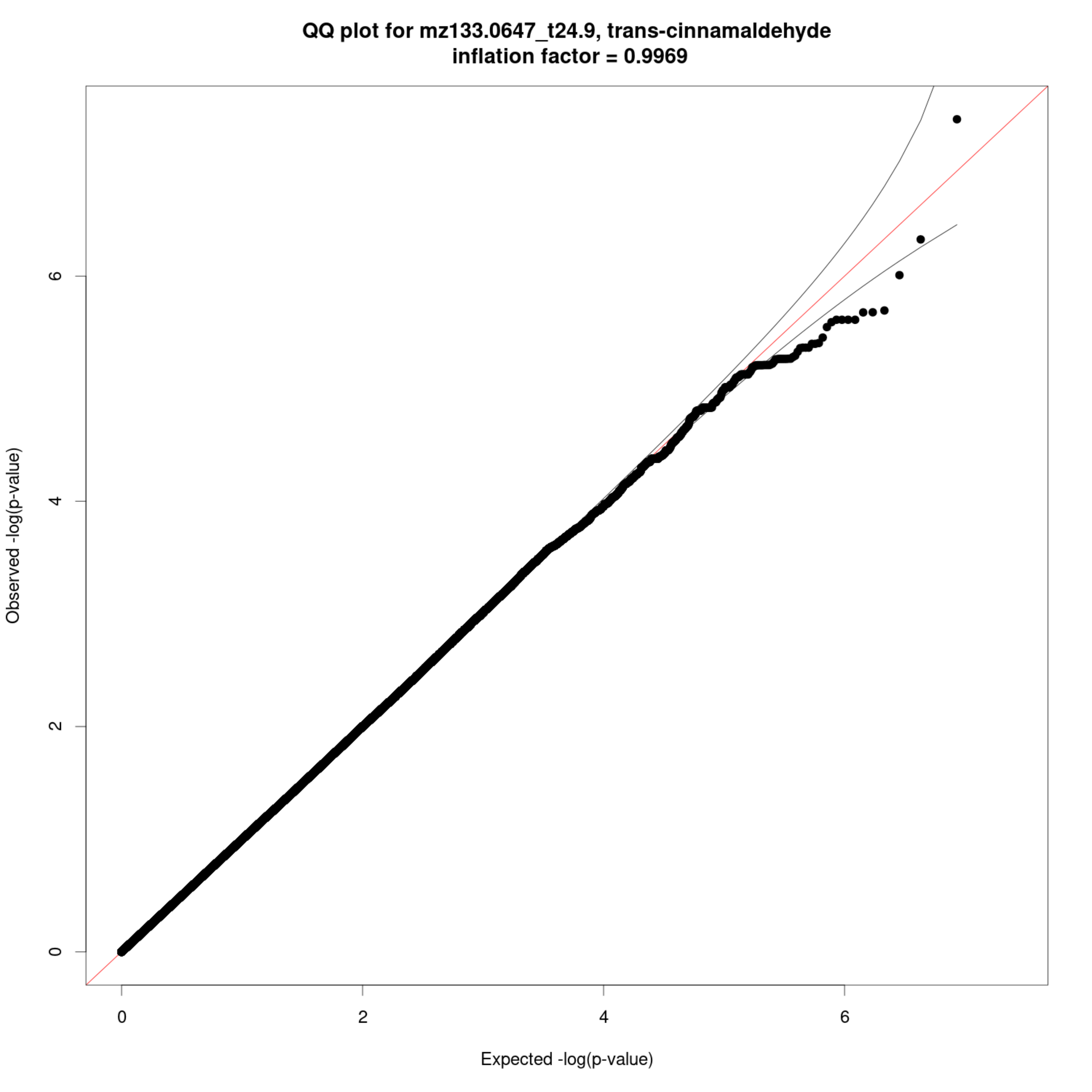
QQ plot for mz133.0647_t24.9, trans-cinnamaldehyde
inflation factor = 0.9969
Observed -log(p-value)
Expected -log(p-value)
0
2
4
6

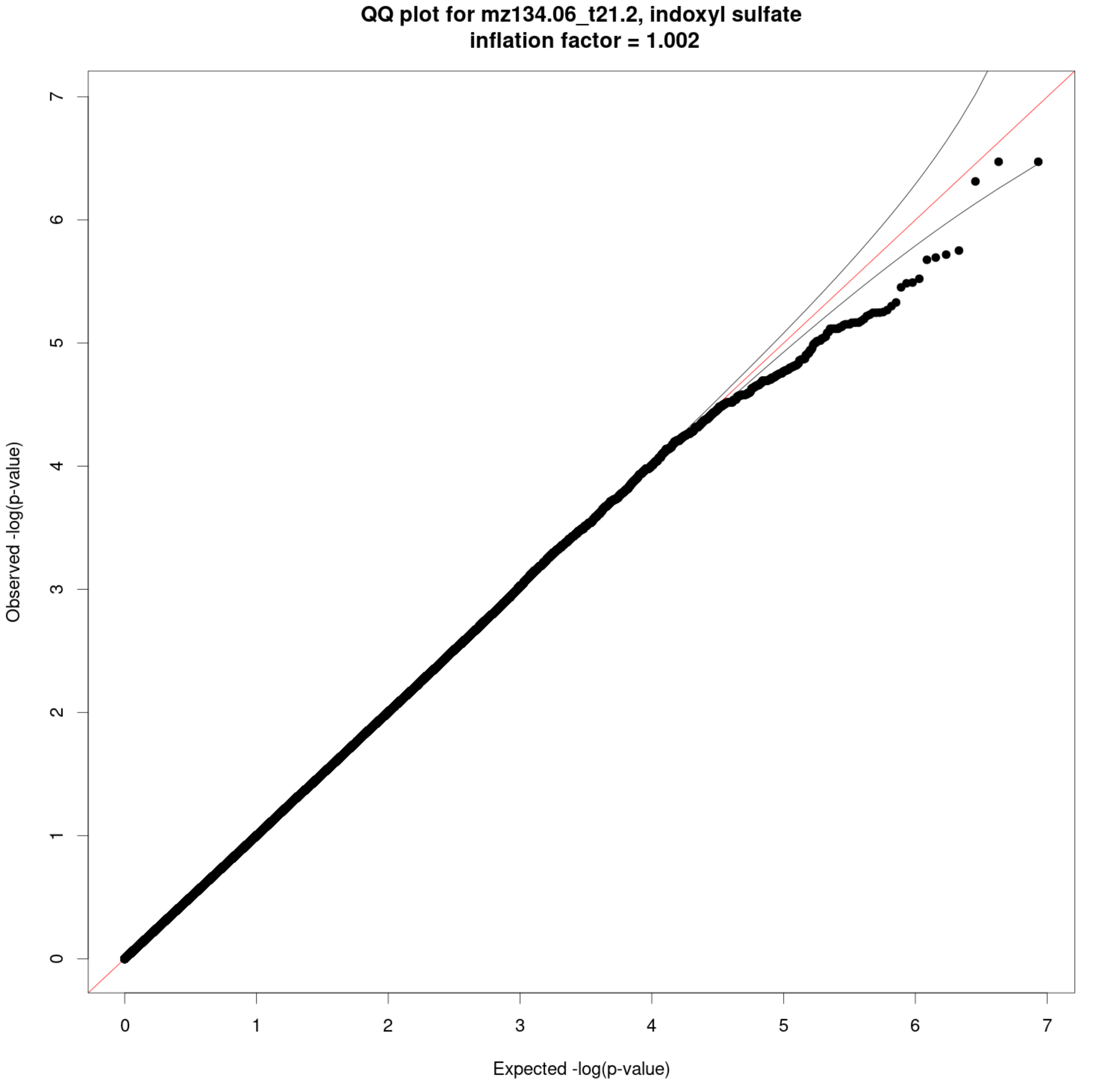
QQ plot for mz134.06_t21.2, indoxyl sulfate
inflation factor = 1.002
Observed -log(p-value)
Expected -log(p-value)
0
1
2
3
4
5
6
7

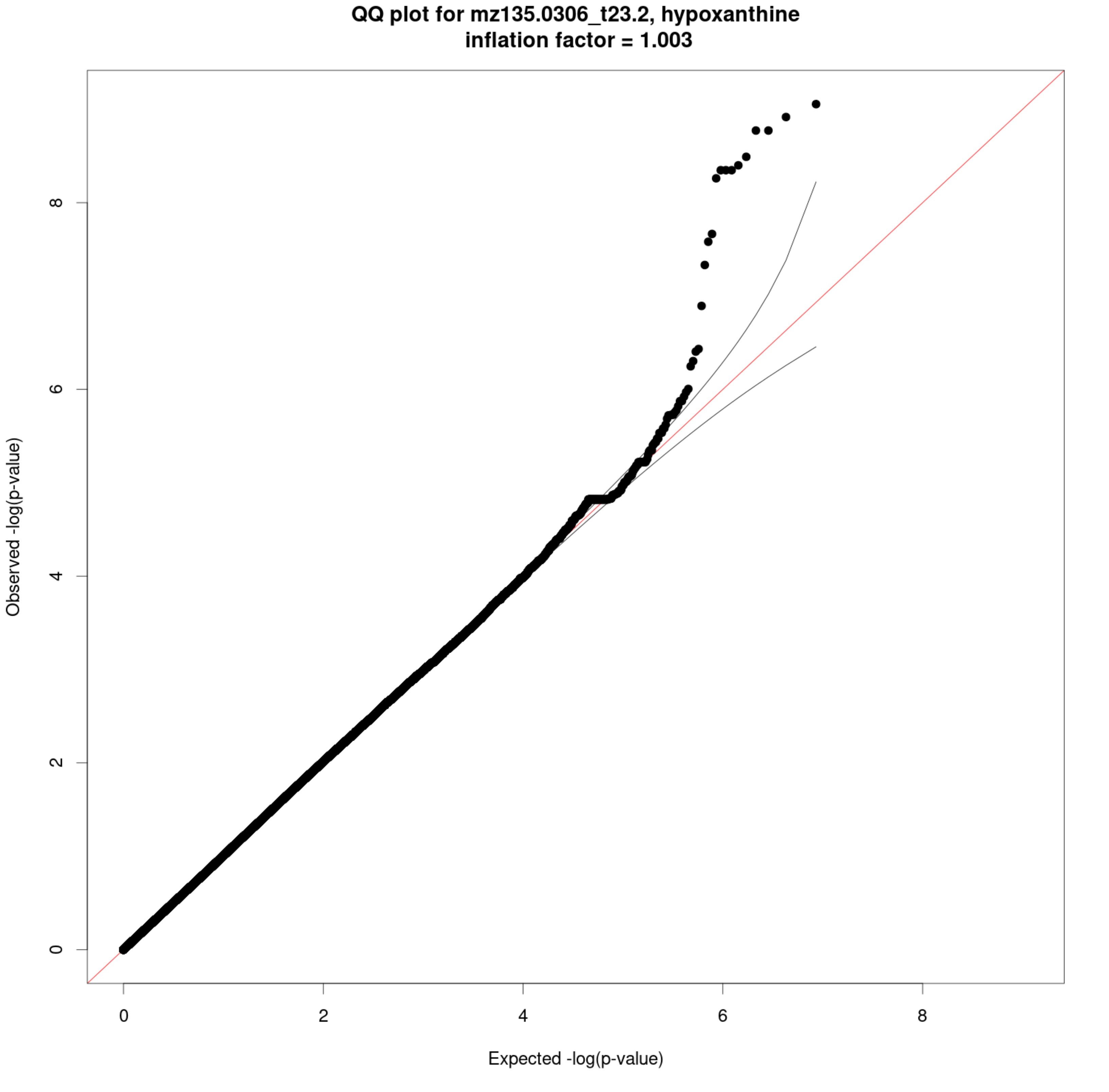
QQ plot for mz135.0306_t23.2, hypoxanthine
inflation factor = 1.003
Observed -log(p-value)
Expected -log(p-value)
0
2
4
6
8

QQ plot for mz136.0428_t56.3, homocysteine
inflation factor = 0.9928

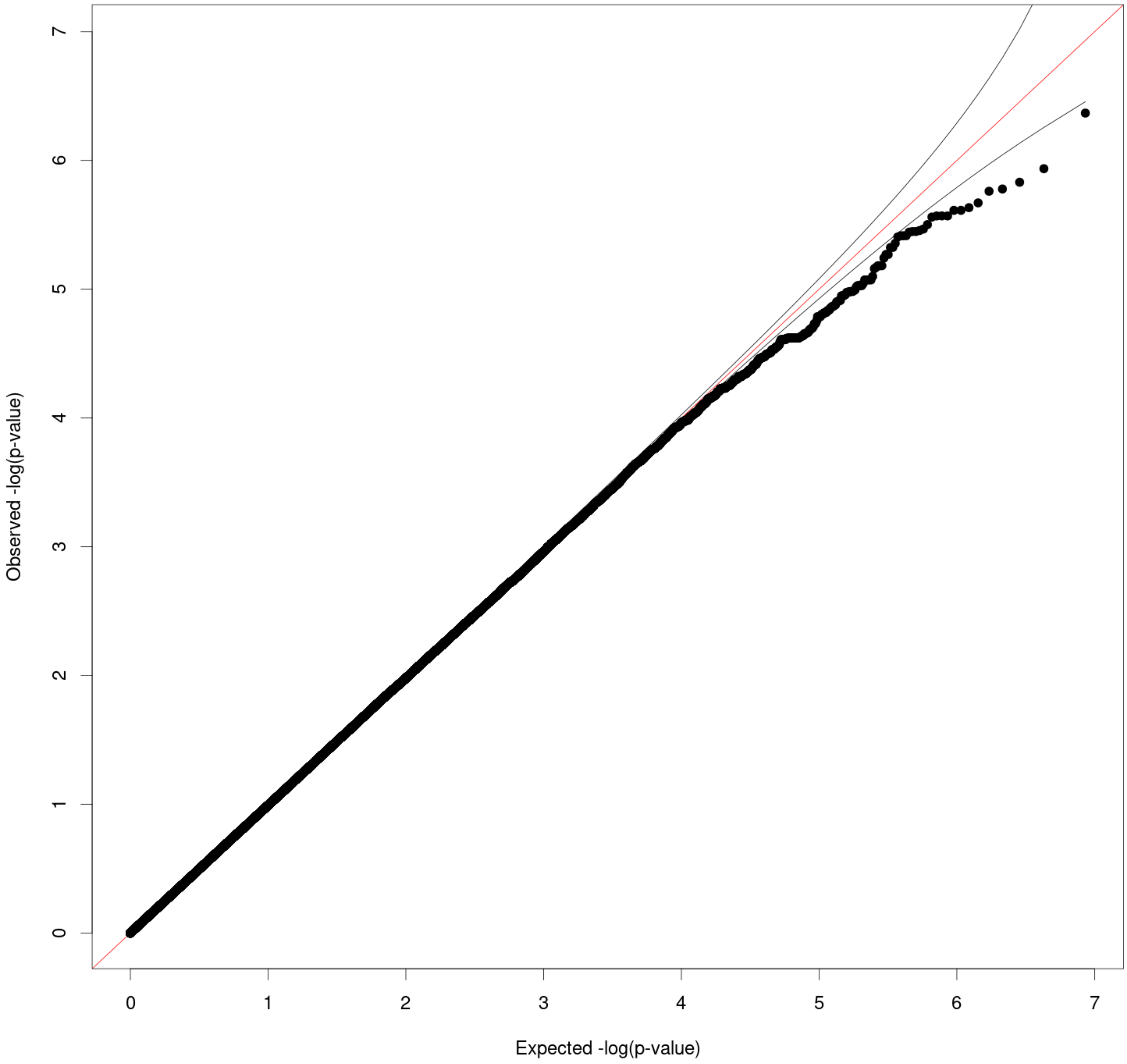

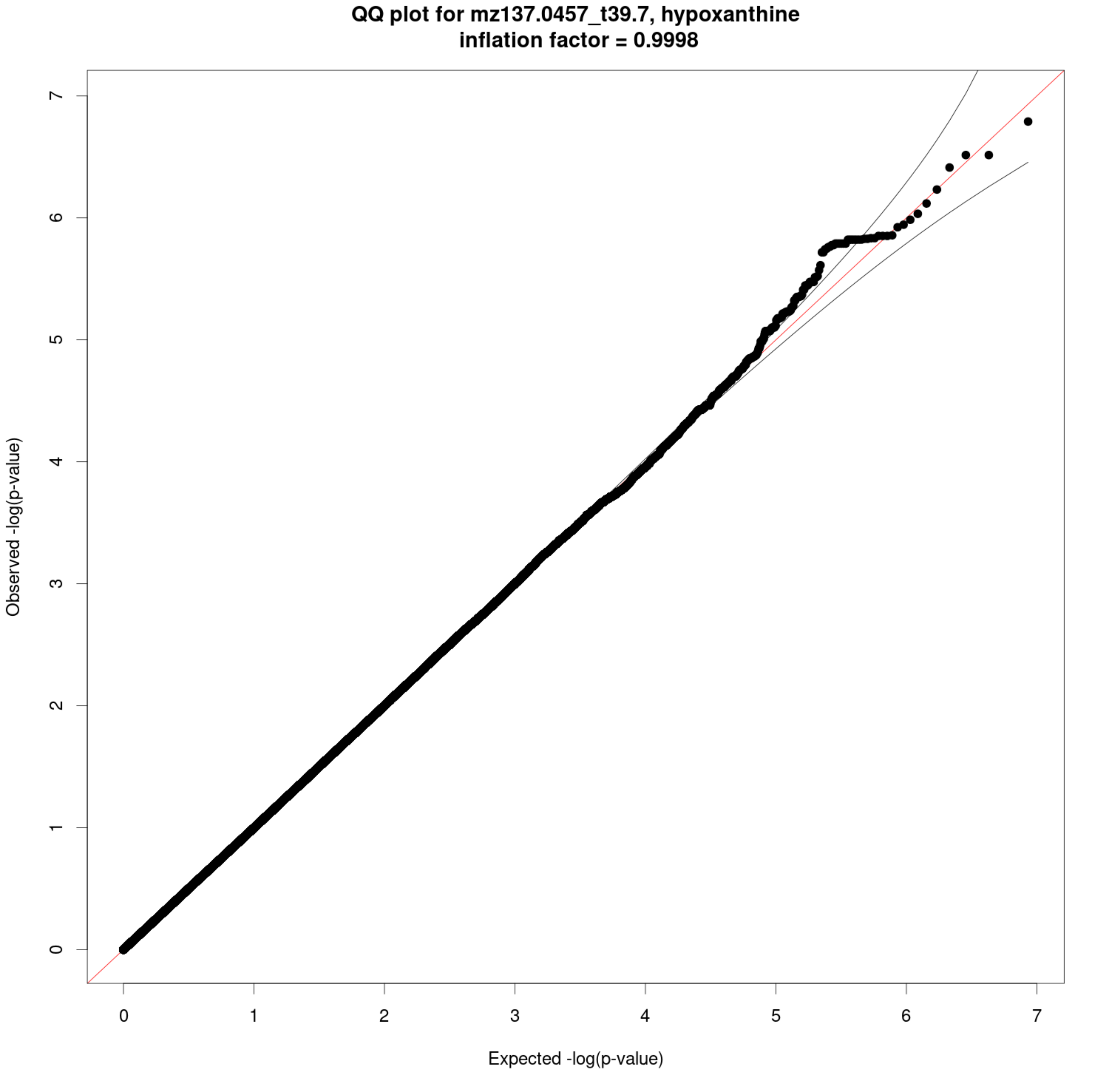
QQ plot for mz137.0457_t39.7, hypoxanthine
inflation factor = 0.9998
Observed -log(p-value)
Expected -log(p-value)
0
1
2
3
4
5
6
7

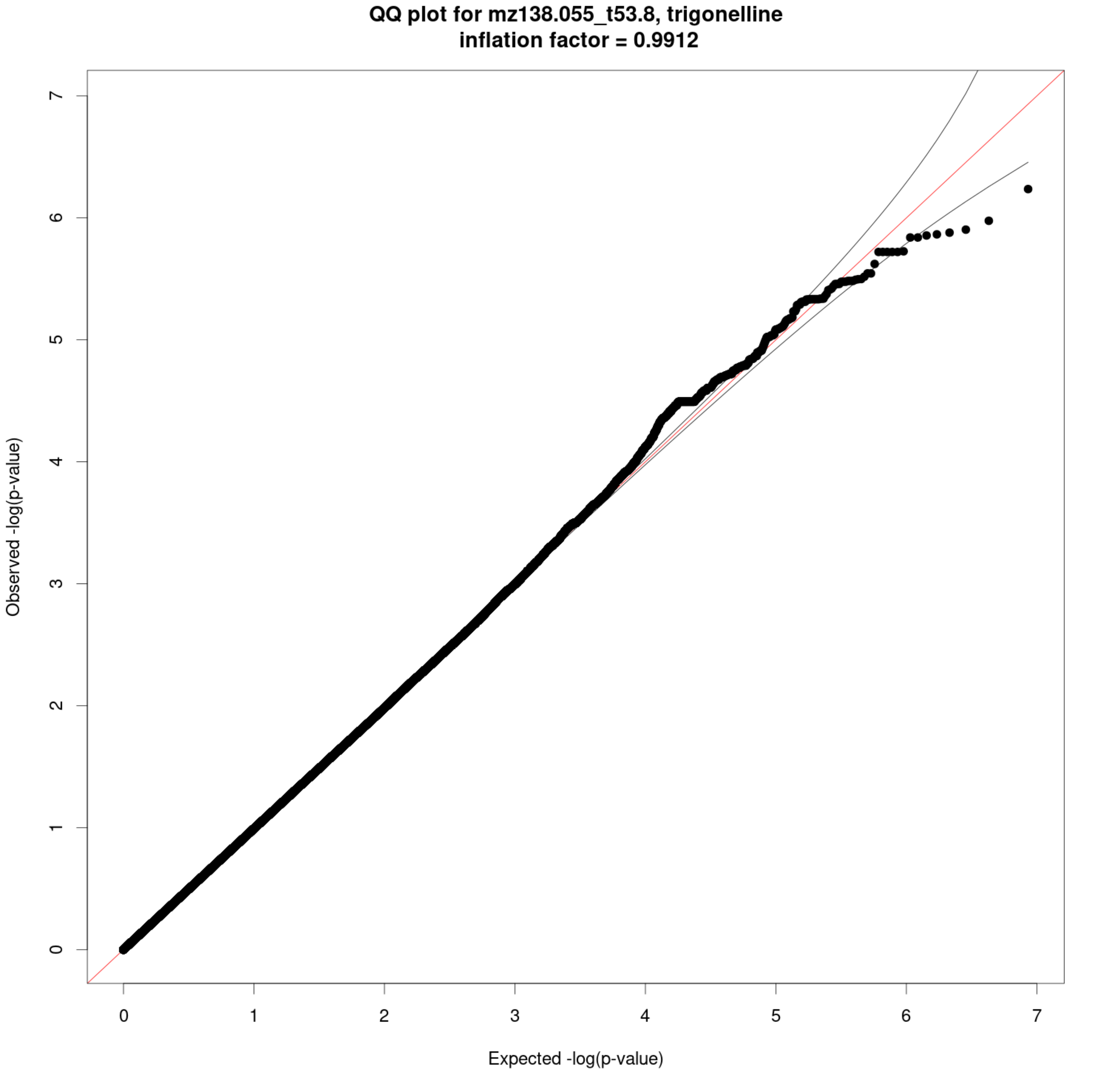
QQ plot for mz138.055_t53.8, trigonelline
inflation factor = 0.9912
Observed -log(p-value)
Expected -log(p-value)
0
1
2
3
4
5
6
7

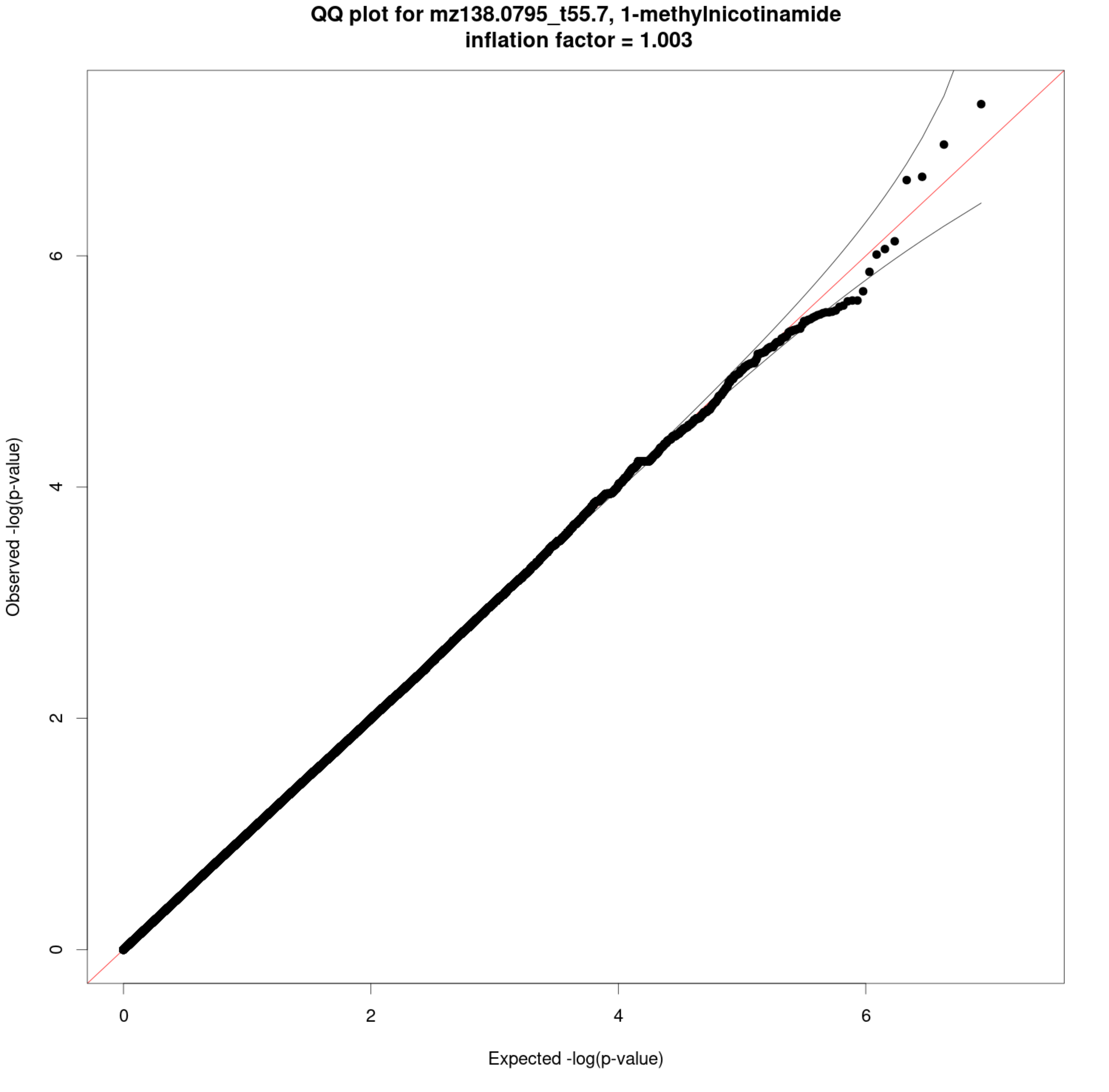
QQ plot for mz138.0795_t55.7, 1-methylnicotinamide
inflation factor = 1.003
Observed -log(p-value)
Expected -log(p-value)
0
2
4
6
0
2
4
6

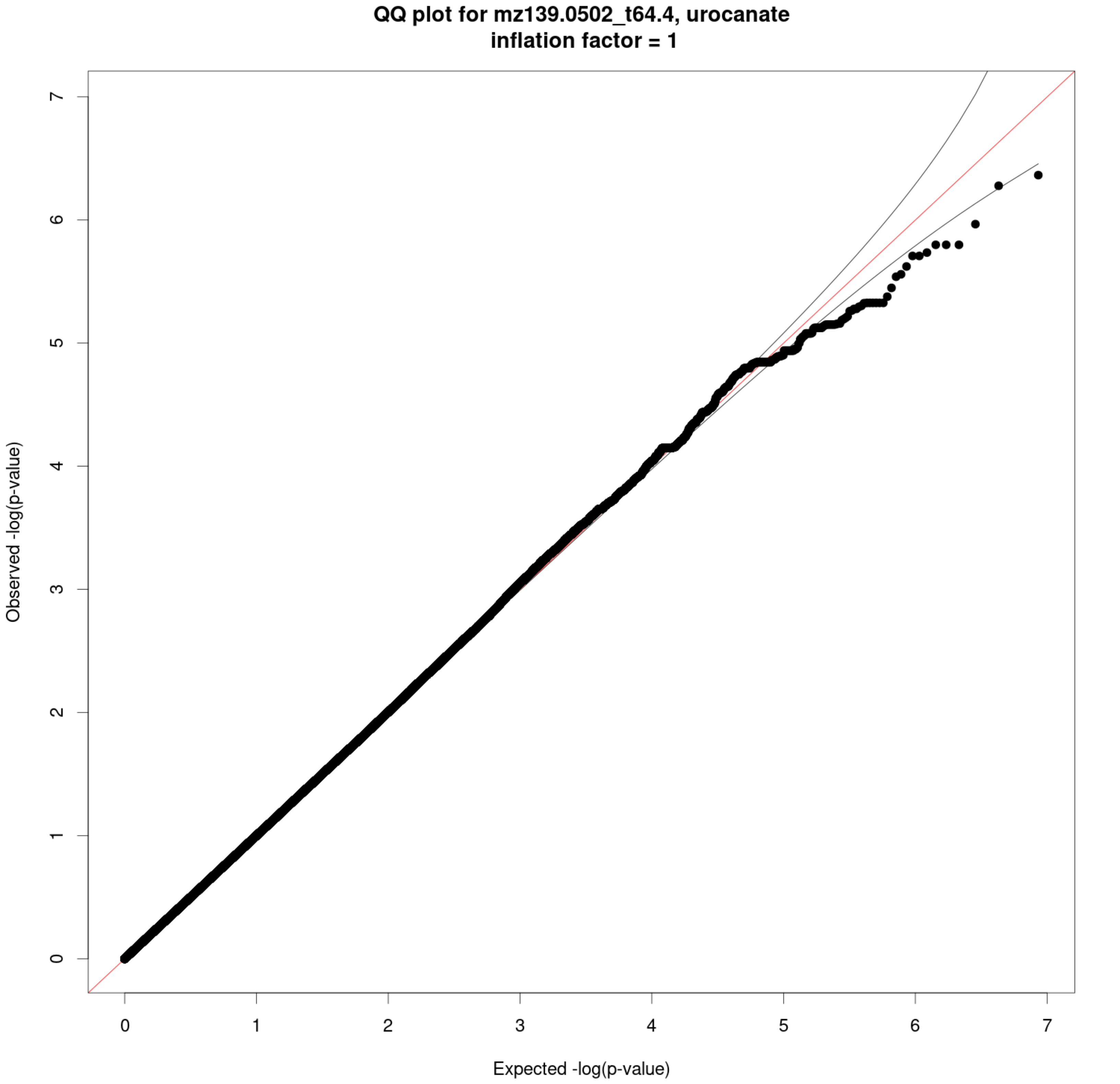
QQ plot for mz139.0502_t64.4, urocanate
inflation factor = 1
Observed -log(p-value)
Expected -log(p-value)
0
1
2
3
4
5
6
7

QQ plot for mz140.0118_t36.8, ethanolamine phosphate
inflation factor = 0.9929

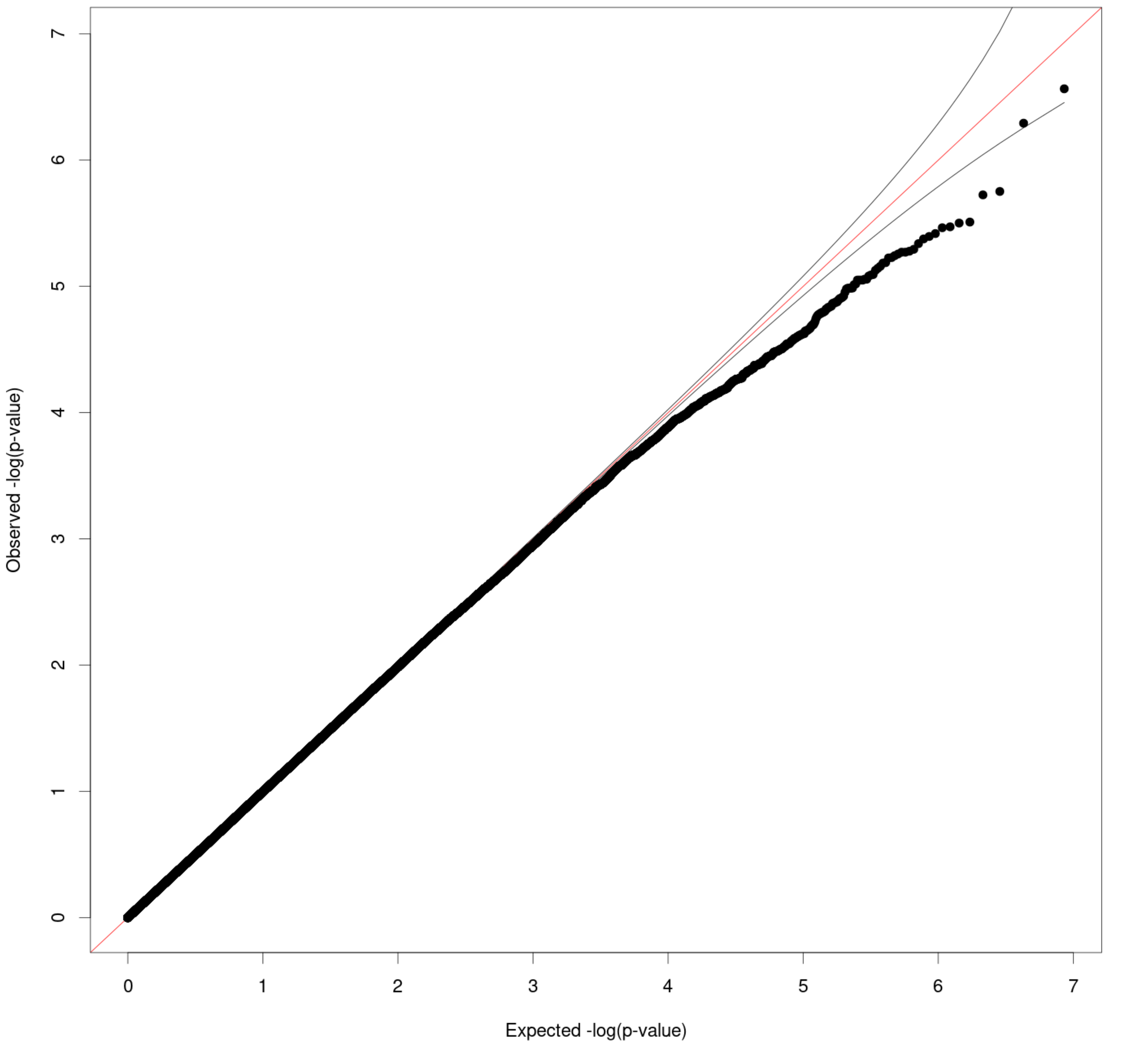

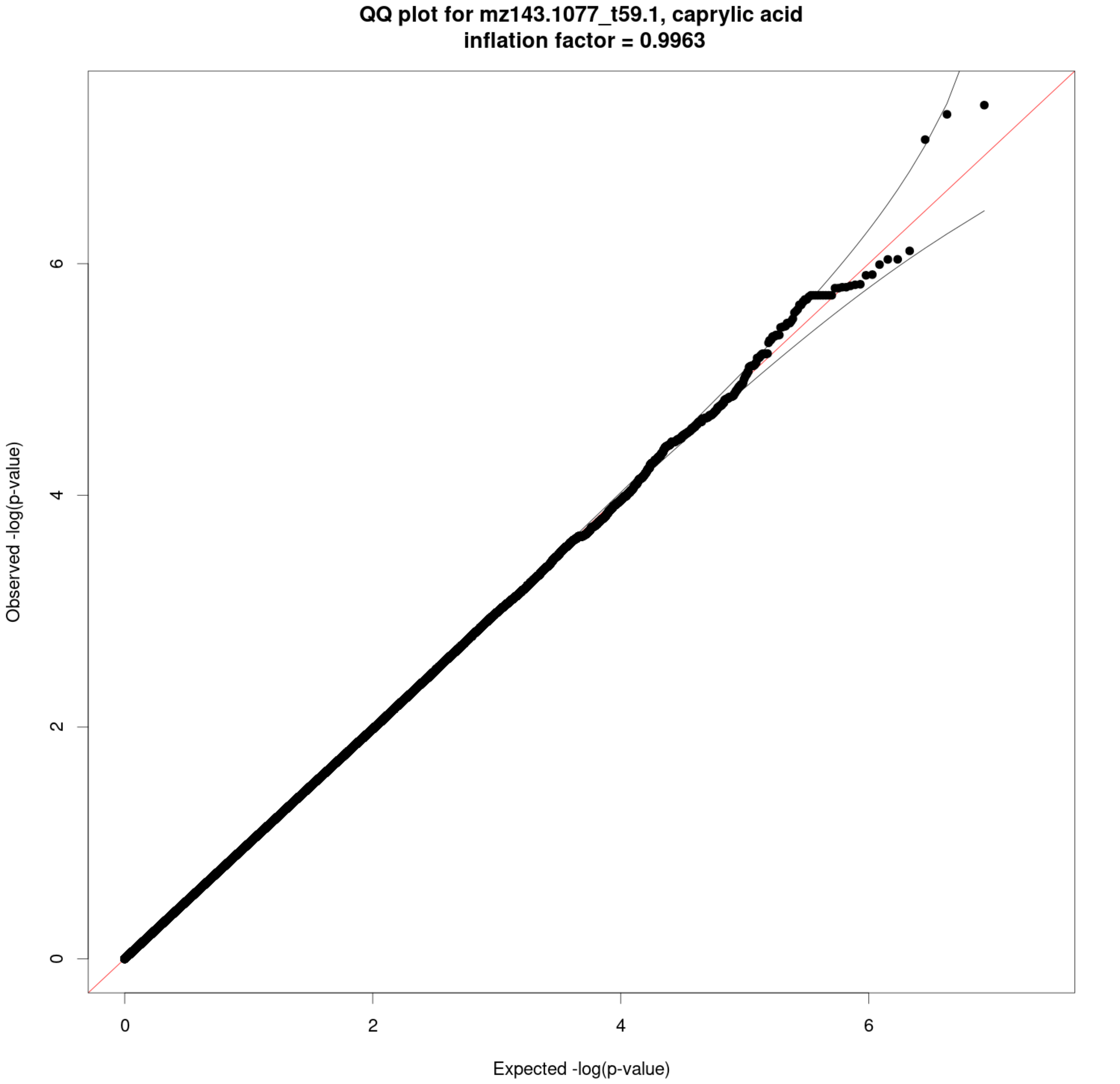
QQ plot for mz143.1077_t59.1, caprylic acid
inflation factor = 0.9963
Observed -log(p-value)
Expected -log(p-value)
0
2
4
6

QQ plot for mz144.0807_t46.2, 1-naphthylamine
inflation factor = 1.001

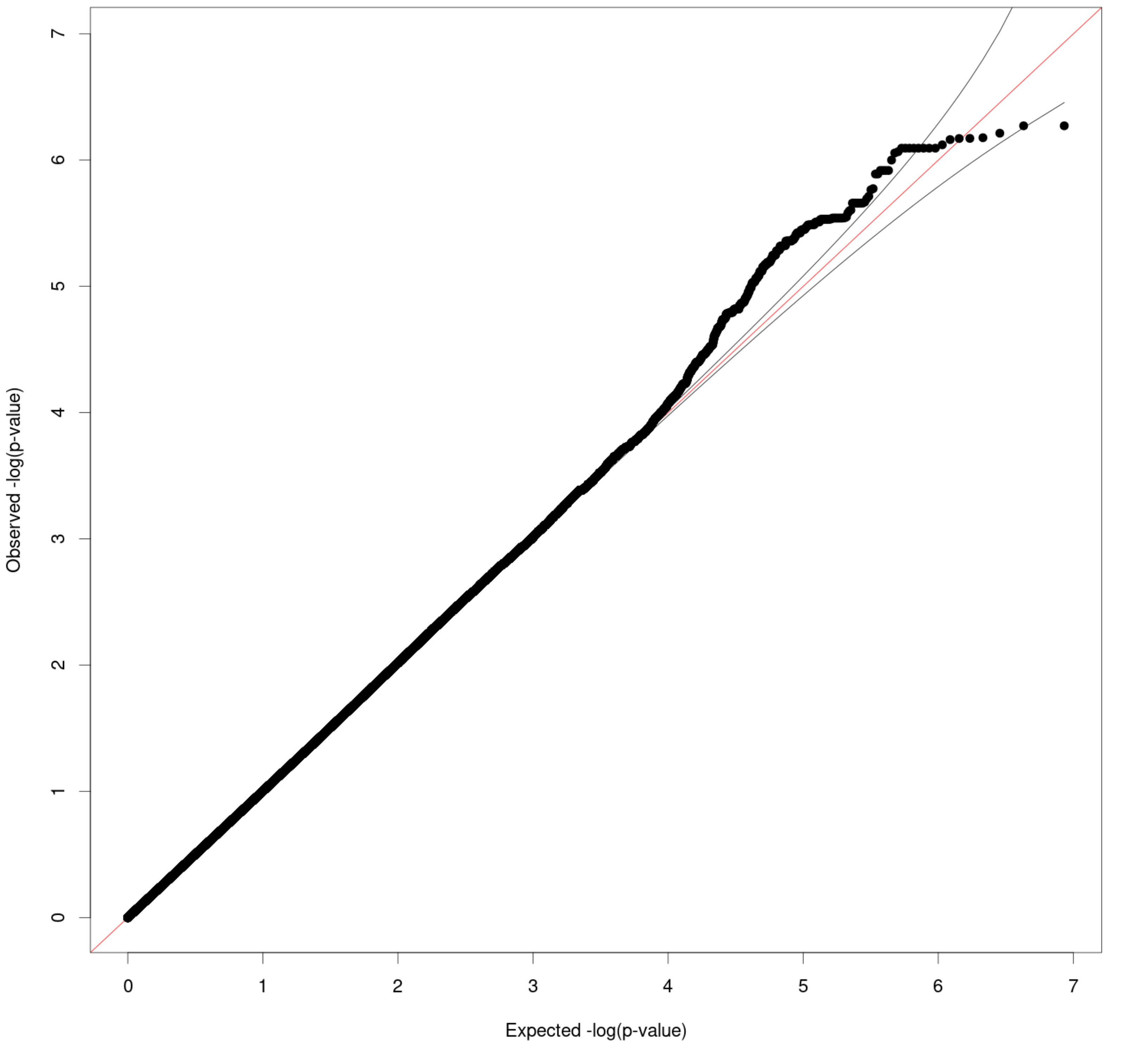

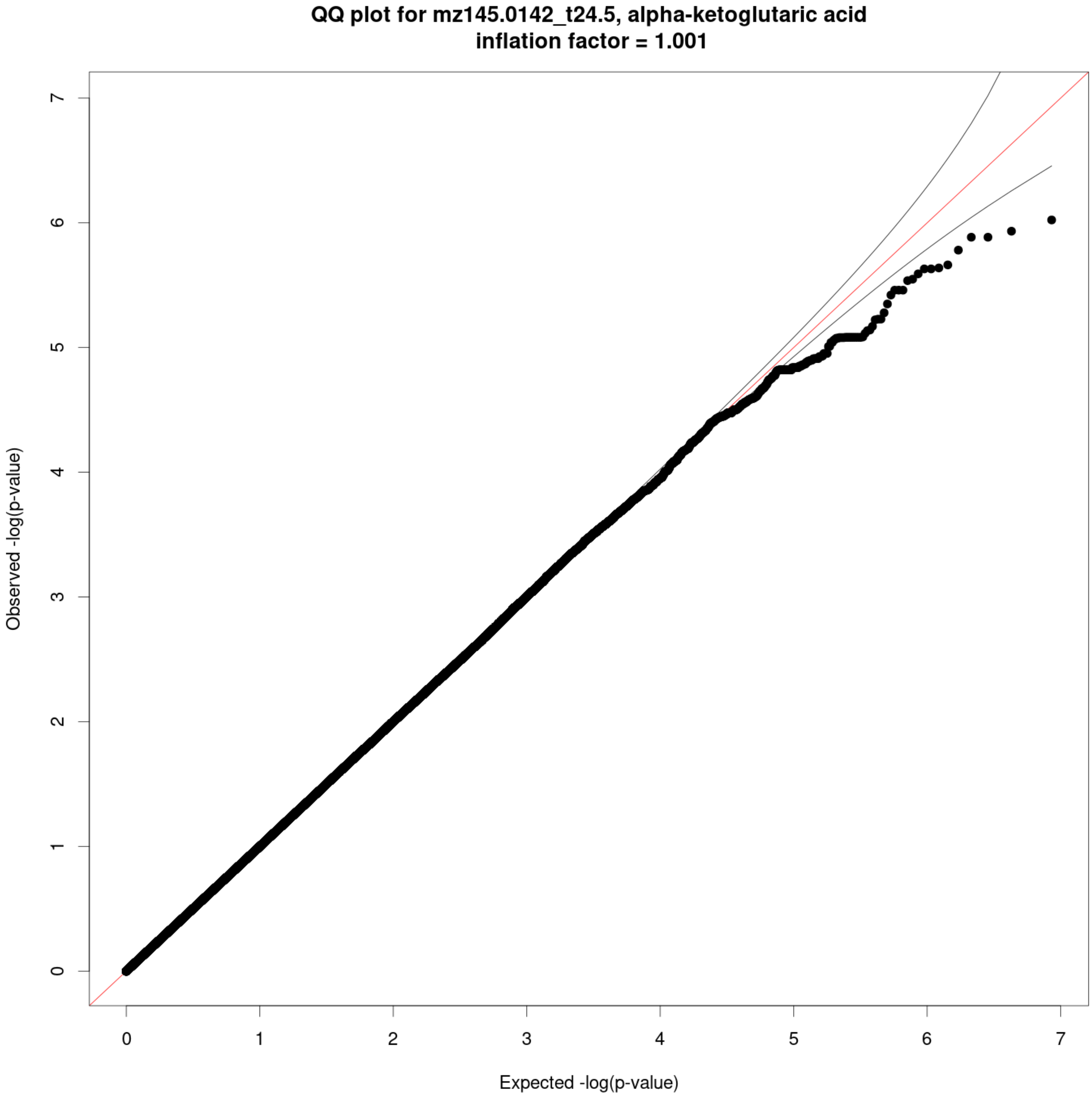
QQ plot for mz145.0142_t24.5, alpha-ketoglutaric acid
inflation factor = 1.001
Observed -log(p-value)
Expected -log(p-value)
0
1
2
3
4
5
6
7

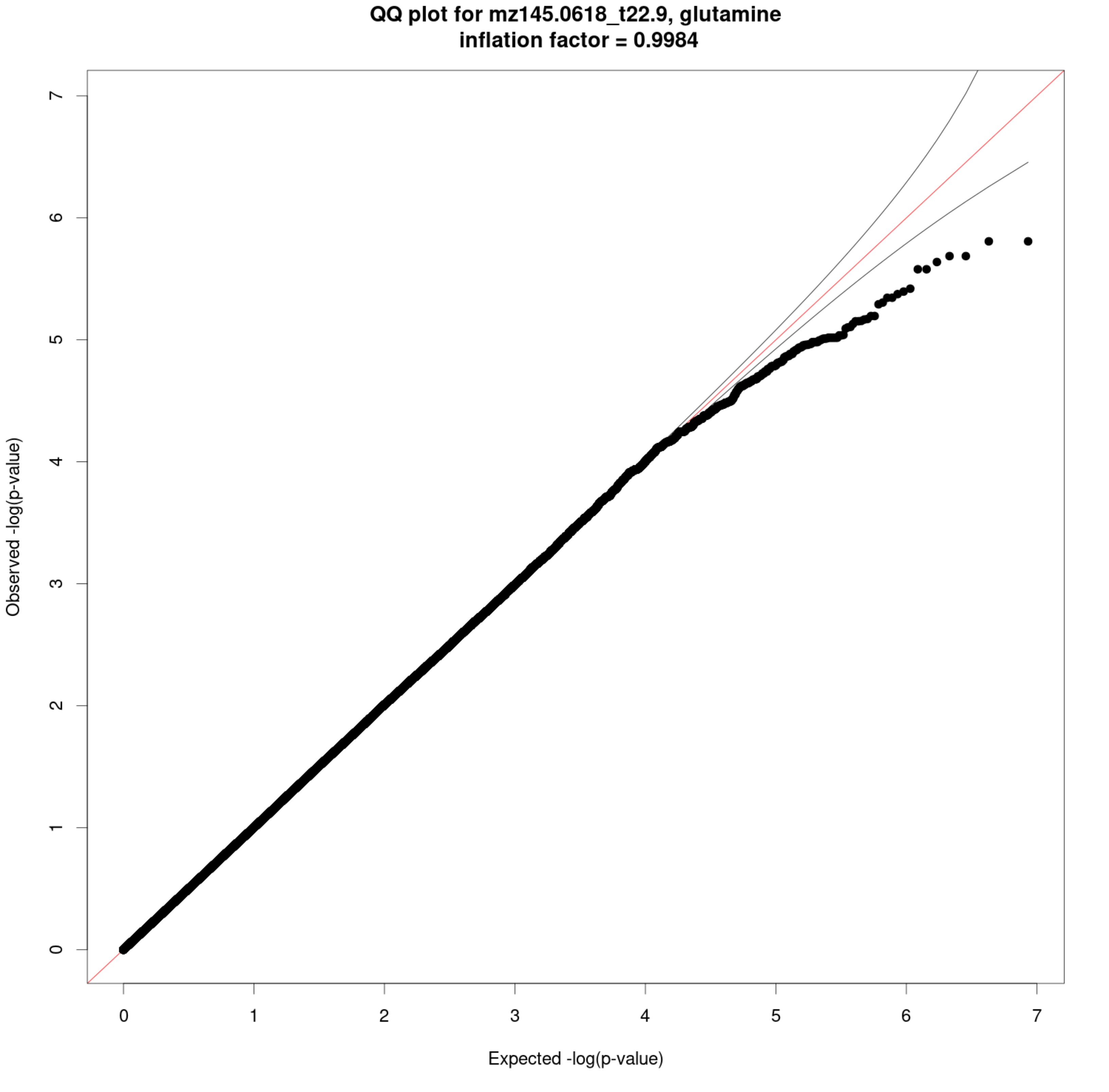
QQ plot for mz145.0618_t22.9, glutamine
inflation factor = 0.9984
Observed -log(p-value)
Expected -log(p-value)
0
1
2
3
4
5
6
7

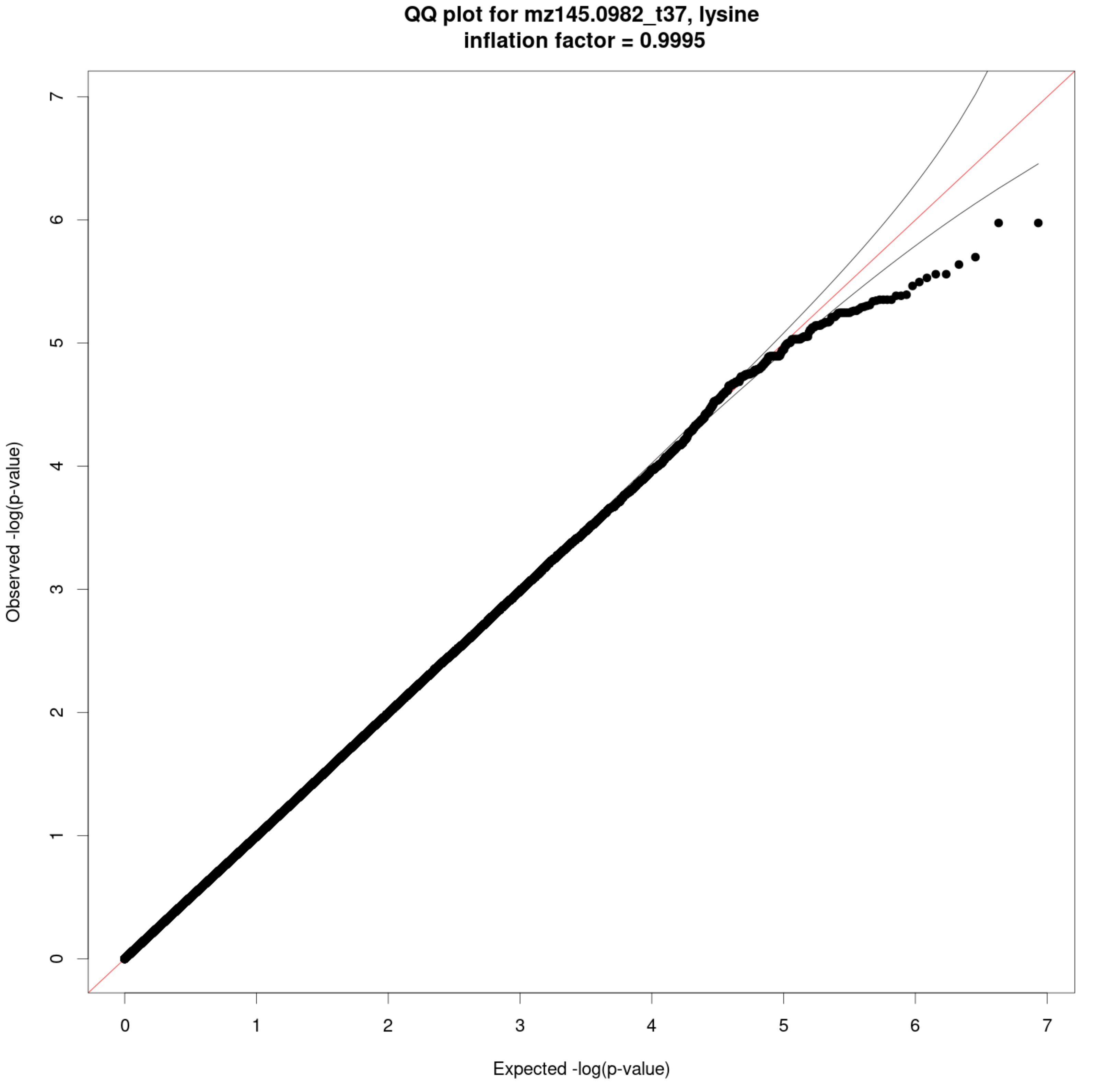
QQ plot for mz145.0982_t37, lysine
inflation factor = 0.9995
Observed -log(p-value)
Expected -log(p-value)
0
1
2
3
4
5
6
7

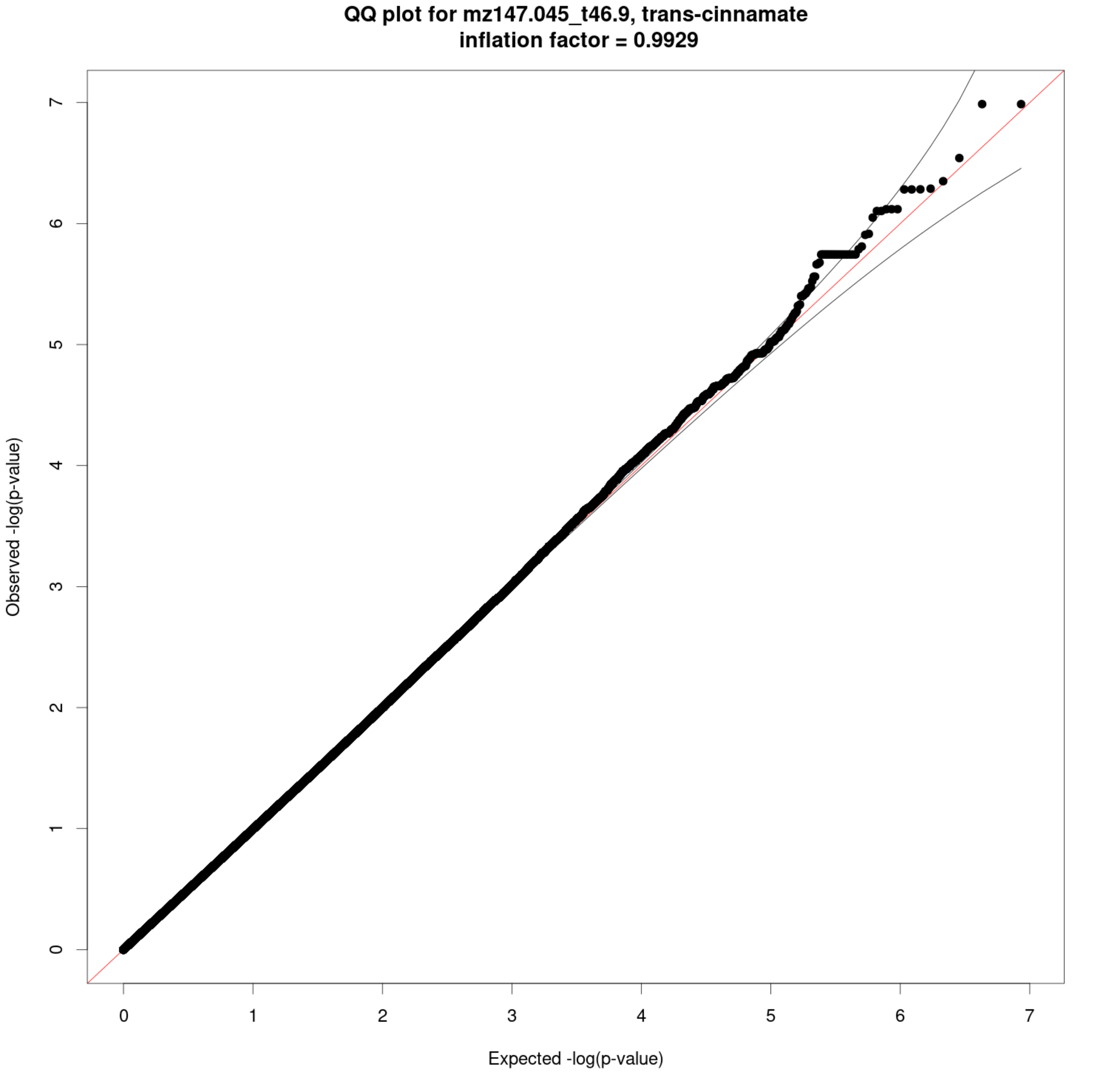
QQ plot for mz147.045_t46.9, trans-cinnamate
inflation factor = 0.9929
Observed -log(p-value)
Expected -log(p-value)
0
1
2
3
4
5
6
7

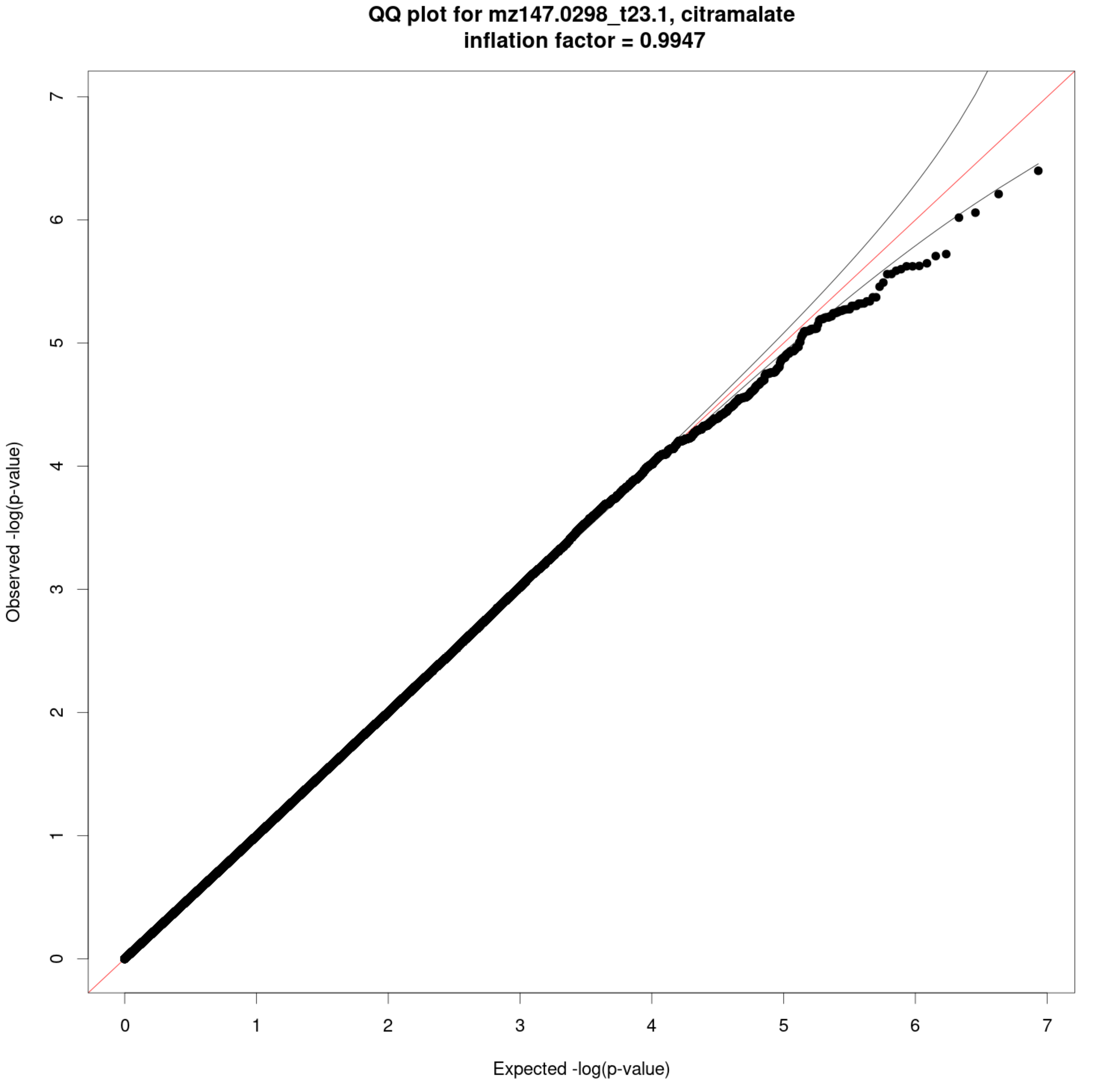
QQ plot for mz147.0298_t23.1, citramalate
inflation factor = 0.9947
Observed -log(p-value)
Expected -log(p-value)
0
1
2
3
4
5
6
7

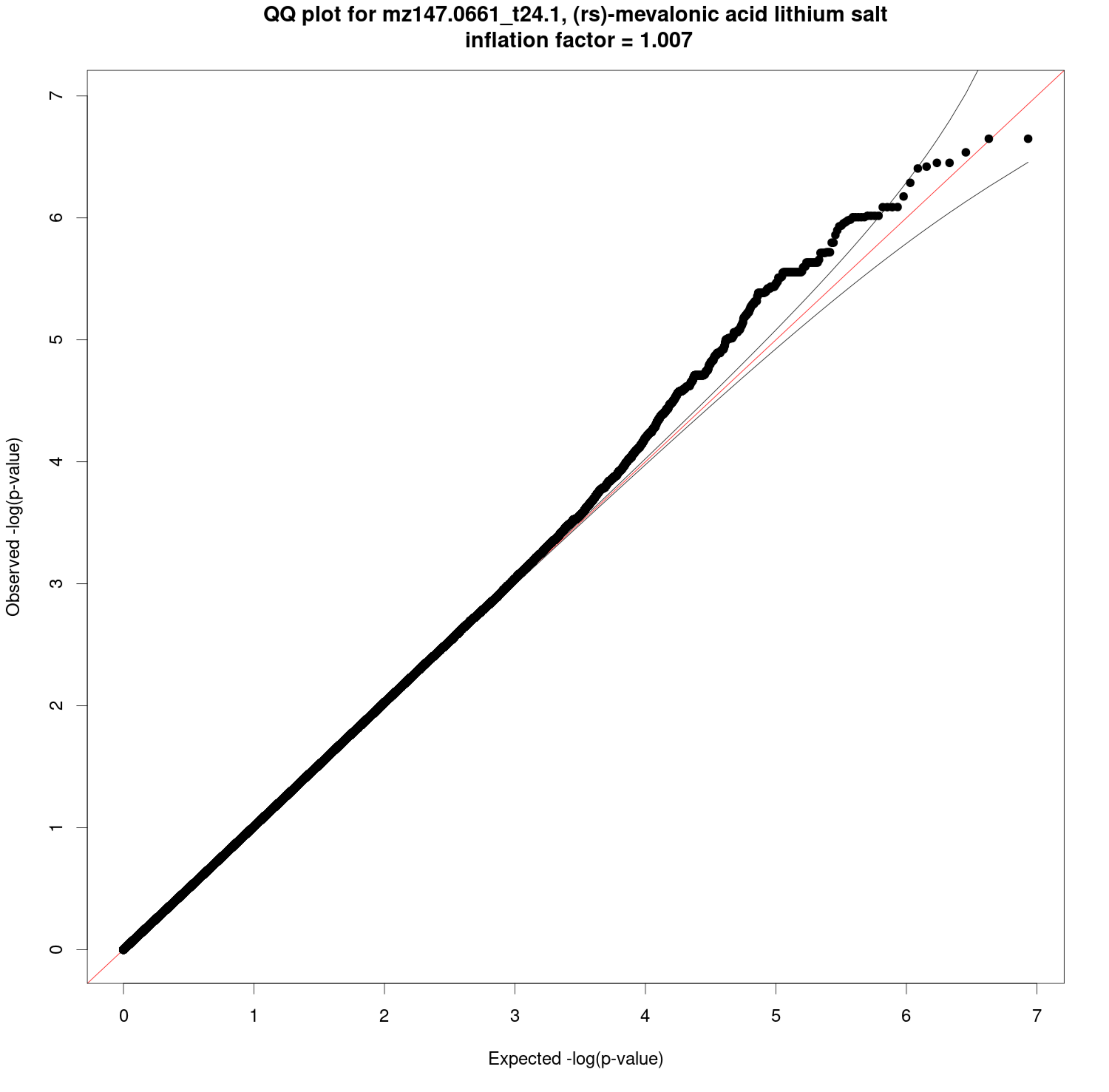
QQ plot for mz147.0661_t24.1, (rs)-mevalonic acid lithium salt
inflation factor = 1.007
Observed -log(p-value)
Expected -log(p-value)
0
1
2
3
4
5
6
7

QQ plot for mz147.0764_t81.7, glutamine
inflation factor = 0.9966

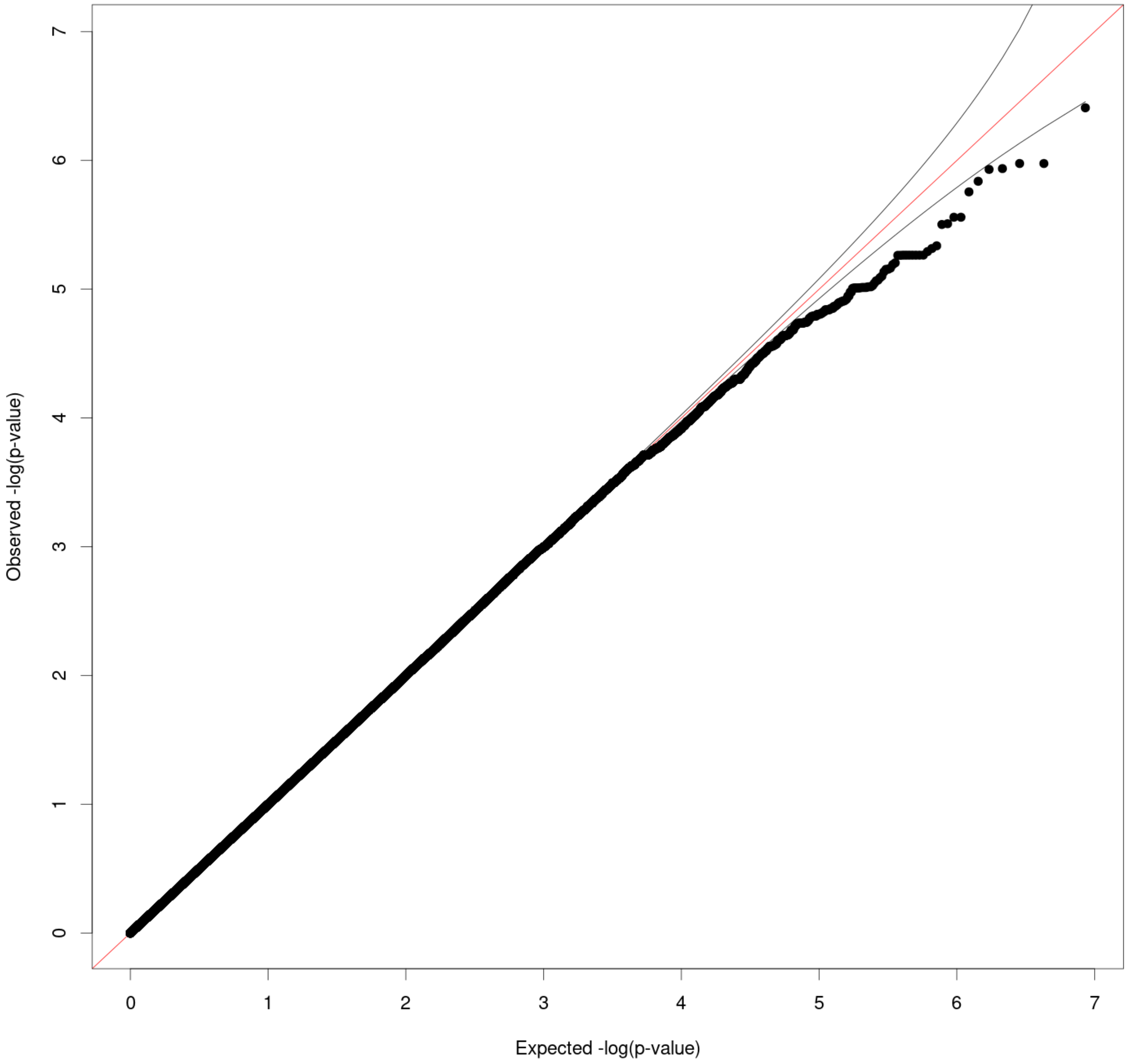

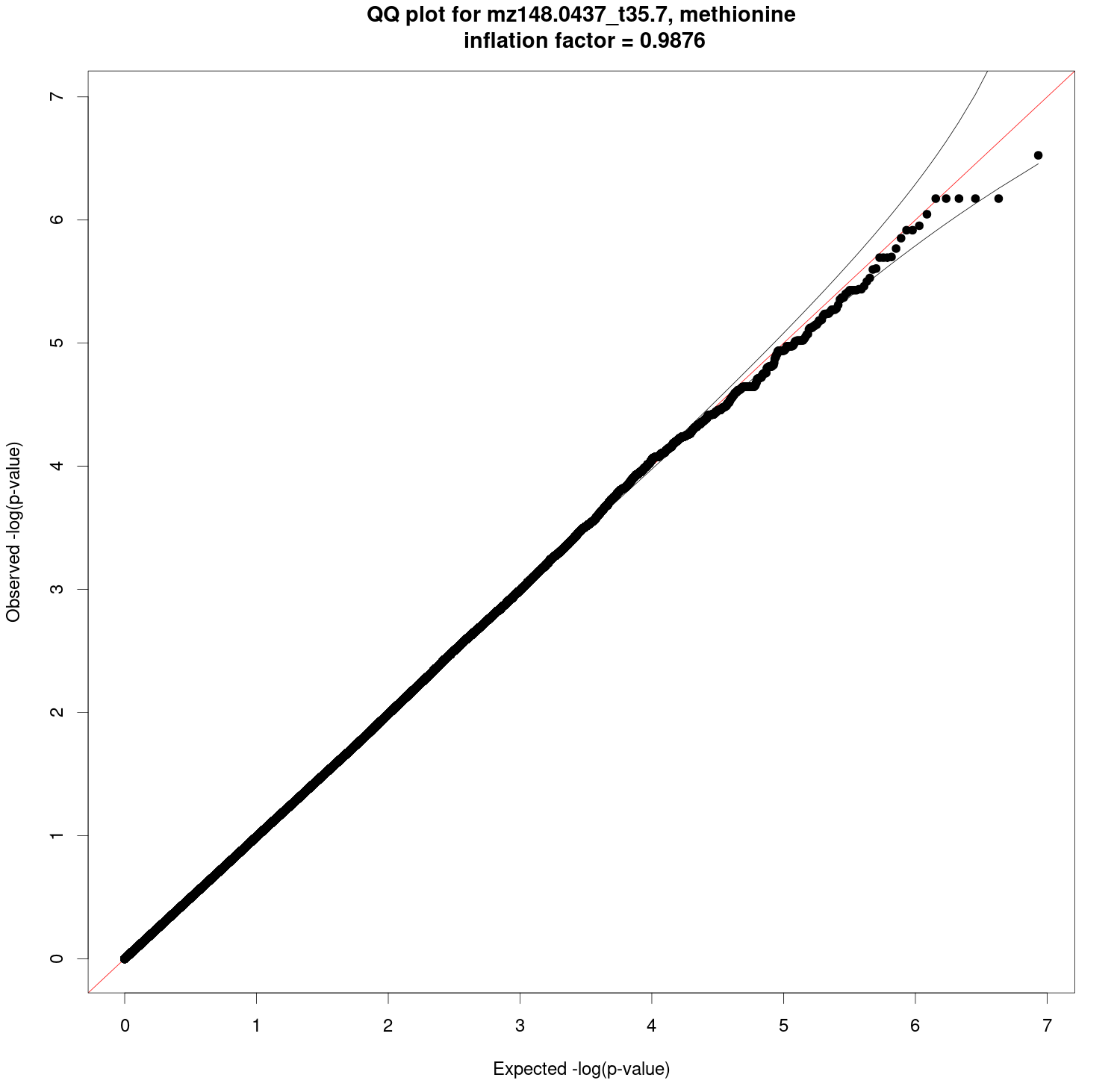
QQ plot for mz148.0437_t35.7, methionine
inflation factor = 0.9876
Observed -log(p-value)
Expected -log(p-value)
0
1
2
3
4
5
6
7

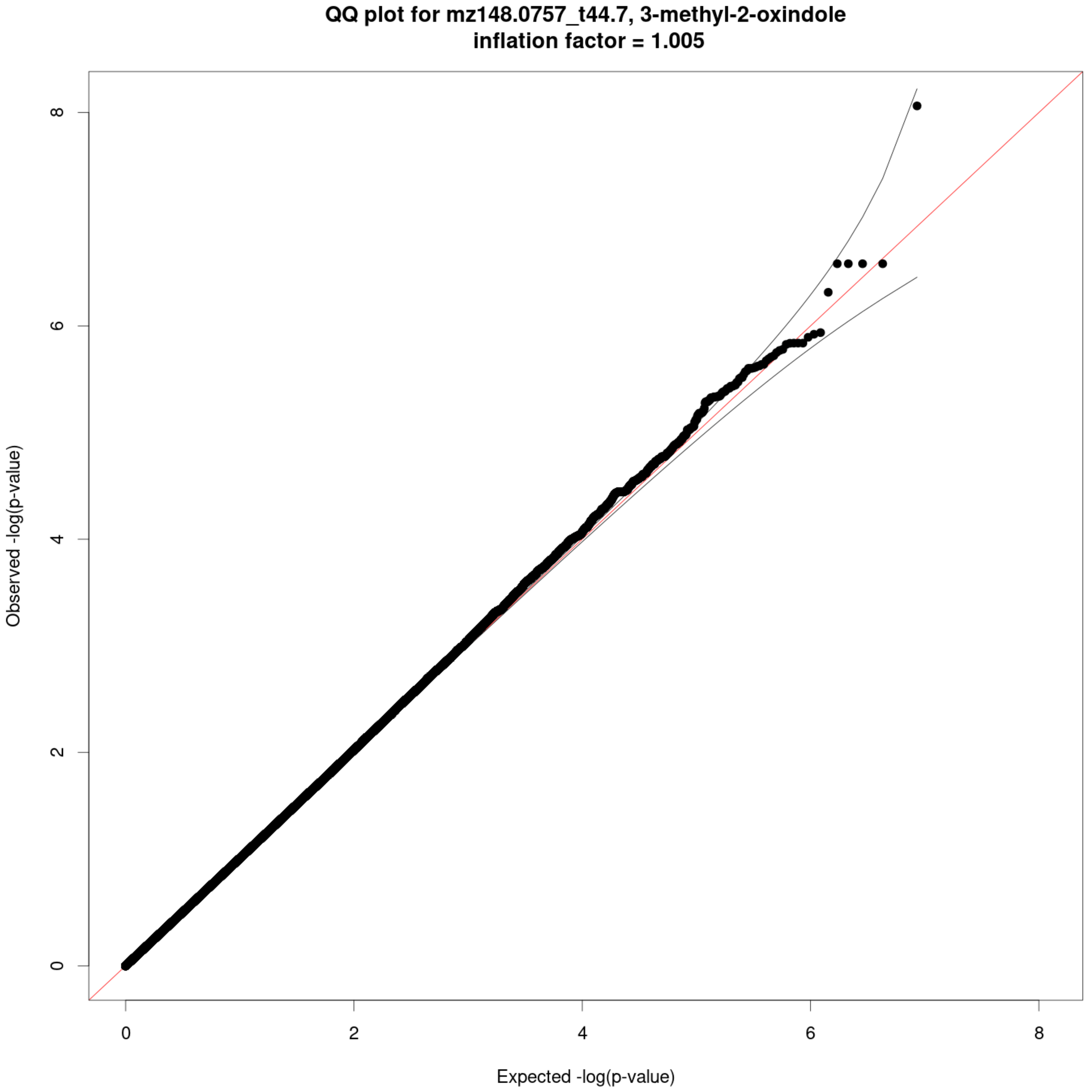
QQ plot for mz148.0757_t44.7, 3-methyl-2-oxindole
inflation factor = 1.005
Observed -log(p-value)
Expected -log(p-value)
0
2
4
6
8

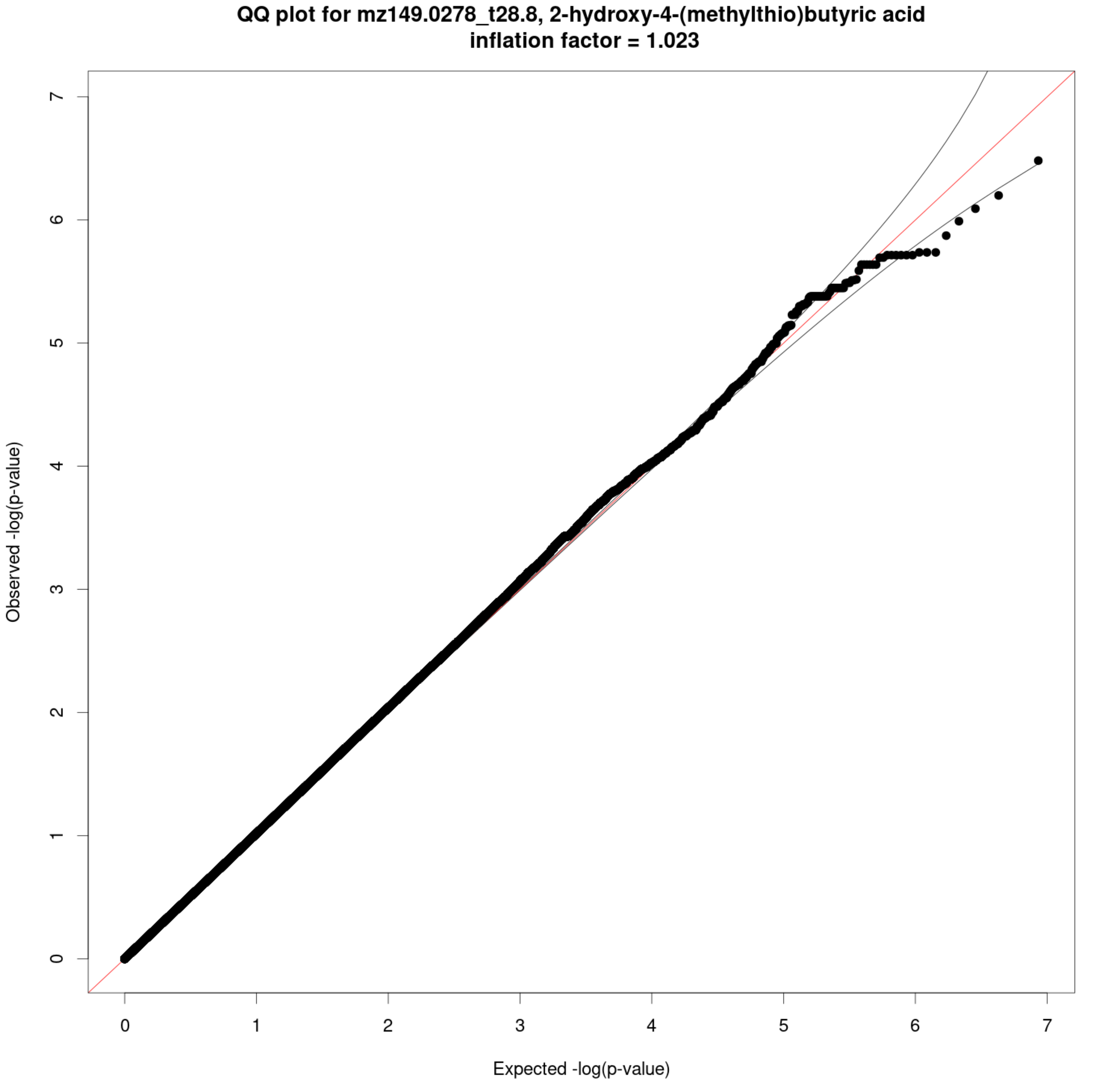
QQ plot for mz149.0278_t28.8, 2-hydroxy-4-(methylthio)butyric acid
inflation factor = 1.023
Observed -log(p-value)
Expected -log(p-value)
0
1
2
3
4
5
6
7

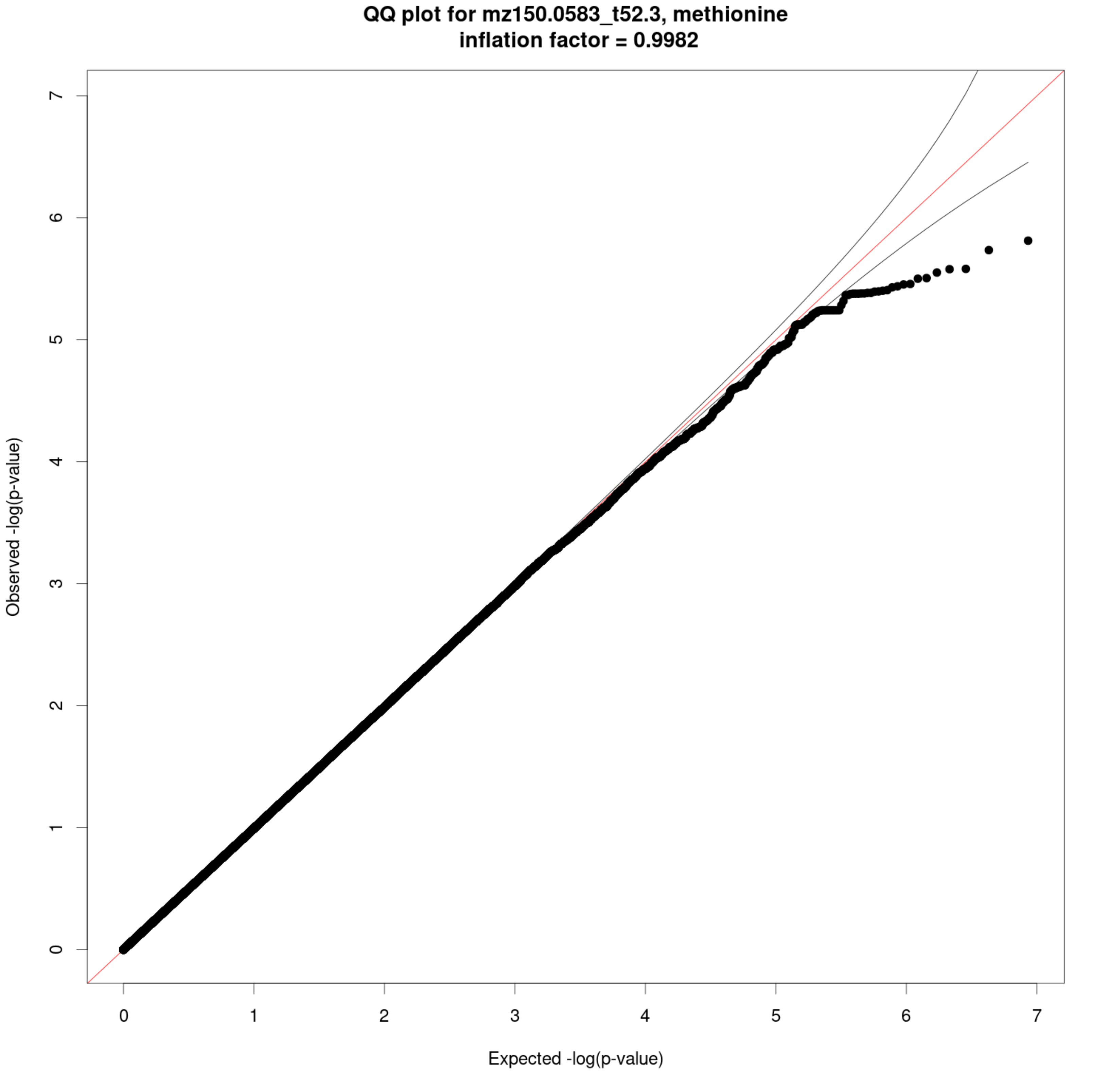

QQ plot for mz150.0583_t52.3, methionine
inflation factor = 0.9982
Observed -log(p-value)
Expected -log(p-value)
0
1
2
3
4
5
6
7

QQ plot for mz151.026_t22.8, xanthine
inflation factor = 0.9914

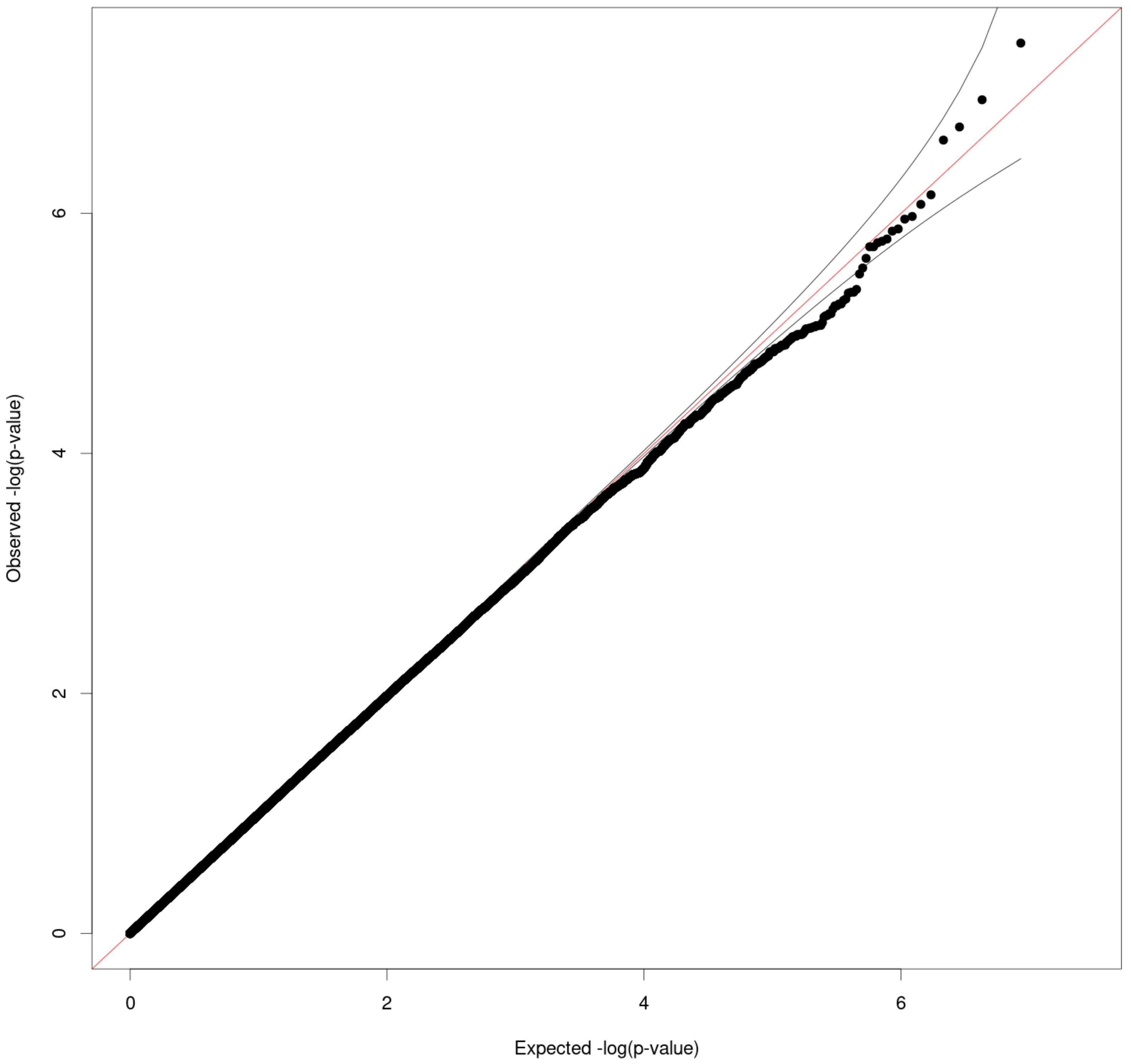

QQ plot for mz153.0407_t42, xanthine
inflation factor = 1.004

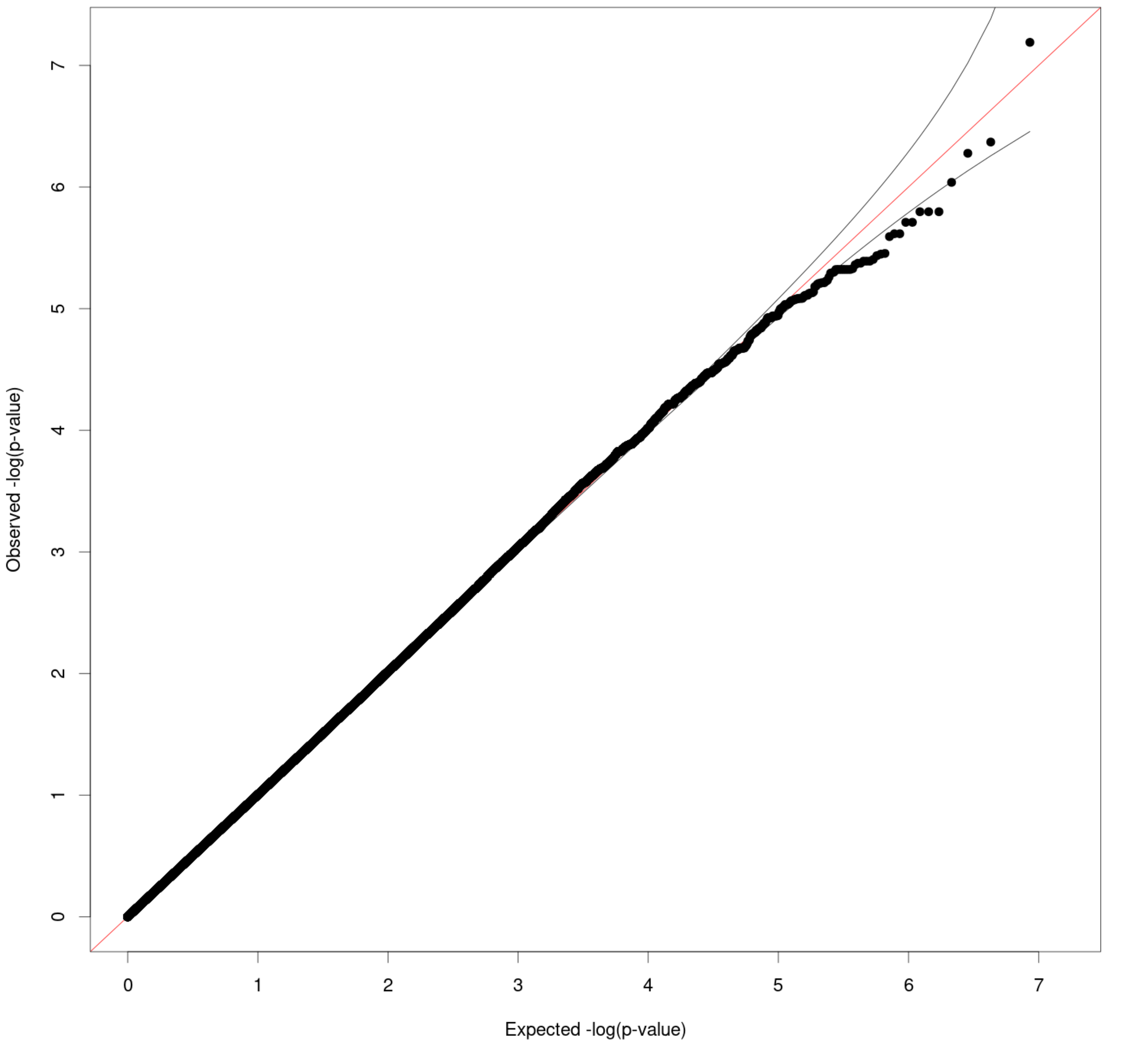

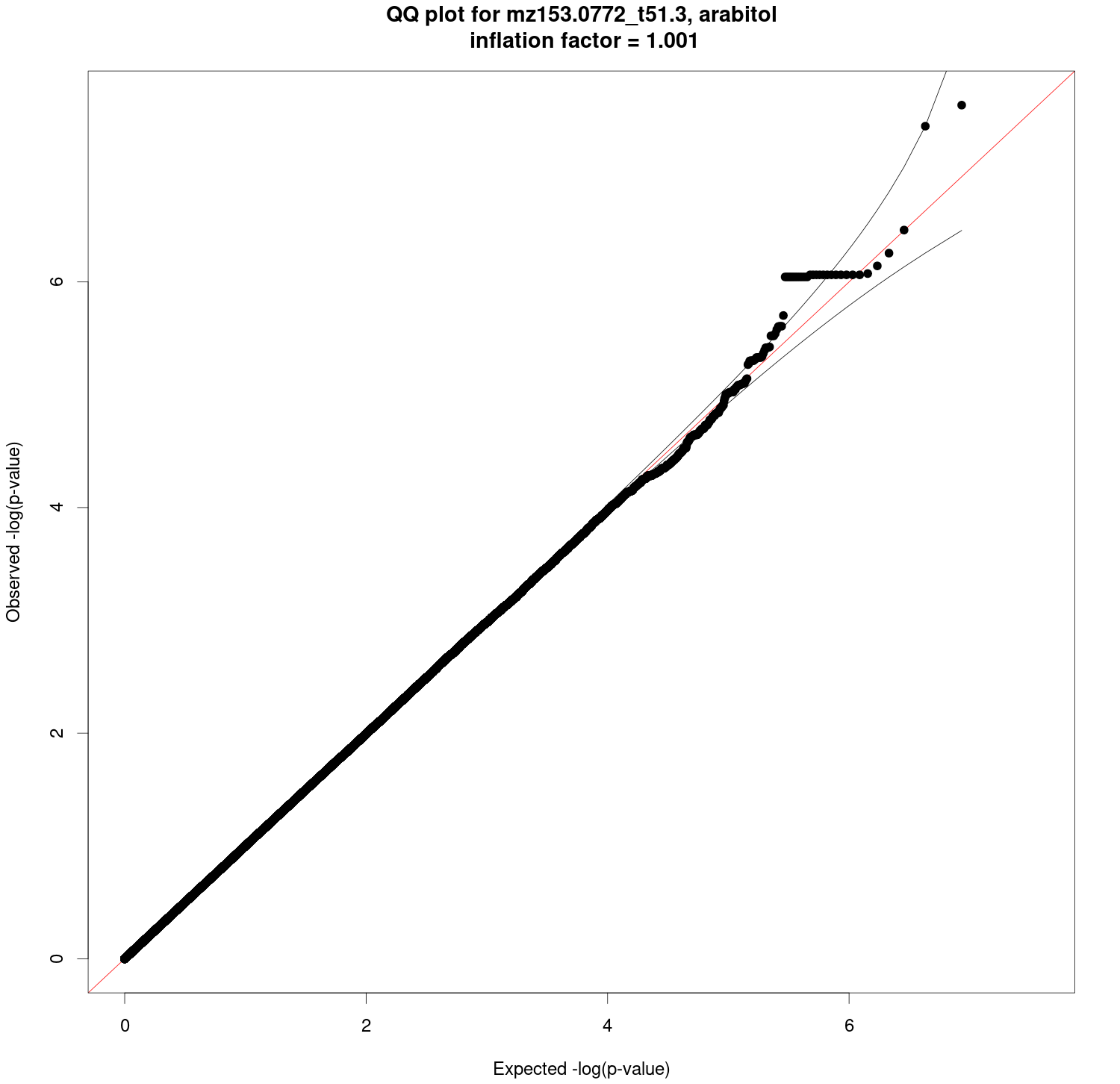

QQ plot for mz153.0772_t51.3, arabitol
inflation factor = 1.001
Observed -log(p-value)
Expected -log(p-value)
0
2
4
6
0
2
4
6

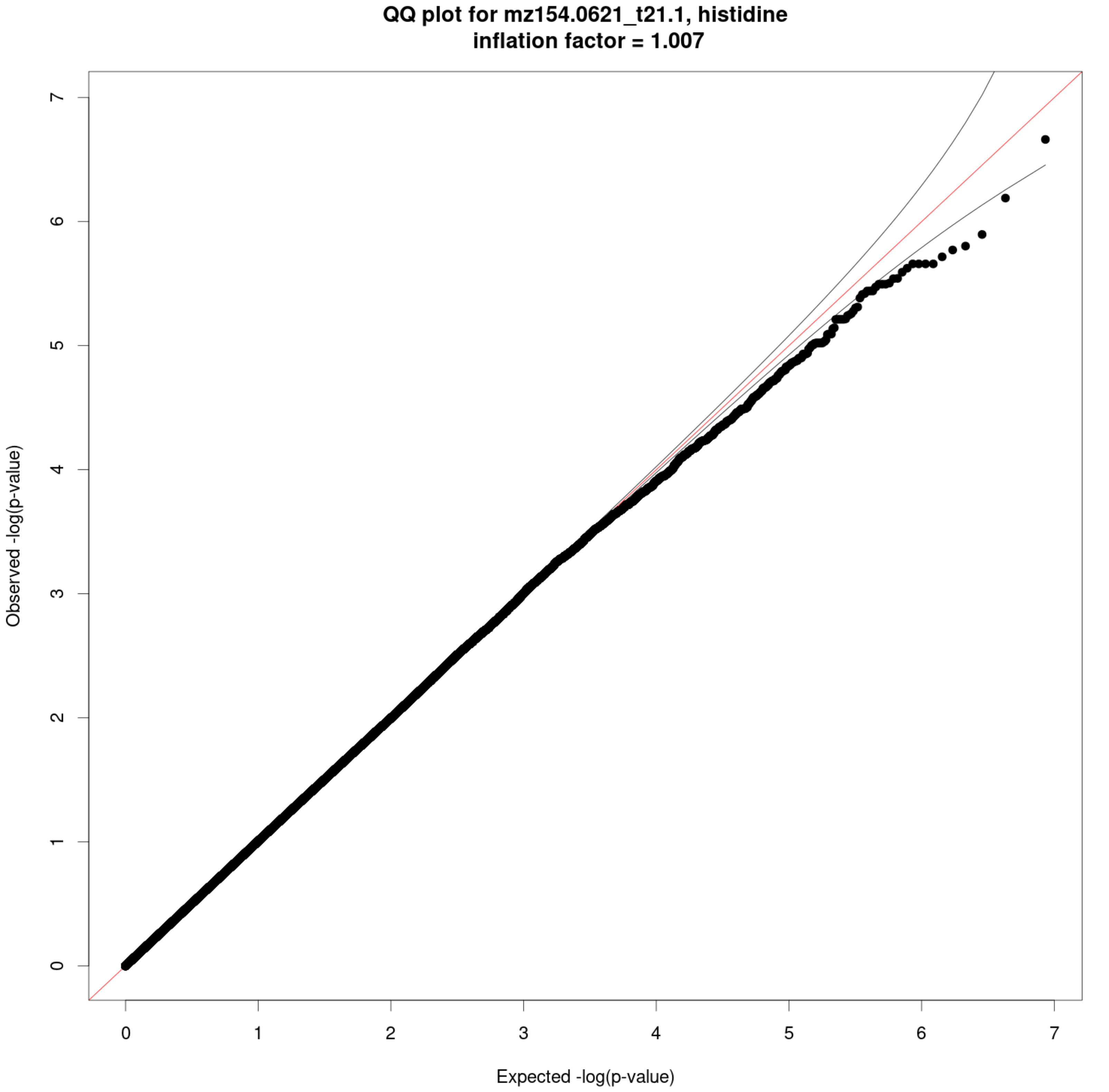

QQ plot for mz154.0621_t21.1, histidine
inflation factor = 1.007
Observed -log(p-value)
Expected -log(p-value)
0
1
2
3
4
5
6
7

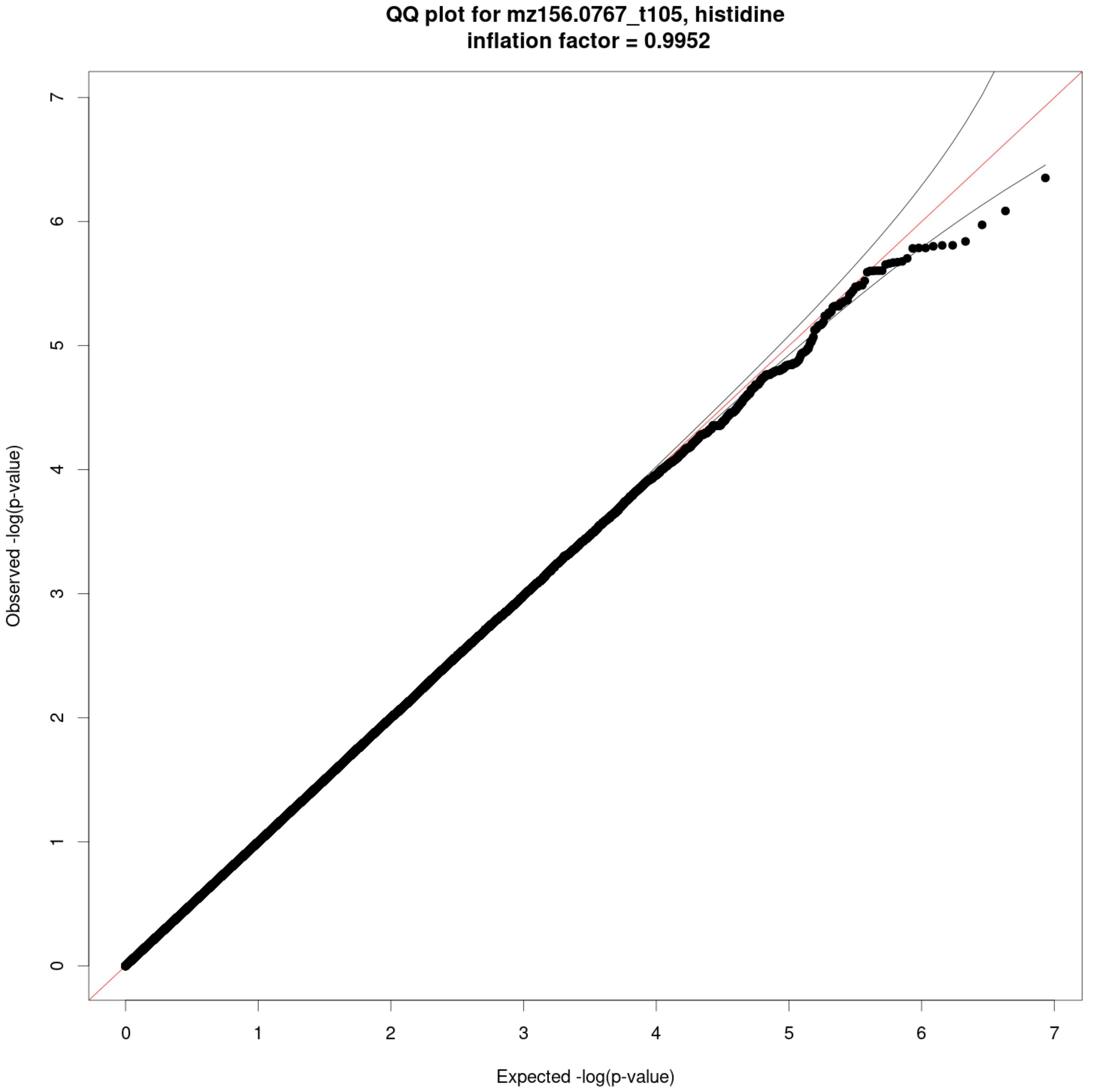

QQ plot for mz156.0767_t105, histidine
inflation factor = 0.9952
Observed -log(p-value)
Expected -log(p-value)
0
1
2
3
4
5
6
7

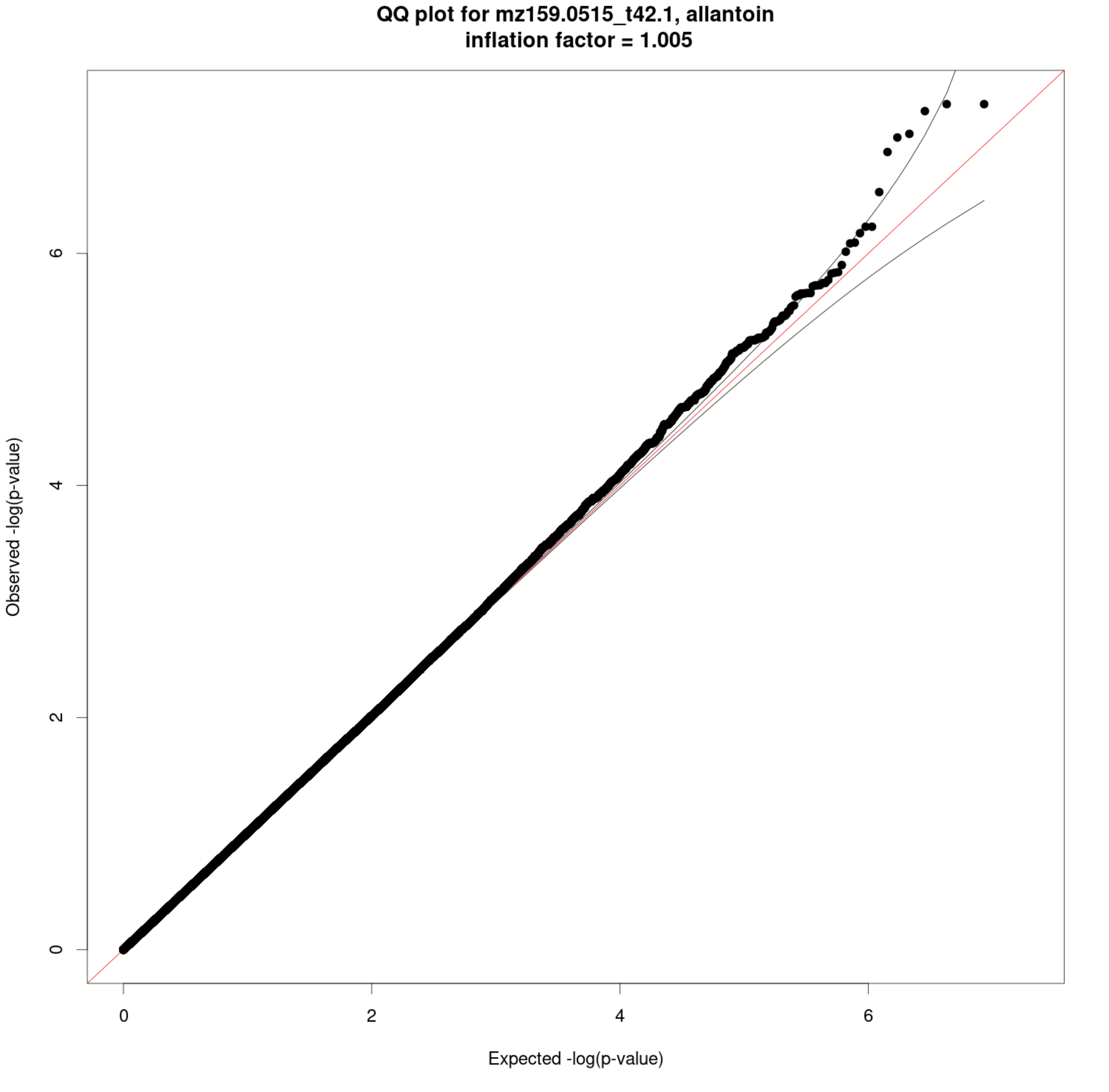
QQ plot for mz159.0515_t42.1, allantoin
inflation factor = 1.005
Observed -log(p-value)
Expected -log(p-value)
0
2
4
6
0
2
4
6

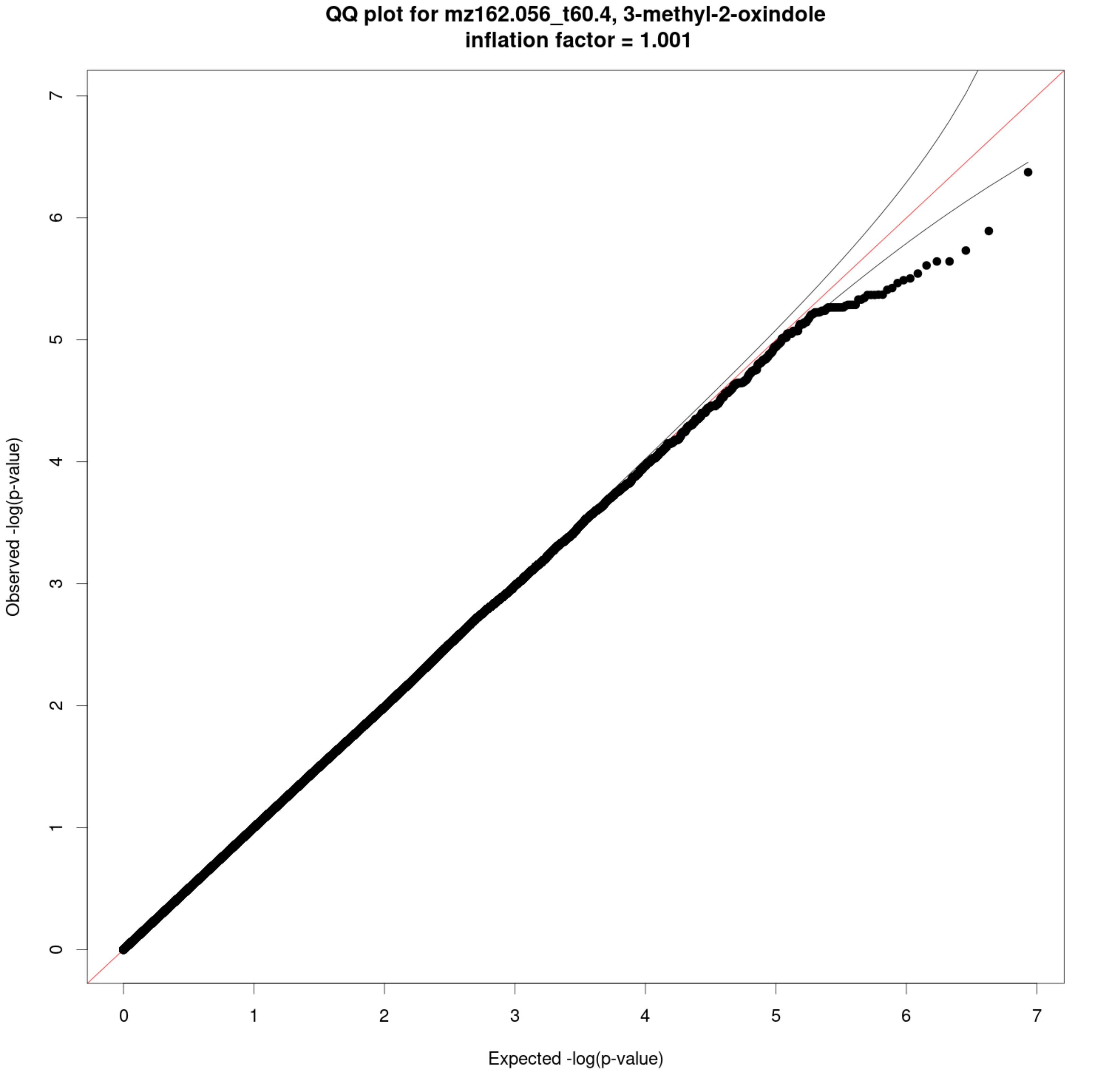
QQ plot for mz162.056_t60.4, 3-methyl-2-oxindole
inflation factor = 1.001
Observed -log(p-value)
Expected -log(p-value)
0
1
2
3
4
5
6
7

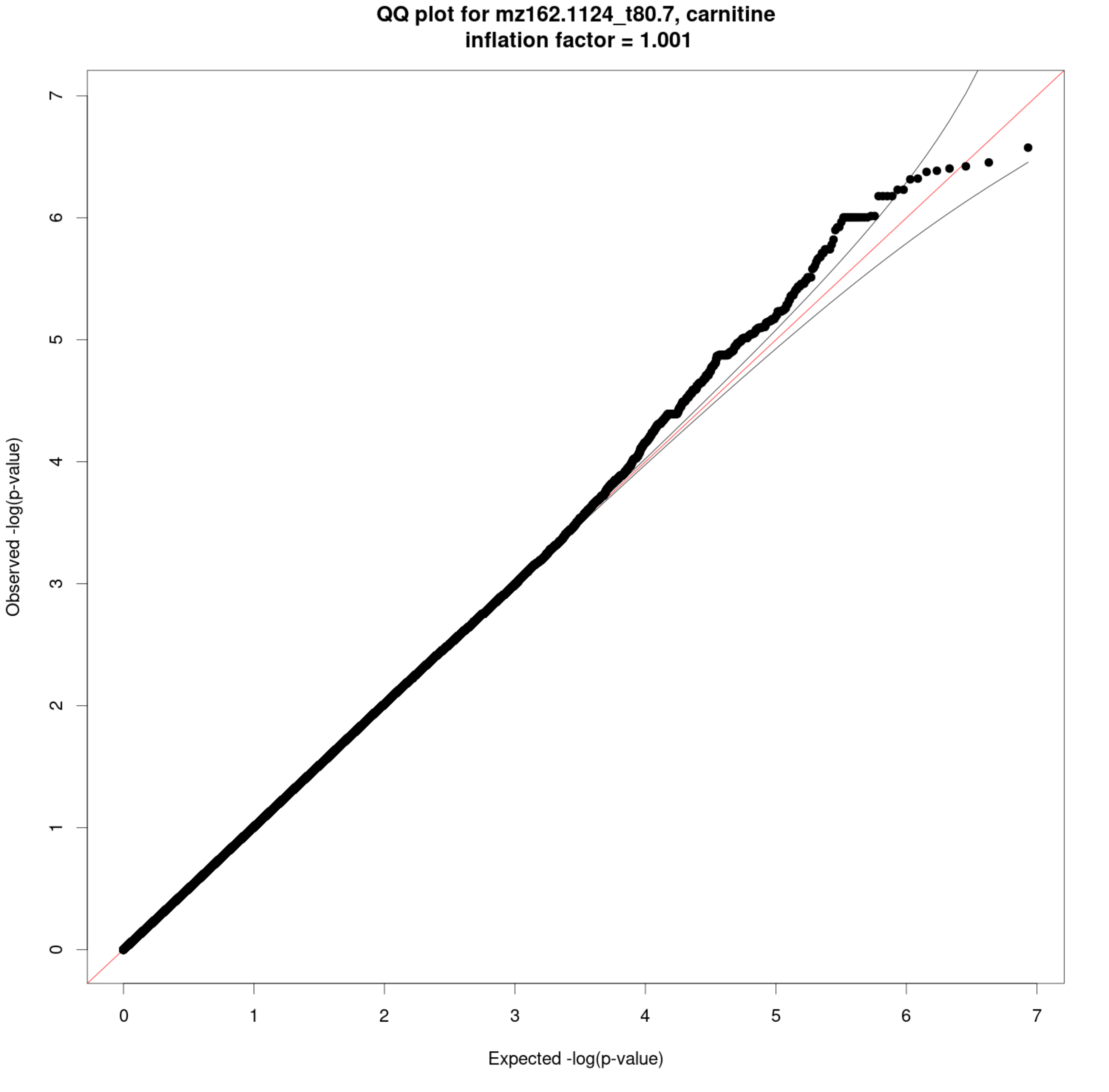
QQ plot for mz162.1124_t80.7, carnitine
inflation factor = 1.001
Observed -log(p-value)
Expected -log(p-value)
0
1
2
3
4
5
6
7

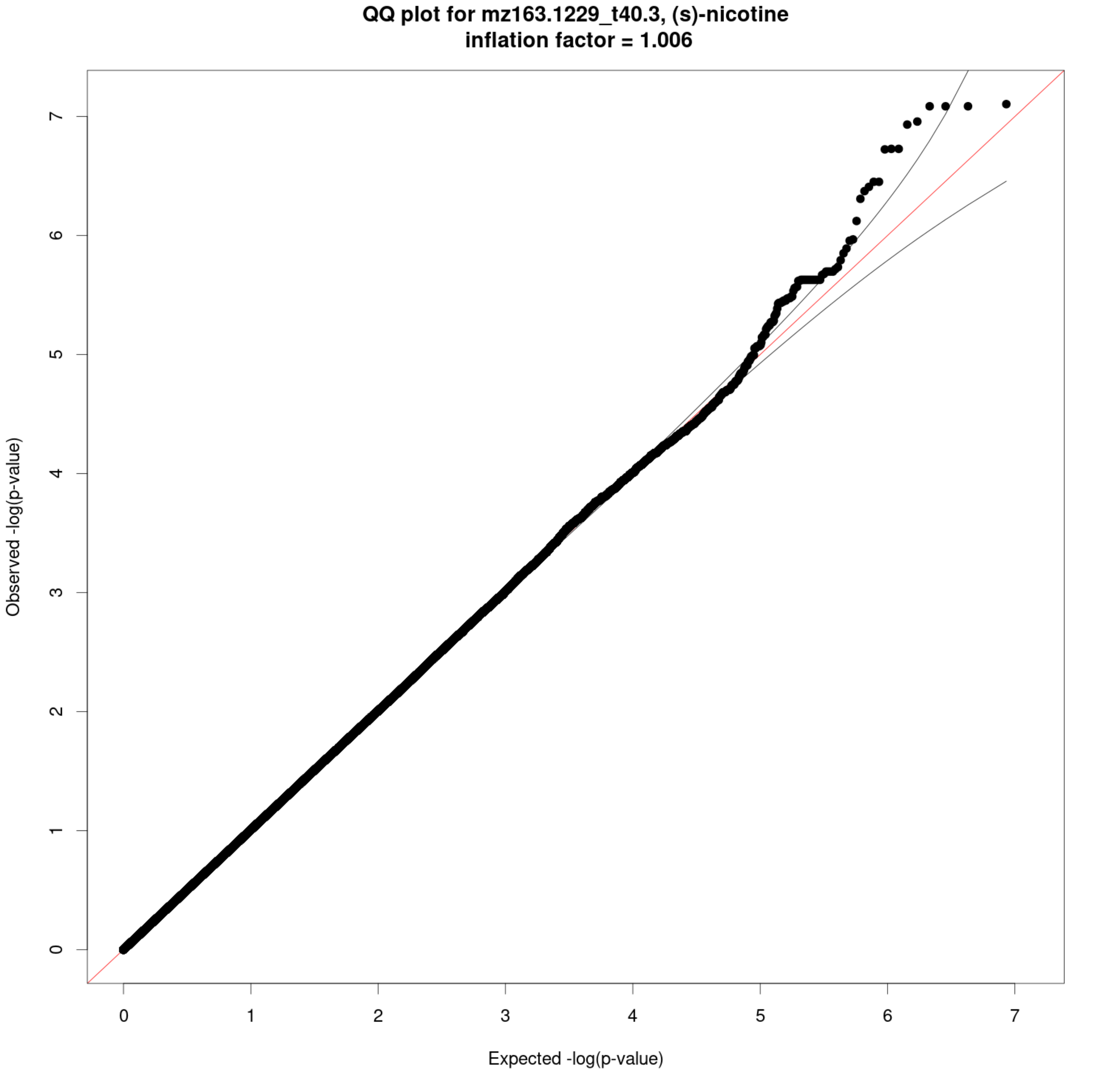
QQ plot for mz163.1229_t40.3, (s)-nicotine
inflation factor = 1.006
Observed -log(p-value)
Expected -log(p-value)
0
1
2
3
4
5
6
7

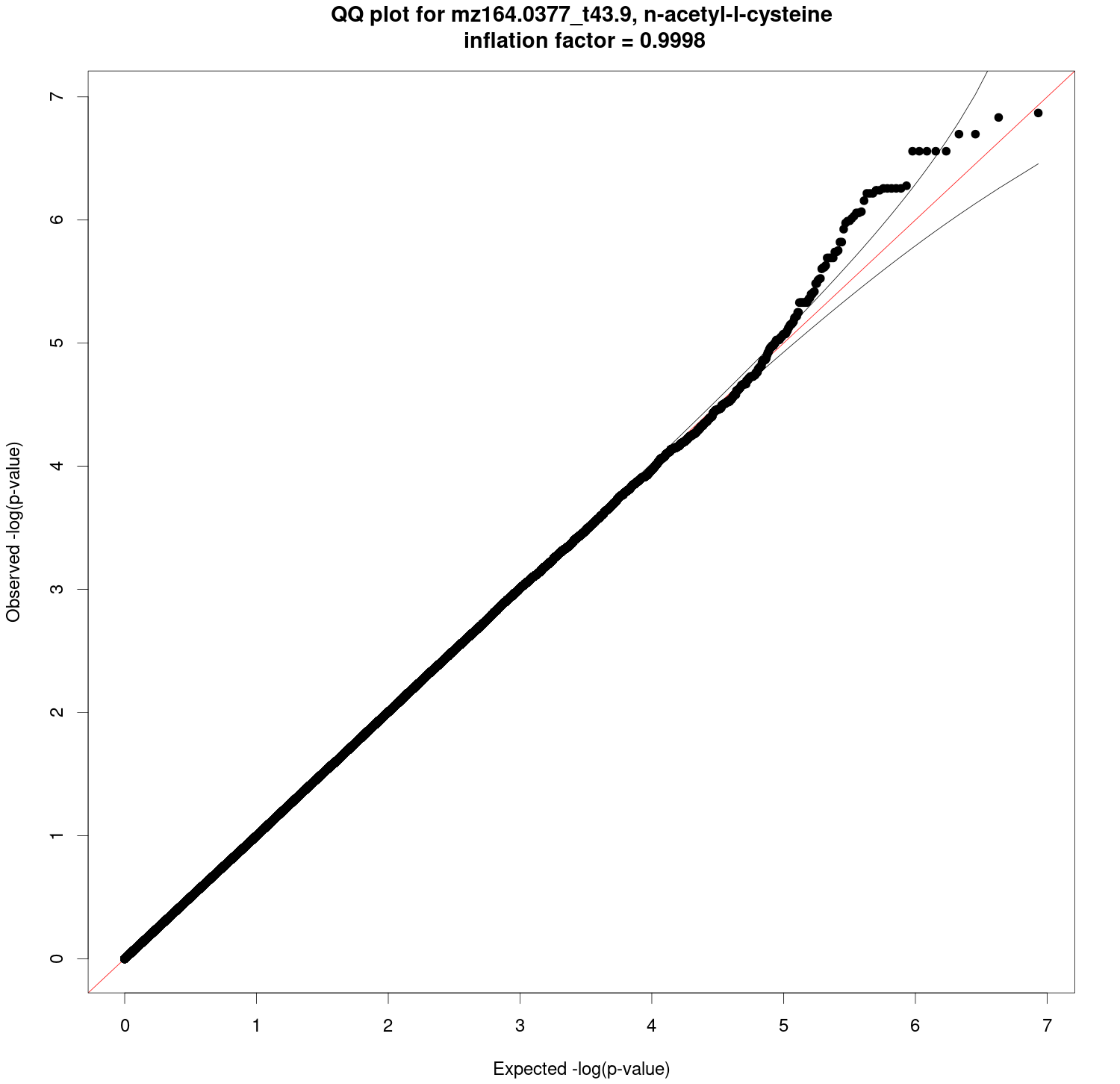
QQ plot for mz164.0377_t43.9, n-acetyl-l-cysteine
inflation factor = 0.9998
Observed -log(p-value)
Expected -log(p-value)
0
1
2
3
4
5
6
7

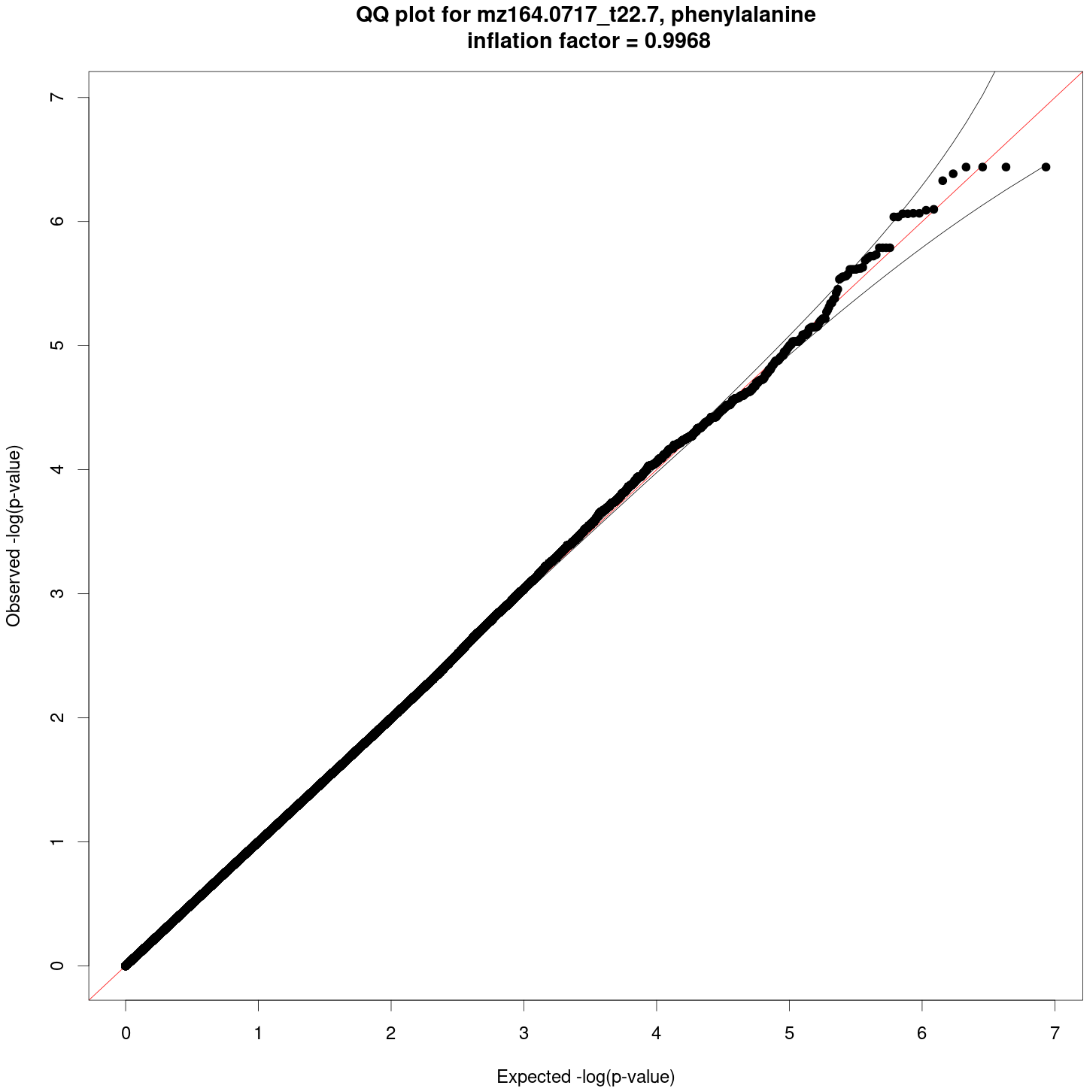
QQ plot for mz164.0717_t22.7, phenylalanine
inflation factor = 0.9968
Observed -log(p-value)
Expected -log(p-value)
0
1
2
3
4
5
6
7

QQ plot for mz165.0557_t49.5, 3-(2-hydroxyphenyl)propanoate
inflation factor = 1.003

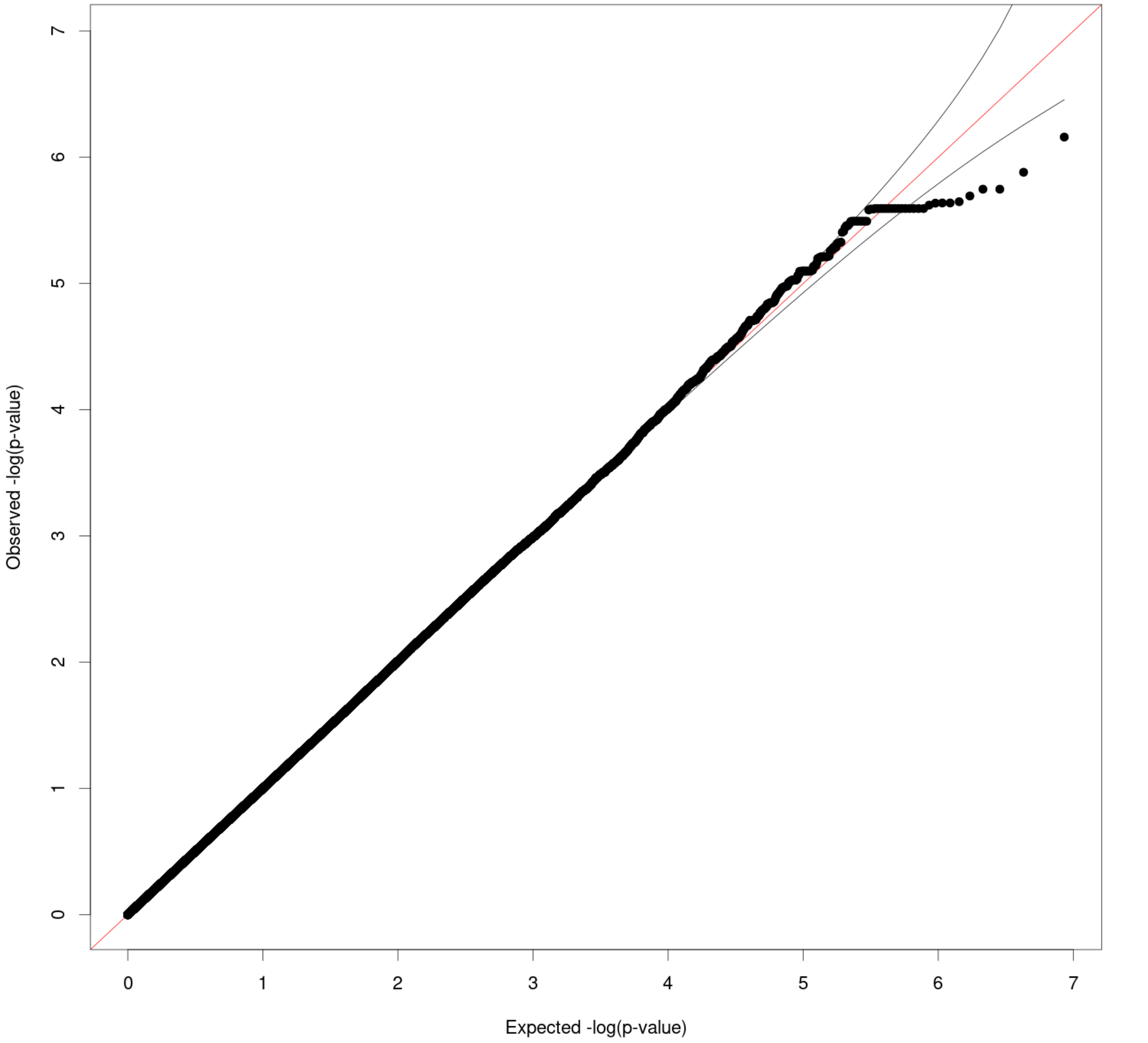

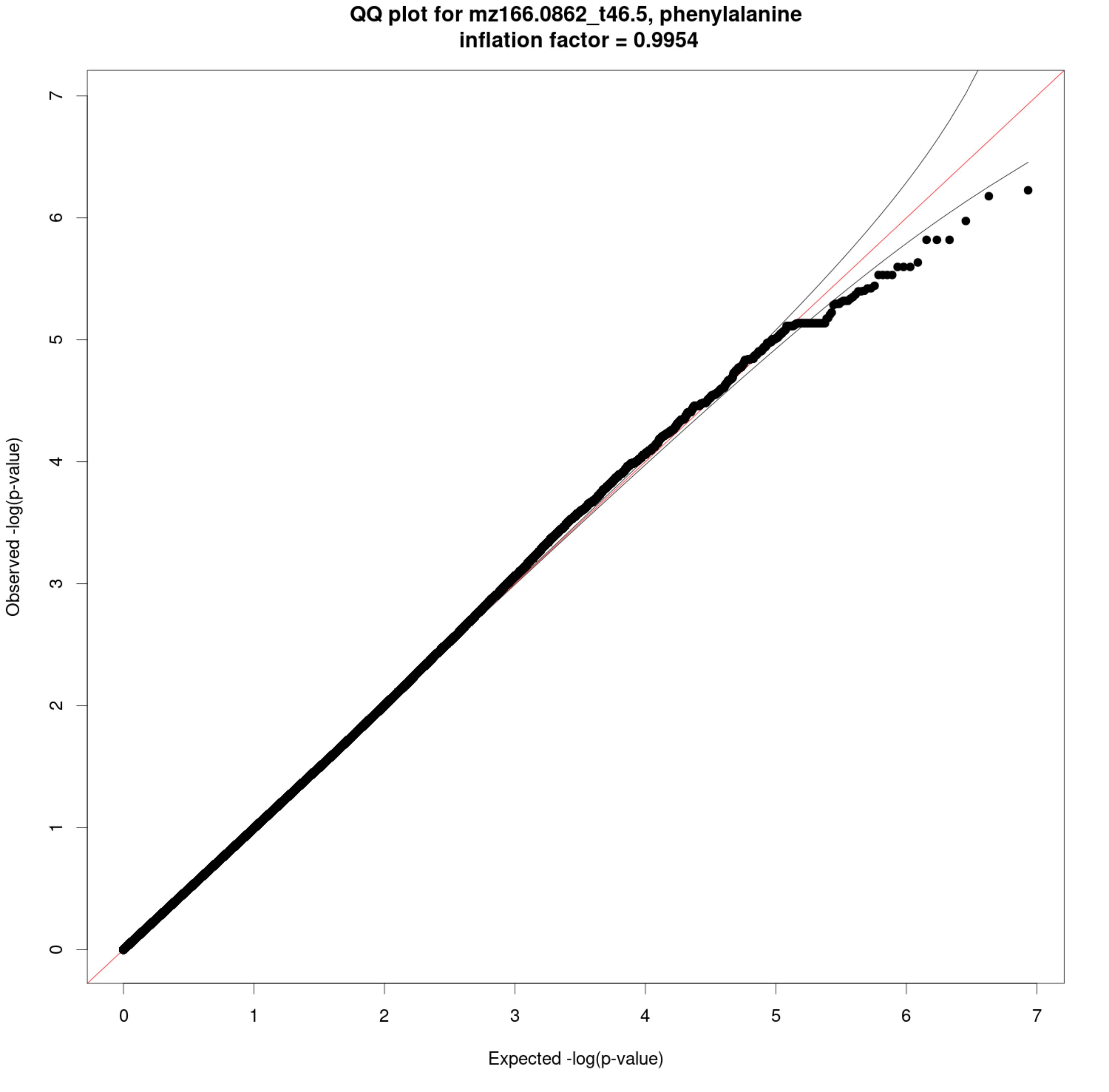
QQ plot for mz166.0862_t46.5, phenylalanine
inflation factor = 0.9954
Observed -log(p-value)
Expected -log(p-value)
0
1
2
3
4
5
6
7

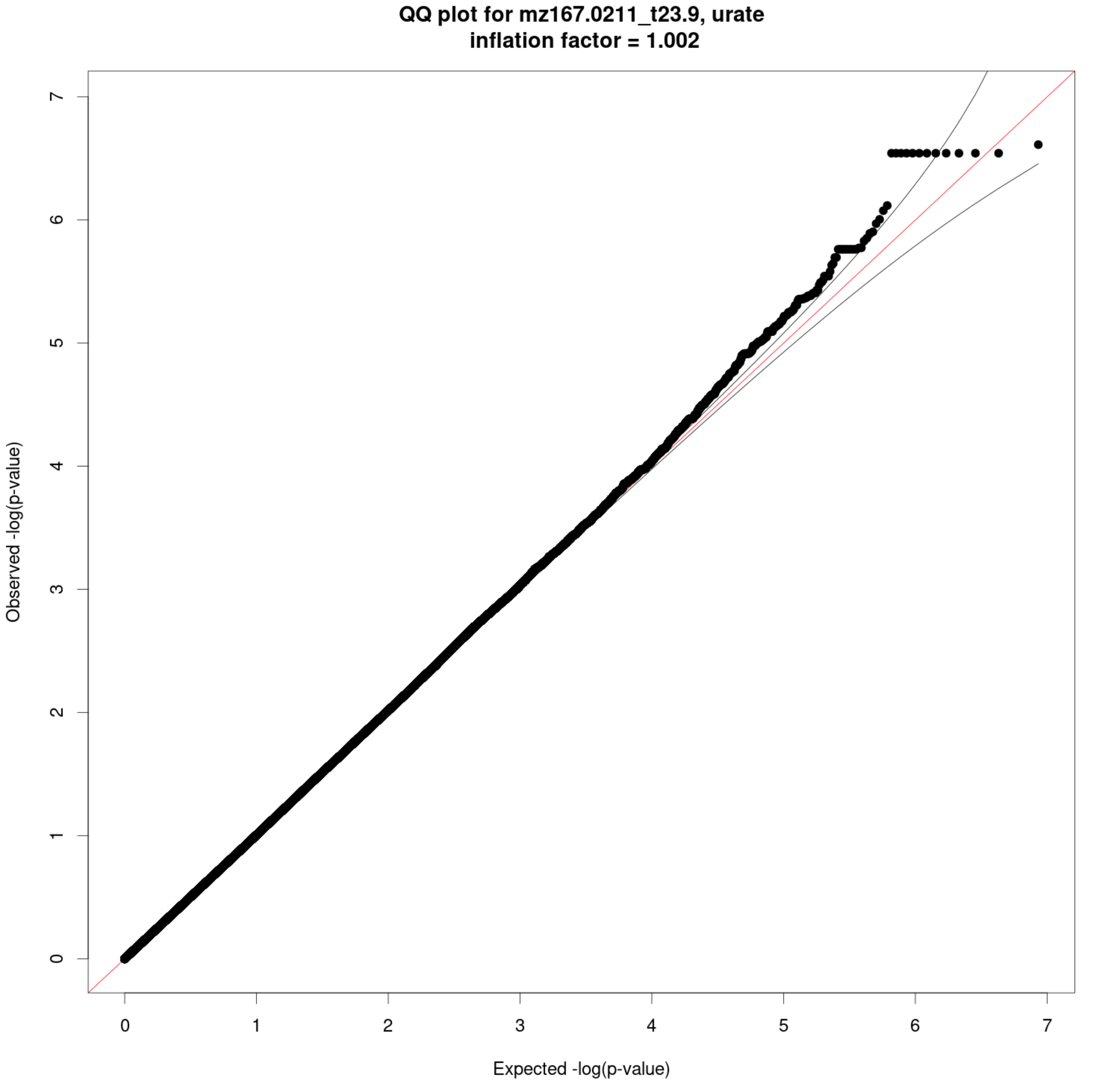
QQ plot for mz167.0211_t23.9, urate
inflation factor = 1.002
Observed -log(p-value)
Expected -log(p-value)
0
1
2
3
4
5
6
7

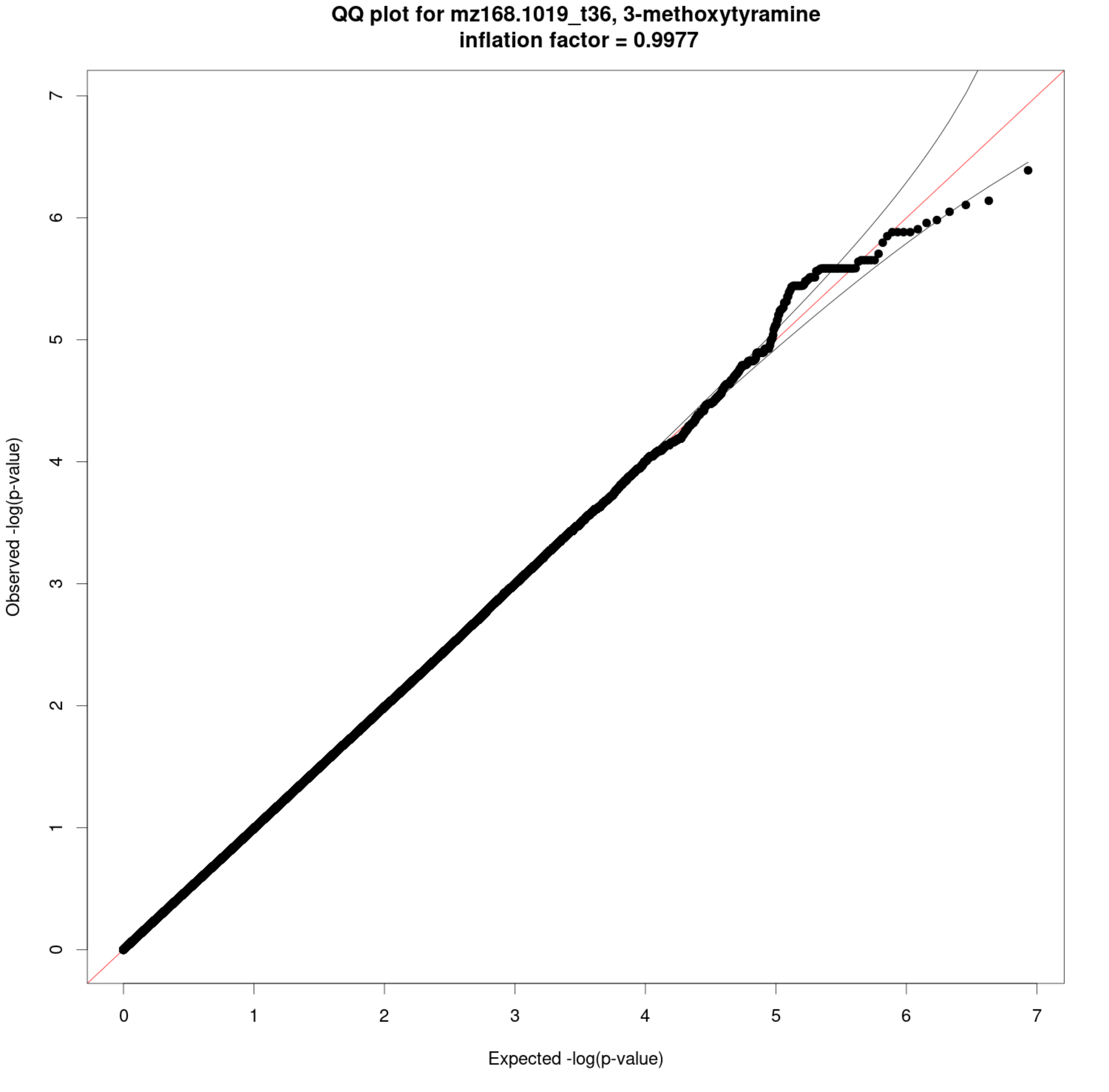
QQ plot for mz168.1019_t36, 3-methoxytyramine
inflation factor = 0.9977
Observed -log(p-value)
Expected -log(p-value)
0
1
2
3
4
5
6
7

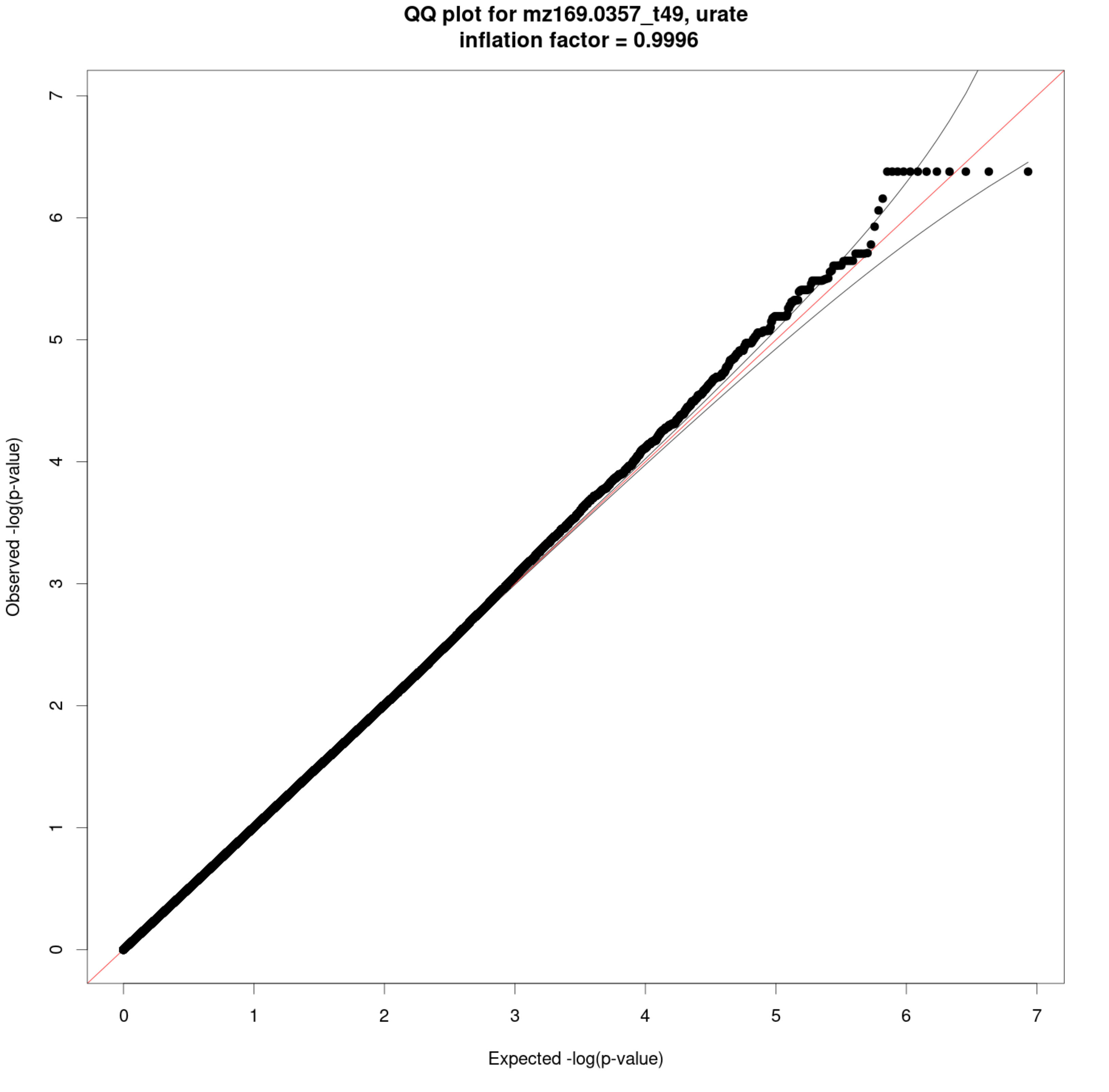
QQ plot for mz169.0357_t49, urate
inflation factor = 0.9996
Observed -log(p-value)
Expected -log(p-value)
0
1
2
3
4
5
6
7

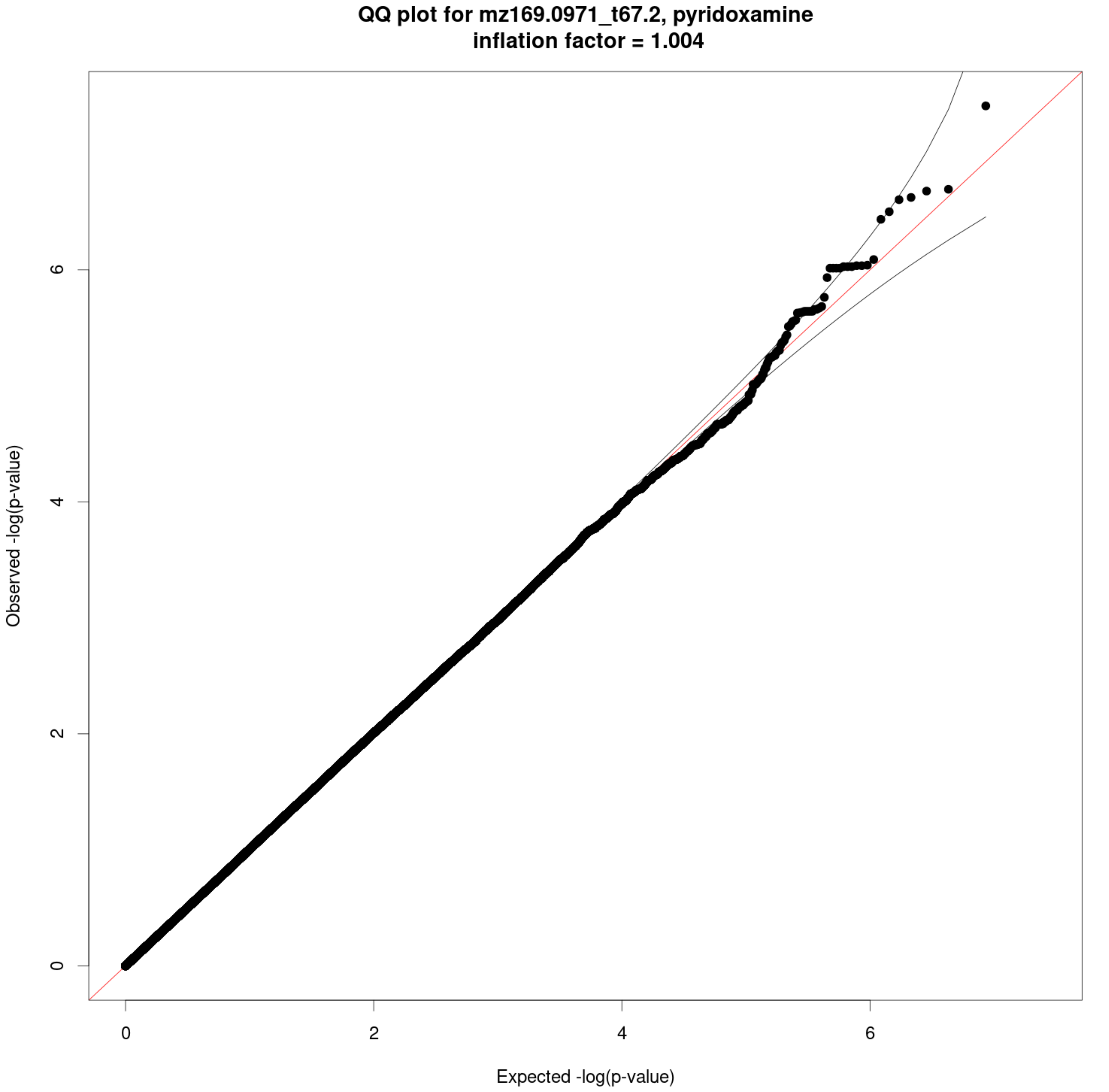
QQ plot for mz169.0971_t67.2, pyridoxamine
inflation factor = 1.004
Observed -log(p-value)
Expected -log(p-value)
0
2
4
6
0
2
4
6

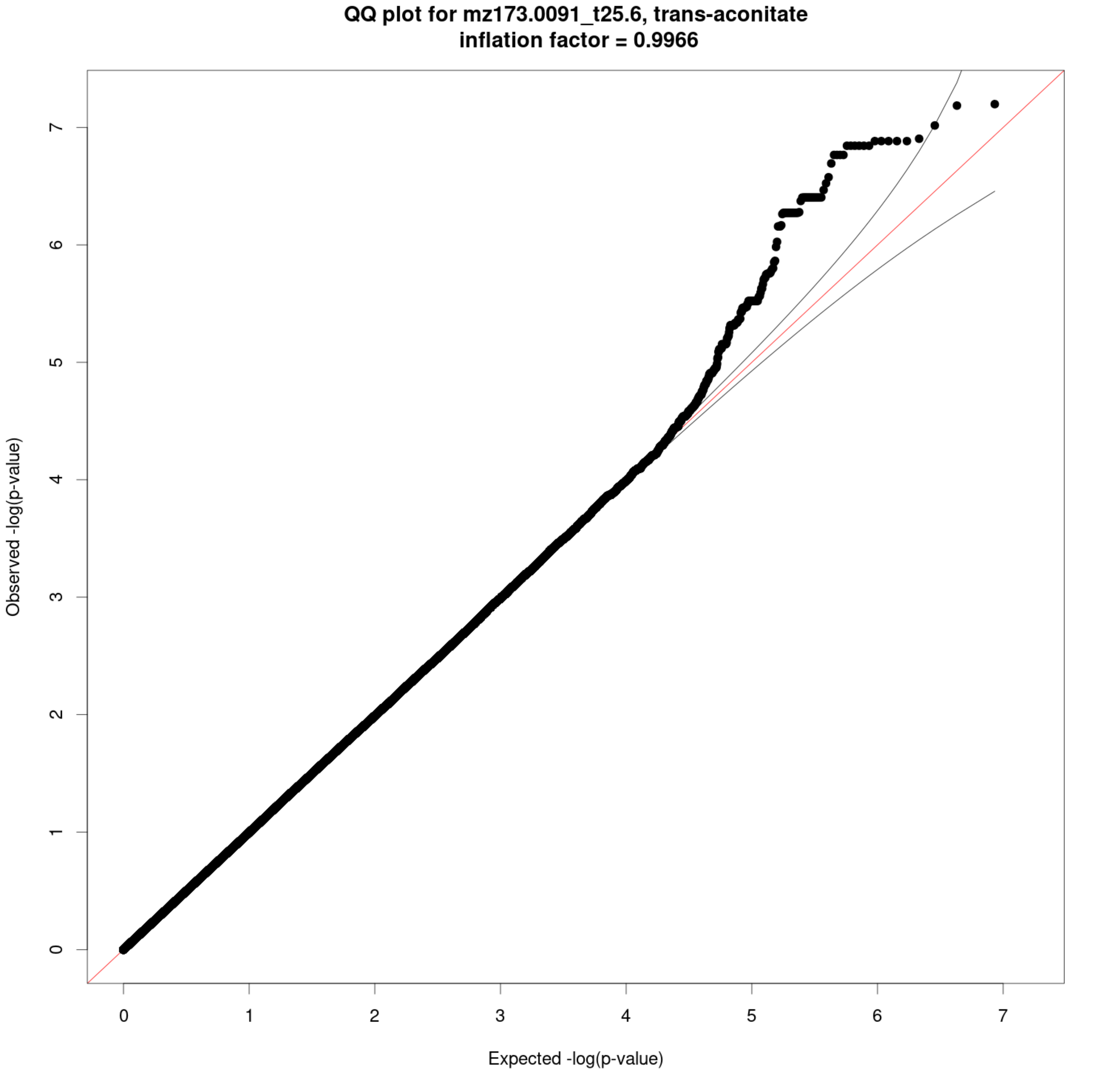

QQ plot for mz173.0091_t25.6, trans-aconitate
inflation factor = 0.9966
Observed -log(p-value)
Expected -log(p-value)
0
1
2
3
4
5
6
7

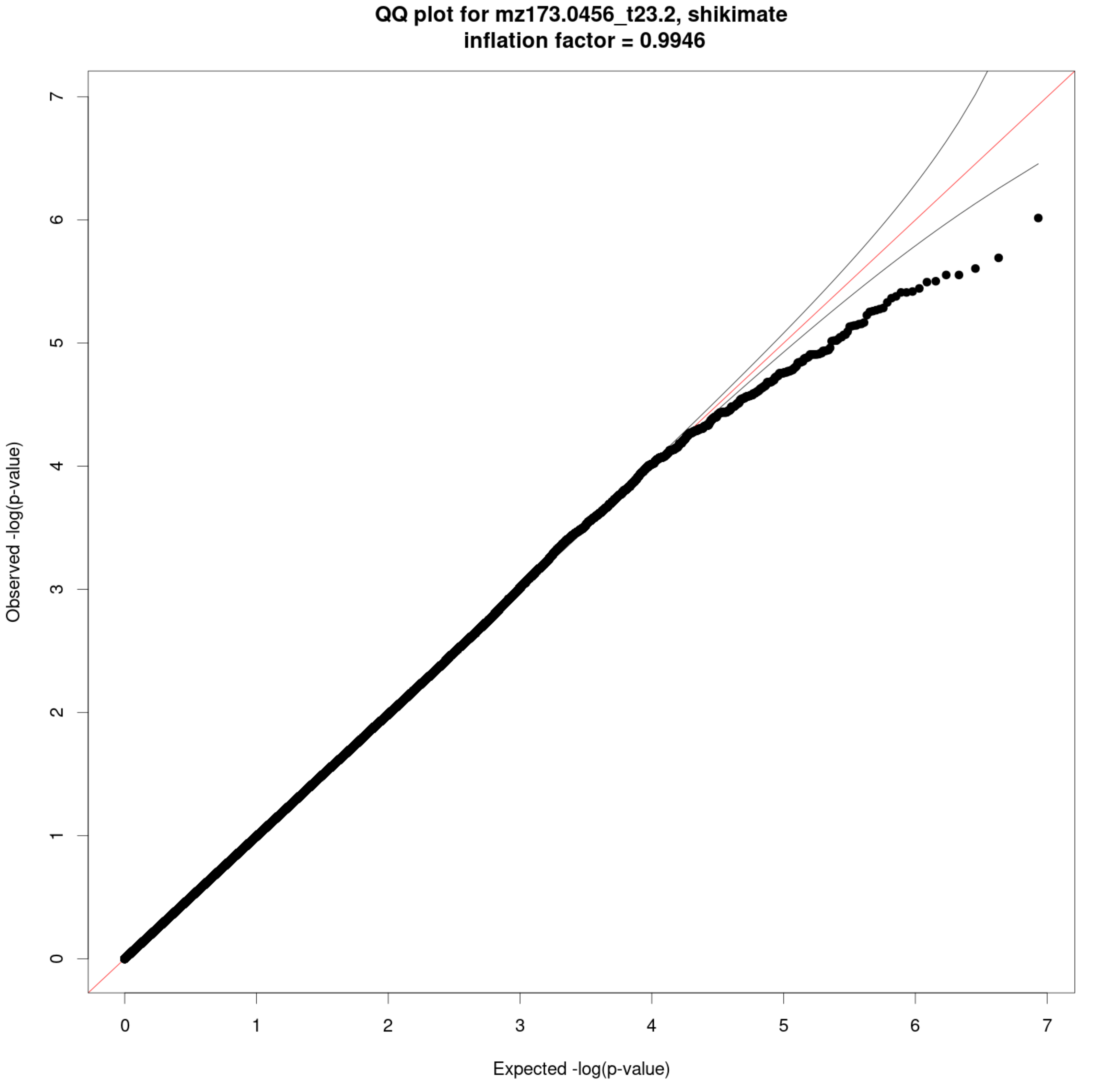
QQ plot for mz173.0456_t23.2, shikimate
inflation factor = 0.9946
Observed -log(p-value)
Expected -log(p-value)
0
1
2
3
4
5
6
7

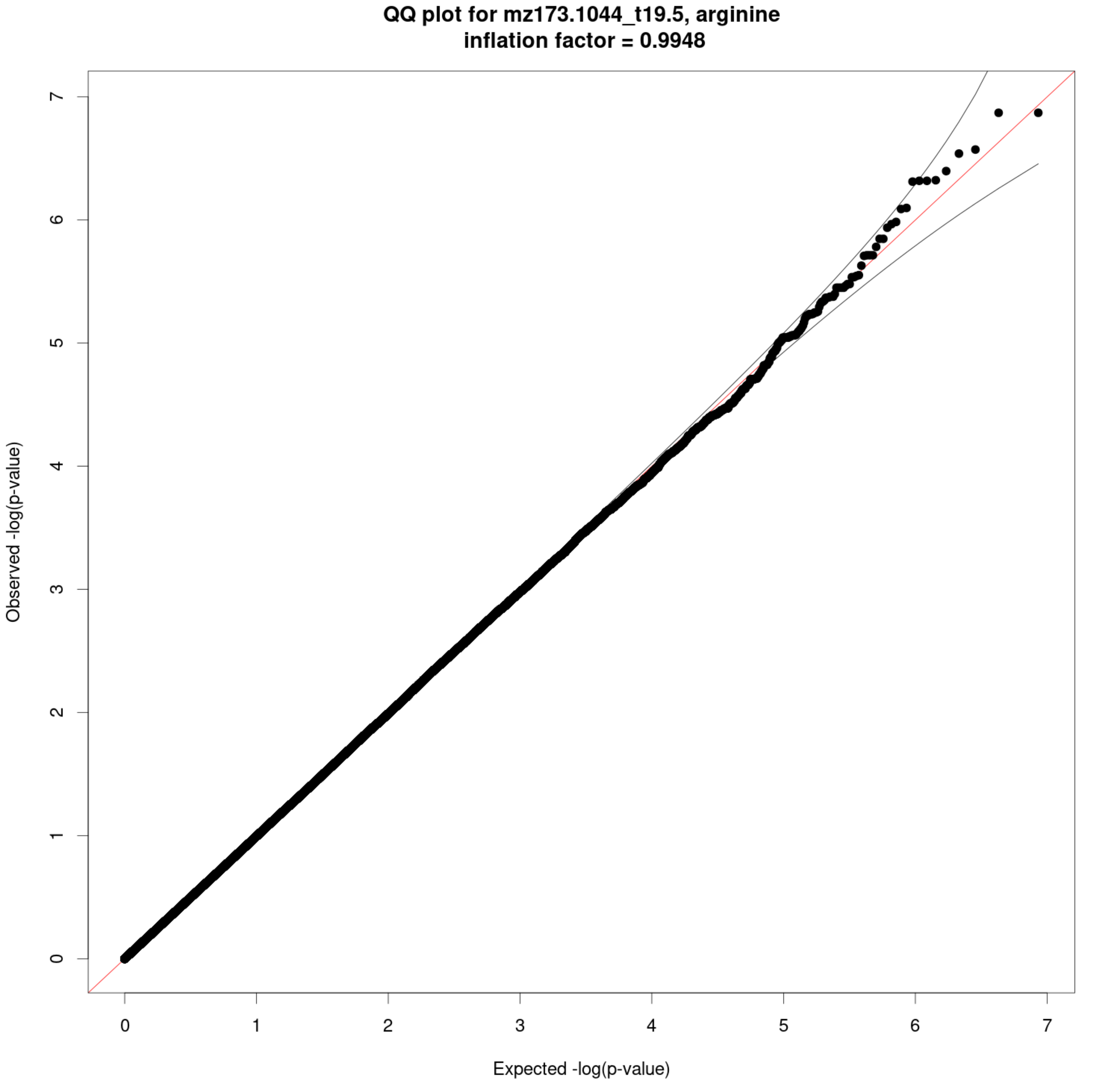
QQ plot for mz173.1044_t19.5, arginine
inflation factor = 0.9948
Observed -log(p-value)
Expected -log(p-value)
0
1
2
3
4
5
6
7

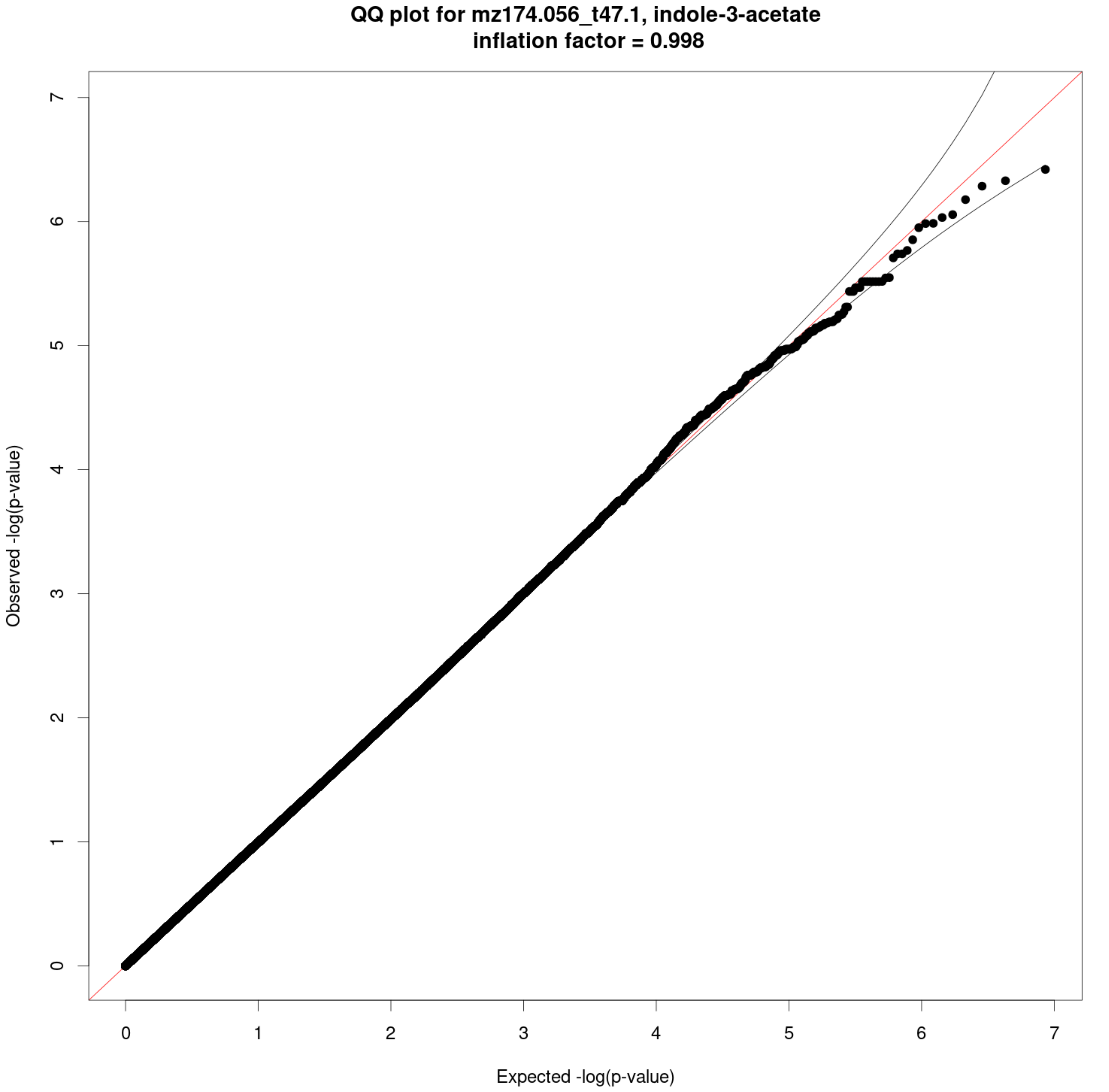
QQ plot for mz174.056_t47.1, indole-3-acetate
inflation factor = 0.998
Observed -log(p-value)
Expected -log(p-value)
0
1
2
3
4
5
6
7

QQ plot for mz174.0884_t21.1, citrulline
inflation factor = 1.011

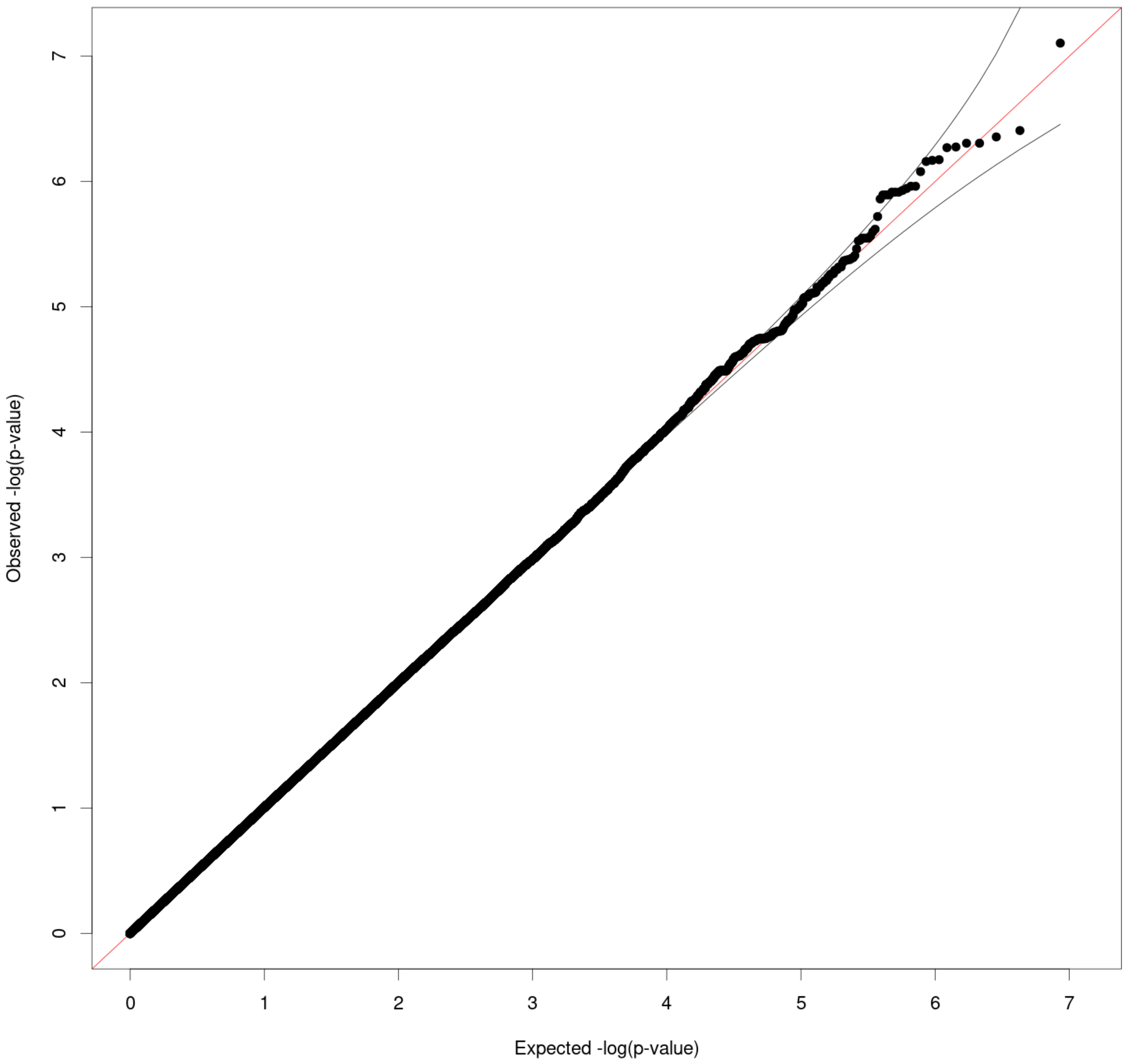

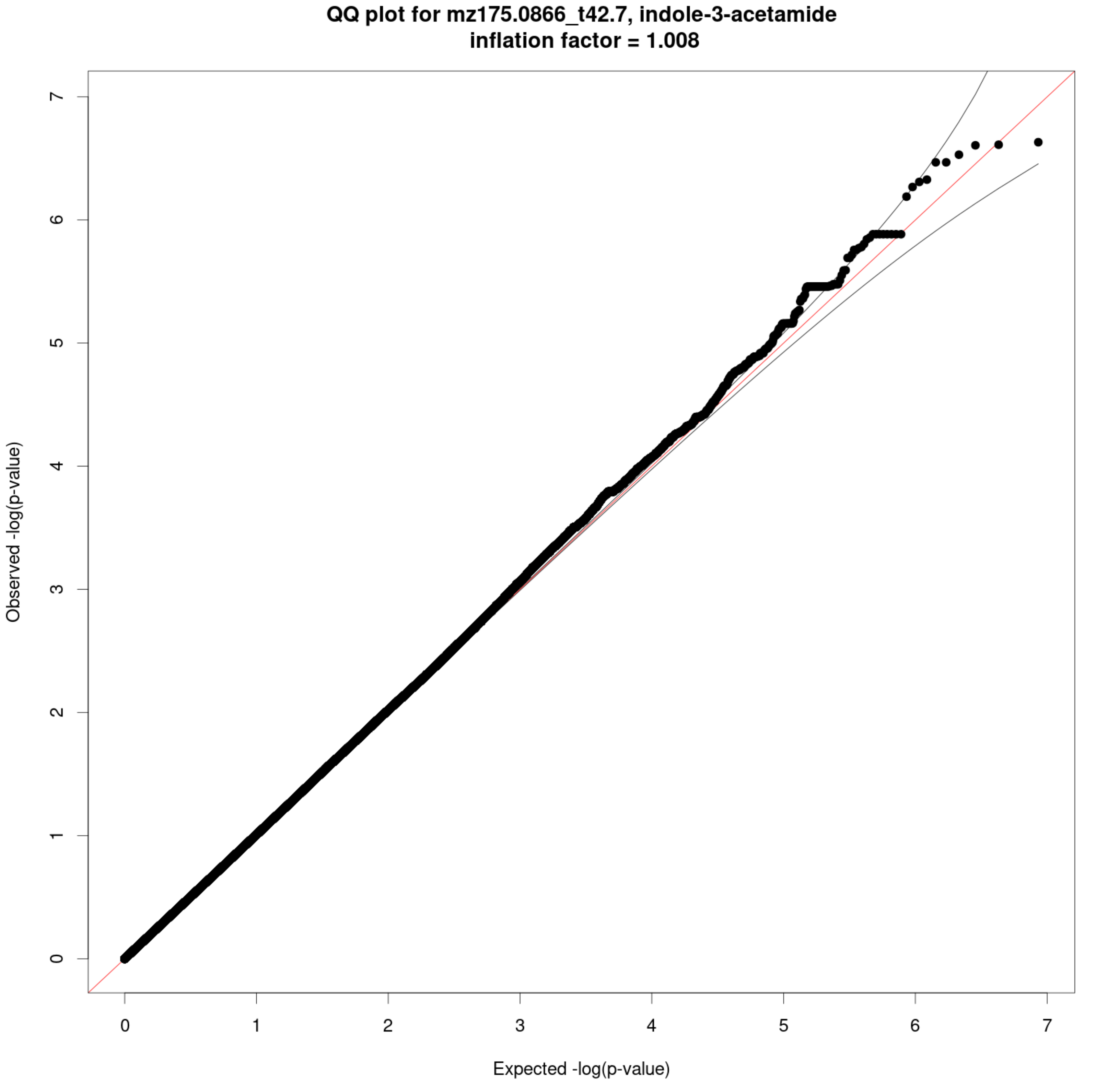
QQ plot for mz175.0866_t42.7, indole-3-acetamide
inflation factor = 1.008
Observed -log(p-value)
Expected -log(p-value)
0
1
2
3
4
5
6
7

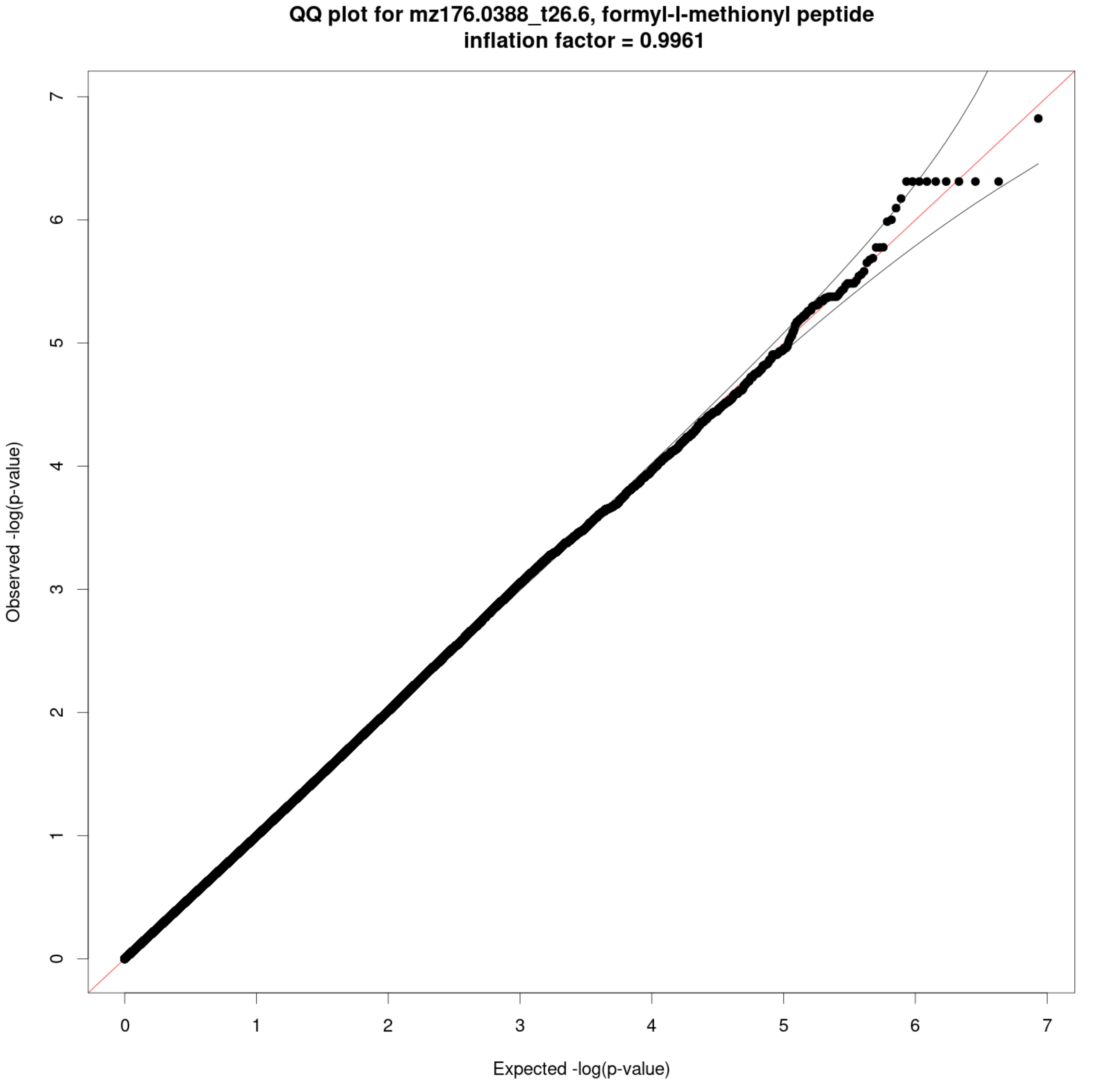
QQ plot for mz176.0388_t26.6, formyl-l-methionyl peptide
inflation factor = 0.9961
Observed -log(p-value)
Expected -log(p-value)
0
1
2
3
4
5
6
7

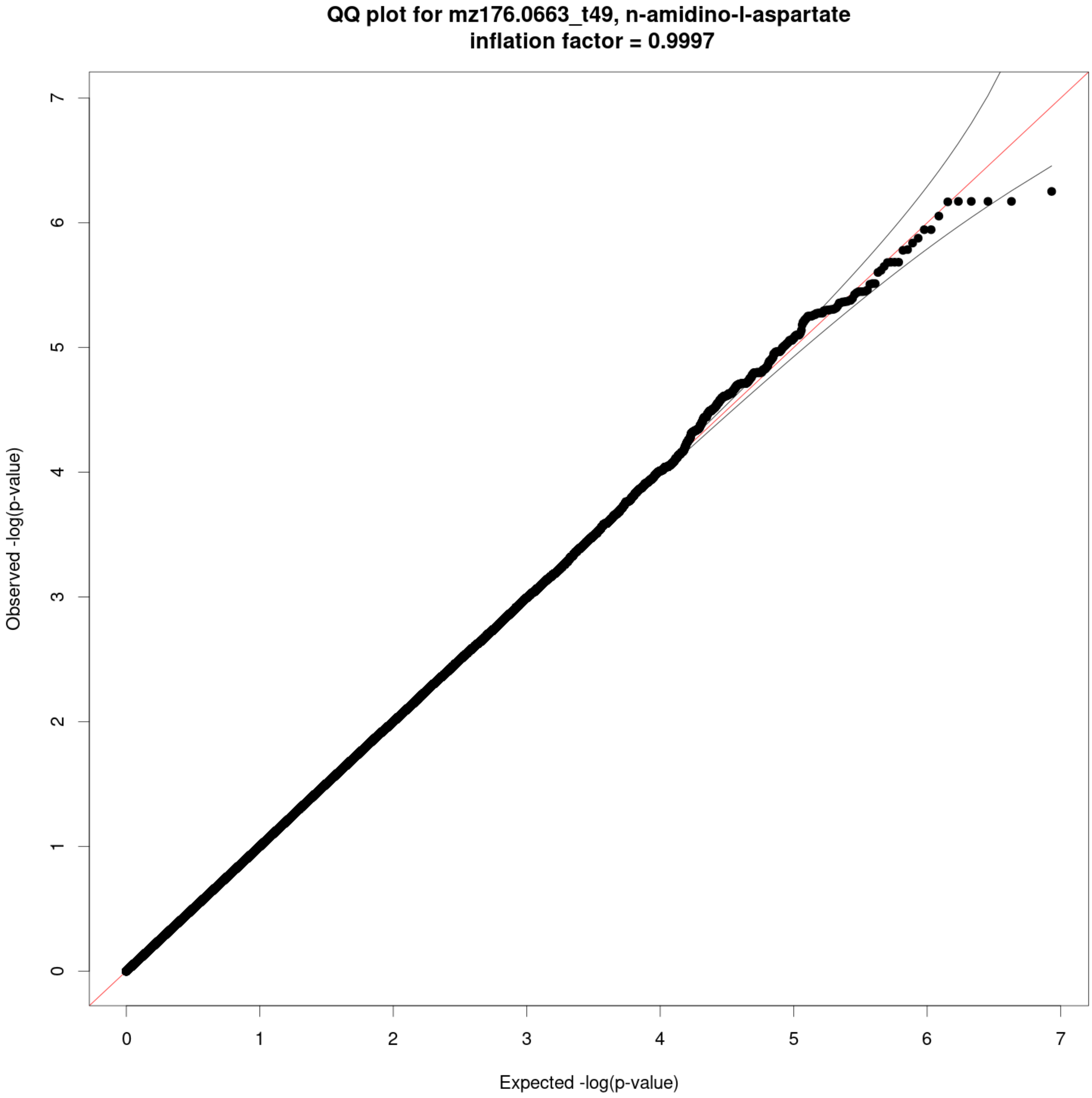
QQ plot for mz176.0663_t49, n-amidino-l-aspartate
inflation factor = 0.9997
Observed -log(p-value)
Expected -log(p-value)
0
1
2
3
4
5
6
7

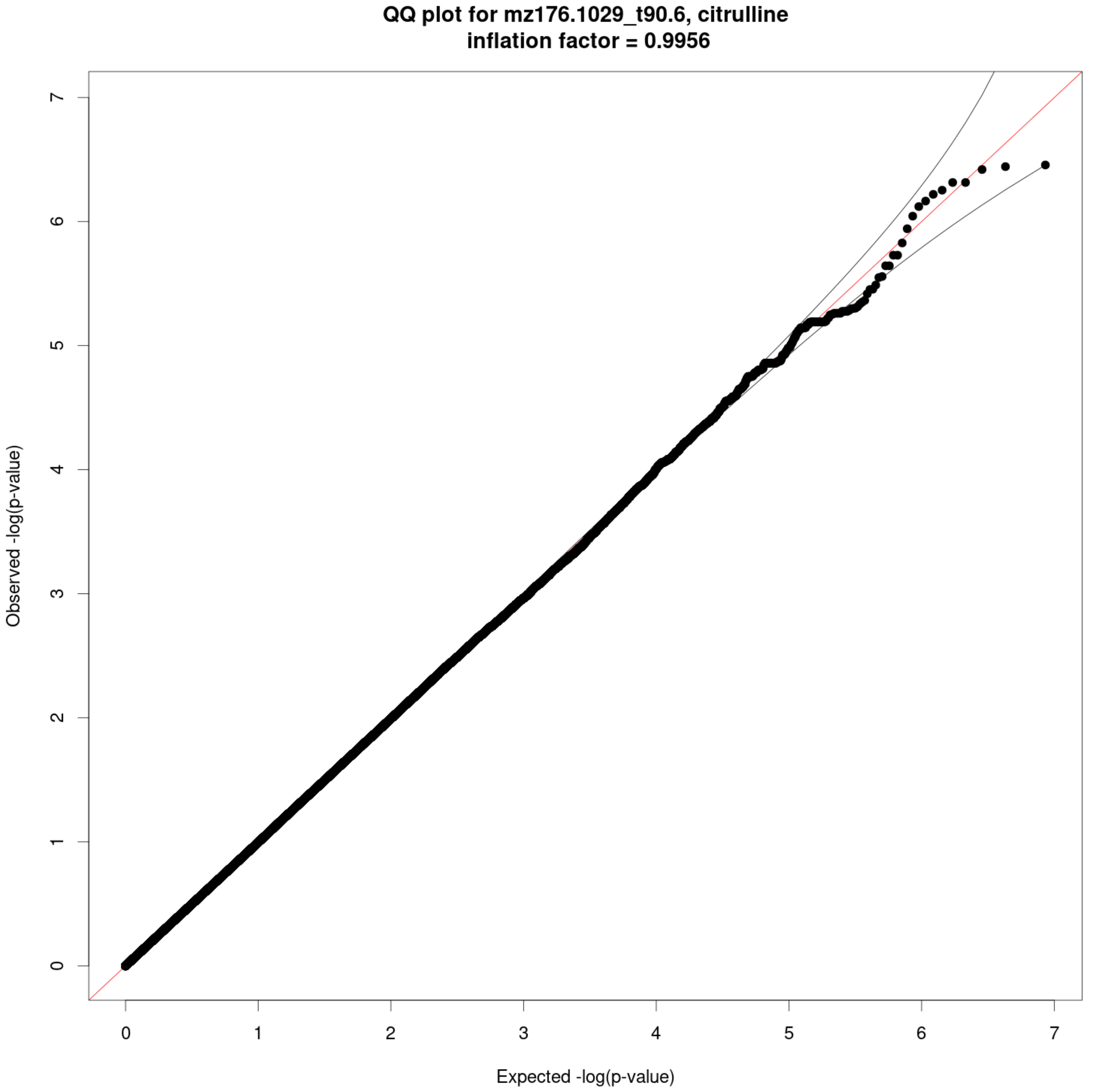
QQ plot for mz176.1029_t90.6, citrulline
inflation factor = 0.9956
Observed -log(p-value)
Expected -log(p-value)
0
1
2
3
4
5
6
7

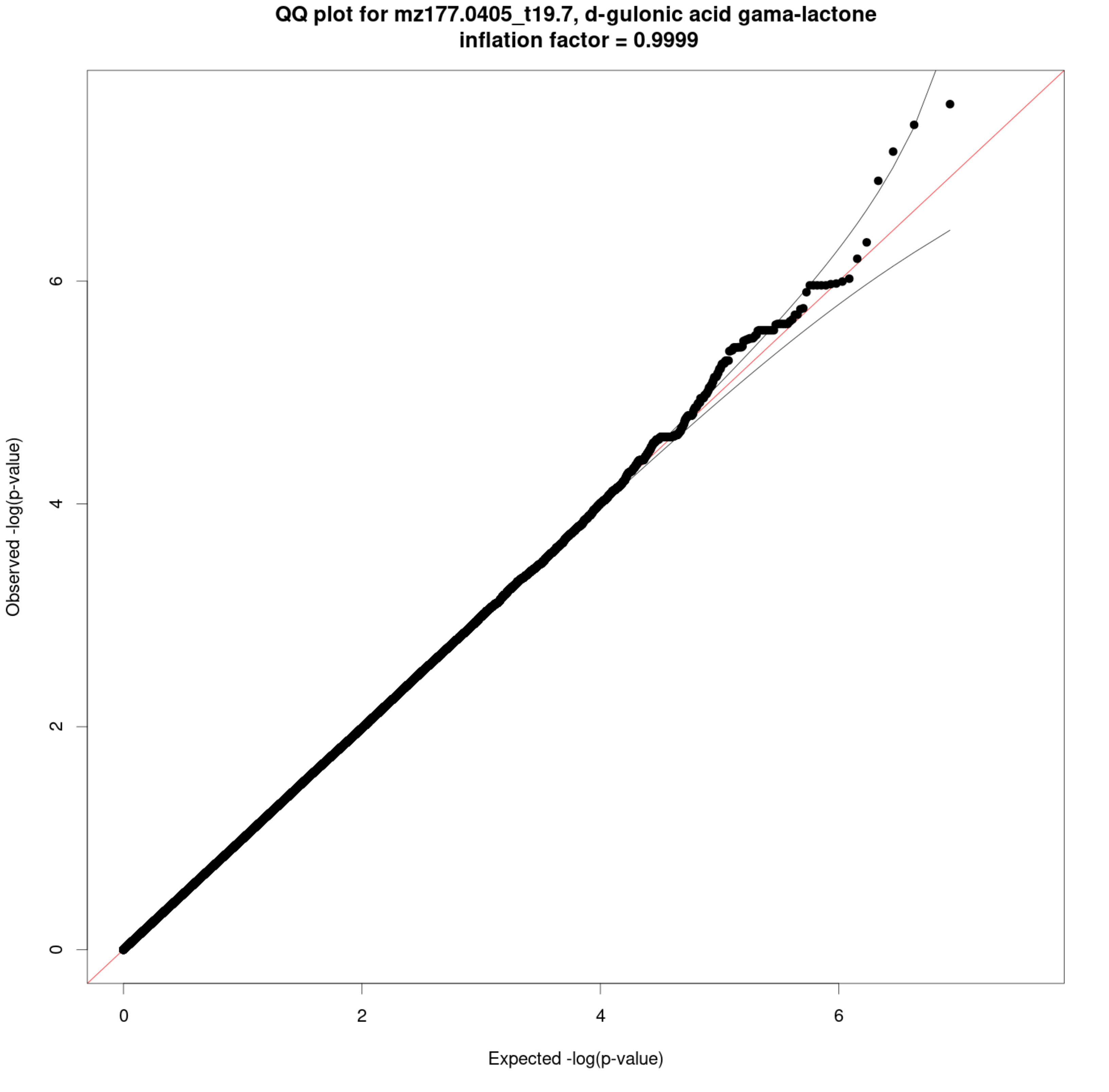
QQ plot for mz177.0405_t19.7, d-gulonic acid gama-lactone
inflation factor = 0.9999
Observed -log(p-value)
Expected -log(p-value)
0
2
4
6
0
2
4
6

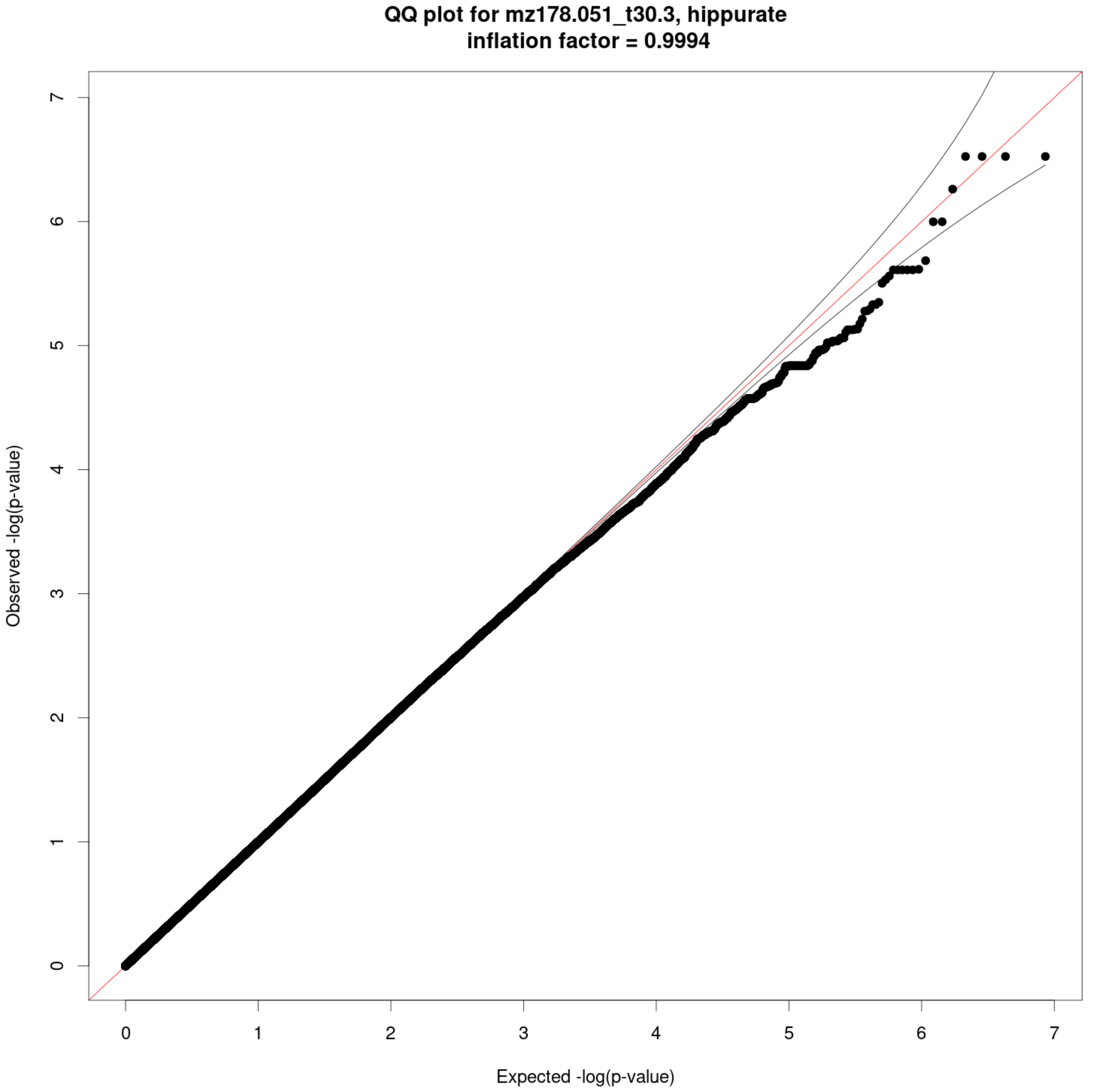
QQ plot for mz178.051_t30.3, hippurate
inflation factor = 0.9994
Observed -log(p-value)
Expected -log(p-value)
0
1
2
3
4
5
6
7

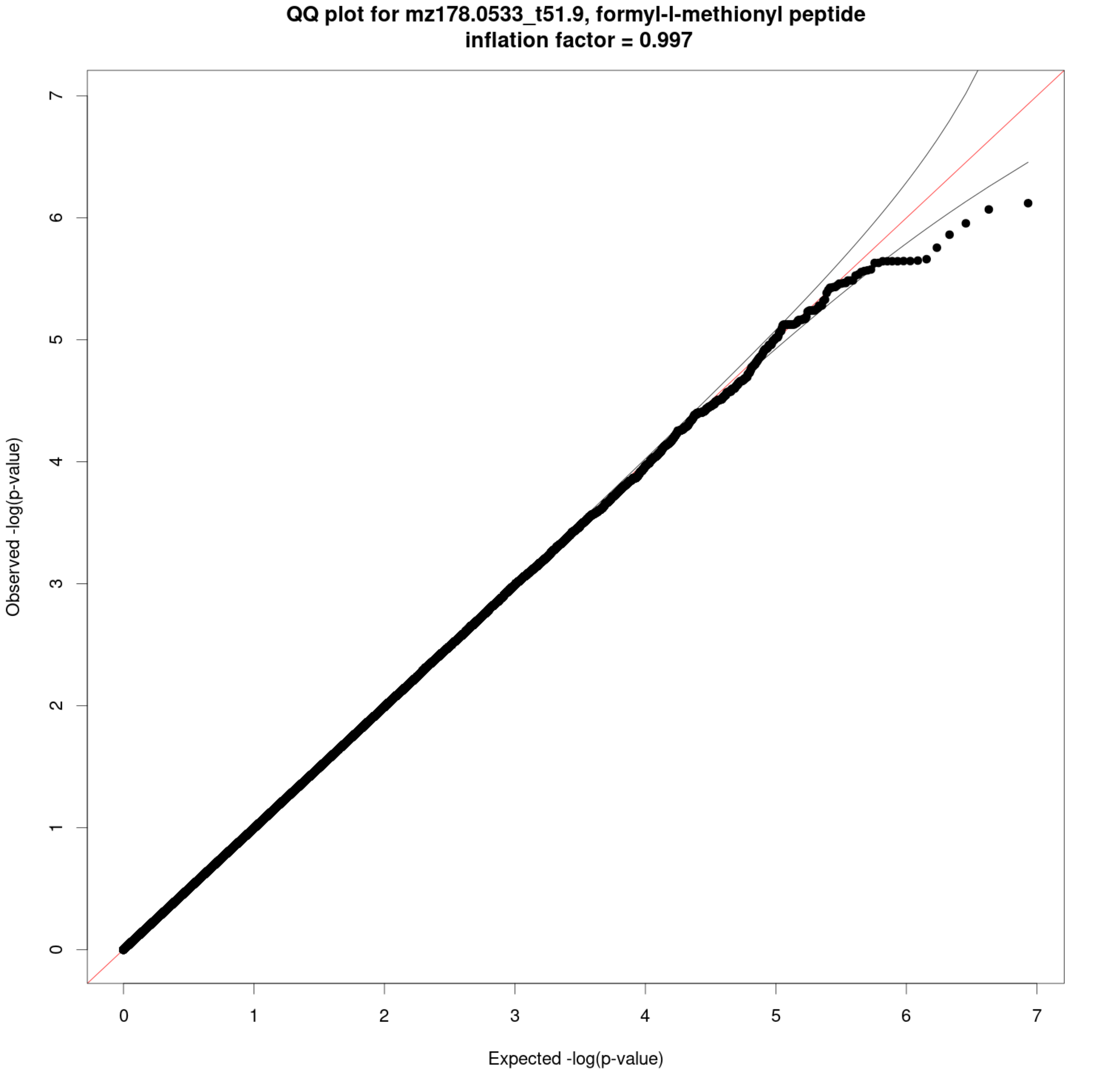
QQ plot for mz178.0533_t51.9, formyl-l-methionyl peptide
inflation factor = 0.997
Observed -log(p-value)
Expected -log(p-value)
0
1
2
3
4
5
6
7

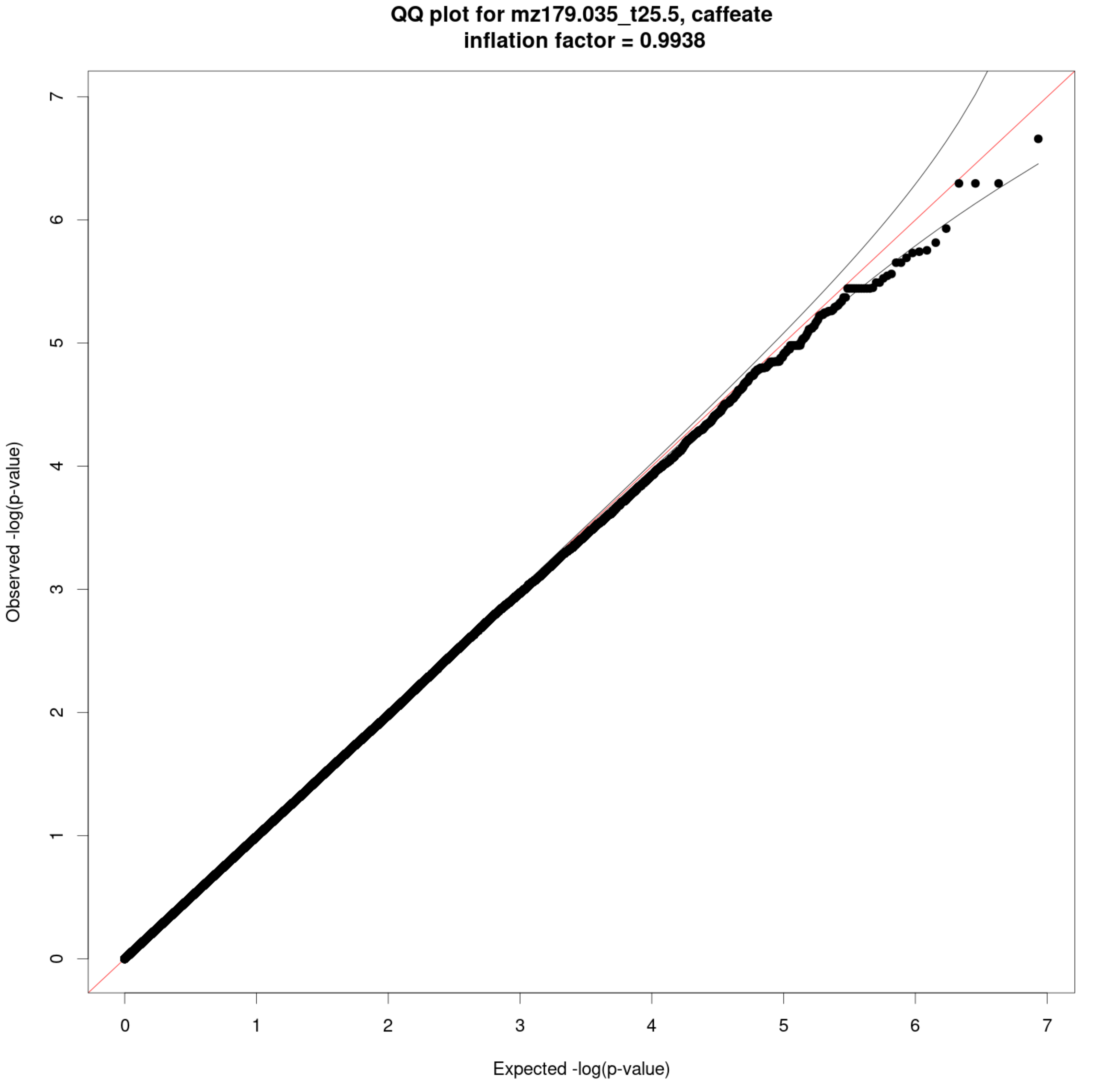
QQ plot for mz179.035_t25.5, caffeate
inflation factor = 0.9938
Observed -log(p-value)
Expected -log(p-value)
0
1
2
3
4
5
6
7

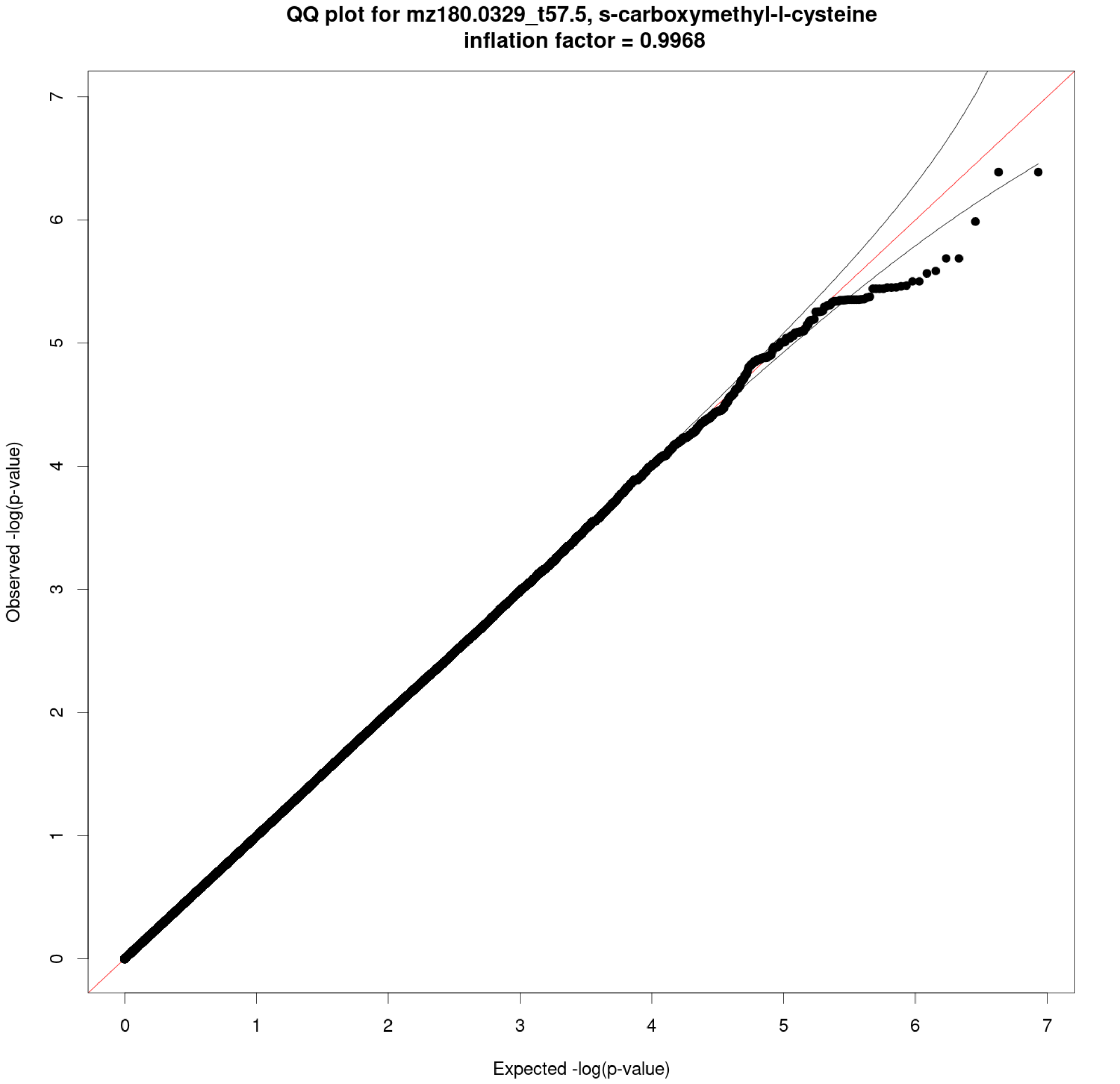
QQ plot for mz180.0329_t57.5, s-carboxymethyl-l-cysteine
inflation factor = 0.9968
Observed -log(p-value)
Expected -log(p-value)
0
1
2
3
4
5
6
7

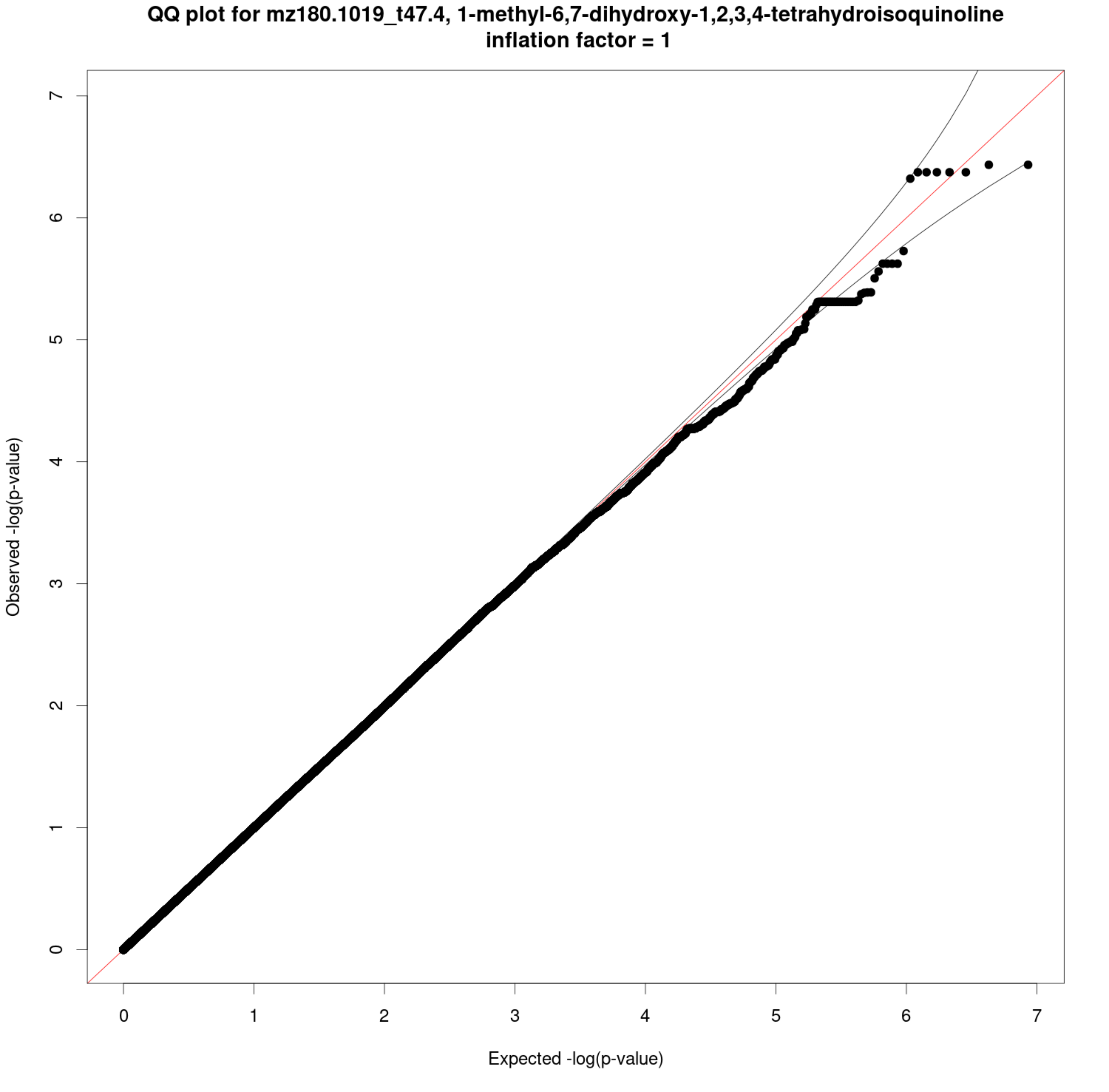
QQ plot for mz180.1019_t47.4, 1-methyl-6,7-dihydroxy-1,2,3,4-tetrahydroisoquinoline
inflation factor = 1
Observed -log(p-value)
Expected -log(p-value)
0
1
2
3
4
5
6
7

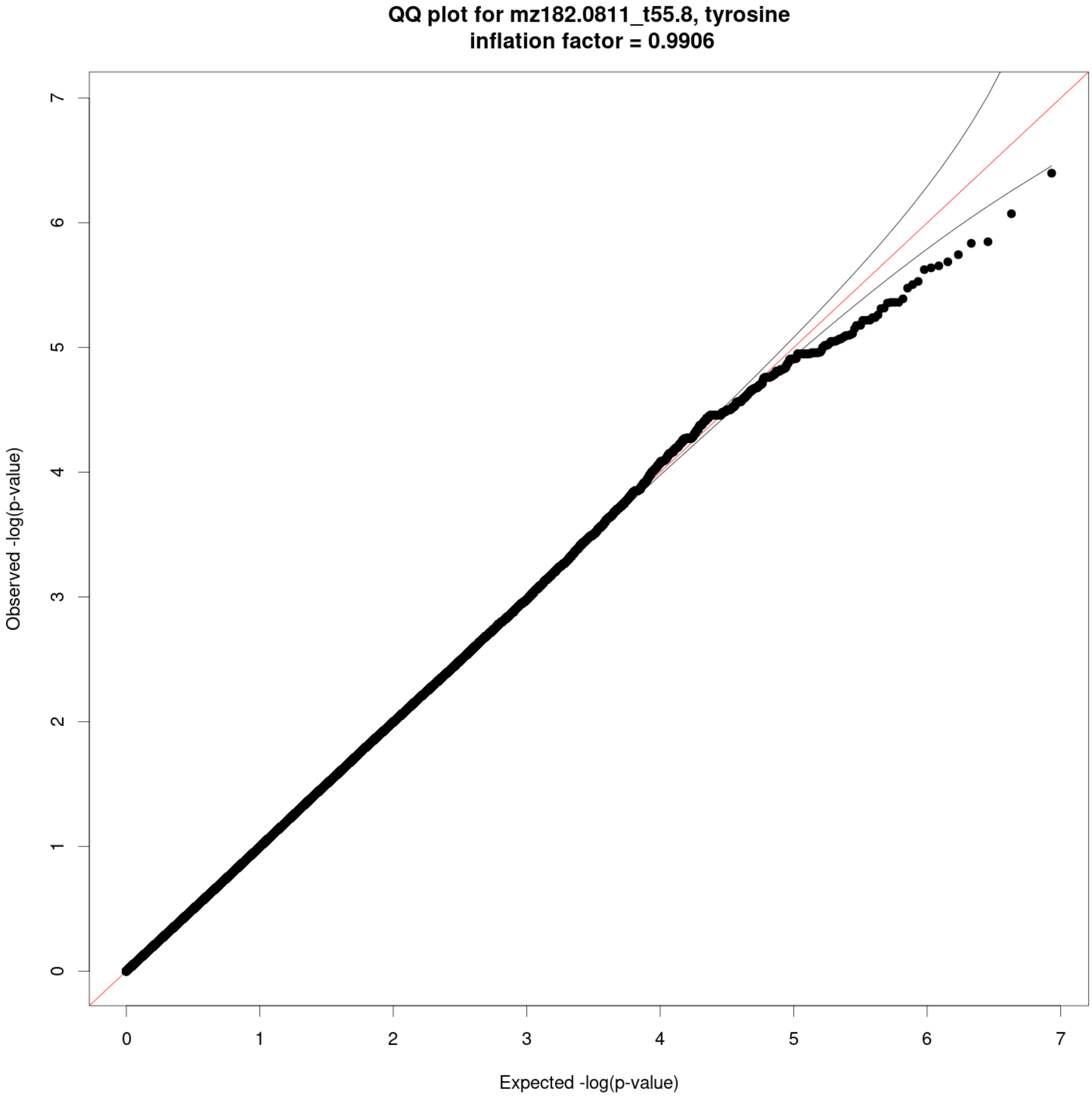
QQ plot for mz182.0811_t55.8, tyrosine
inflation factor = 0.9906
Observed -log(p-value)
Expected -log(p-value)
0
1
2
3
4
5
6
7

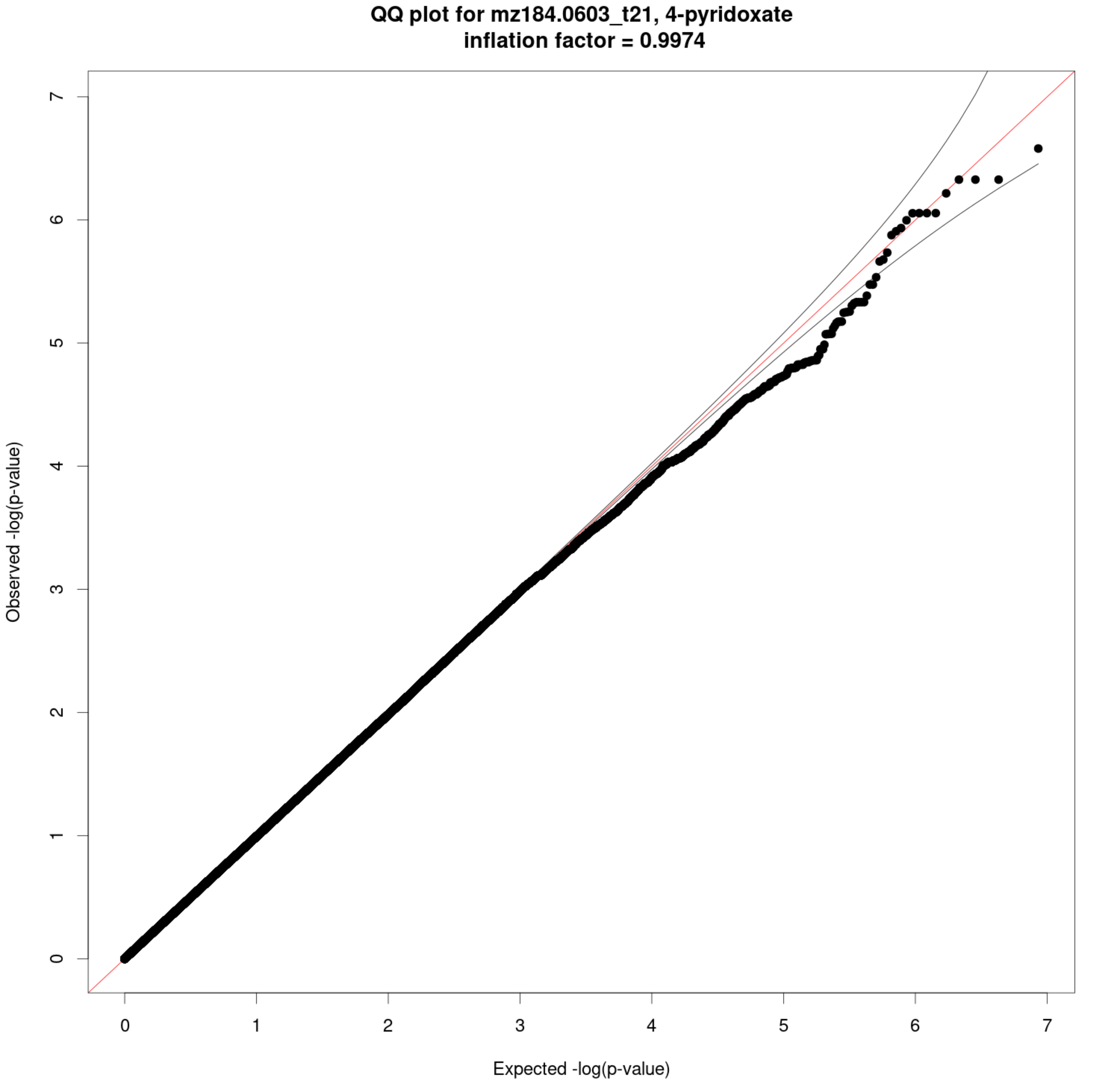
QQ plot for mz184.0603_t21, 4-pyridoxate
inflation factor = 0.9974
Observed -log(p-value)
Expected -log(p-value)
0
1
2
3
4
5
6
7

QQ plot for mz189.088_t31.9, ll-2,6-diaminoheptanedioate
inflation factor = 0.9939

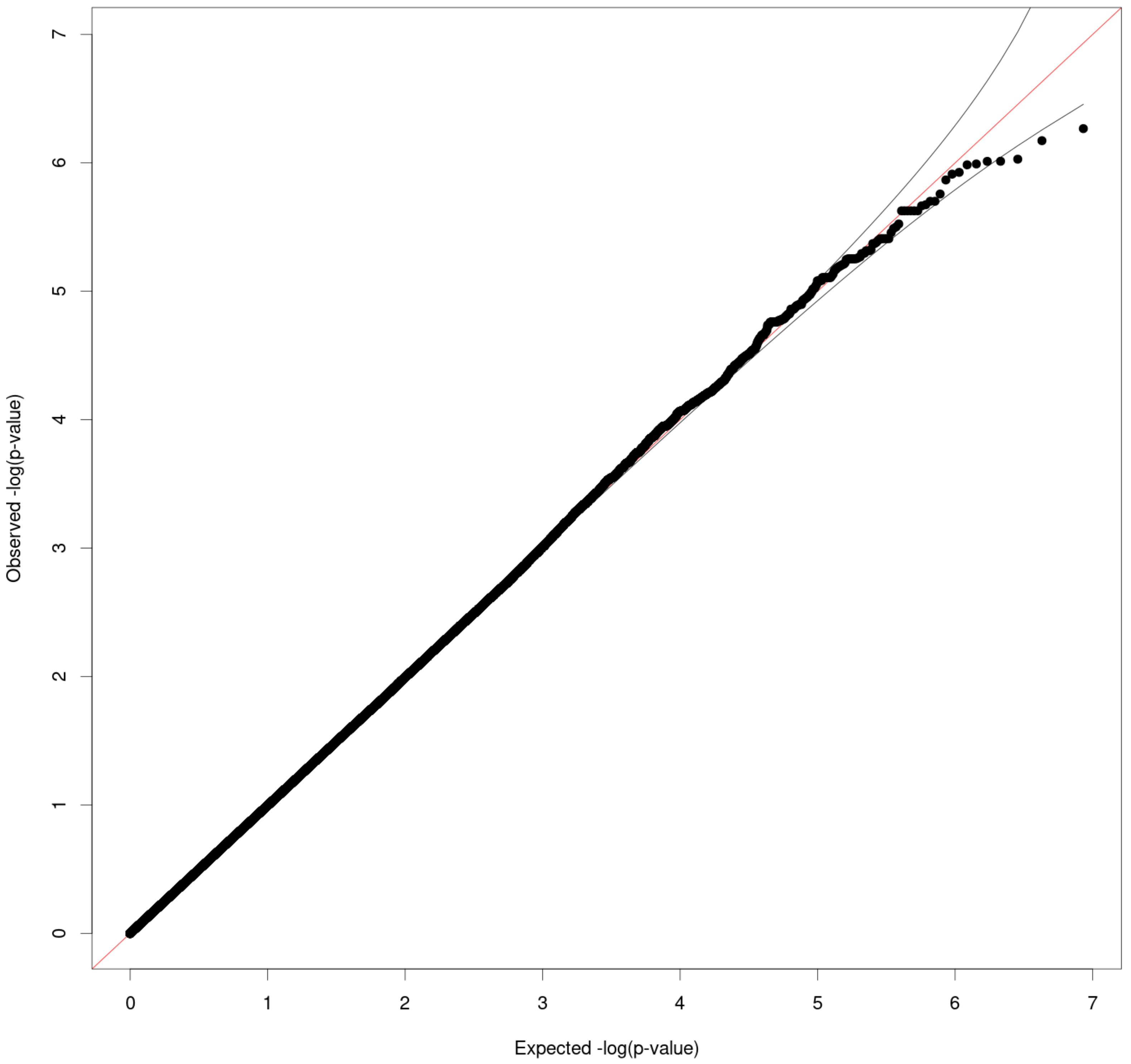

QQ plot for mz189.1233_t69.3, nalpha-acetyl-l-lysine
inflation factor = 0.9941

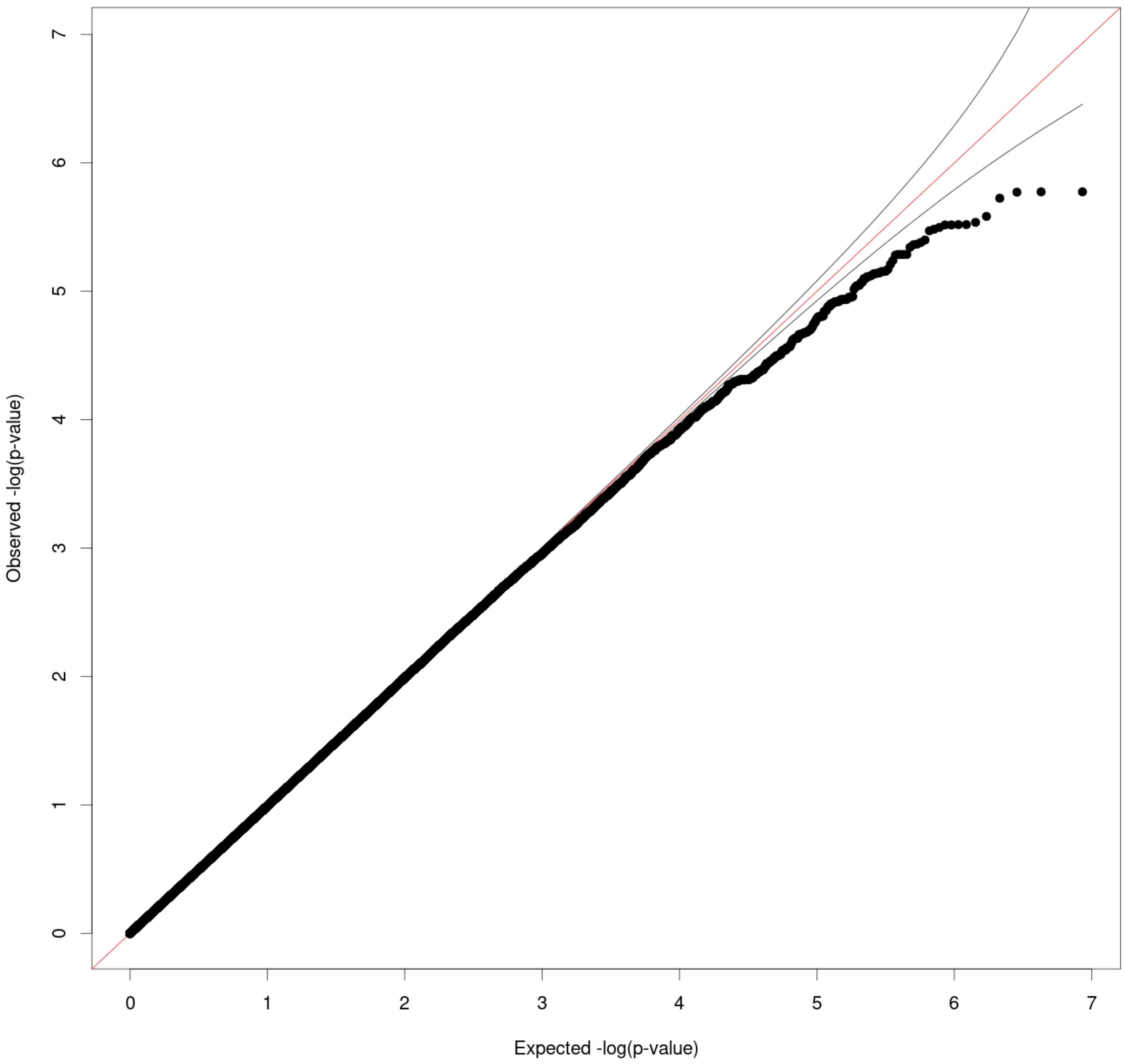

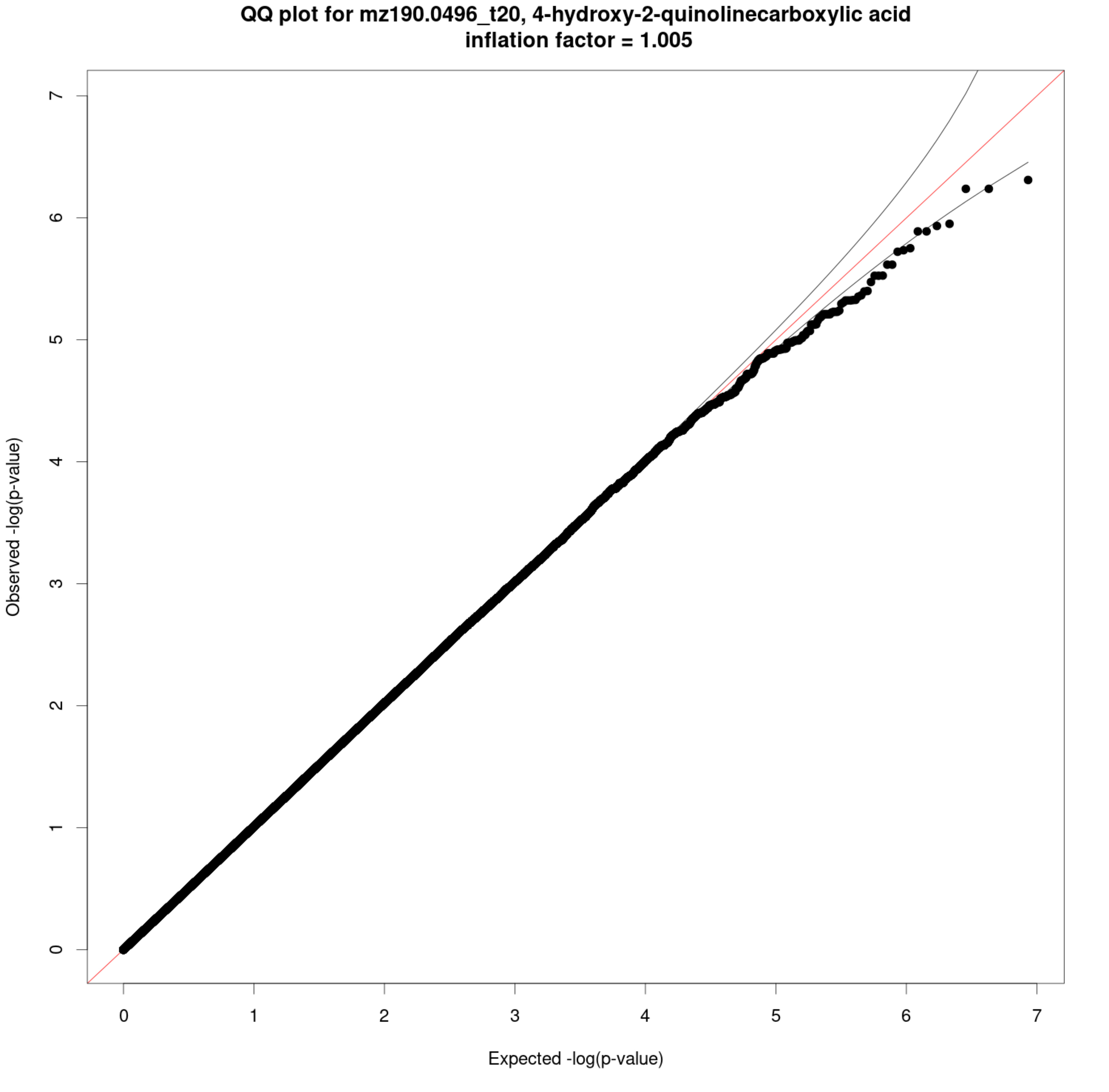
QQ plot for mz190.0496_t20, 4-hydroxy-2-quinolinecarboxylic acid
inflation factor = 1.005
Observed -log(p-value)
Expected -log(p-value)
0
1
2
3
4
5
6
7

**QQ plot for mz190.0544_t27.4, n-acetyl-dl-methionine
inflation factor = 0.9999**

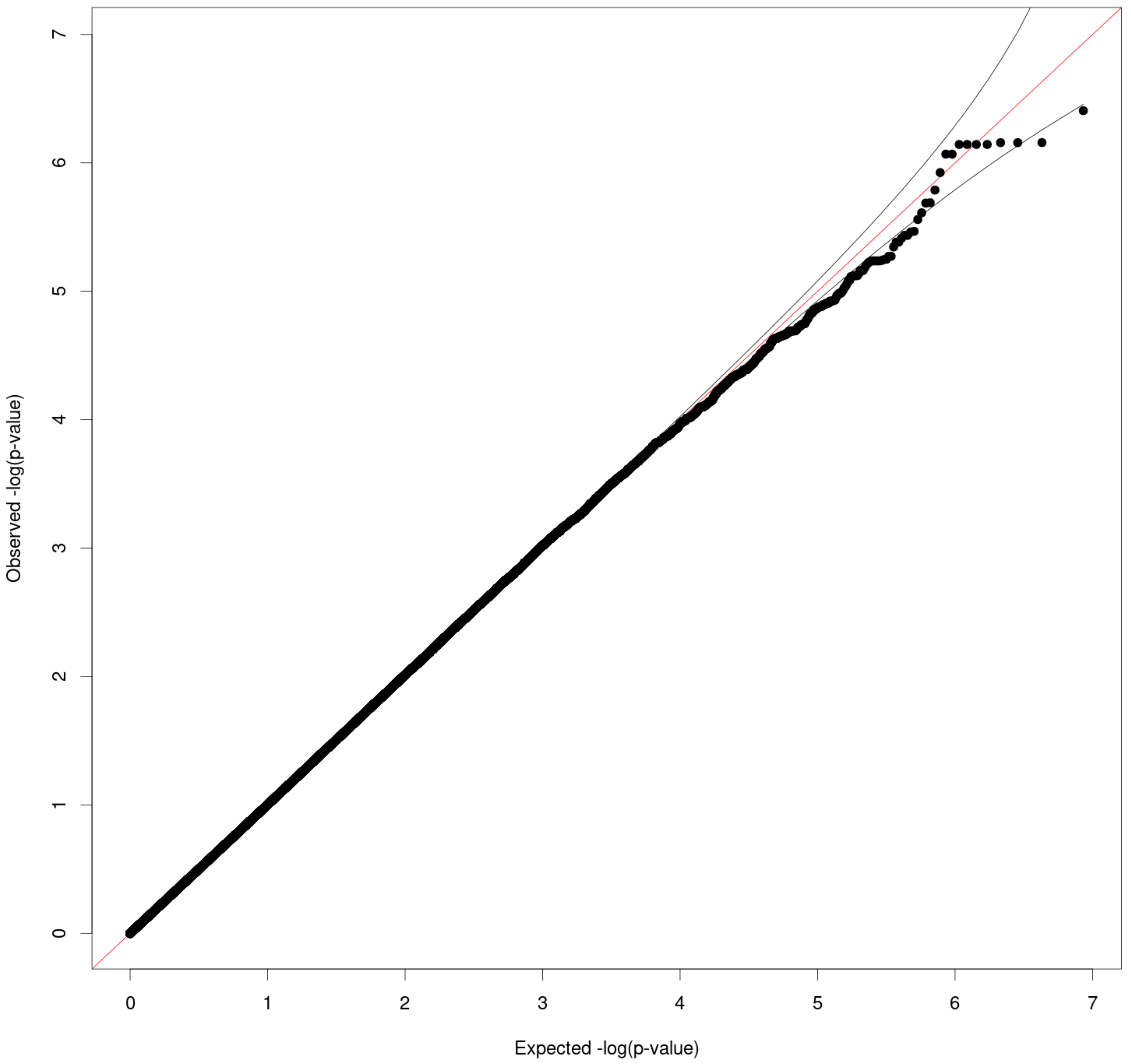

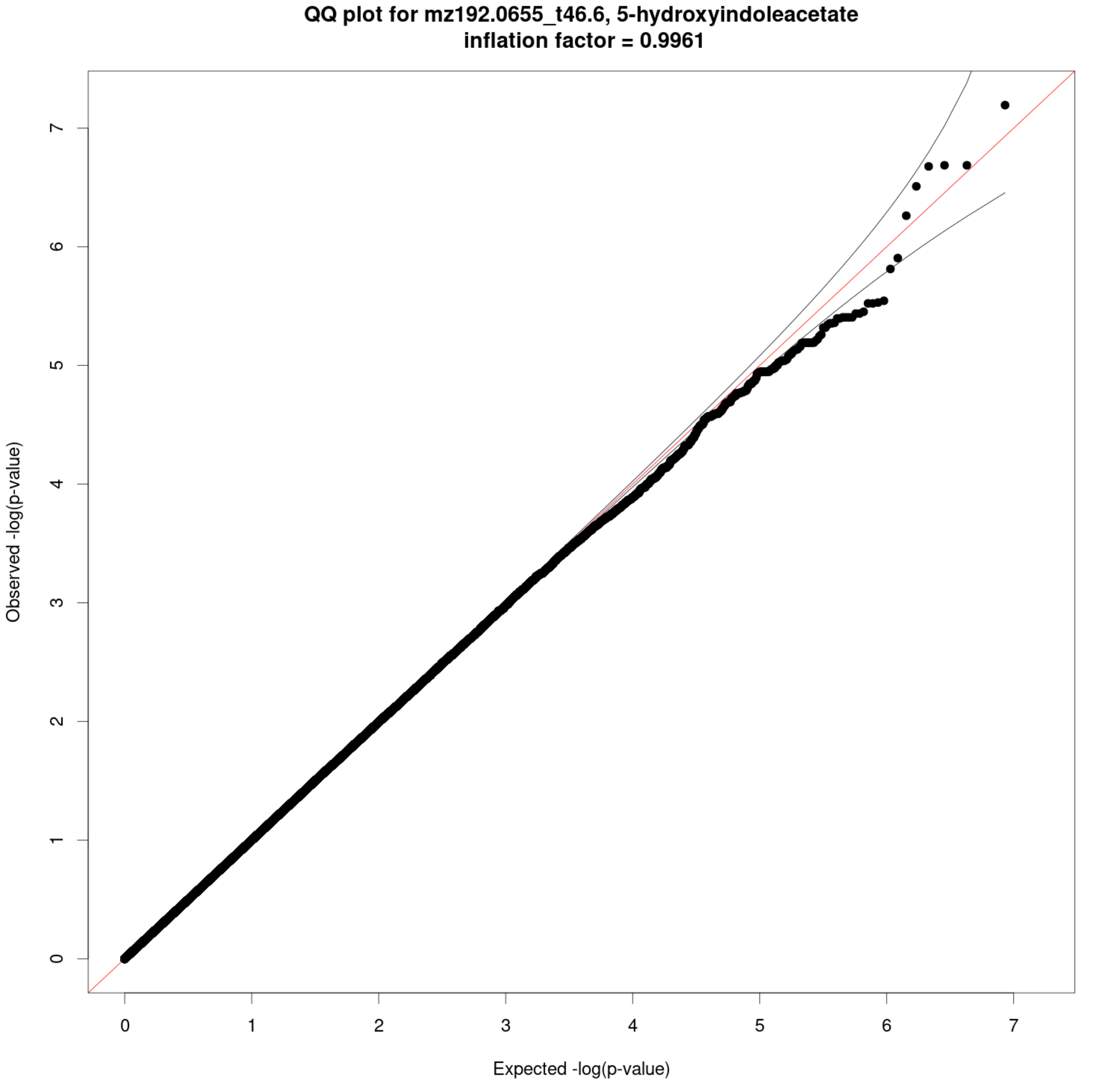
QQ plot for mz192.0655_t46.6, 5-hydroxyindoleacetate
inflation factor = 0.9961
Observed -log(p-value)
Expected -log(p-value)
0
1
2
3
4
5
6
7

QQ plot for mz195.0505_t48.3, d-glucuronic acid
inflation factor = 1.006

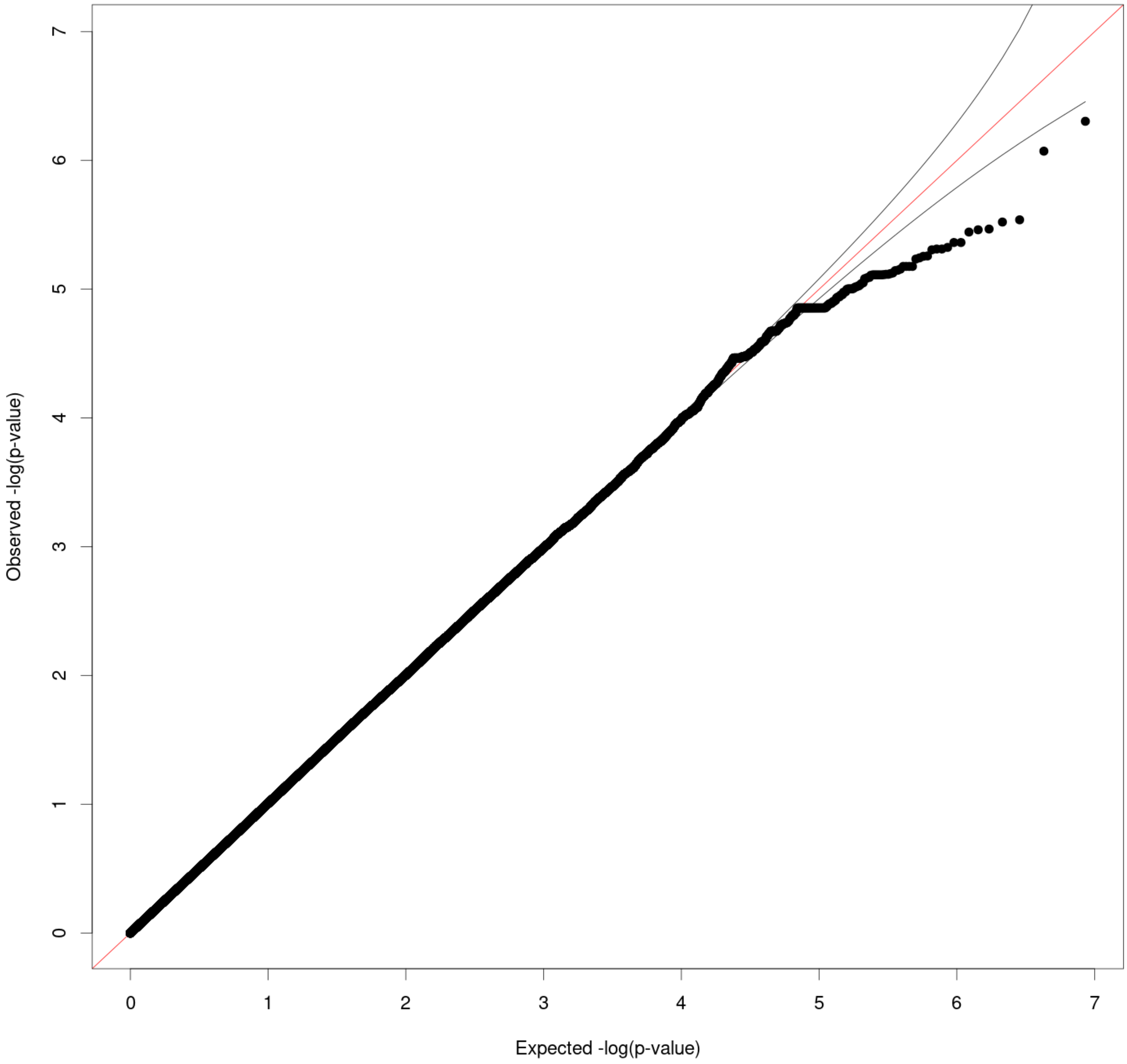

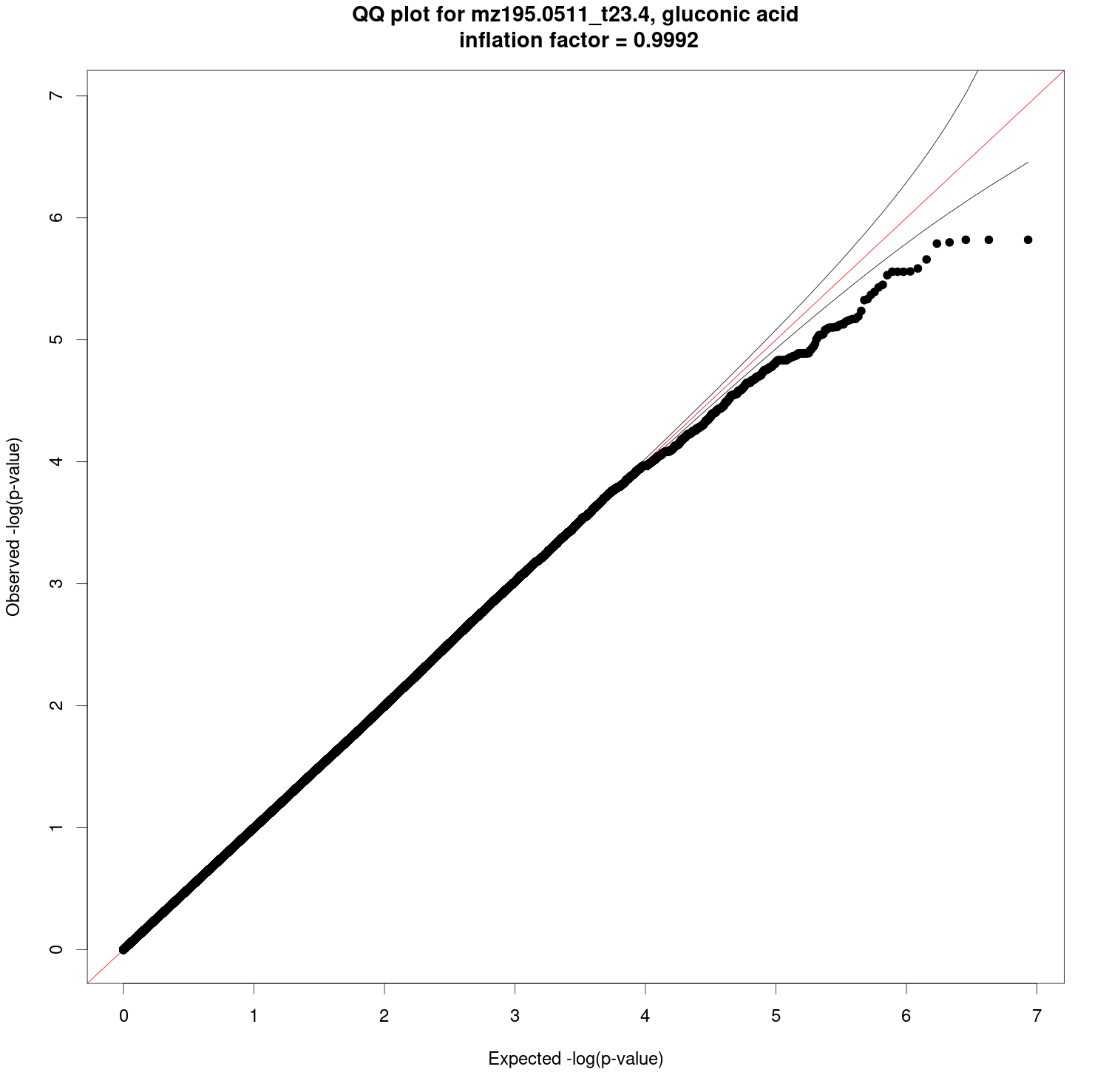
QQ plot for mz195.0511_t23.4, gluconic acid
inflation factor = 0.9992
Observed -log(p-value)
Expected -log(p-value)
0
1
2
3
4
5
6
7

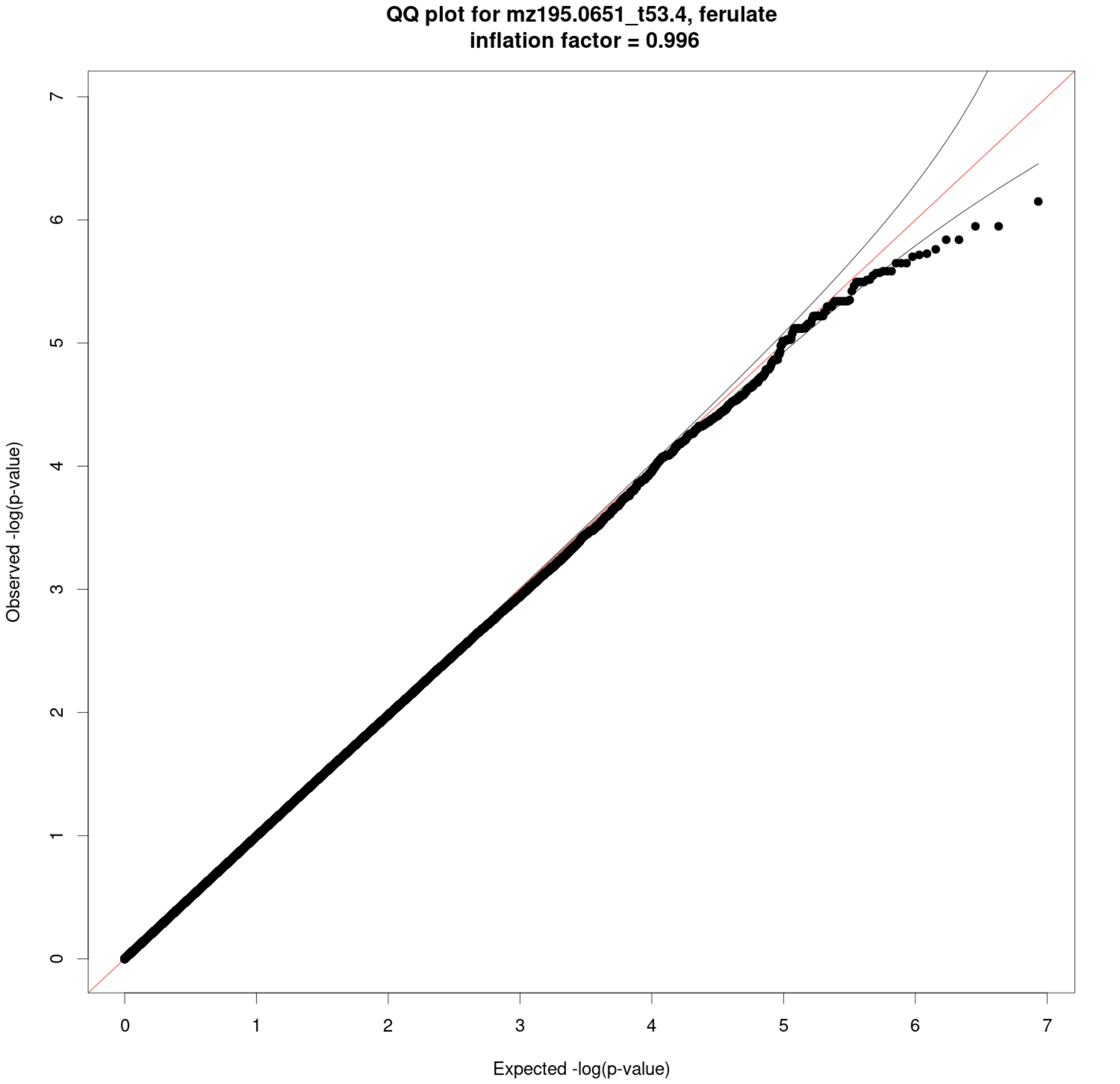
QQ plot for mz195.0651_t53.4, ferulate
inflation factor = 0.996
Observed -log(p-value)
Expected -log(p-value)
0
1
2
3
4
5
6
7

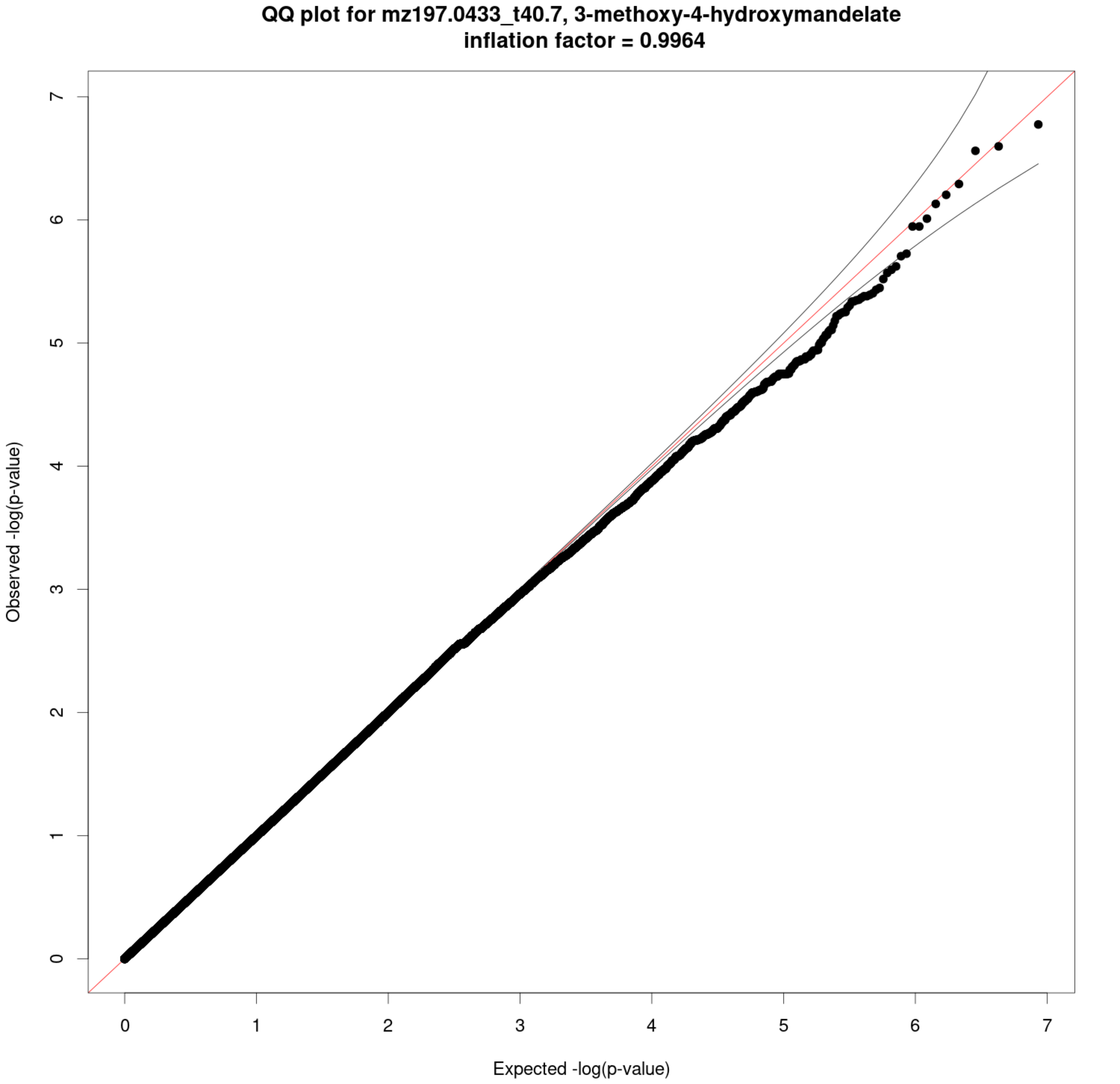
QQ plot for mz197.0433_t40.7, 3-methoxy-4-hydroxymandelate
inflation factor = 0.9964
Observed -log(p-value)
Expected -log(p-value)
0
1
2
3
4
5
6
7

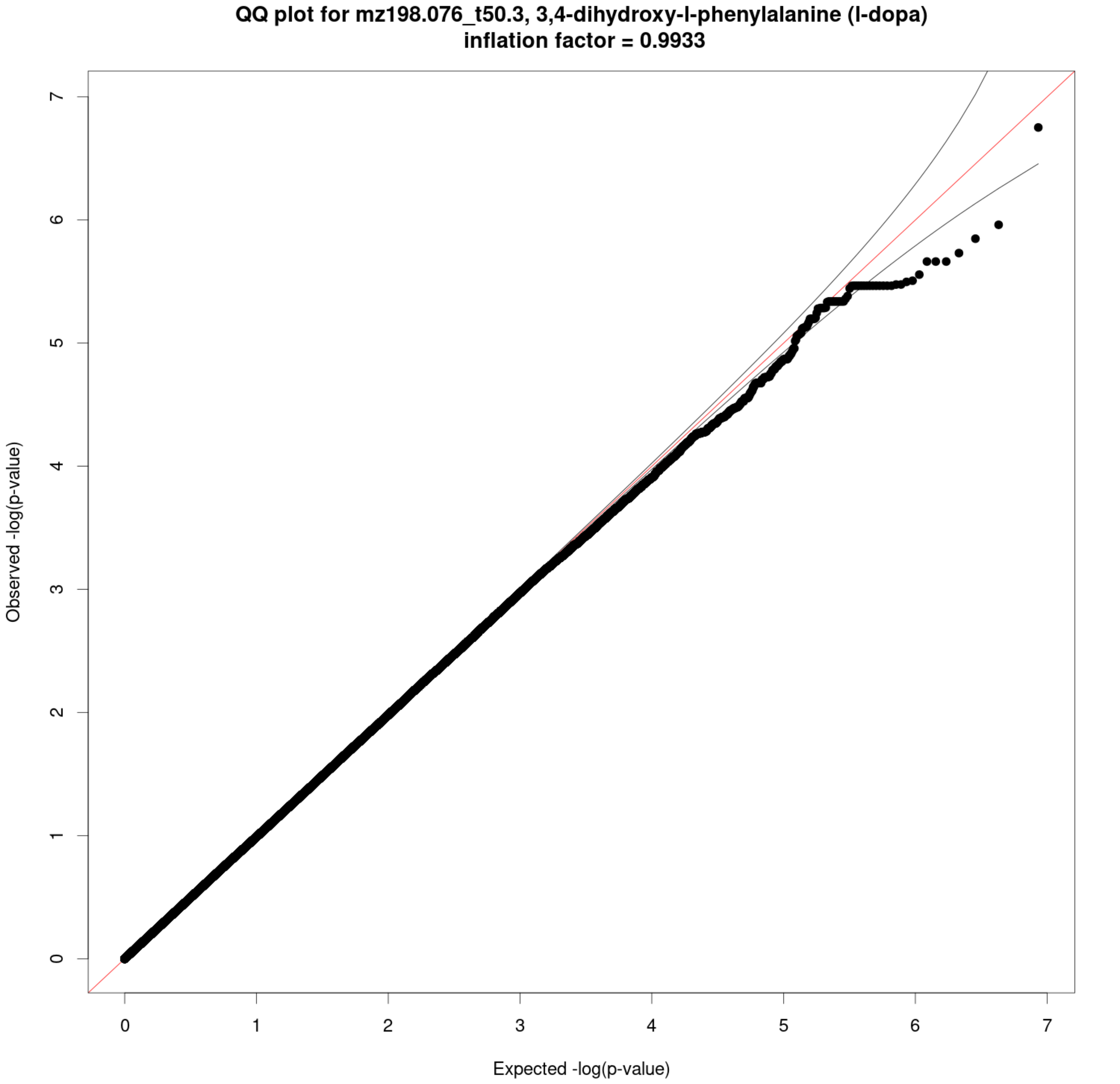
QQ plot for mz198.076_t50.3, 3,4-dihydroxy-l-phenylalanine (l-dopa)
inflation factor = 0.9933
Observed -log(p-value)
Expected -log(p-value)
0
1
2
3
4
5
6
7

QQ plot for mz199.1704_t152.3, lauric acid
inflation factor = 1

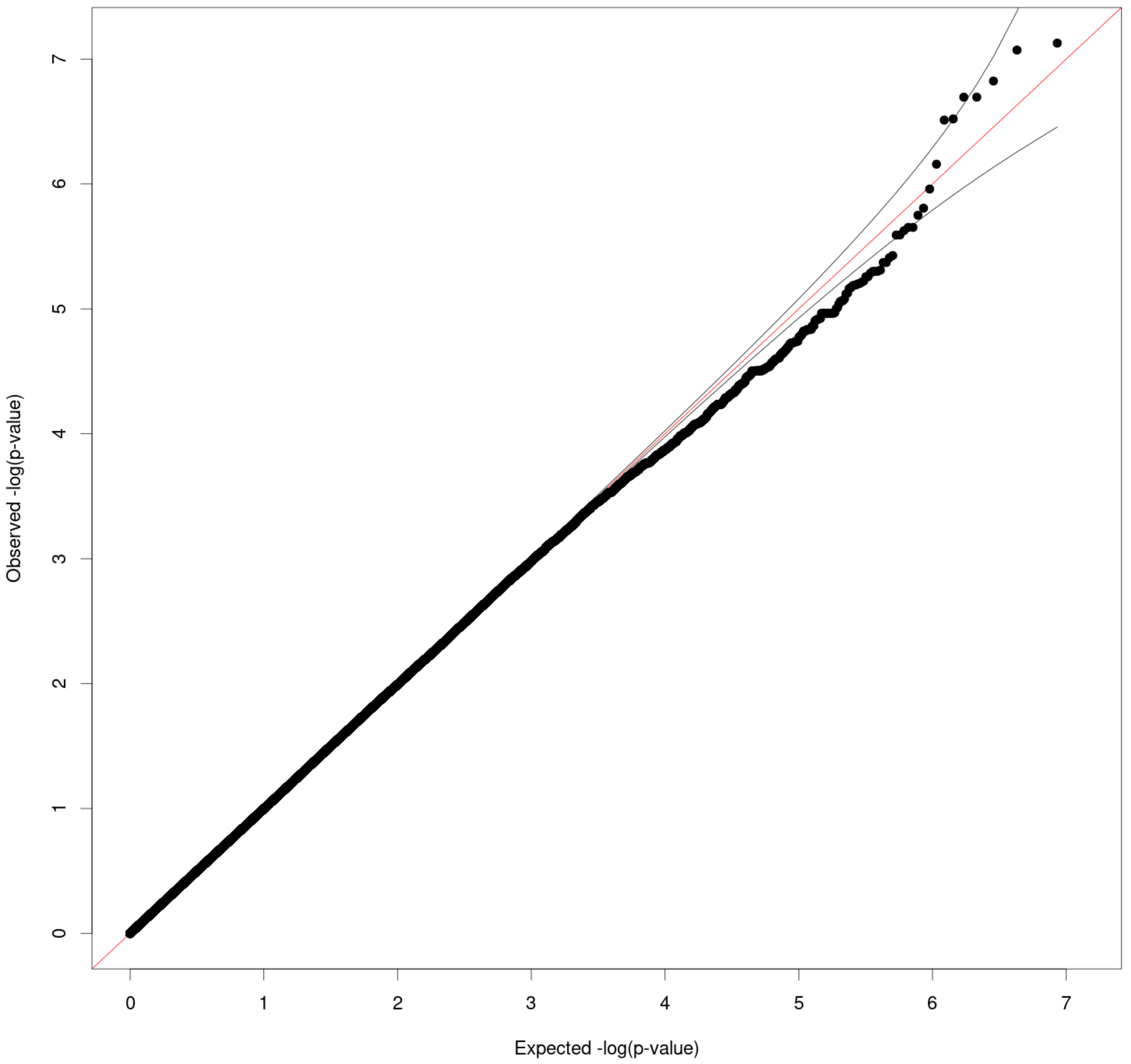

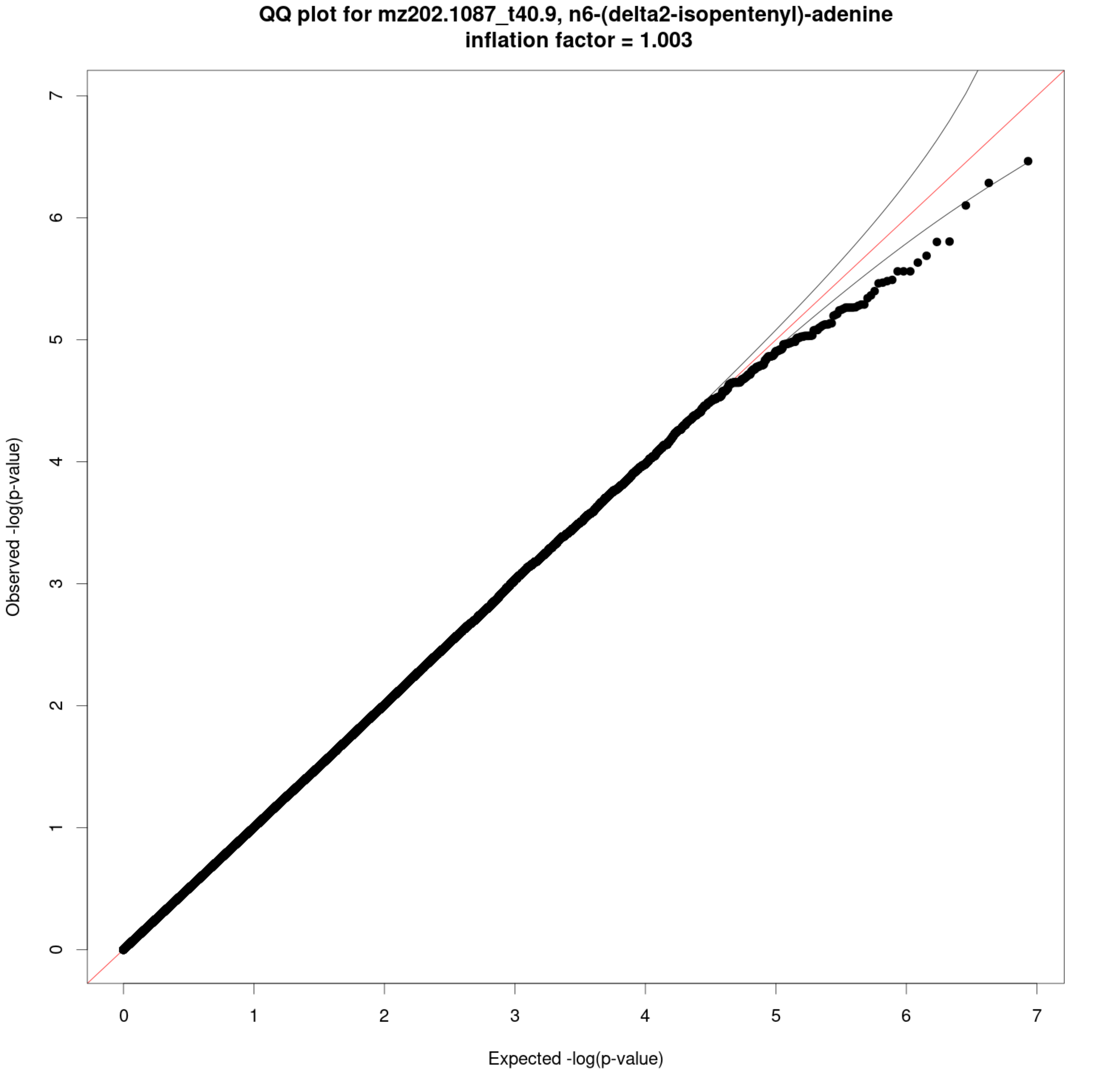
QQ plot for mz202.1087_t40.9, n6-(delta2-isopentenyl)-adenine
inflation factor = 1.003
Observed -log(p-value)
Expected -log(p-value)
0
1
2
3
4
5
6
7

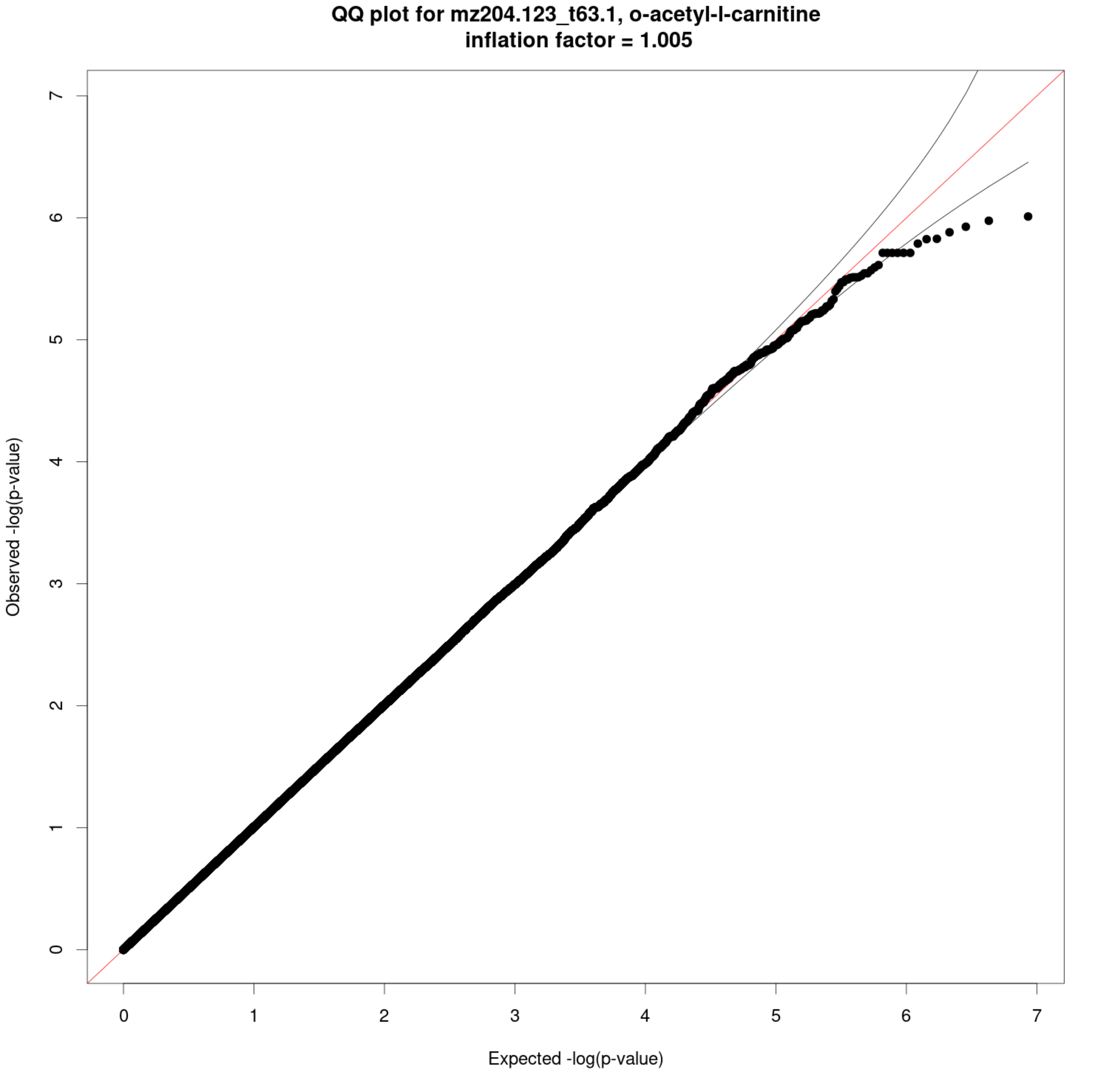
QQ plot for mz204.123_t63.1, o-acetyl-l-carnitine
inflation factor = 1.005
Observed -log(p-value)
Expected -log(p-value)
0
1
2
3
4
5
6
7

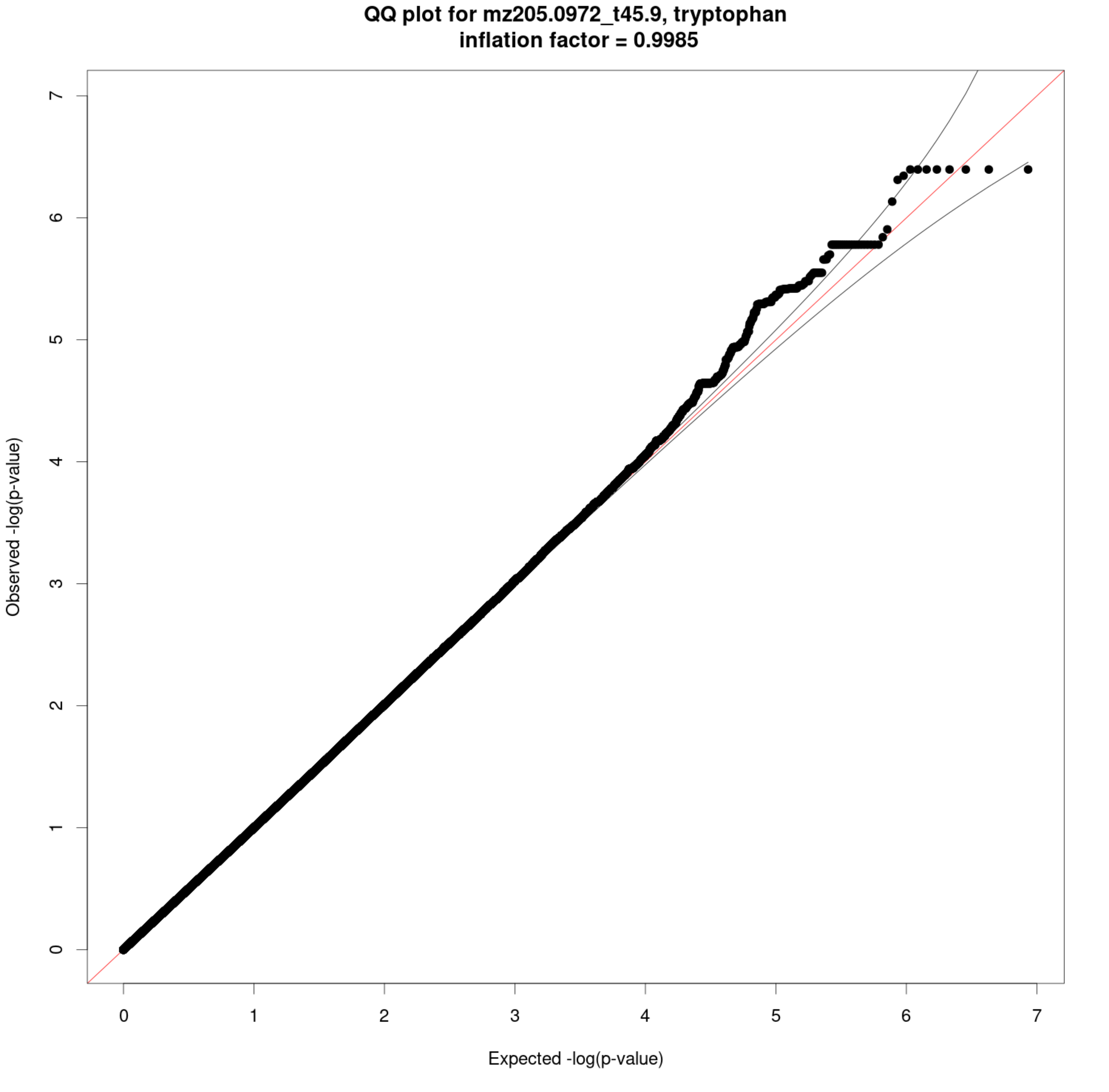
QQ plot for mz205.0972_t45.9, tryptophan
inflation factor = 0.9985
Observed -log(p-value)
Expected -log(p-value)
0
1
2
3
4
5
6
7

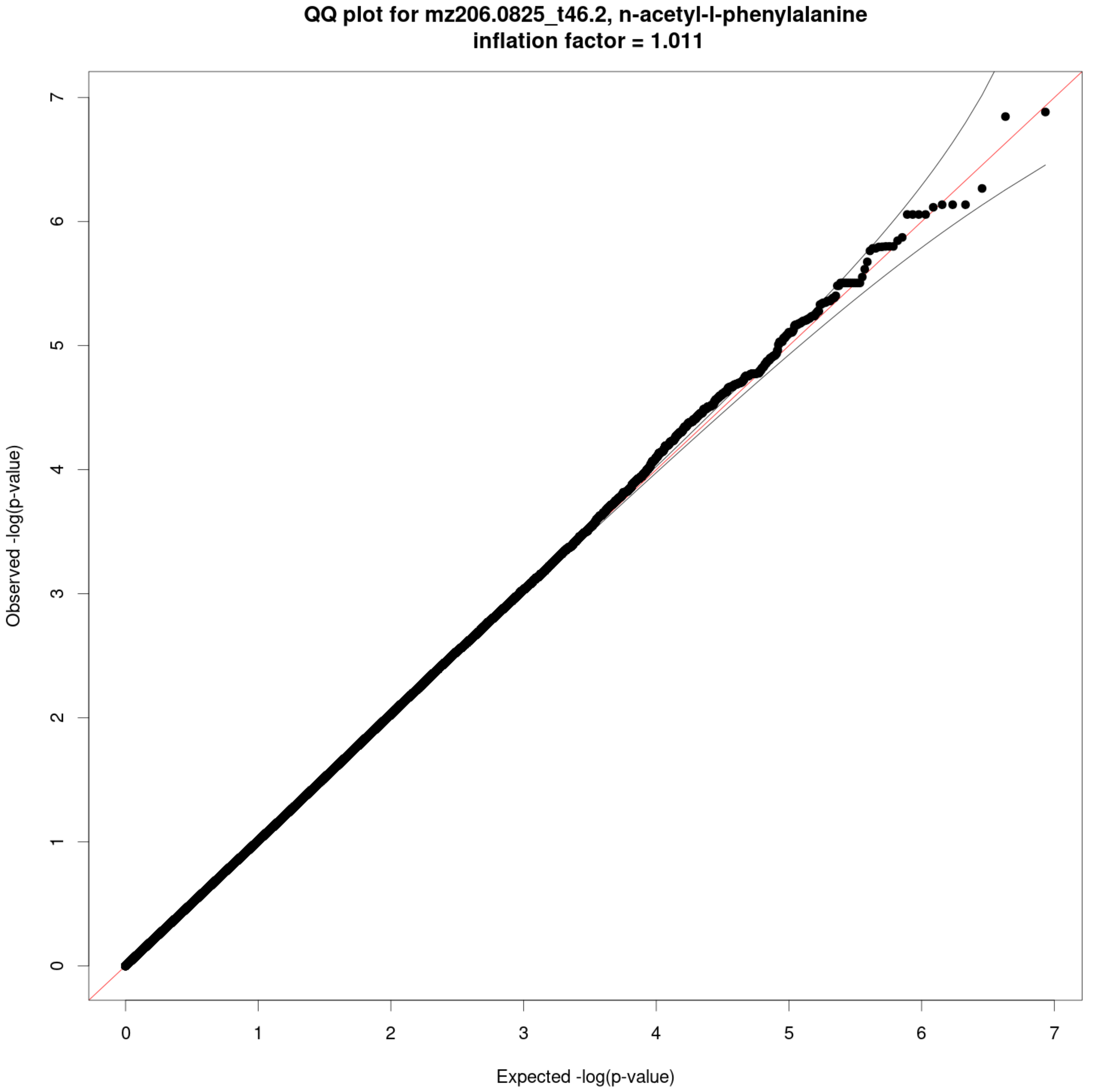
QQ plot for mz206.0825_t46.2, n-acetyl-l-phenylalanine
inflation factor = 1.011
Observed -log(p-value)
Expected -log(p-value)
0
1
2
3
4
5
6
7

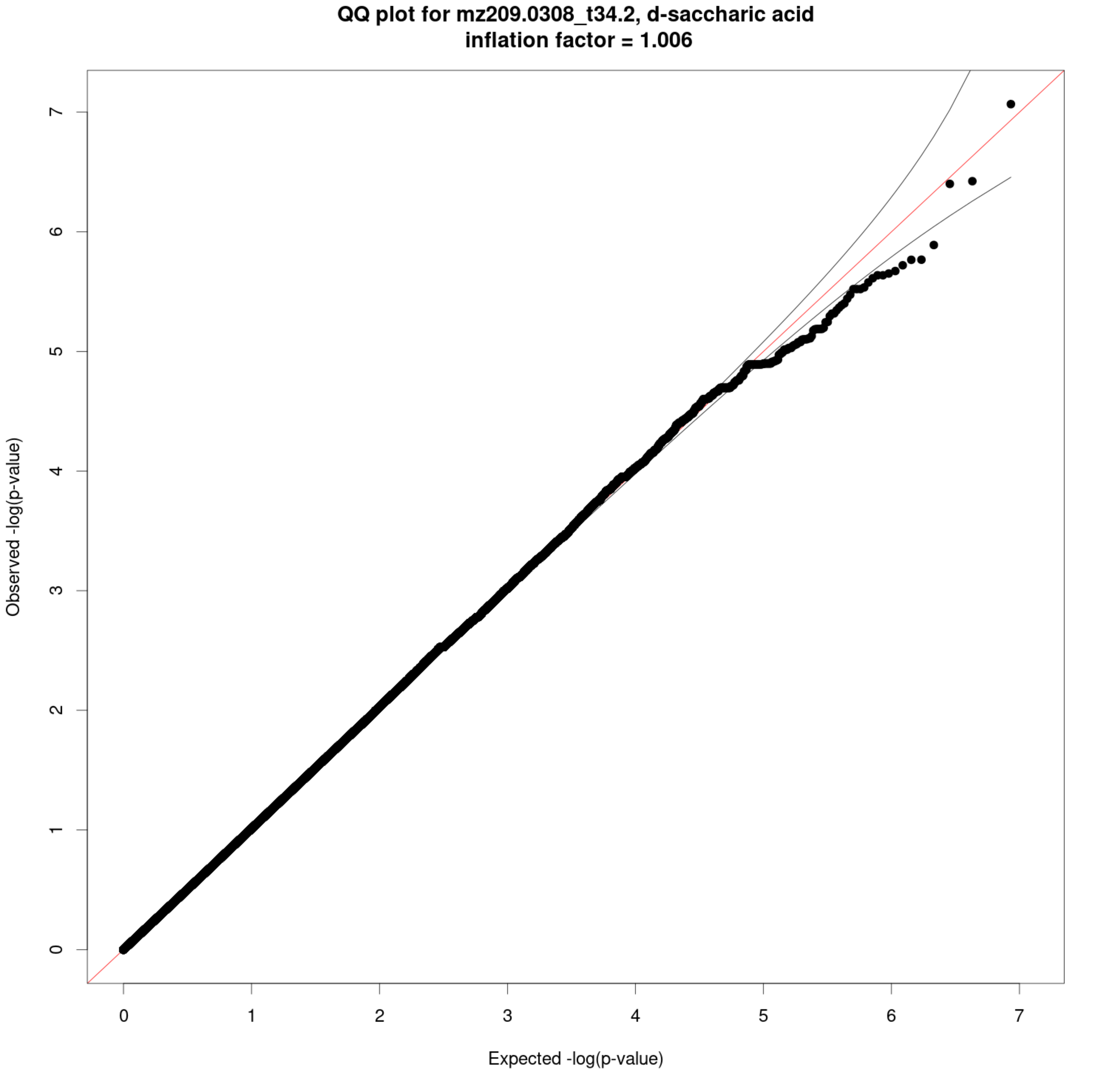
QQ plot for mz209.0308_t34.2, d-saccharic acid
inflation factor = 1.006
Observed -log(p-value)
Expected -log(p-value)
0
1
2
3
4
5
6
7

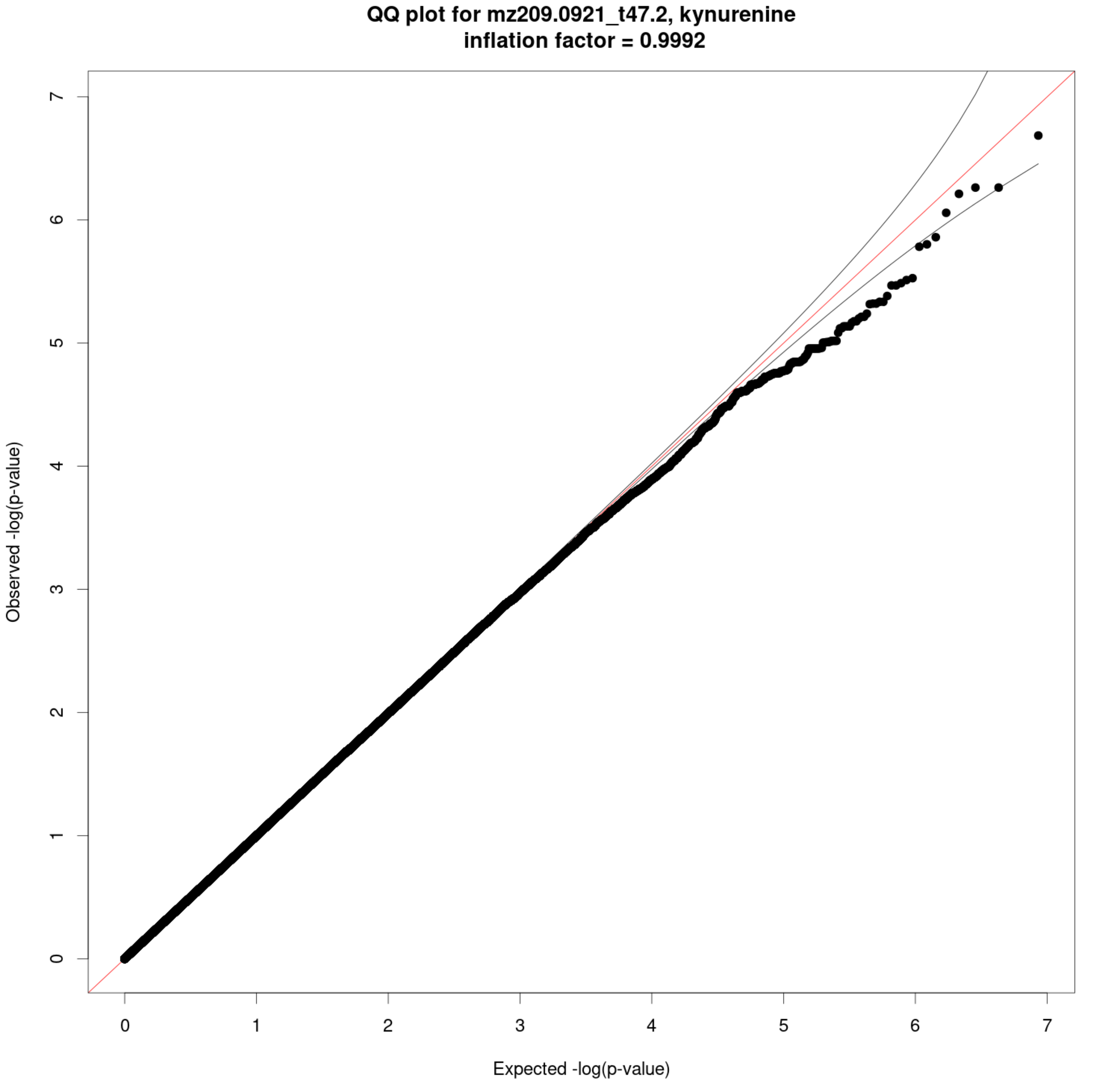
QQ plot for mz209.0921_t47.2, kynurenine
inflation factor = 0.9992
Observed -log(p-value)
Expected -log(p-value)
0
1
2
3
4
5
6
7

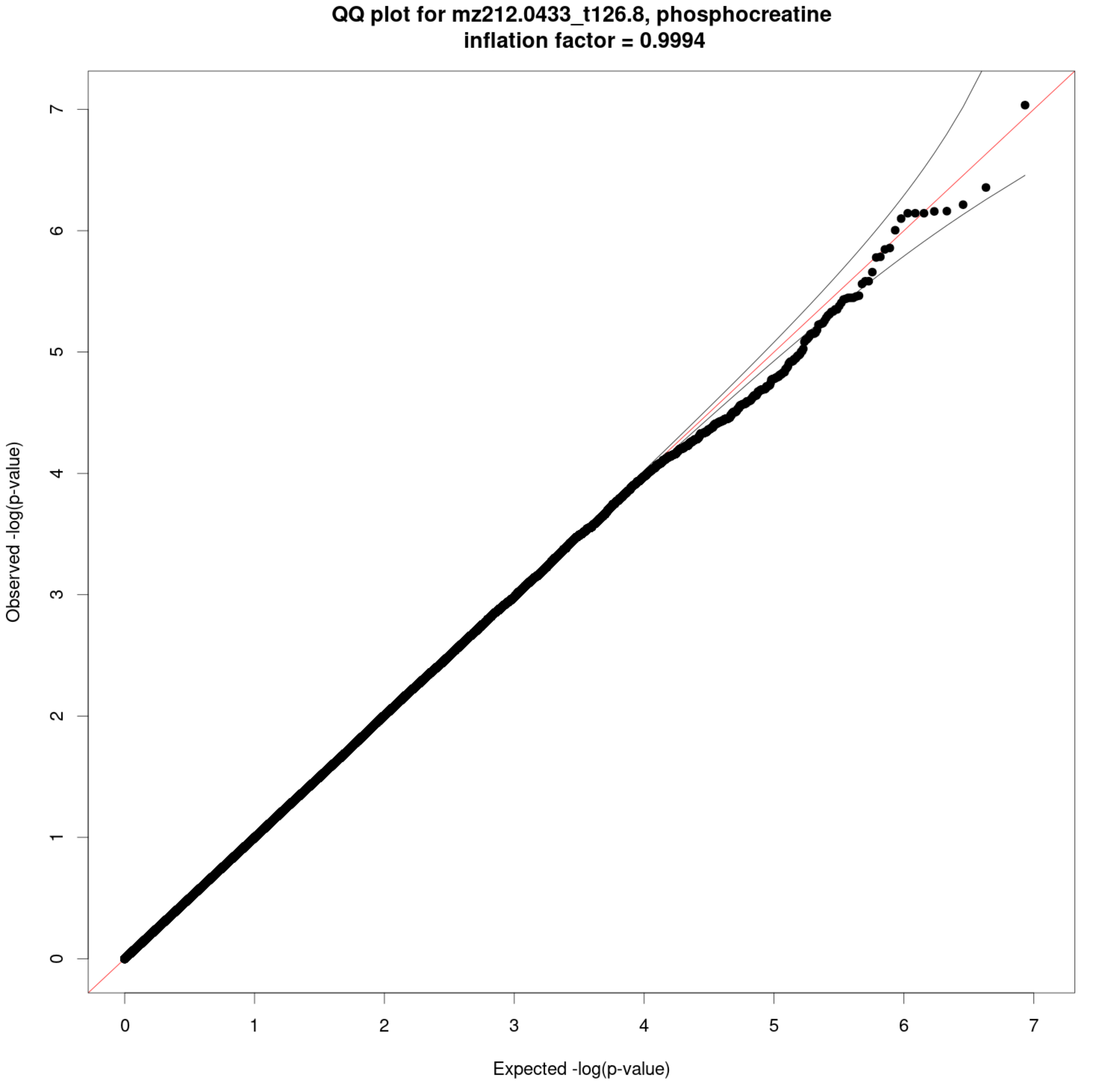
QQ plot for mz212.0433_t126.8, phosphocreatine
inflation factor = 0.9994
Observed -log(p-value)
Expected -log(p-value)
0
1
2
3
4
5
6
7
0
1
2
3
4
5
6
7

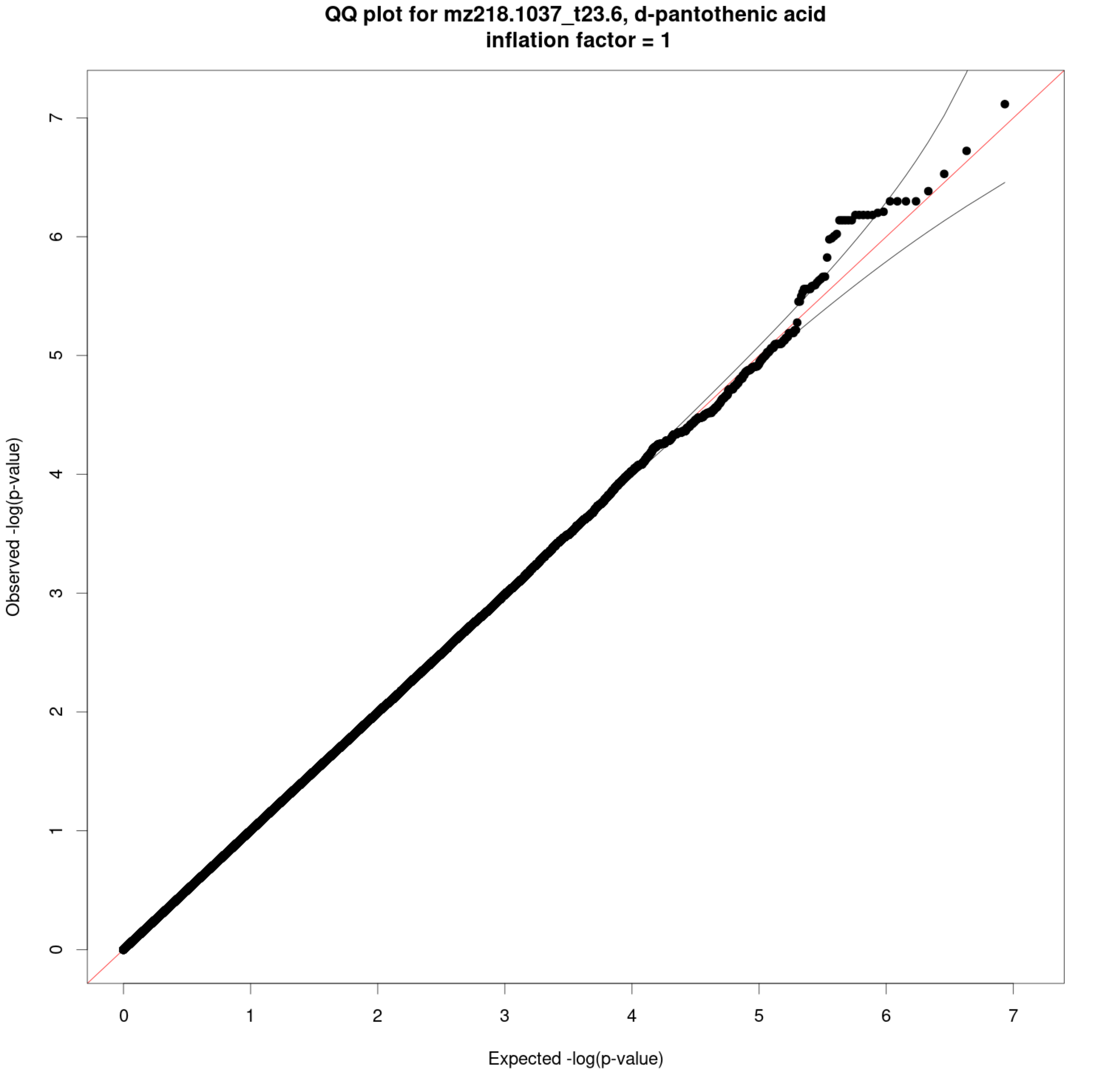
QQ plot for mz218.1037_t23.6, d-pantothenic acid
inflation factor = 1
Observed -log(p-value)
Expected -log(p-value)
0
1
2
3
4
5
6
7

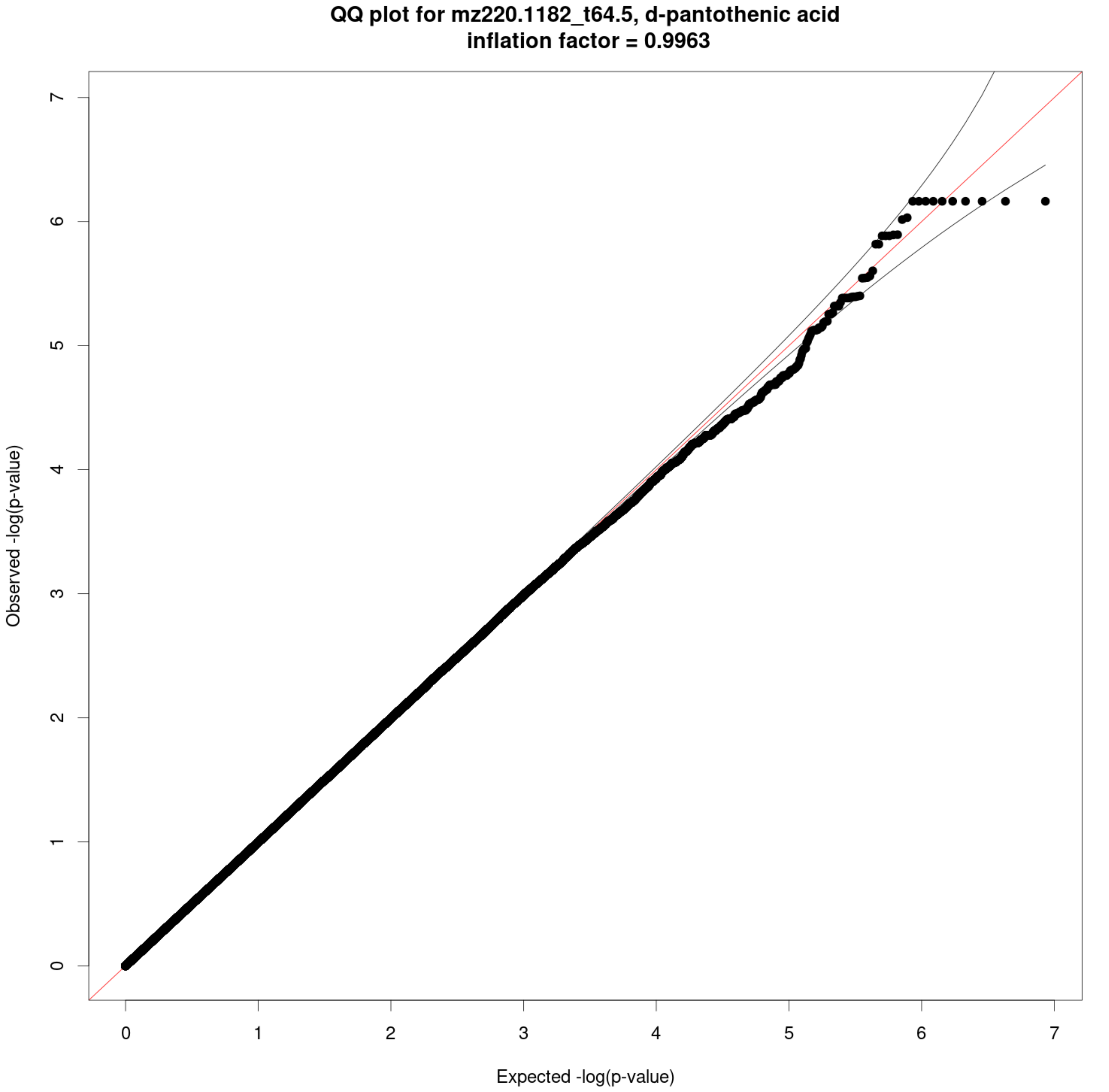
QQ plot for mz220.1182_t64.5, d-pantothenic acid
inflation factor = 0.9963
Observed -log(p-value)
Expected -log(p-value)
0
1
2
3
4
5
6
7

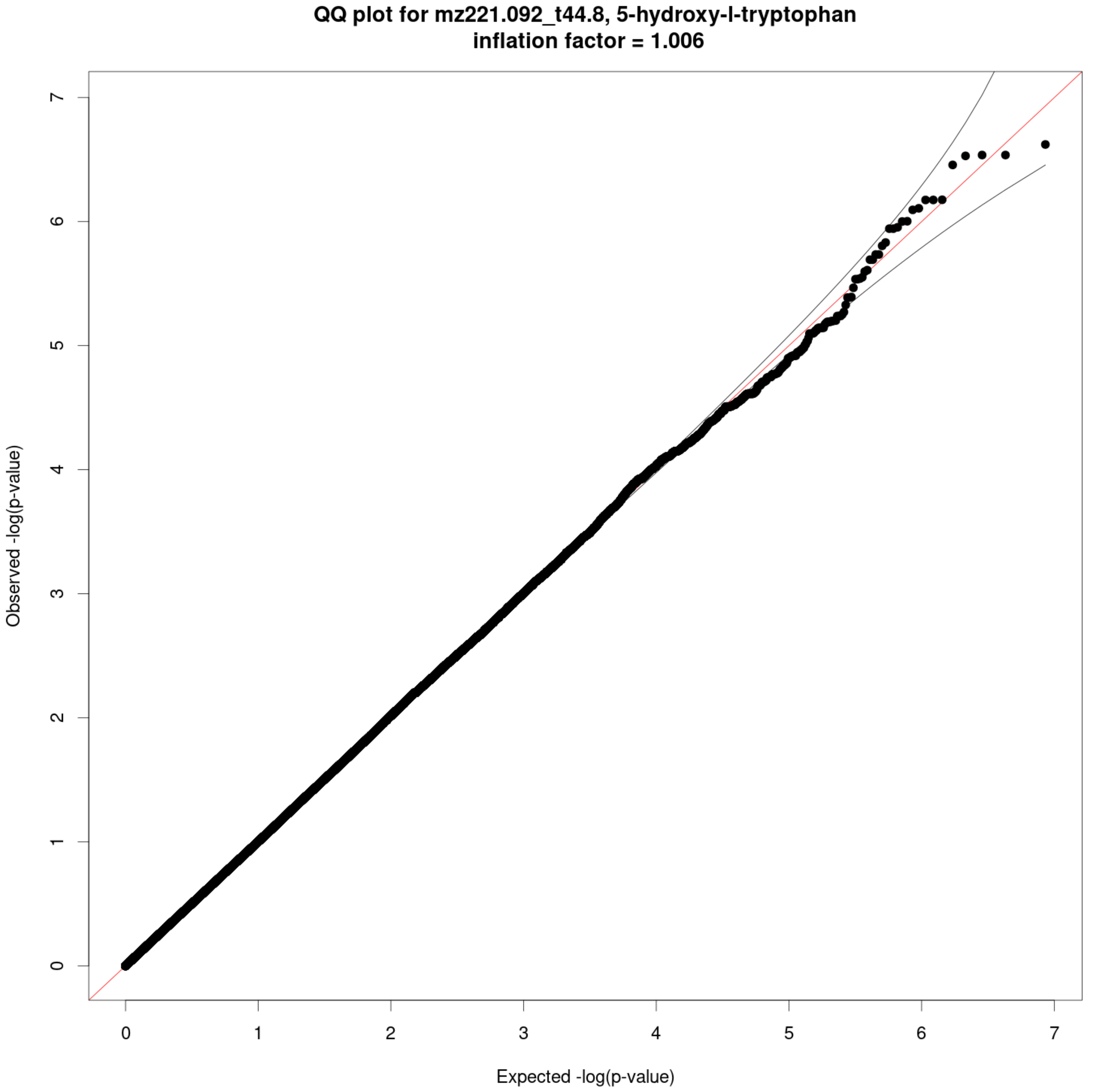
QQ plot for mz221.092_t44.8, 5-hydroxy-l-tryptophan
inflation factor = 1.006
Observed -log(p-value)
Expected -log(p-value)
0
1
2
3
4
5
6
7

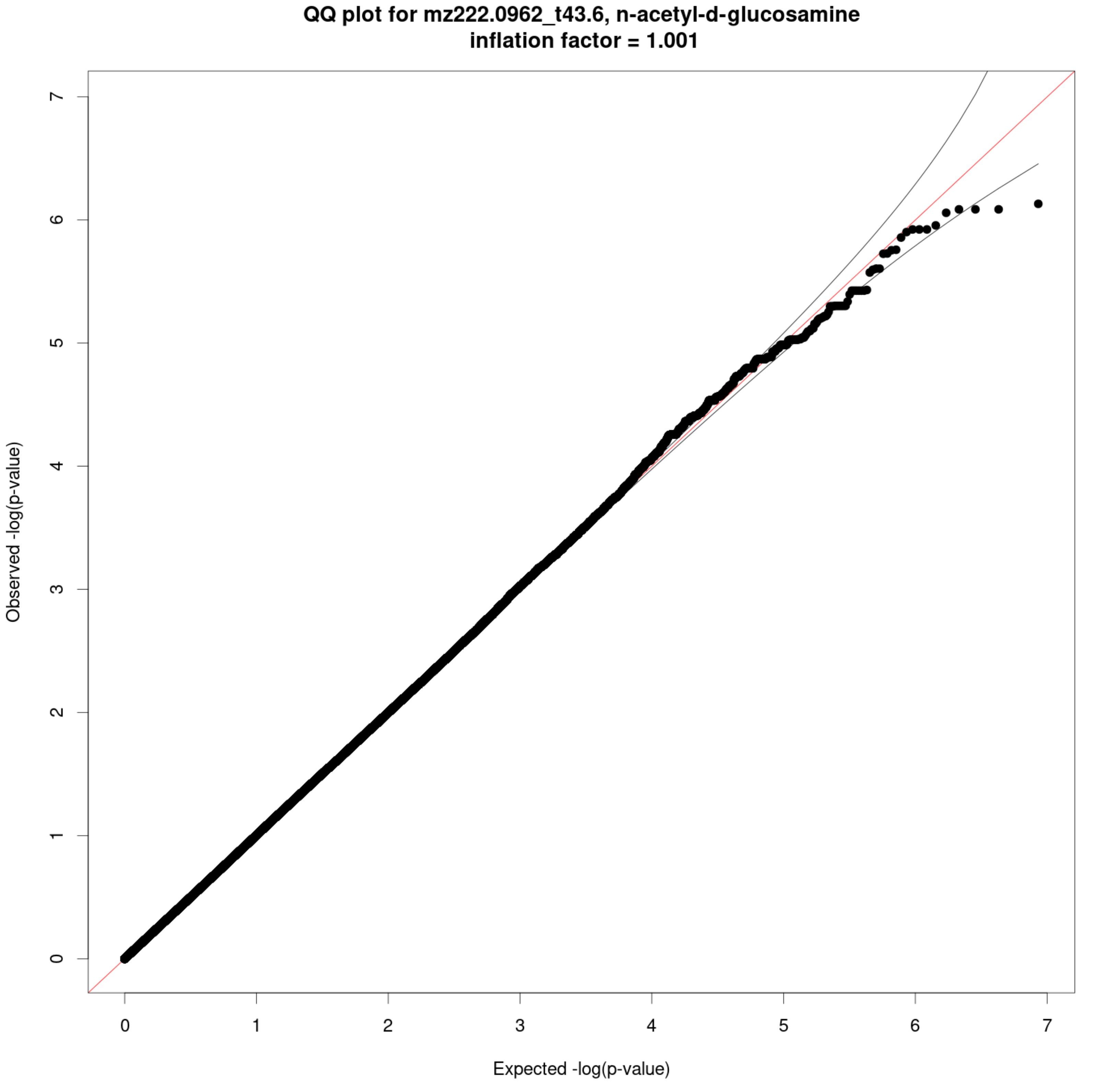
QQ plot for mz222.0962_t43.6, n-acetyl-d-glucosamine
inflation factor = 1.001
Observed -log(p-value)
Expected -log(p-value)
0
1
2
3
4
5
6
7

QQ plot for mz225.1484_t26.7, methyl jasmonate
inflation factor = 1.002

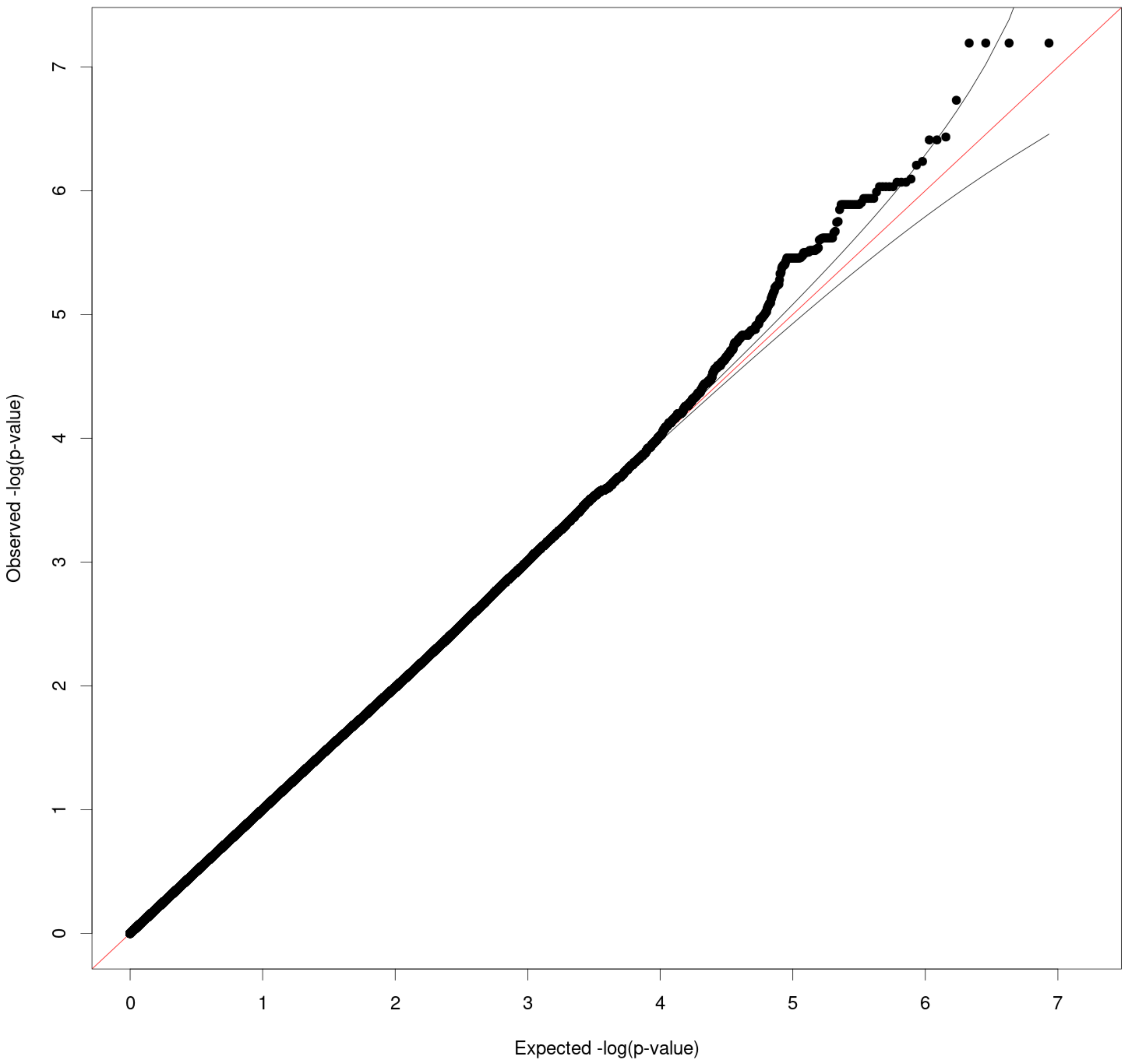

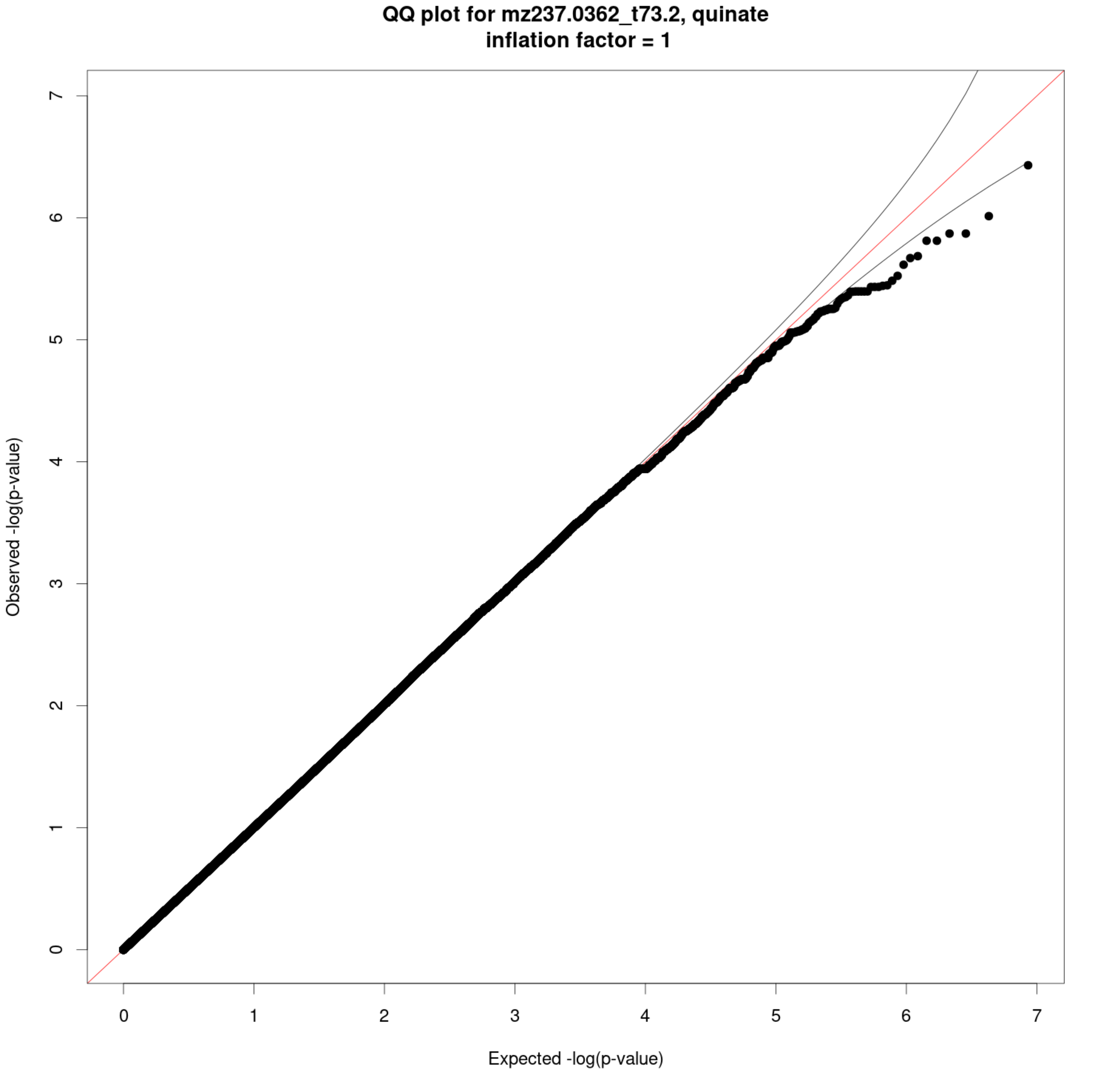
QQ plot for mz237.0362_t73.2, quinate
inflation factor = 1
Observed -log(p-value)
Expected -log(p-value)
0
1
2
3
4
5
6
7

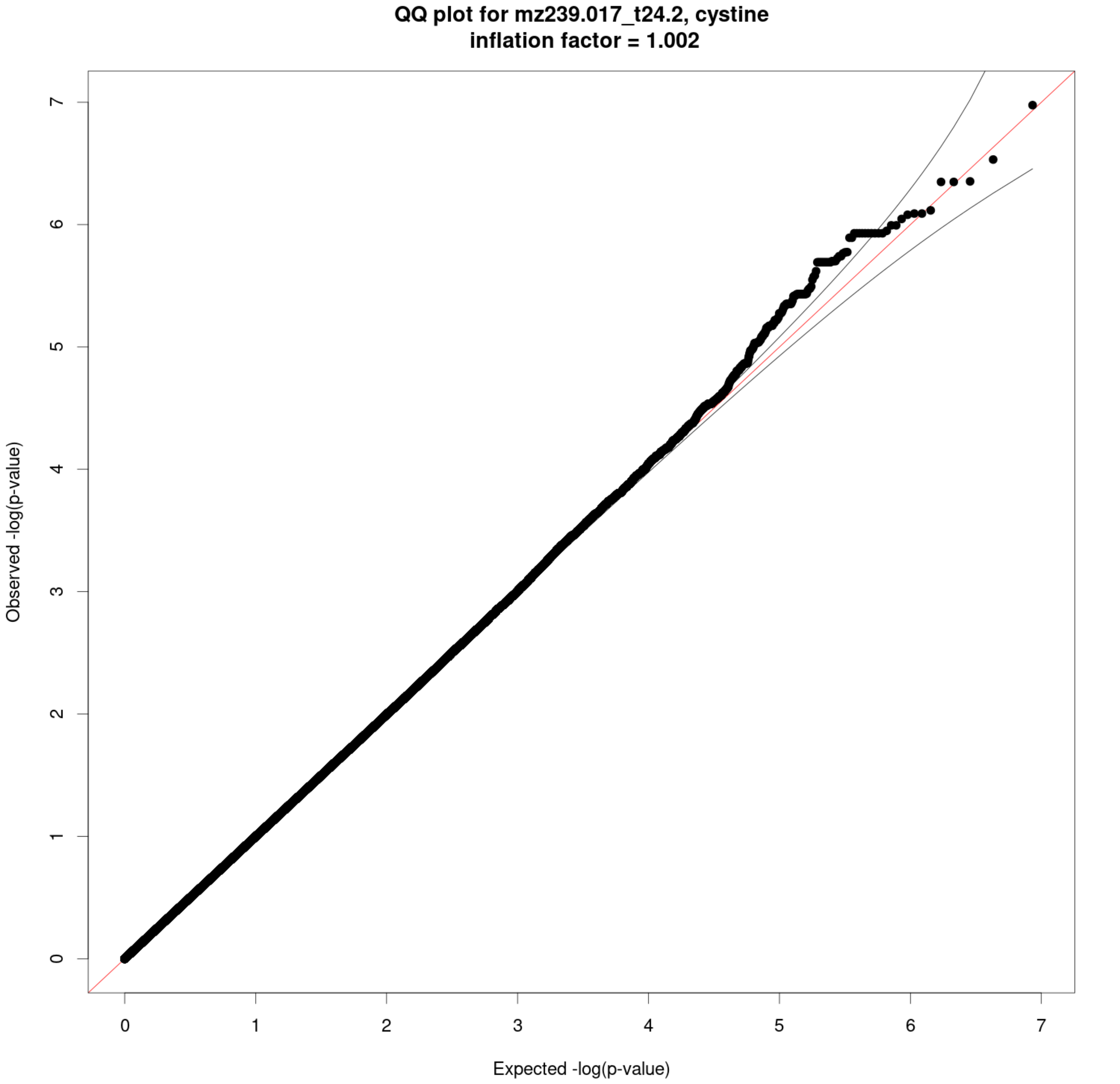
QQ plot for mz239.017_t24.2, cystine
inflation factor = 1.002
Observed -log(p-value)
Expected -log(p-value)
0
1
2
3
4
5
6
7
0
1
2
3
4
5
6
7

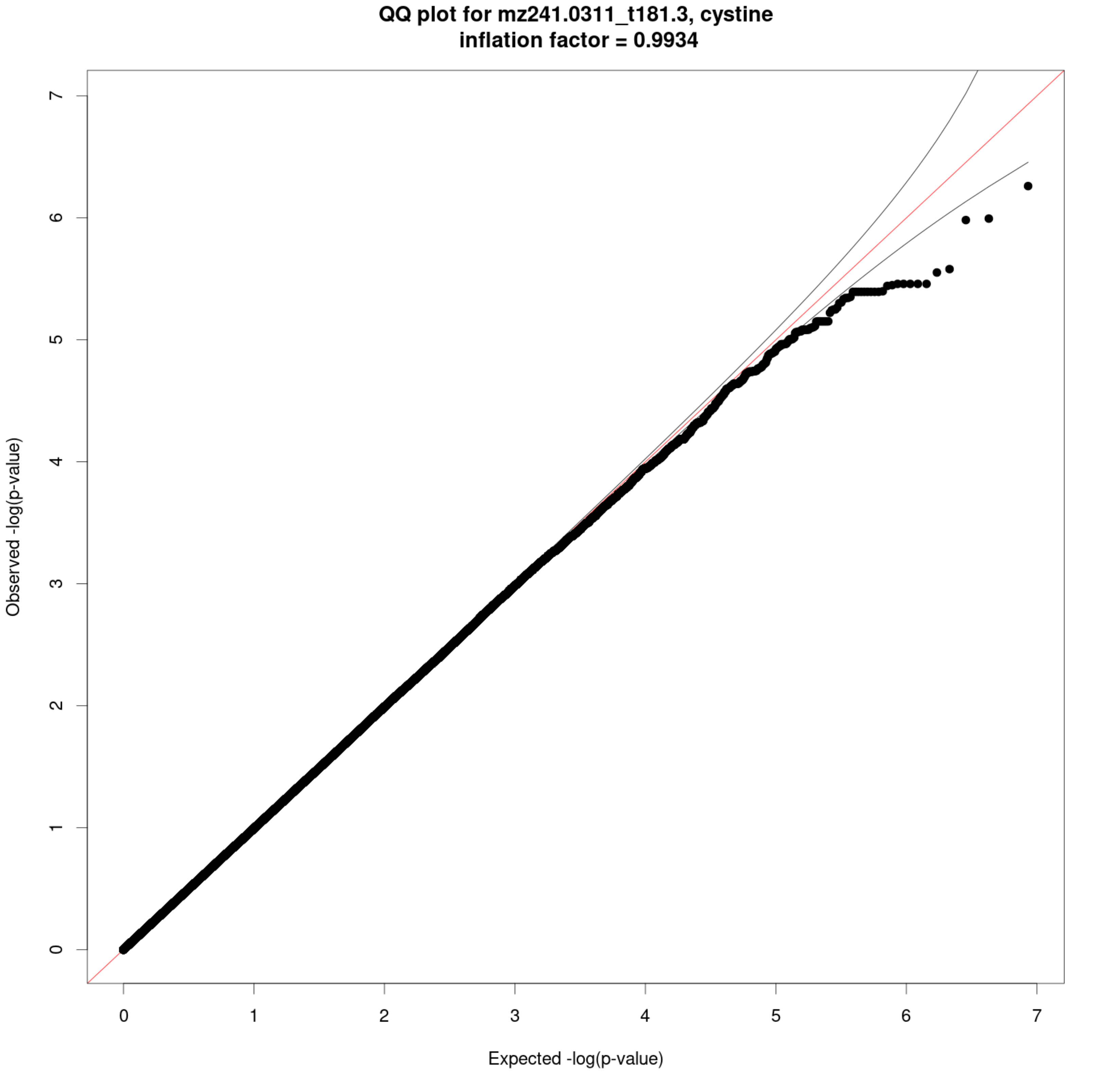
QQ plot for mz241.0311_t181.3, cystine
inflation factor = 0.9934
Observed -log(p-value)
Expected -log(p-value)
0
1
2
3
4
5
6
7

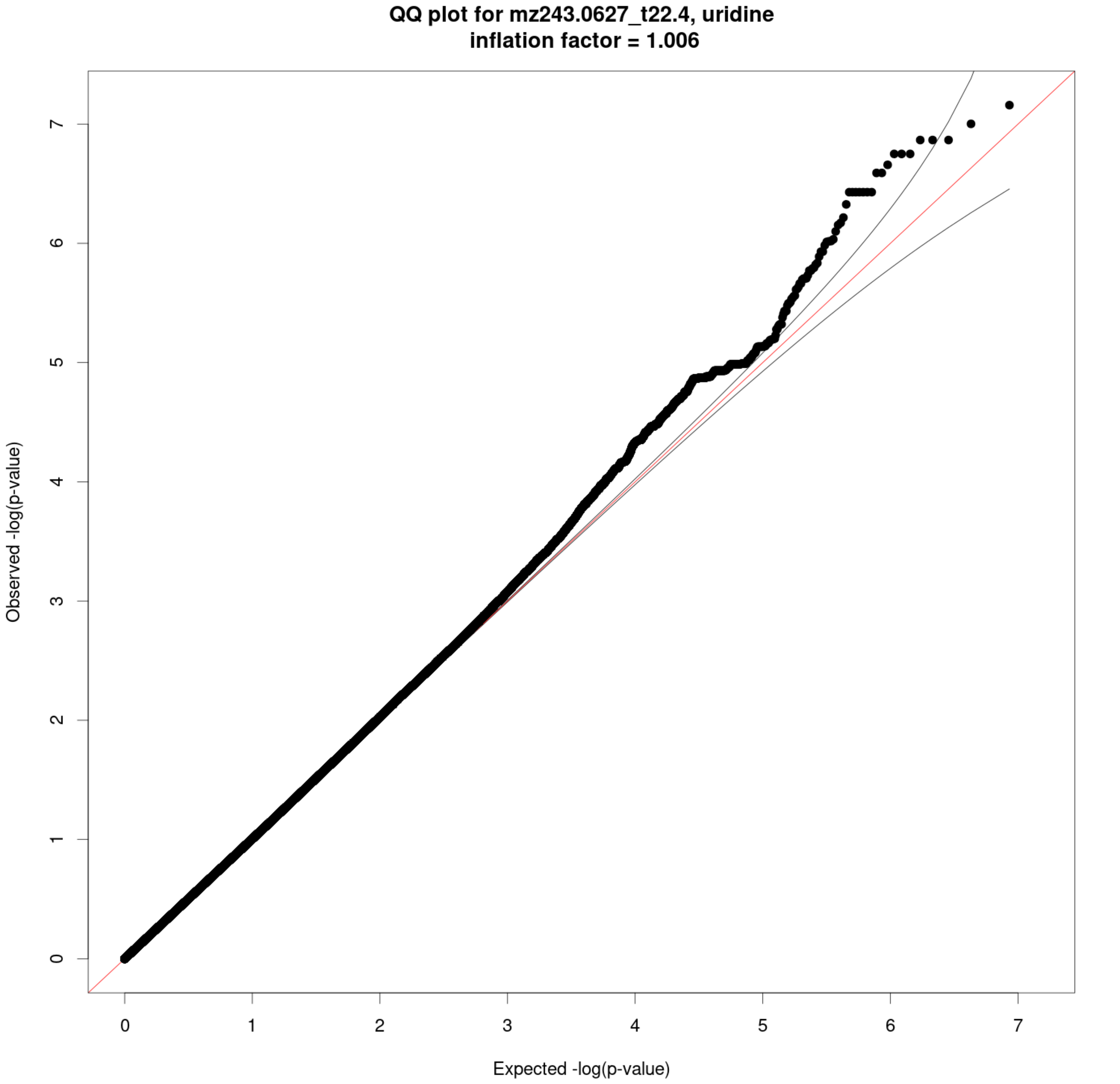
QQ plot for mz243.0627_t22.4, uridine
inflation factor = 1.006
Observed -log(p-value)
Expected -log(p-value)
0
1
2
3
4
5
6
7

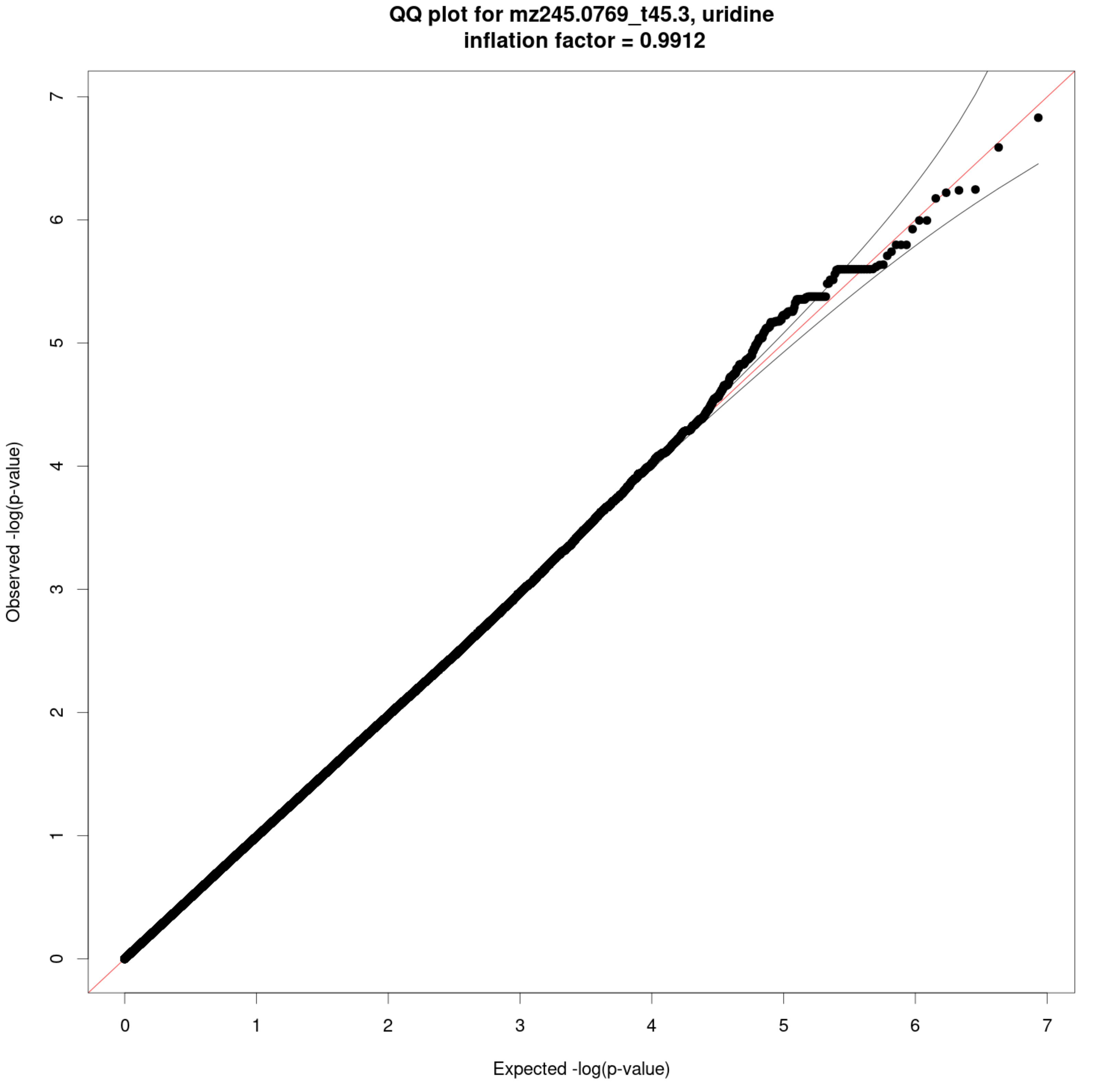
QQ plot for mz245.0769_t45.3, uridine
inflation factor = 0.9912
Observed -log(p-value)
Expected -log(p-value)
0
1
2
3
4
5
6
7

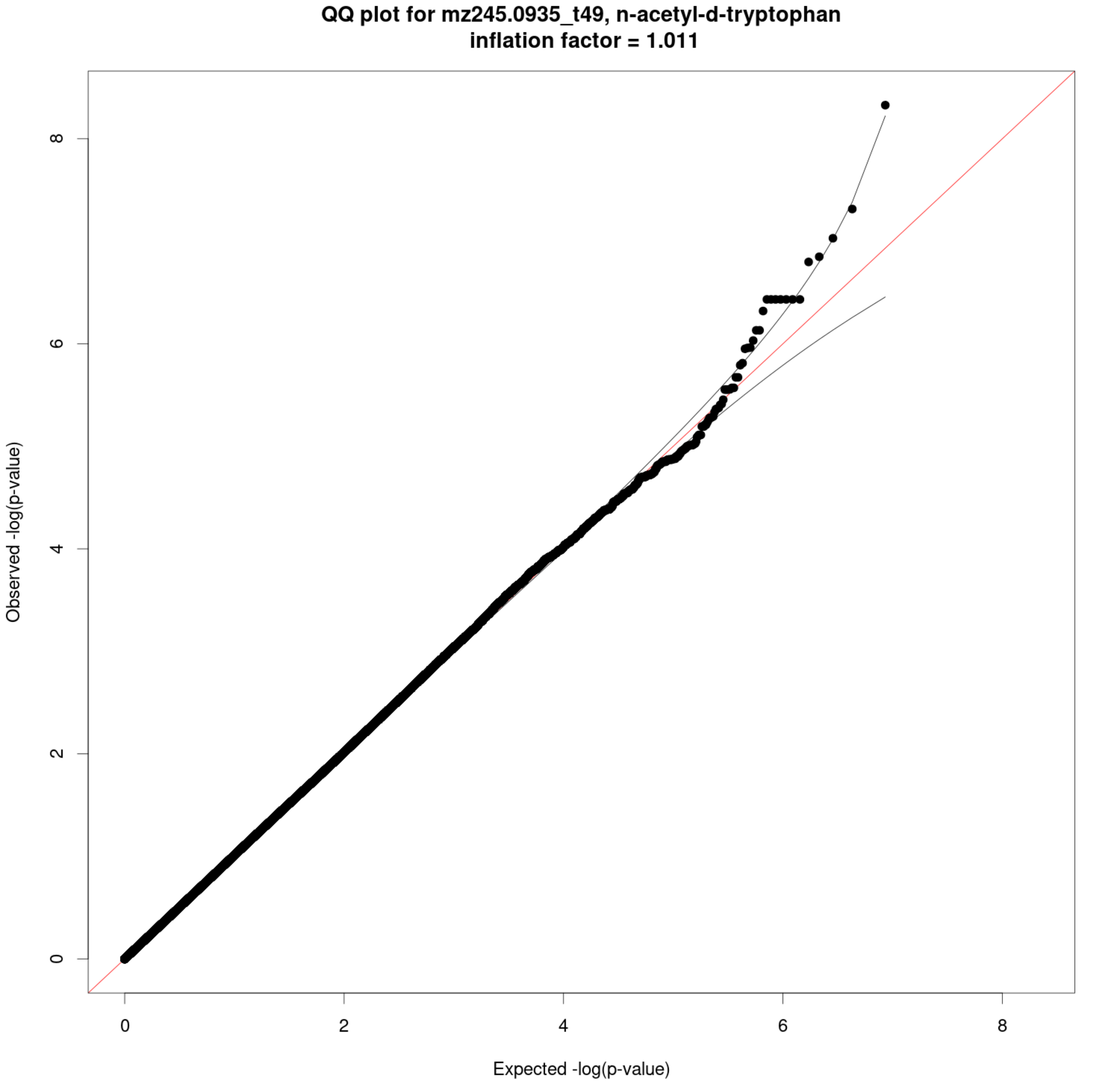
QQ plot for mz245.0935_t49, n-acetyl-d-tryptophan
inflation factor = 1.011
Observed -log(p-value)
Expected -log(p-value)
0
2
4
6
8

QQ plot for mz245.0971_t55, biotin
inflation factor = 1.008

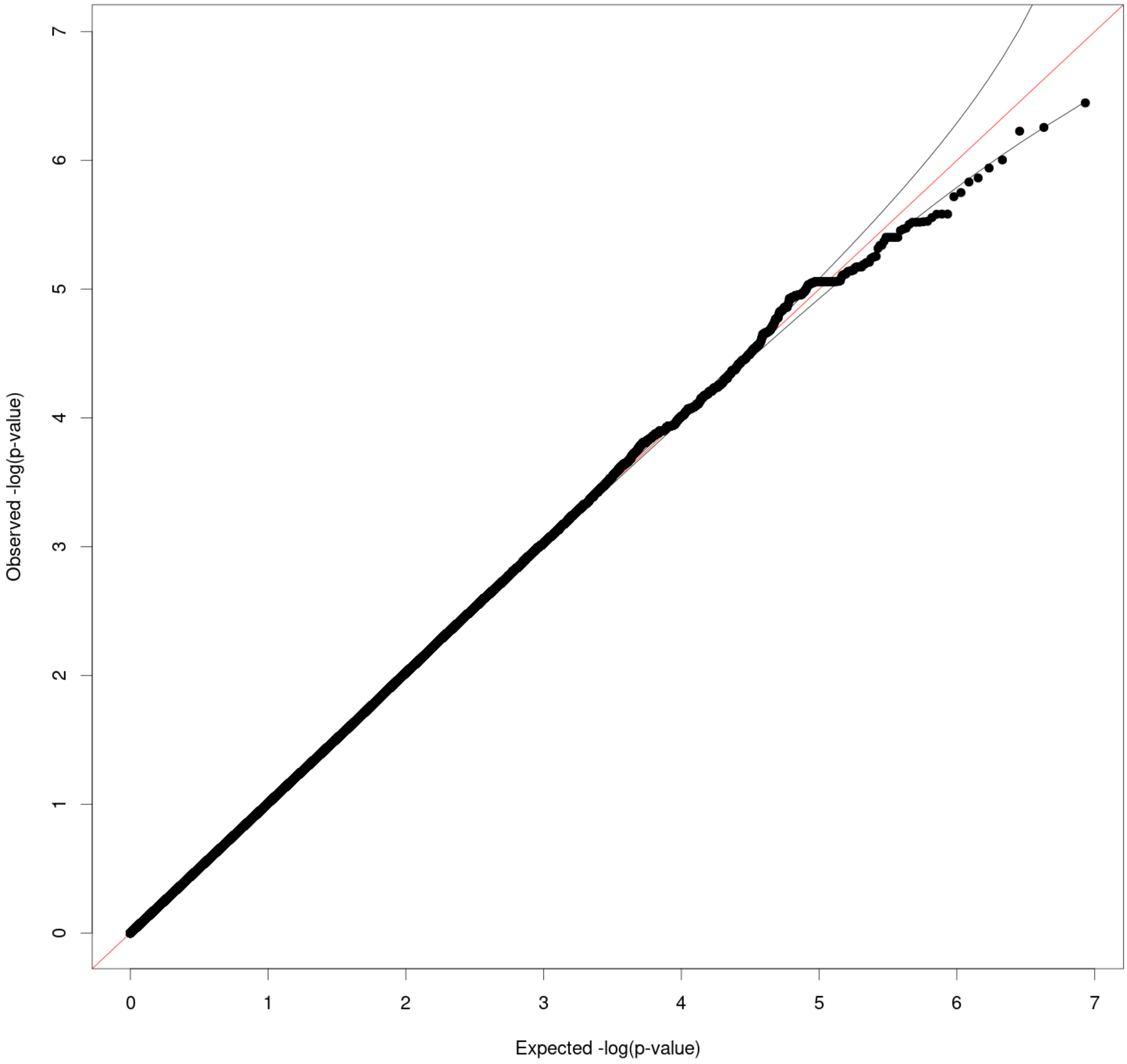

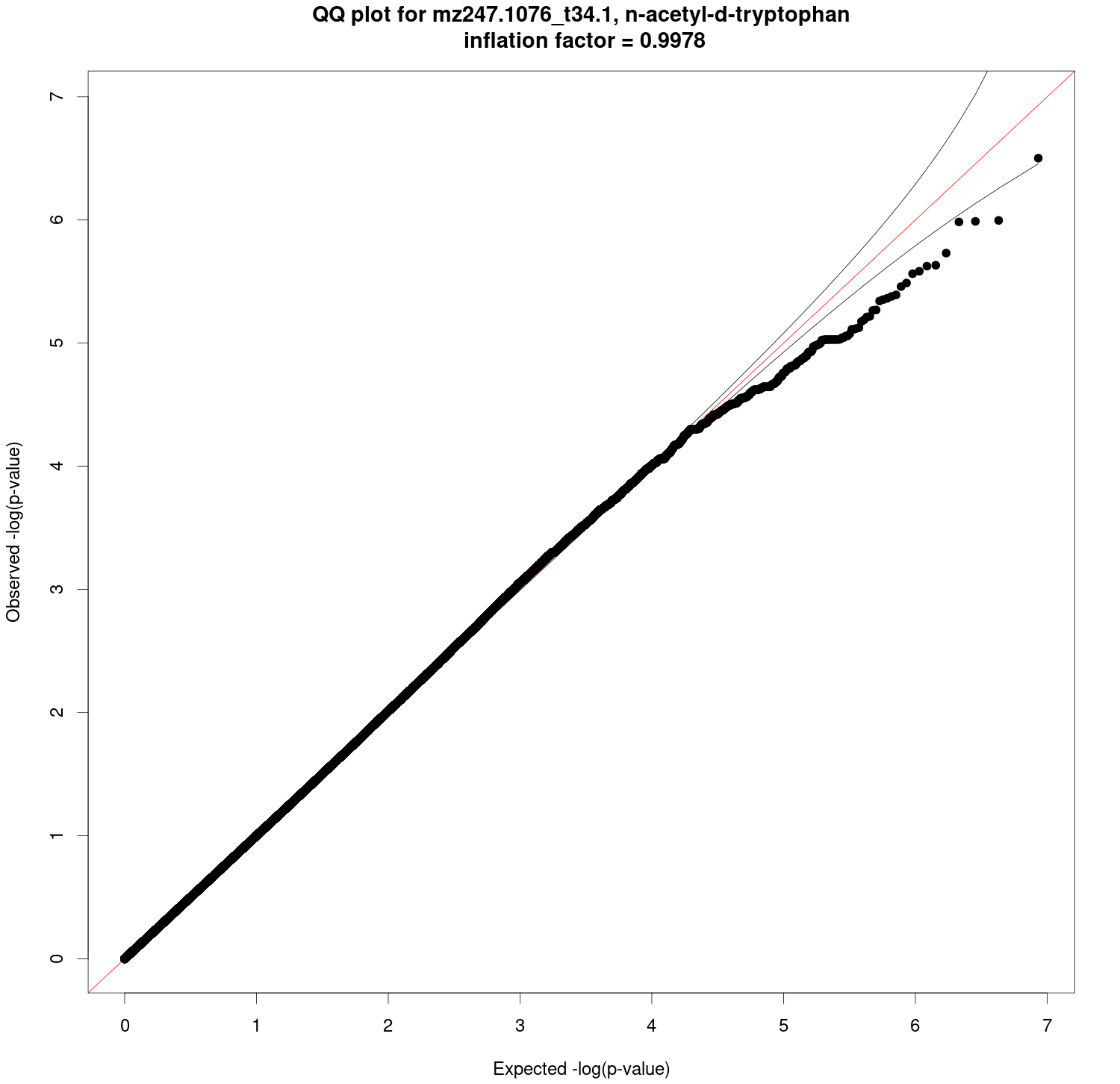
QQ plot for mz247.1076_t34.1, n-acetyl-d-tryptophan
inflation factor = 0.9978
Observed -log(p-value)
Expected -log(p-value)
0
1
2
3
4
5
6
7

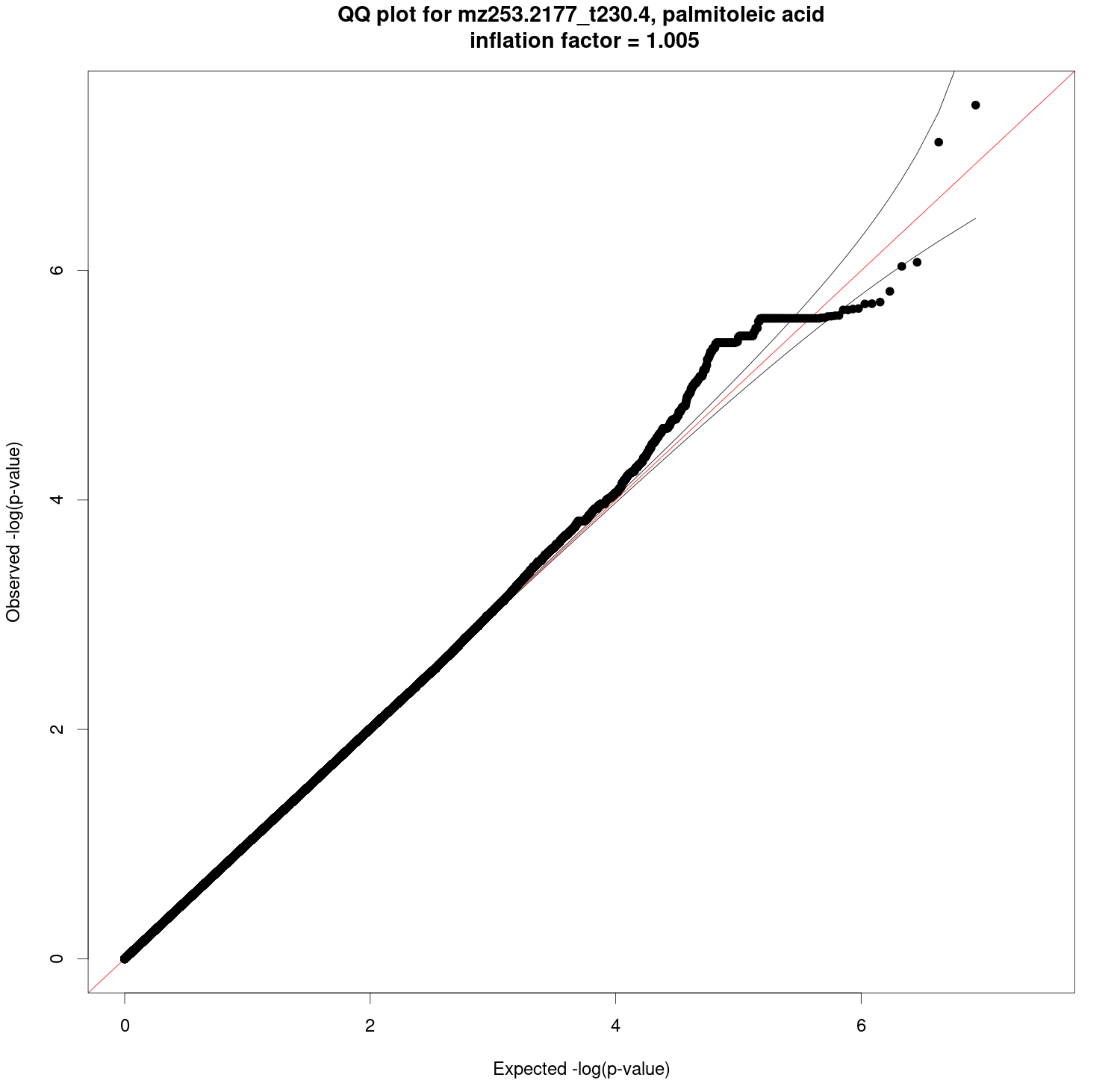
QQ plot for mz253.2177_t230.4, palmitoleic acid
inflation factor = 1.005
Observed -log(p-value)
Expected -log(p-value)
0
2
4
6
0
2
4
6

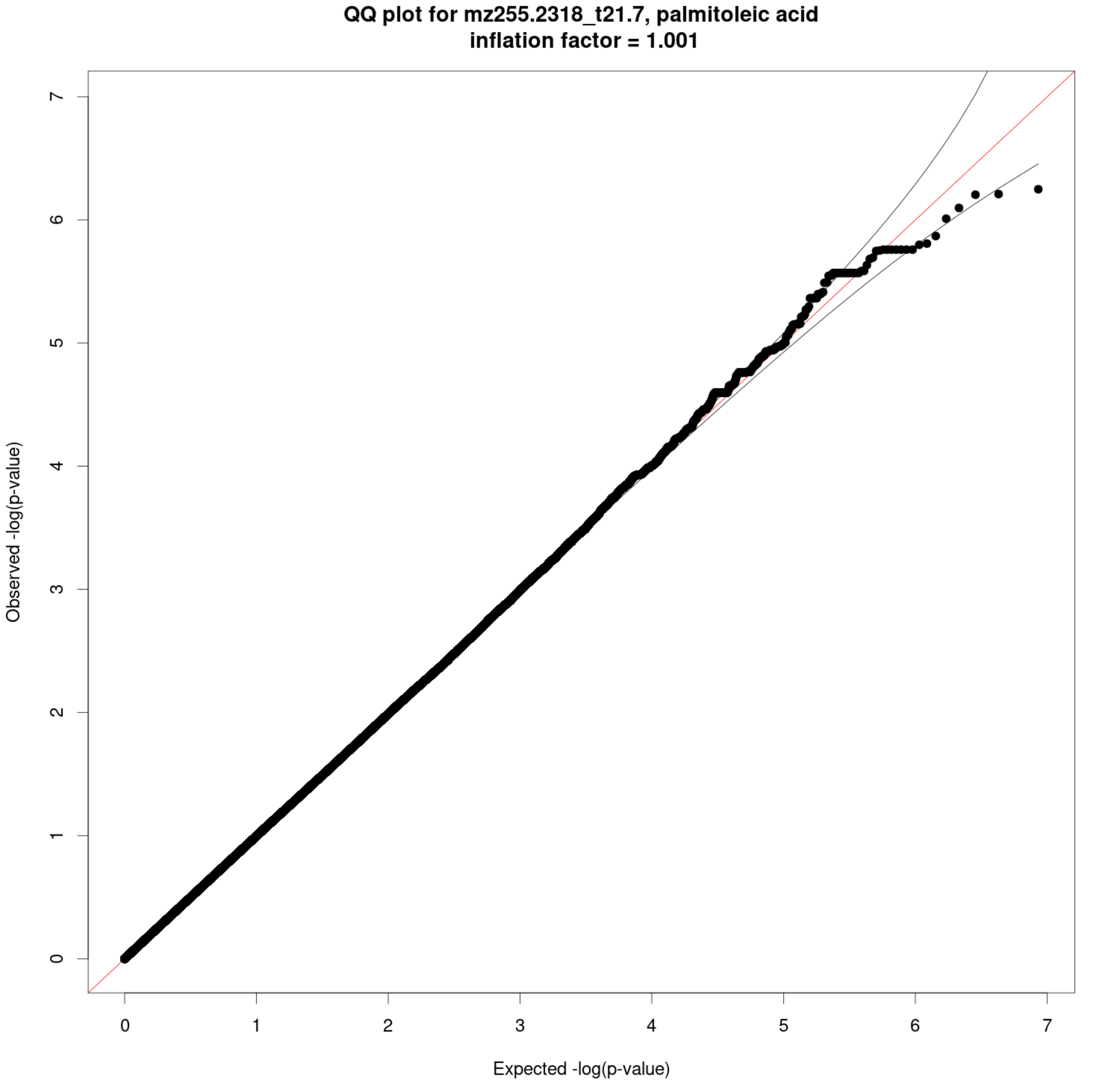
QQ plot for mz255.2318_t21.7, palmitoleic acid
inflation factor = 1.001
Observed -log(p-value)
Expected -log(p-value)
0
1
2
3
4
5
6
7

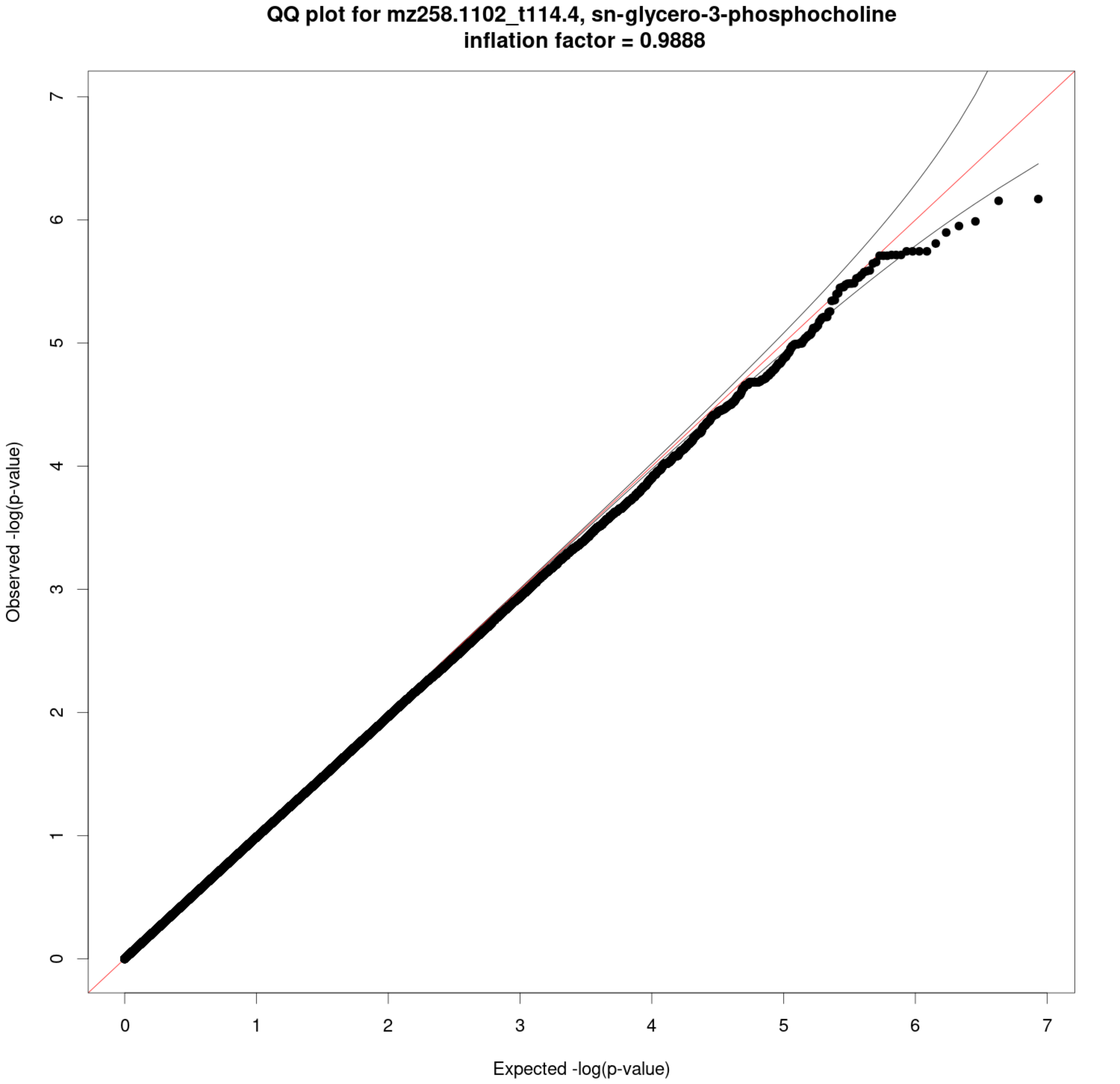
QQ plot for mz258.1102_t114.4, sn-glycero-3-phosphocholine
inflation factor = 0.9888
Observed -log(p-value)
Expected -log(p-value)
0
1
2
3
4
5
6
7

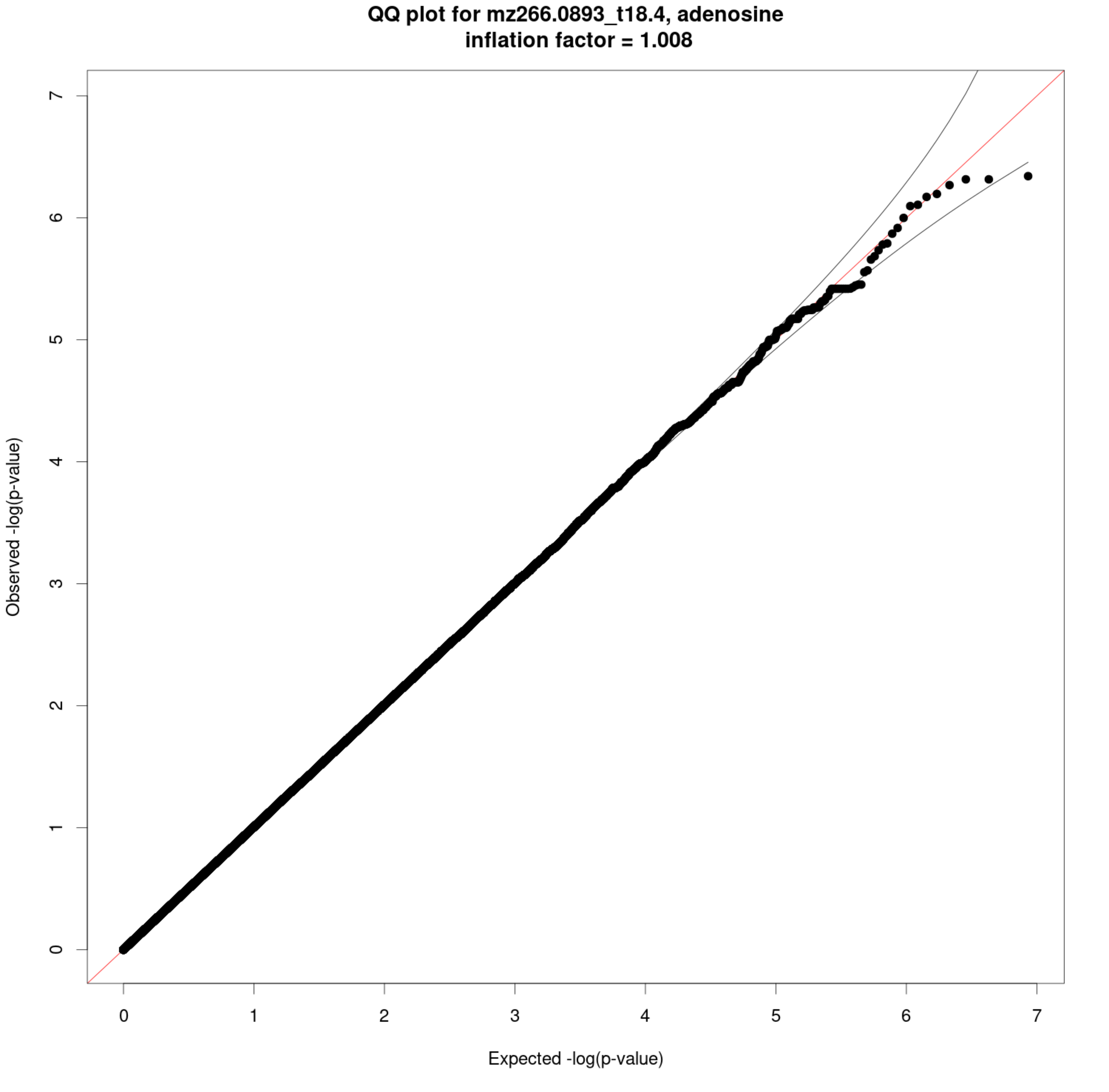
QQ plot for mz266.0893_t18.4, adenosine
inflation factor = 1.008
Observed -log(p-value)
Expected -log(p-value)
0
1
2
3
4
5
6
7

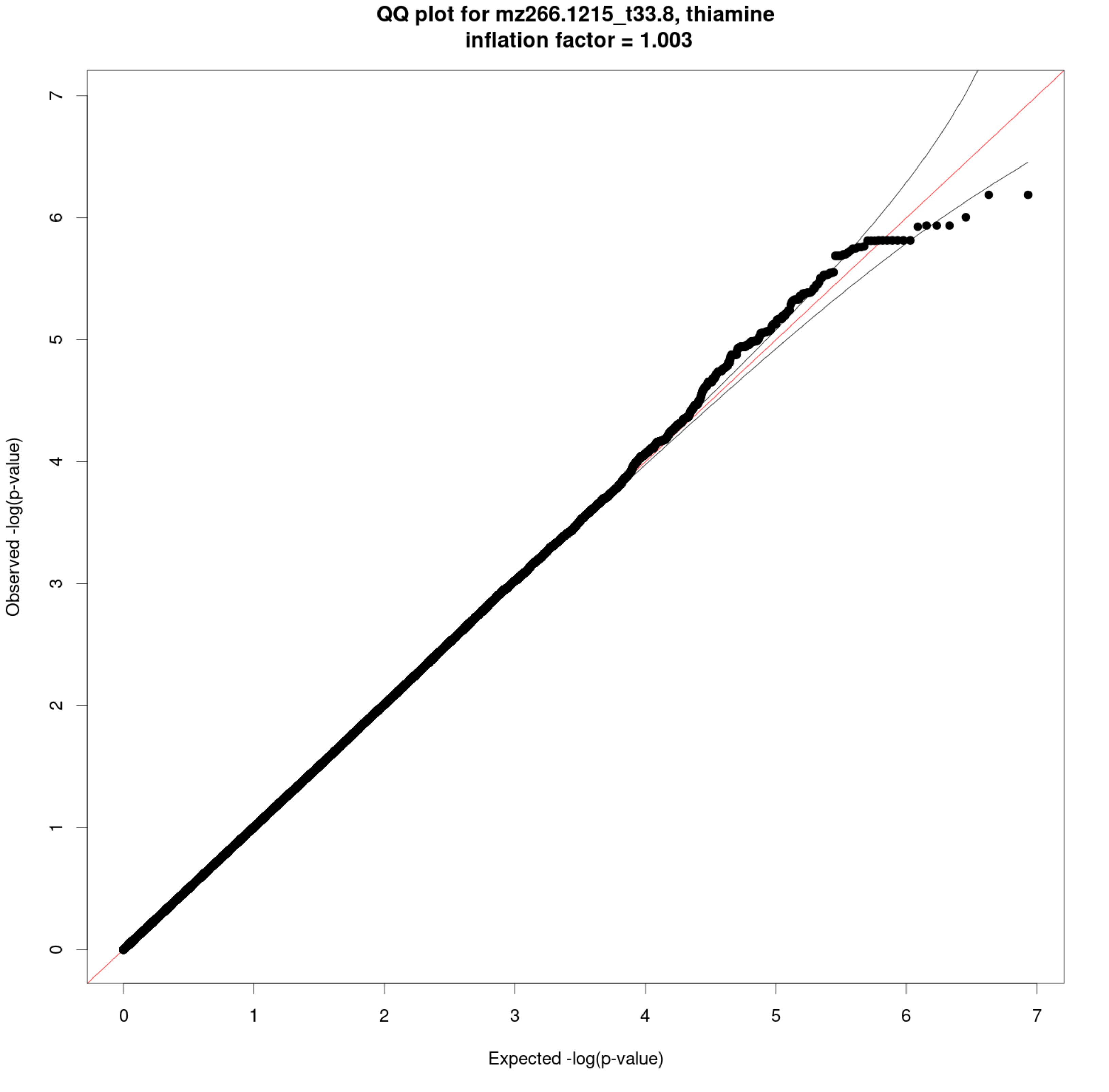

QQ plot for mz266.1215_t33.8, thiamine
inflation factor = 1.003
Observed -log(p-value)
Expected -log(p-value)
0
1
2
3
4
5
6
7

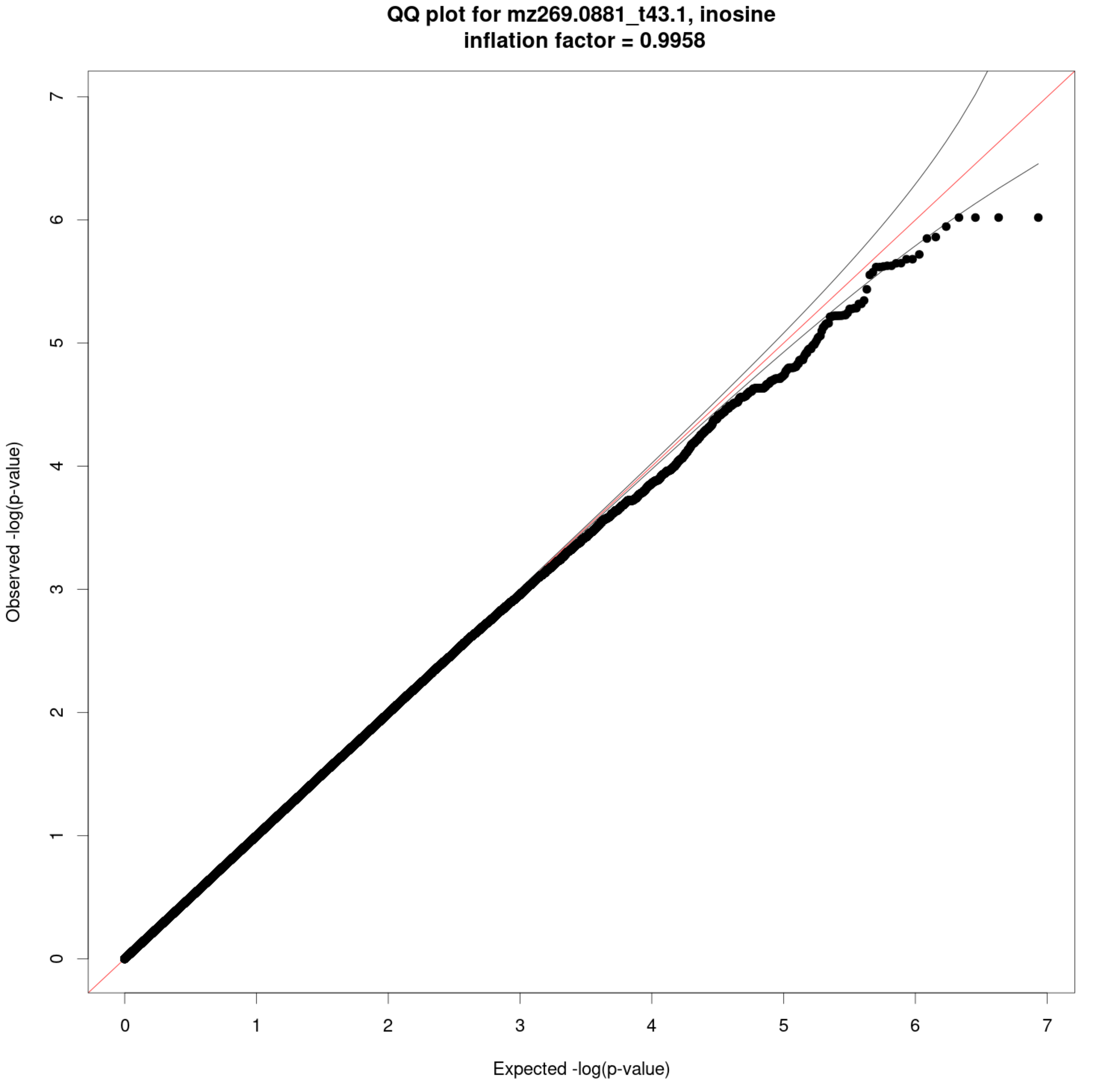
QQ plot for mz269.0881_t43.1, inosine
inflation factor = 0.9958
Observed -log(p-value)
Expected -log(p-value)
0
1
2
3
4
5
6
7

QQ plot for mz279.2319_t23.7, gamma-linolenic acid
inflation factor = 0.9958

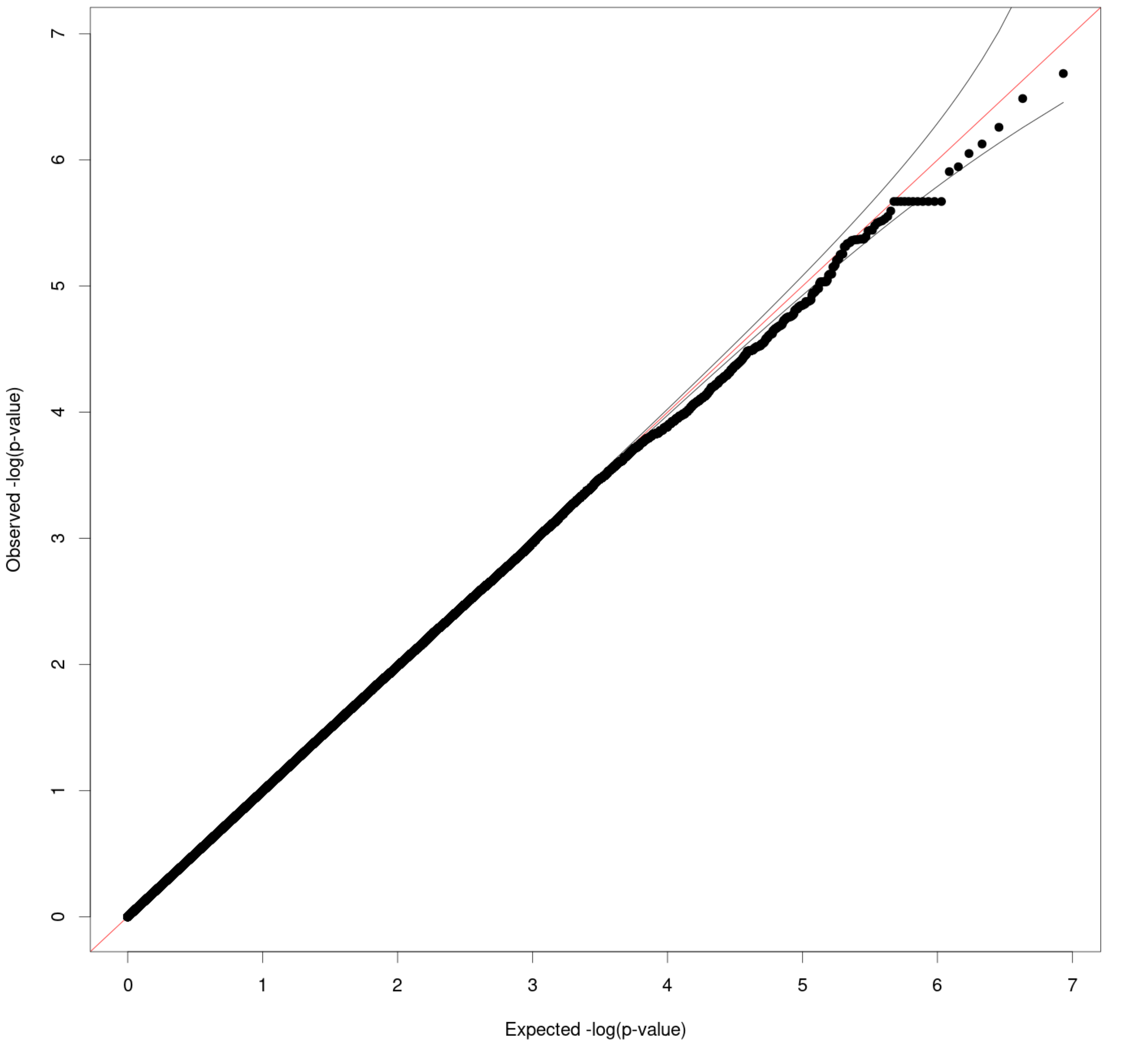

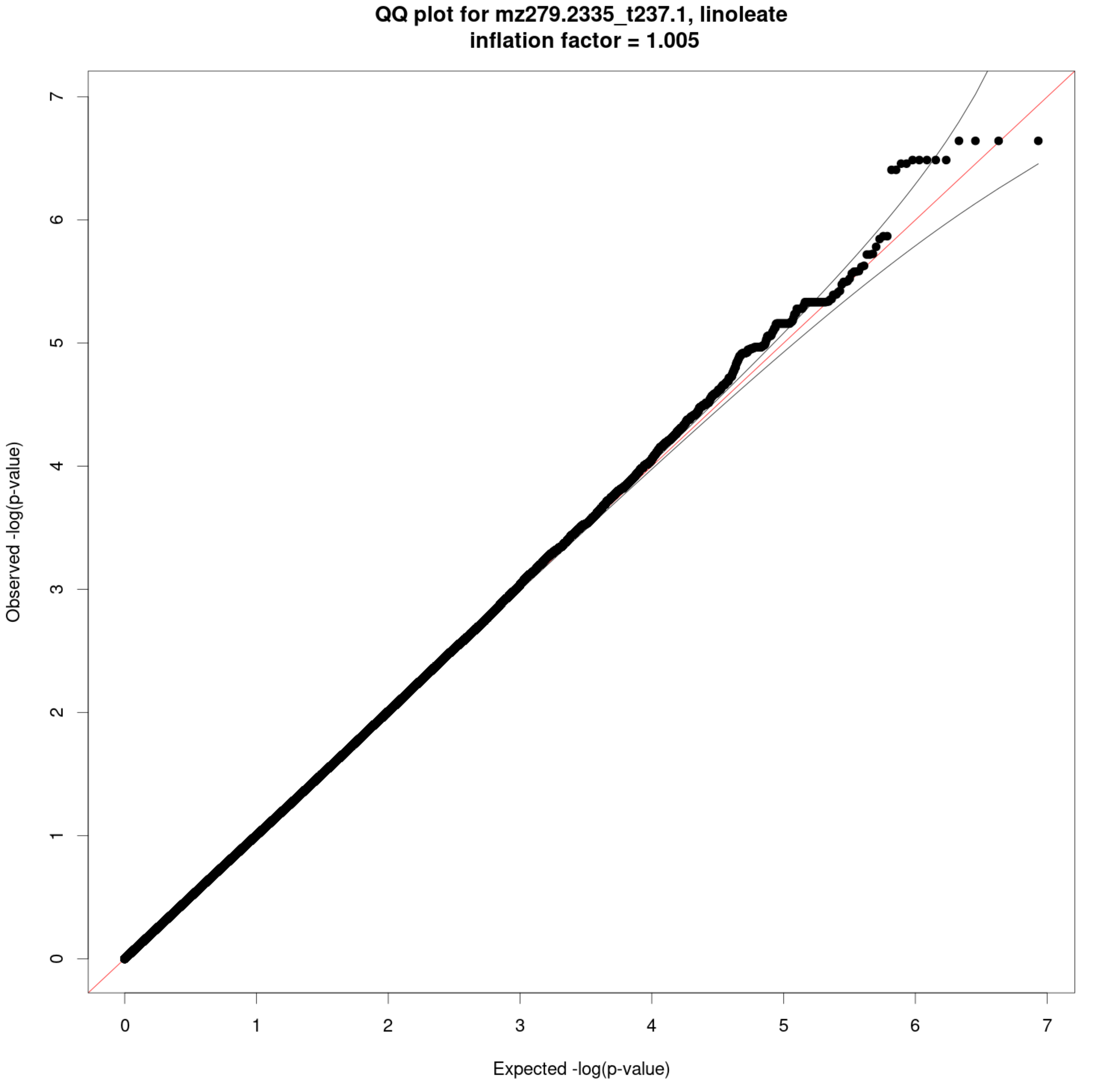
QQ plot for mz279.2335_t237.1, linoleate
inflation factor = 1.005
Observed -log(p-value)
Expected -log(p-value)
0
1
2
3
4
5
6
7

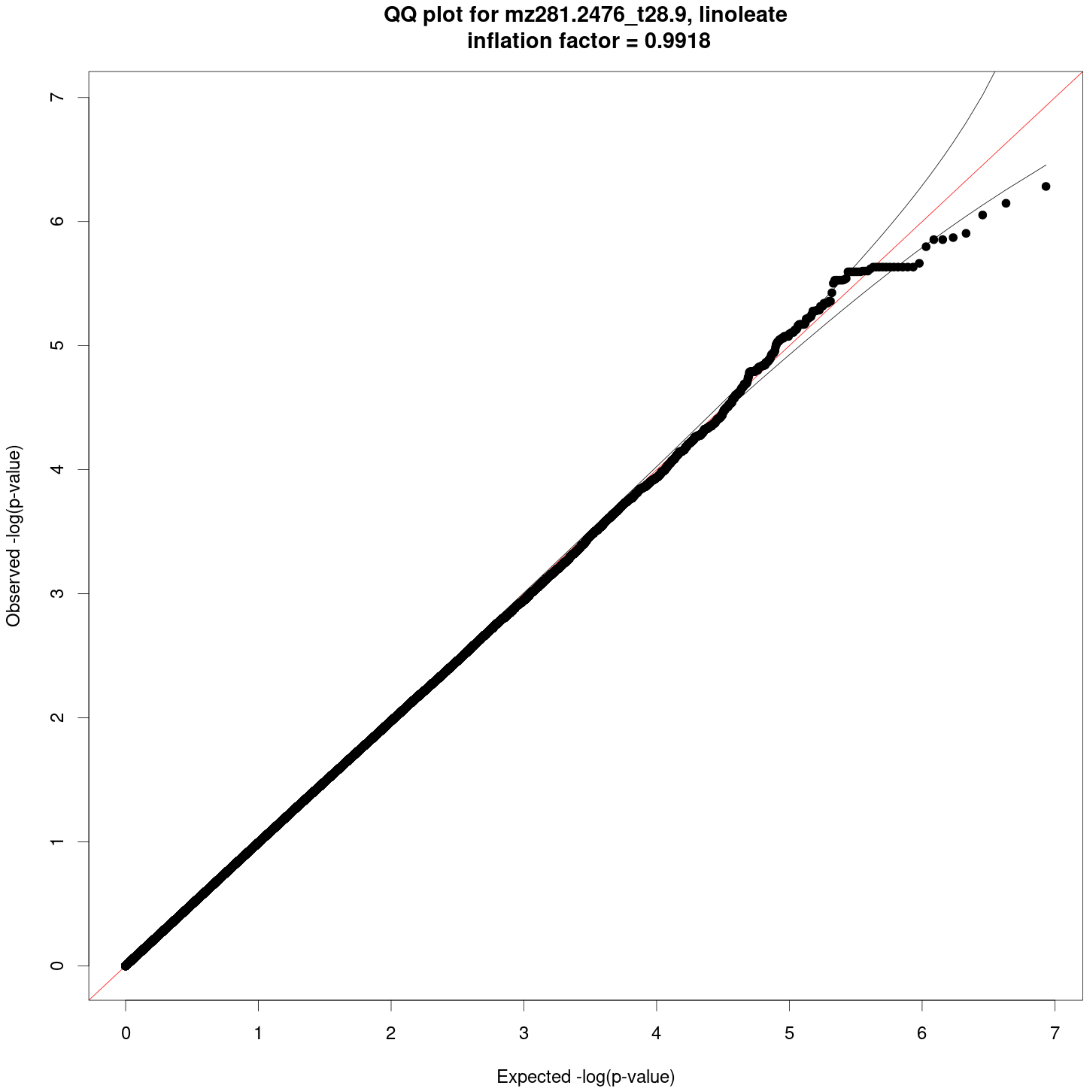
QQ plot for mz281.2476_t28.9, linoleate
inflation factor = 0.9918
Observed -log(p-value)
Expected -log(p-value)
0
1
2
3
4
5
6
7

QQ plot for mz281.2493_t256.8, elaidic acid
inflation factor = 1.007

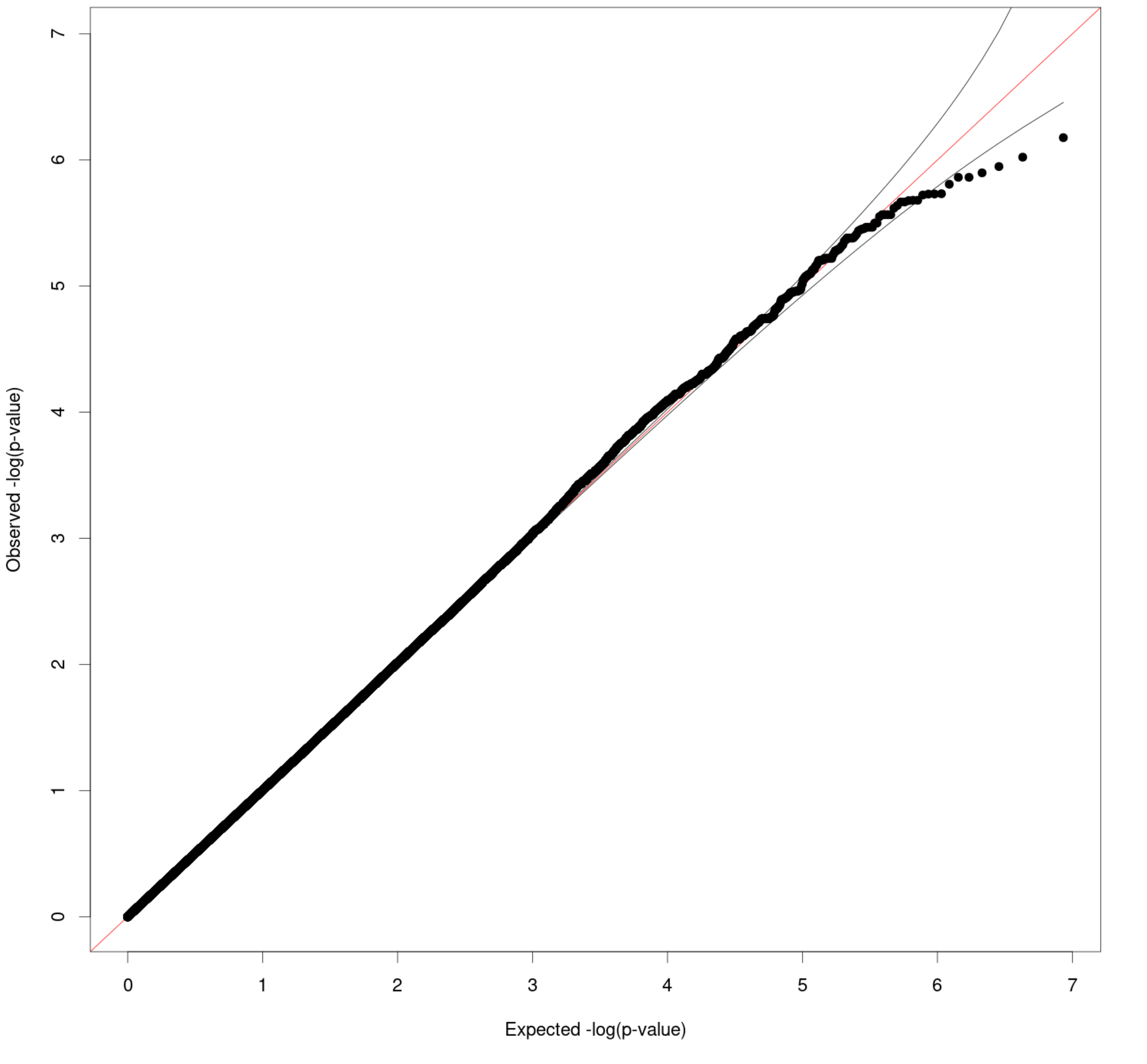

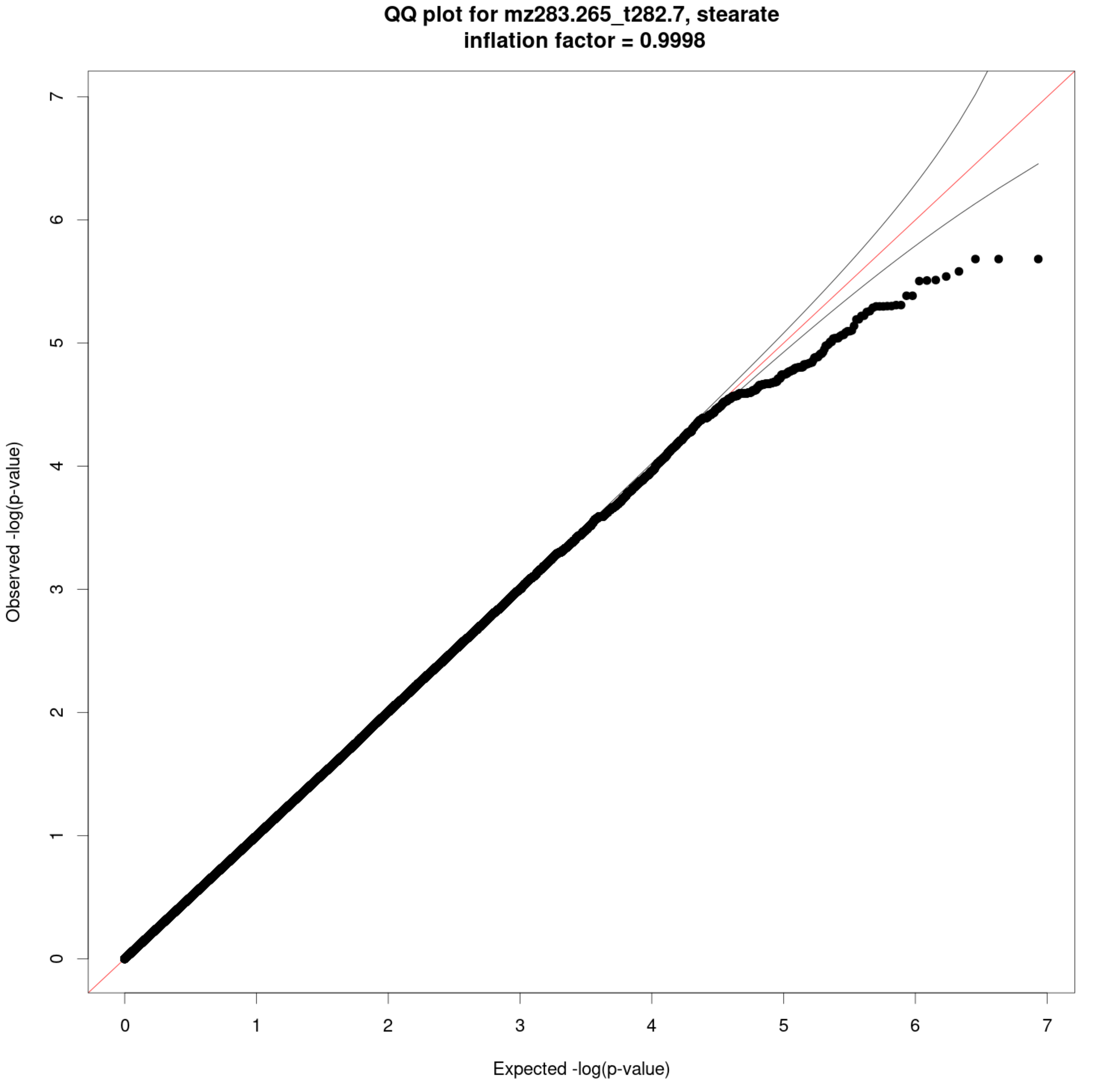
QQ plot for mz283.265_t282.7, stearate
inflation factor = 0.9998
Observed -log(p-value)
Expected -log(p-value)
0
1
2
3
4
5
6
7

QQ plot for mz301.239_t252, rac-glycerol 1-myristate
inflation factor = 1.008

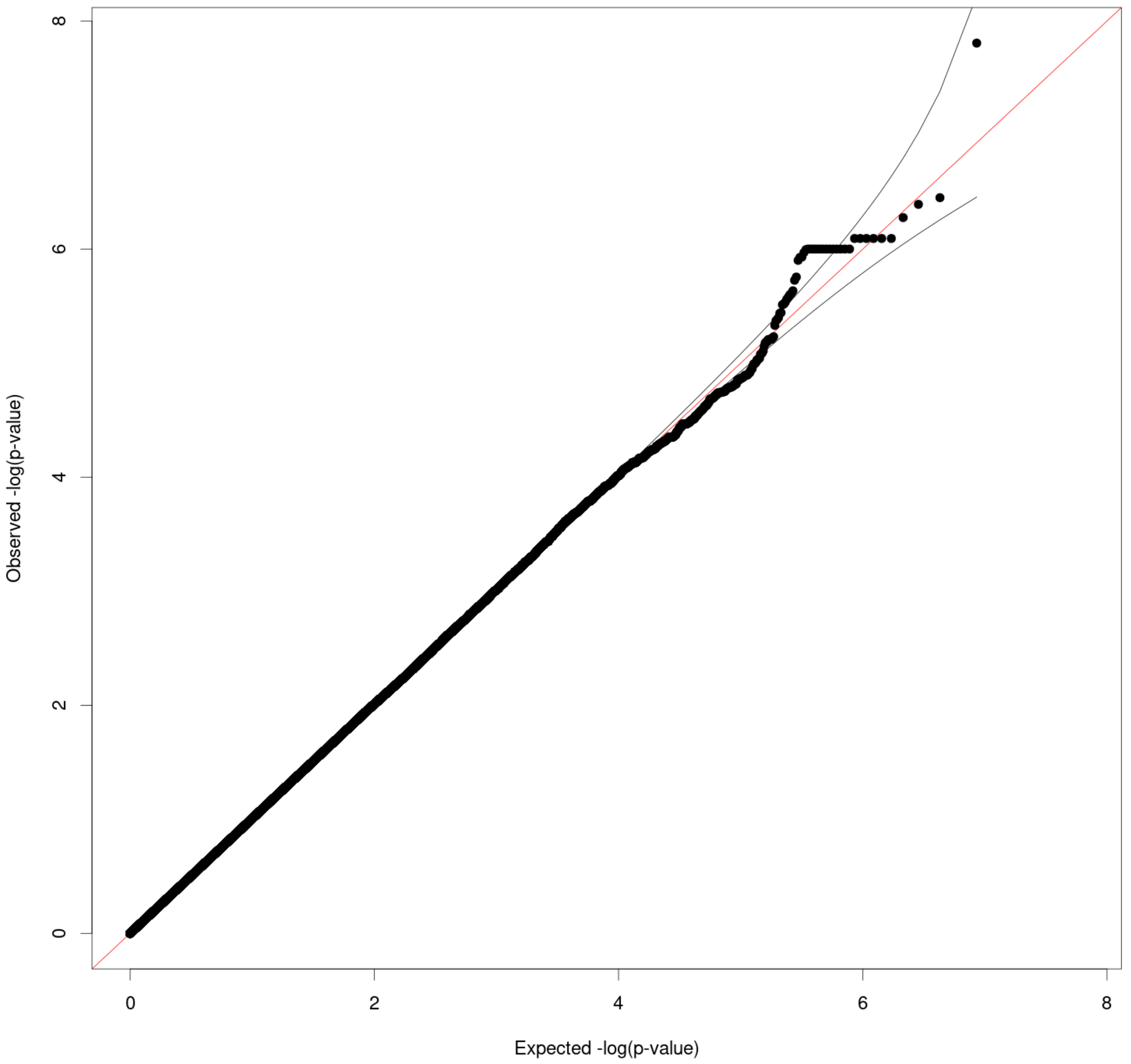

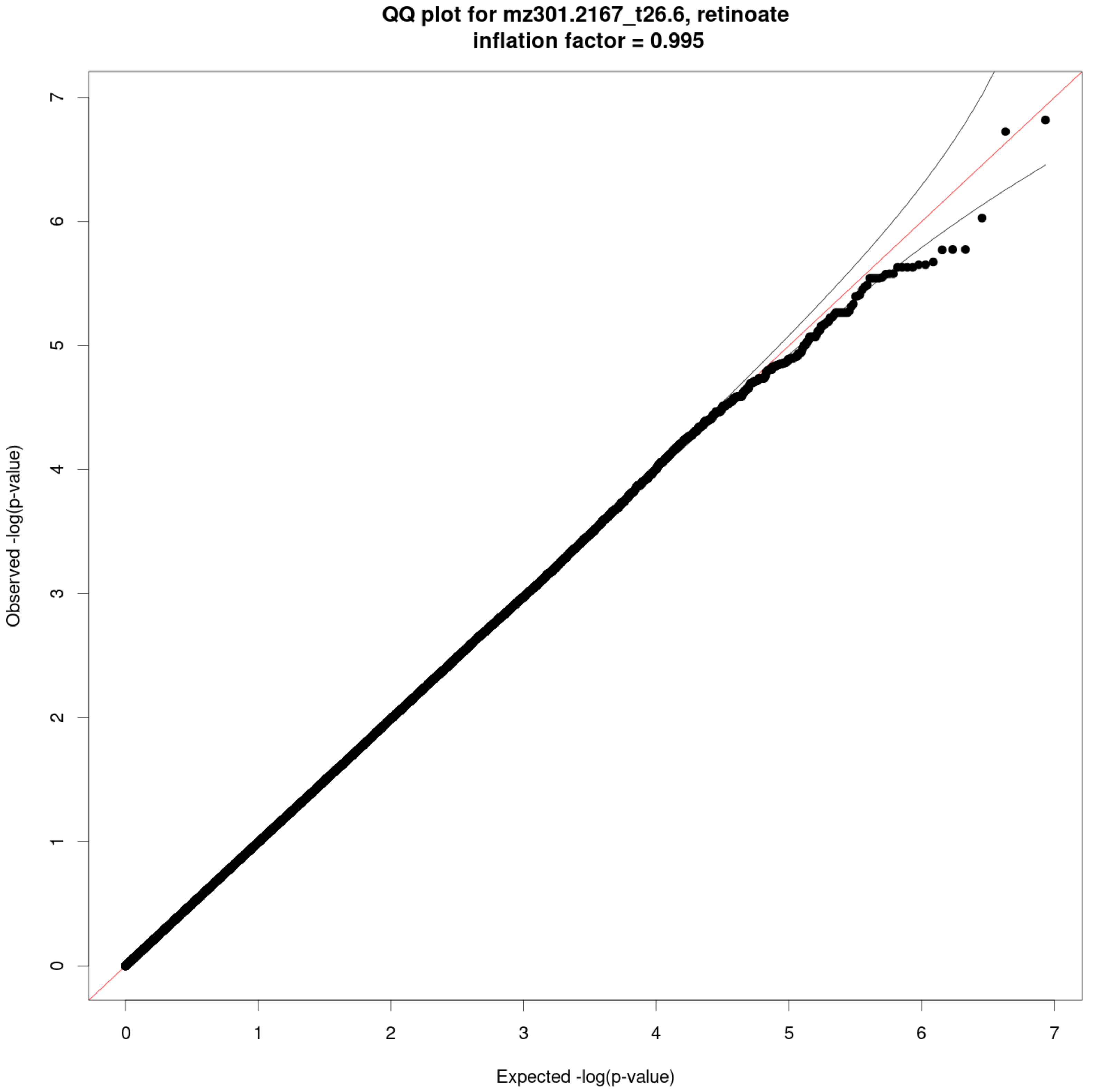
QQ plot for mz301.2167_t26.6, retinoate
inflation factor = 0.995
Observed -log(p-value)
Expected -log(p-value)
0
1
2
3
4
5
6
7

QQ plot for mz302.3054_t35.1, sphinganine
inflation factor = 1.002

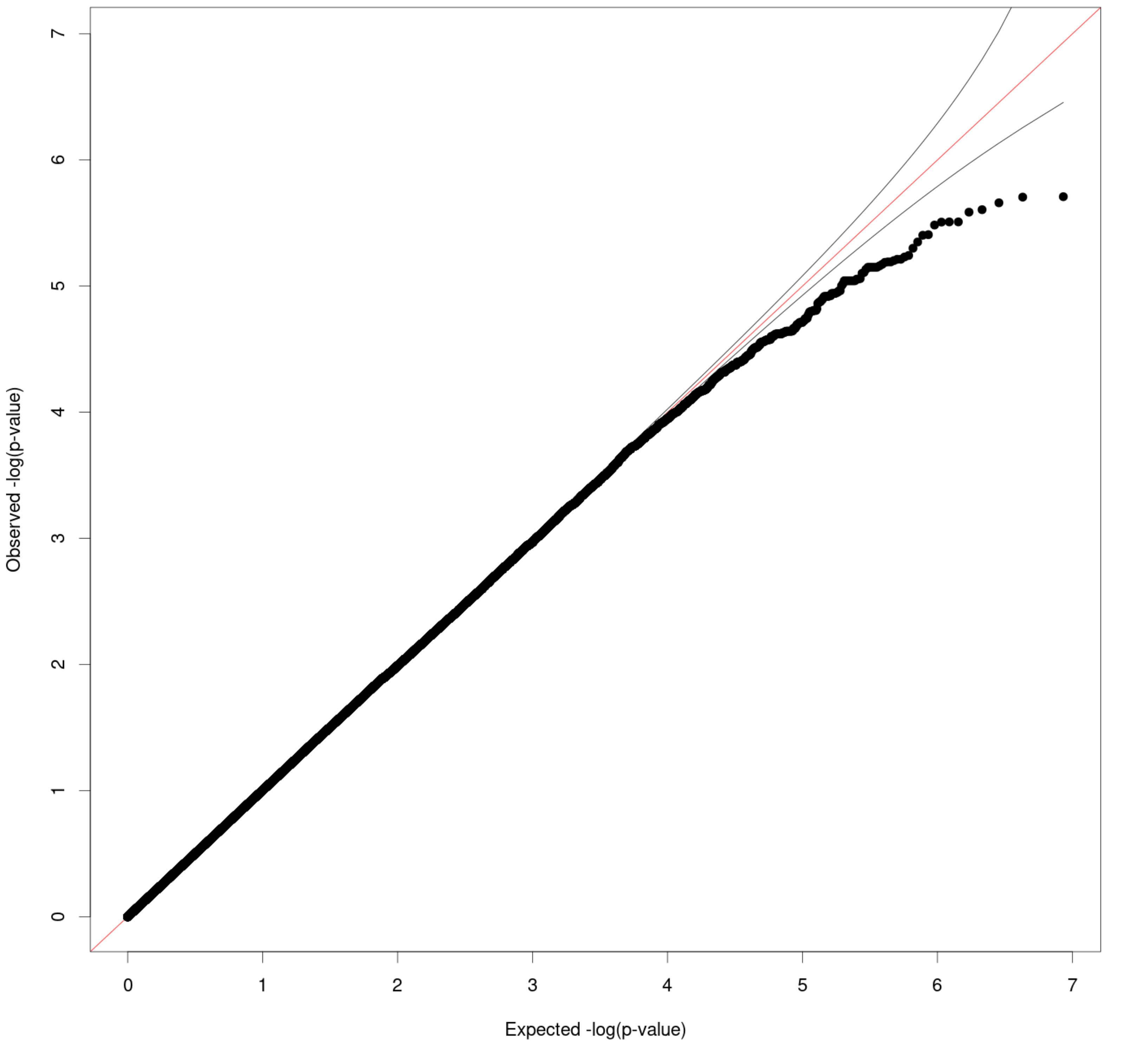

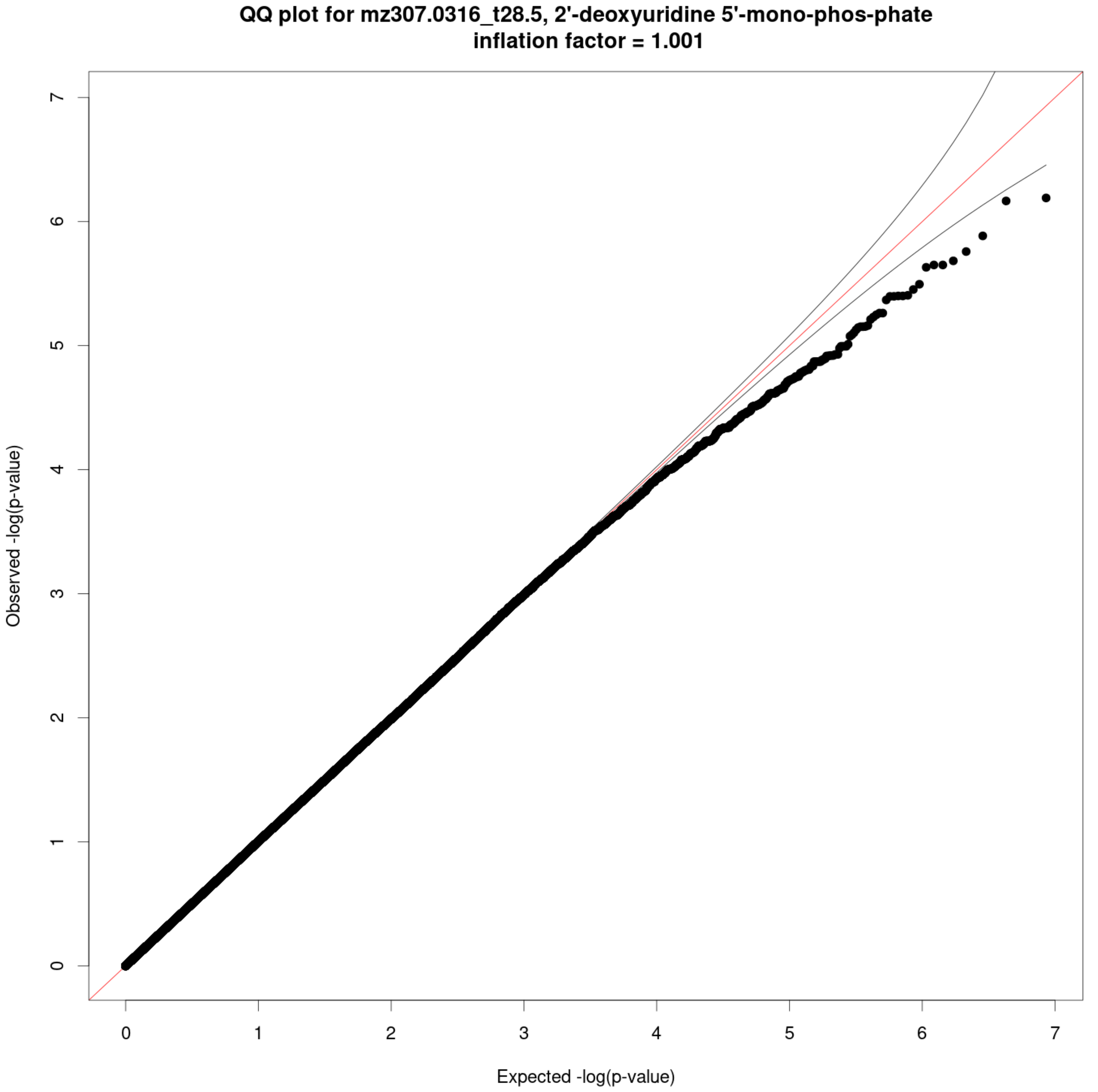
QQ plot for mz307.0316_t28.5, 2'-deoxyuridine 5'-mono-phos-phate
inflation factor = 1.001
Observed -log(p-value)
Expected -log(p-value)
0
1
2
3
4
5
6
7

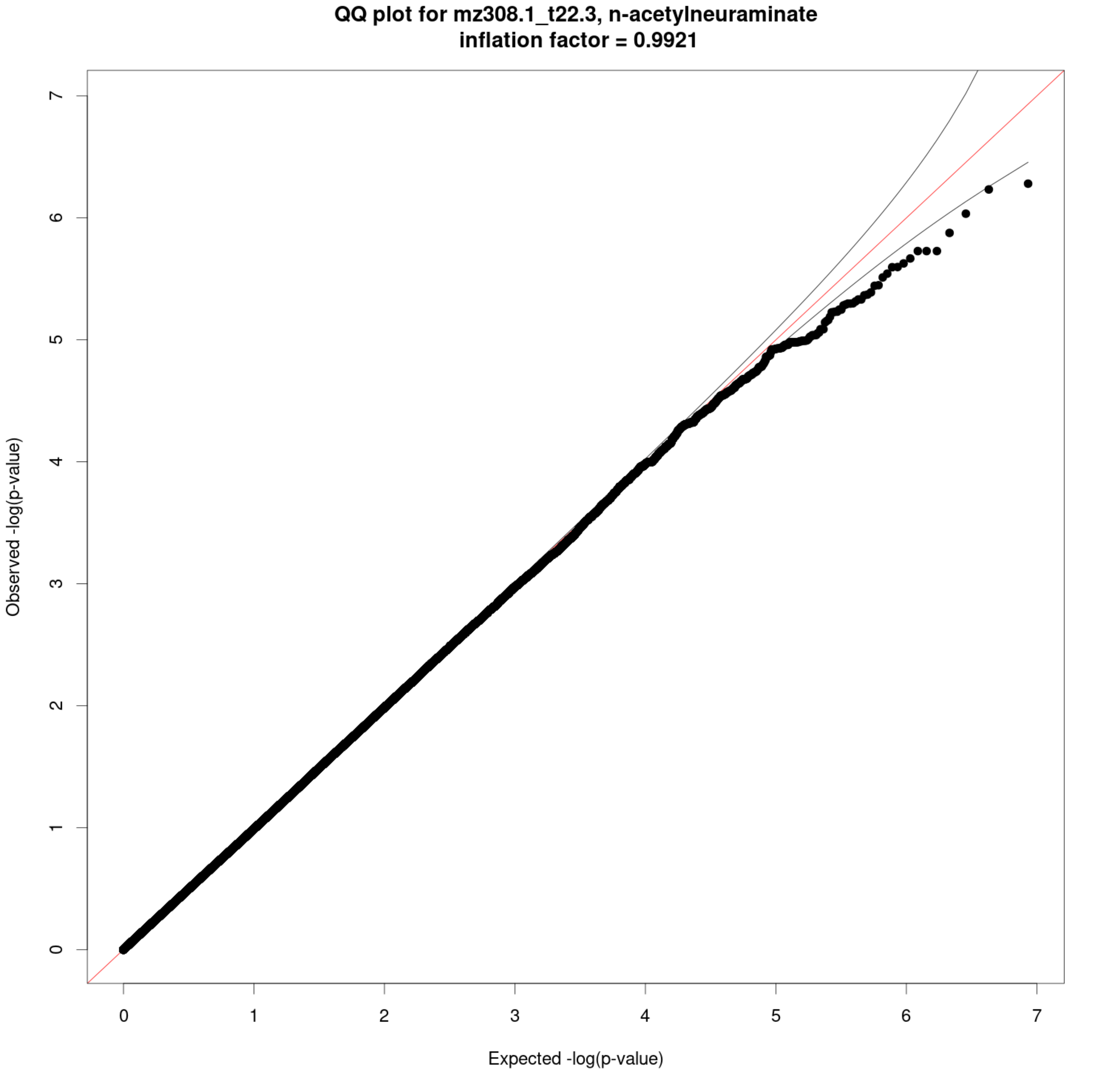
QQ plot for mz308.1_t22.3, n-acetylneuraminate
inflation factor = 0.9921
Observed -log(p-value)
Expected -log(p-value)
0
1
2
3
4
5
6
7

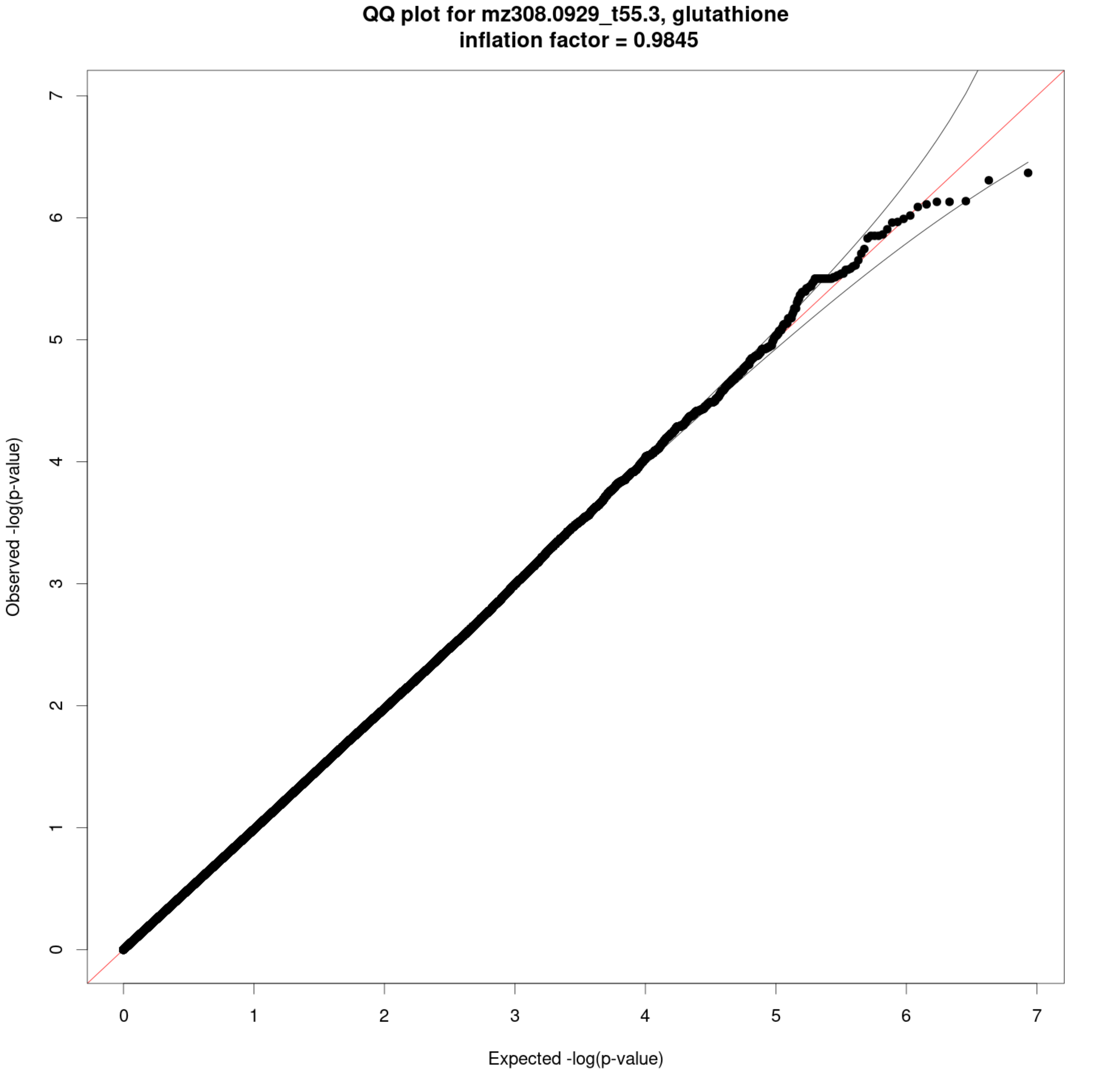
QQ plot for mz308.0929_t55.3, glutathione
inflation factor = 0.9845
Observed -log(p-value)
Expected -log(p-value)
0
1
2
3
4
5
6
7

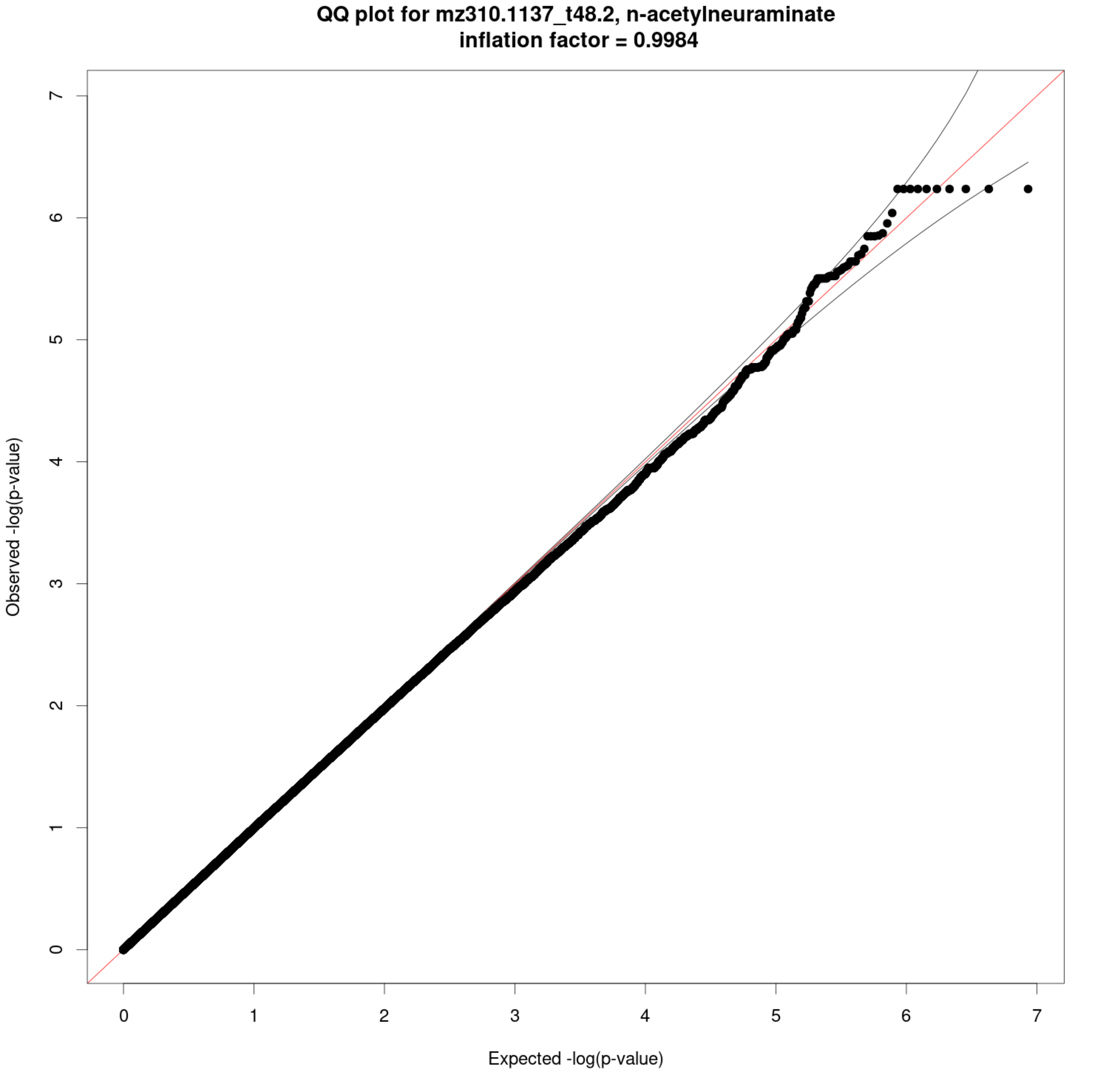
QQ plot for mz310.1137_t48.2, n-acetylneuraminate
inflation factor = 0.9984
Observed -log(p-value)
Expected -log(p-value)
0
1
2
3
4
5
6
7

QQ plot for mz311.296_t295.2, arachidic acid
inflation factor = 1.01

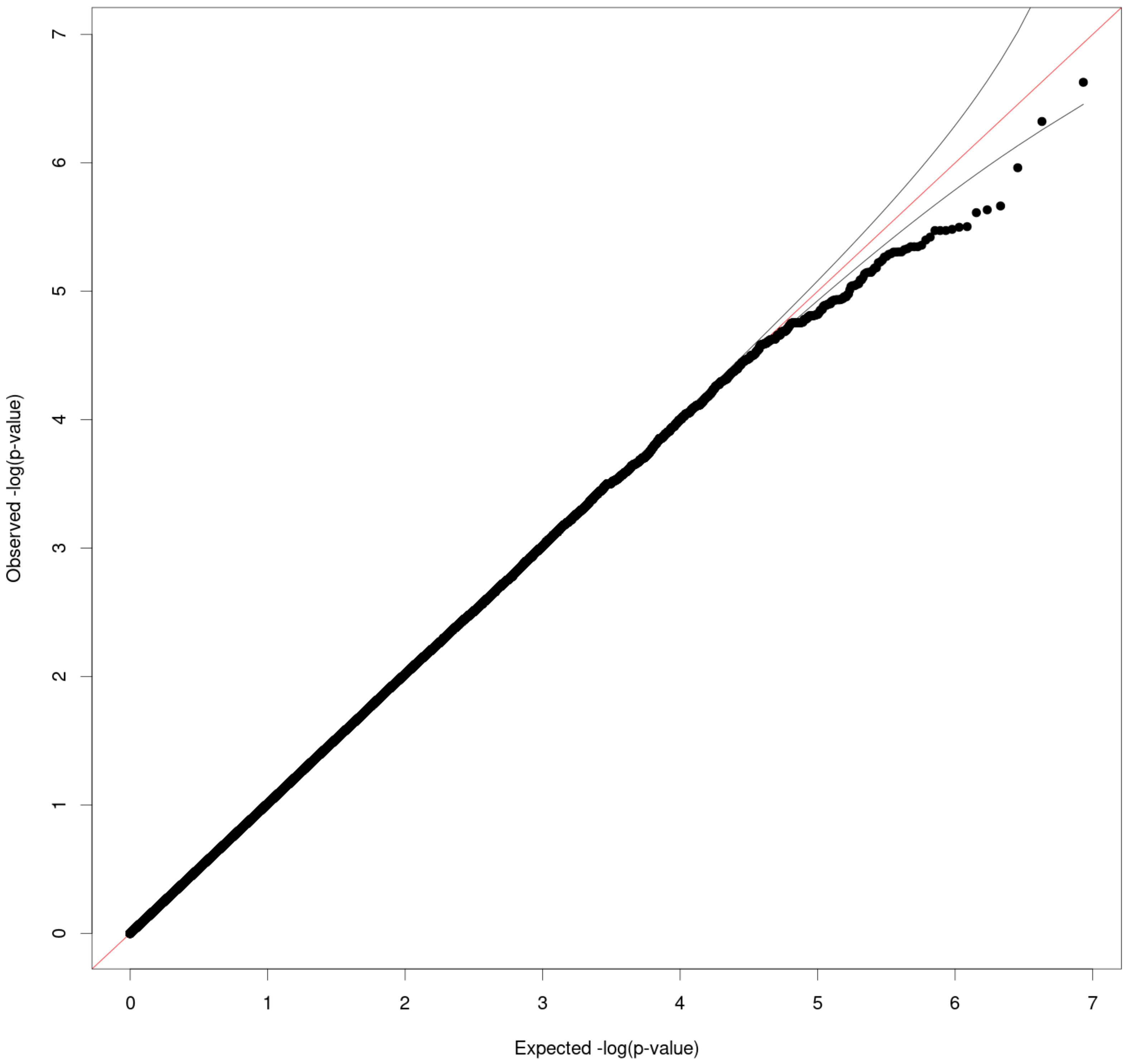

QQ plot for mz319.0692_t48.2, melanin
inflation factor = 1.002

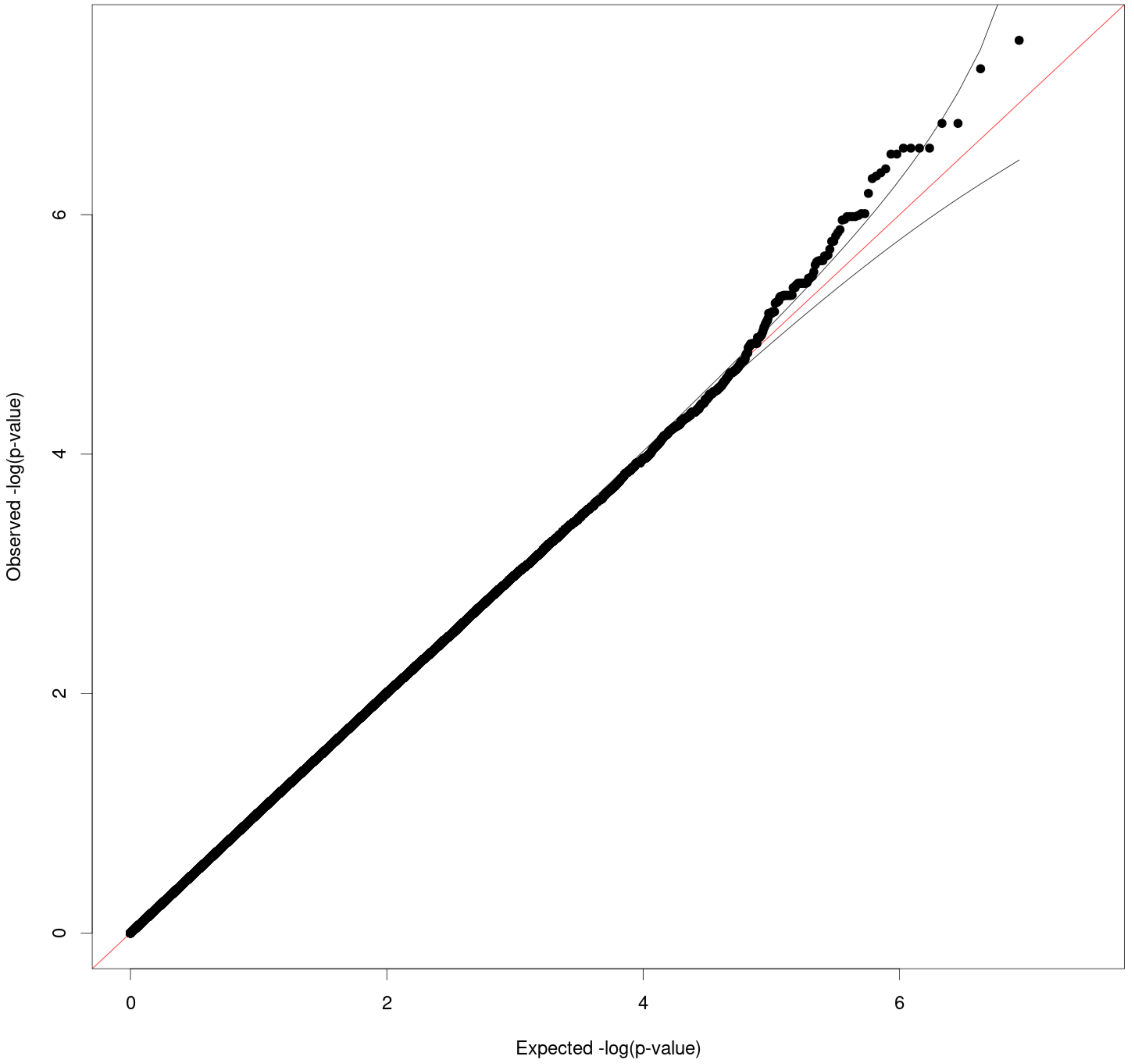

QQ plot for mz329.2502_t29.9, docosahexaenoic acid
inflation factor = 1.009

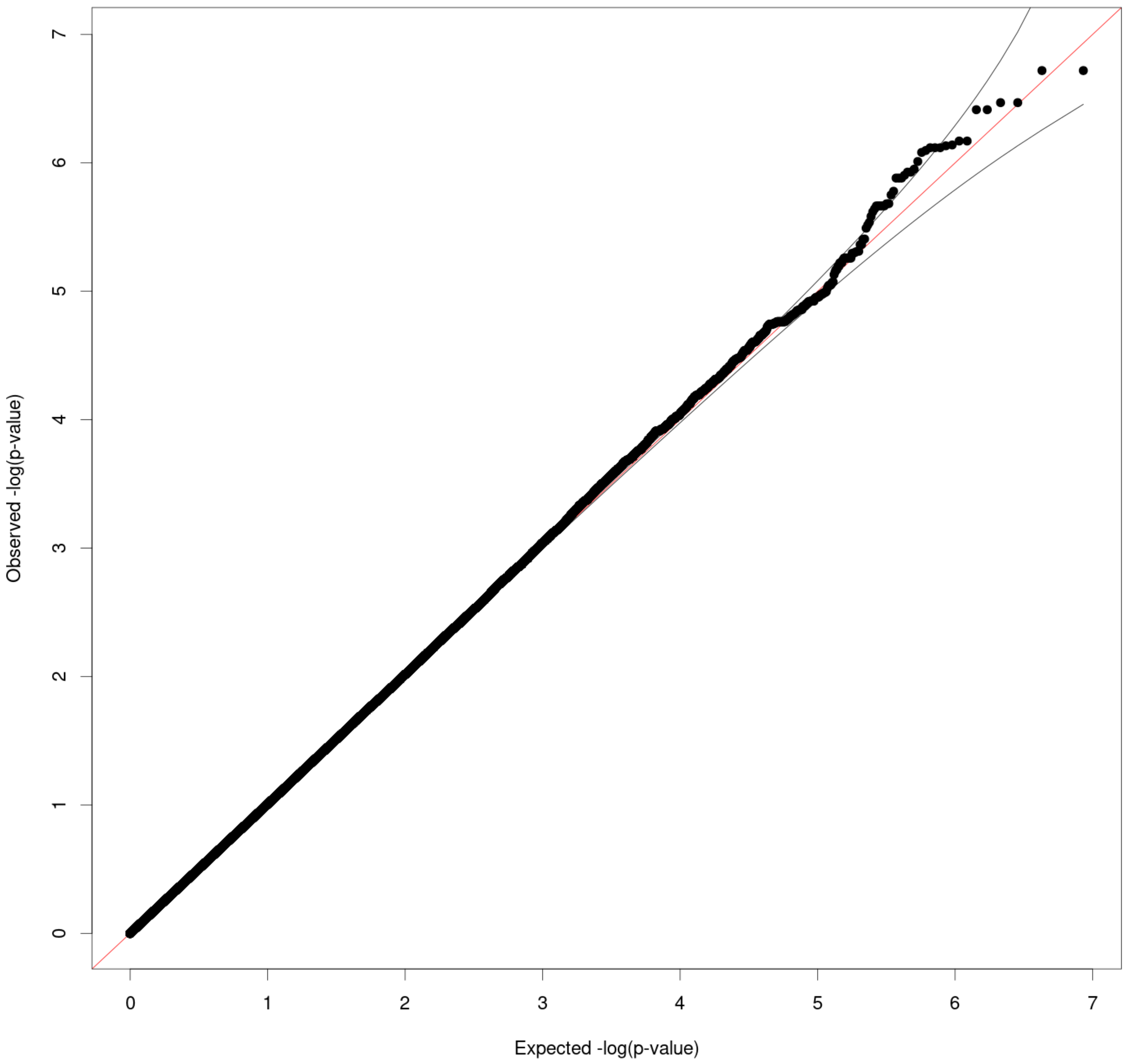

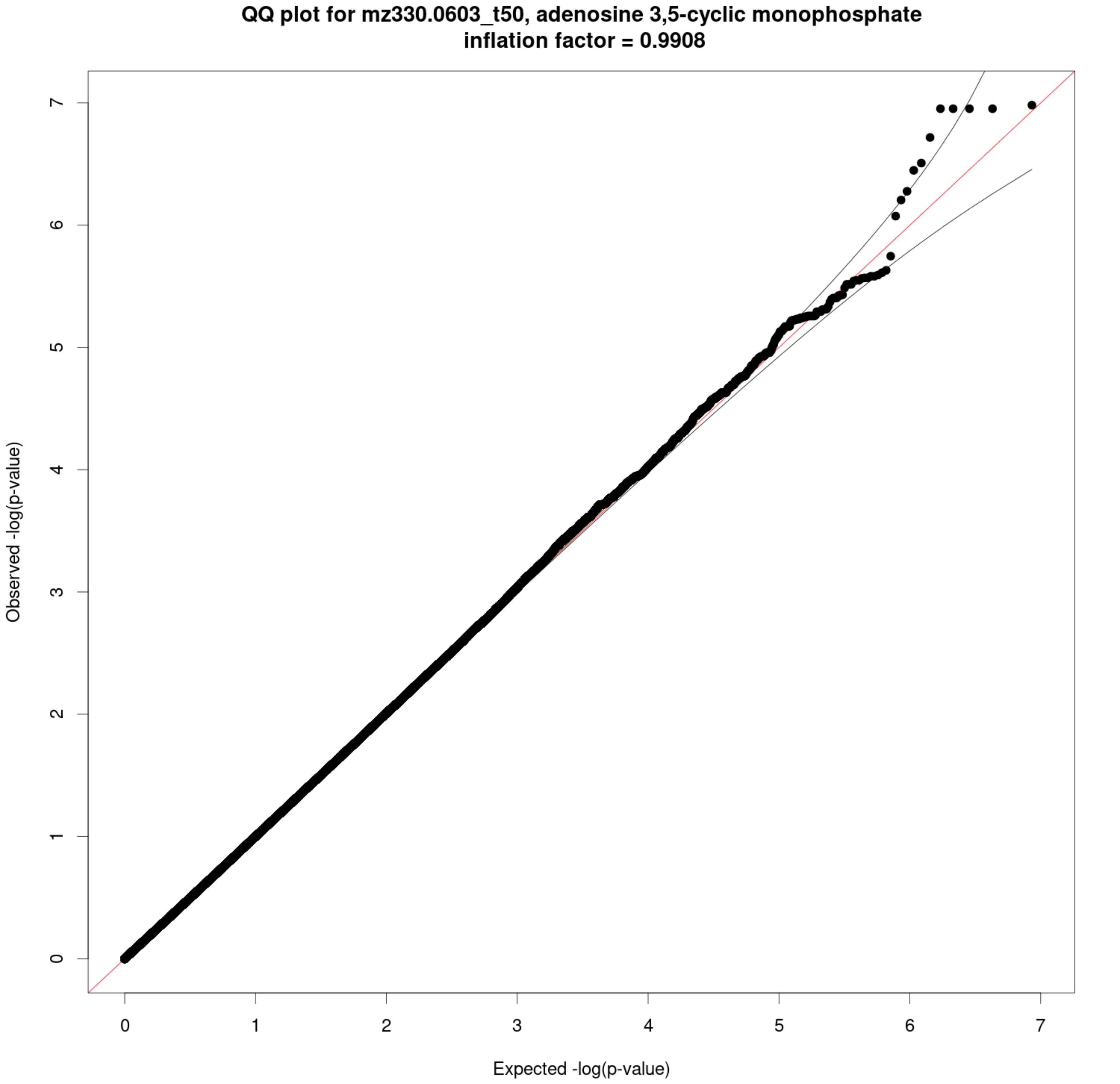
QQ plot for mz330.0603_t50, adenosine 3,5-cyclic monophosphate
inflation factor = 0.9908
Observed -log(p-value)
Expected -log(p-value)
0
1
2
3
4
5
6
7

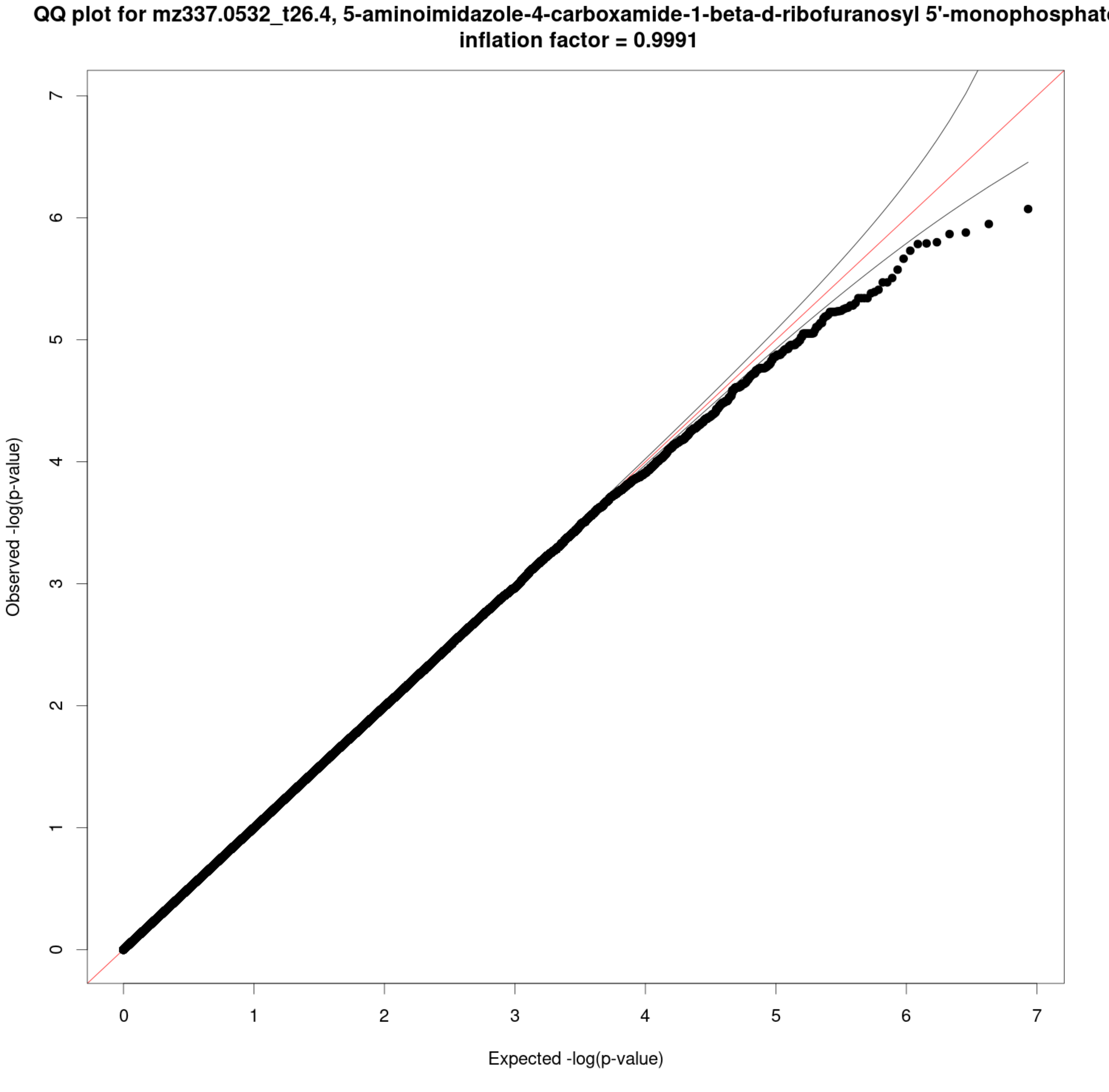
QQ plot for mz337.0532_t26.4, 5-aminoimidazole-4-carboxamide-1-beta-d-ribofuranosyl 5'-monophosphat
inflation factor = 0.9991
Observed -log(p-value)
Expected -log(p-value)
0
1
2
3
4
5
6
7

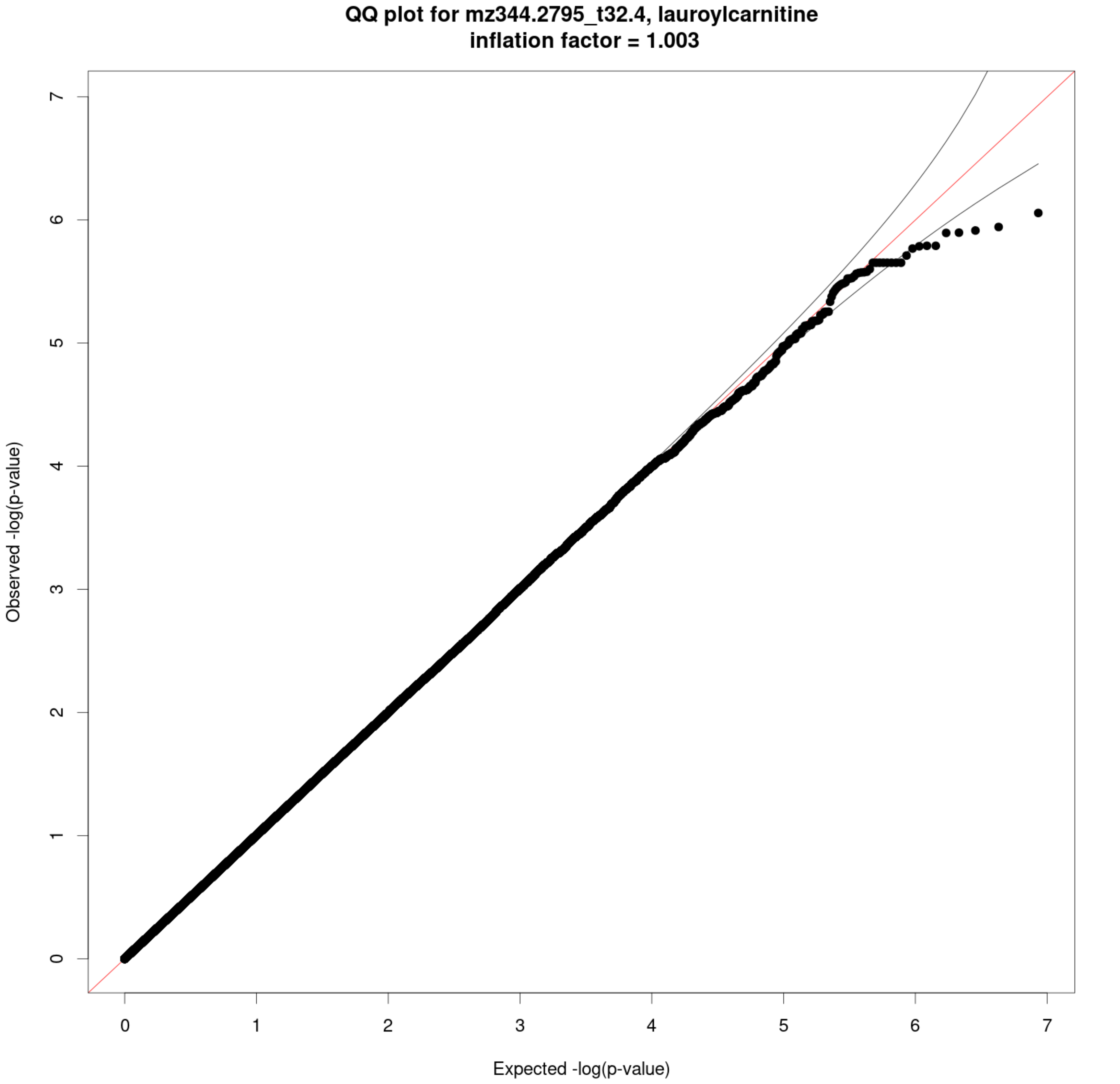
QQ plot for mz344.2795_t32.4, lauroylcarnitine
inflation factor = 1.003
Observed -log(p-value)
Expected -log(p-value)
0
1
2
3
4
5
6
7

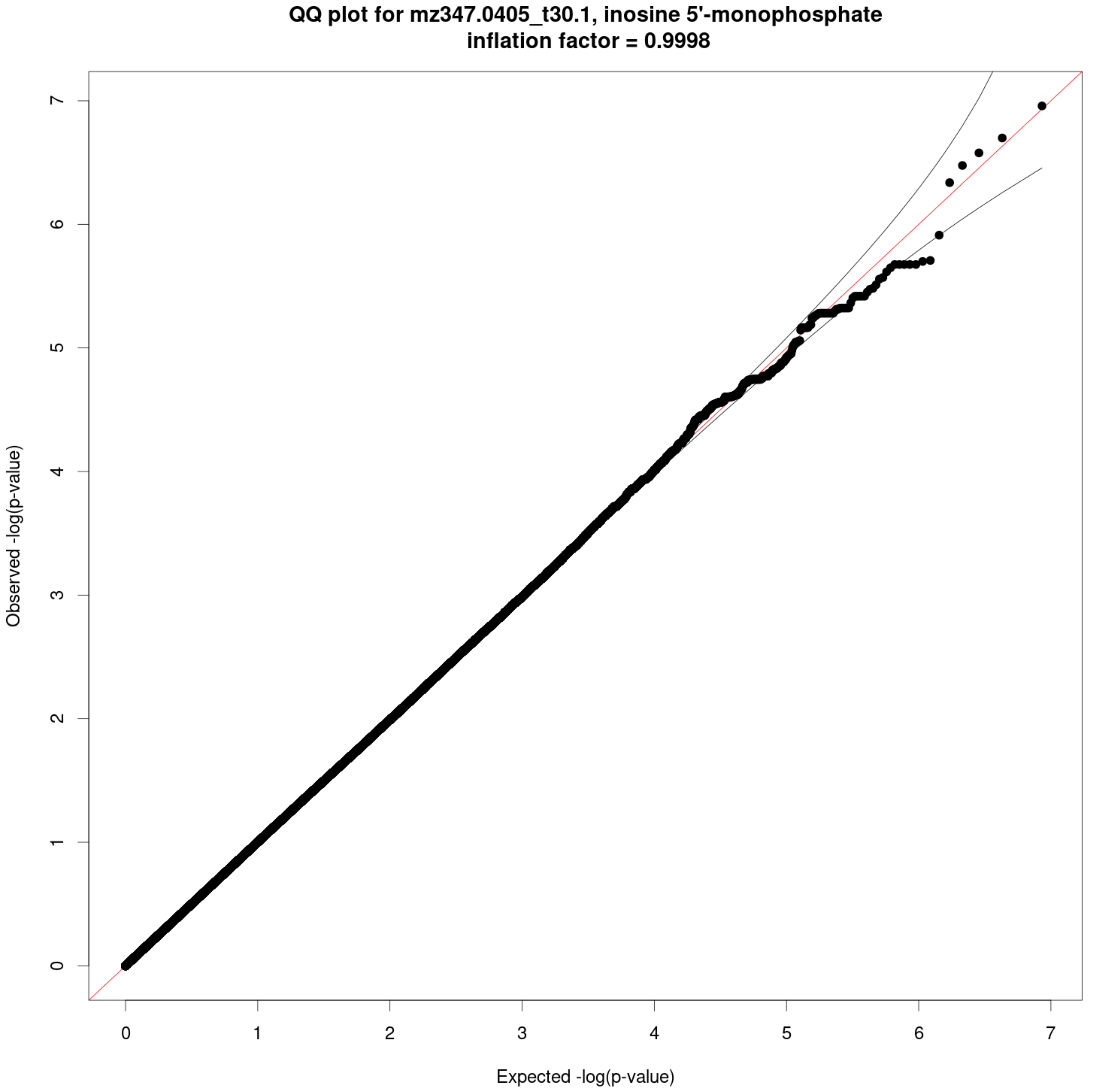
QQ plot for mz347.0405_t30.1, inosine 5'-monophosphate
inflation factor = 0.9998
Observed -log(p-value)
Expected -log(p-value)
0
1
2
3
4
5
6
7

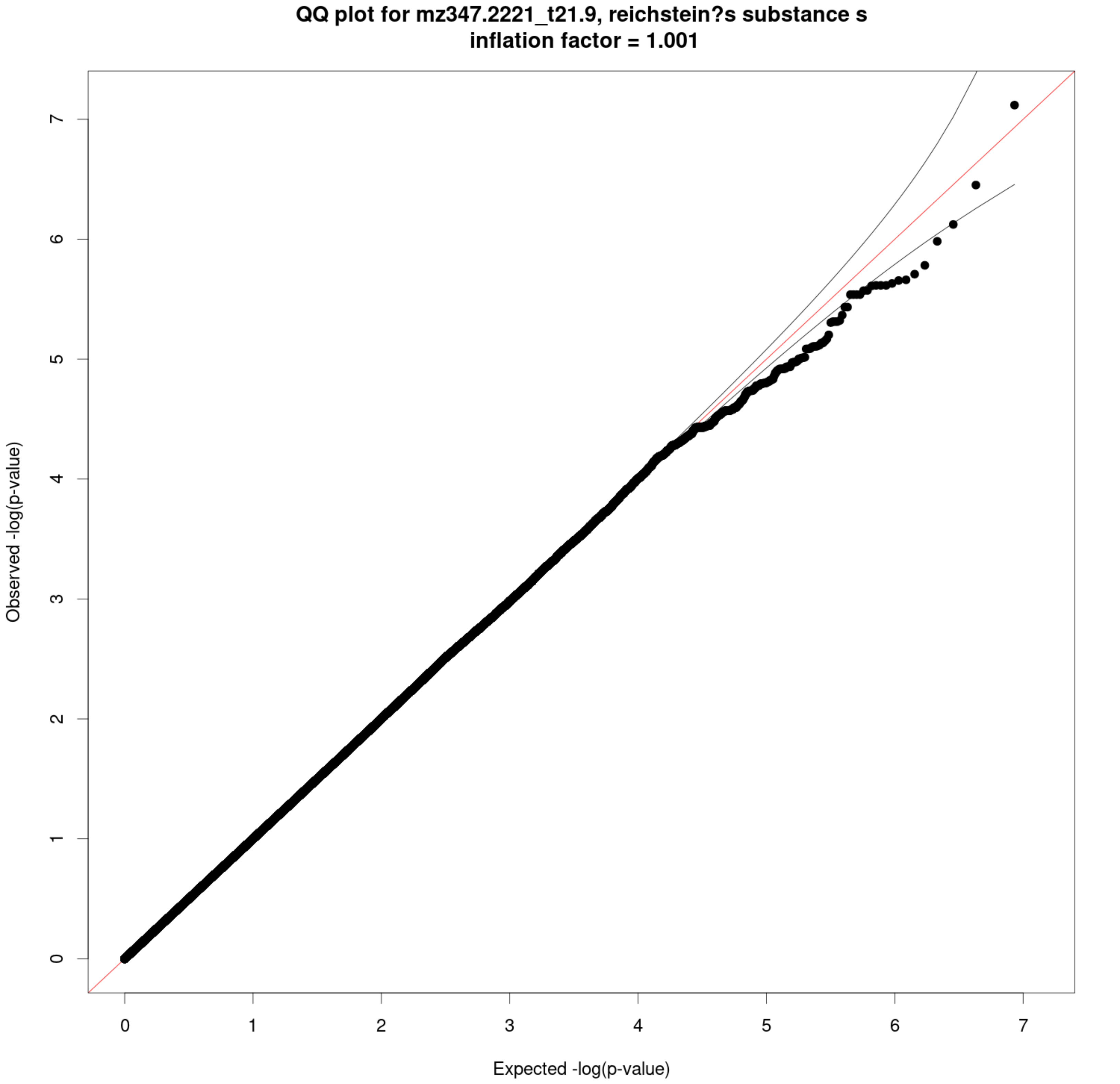
QQ plot for mz347.2221_t21.9, reichstein?s substance s
inflation factor = 1.001
Observed -log(p-value)
Expected -log(p-value)
0
1
2
3
4
5
6
7

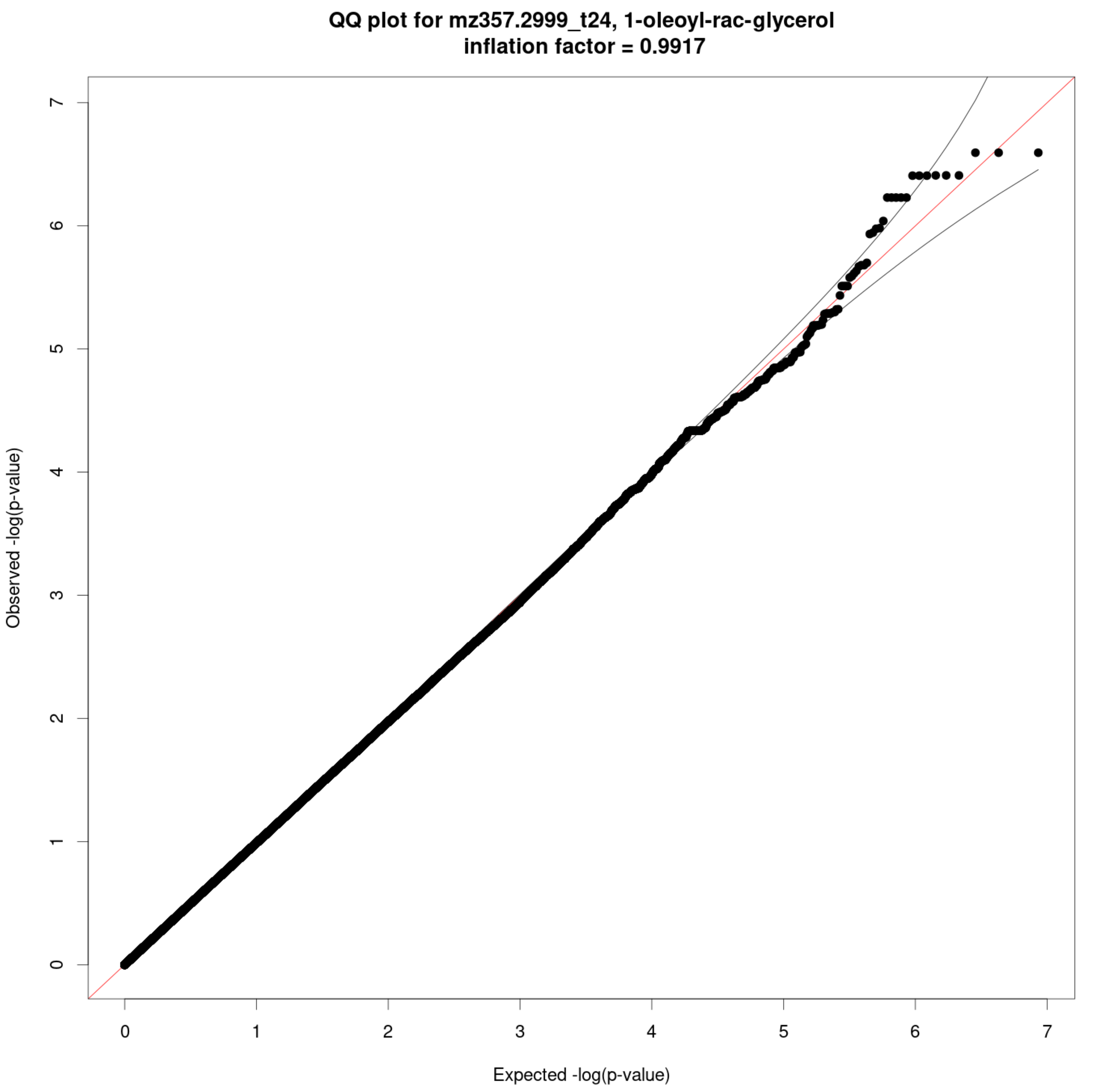
QQ plot for mz357.2999_t24, 1-oleoyl-rac-glycerol
inflation factor = 0.9917
Observed -log(p-value)
Expected -log(p-value)
0
1
2
3
4
5
6
7

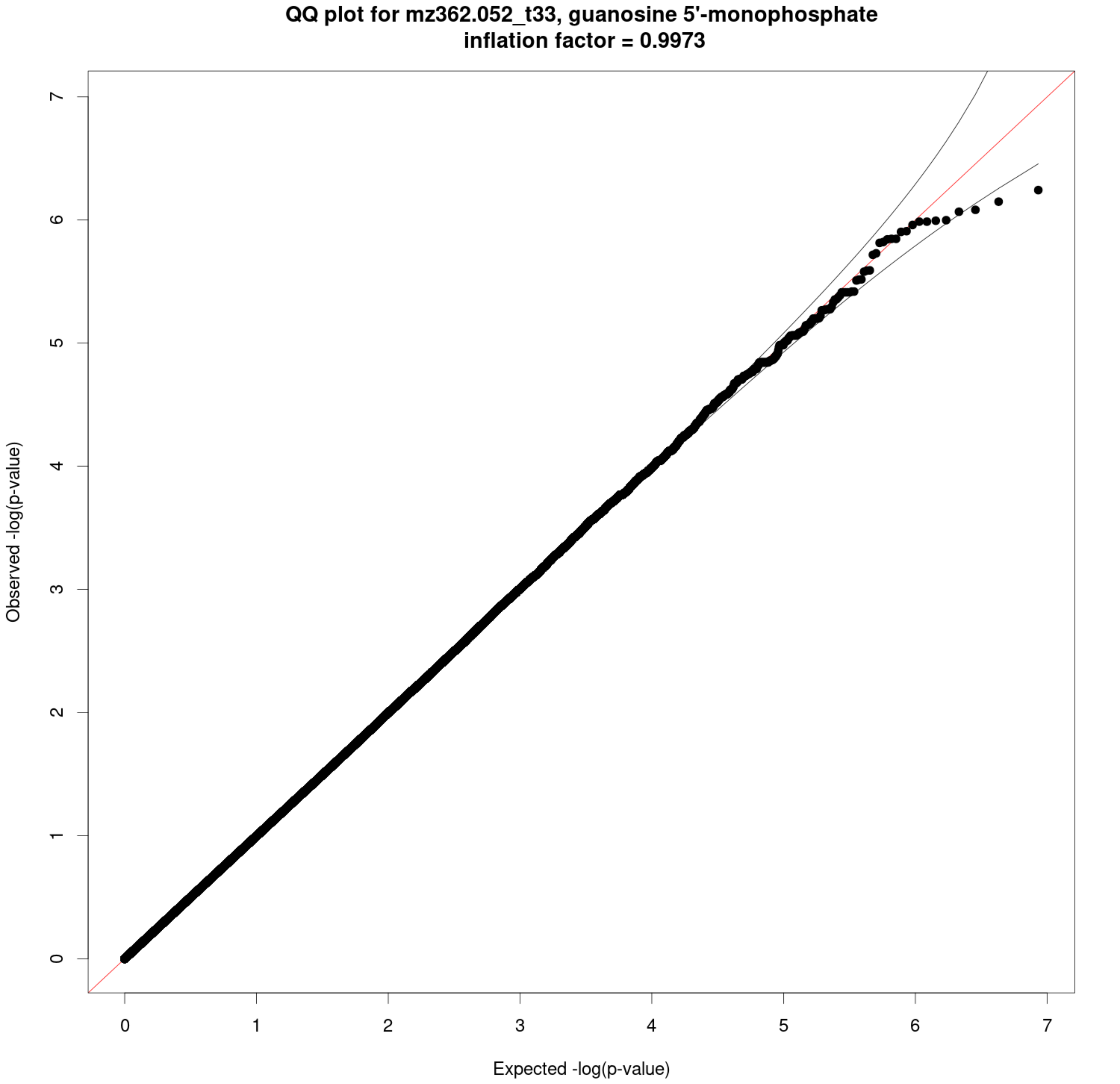
QQ plot for mz362.052_t33, guanosine 5'-monophosphate
inflation factor = 0.9973
Observed -log(p-value)
Expected -log(p-value)
0
1
2
3
4
5
6
7

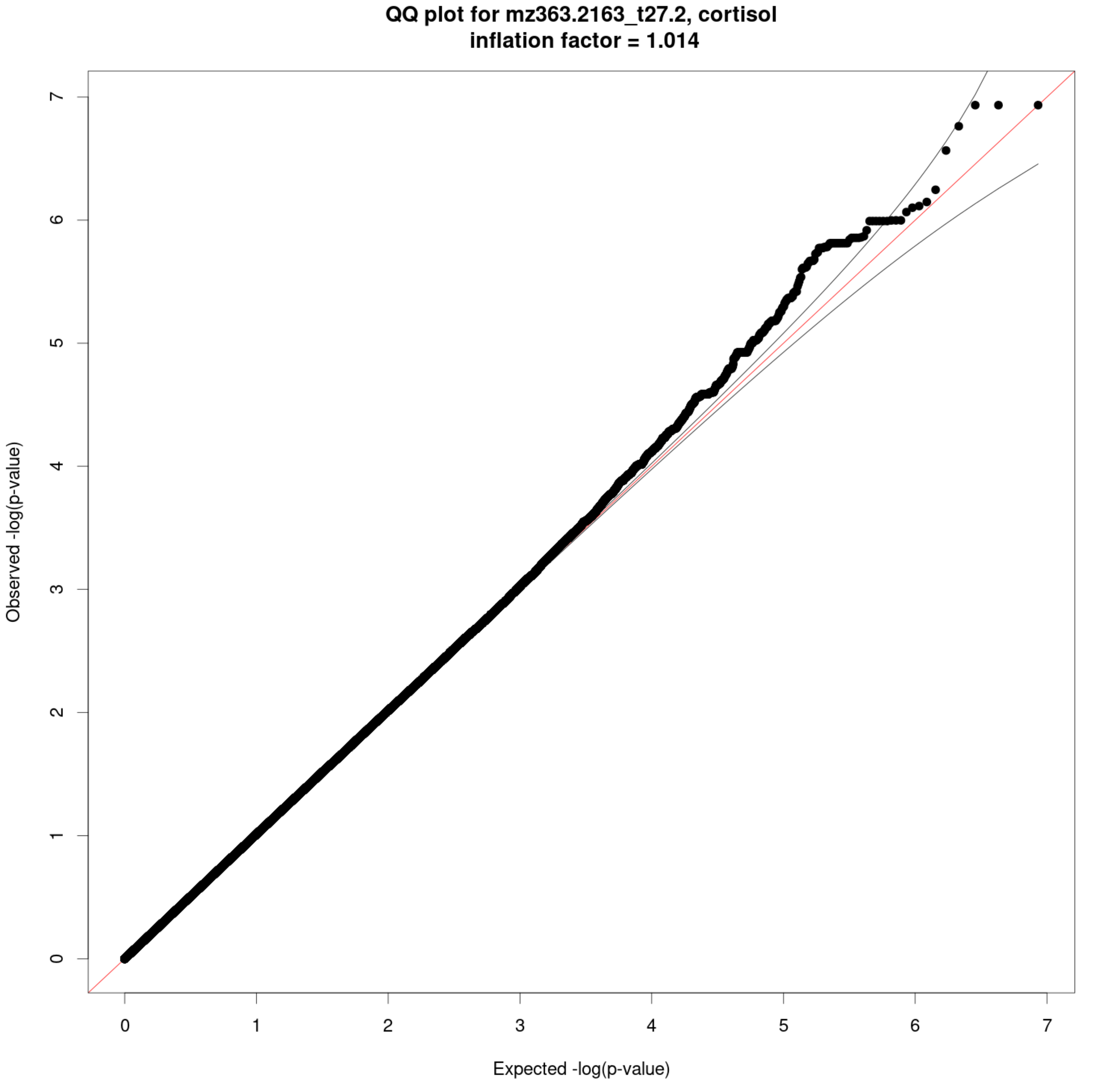
QQ plot for mz363.2163_t27.2, cortisol
inflation factor = 1.014
Observed -log(p-value)
Expected -log(p-value)
0
1
2
3
4
5
6
7

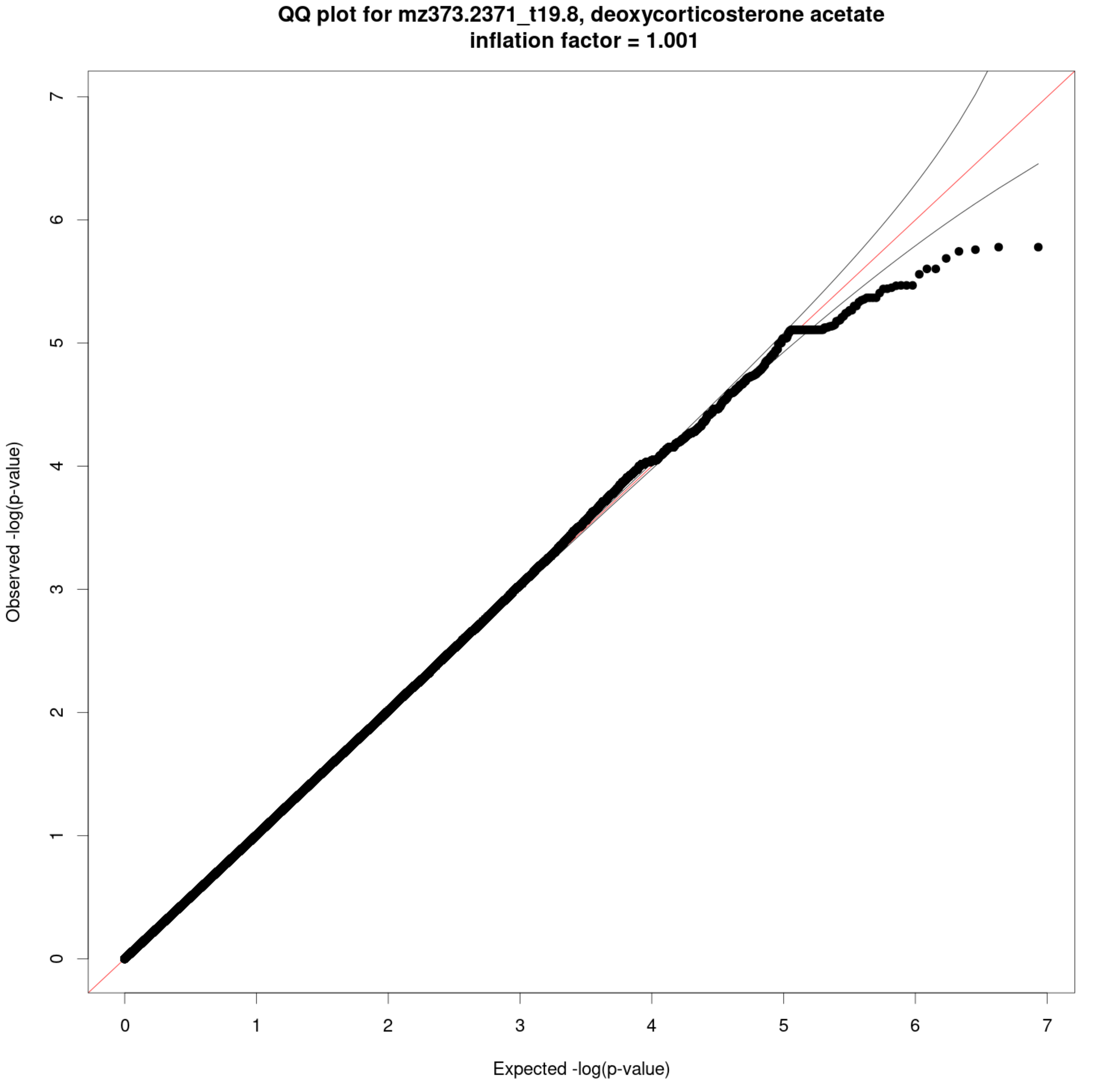
QQ plot for mz373.2371_t19.8, deoxycorticosterone acetate
inflation factor = 1.001
Observed -log(p-value)
Expected -log(p-value)
0
1
2
3
4
5
6
7

QQ plot for mz375.2926_t209.2, 3alpha-hydroxy-5beta-cholanate
inflation factor = 0.9984

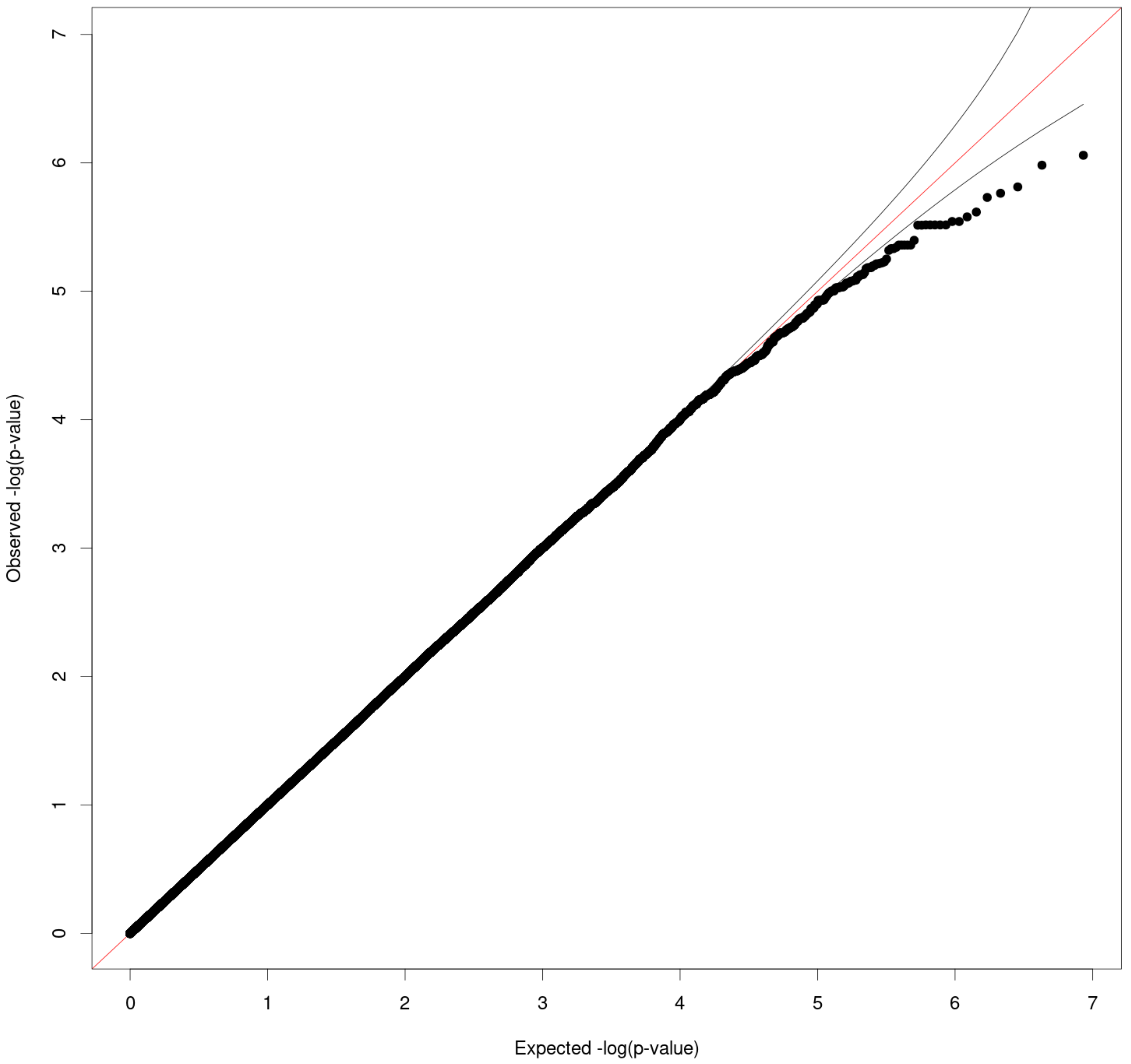

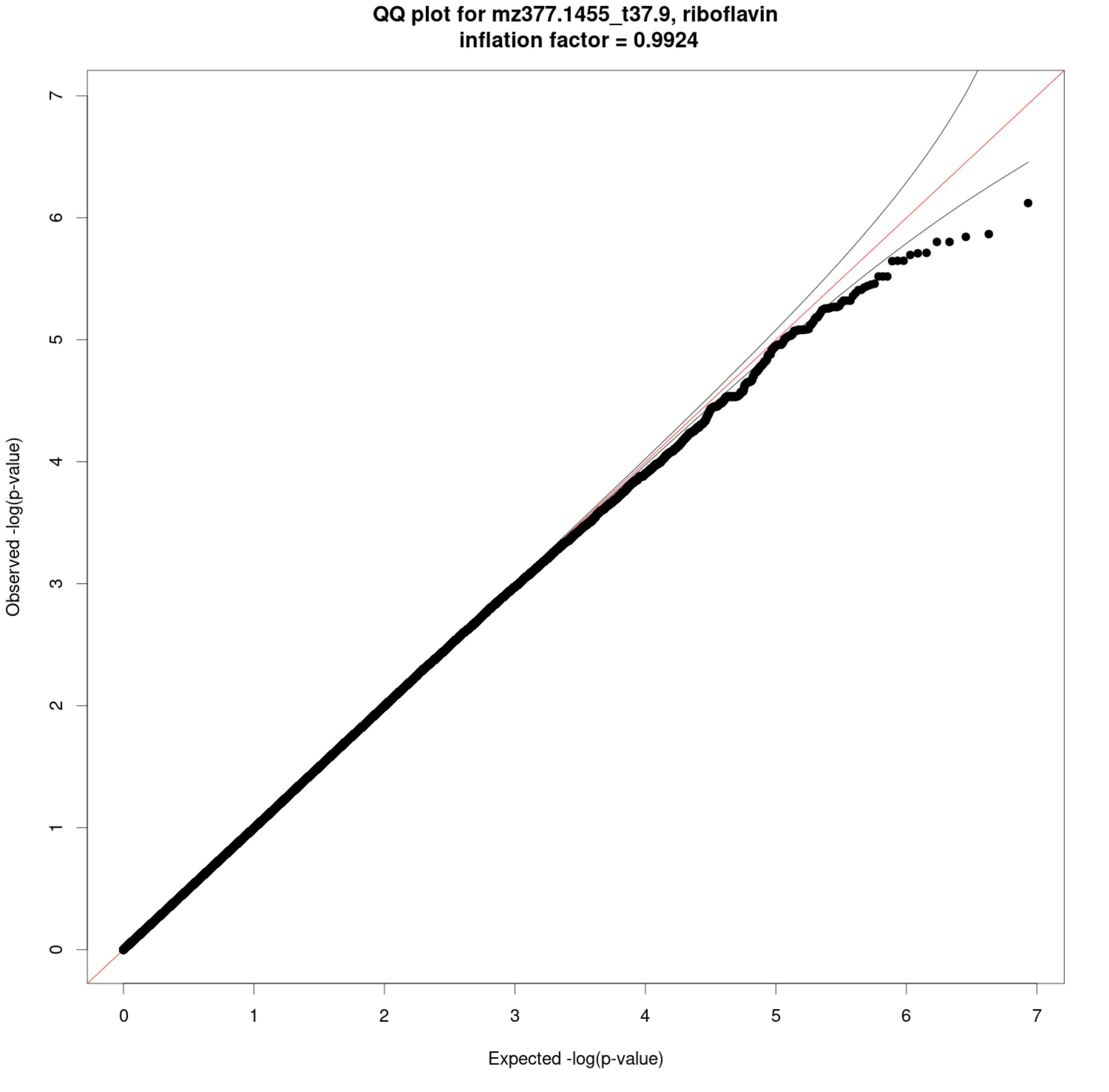
QQ plot for mz377.1455_t37.9, riboflavin
inflation factor = 0.9924
Observed -log(p-value)
Expected -log(p-value)
0
1
2
3
4
5
6
7

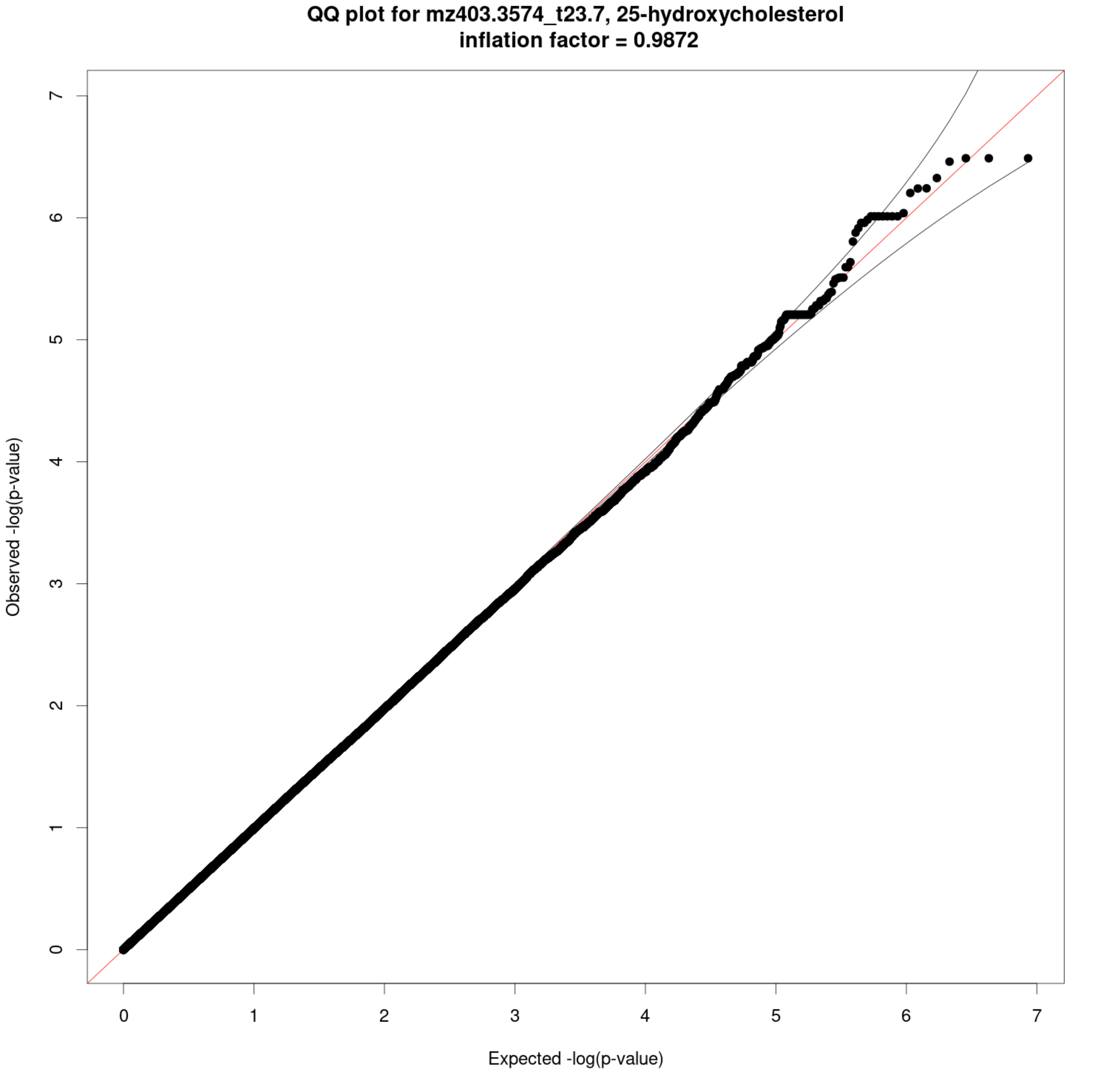
QQ plot for mz403.3574_t23.7, 25-hydroxycholesterol
inflation factor = 0.9872
Observed -log(p-value)
Expected -log(p-value)
0
1
2
3
4
5
6
7

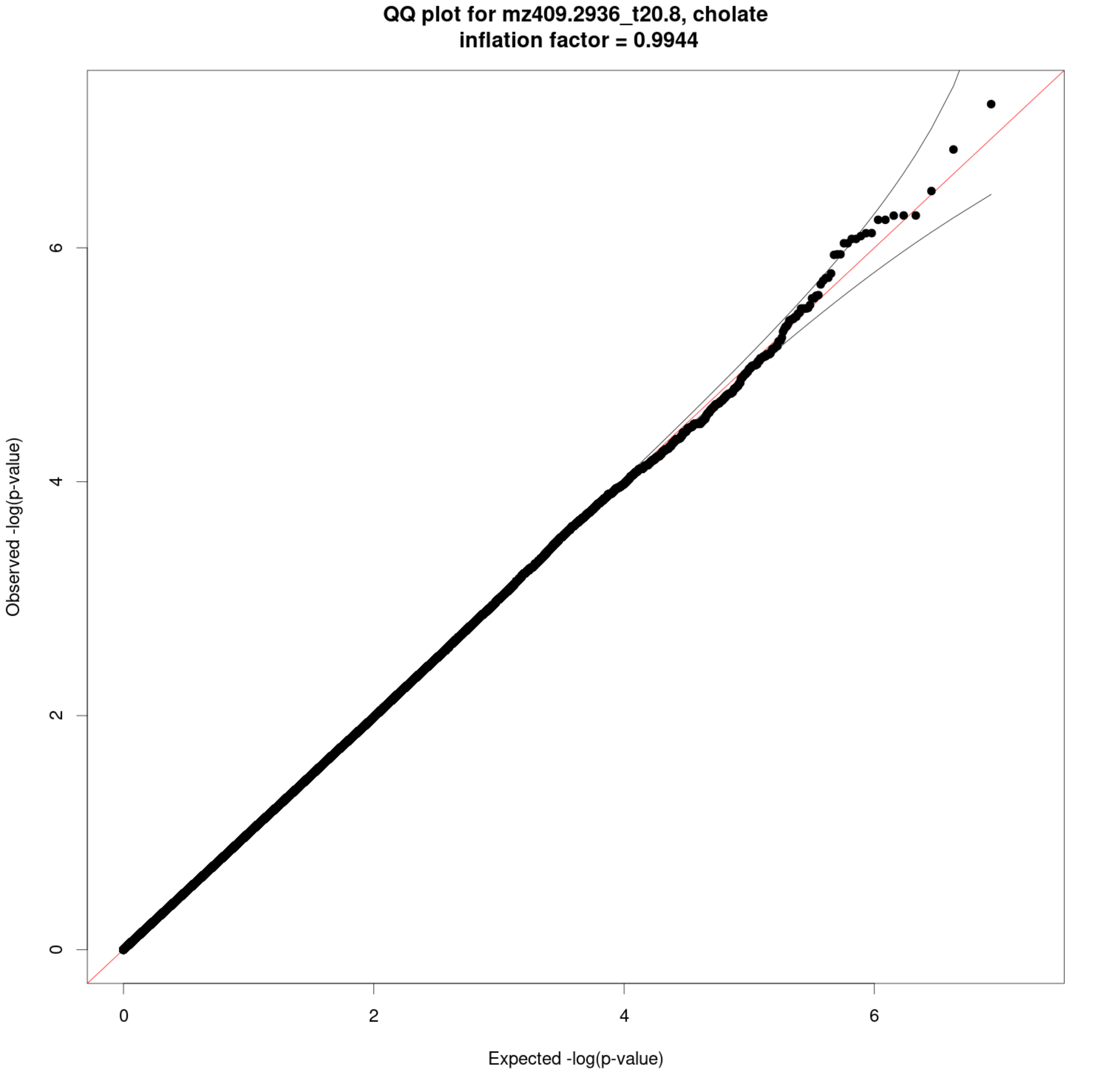
QQ plot for mz409.2936_t20.8, cholate
inflation factor = 0.9944
Observed -log(p-value)
Expected -log(p-value)
0
2
4
6
0
2
4
6

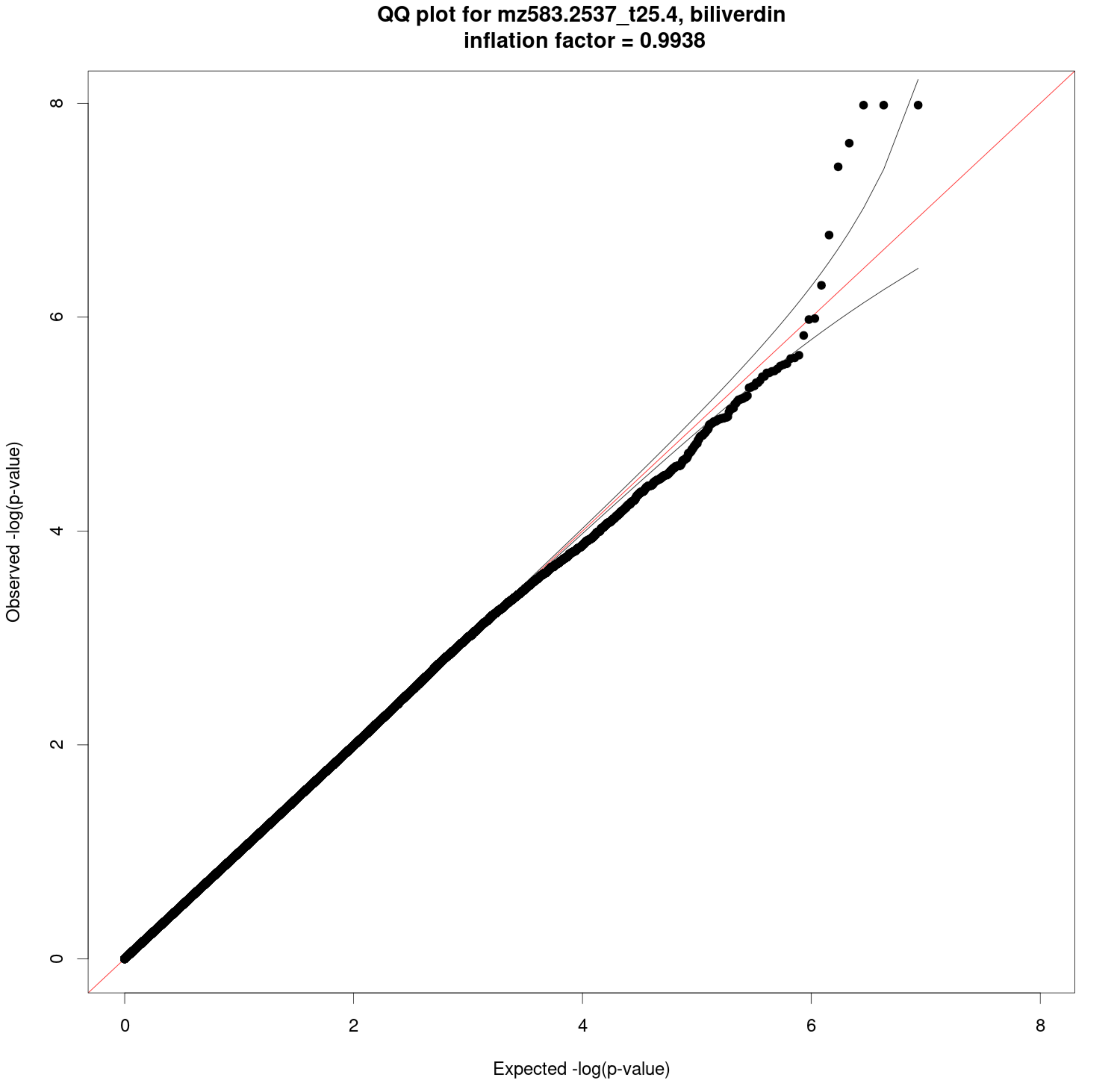
QQ plot for mz583.2537_t25.4, biliverdin
inflation factor = 0.9938
Observed -log(p-value)
Expected -log(p-value)
0
2
4
6
8

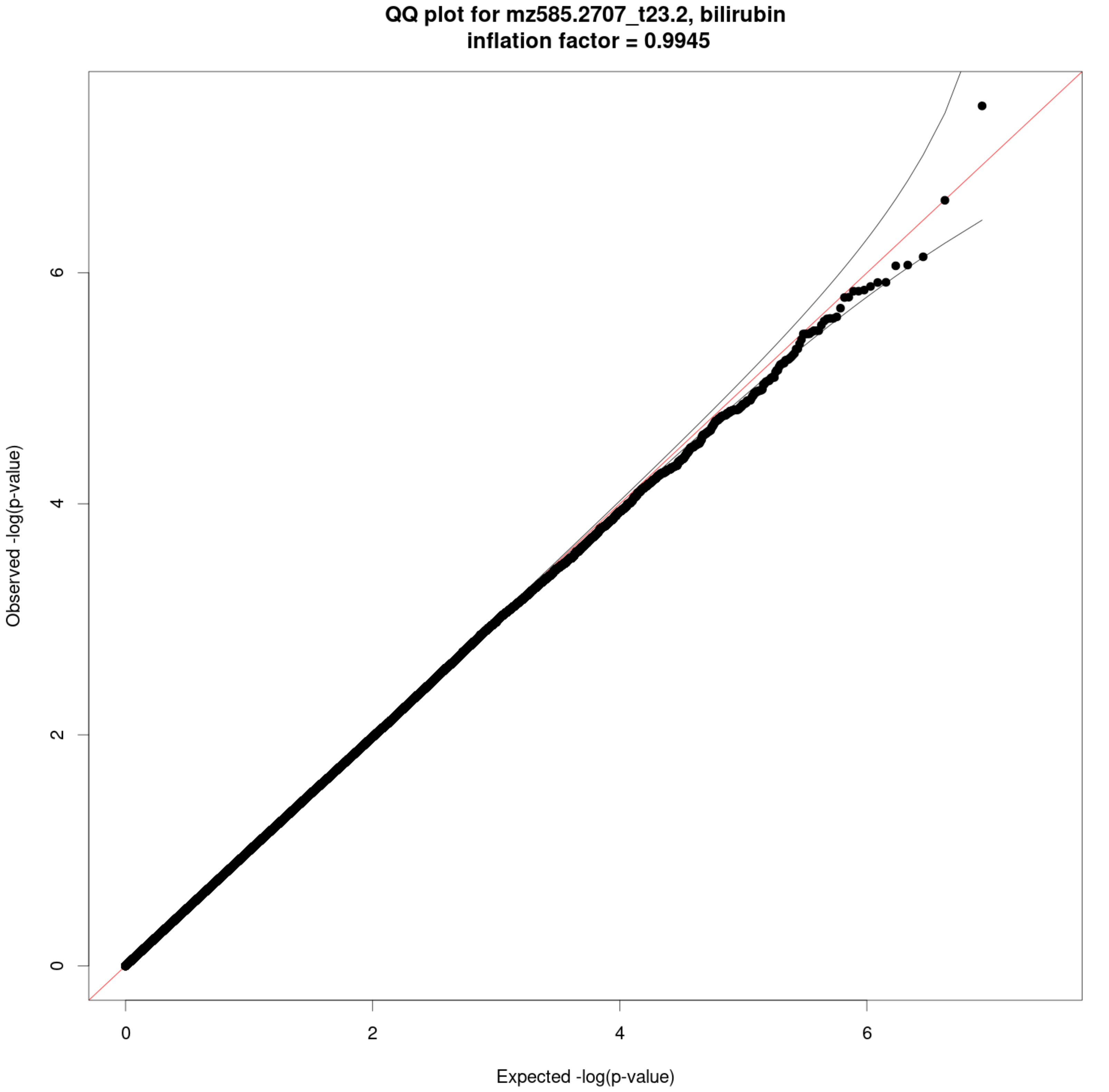
QQ plot for mz585.2707_t23.2, bilirubin
inflation factor = 0.9945
Observed -log(p-value)
Expected -log(p-value)
0
2
4
6
0
2
4
6

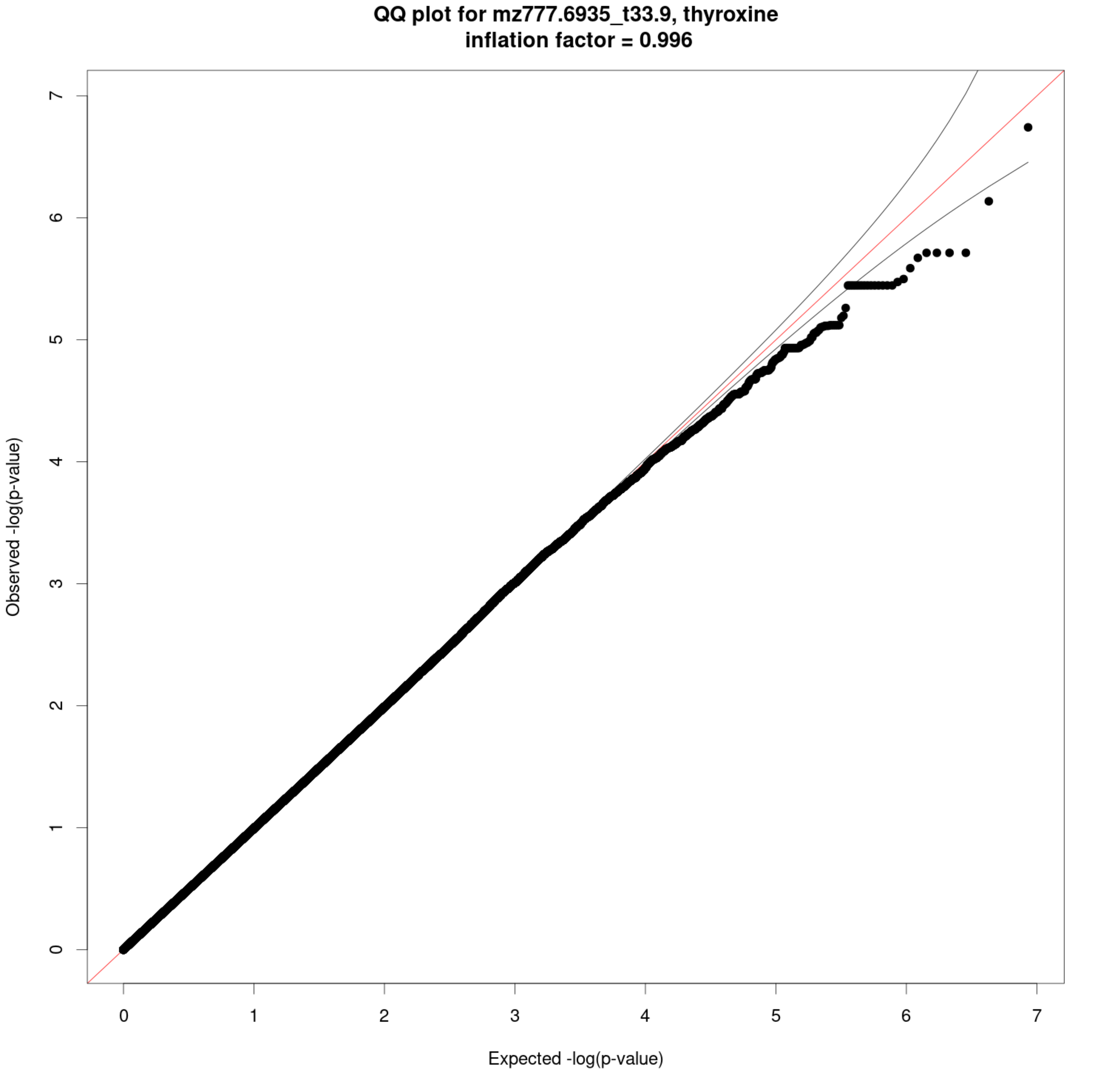
QQ plot for mz777.6935_t33.9, thyroxine
inflation factor = 0.996
Observed -log(p-value)
Expected -log(p-value)
0
1
2
3
4
5
6
7
