# Supplementary material for: Crosstalk between Host Genome and Metabolome among People with HIV in South Africa
Source: Metabolites. 2022 Jul 6;12(7):624. doi: 10.3390/metabo12070624 (PMC9316179; doi:10.3390/metabo12070624)

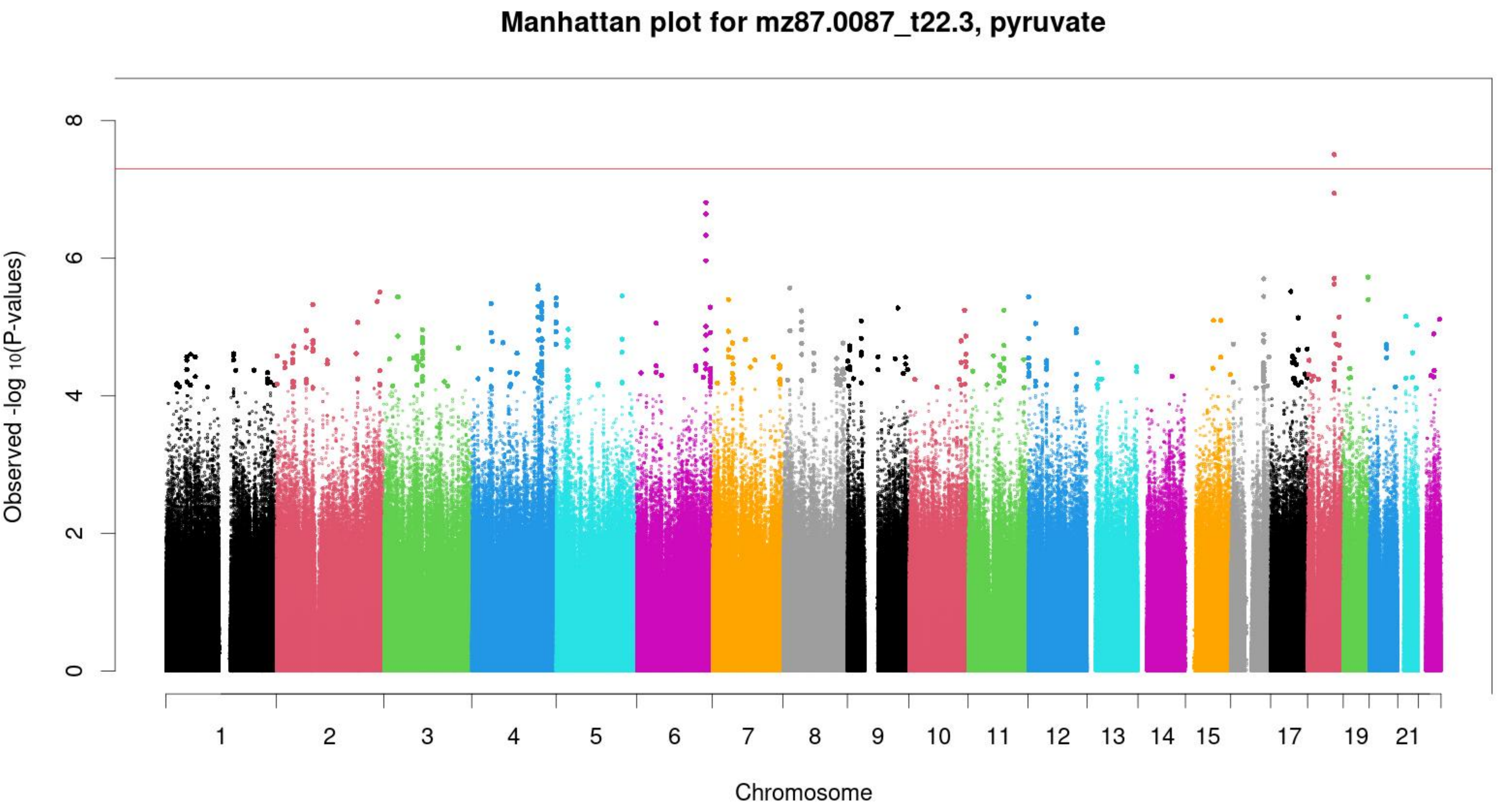

Manhattan plot for mz87.0087_t22.3, pyruvate
Observed -log 10(P-values)
0
2
4
6
8
Chromosome
1
2
3
4
5
6
7
8
9
10
11
12
13
14
15
17
19
21

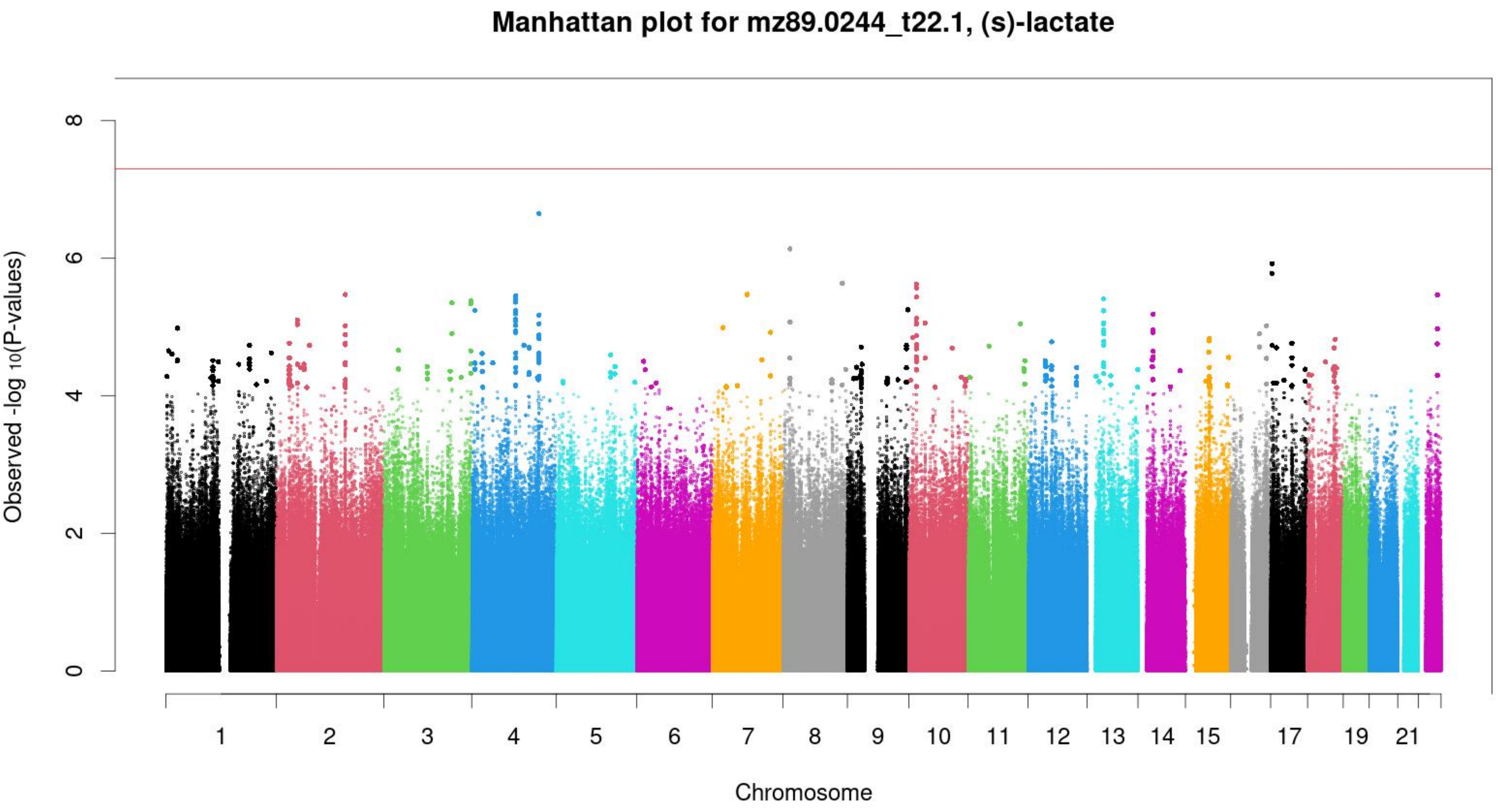

Manhattan plot for mz89.0244_t22.1, (s)-lactate
Observed -log 10(P-values)
0
2
4
6
8
1
2
3
4
5
6
7
8
9
10
11
12
13
14
15
17
19
21
Chromosome

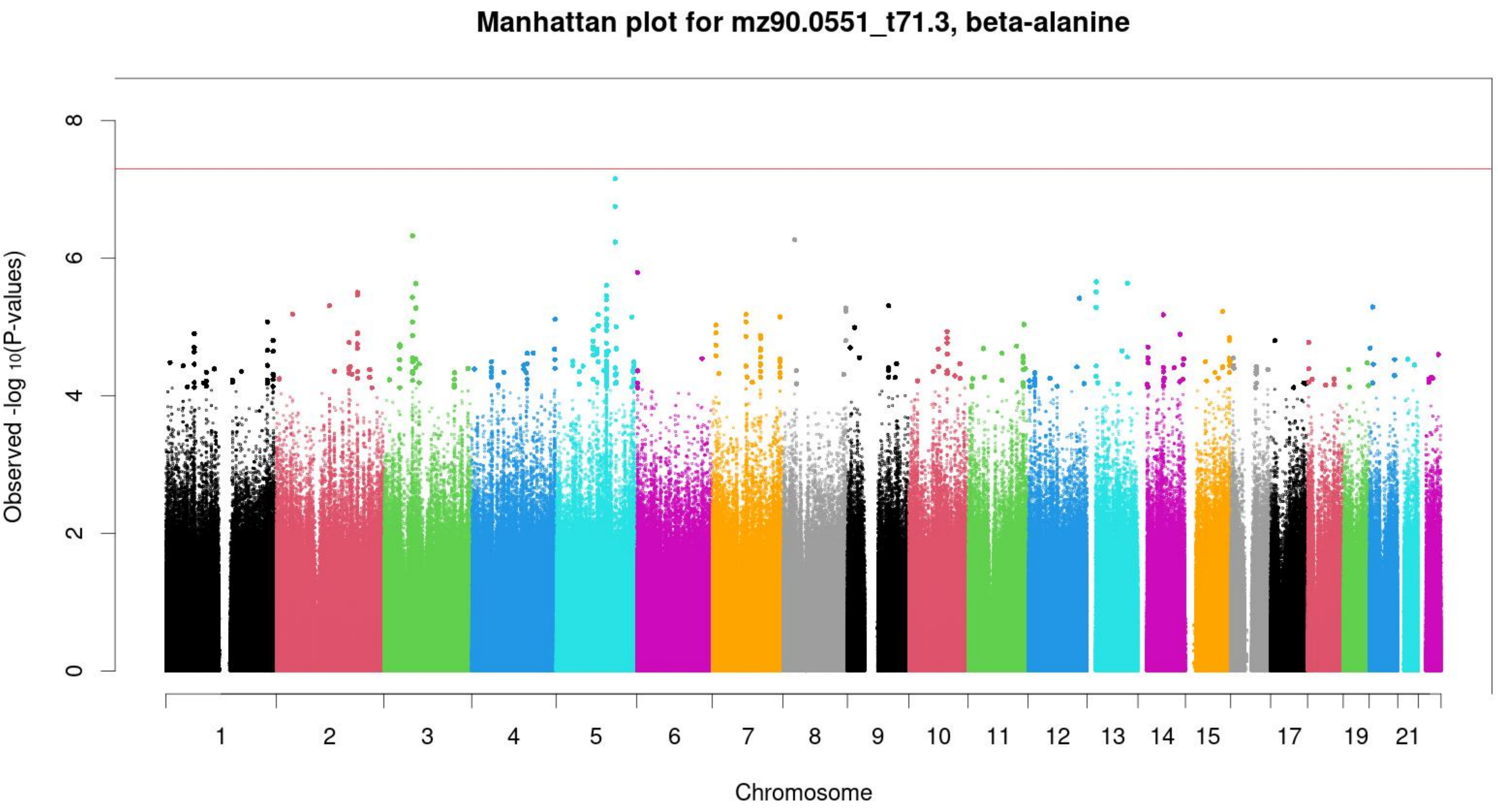

Manhattan plot for mz90.0551_t71.3, beta-alanine
Observed -log 10(P-values)
0
2
4
6
8
1
2
3
4
5
6
7
8
9
10
11
12
13
14
15
17
19
21
Chromosome

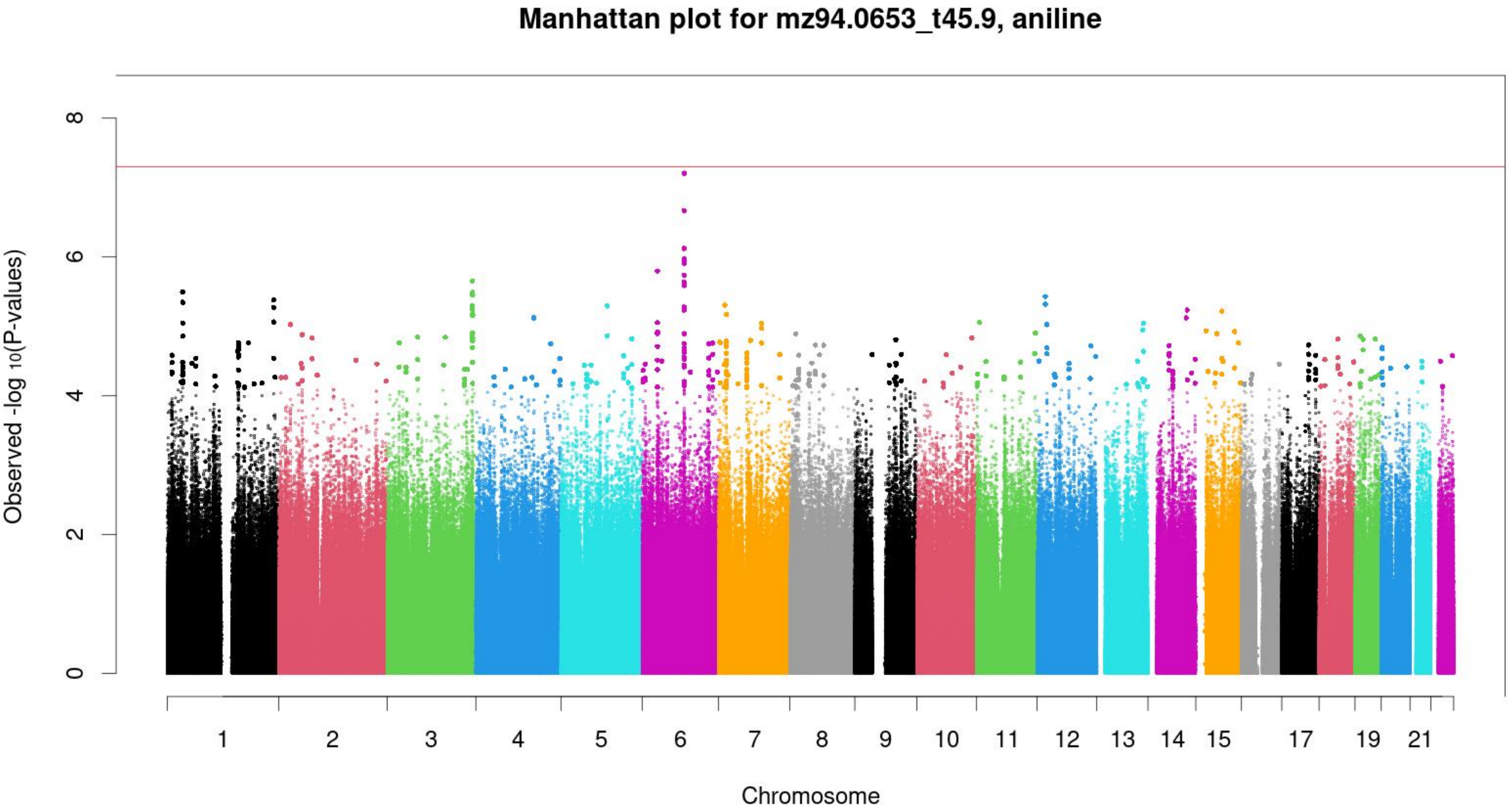

Manhattan plot for mz94.0653_t45.9, aniline
Observed -log 10(P-values)
0
2
4
6
8
1
2
3
4
5
6
7
8
9
10
11
12
13
14
15
17
19
21
Chromosome

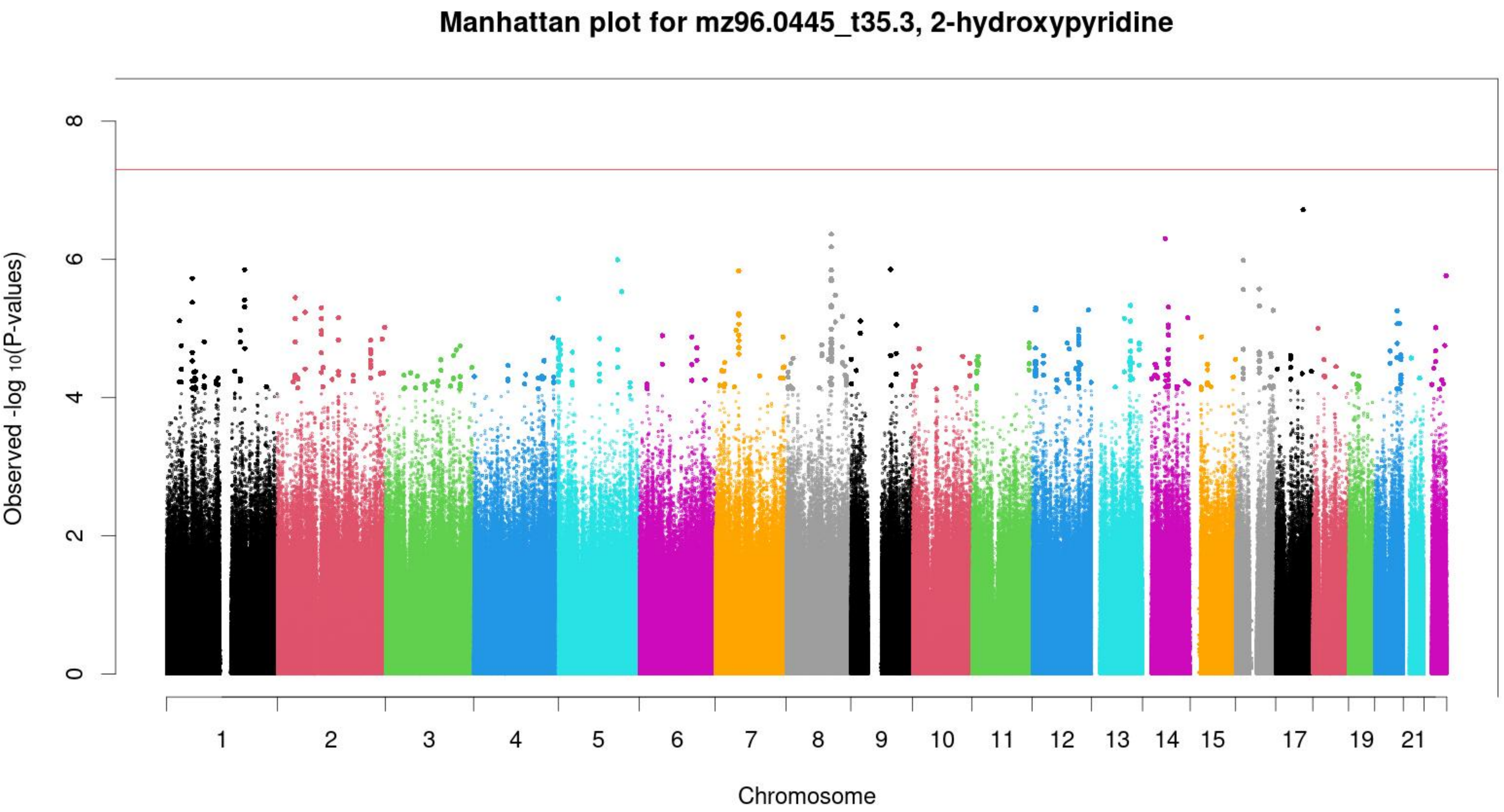

Manhattan plot for mz96.0445_t35.3, 2-hydroxypyridine
Observed -log 10(P-values)
0
2
4
6
8
1
2
3
4
5
6
7
8
9
10
11
12
13
14
15
17
19
21
Chromosome

Manhattan plot for mz101.06_t20, 5-valerolactone

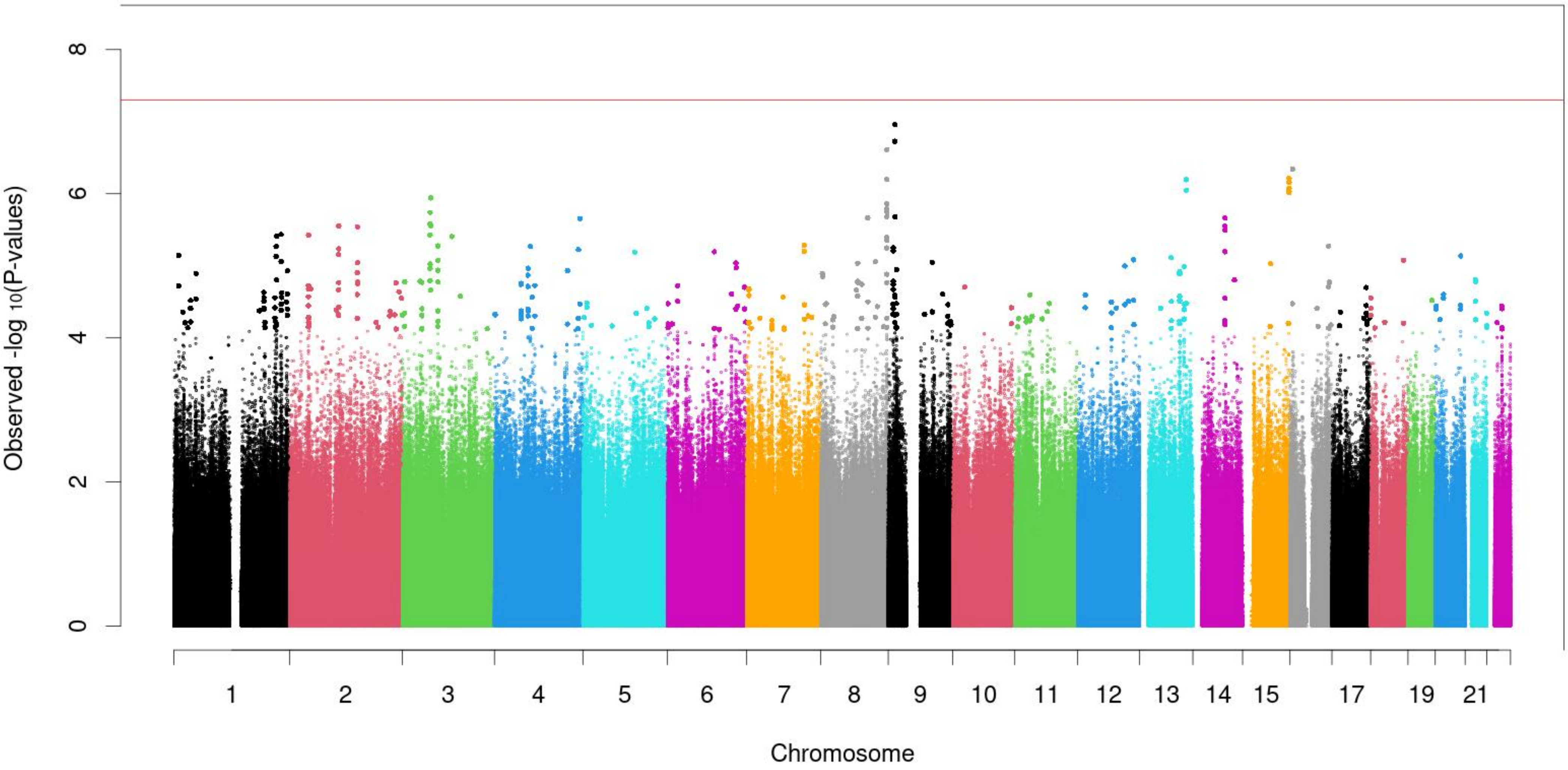

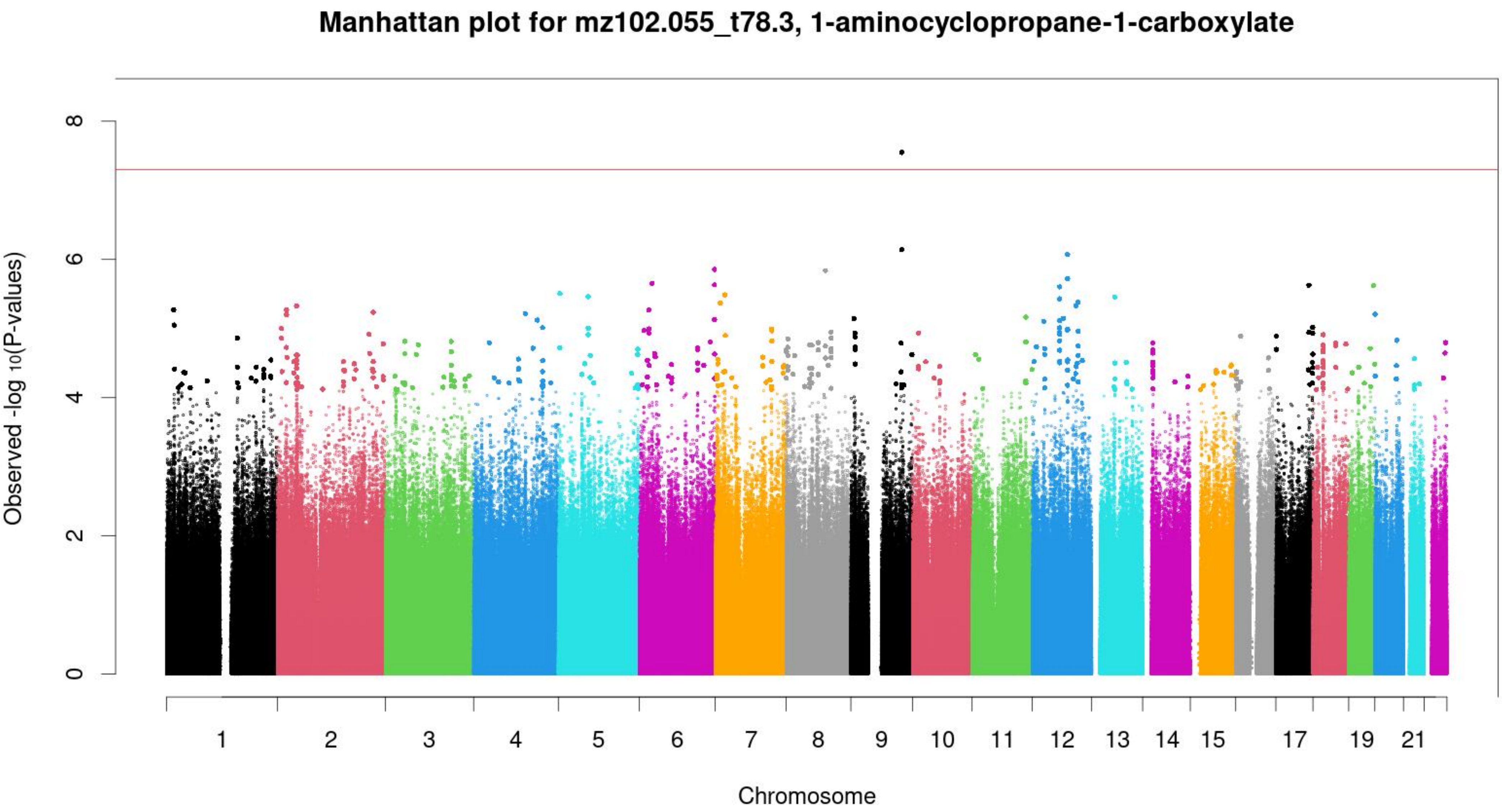
Manhattan plot for mz102.055_t78.3, 1-aminocyclopropane-1-carboxylate
Observed -log 10(P-values)
0
2
4
6
8
1
2
3
4
5
6
7
8
9
10
11
12
13
14
15
17
19
21
Chromosome

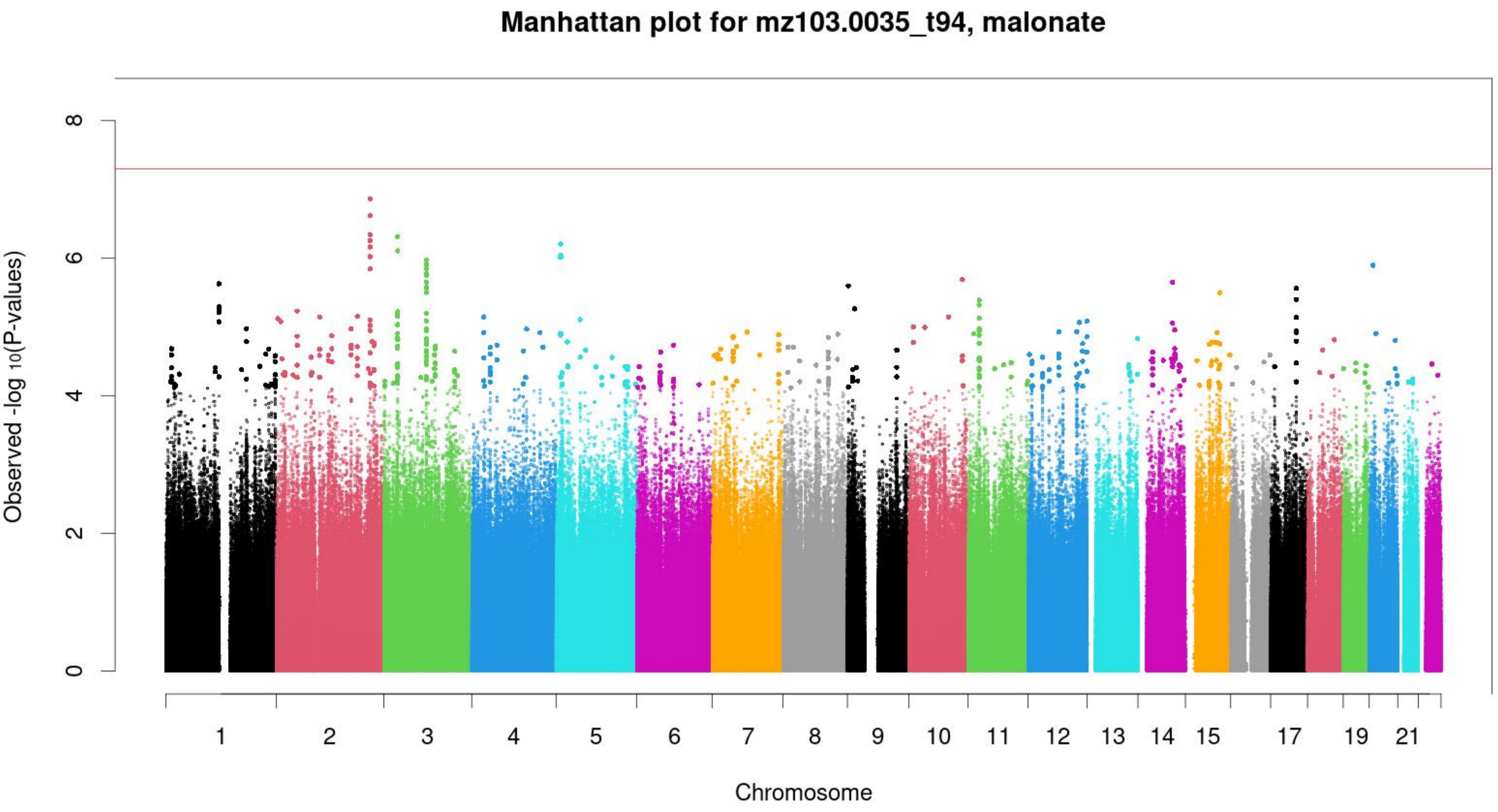
Manhattan plot for mz103.0035_t94, malonate
Observed -log 10(P-values)
0
2
4
6
8
1
2
3
4
5
6
7
8
9
10
11
12
13
14
15
17
19
21
Chromosome

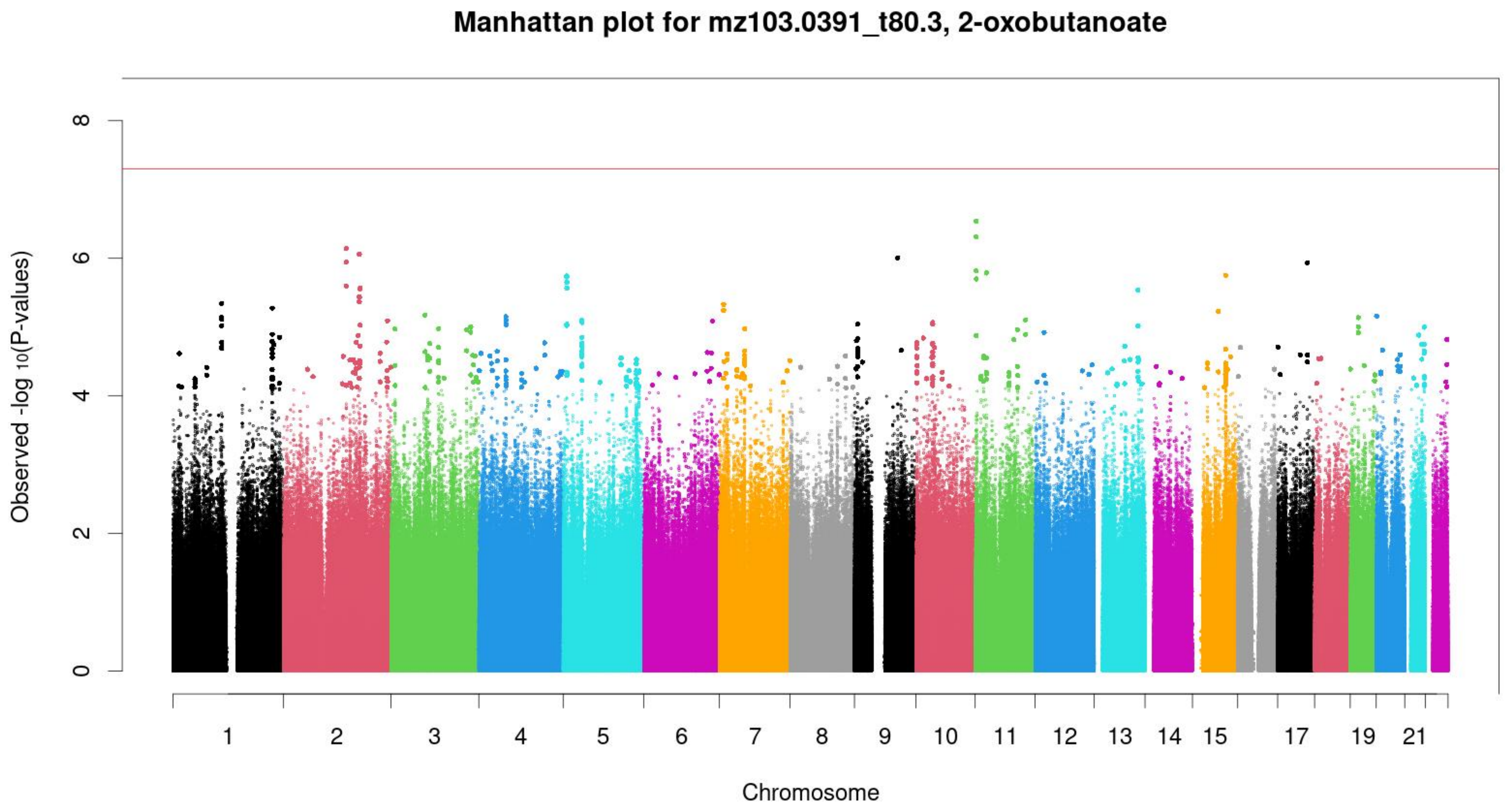

Manhattan plot for mz103.0391_t80.3, 2-oxobutanoate
Observed -log 10(P-values)
0
2
4
6
8
1
2
3
4
5
6
7
8
9
10
11
12
13
14
15
17
19
21
Chromosome

Manhattan plot for mz104.0351_t26.8, serine

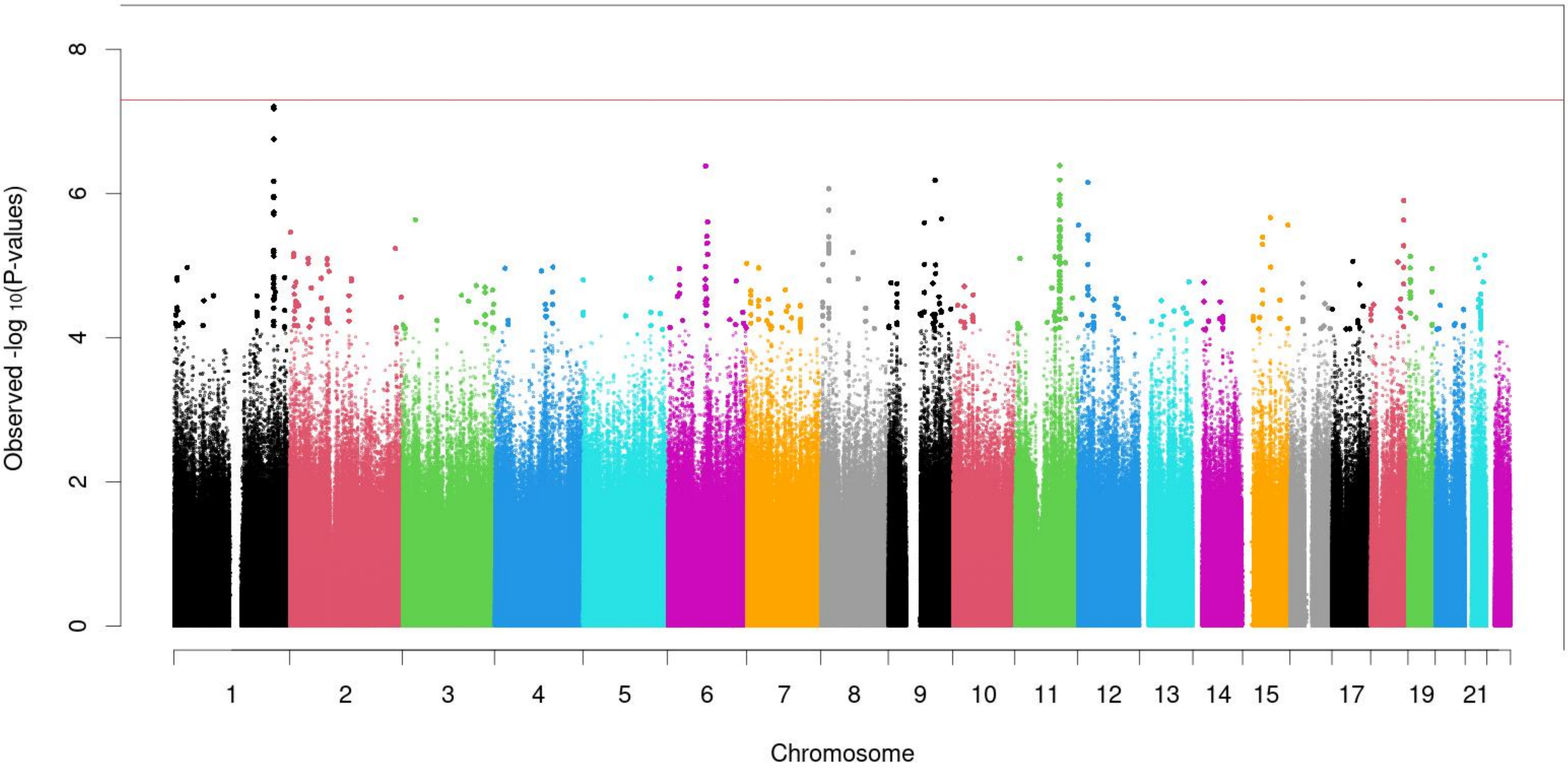

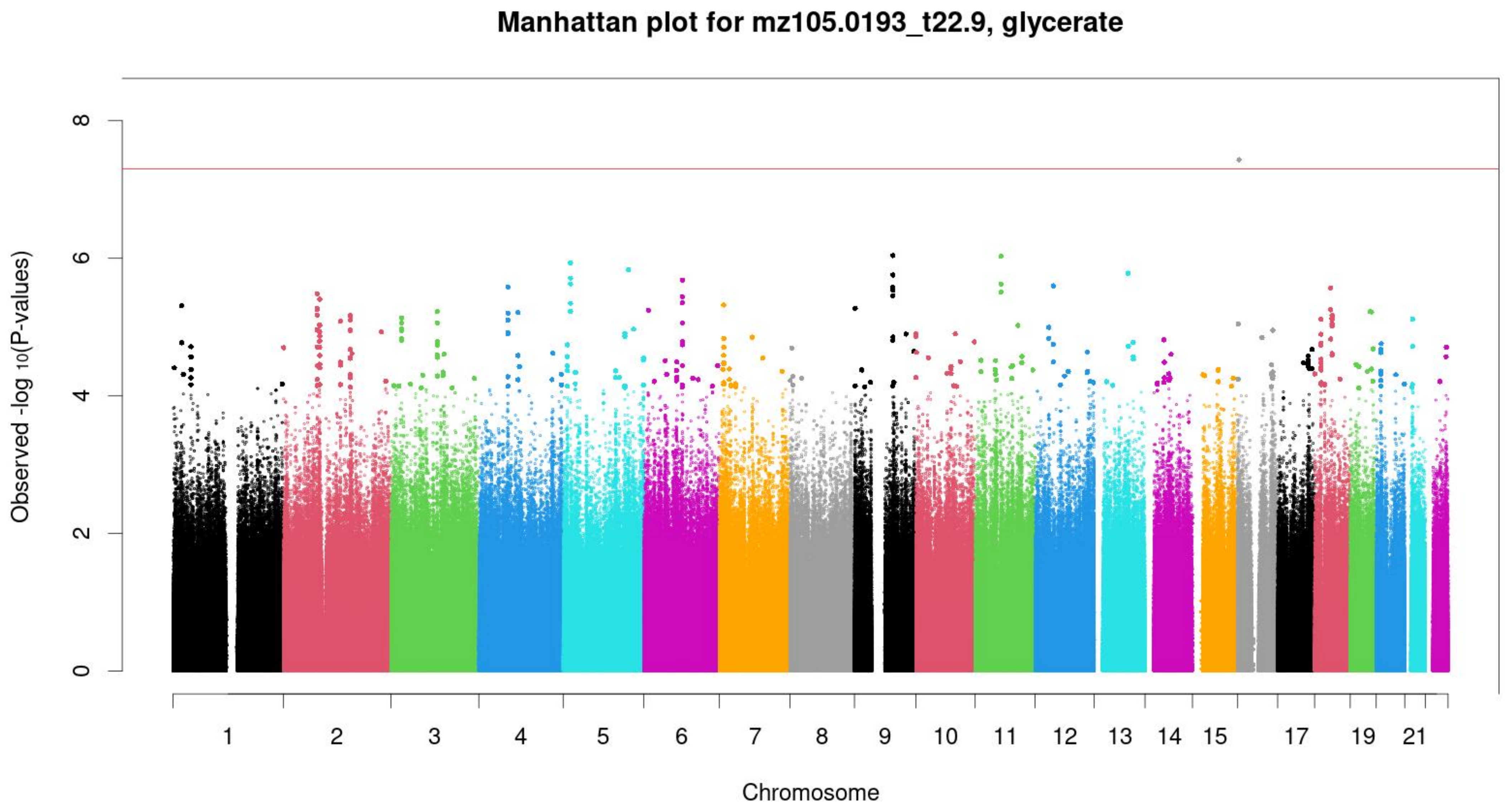
Manhattan plot for mz105.0193_t22.9, glycerate
Observed -log 10(P-values)
0
2
4
6
8
1
2
3
4
5
6
7
8
9
10
11
12
13
14
15
17
19
21
Chromosome

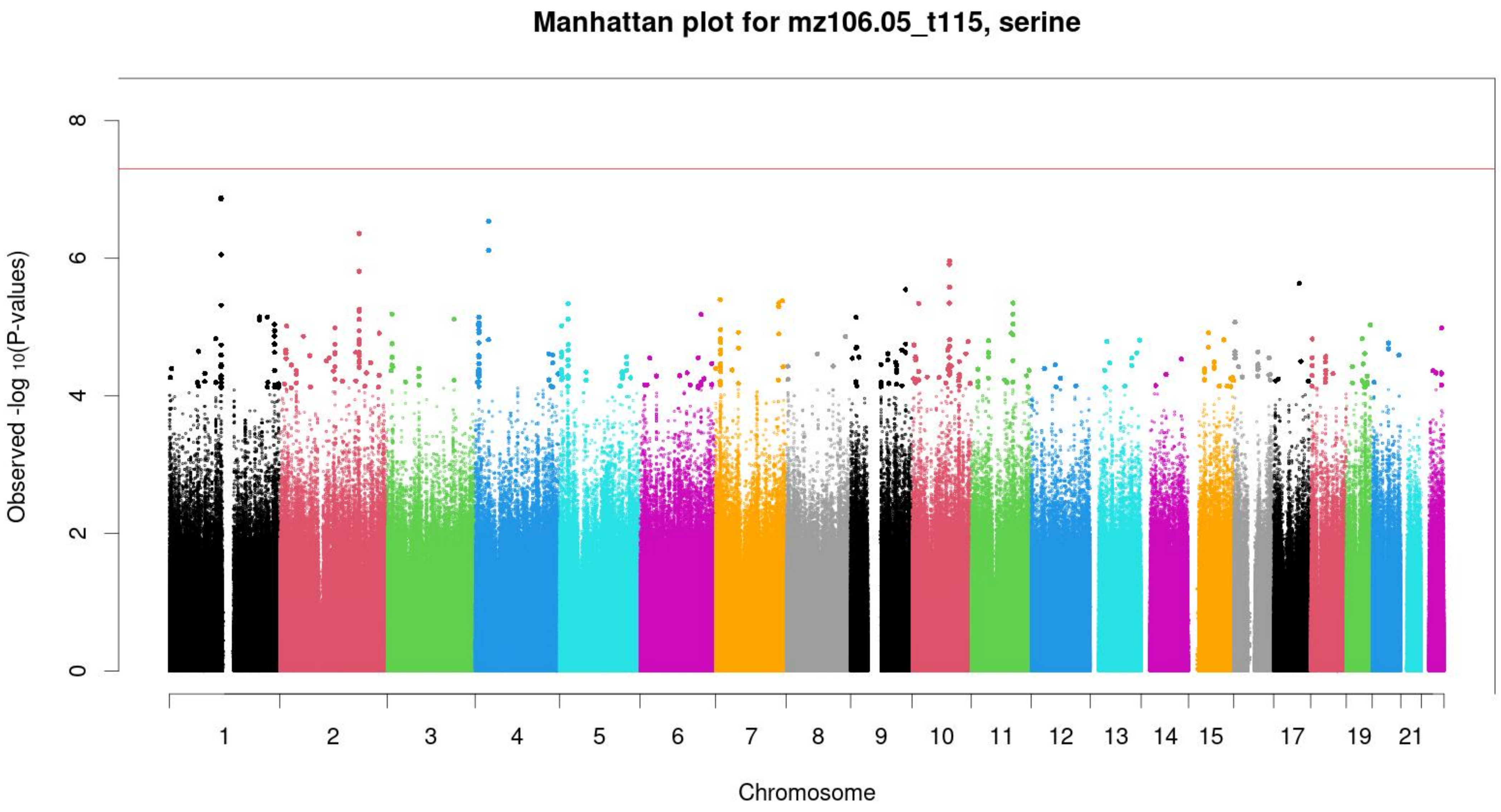

Manhattan plot for mz106.05_t115, serine
Observed -log 10(P-values)
0
2
4
6
8
1
2
3
4
5
6
7
8
9
10
11
12
13
14
15
17
19
21
Chromosome

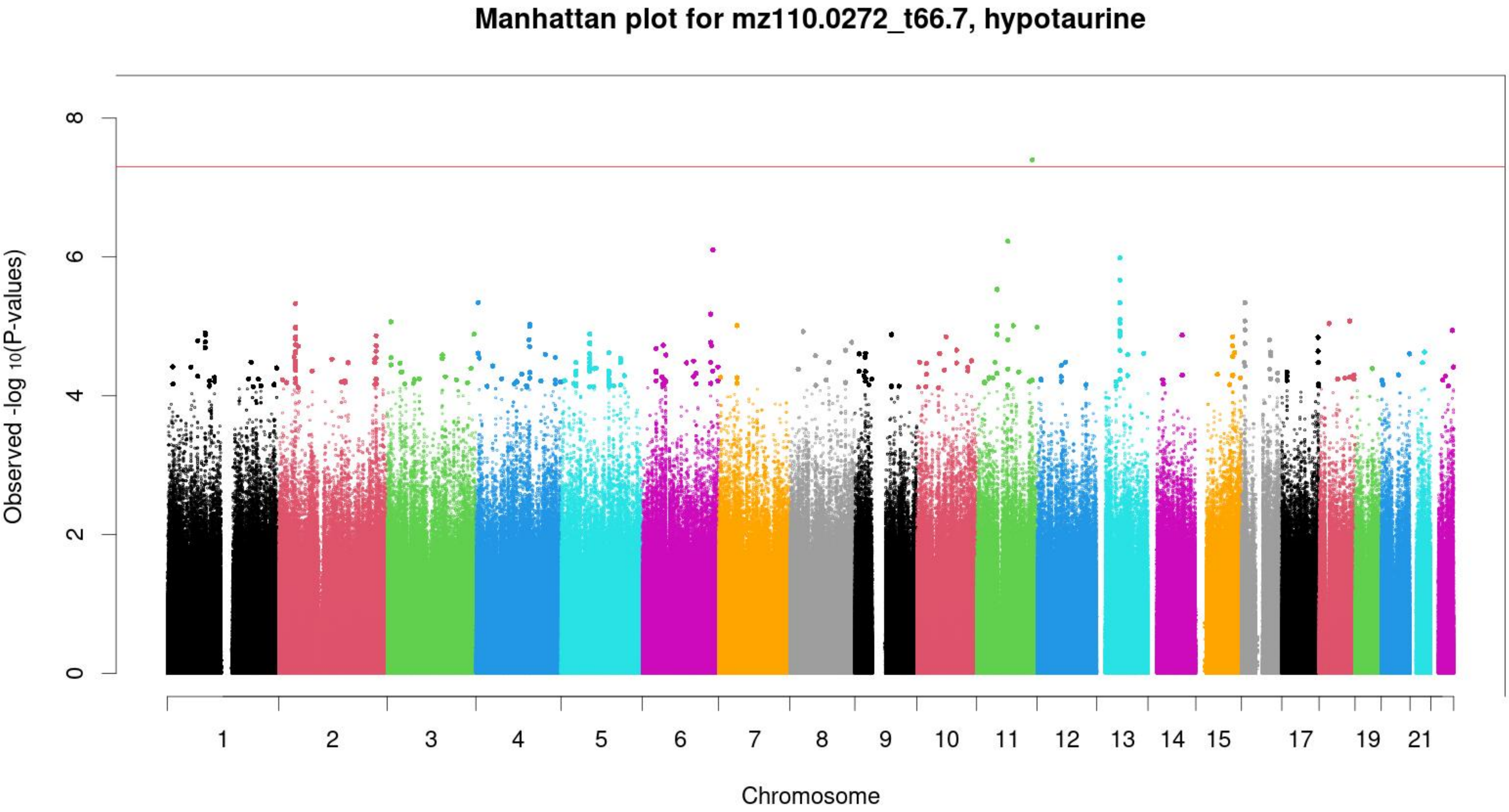

Manhattan plot for mz110.0272_t66.7, hypotaurine
Observed -log 10(P-values)
0
2
4
6
8
1
2
3
4
5
6
7
8
9
10
11
12
13
14
15
17
19
21
Chromosome

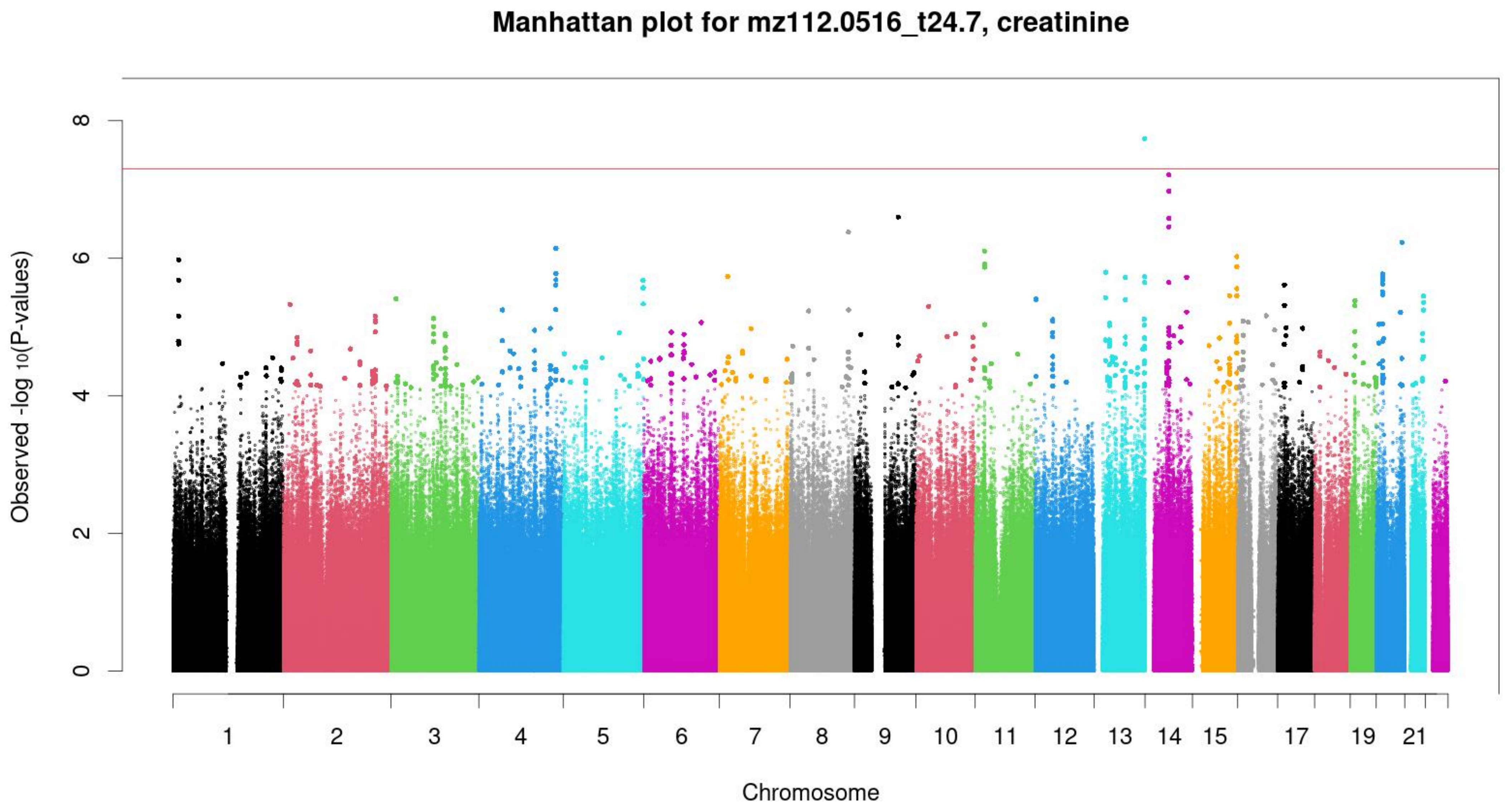

Manhattan plot for mz112.0516_t24.7, creatinine
Observed -log 10(P-values)
0
2
4
6
8
1
2
3
4
5
6
7
8
9
10
11
12
13
14
15
17
19
21
Chromosome

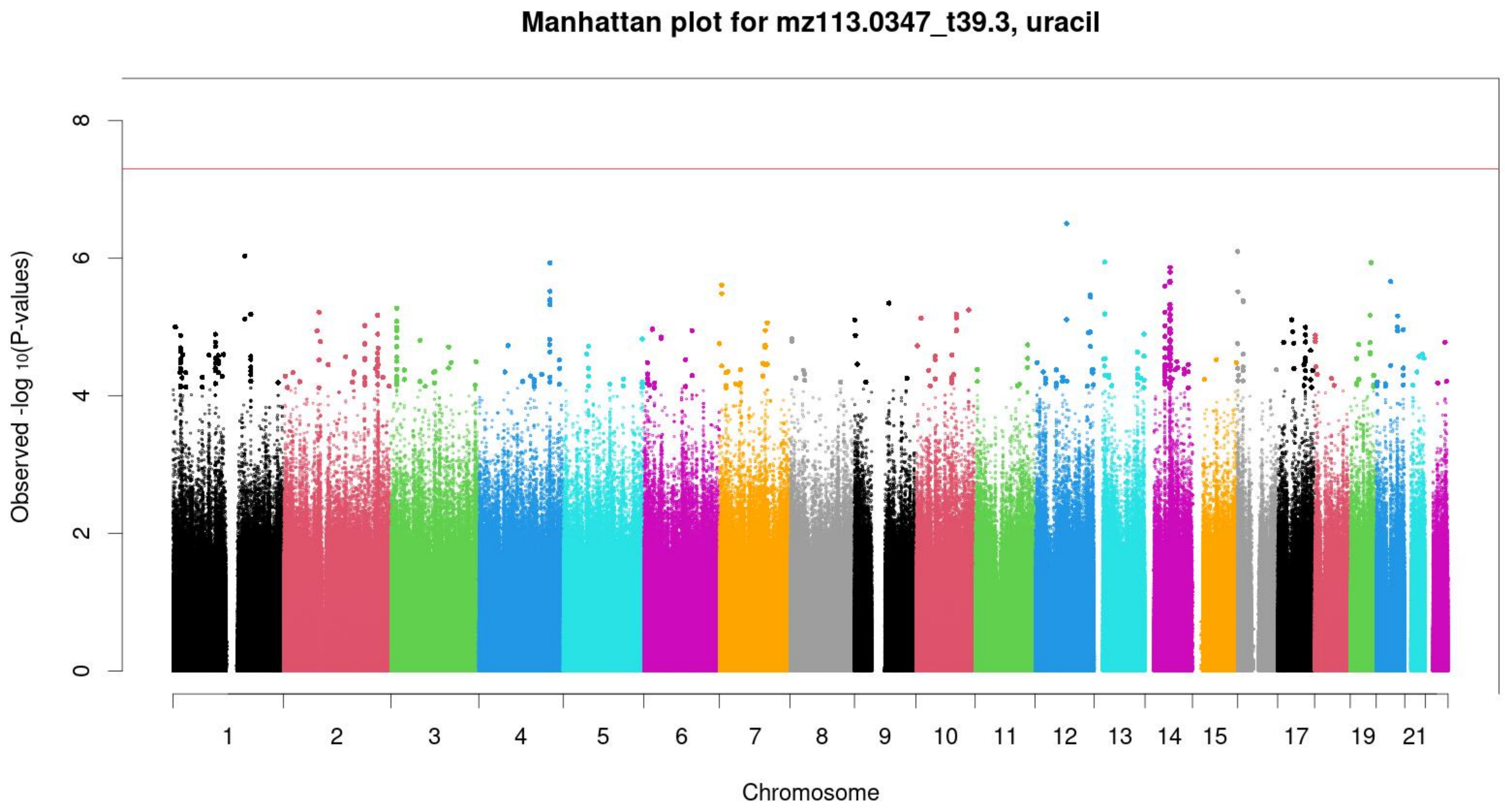

Manhattan plot for mz113.0347_t39.3, uracil
Observed -log 10(P-values)
0
2
4
6
8
1
2
3
4
5
6
7
8
9
10
11
12
13
14
15
17
19
21
Chromosome

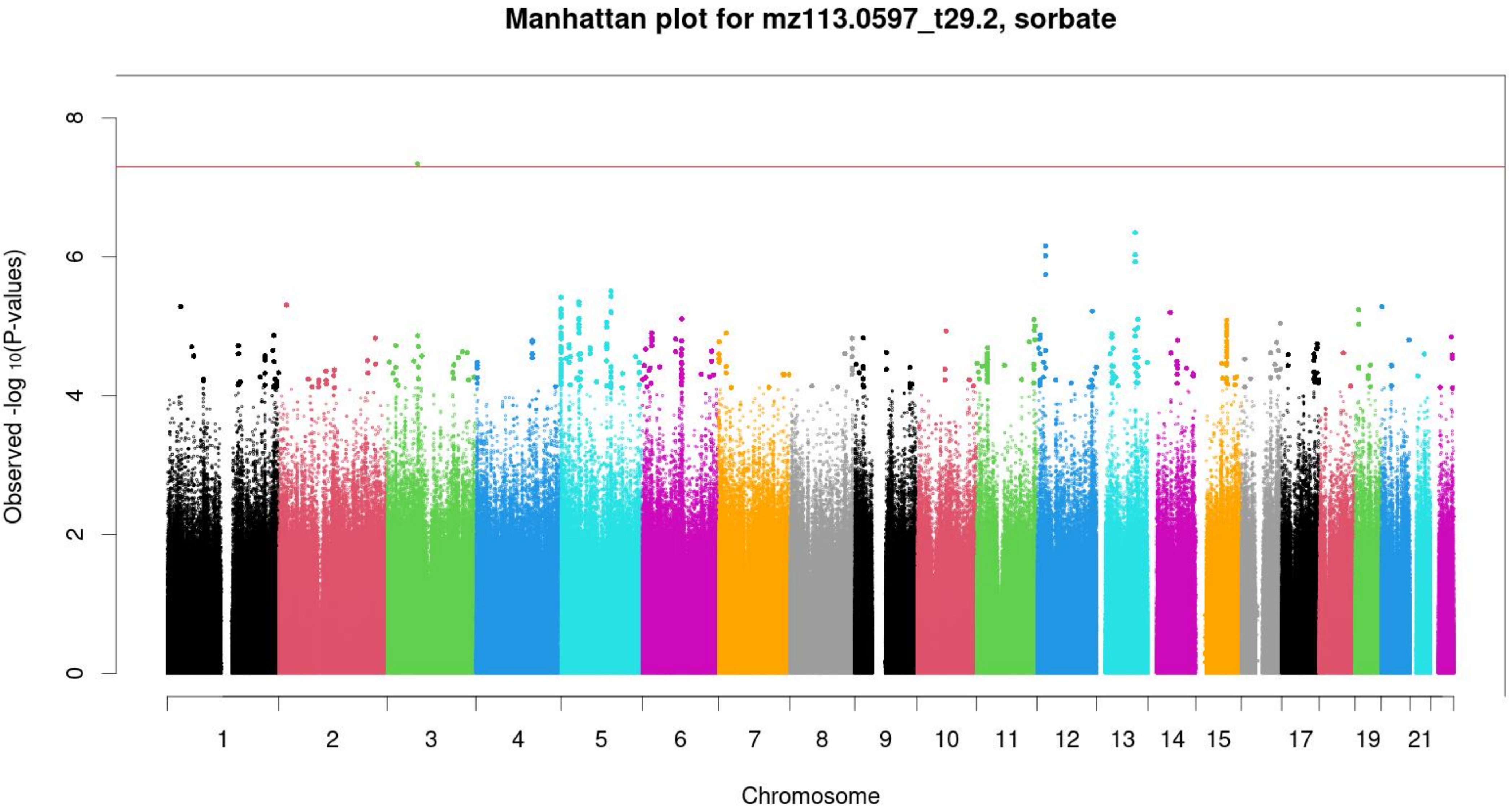
Manhattan plot for mz113.0597_t29.2, sorbate
Observed -log 10(P-values)
0
2
4
6
8
1
2
3
4
5
6
7
8
9
10
11
12
13
14
15
17
19
21
Chromosome

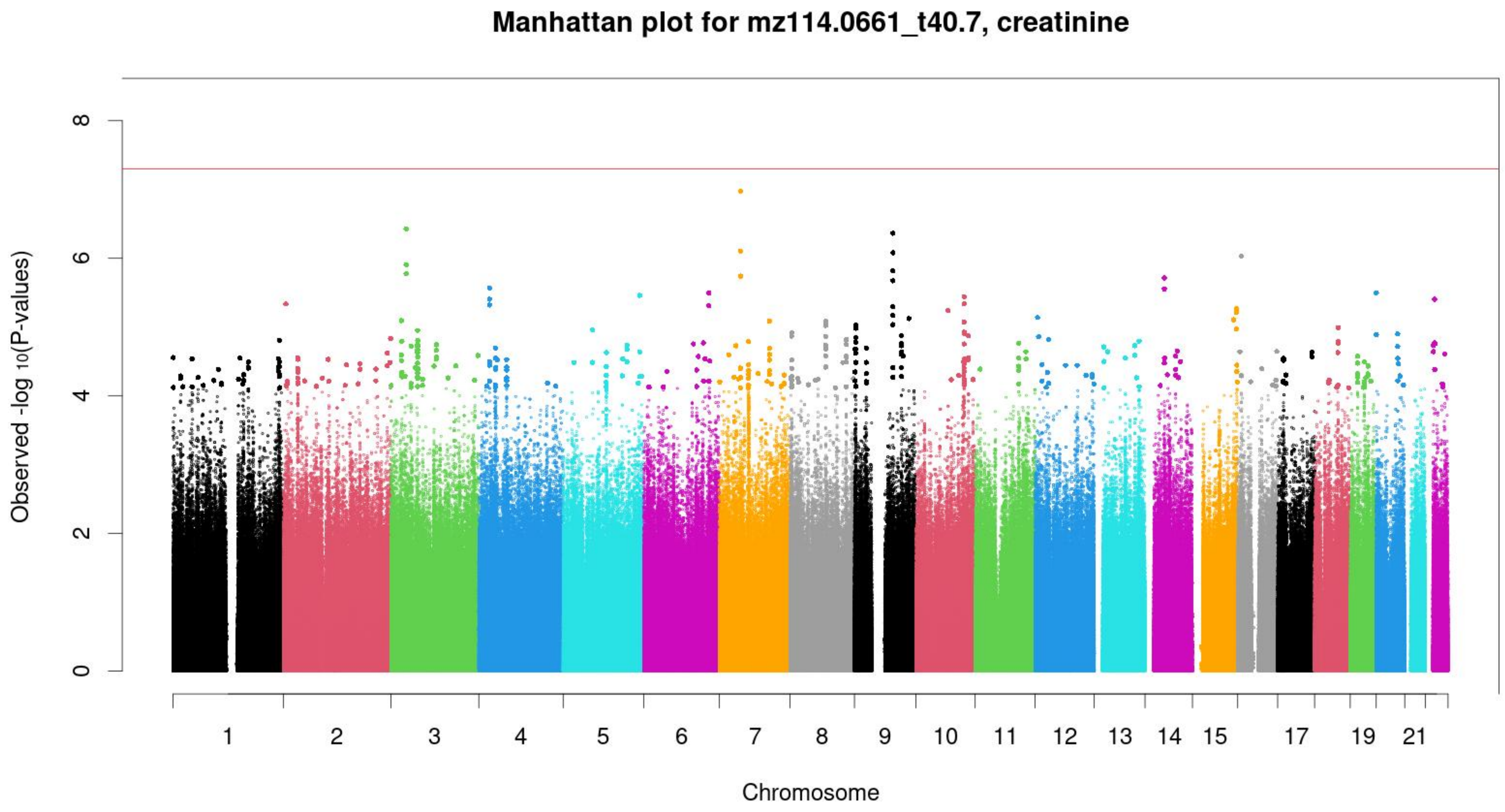

Manhattan plot for mz114.0661_t40.7, creatinine
Observed -log 10(P-values)
0
2
4
6
8
1
2
3
4
5
6
7
8
9
10
11
12
13
14
15
17
19
21
Chromosome

Manhattan plot for mz116.0343_t85.6, maleamate

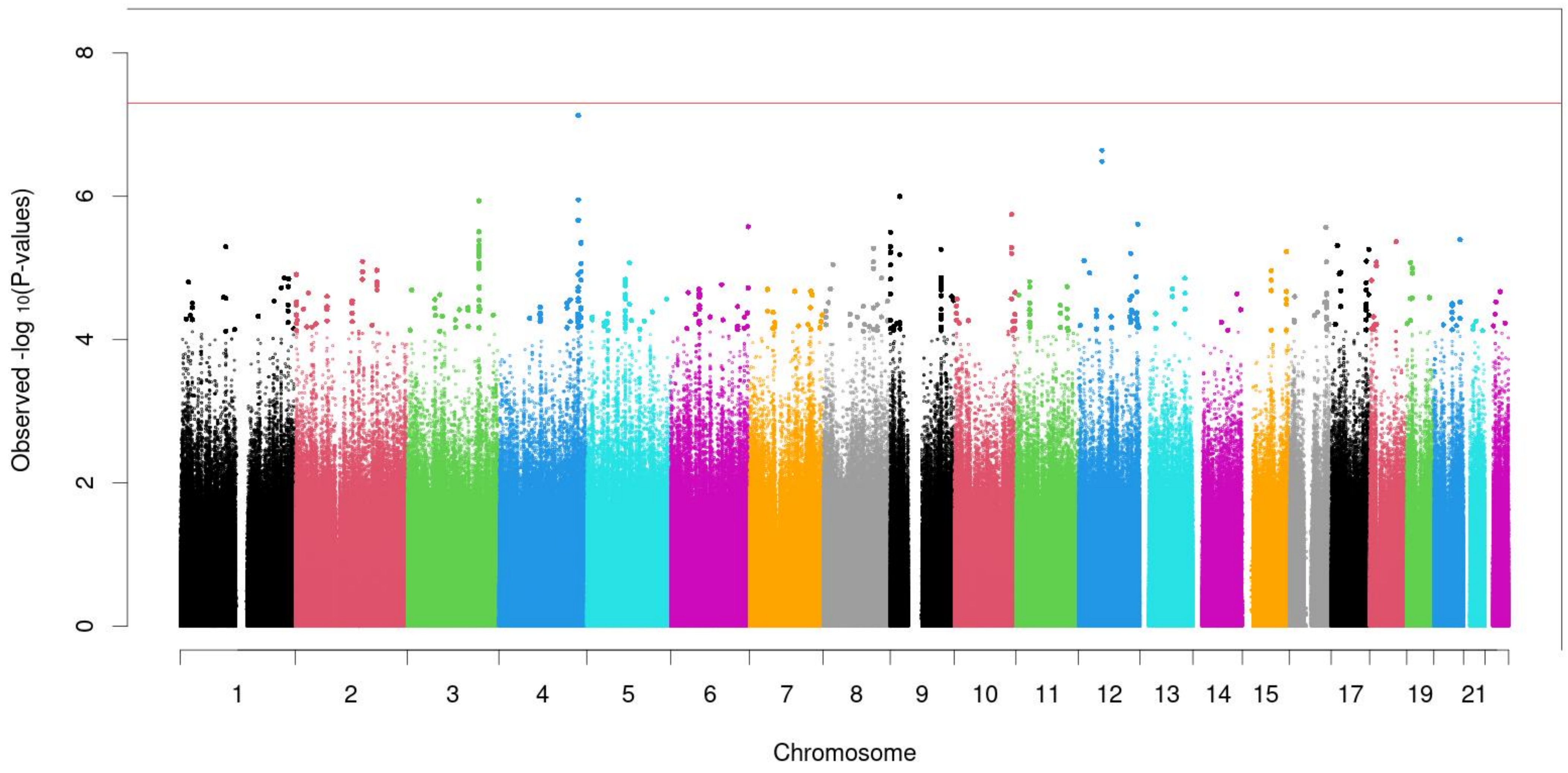

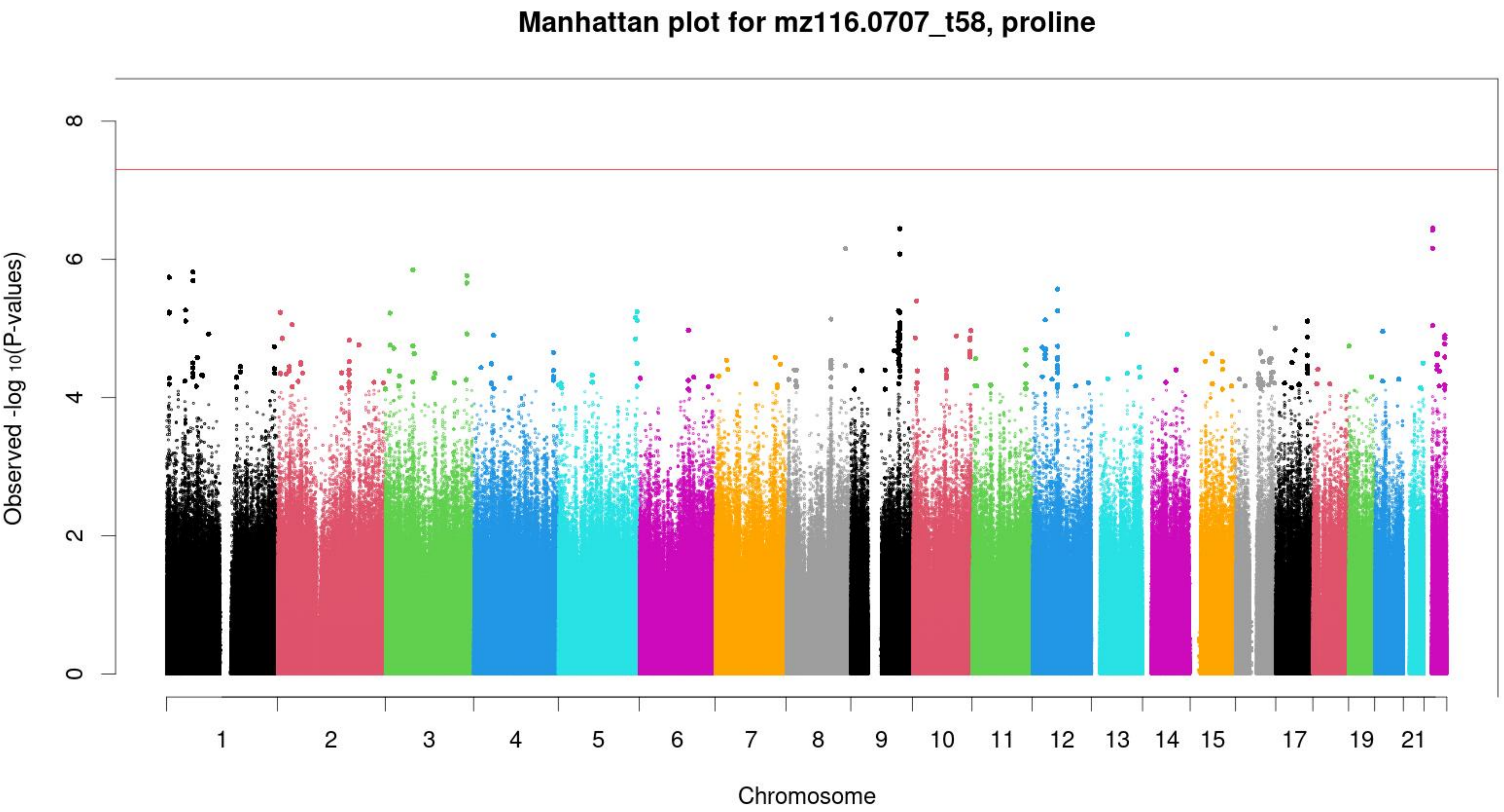

Manhattan plot for mz116.0707_t58, proline
Observed -log 10(P-values)
0
2
4
6
8
Chromosome
1
2
3
4
5
6
7
8
9
10
11
12
13
14
15
17
19
21

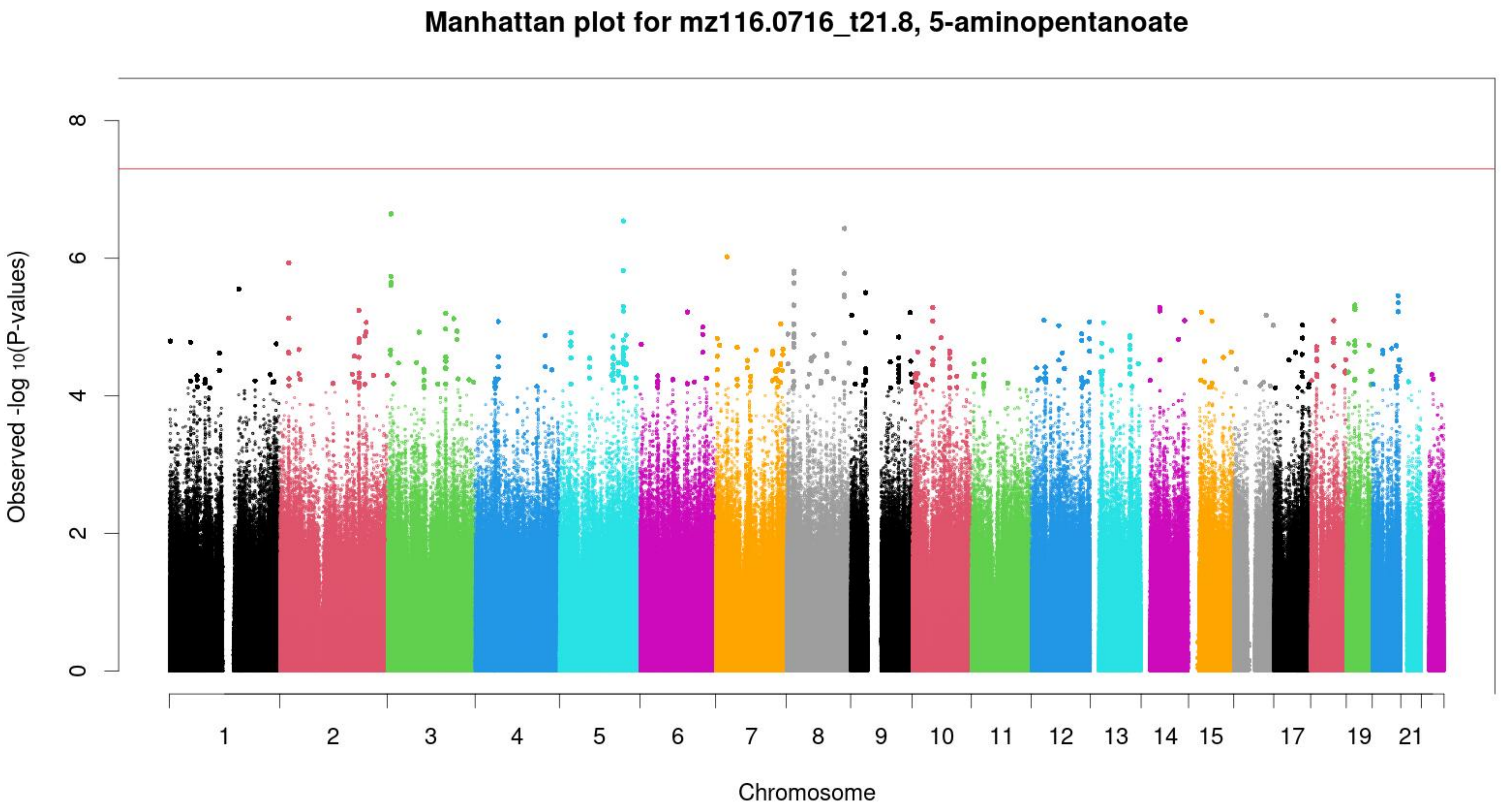
Manhattan plot for mz116.0716_t21.8, 5-aminopentanoate
Observed -log 10(P-values)
0
2
4
6
8
Chromosome
1
2
3
4
5
6
7
8
9
10
11
12
13
14
15
17
19
21

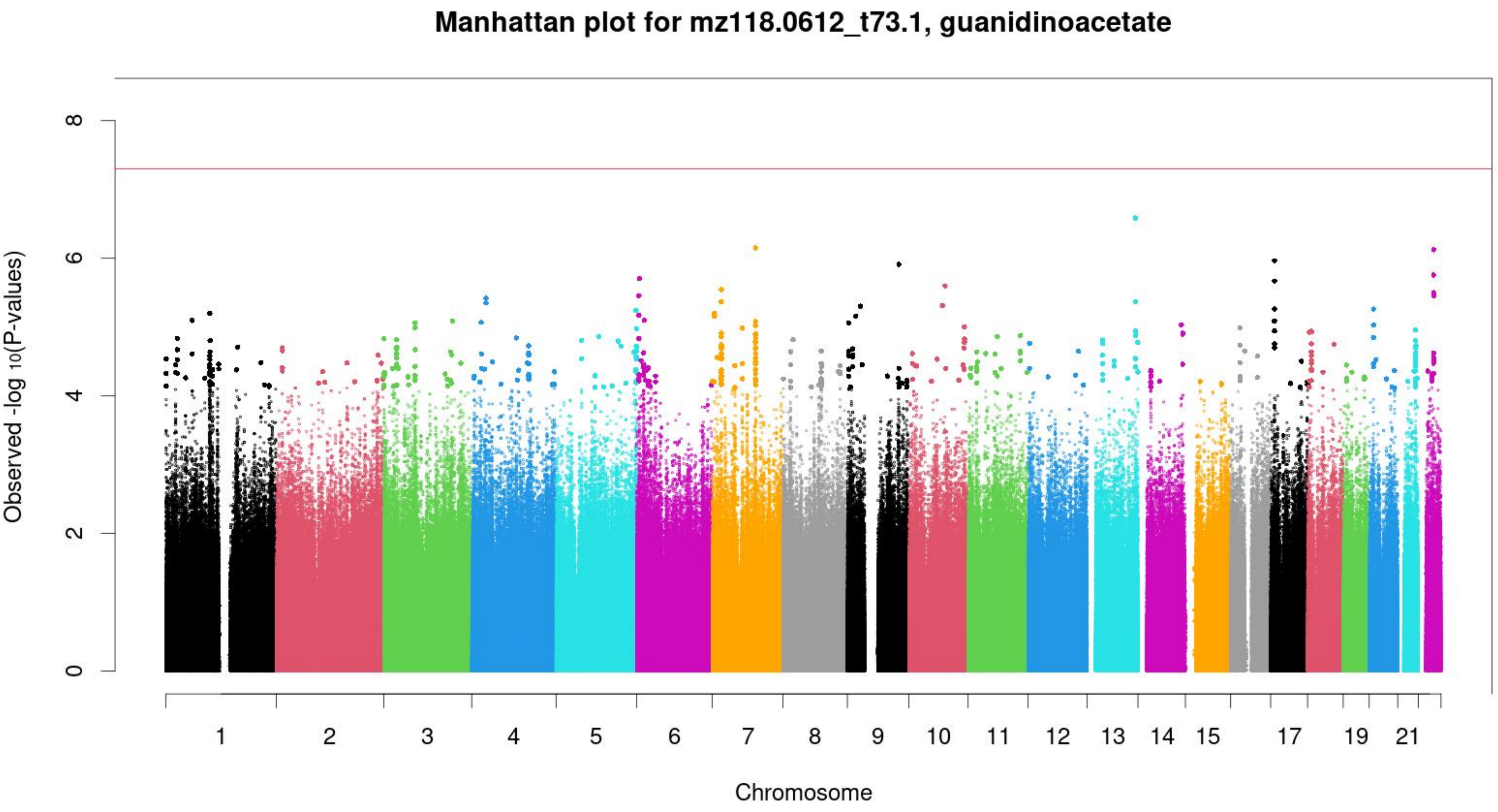

Manhattan plot for mz118.0612_t73.1, guanidinoacetate
Observed -log 10(P-values)
0
2
4
6
8
1
2
3
4
5
6
7
8
9
10
11
12
13
14
15
17
19
21
Chromosome

Manhattan plot for mz123.045_t27.4, 3-hydroxybenzyl alcohol

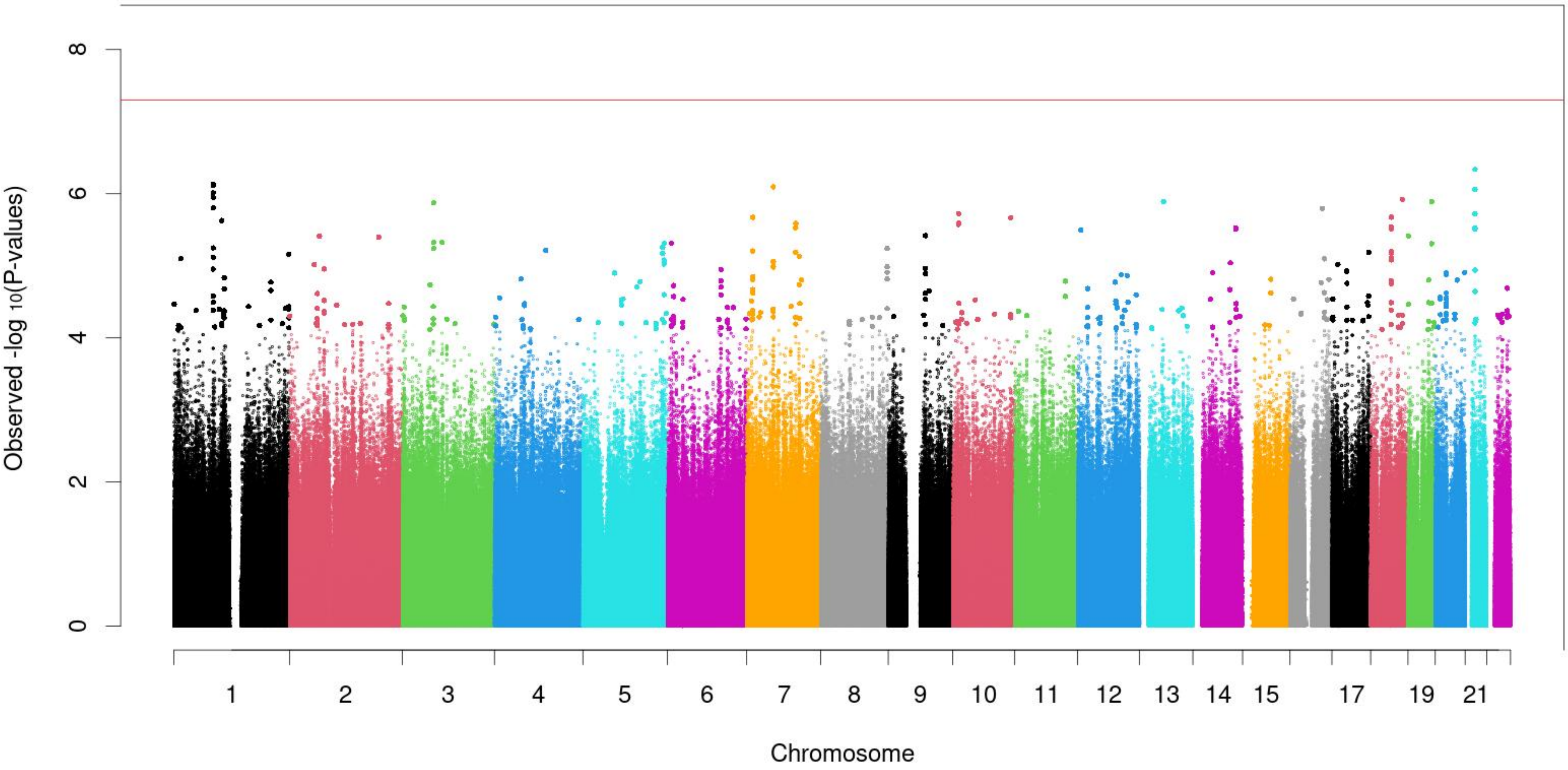

Manhattan plot for mz123.0441_t56.2, 3-hydroxybenzaldehyde

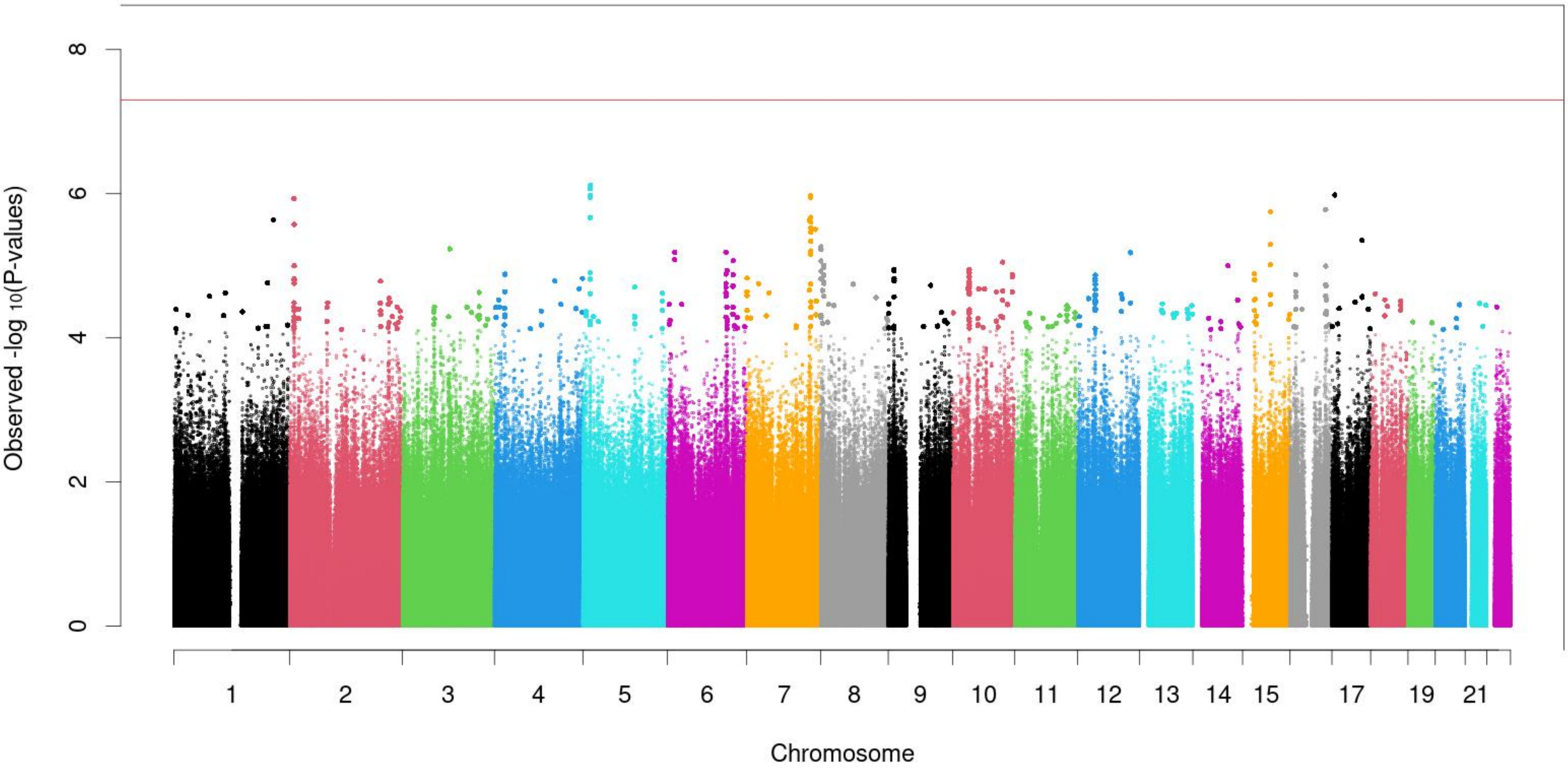

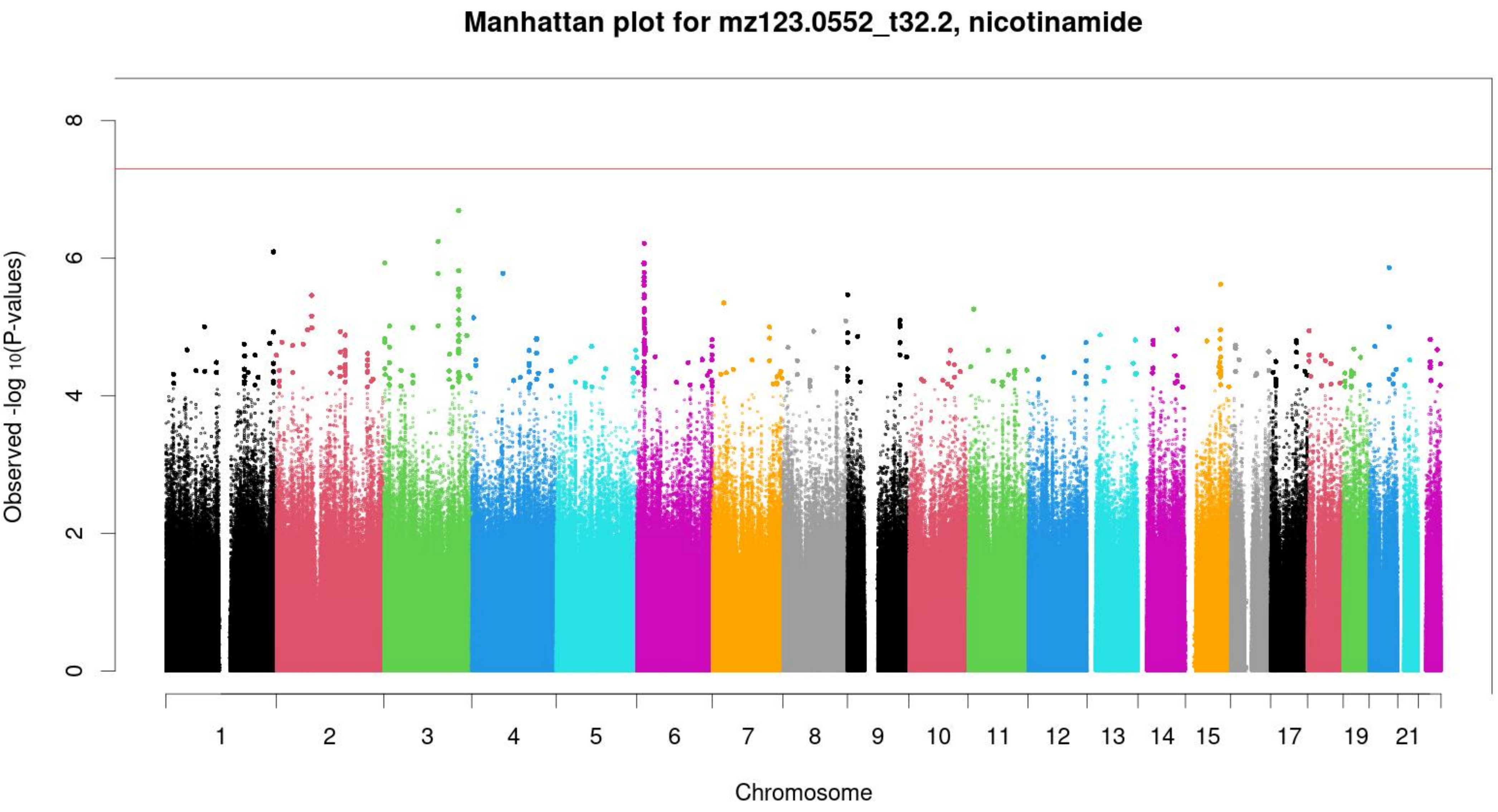

Manhattan plot for mz123.0552_t32.2, nicotinamide
Observed -log 10(P-values)
0
2
4
6
8
1
2
3
4
5
6
7
8
9
10
11
12
13
14
15
17
19
21
Chromosome

Manhattan plot for mz126.022_t55.6, taurine

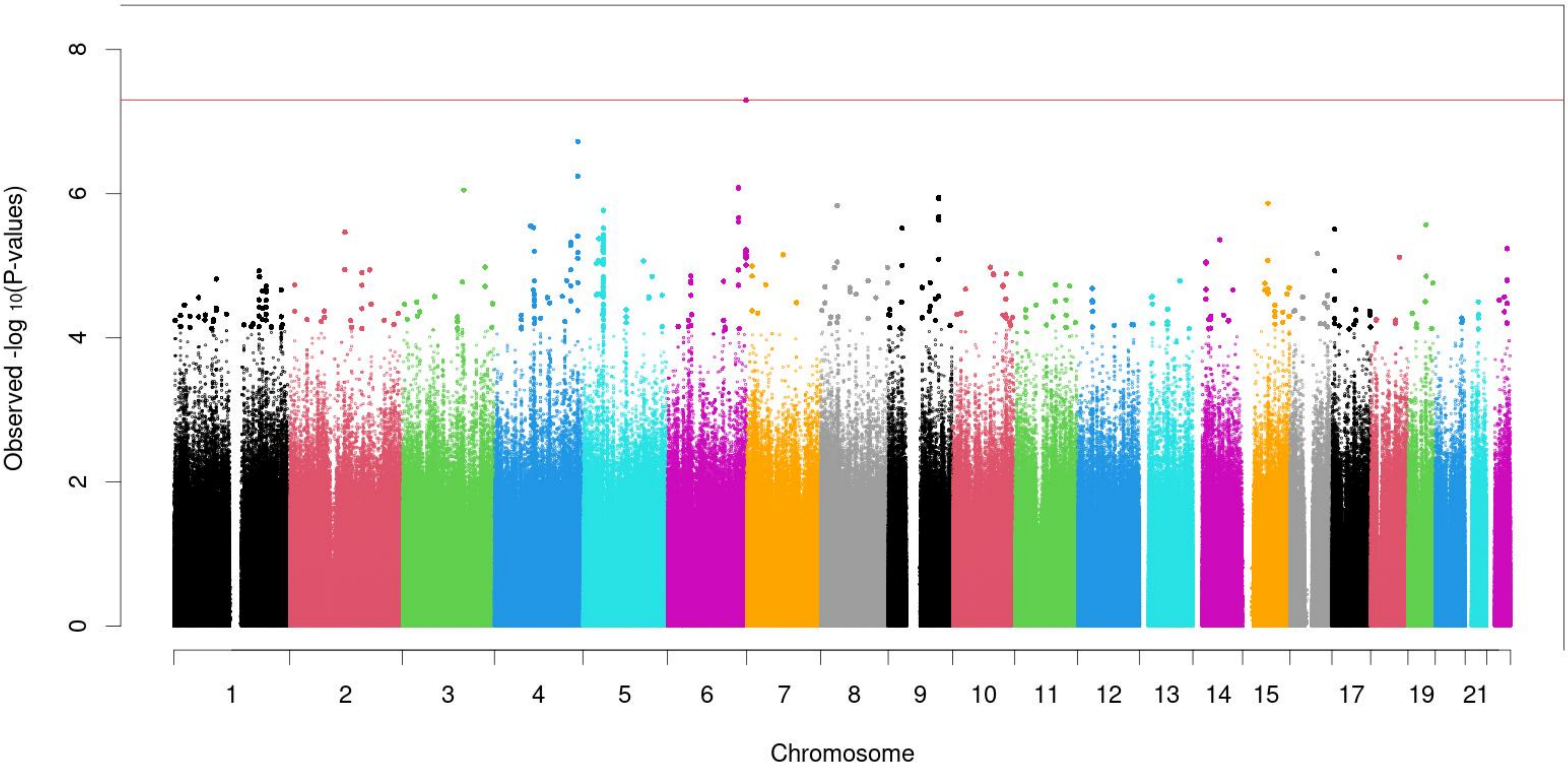

Manhattan plot for mz127.0503_t70.4, 4-imidazoleacetic acid

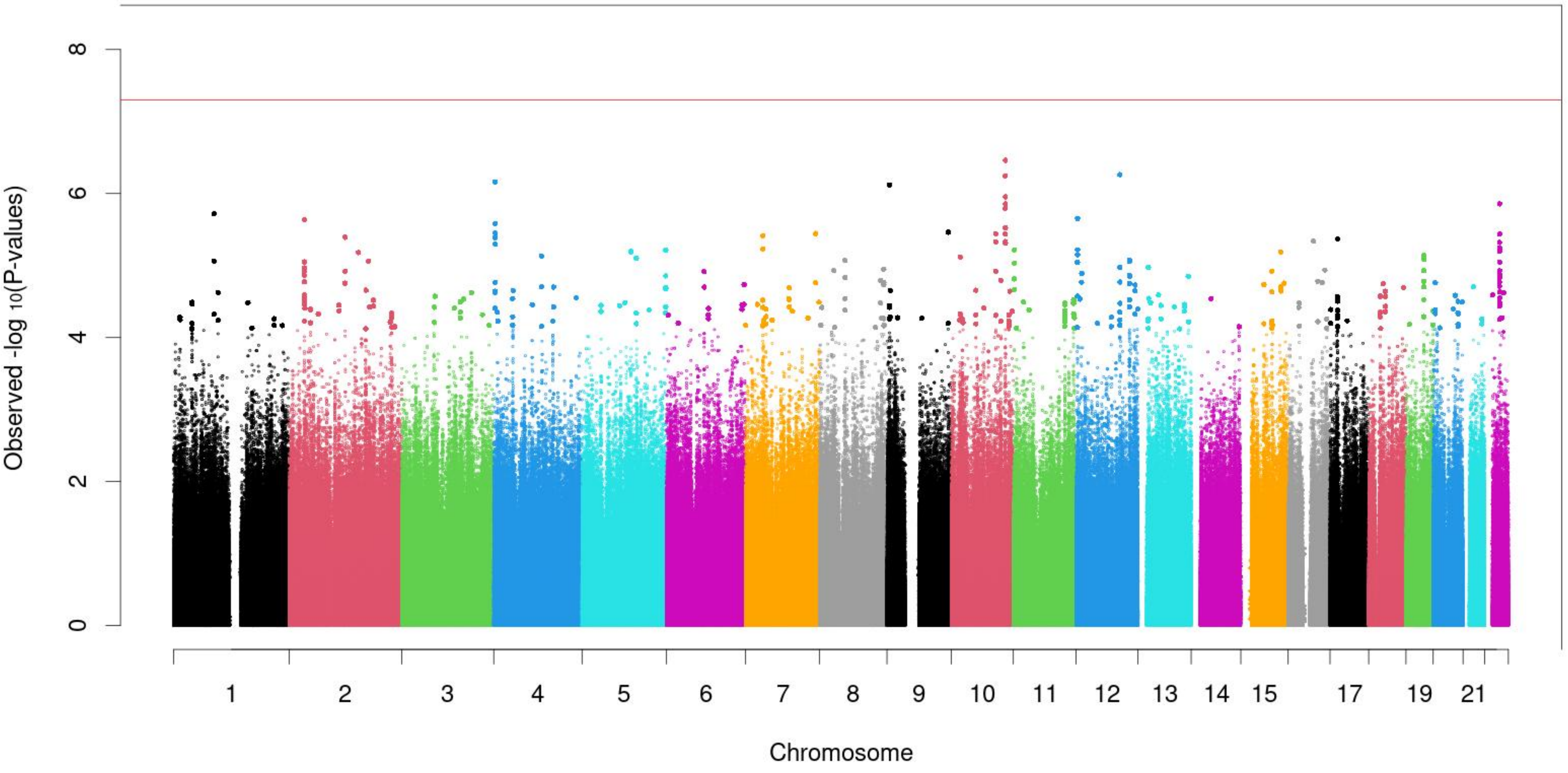

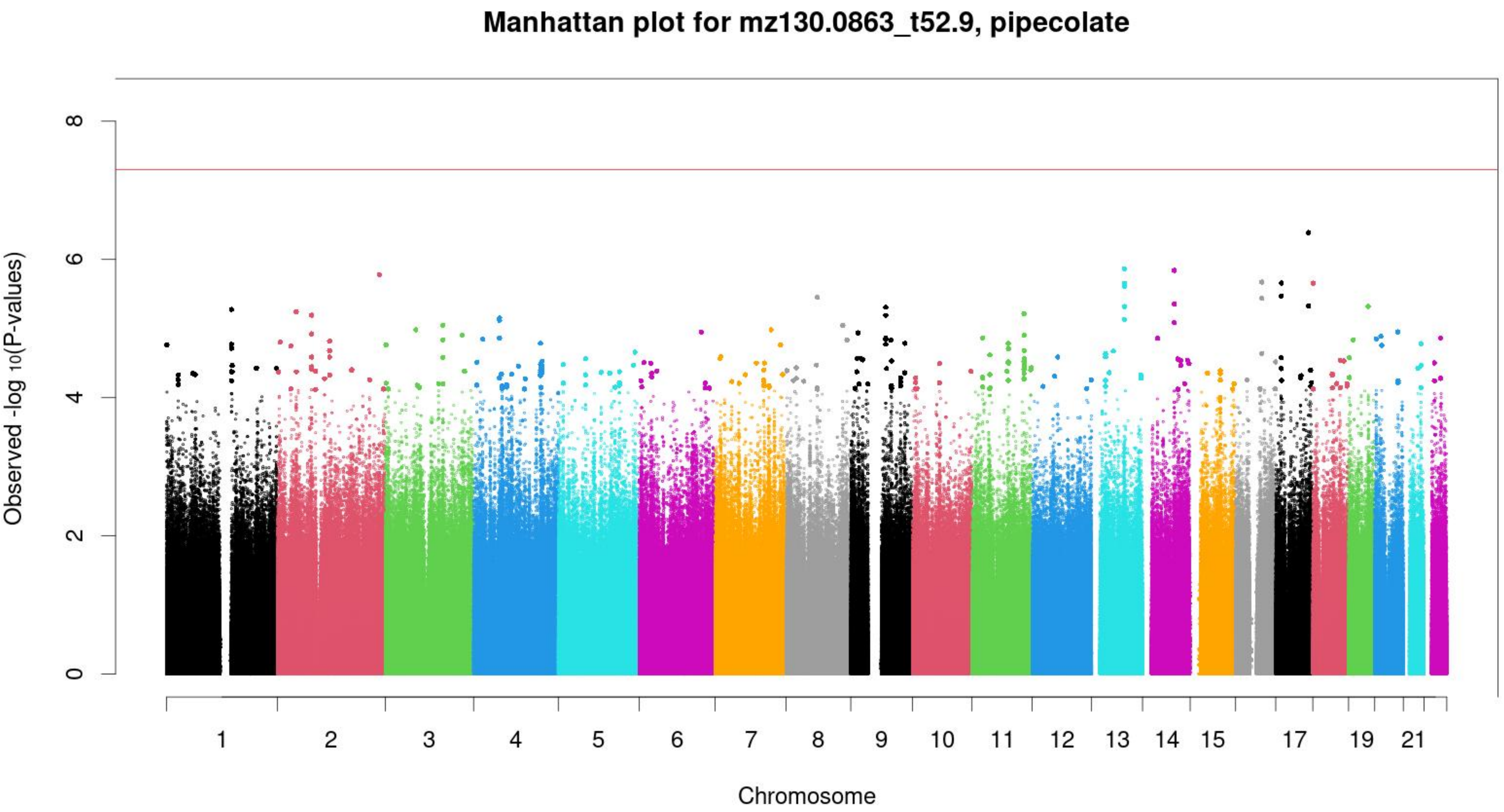

Manhattan plot for mz130.0863_t52.9, pipecolate
Observed -log 10(P-values)
0
2
4
6
8
1
2
3
4
5
6
7
8
9
10
11
12
13
14
15
17
19
21
Chromosome

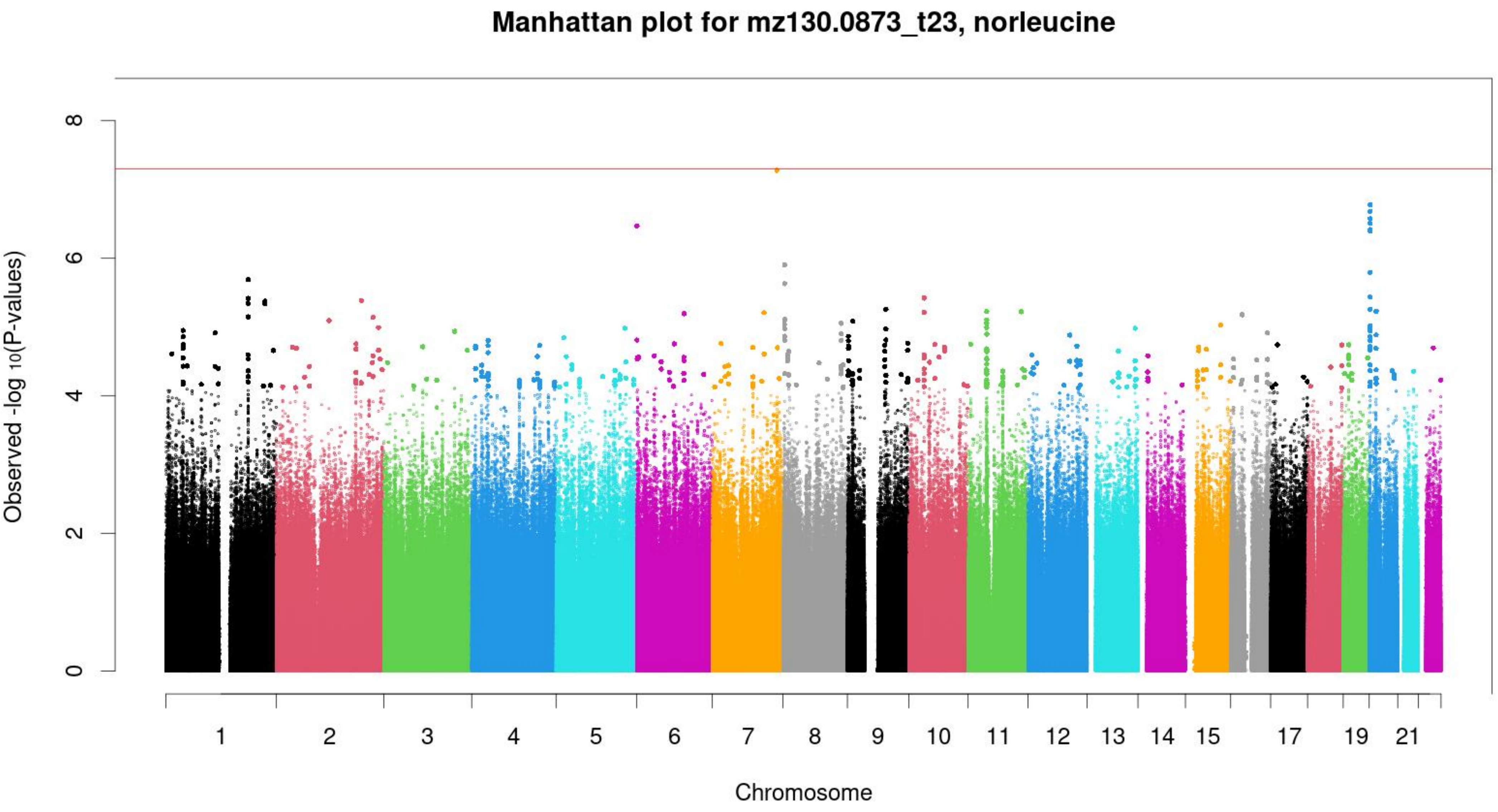

Manhattan plot for mz130.0873_t23, norleucine
Observed -log 10(P-values)
0
2
4
6
8
1
2
3
4
5
6
7
8
9
10
11
12
13
14
15
17
19
21
Chromosome

Manhattan plot for mz131.0459_t20.7, 3-ureidopropionate

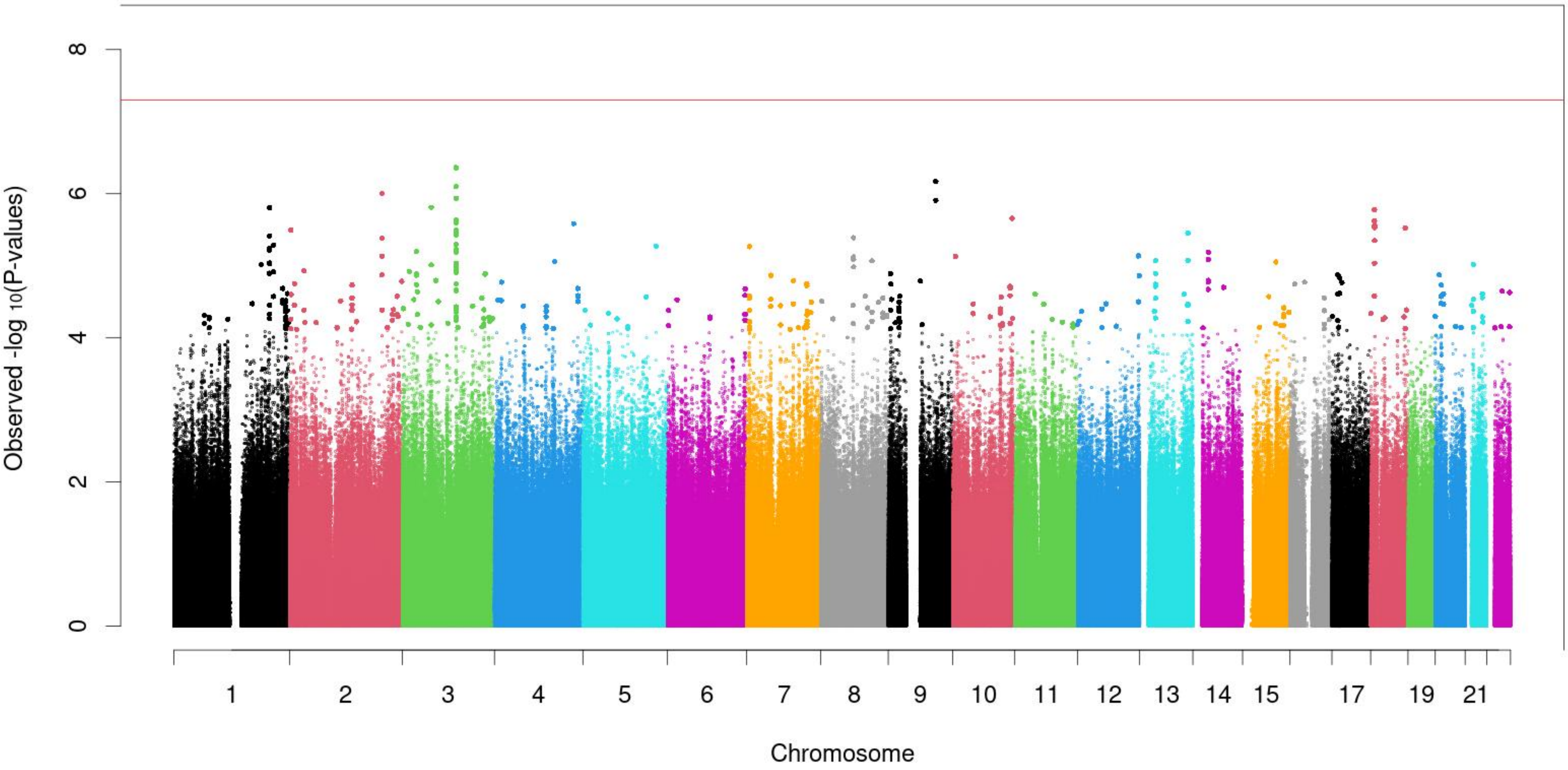

Manhattan plot for mz132.0765_t72.5, creatine

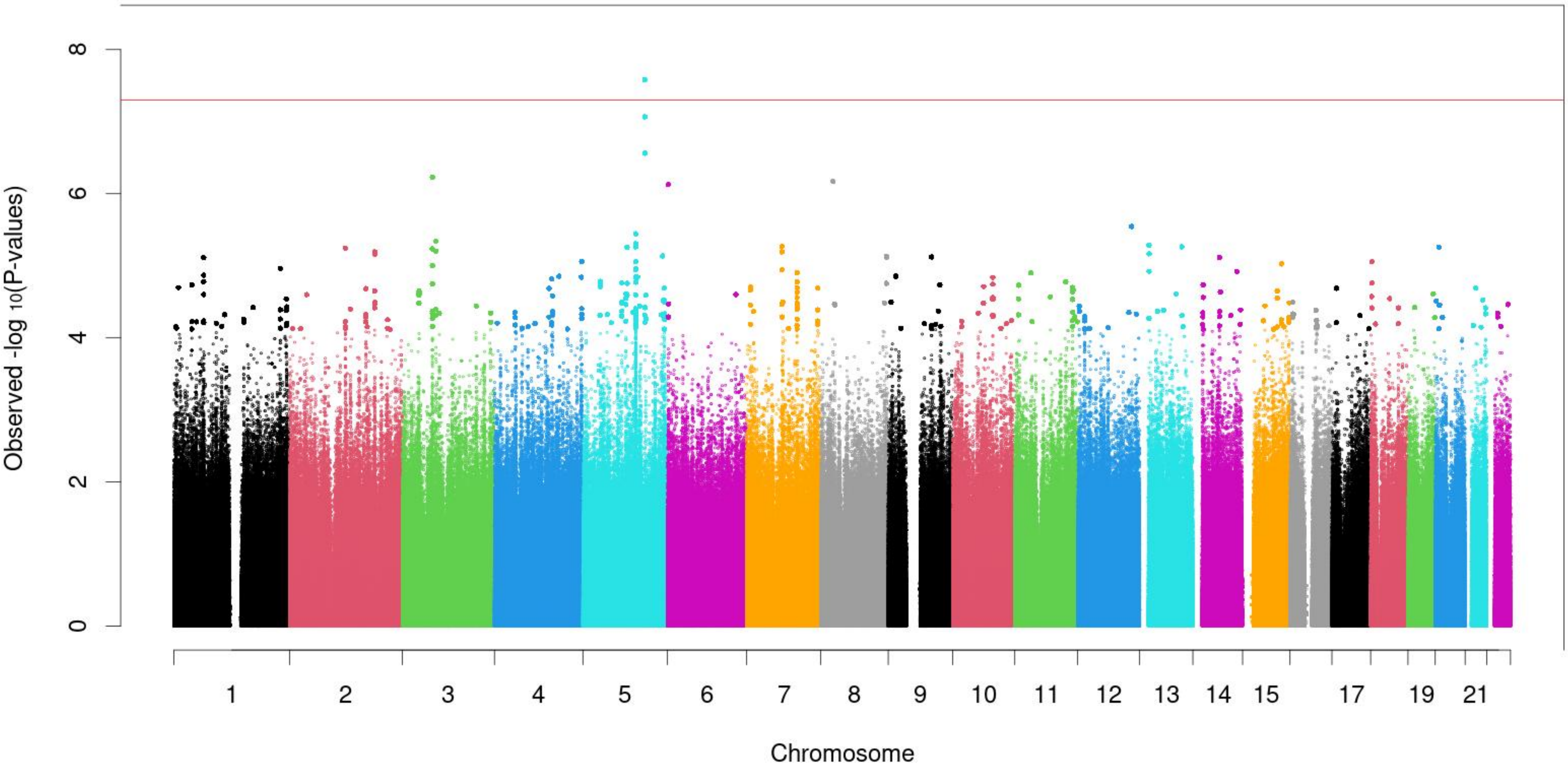

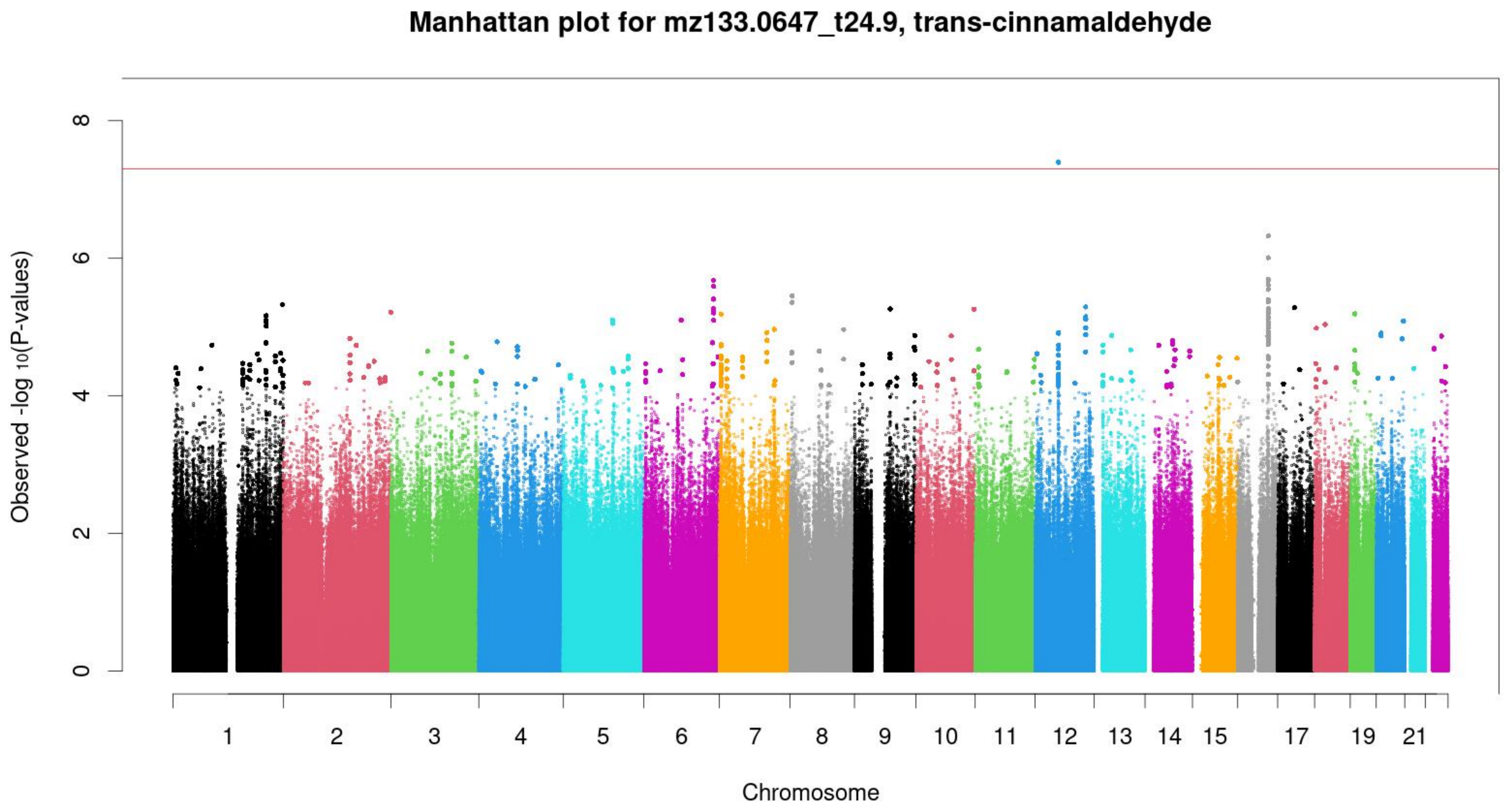

Manhattan plot for mz133.0647_t24.9, trans-cinnamaldehyde
Observed -log 10(P-values)
0
2
4
6
8
1
2
3
4
5
6
7
8
9
10
11
12
13
14
15
17
19
21
Chromosome

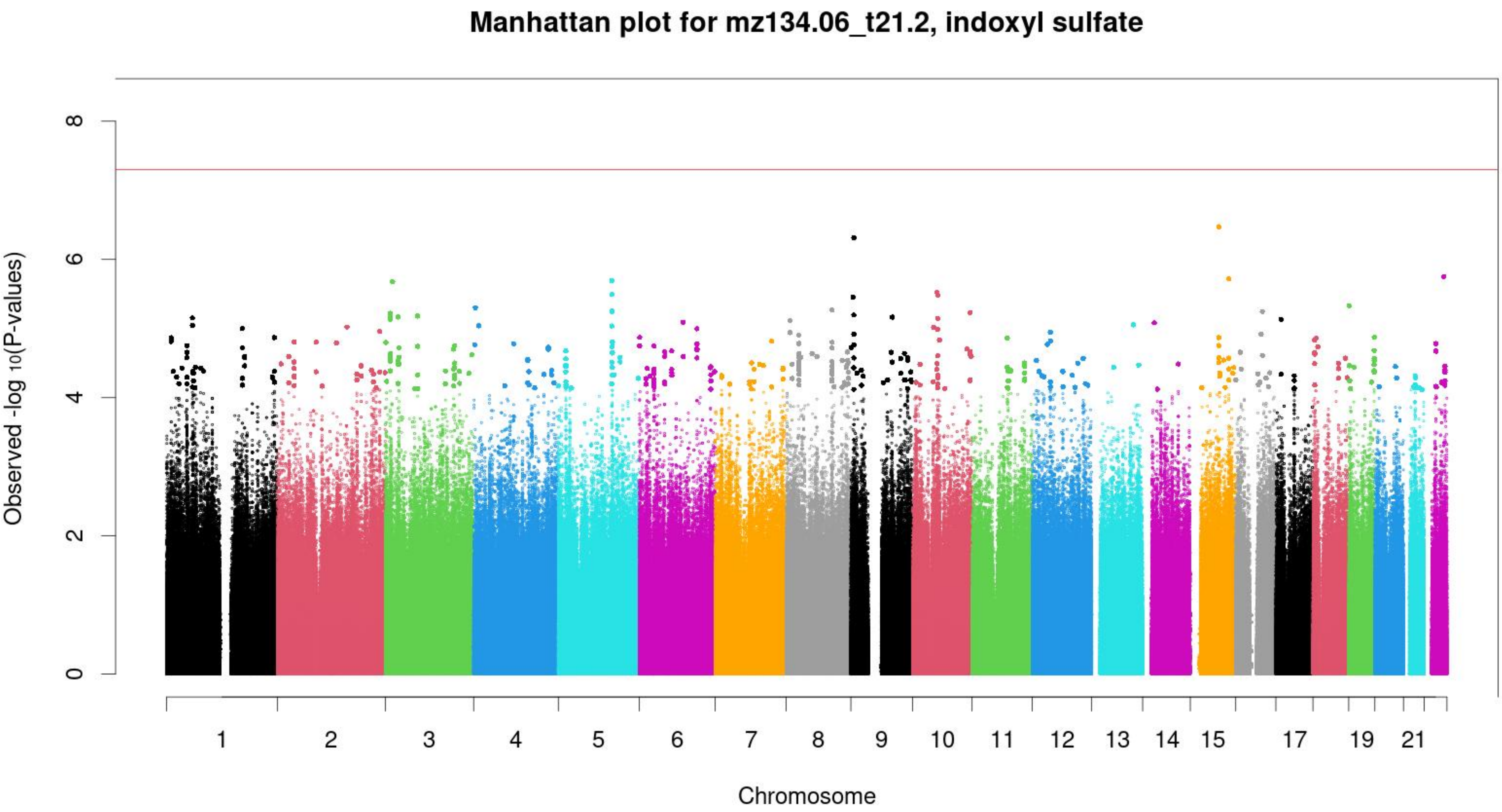
Manhattan plot for mz134.06_t21.2, indoxyl sulfate
Observed -log 10(P-values)
0
2
4
6
8
1
2
3
4
5
6
7
8
9
10
11
12
13
14
15
17
19
21
Chromosome

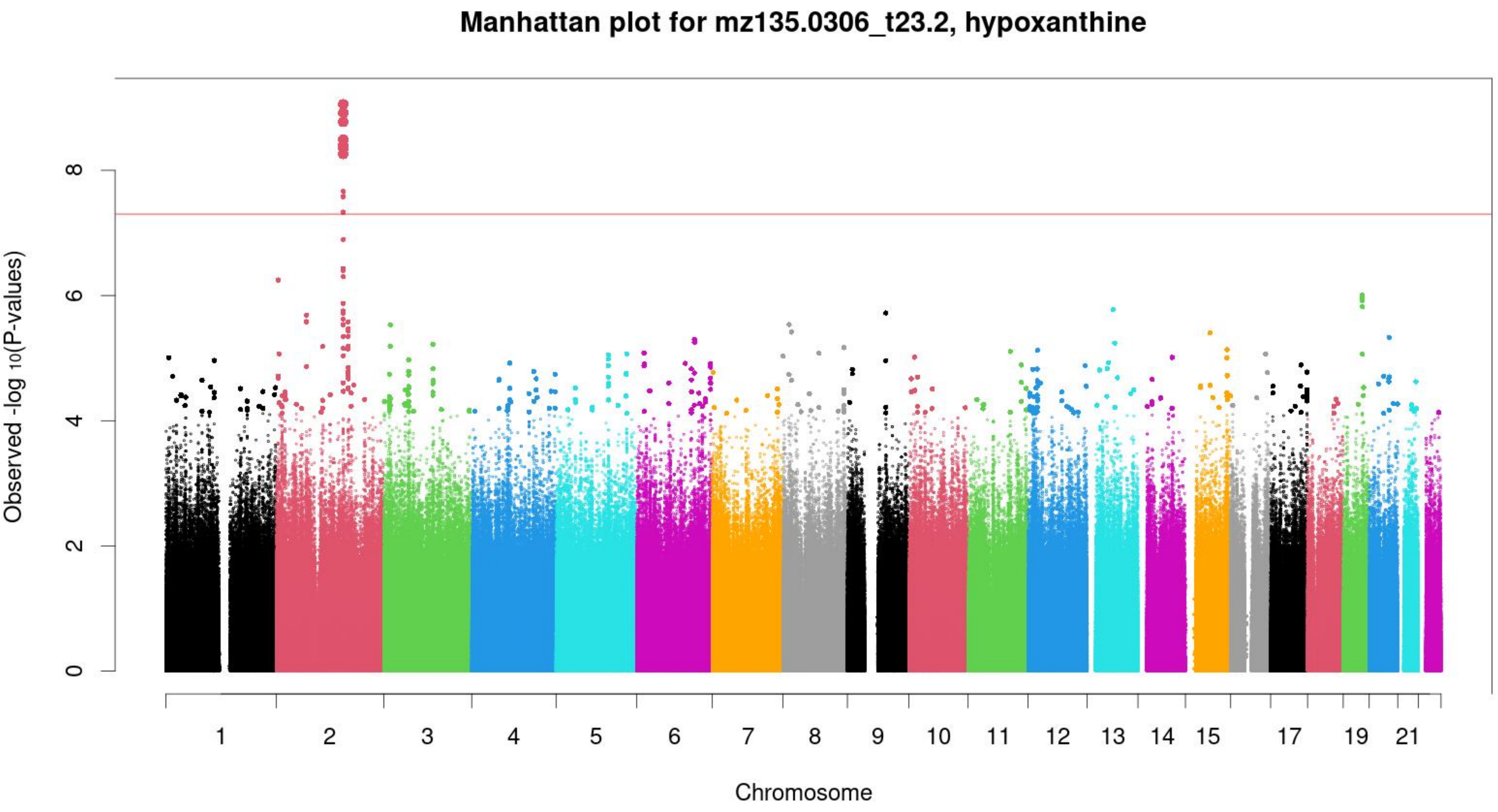
Manhattan plot for mz135.0306_t23.2, hypoxanthine
Observed -log 10(P-values)
0
2
4
6
8
Chromosome
1
2
3
4
5
6
7
8
9
10
11
12
13
14
15
17
19
21

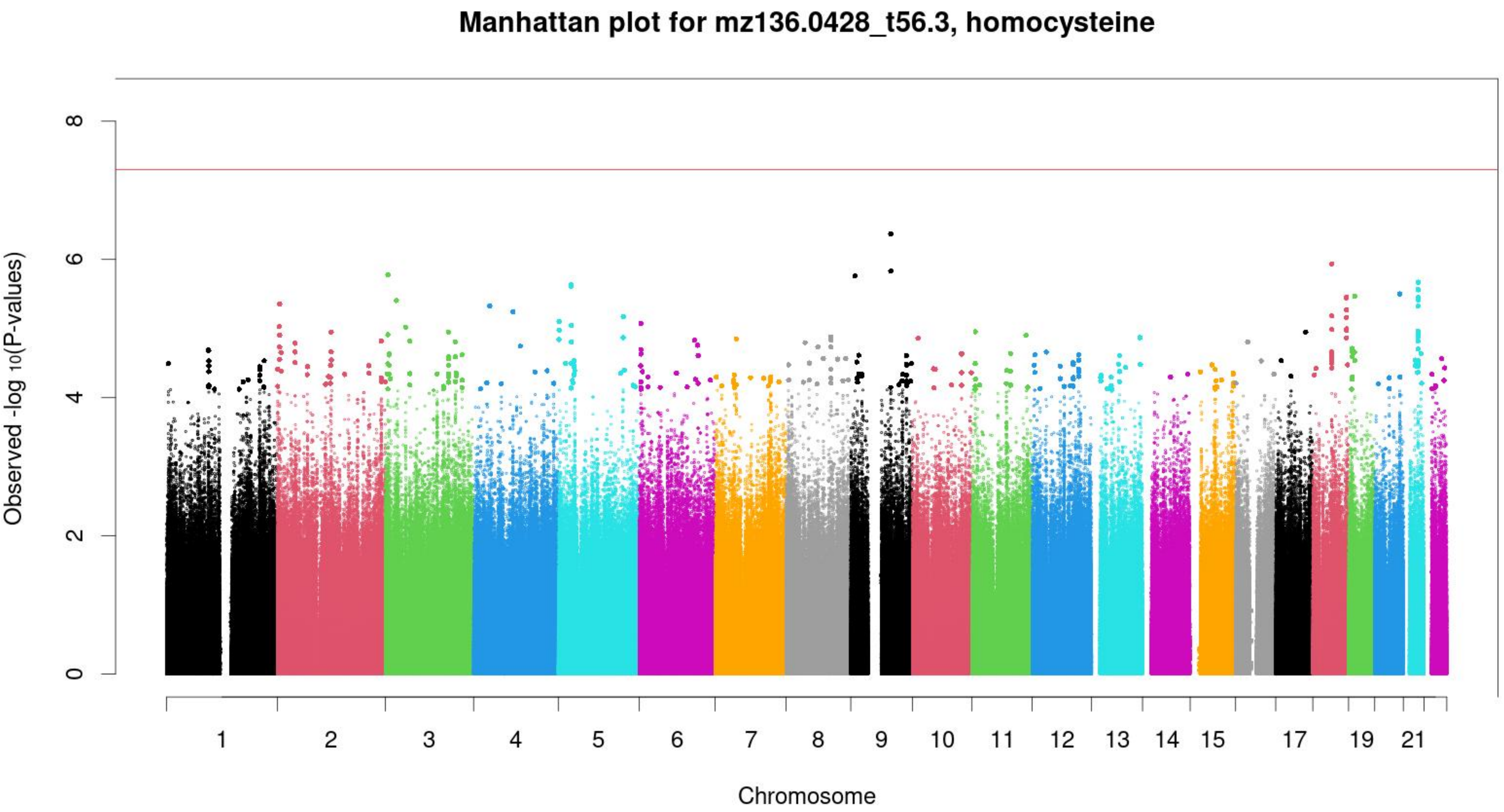
Manhattan plot for mz136.0428_t56.3, homocysteine
Observed -log 10(P-values)
0
2
4
6
8
Chromosome
1
2
3
4
5
6
7
8
9
10
11
12
13
14
15
17
19
21

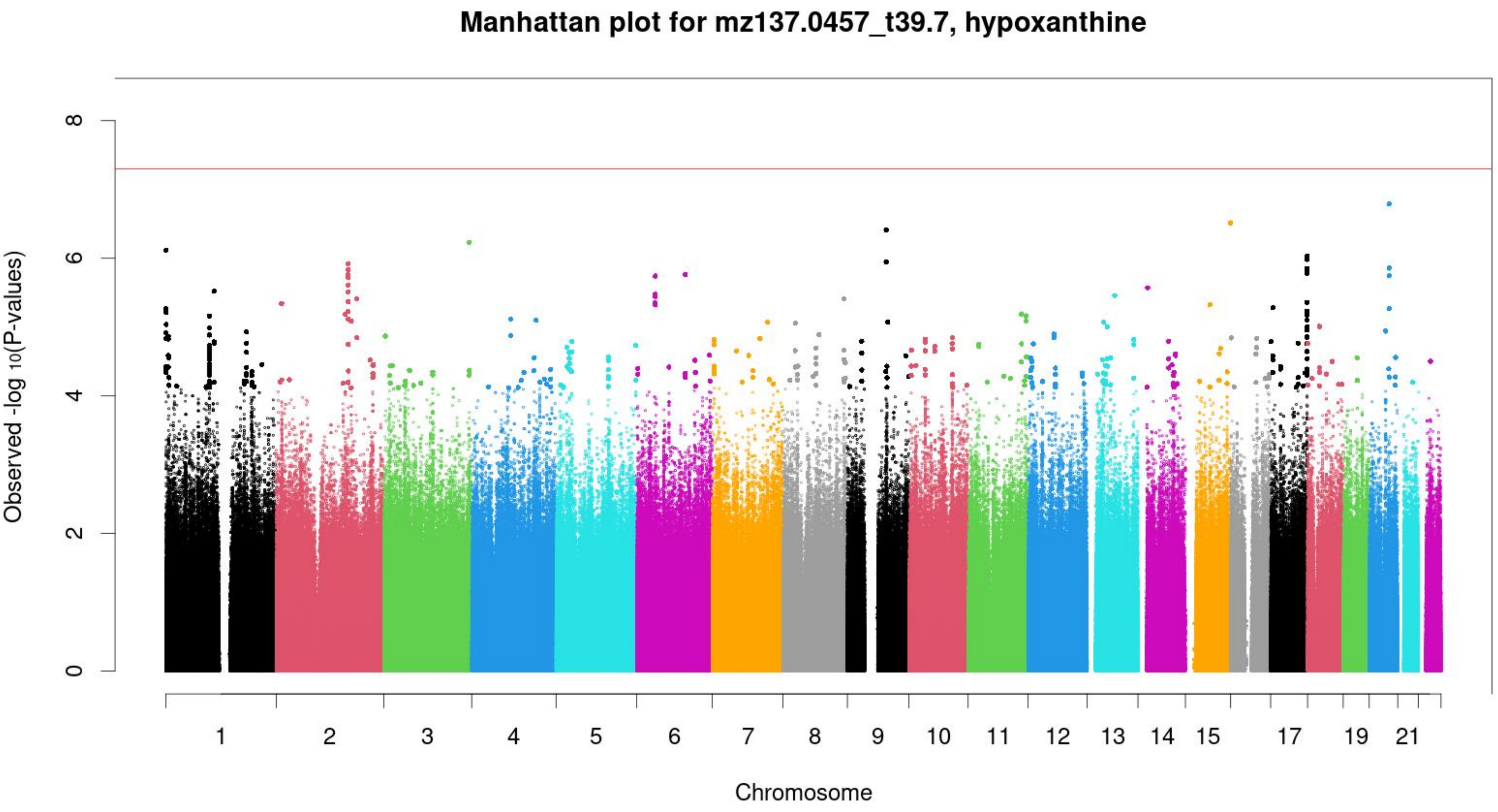

Manhattan plot for mz137.0457_t39.7, hypoxanthine
Observed -log 10(P-values)
0
2
4
6
8
1
2
3
4
5
6
7
8
9
10
11
12
13
14
15
17
19
21
Chromosome

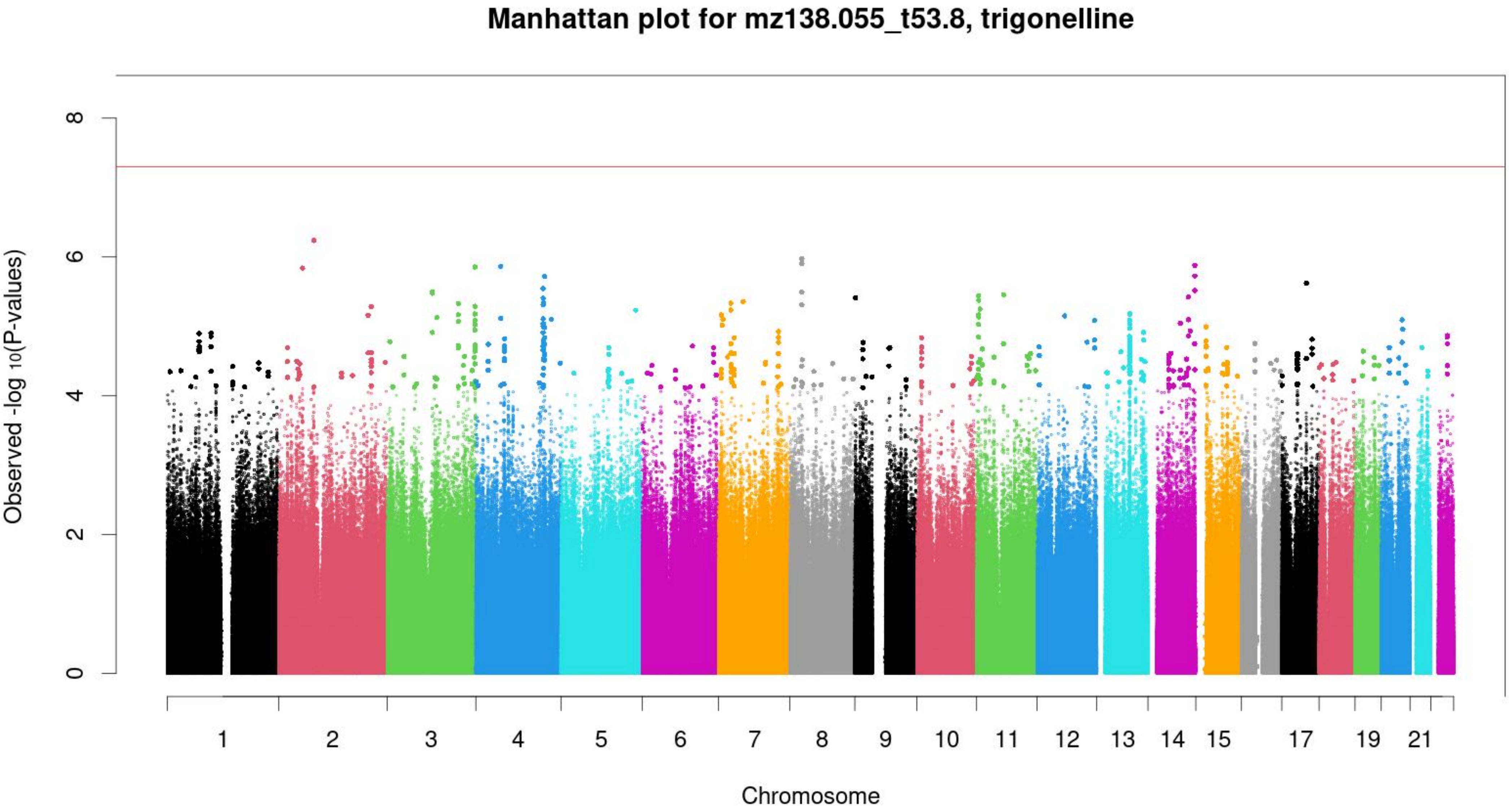

Manhattan plot for mz138.055_t53.8, trigonelline
Observed -log 10(P-values)
0
2
4
6
8
1
2
3
4
5
6
7
8
9
10
11
12
13
14
15
17
19
21
Chromosome

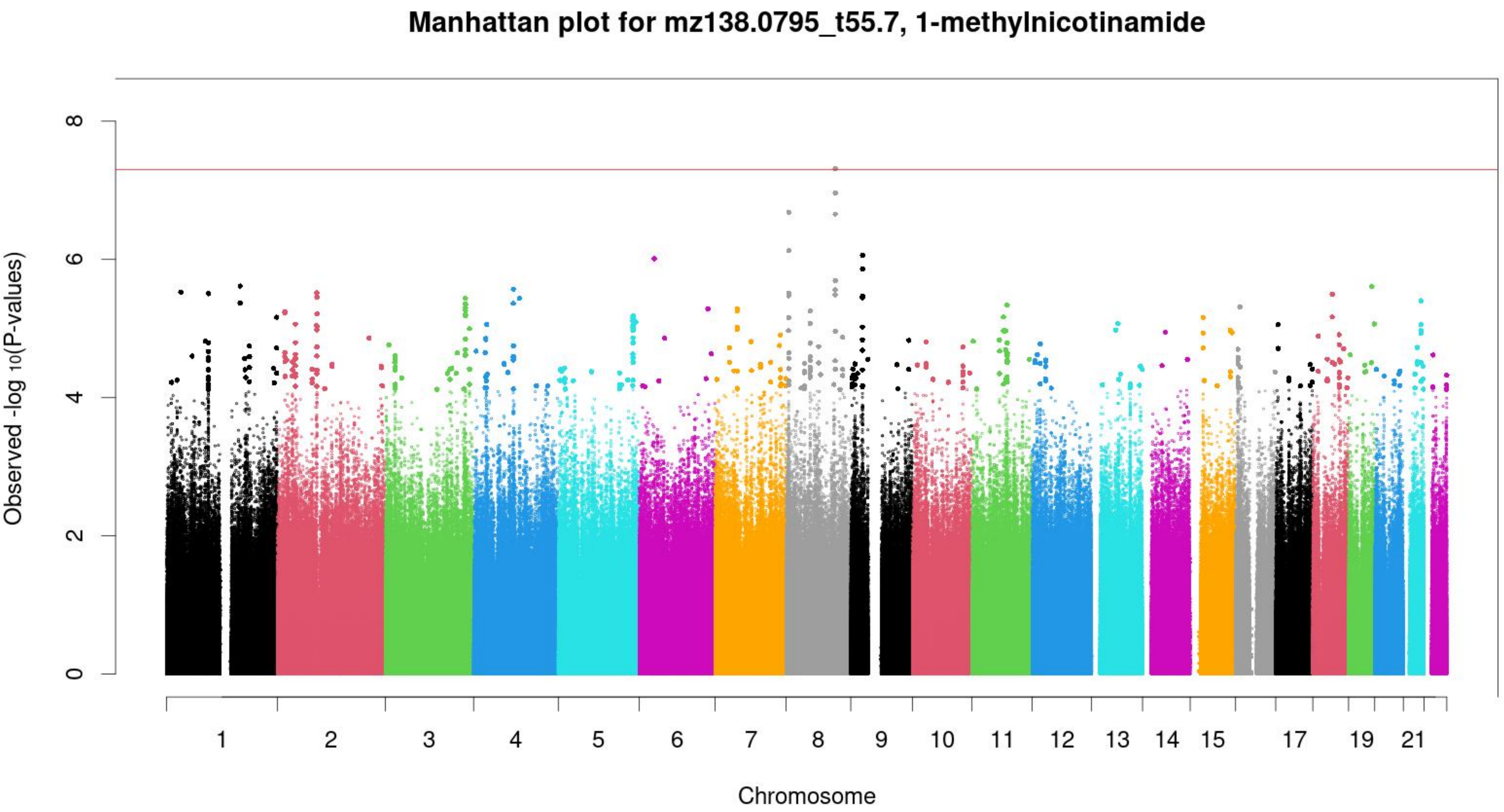

Manhattan plot for mz138.0795_t55.7, 1-methylnicotinamide
Observed -log 10(P-values)
0
2
4
6
8
1
2
3
4
5
6
7
8
9
10
11
12
13
14
15
17
19
21
Chromosome

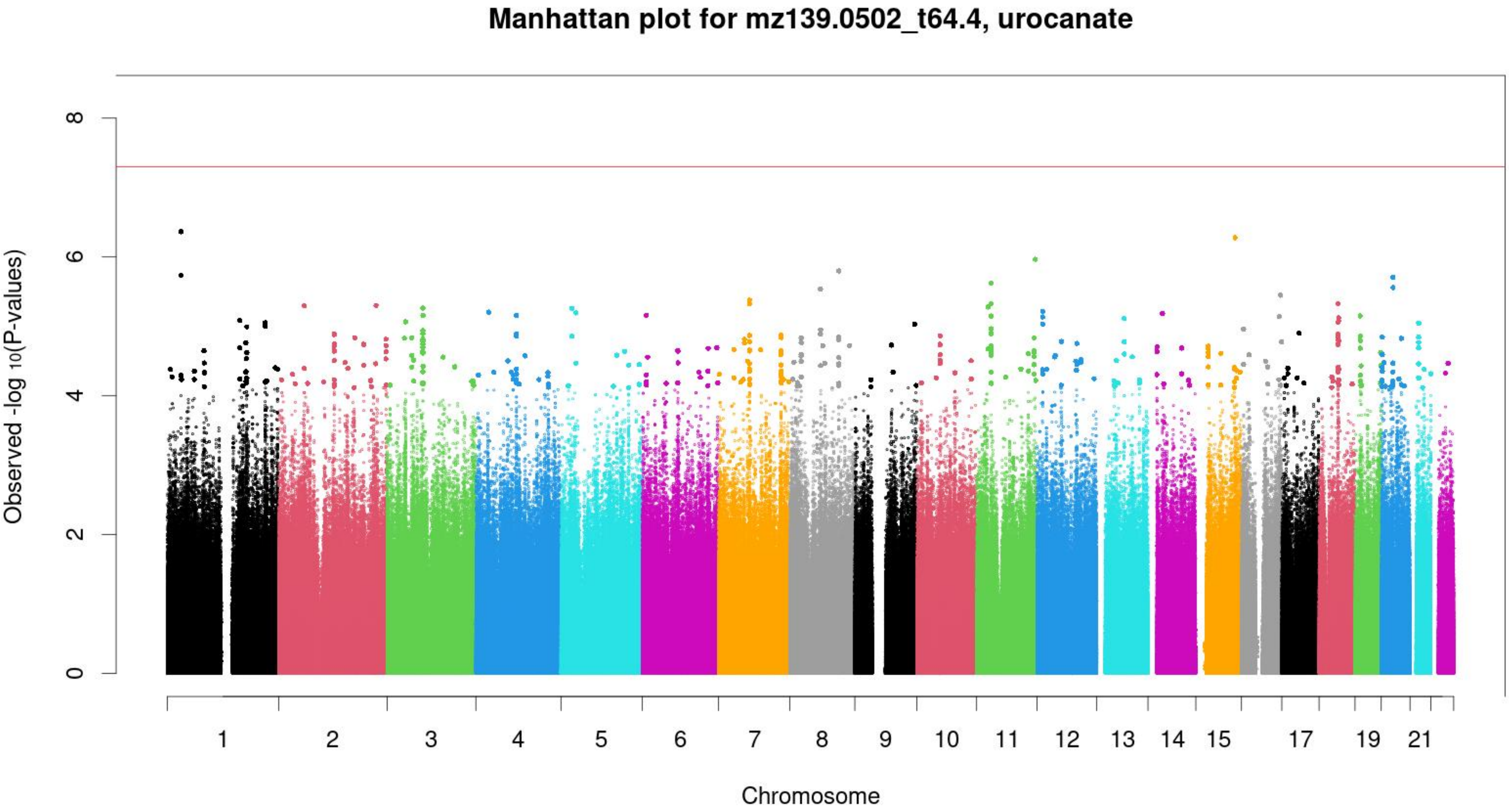

Manhattan plot for mz139.0502_t64.4, urocanate
Observed -log 10(P-values)
0
2
4
6
8
1
2
3
4
5
6
7
8
9
10
11
12
13
14
15
17
19
21
Chromosome

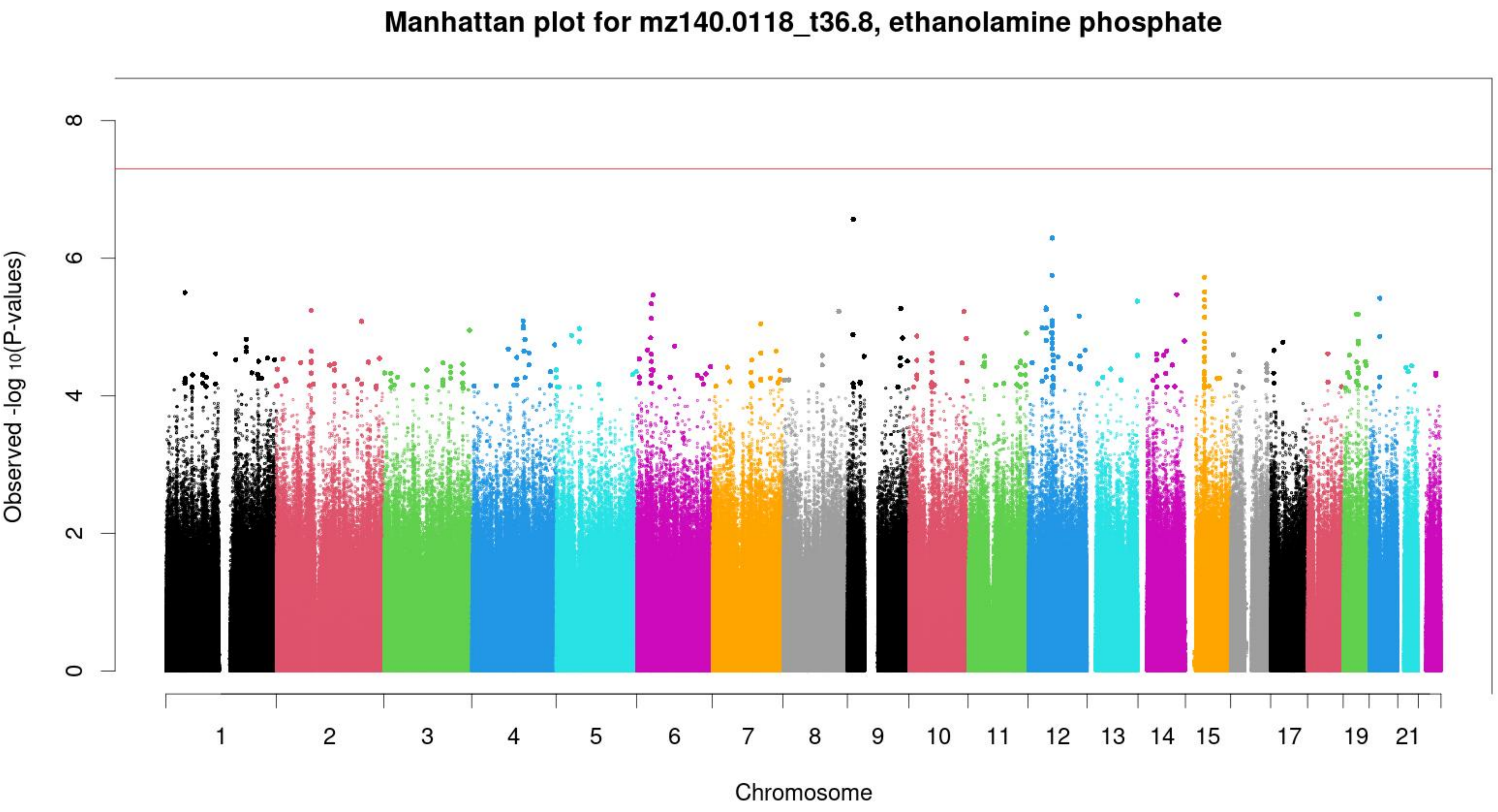

Manhattan plot for mz140.0118_t36.8, ethanolamine phosphate
Observed -log 10(P-values)
0
2
4
6
8
1
2
3
4
5
6
7
8
9
10
11
12
13
14
15
17
19
21
Chromosome

Manhattan plot for mz143.1077_t59.1, caprylic acid

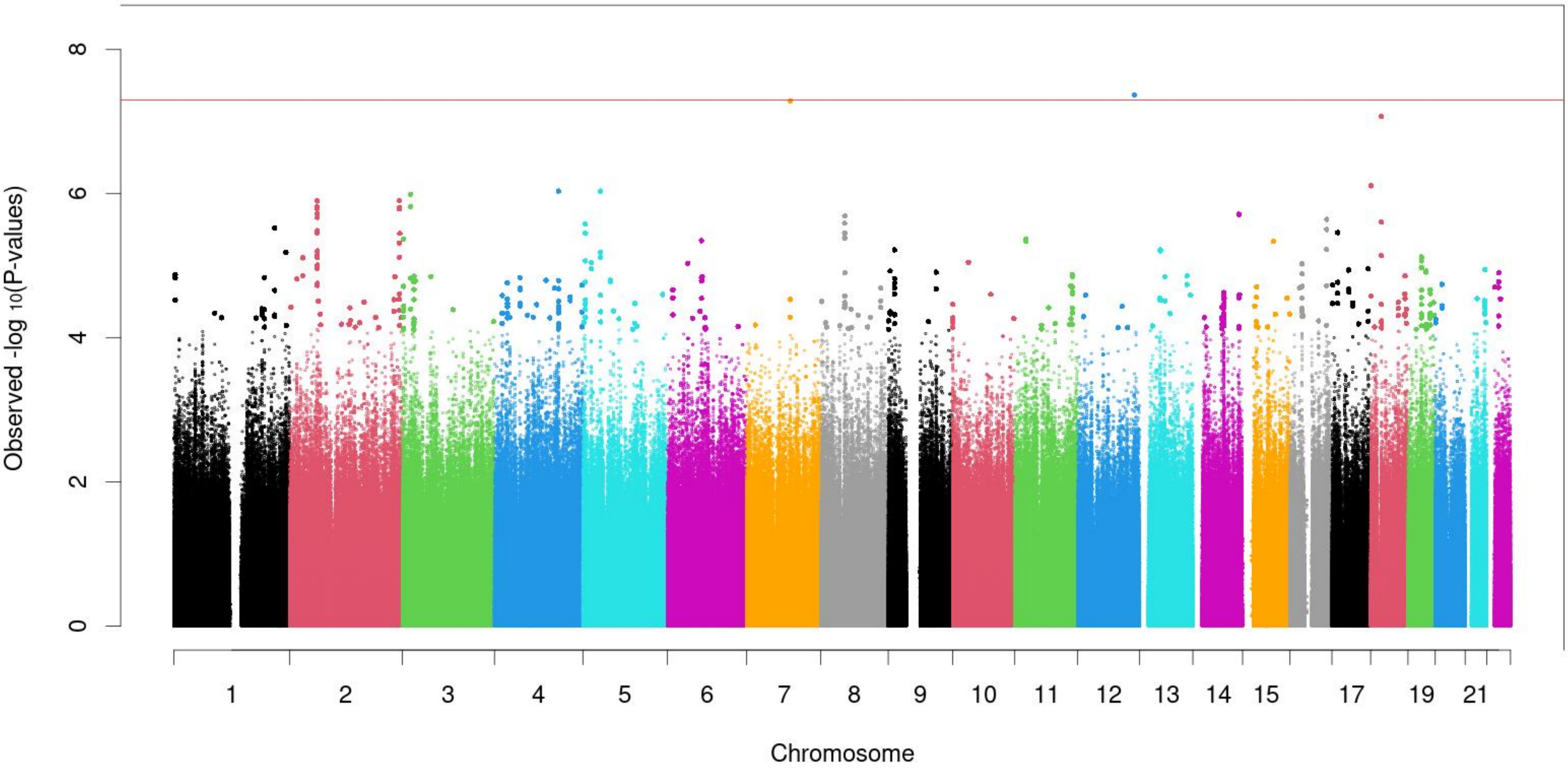

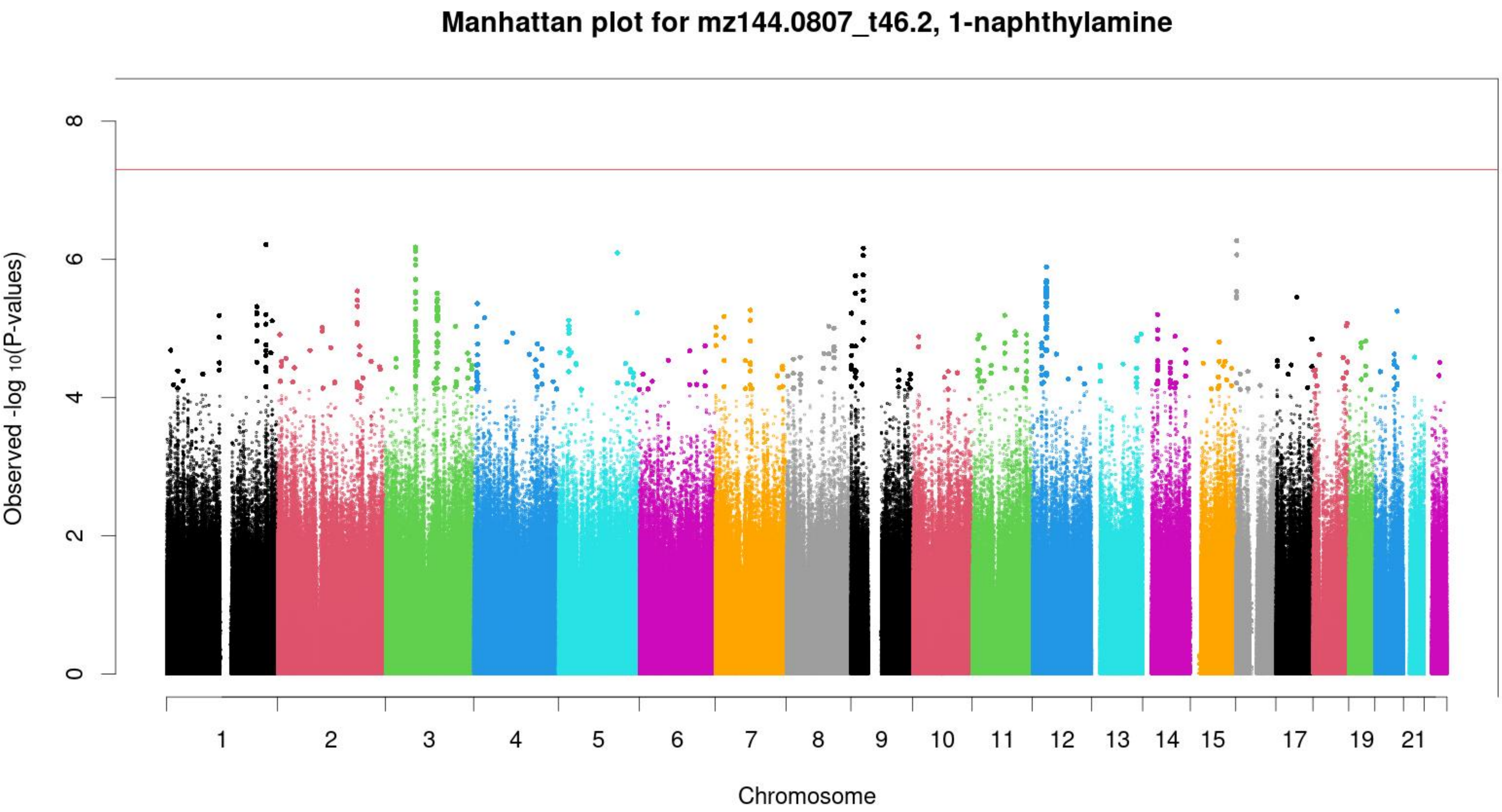

Manhattan plot for mz144.0807_t46.2, 1-naphthylamine
Observed -log 10(P-values)
0
2
4
6
8
1
2
3
4
5
6
7
8
9
10
11
12
13
14
15
17
19
21
Chromosome

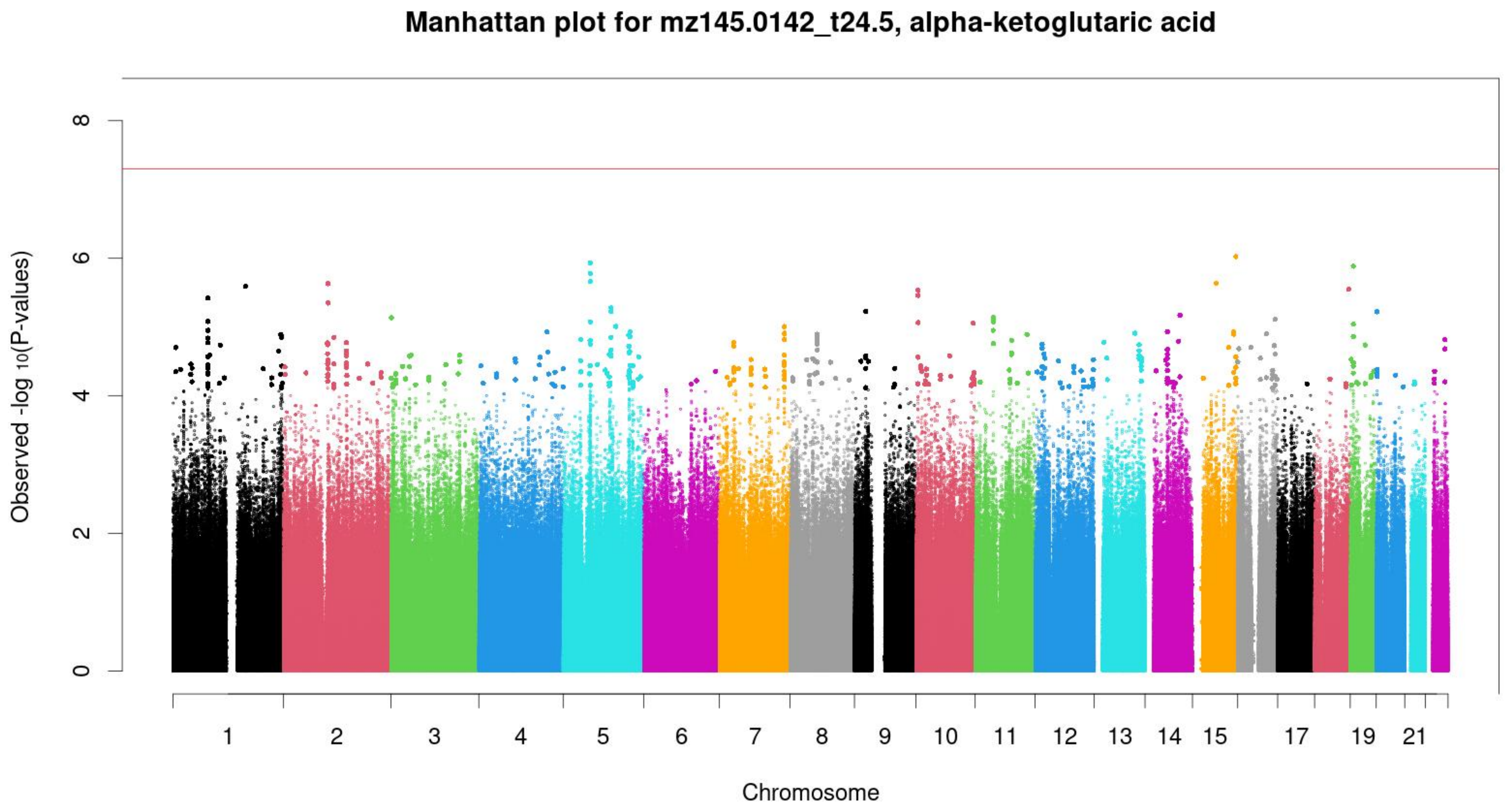

Manhattan plot for mz145.0142_t24.5, alpha-ketoglutaric acid
Observed -log 10(P-values)
0
2
4
6
8
1
2
3
4
5
6
7
8
9
10
11
12
13
14
15
17
19
21
Chromosome

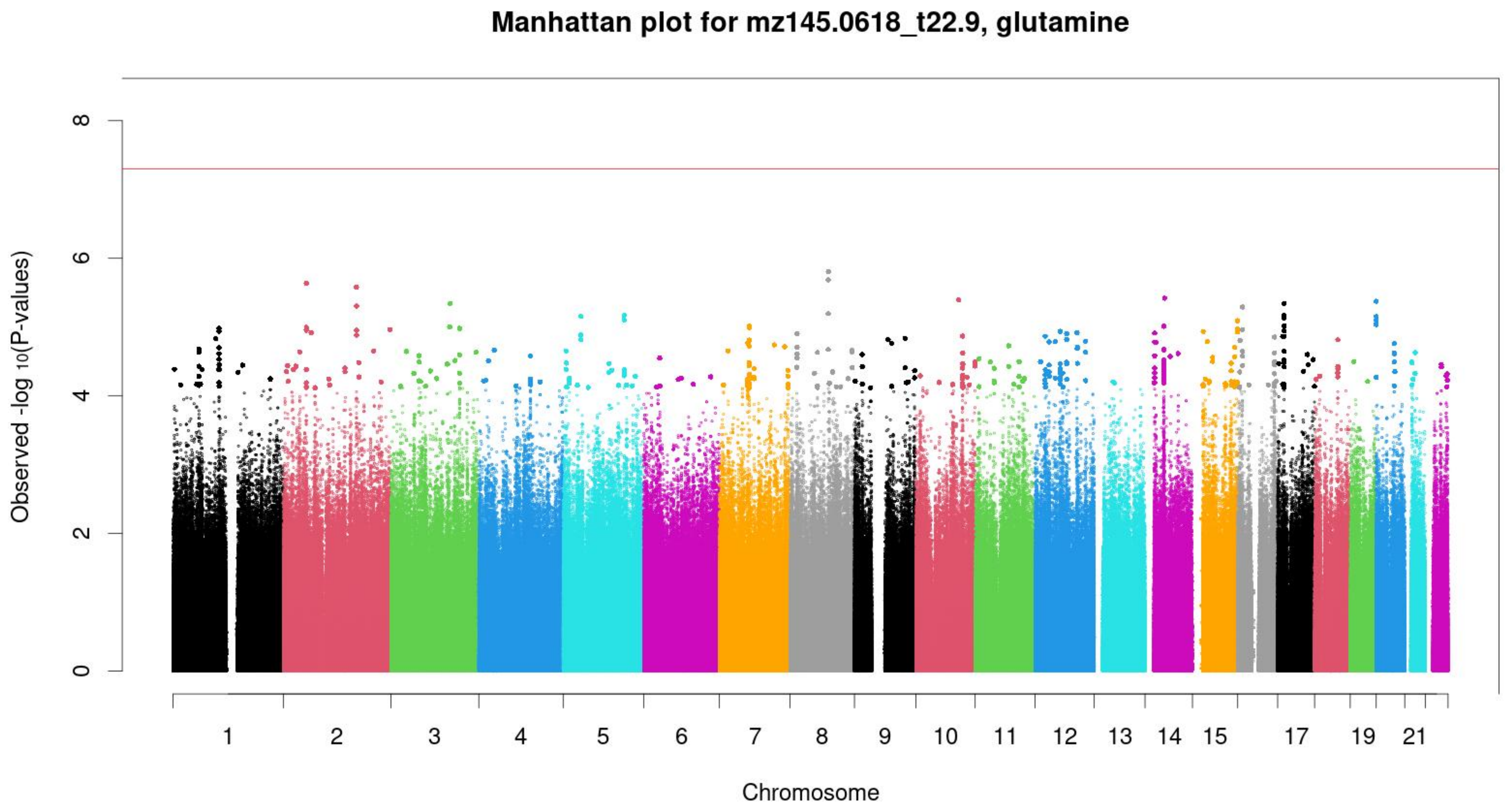
Manhattan plot for mz145.0618_t22.9, glutamine
Observed -log 10(P-values)
0
2
4
6
8
1
2
3
4
5
6
7
8
9
10
11
12
13
14
15
17
19
21
Chromosome

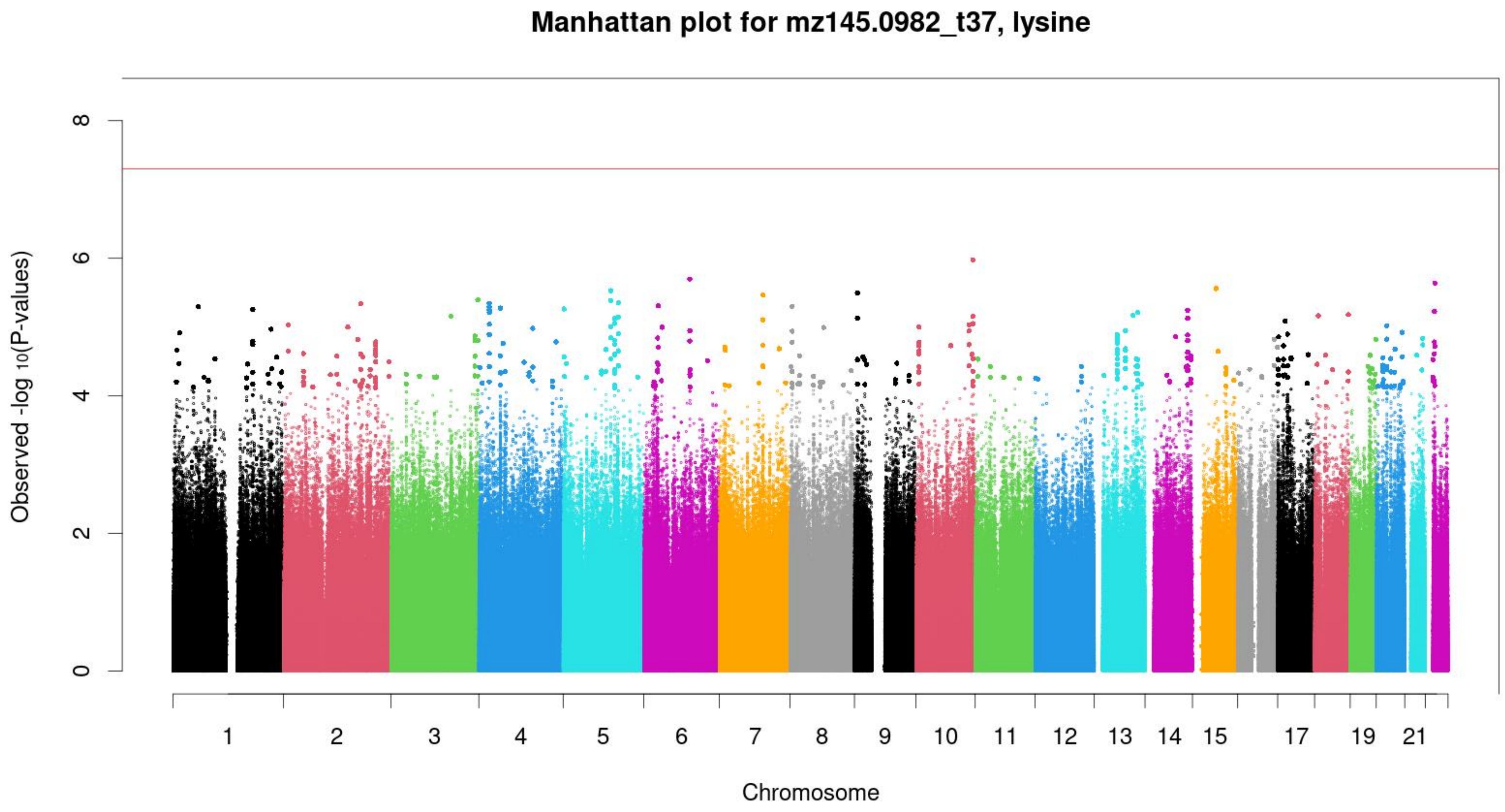

Manhattan plot for mz145.0982_t37, lysine
Observed -log 10(P-values)
0
2
4
6
8
1
2
3
4
5
6
7
8
9
10
11
12
13
14
15
17
19
21
Chromosome

Manhattan plot for mz147.045_t46.9, trans-cinnamate

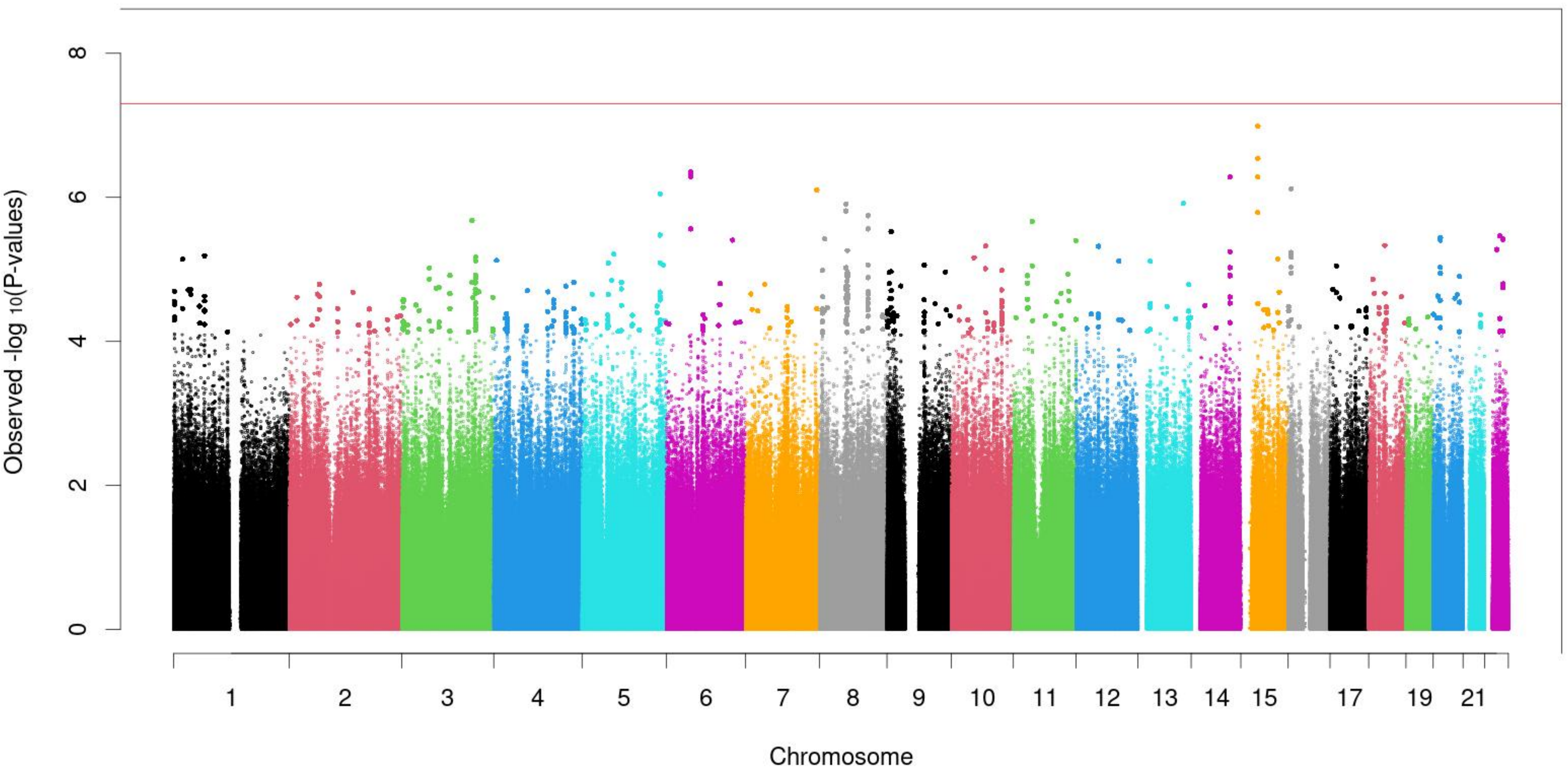

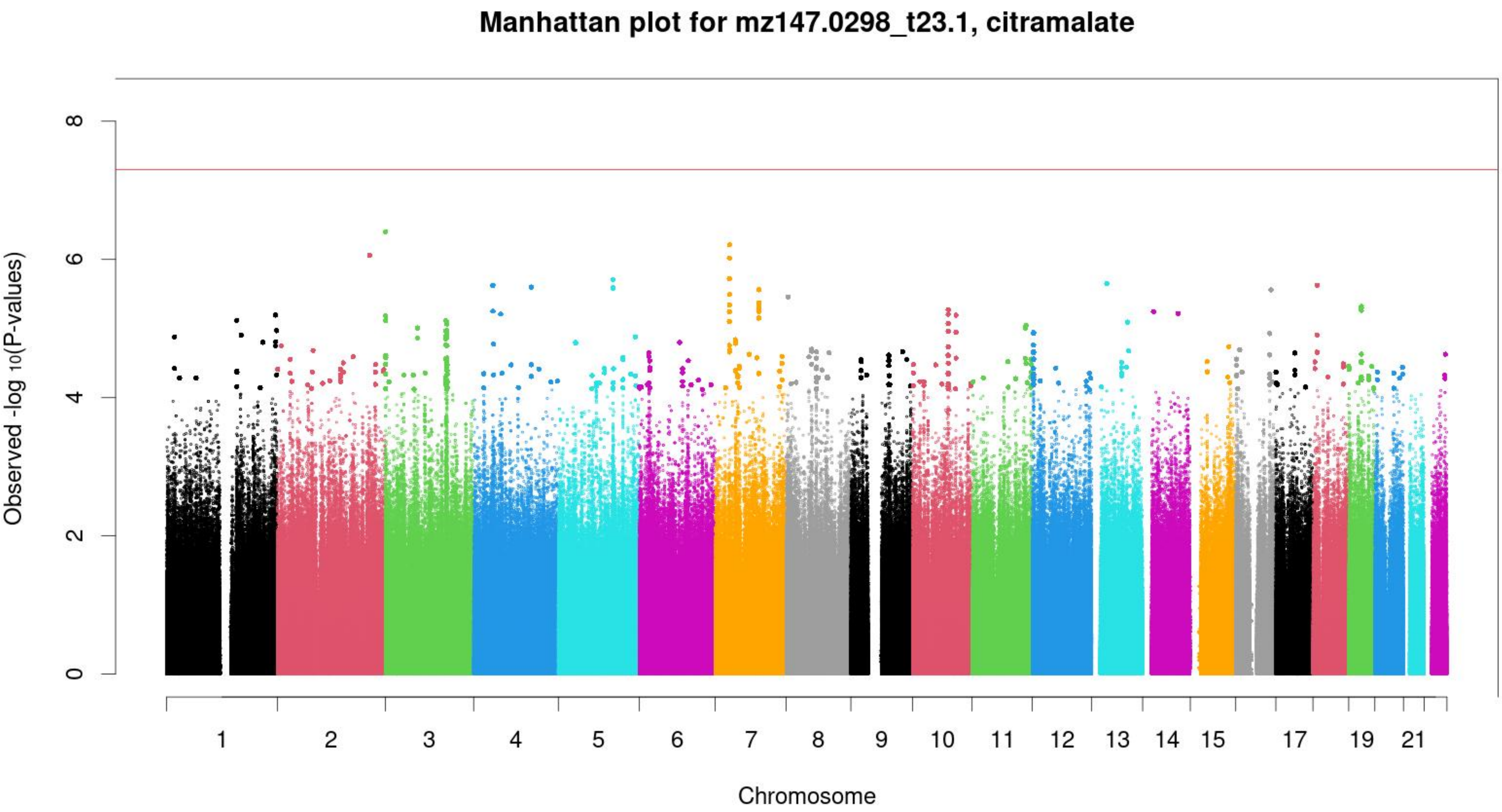

Manhattan plot for mz147.0298_t23.1, citramalate
Observed -log 10(P-values)
0
2
4
6
8
1
2
3
4
5
6
7
8
9
10
11
12
13
14
15
17
19
21
Chromosome

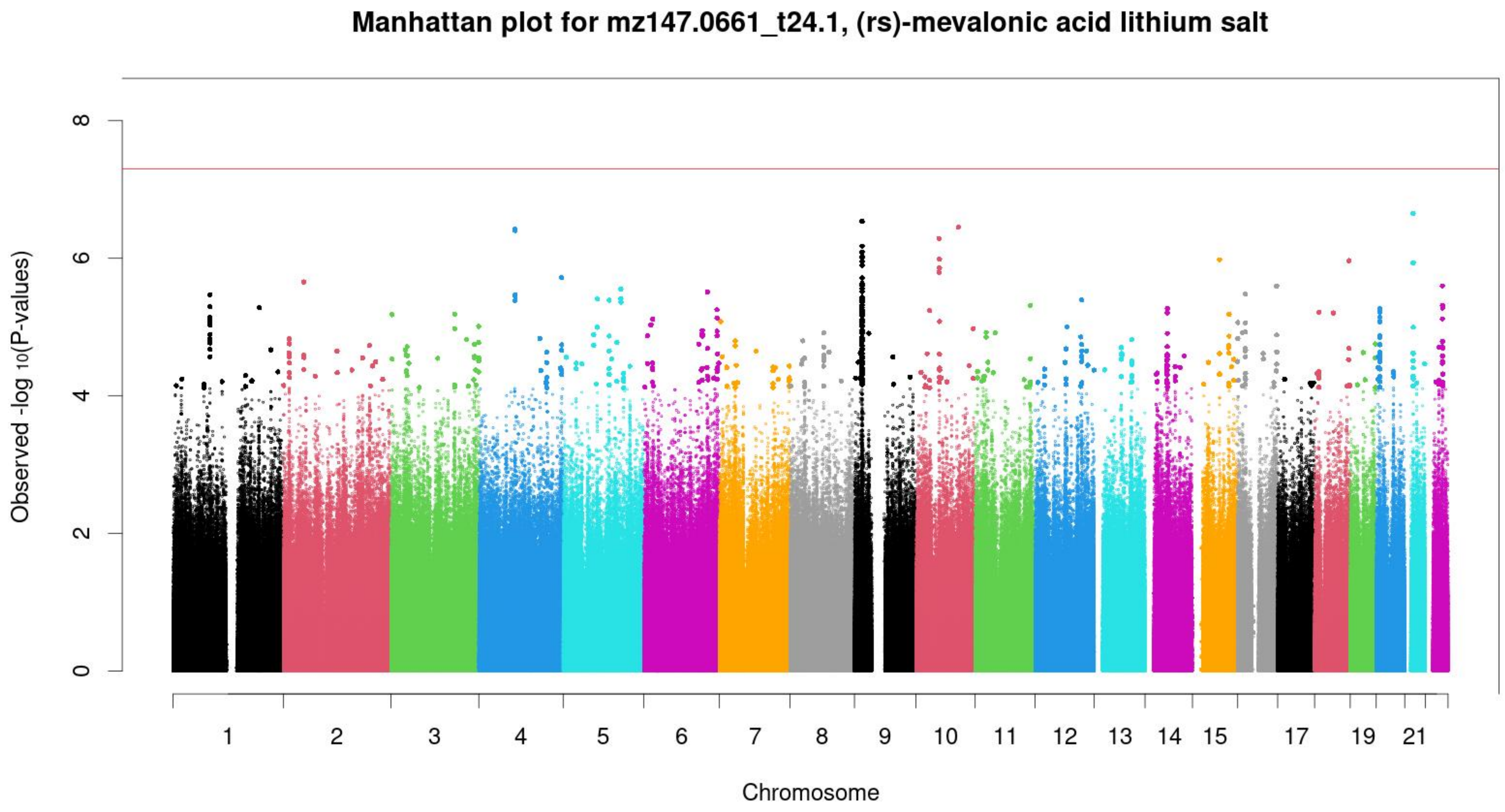
Manhattan plot for mz147.0661_t24.1, (rs)-mevalonic acid lithium salt
Observed -log 10(P-values)
0
2
4
6
8
1
2
3
4
5
6
7
8
9
10
11
12
13
14
15
17
19
21
Chromosome

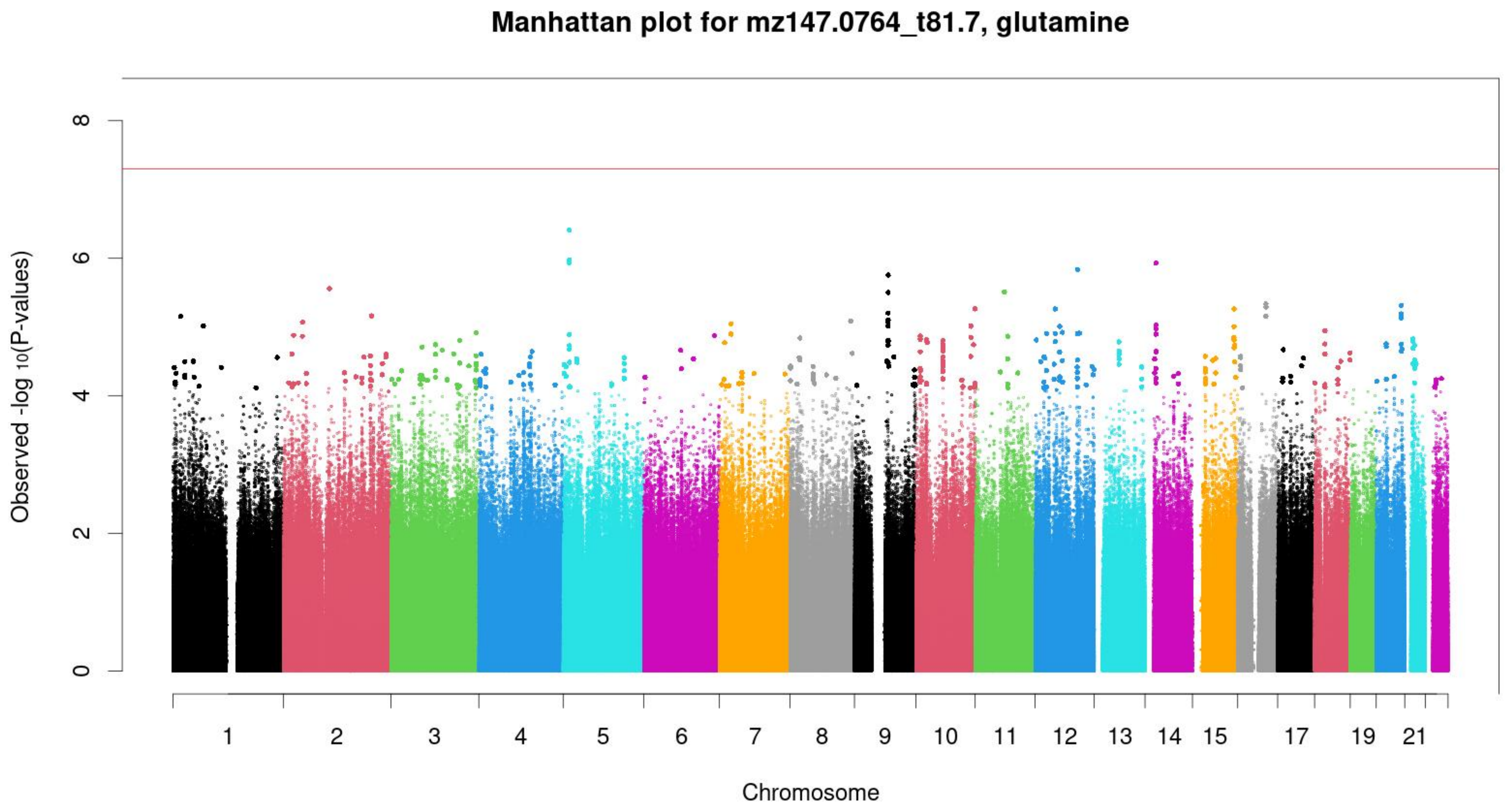

Manhattan plot for mz147.0764_t81.7, glutamine
Observed -log 10(P-values)
0
2
4
6
8
1
2
3
4
5
6
7
8
9
10
11
12
13
14
15
17
19
21
Chromosome

Manhattan plot for mz148.0437_t35.7, methionine

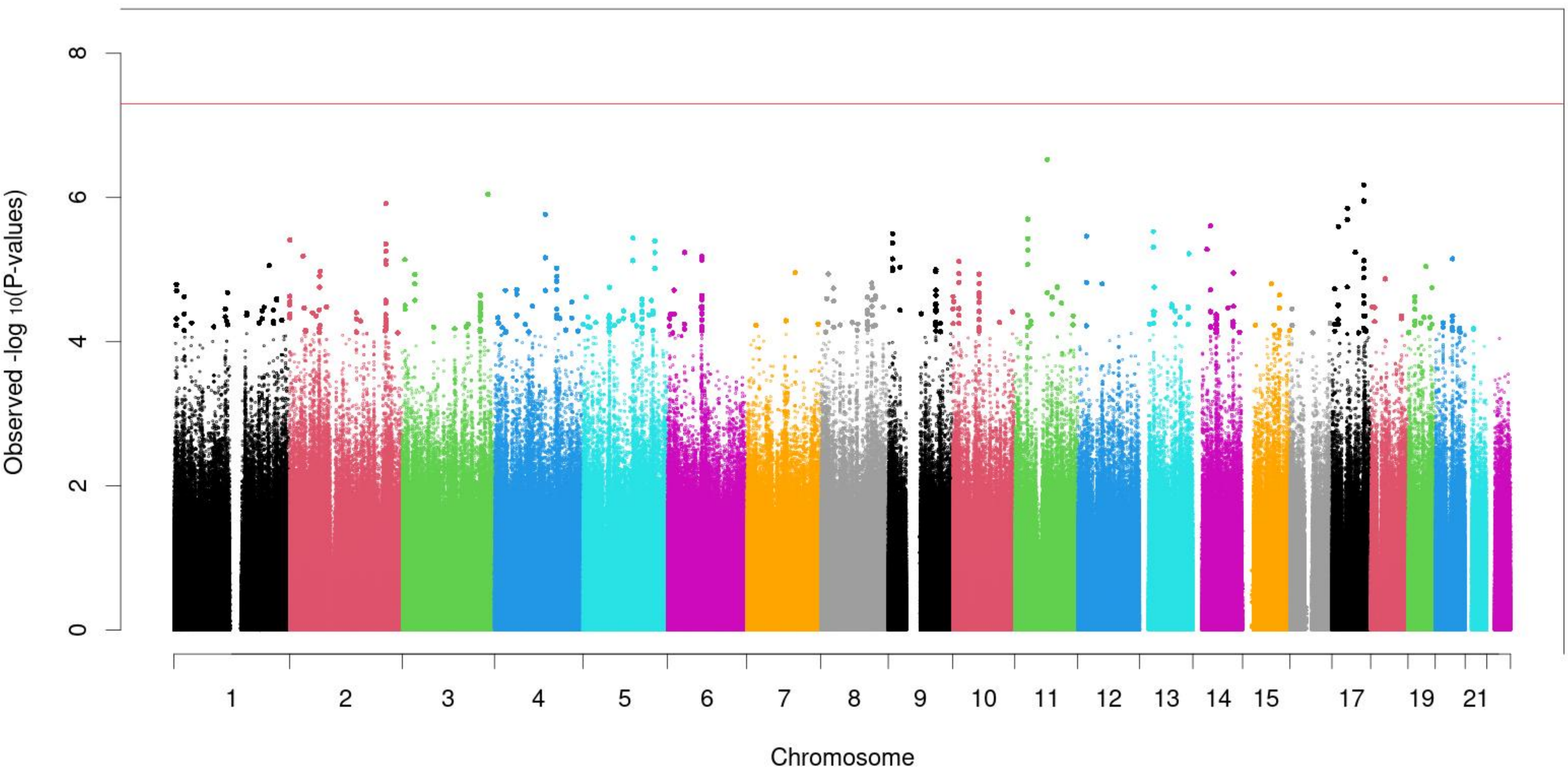

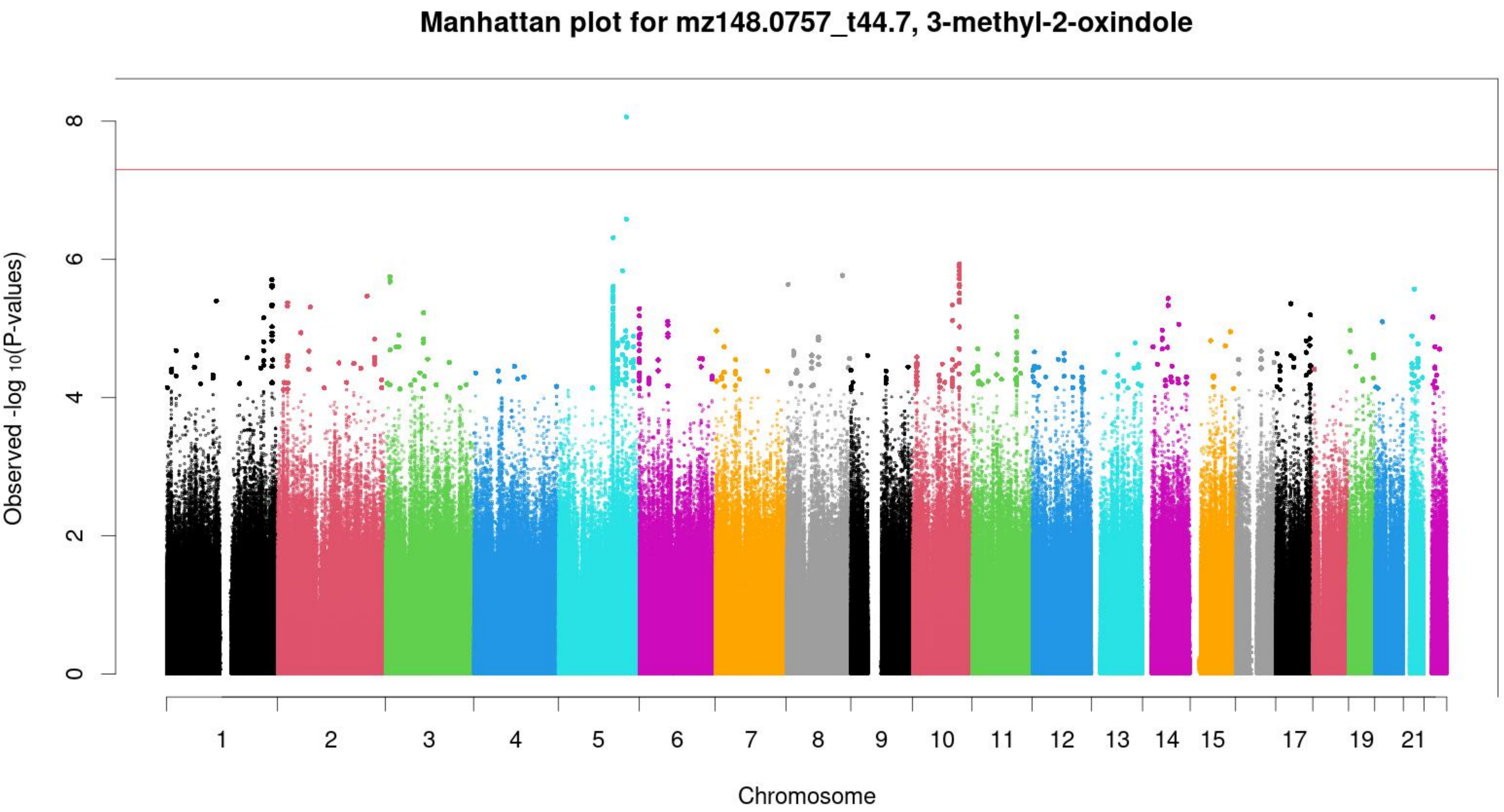
Manhattan plot for mz148.0757_t44.7, 3-methyl-2-oxindole
Observed -log 10(P-values)
0
2
4
6
8
1
2
3
4
5
6
7
8
9
10
11
12
13
14
15
17
19
21
Chromosome

Manhattan plot for mz149.0278_t28.8, 2-hydroxy-4-(methylthio)butyric acid

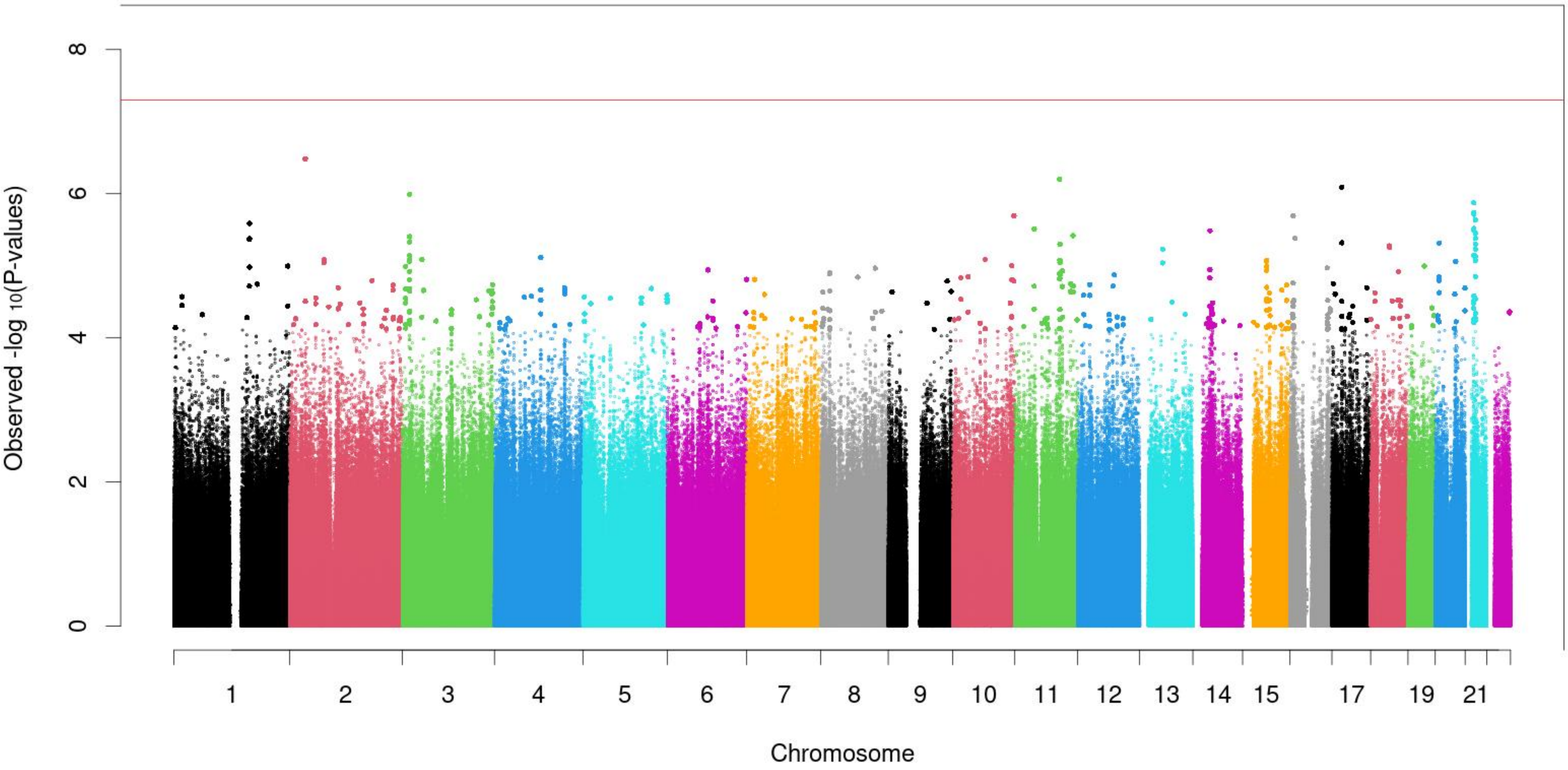

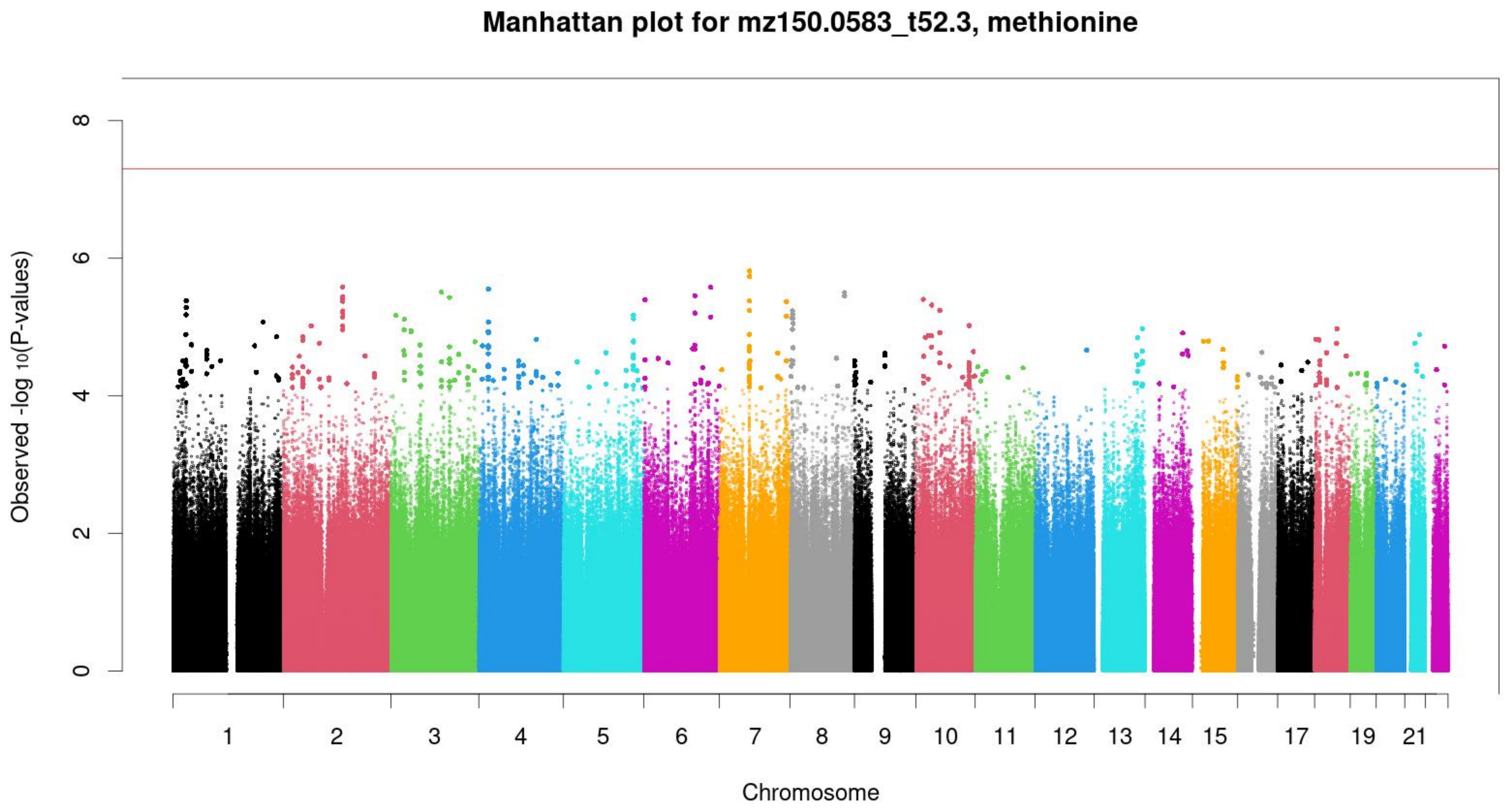
Manhattan plot for mz150.0583_t52.3, methionine
Observed -log 10(P-values)
0
2
4
6
8
1
2
3
4
5
6
7
8
9
10
11
12
13
14
15
17
19
21
Chromosome

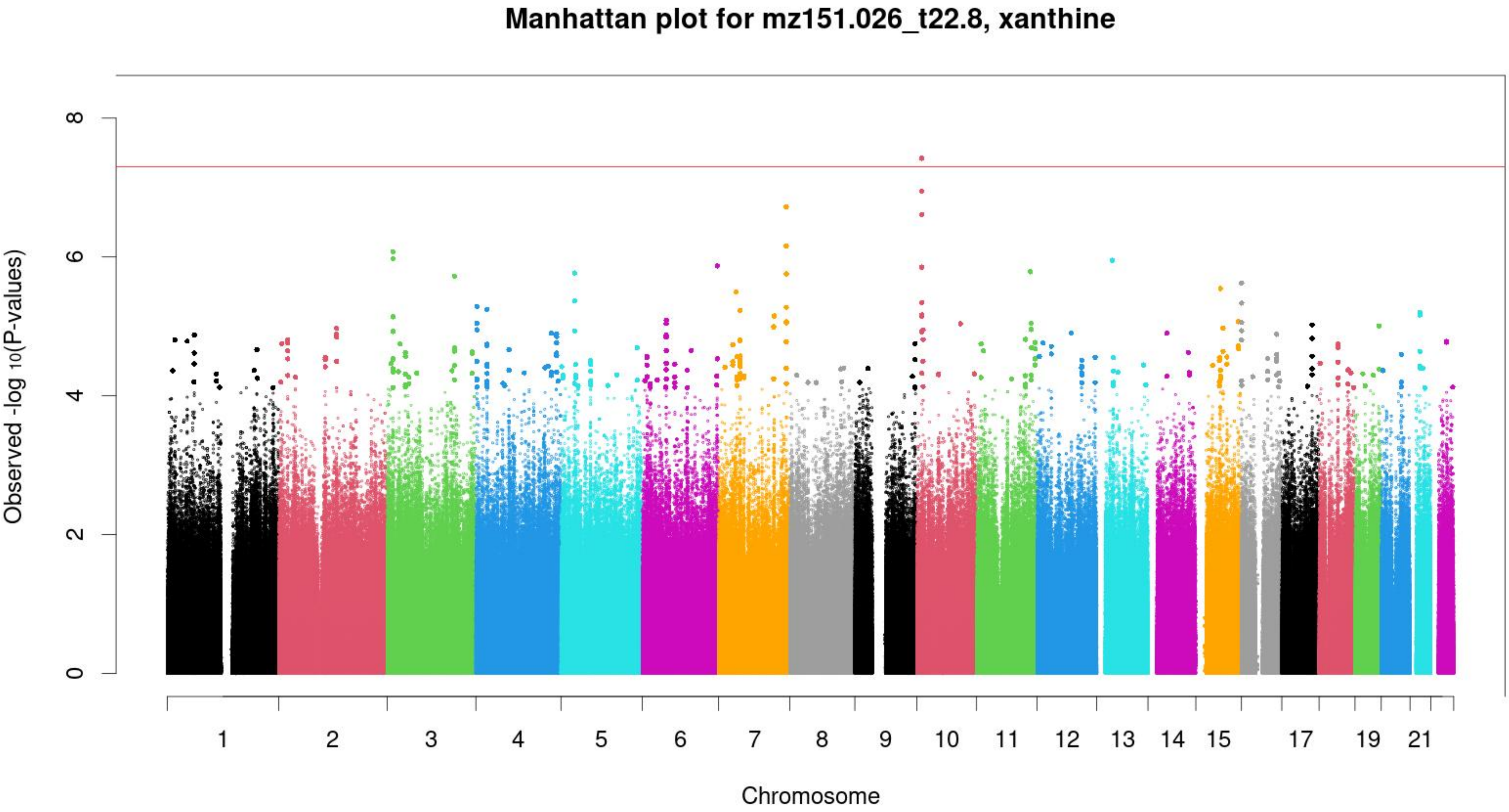

Manhattan plot for mz151.026_t22.8, xanthine
Observed -log 10(P-values)
0
2
4
6
8
1
2
3
4
5
6
7
8
9
10
11
12
13
14
15
17
19
21
Chromosome

Manhattan plot for mz153.0407_t42, xanthine

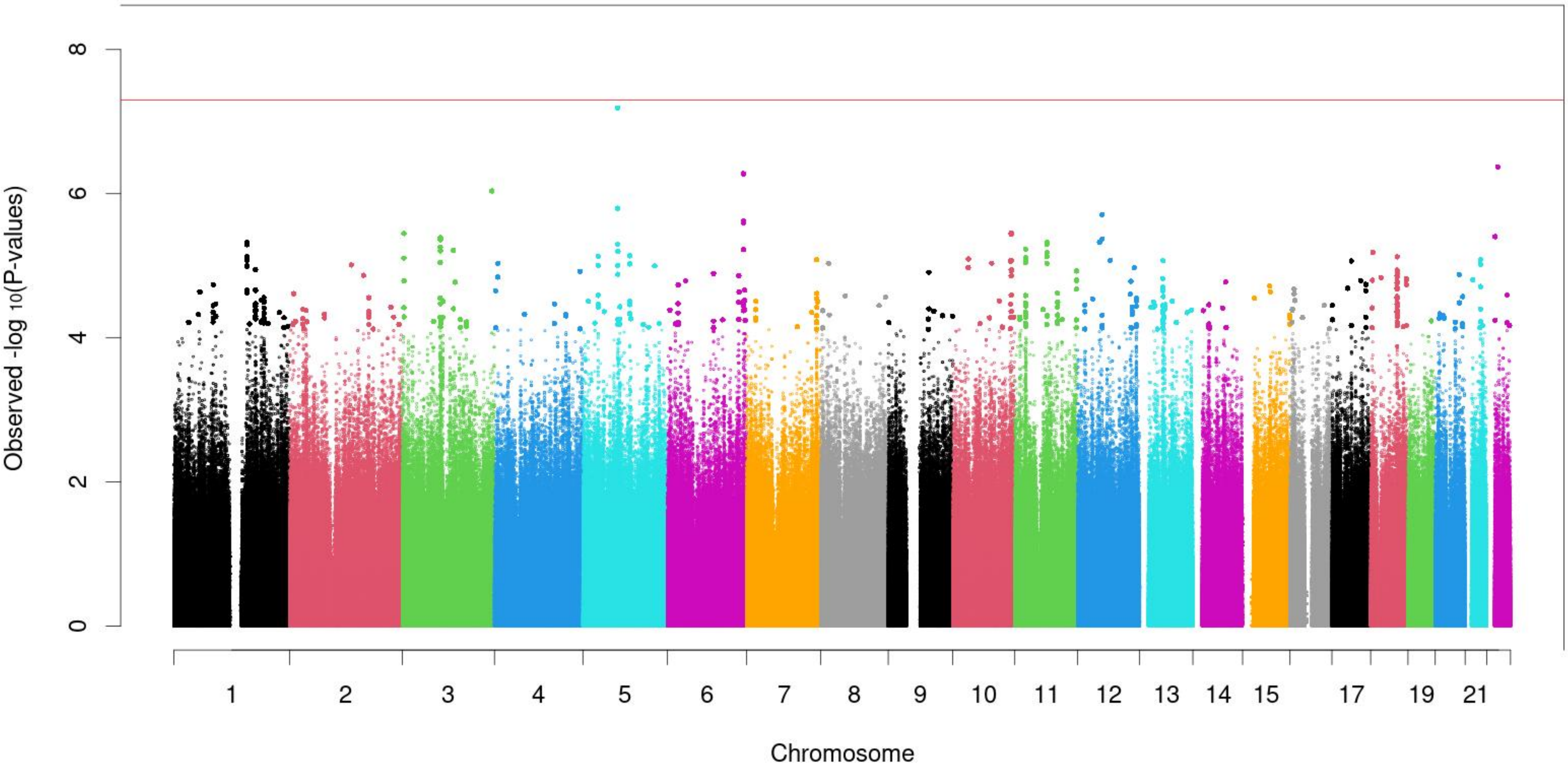

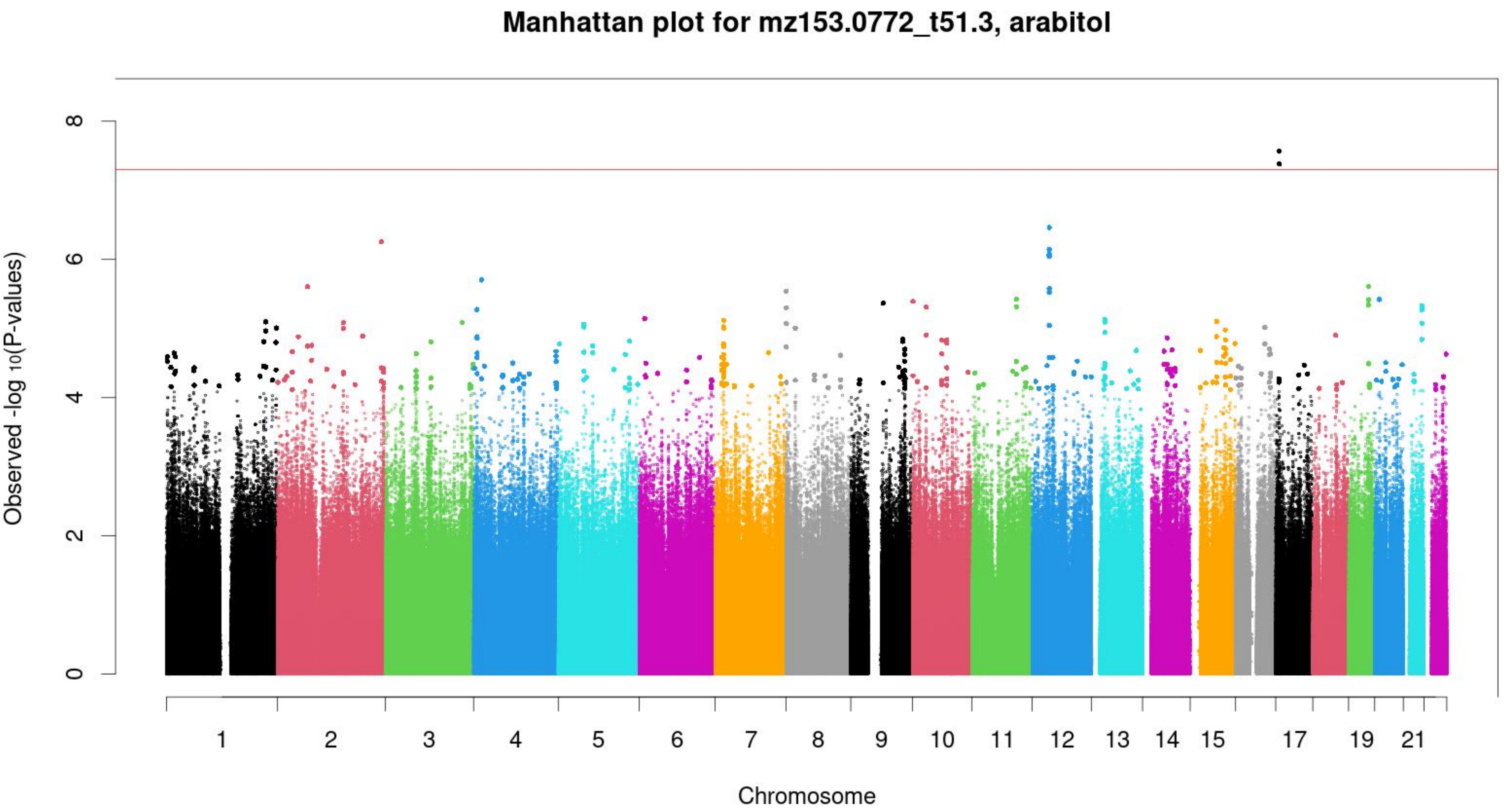
Manhattan plot for mz153.0772_t51.3, arabitol
Observed -log 10(P-values)
0
2
4
6
8
1
2
3
4
5
6
7
8
9
10
11
12
13
14
15
17
19
21
Chromosome

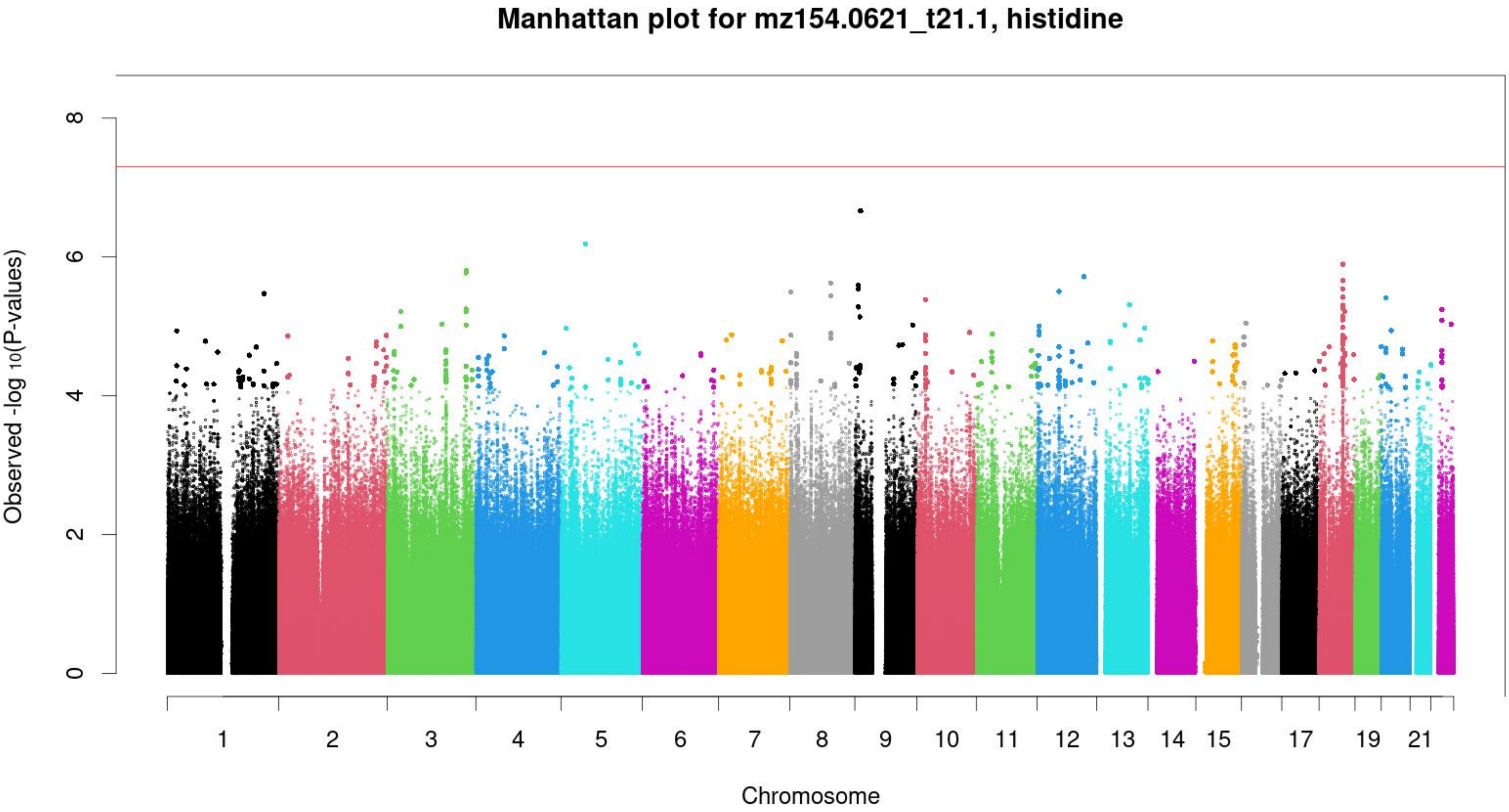

Manhattan plot for mz154.0621_t21.1, histidine
Observed -log 10(P-values)
0
2
4
6
8
Chromosome
1
2
3
4
5
6
7
8
9
10
11
12
13
14
15
17
19
21

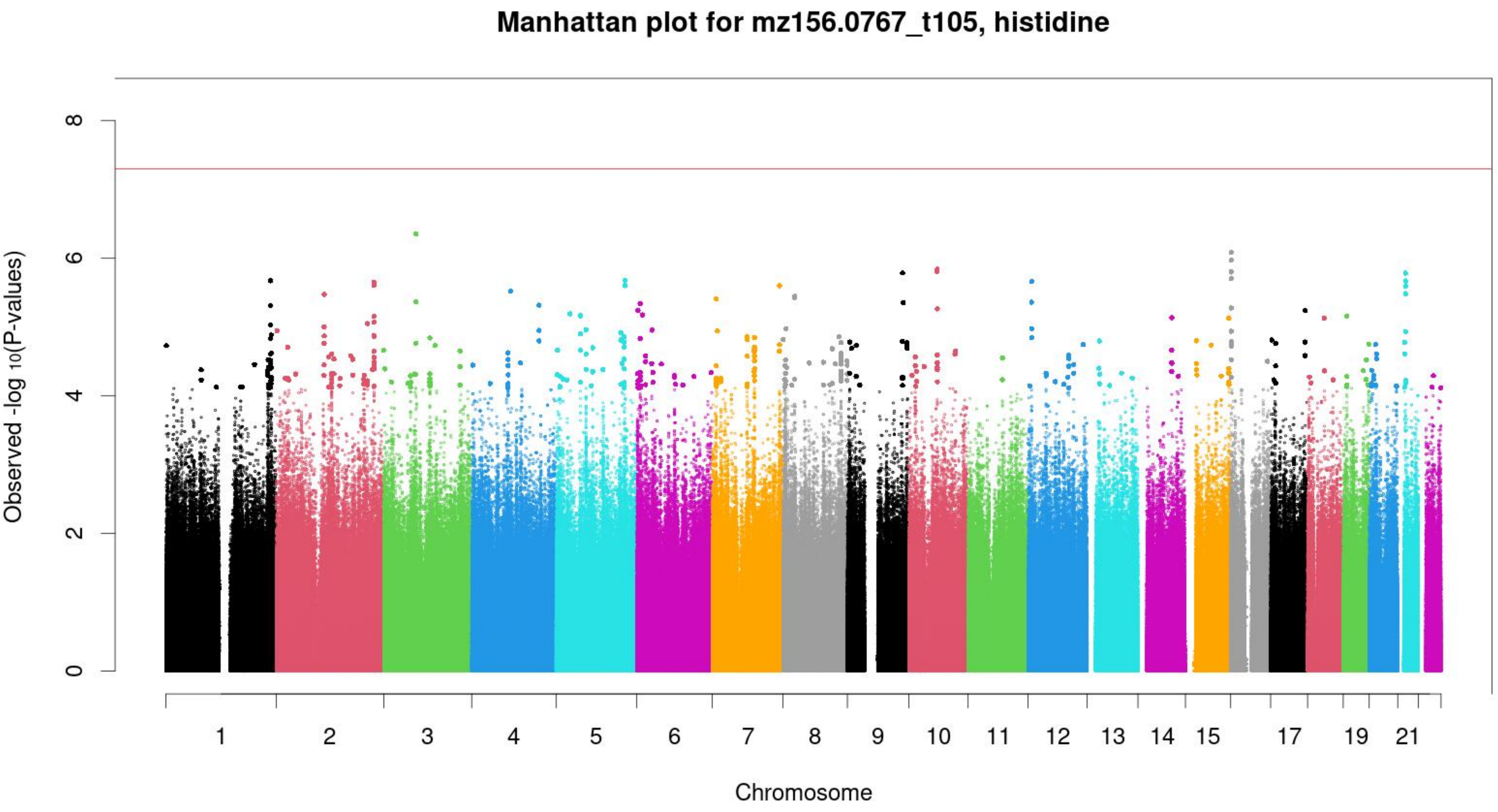
Manhattan plot for mz156.0767_t105, histidine
Observed -log 10(P-values)
0
2
4
6
8
1
2
3
4
5
6
7
8
9
10
11
12
13
14
15
17
19
21
Chromosome

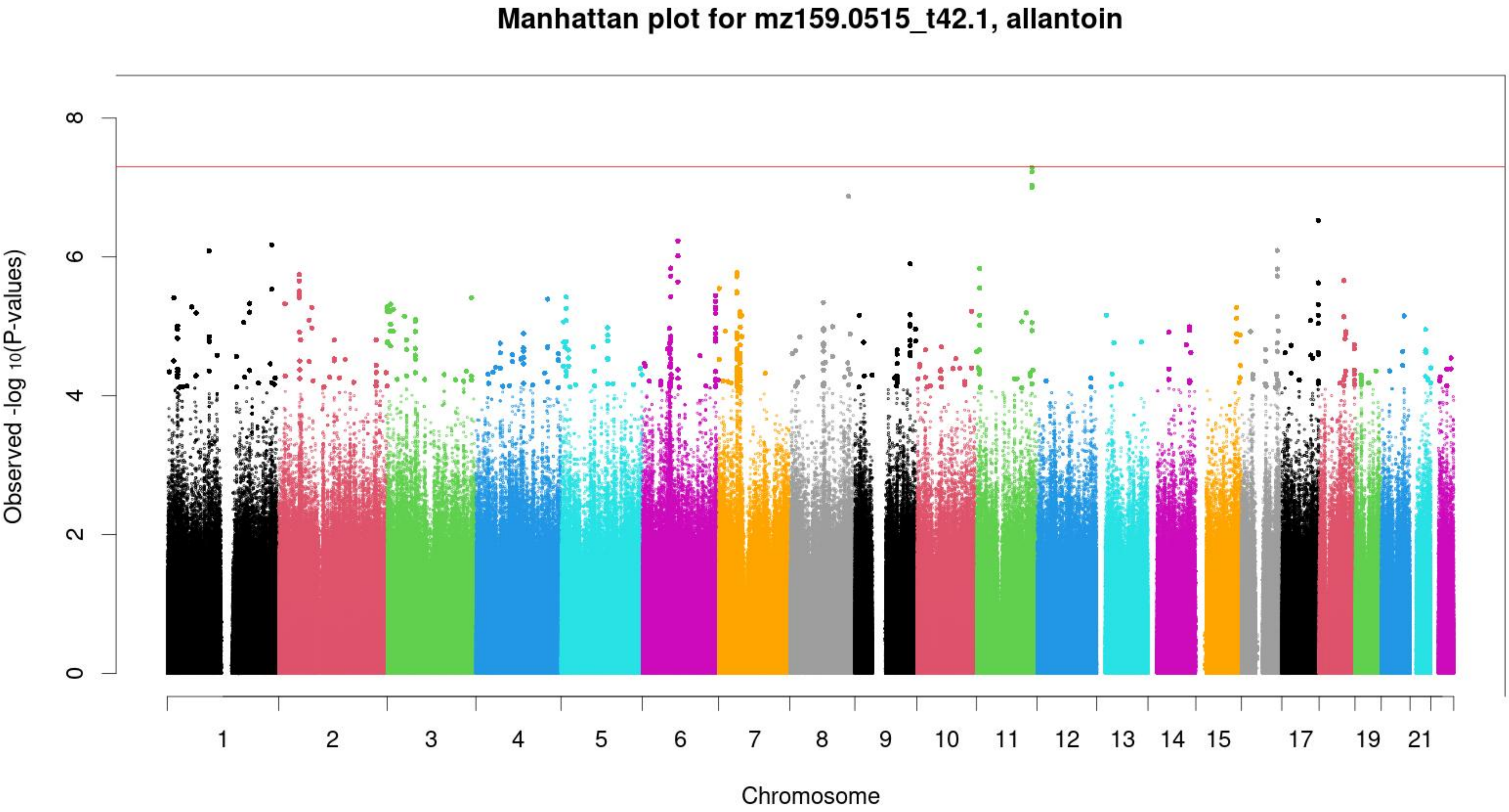

Manhattan plot for mz159.0515_t42.1, allantoin
Observed -log 10(P-values)
0
2
4
6
8
1
2
3
4
5
6
7
8
9
10
11
12
13
14
15
17
19
21
Chromosome

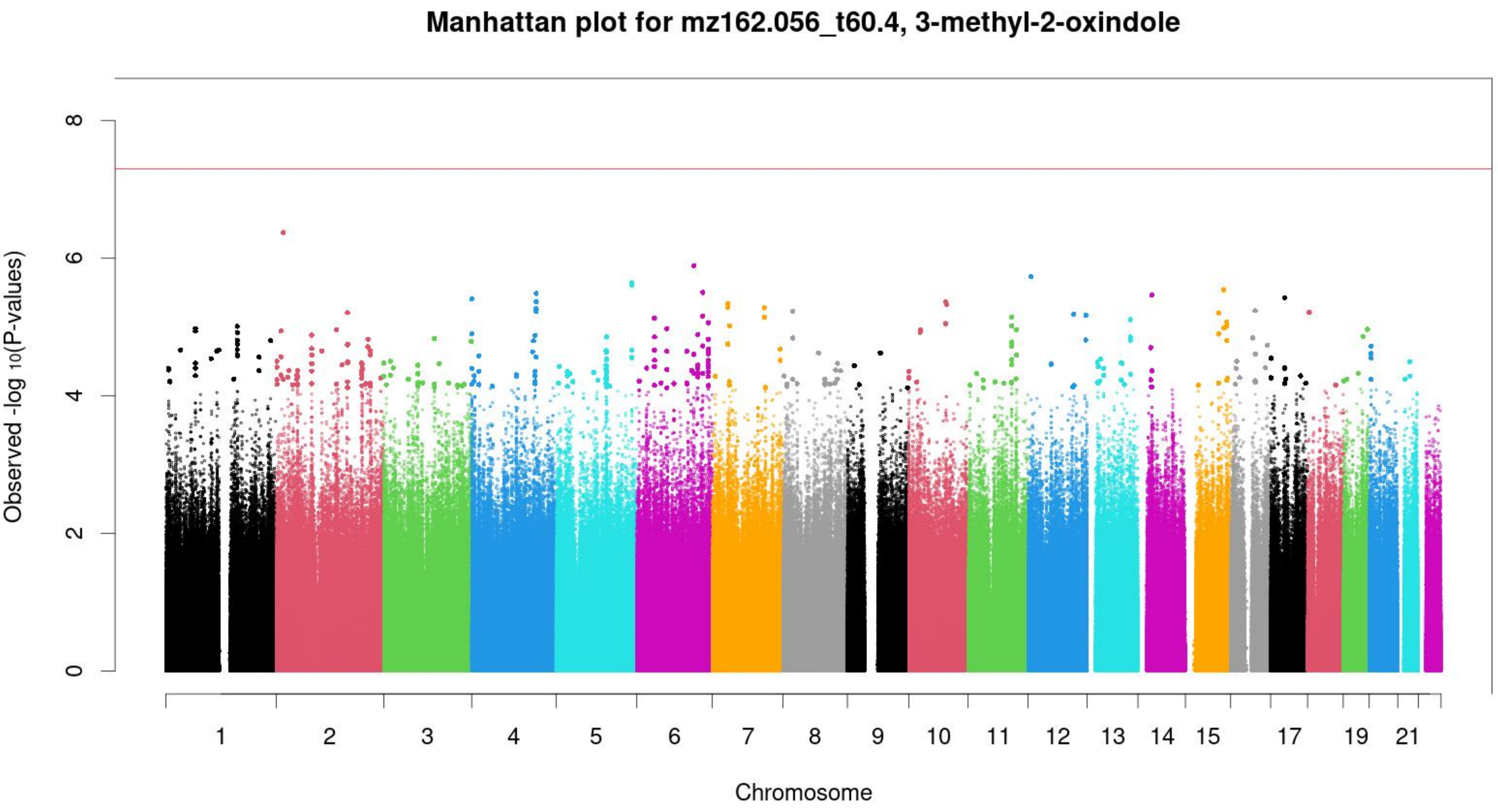

Manhattan plot for mz162.056_t60.4, 3-methyl-2-oxindole
Observed -log 10(P-values)
0
2
4
6
8
1
2
3
4
5
6
7
8
9
10
11
12
13
14
15
17
19
21
Chromosome

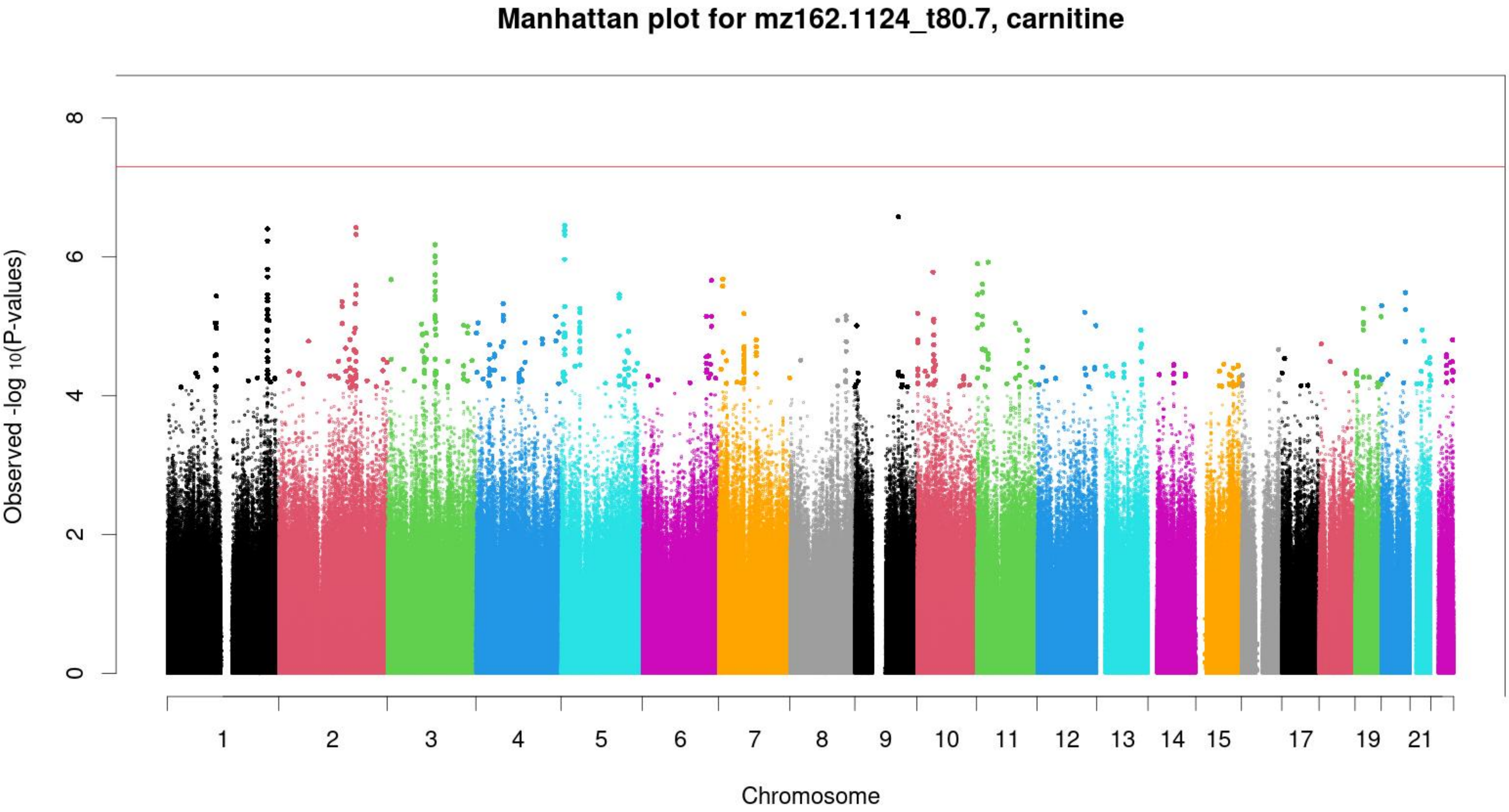
Manhattan plot for mz162.1124_t80.7, carnitine
Observed -log 10(P-values)
0
2
4
6
8
1
2
3
4
5
6
7
8
9
10
11
12
13
14
15
17
19
21
Chromosome

Manhattan plot for mz163.1229_t40.3, (s)-nicotine

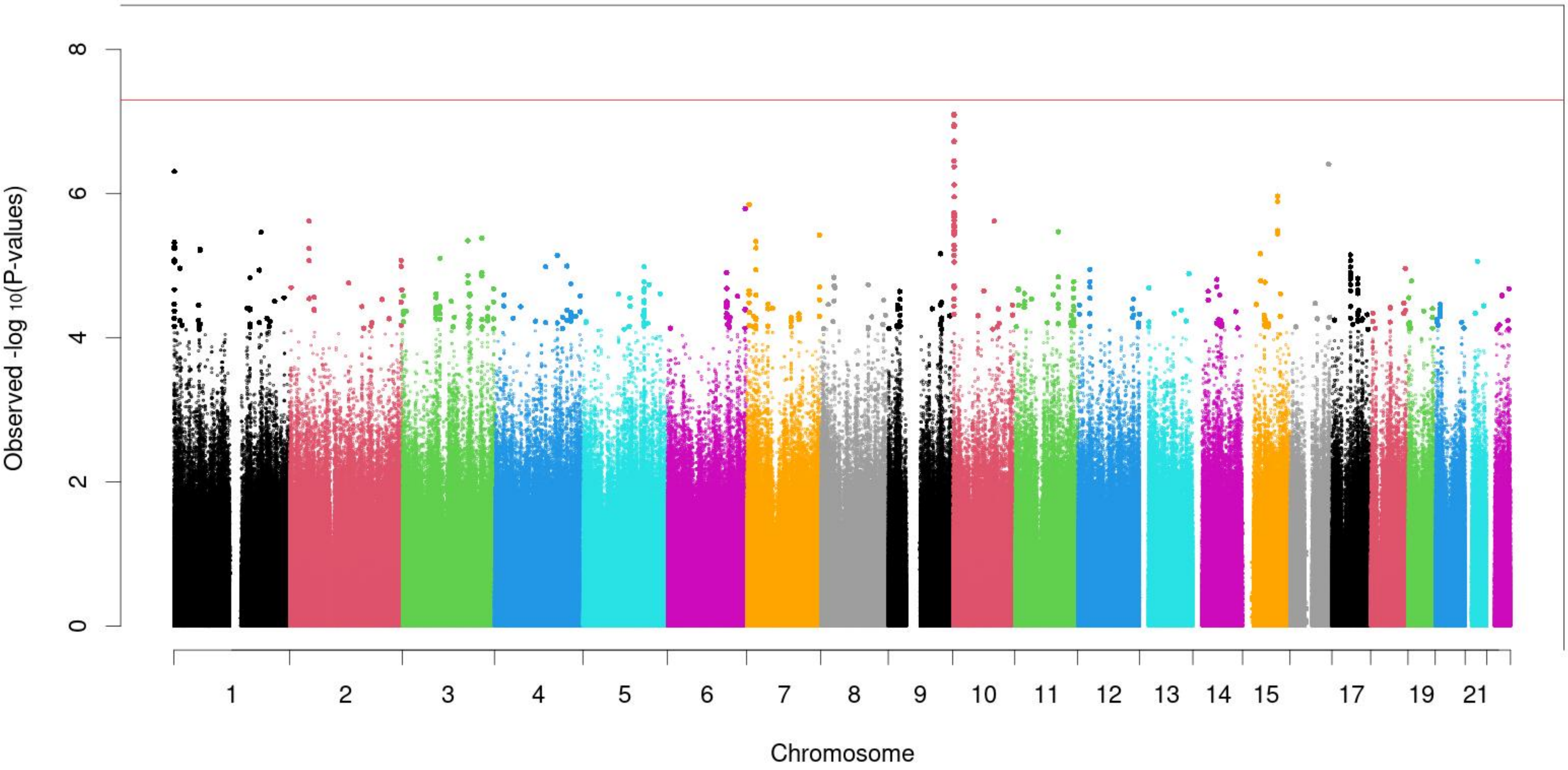

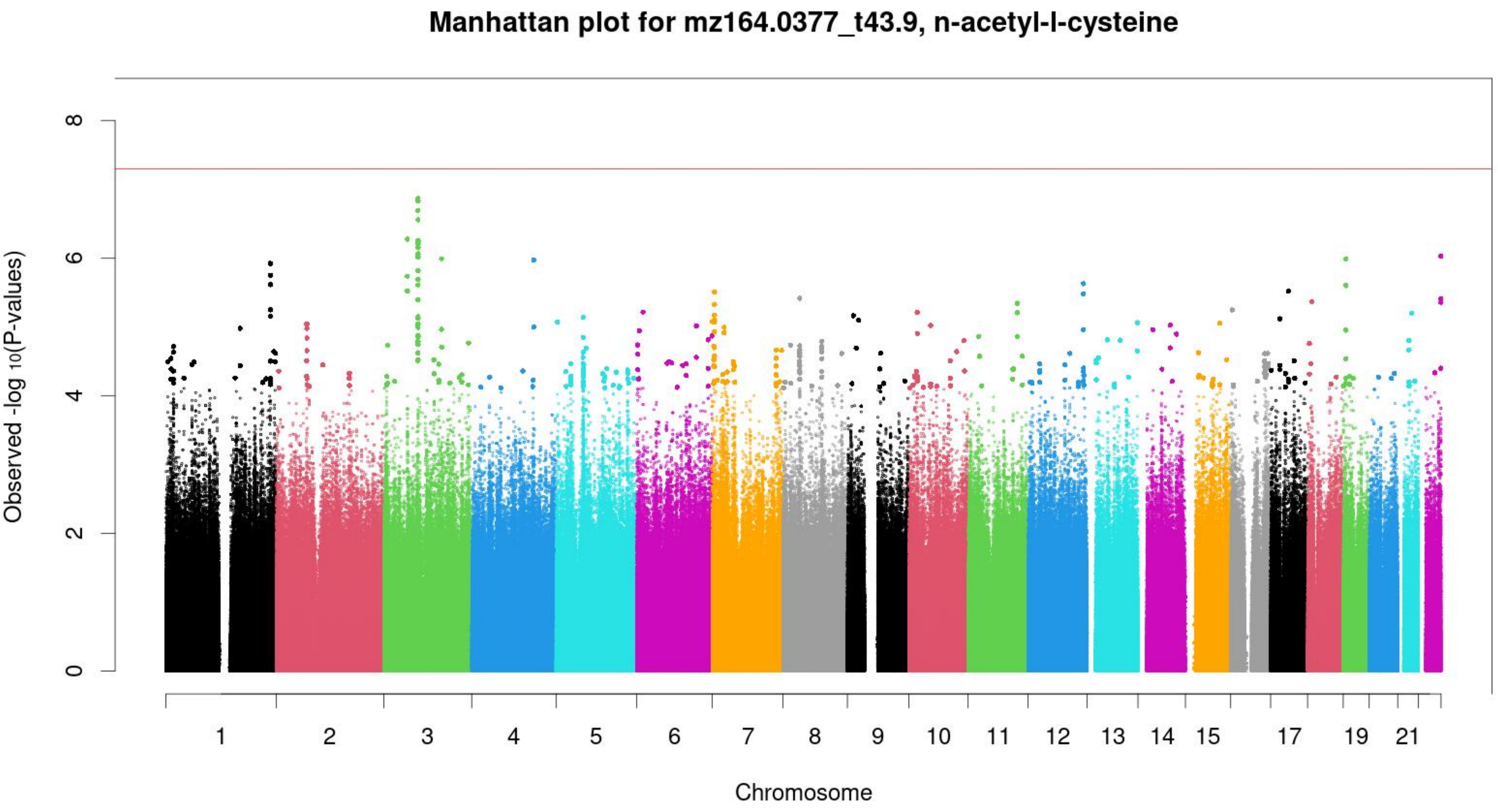
Manhattan plot for mz164.0377_t43.9, n-acetyl-l-cysteine
Observed -log 10(P-values)
0
2
4
6
8
1
2
3
4
5
6
7
8
9
10
11
12
13
14
15
17
19
21
Chromosome

Manhattan plot for mz164.0717_t22.7, phenylalanine

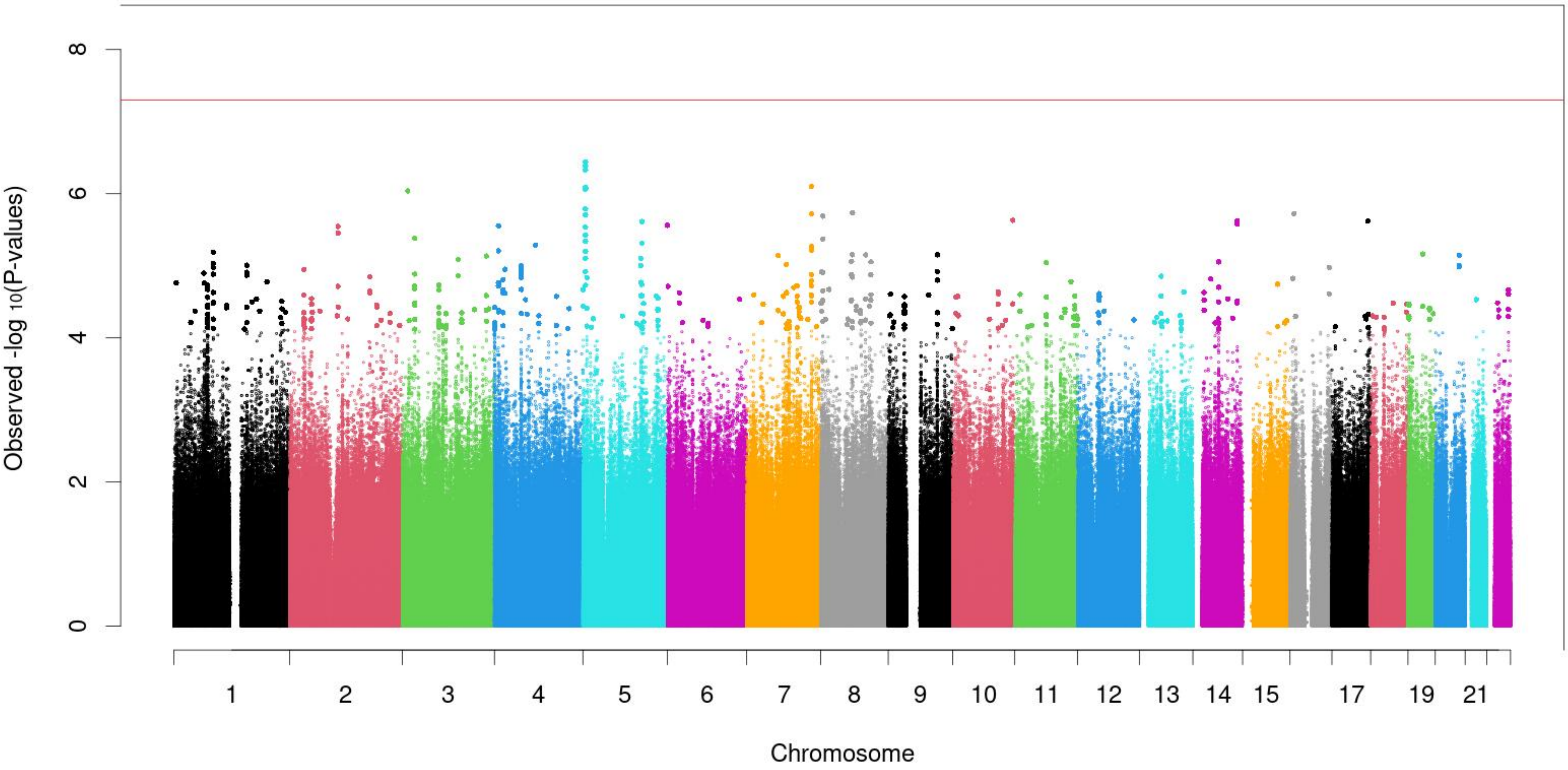

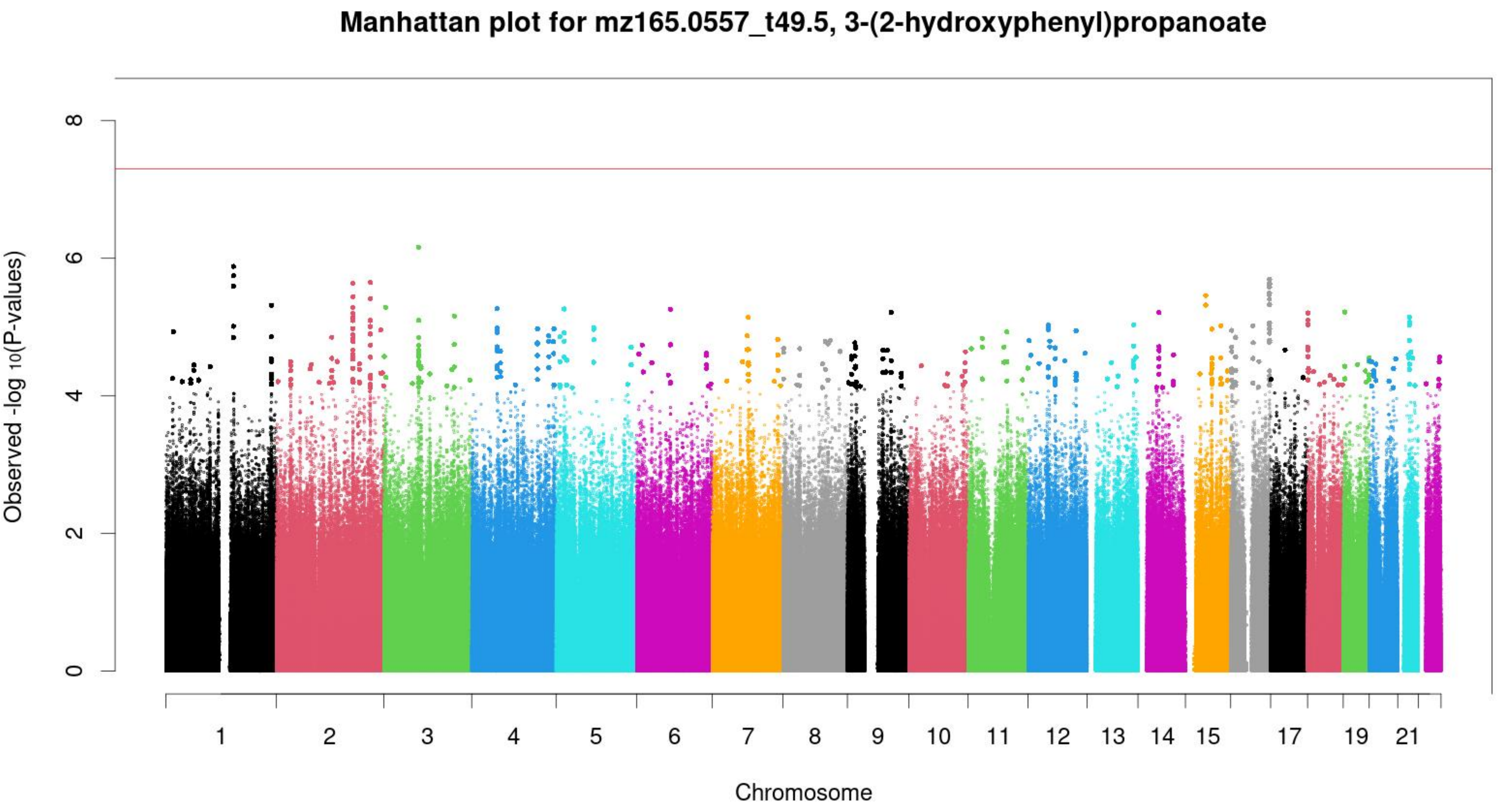

Manhattan plot for mz165.0557_t49.5, 3-(2-hydroxyphenyl)propanoate
Observed -log 10(P-values)
0
2
4
6
8
Chromosome
1
2
3
4
5
6
7
8
9
10
11
12
13
14
15
17
19
21

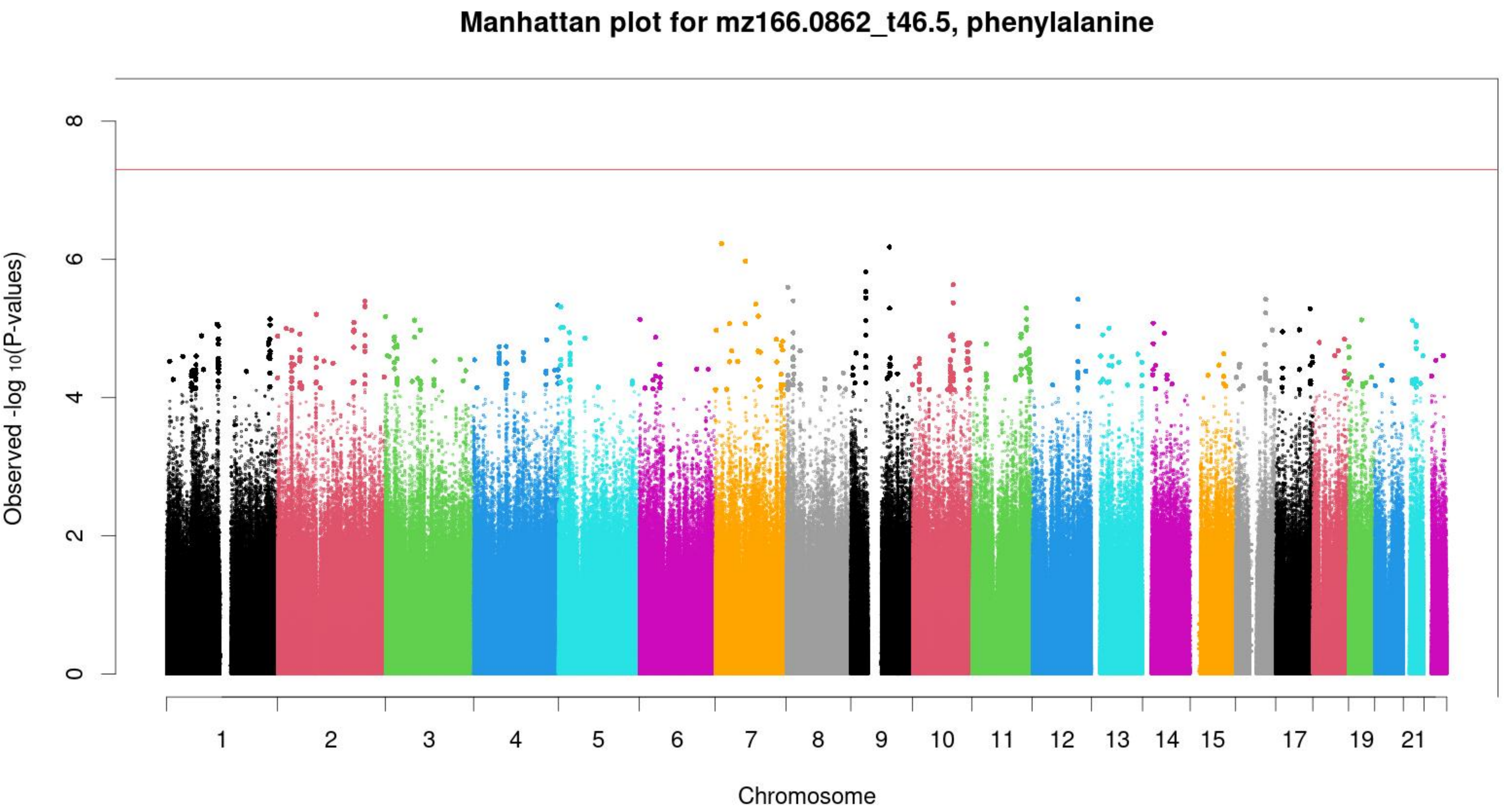
Manhattan plot for mz166.0862_t46.5, phenylalanine
Observed -log 10(P-values)
0
2
4
6
8
1
2
3
4
5
6
7
8
9
10
11
12
13
14
15
17
19
21
Chromosome

Manhattan plot for mz167.0211_t23.9, urate

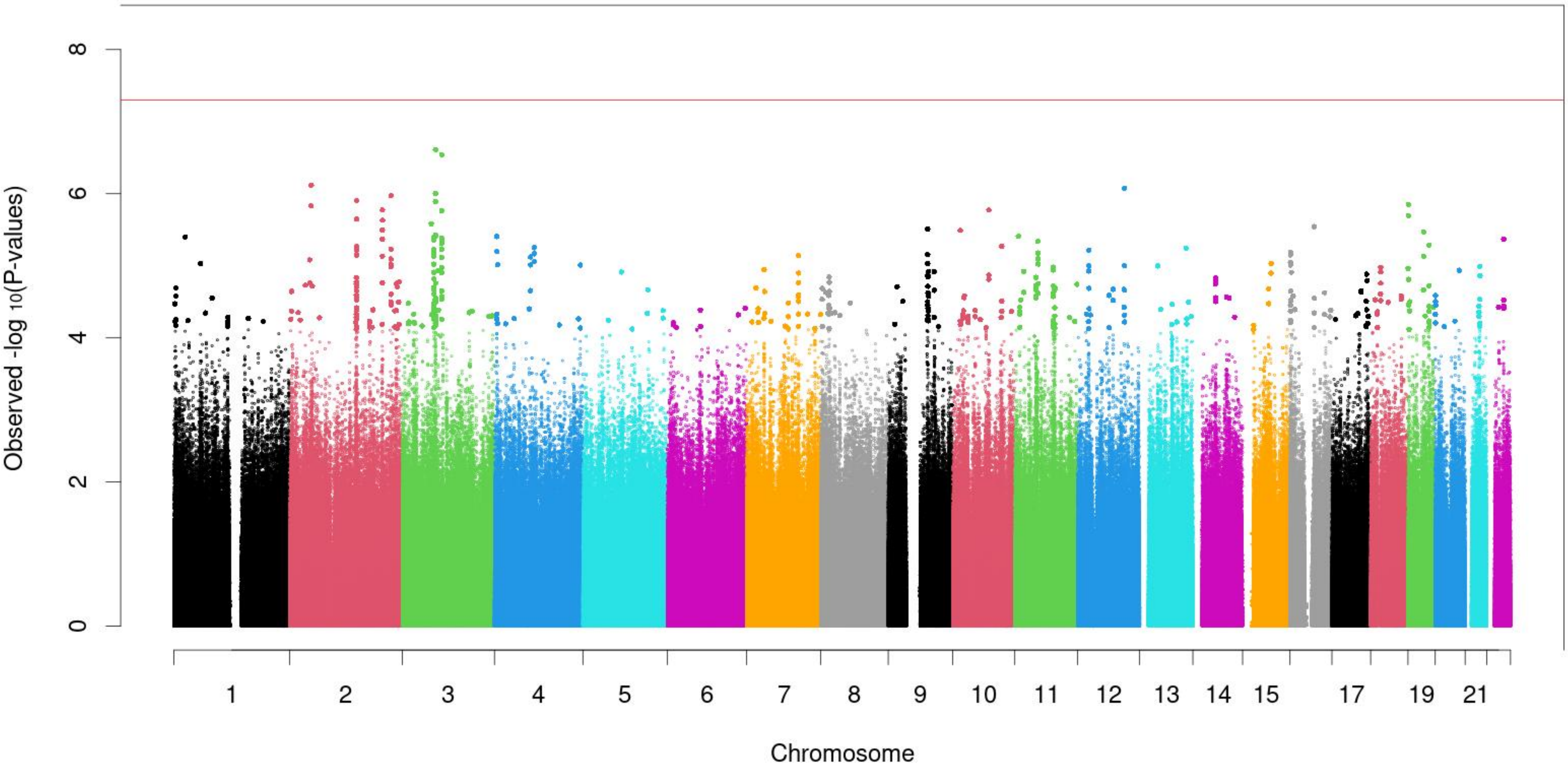

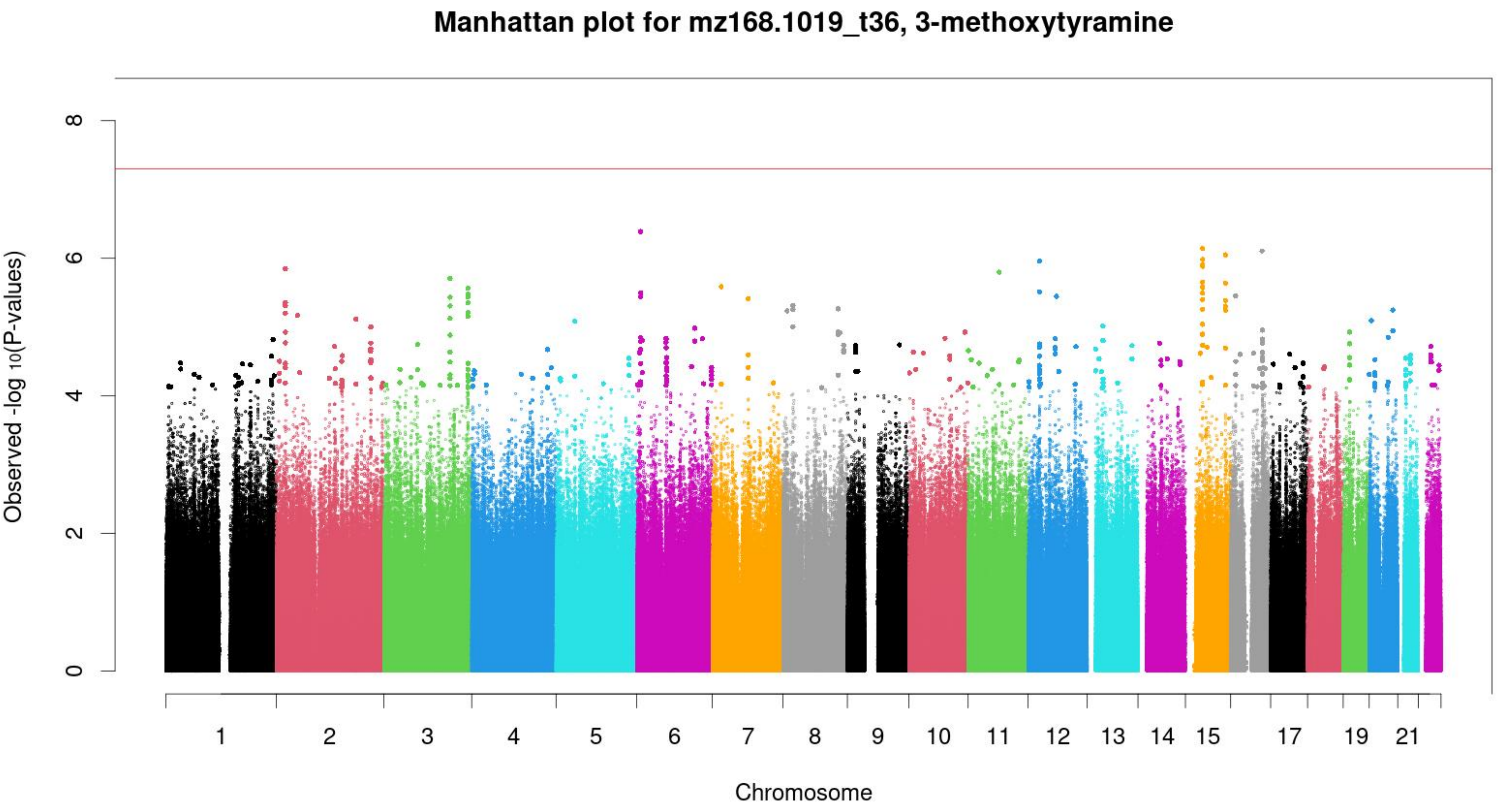

Manhattan plot for mz168.1019_t36, 3-methoxytyramine
Observed -log 10(P-values)
0
2
4
6
8
1
2
3
4
5
6
7
8
9
10
11
12
13
14
15
17
19
21
Chromosome

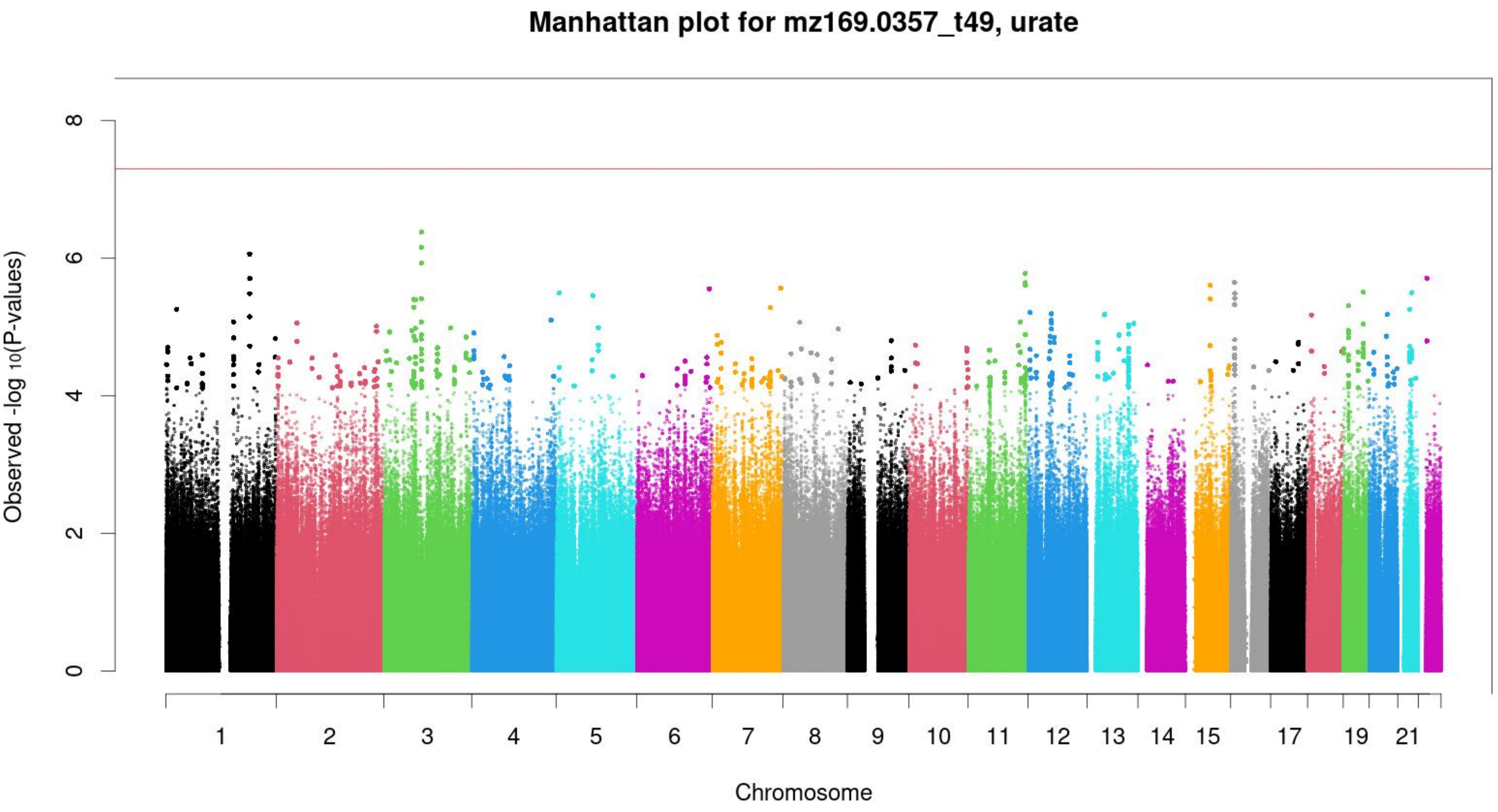
Manhattan plot for mz169.0357_t49, urate
Observed -log 10(P-values)
0
2
4
6
8
1
2
3
4
5
6
7
8
9
10
11
12
13
14
15
17
19
21
Chromosome

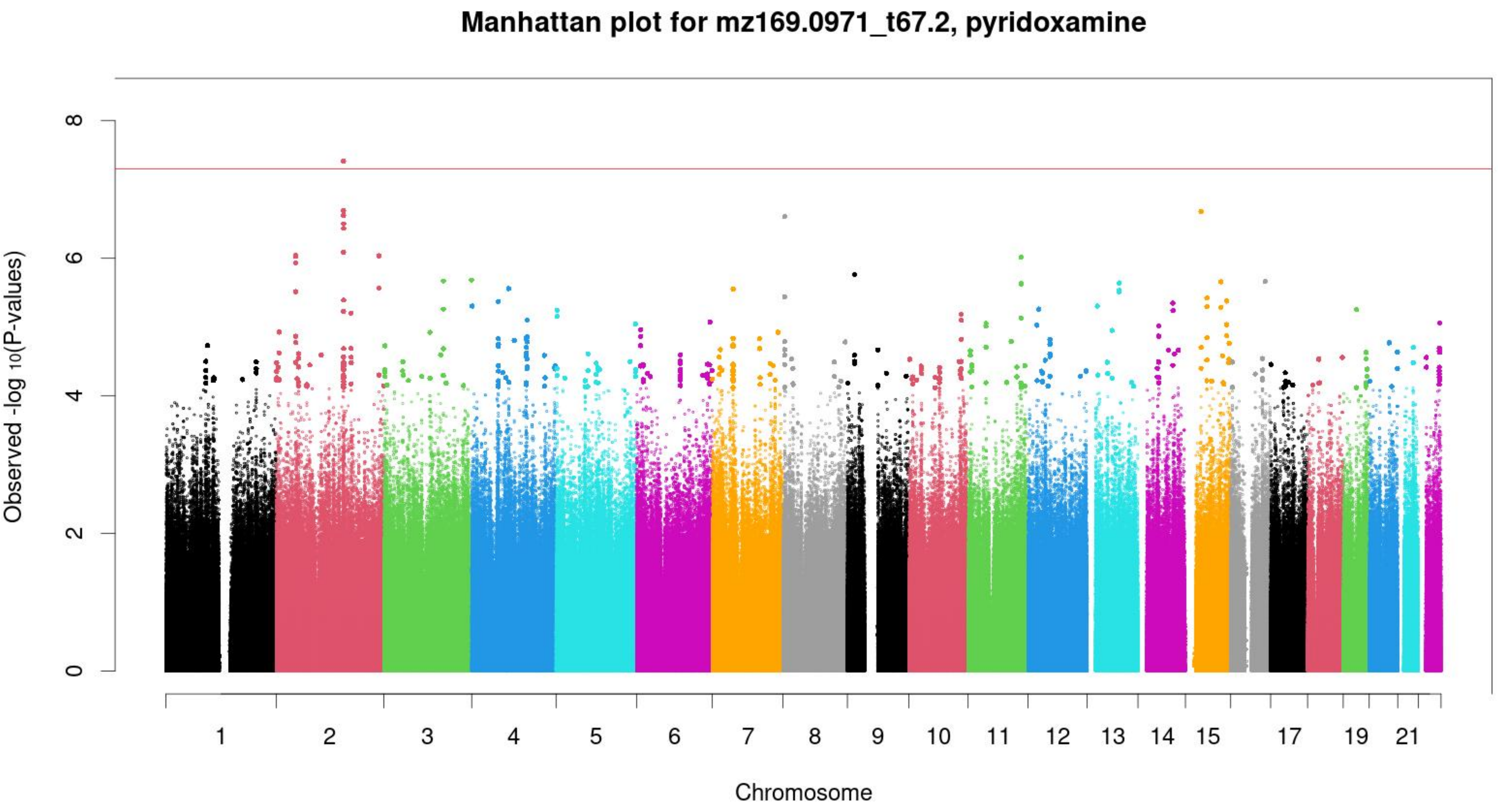

Manhattan plot for mz169.0971_t67.2, pyridoxamine
Observed -log 10(P-values)
0
2
4
6
8
1
2
3
4
5
6
7
8
9
10
11
12
13
14
15
17
19
21
Chromosome

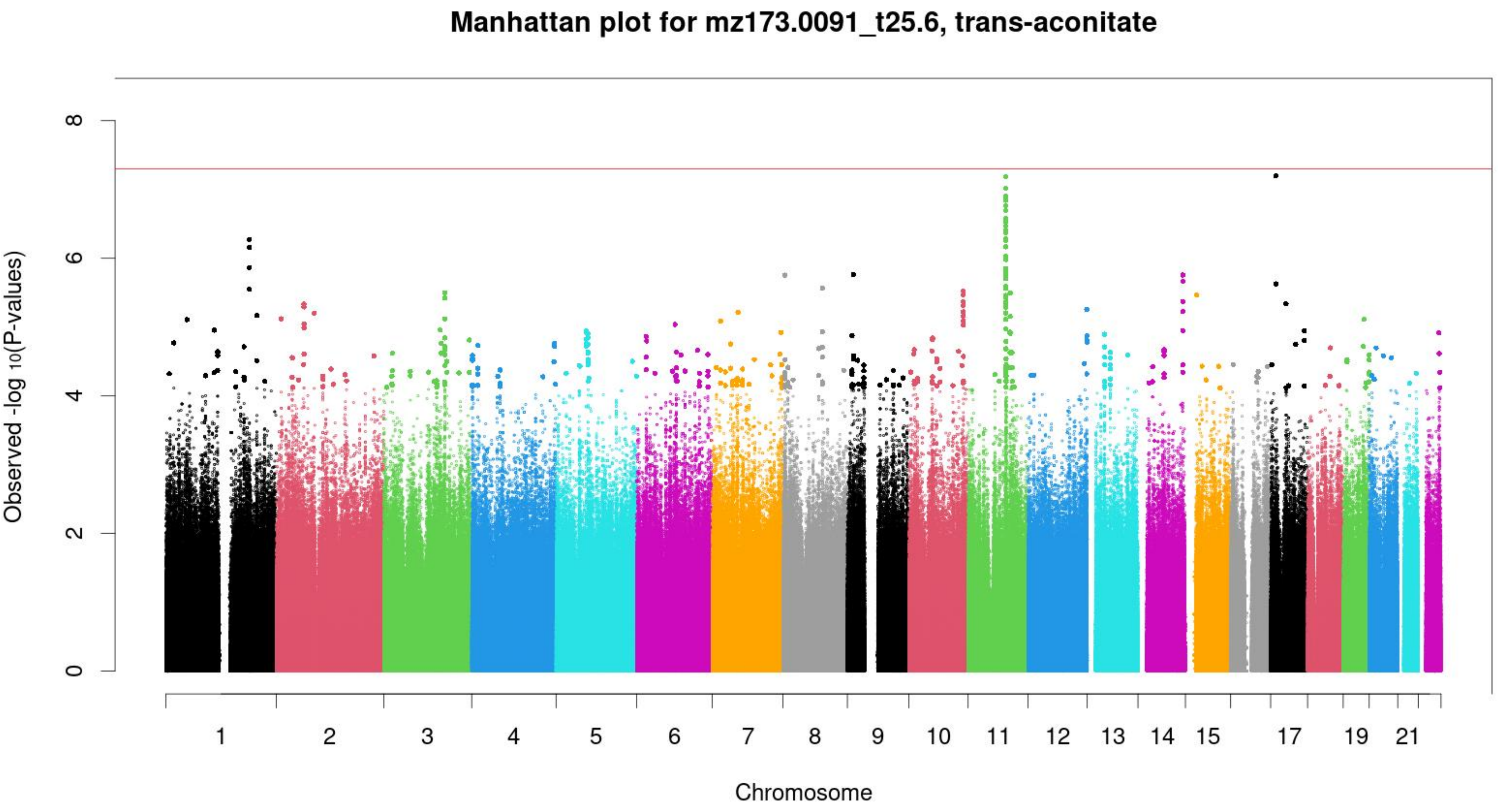
Manhattan plot for mz173.0091_t25.6, trans-aconitate
Observed -log 10(P-values)
0
2
4
6
8
1
2
3
4
5
6
7
8
9
10
11
12
13
14
15
17
19
21
Chromosome

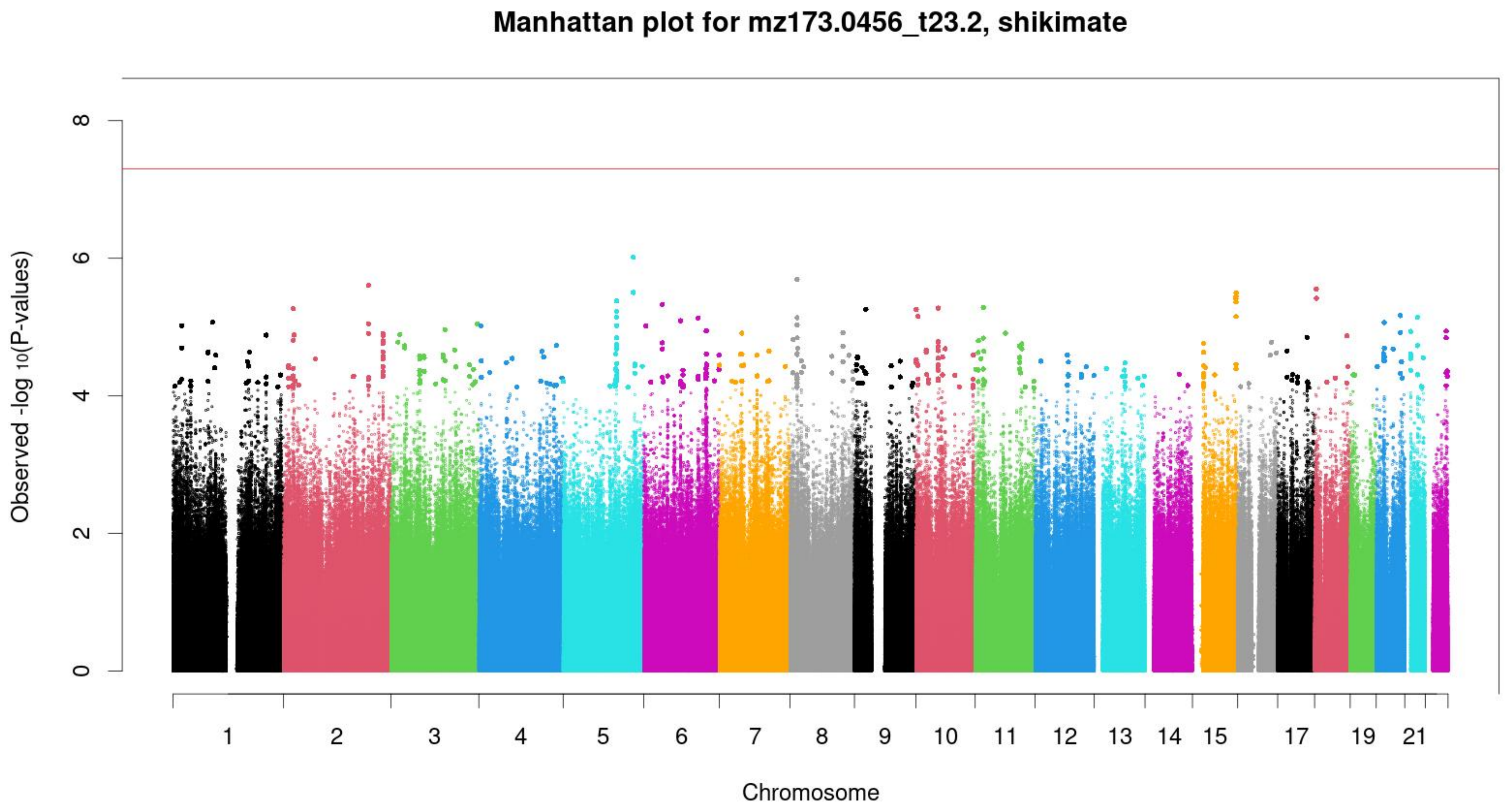

Manhattan plot for mz173.0456_t23.2, shikimate
Observed -log 10(P-values)
0
2
4
6
8
1
2
3
4
5
6
7
8
9
10
11
12
13
14
15
17
19
21
Chromosome

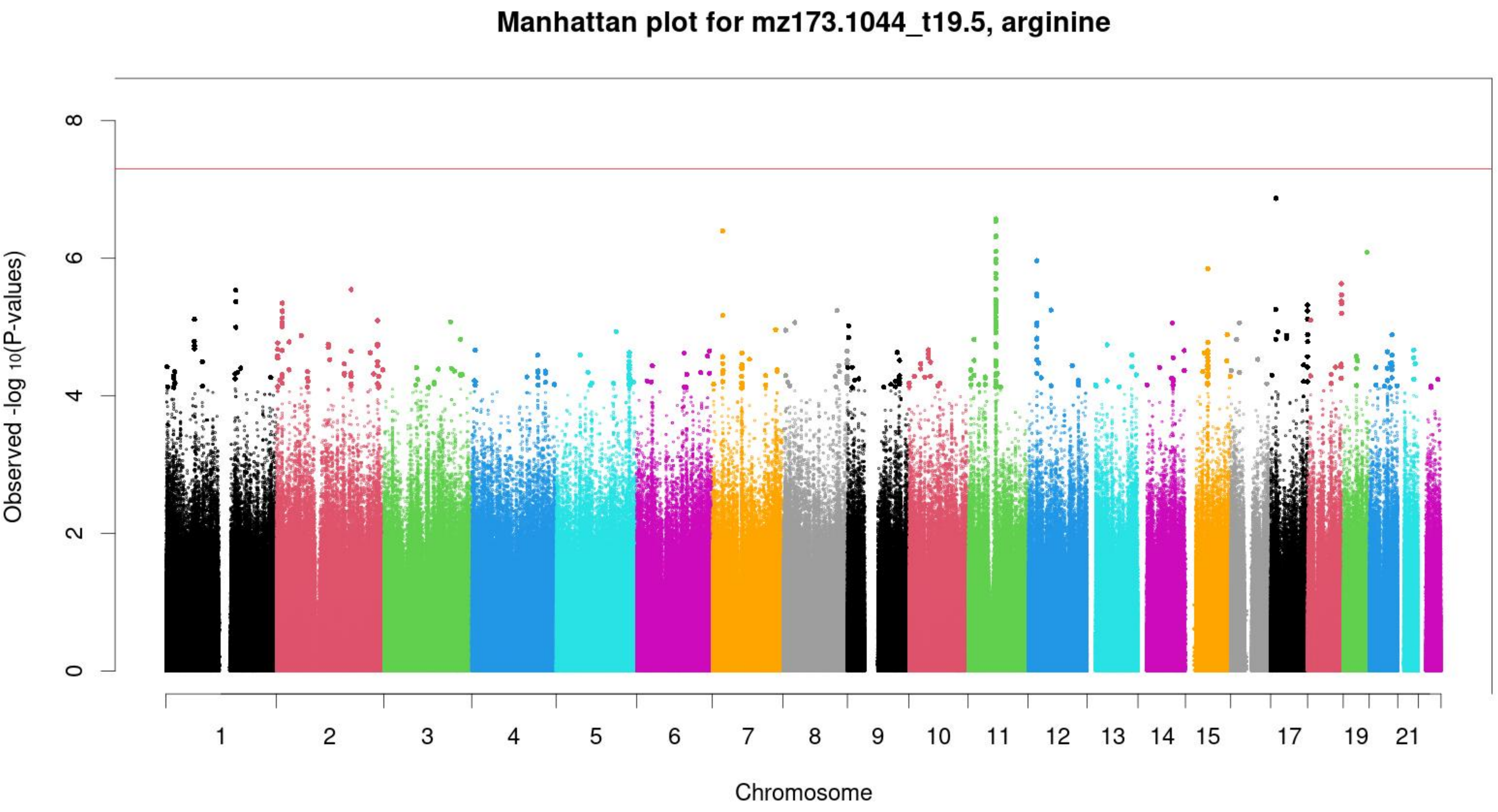
Manhattan plot for mz173.1044_t19.5, arginine
Observed -log 10(P-values)
0
2
4
6
8
1
2
3
4
5
6
7
8
9
10
11
12
13
14
15
17
19
21
Chromosome

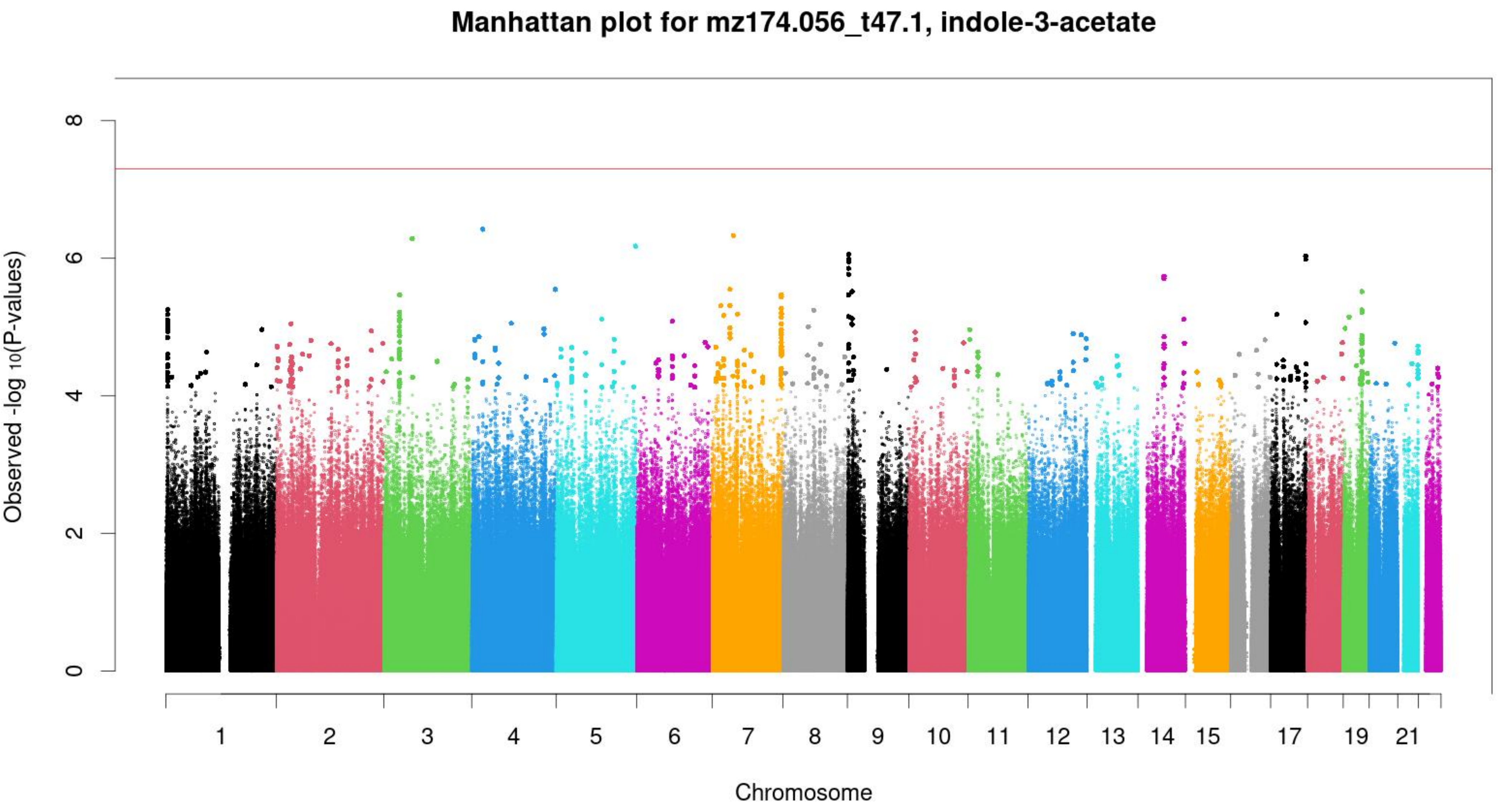
Manhattan plot for mz174.056_t47.1, indole-3-acetate
Observed -log 10(P-values)
0
2
4
6
8
1
2
3
4
5
6
7
8
9
10
11
12
13
14
15
17
19
21
Chromosome

Manhattan plot for mz174.0884_t21.1, citrulline

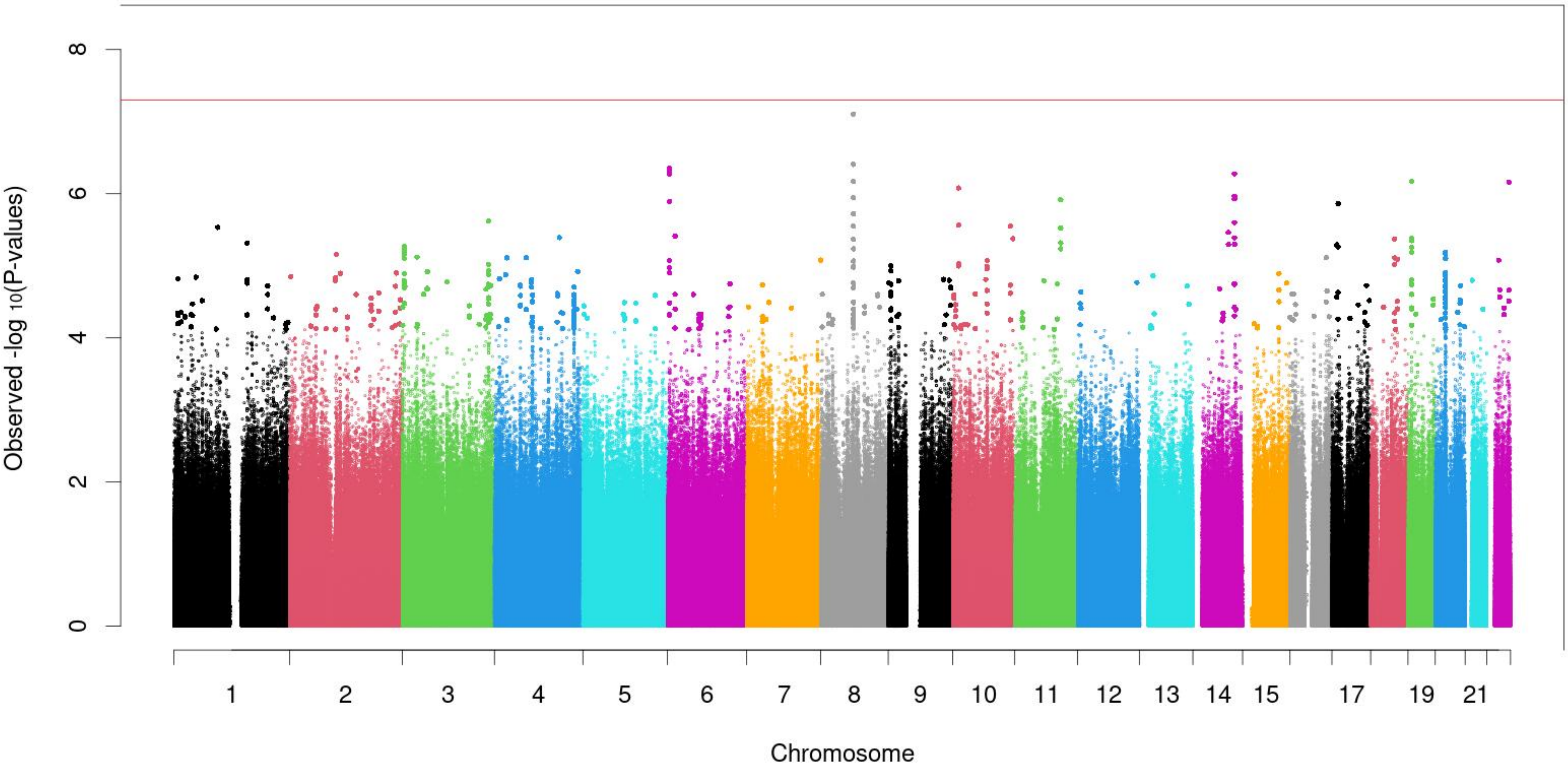

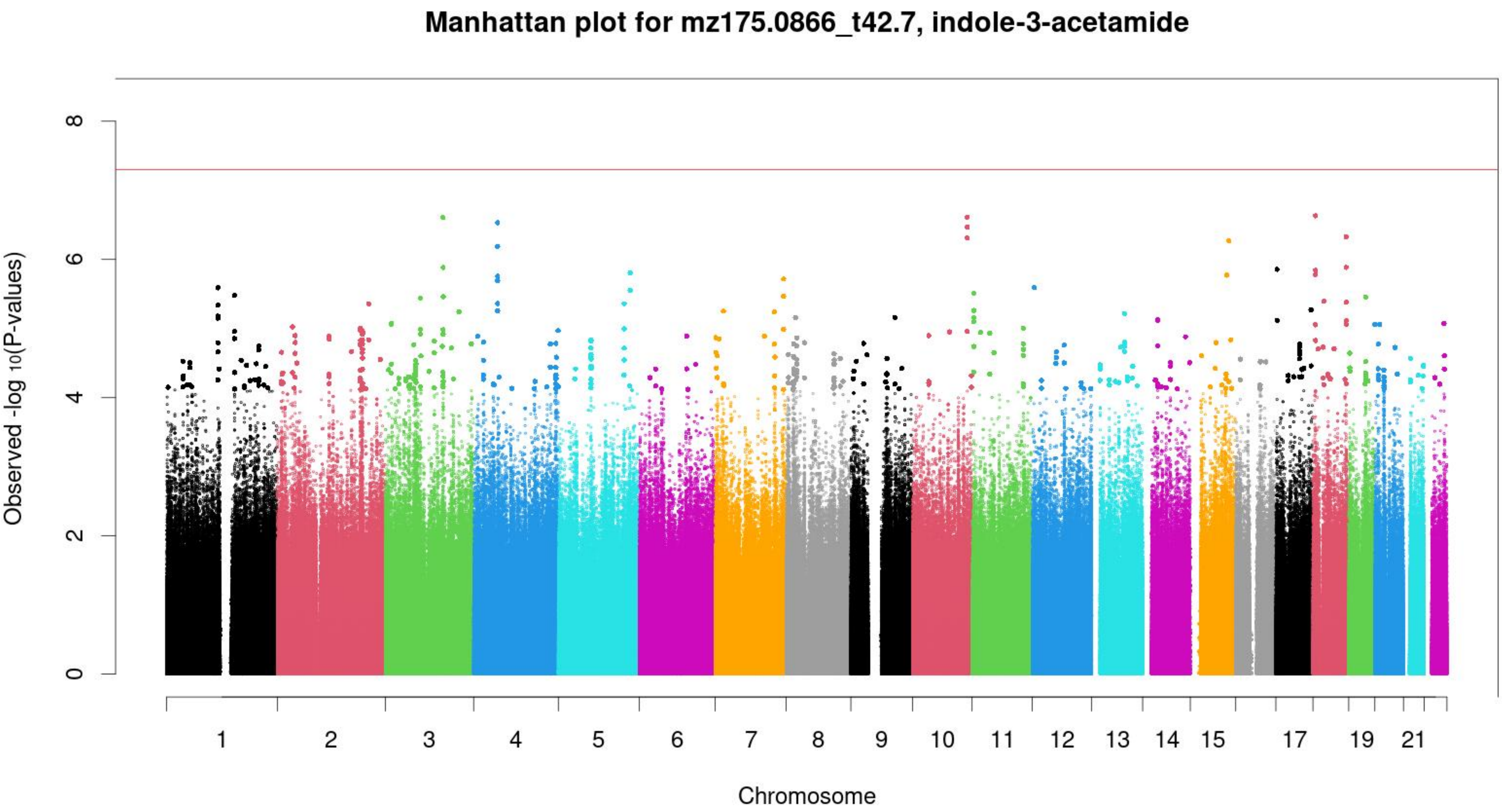

Manhattan plot for mz175.0866_t42.7, indole-3-acetamide
Observed -log 10(P-values)
0
2
4
6
8
1
2
3
4
5
6
7
8
9
10
11
12
13
14
15
17
19
21
Chromosome

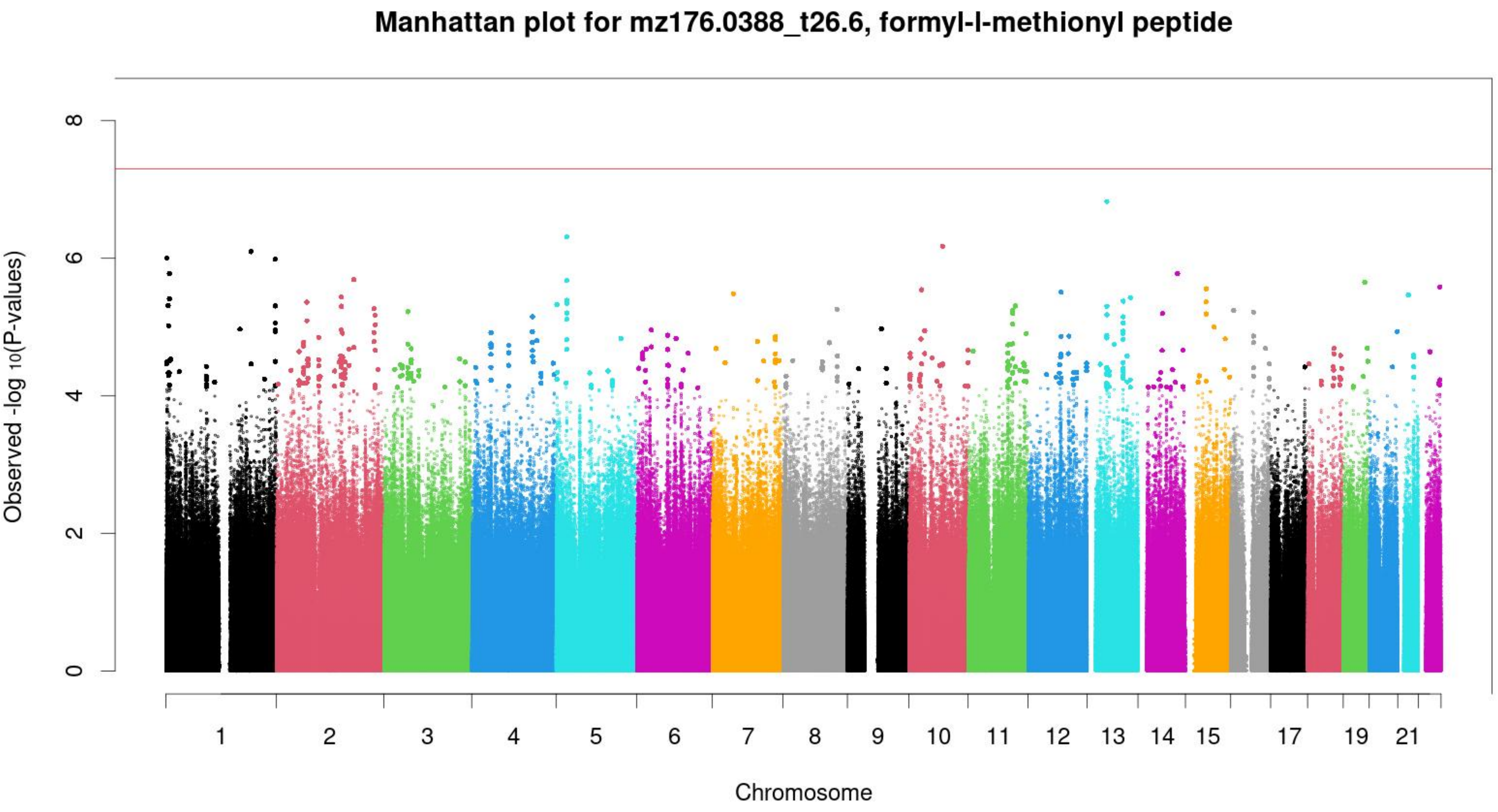
Manhattan plot for mz176.0388_t26.6, formyl-l-methionyl peptide
Observed -log 10(P-values)
0
2
4
6
8
Chromosome
1
2
3
4
5
6
7
8
9
10
11
12
13
14
15
17
19
21

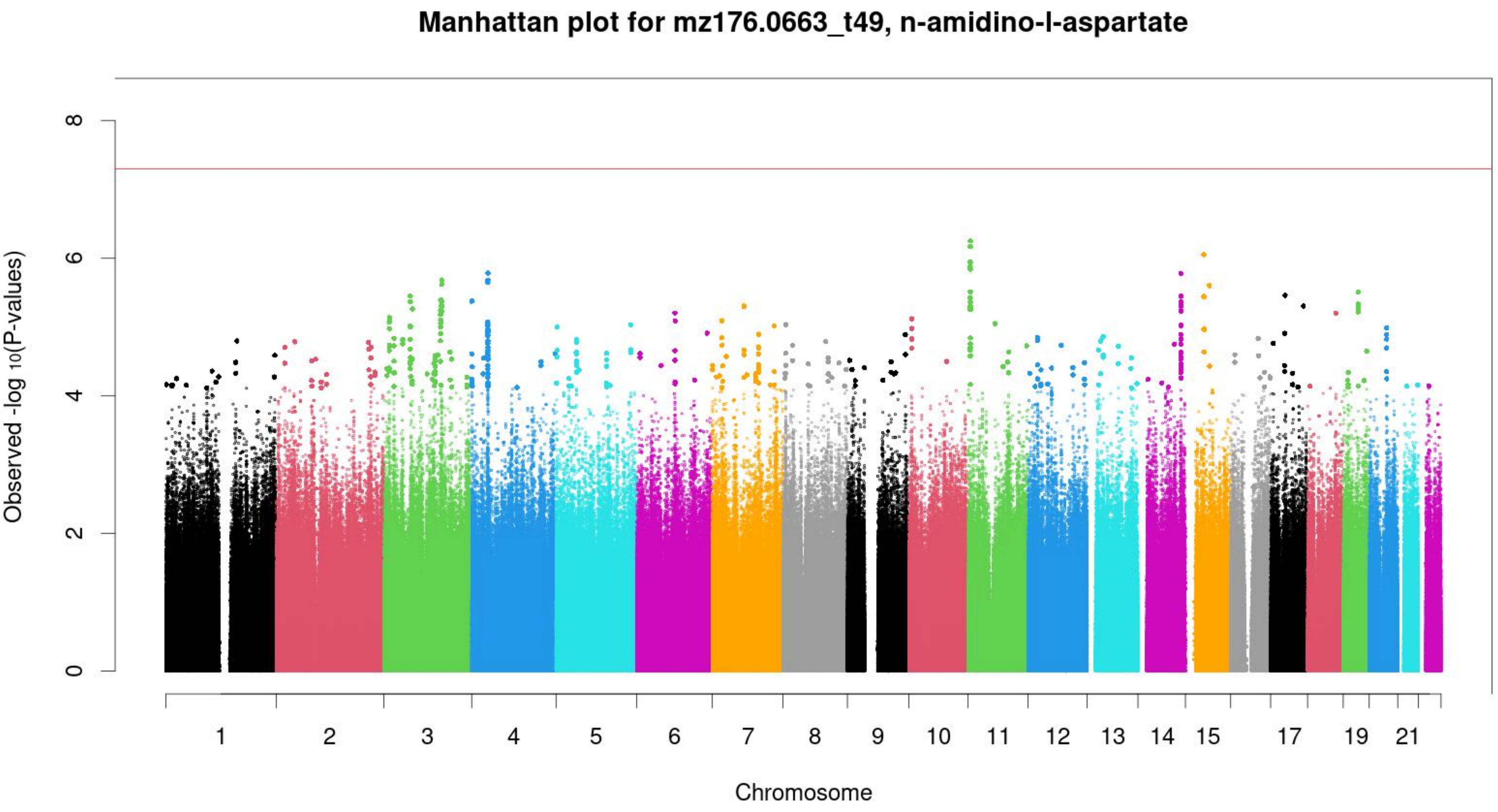
Manhattan plot for mz176.0663_t49, n-amidino-l-aspartate
Observed -log 10(P-values)
0
2
4
6
8
Chromosome
1
2
3
4
5
6
7
8
9
10
11
12
13
14
15
17
19
21

Manhattan plot for mz176.1029_t90.6, citrulline

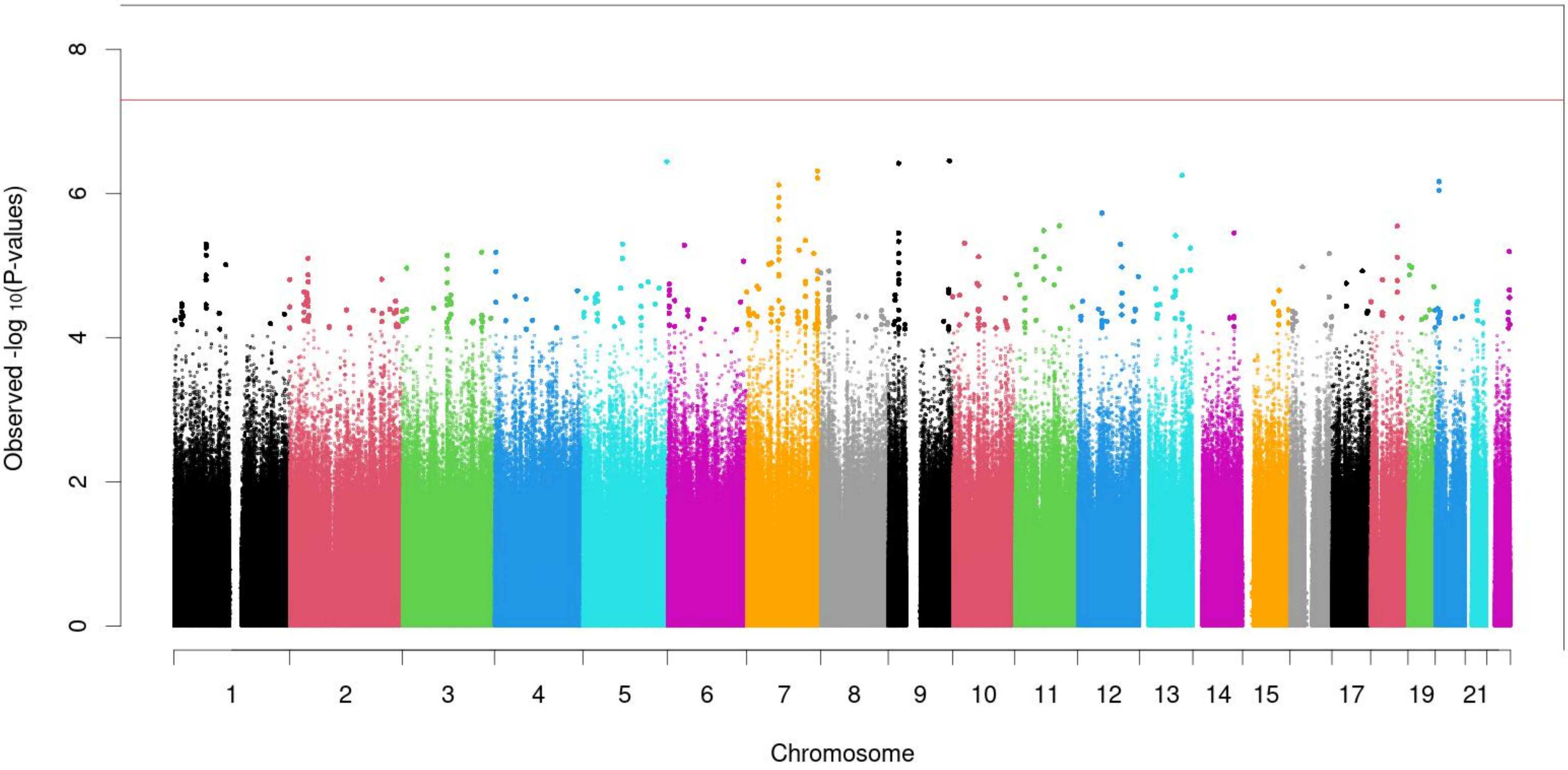

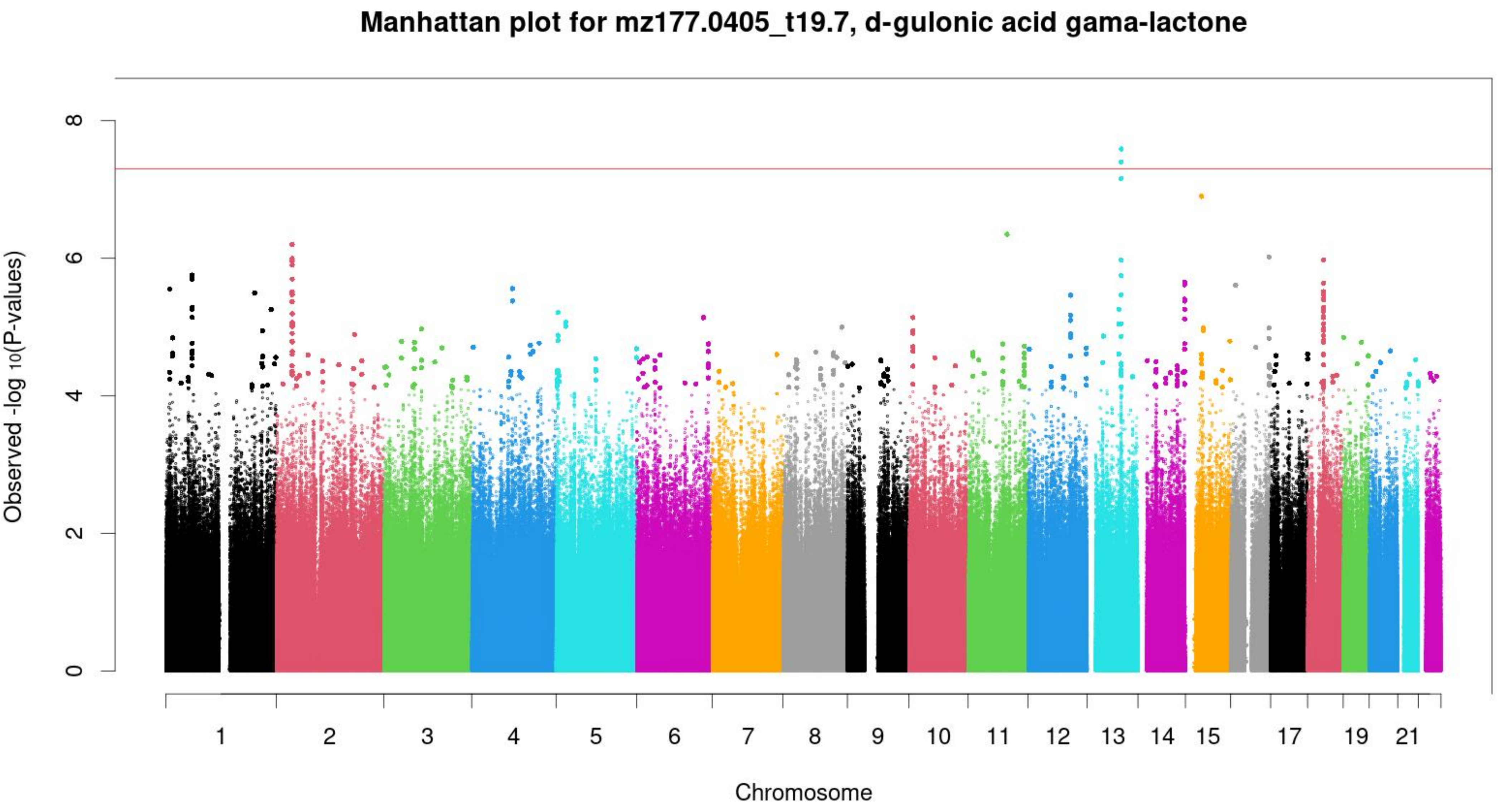
Manhattan plot for mz177.0405_t19.7, d-gulonic acid gama-lactone
Observed -log 10(P-values)
0
2
4
6
8
1
2
3
4
5
6
7
8
9
10
11
12
13
14
15
17
19
21
Chromosome

Manhattan plot for mz178.051_t30.3, hippurate

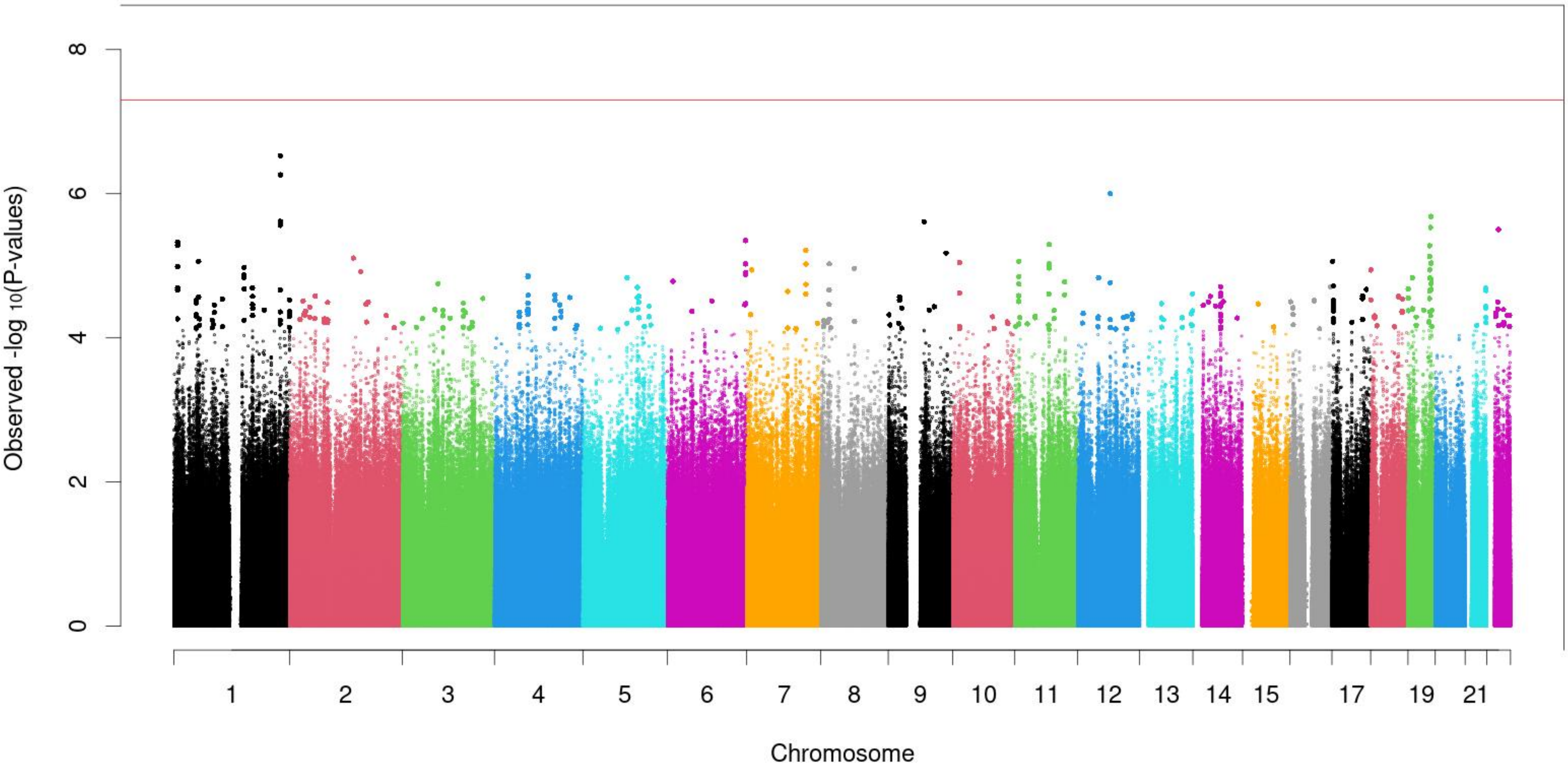

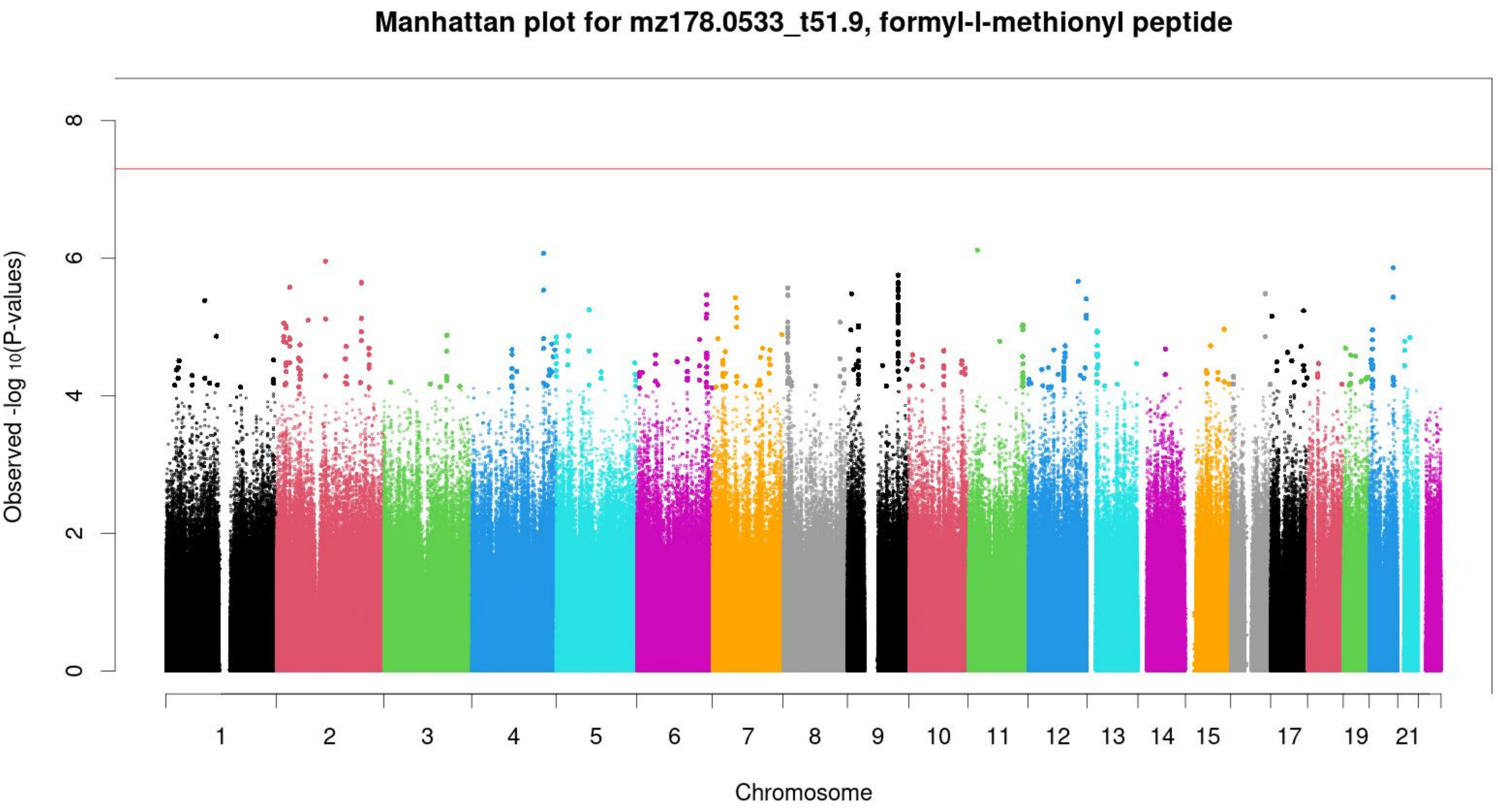
Manhattan plot for mz178.0533_t51.9, formyl-l-methionyl peptide
Observed -log 10(P-values)
0
2
4
6
8
1
2
3
4
5
6
7
8
9
10
11
12
13
14
15
17
19
21
Chromosome

Manhattan plot for mz179.035_t25.5, caffeate

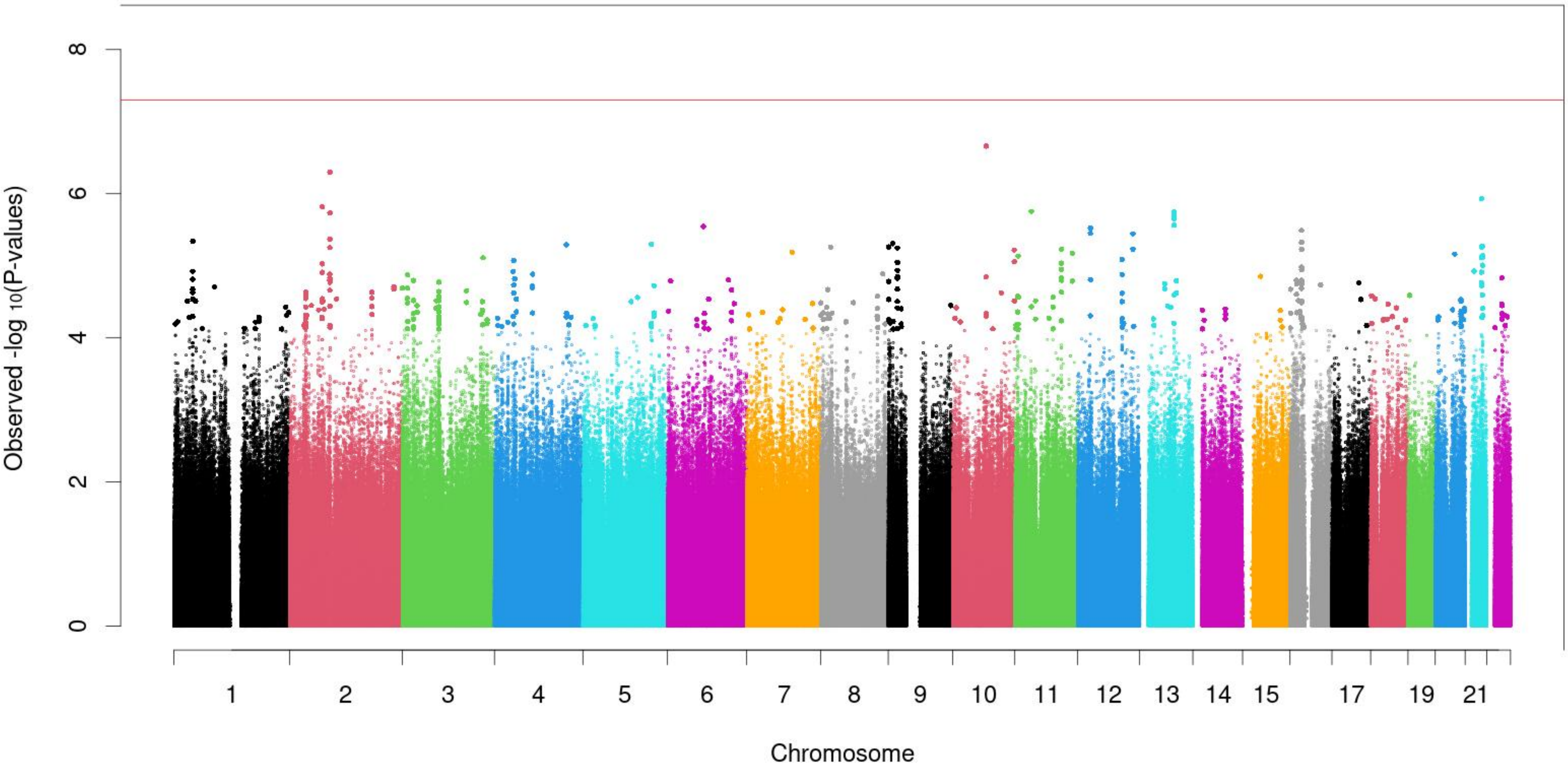

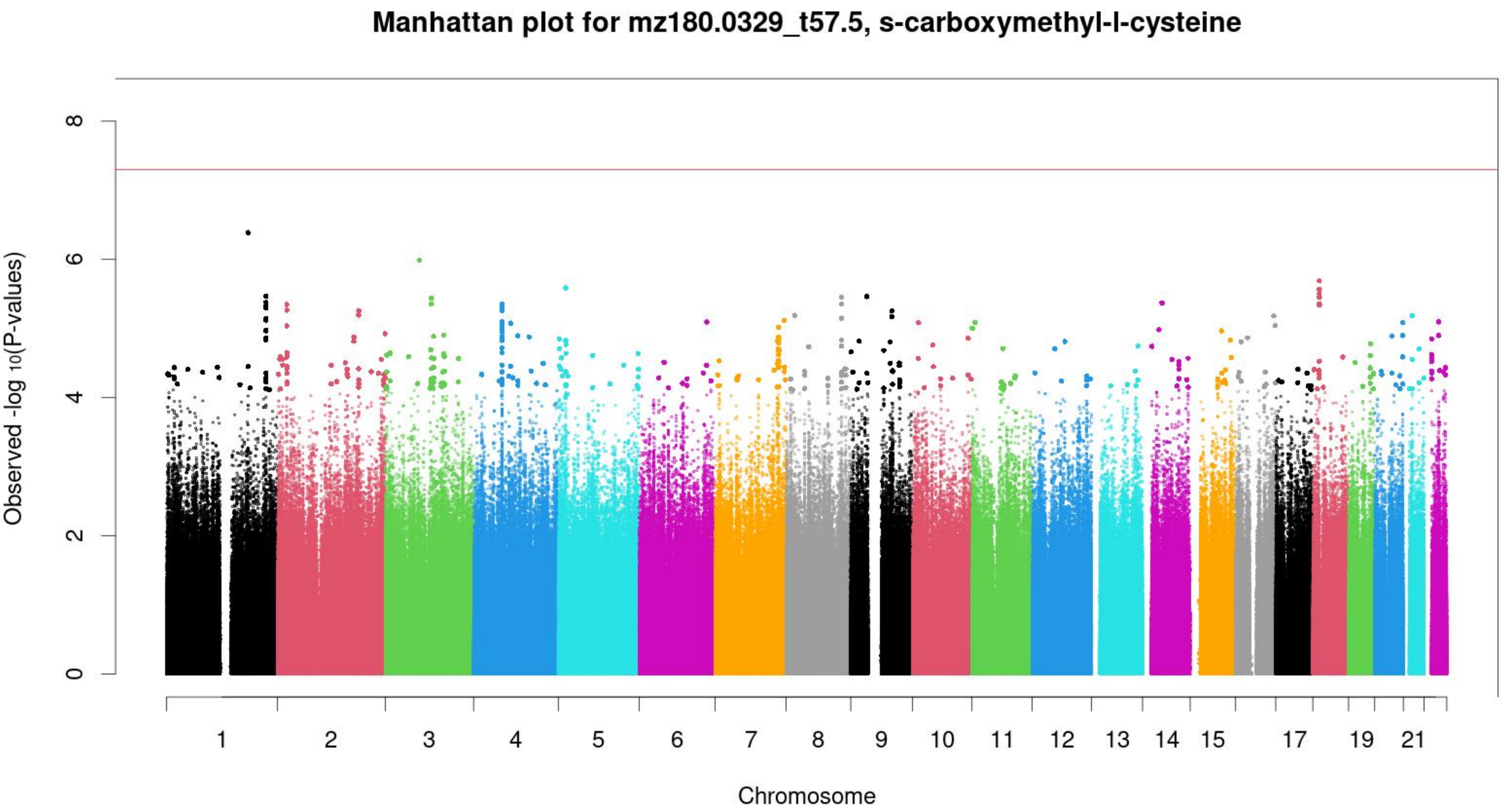
Manhattan plot for mz180.0329_t57.5, s-carboxymethyl-l-cysteine
Observed -log 10(P-values)
0
2
4
6
8
1
2
3
4
5
6
7
8
9
10
11
12
13
14
15
17
19
21
Chromosome

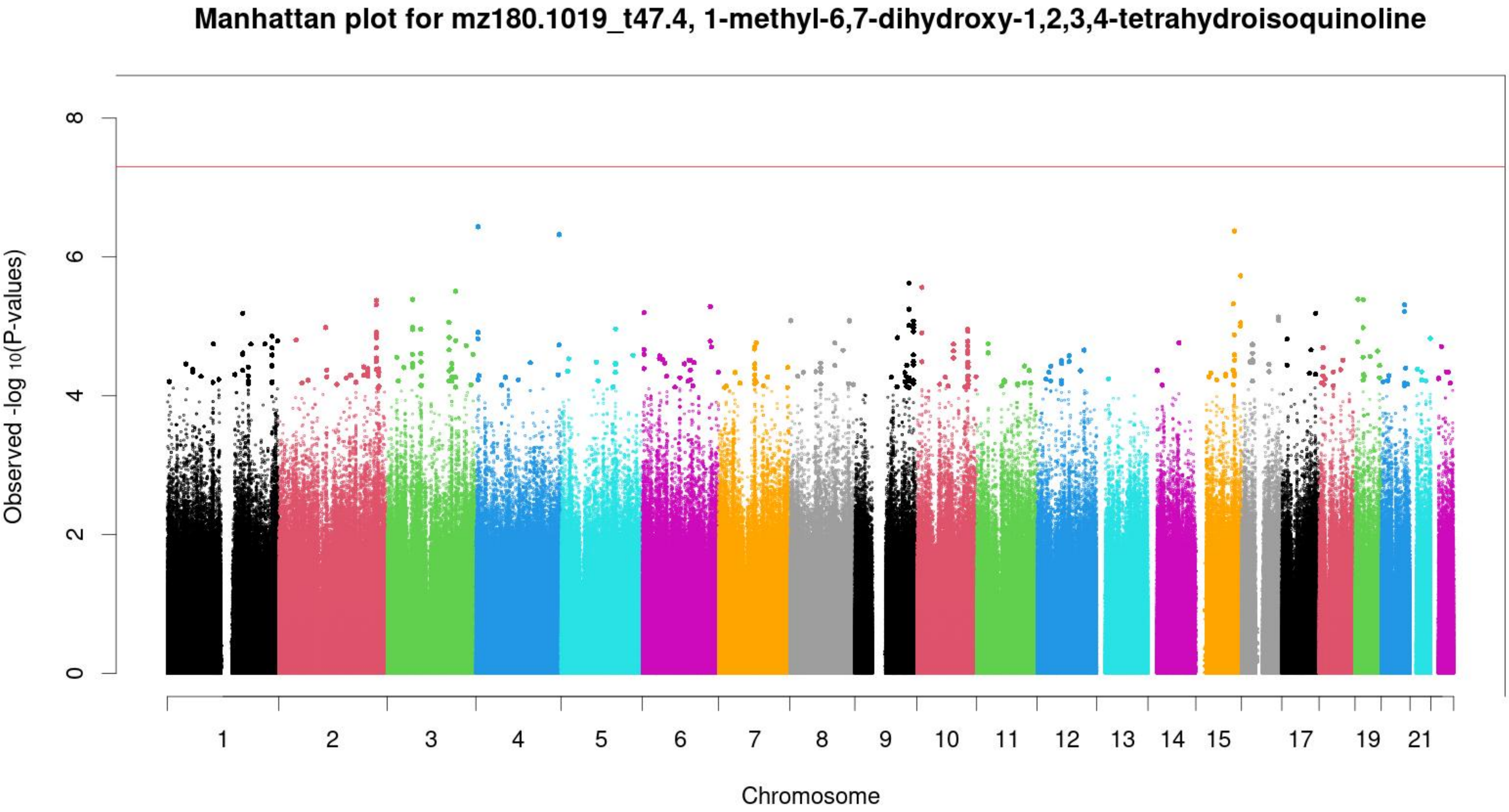
Manhattan plot for mz180.1019_t47.4, 1-methyl-6,7-dihydroxy-1,2,3,4-tetrahydroisoquinoline
Observed -log 10(P-values)
0
2
4
6
8
1
2
3
4
5
6
7
8
9
10
11
12
13
14
15
17
19
21
Chromosome

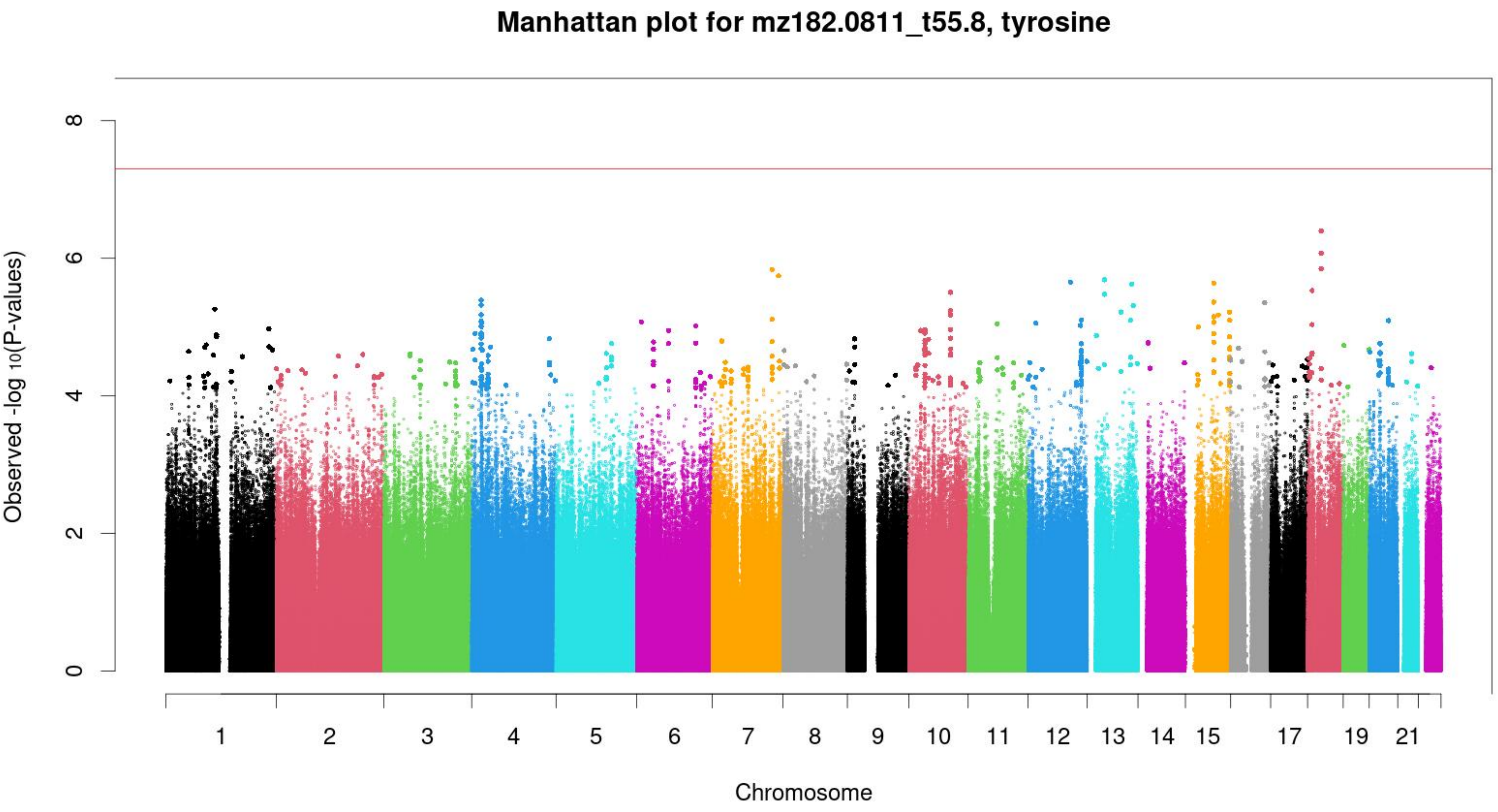

Manhattan plot for mz182.0811_t55.8, tyrosine
Observed -log 10(P-values)
0
2
4
6
8
1
2
3
4
5
6
7
8
9
10
11
12
13
14
15
17
19
21
Chromosome

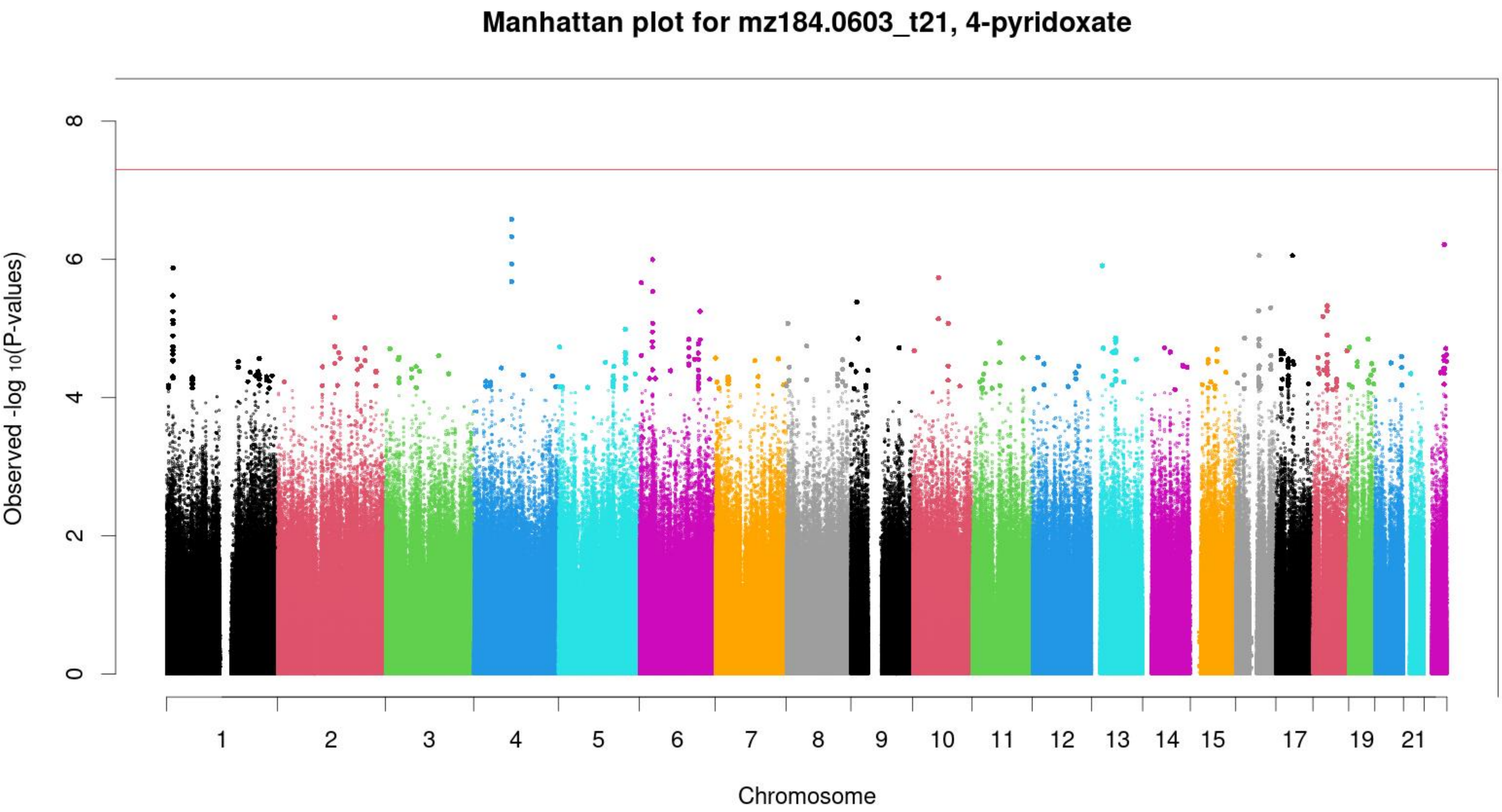

Manhattan plot for mz184.0603_t21, 4-pyridoxate
Observed -log 10(P-values)
0
2
4
6
8
1
2
3
4
5
6
7
8
9
10
11
12
13
14
15
17
19
21
Chromosome

Manhattan plot for mz189.088_t31.9, ll-2,6-diaminoheptanedioate

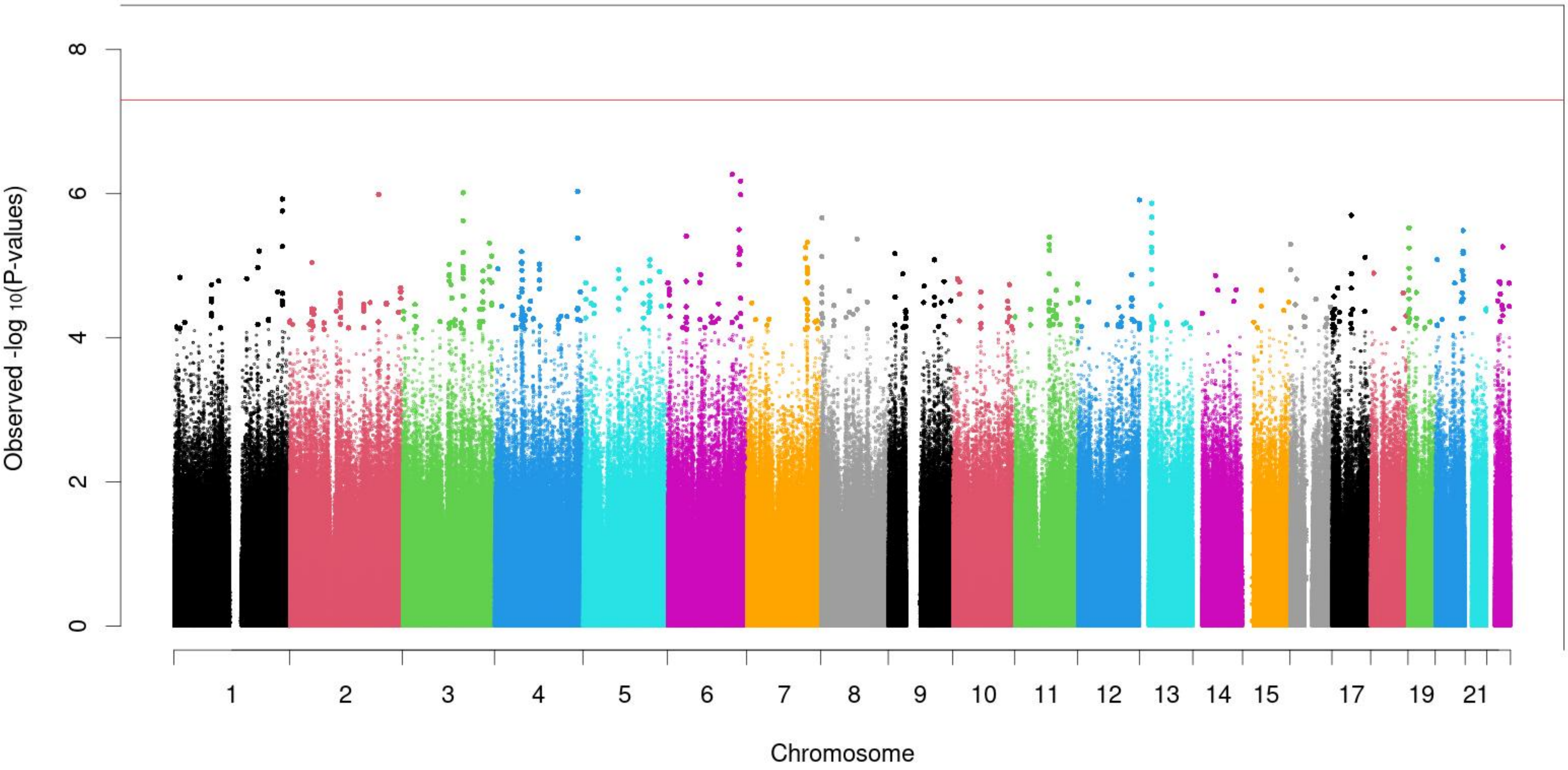

Manhattan plot for mz189.1233_t69.3, nalpha-acetyl-l-lysine

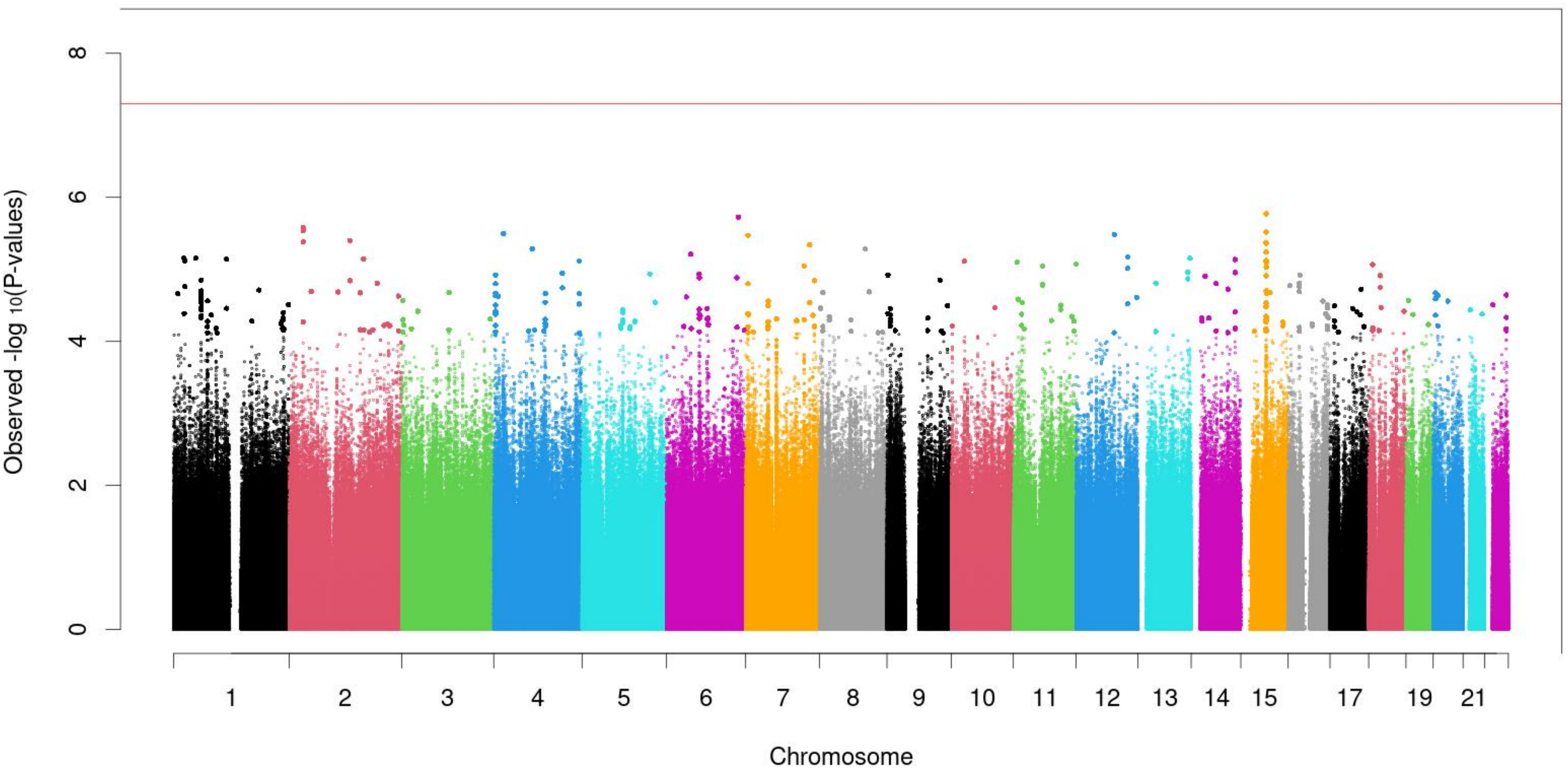

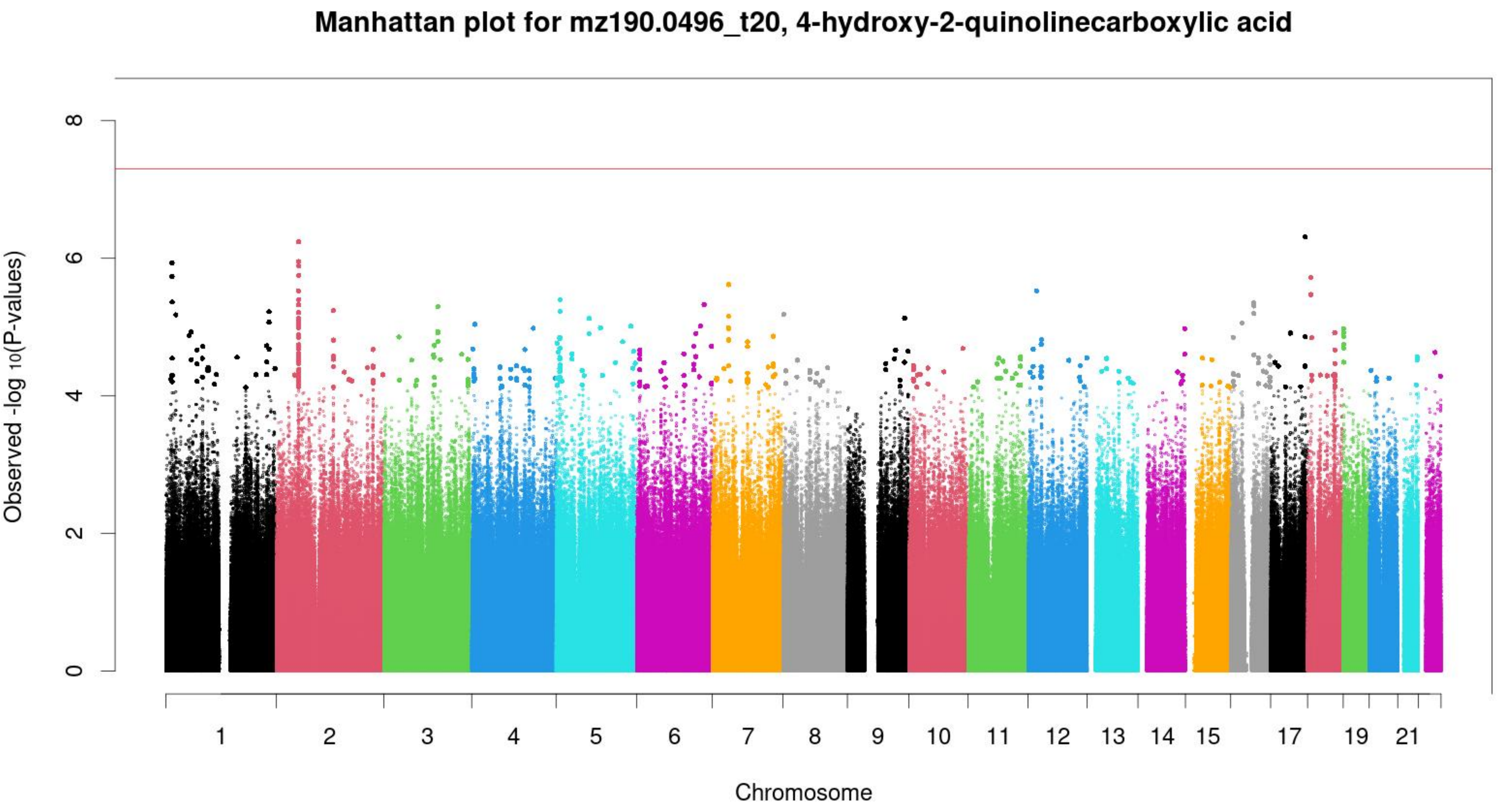

Manhattan plot for mz190.0496_t20, 4-hydroxy-2-quinolinecarboxylic acid
Observed -log 10(P-values)
0
2
4
6
8
1
2
3
4
5
6
7
8
9
10
11
12
13
14
15
17
19
21
Chromosome

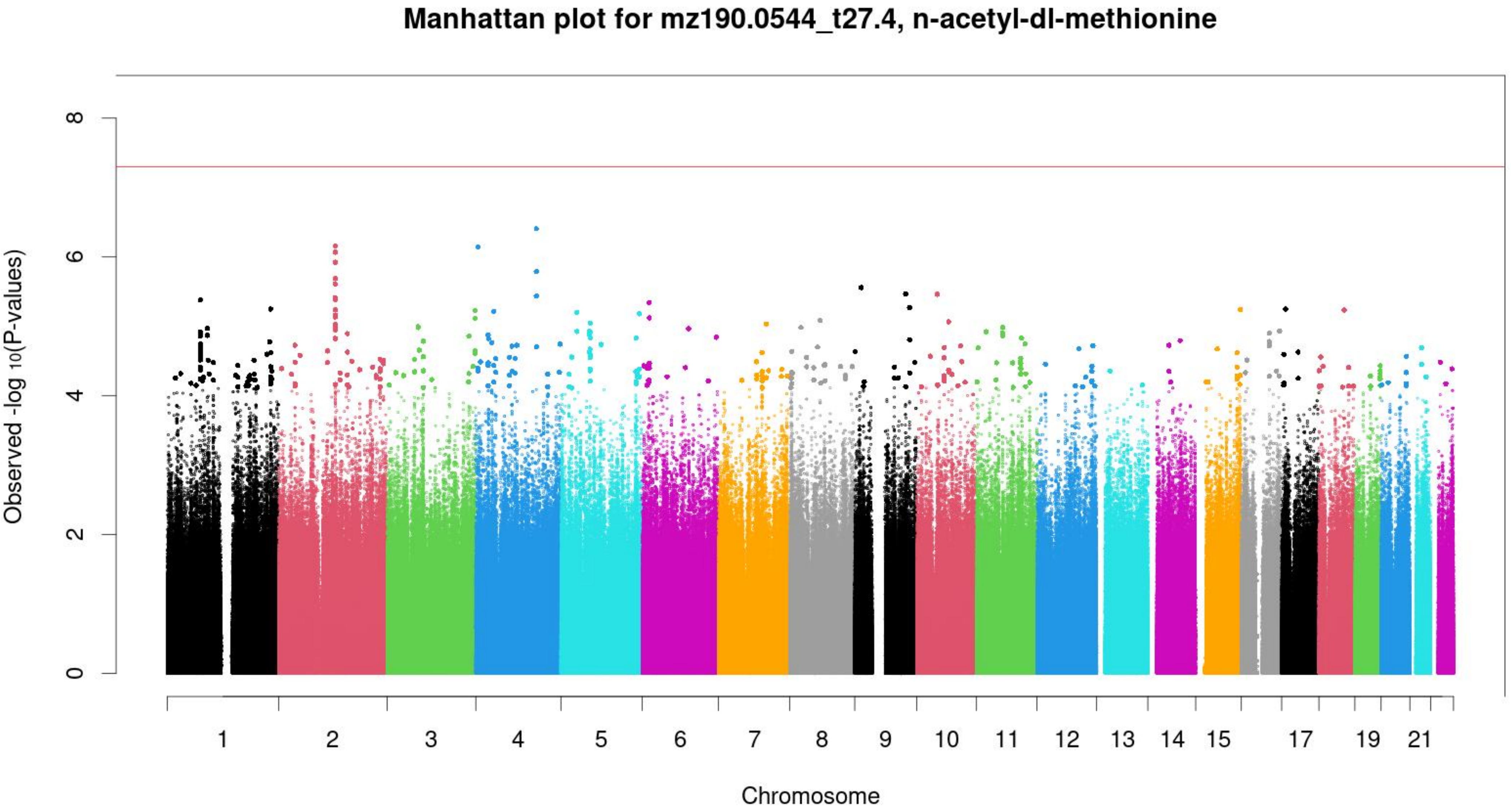
Manhattan plot for mz190.0544_t27.4, n-acetyl-dl-methionine
Observed -log 10(P-values)
0
2
4
6
8
1
2
3
4
5
6
7
8
9
10
11
12
13
14
15
17
19
21
Chromosome

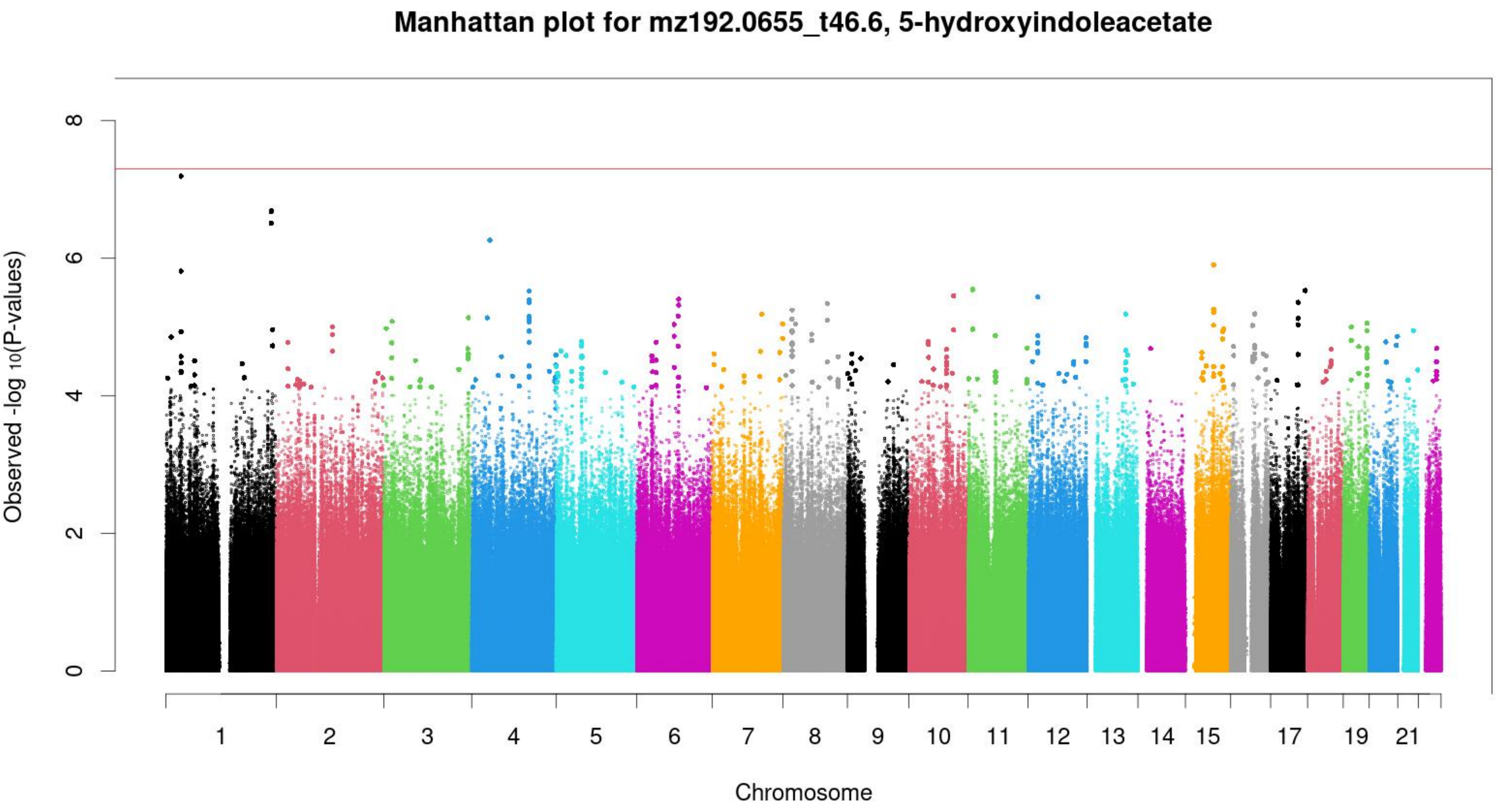

Manhattan plot for mz192.0655_t46.6, 5-hydroxyindoleacetate
Observed -log 10(P-values)
0
2
4
6
8
1
2
3
4
5
6
7
8
9
10
11
12
13
14
15
17
19
21
Chromosome

Manhattan plot for mz195.0505_t48.3, d-glucuronic acid

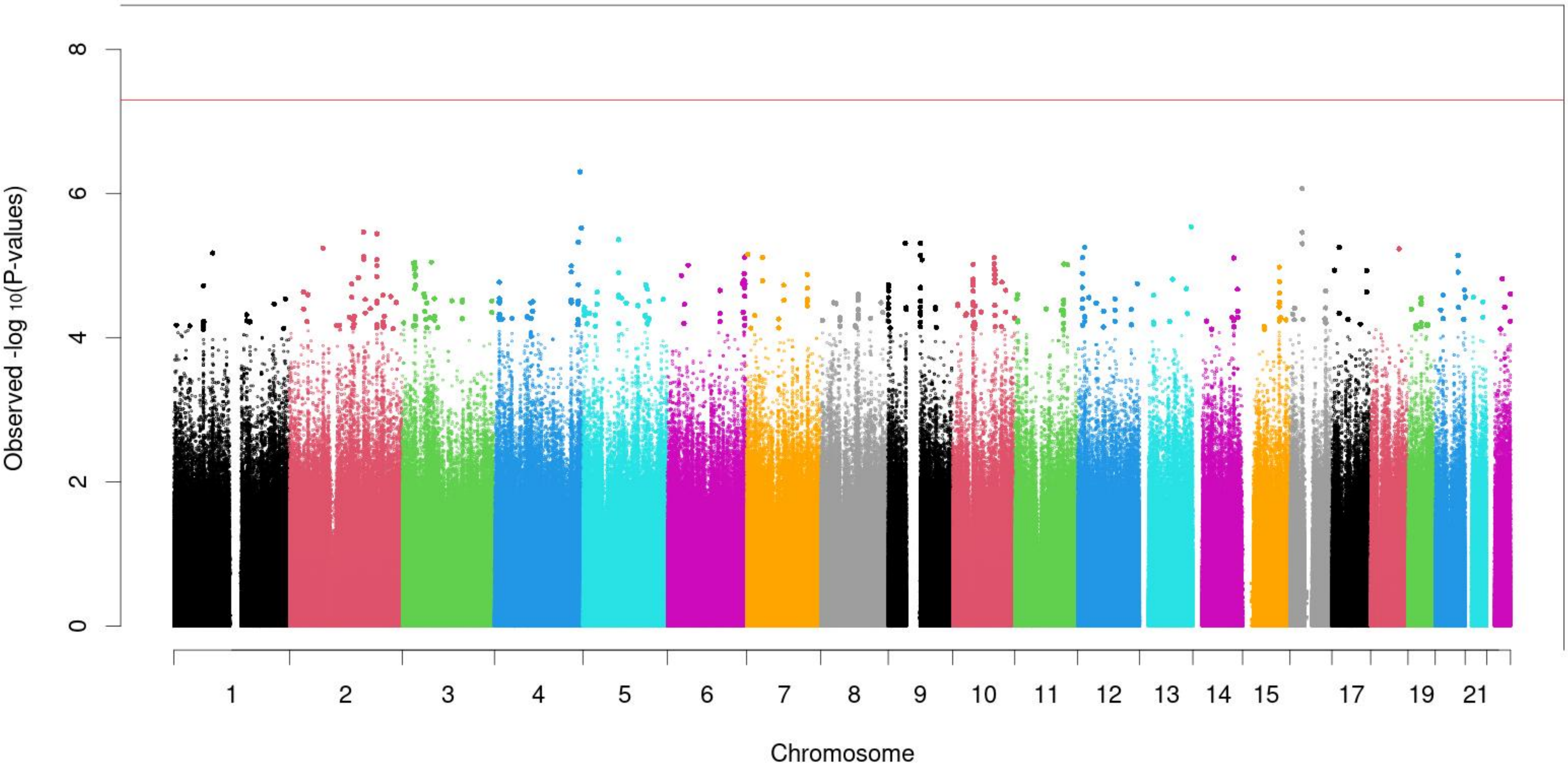

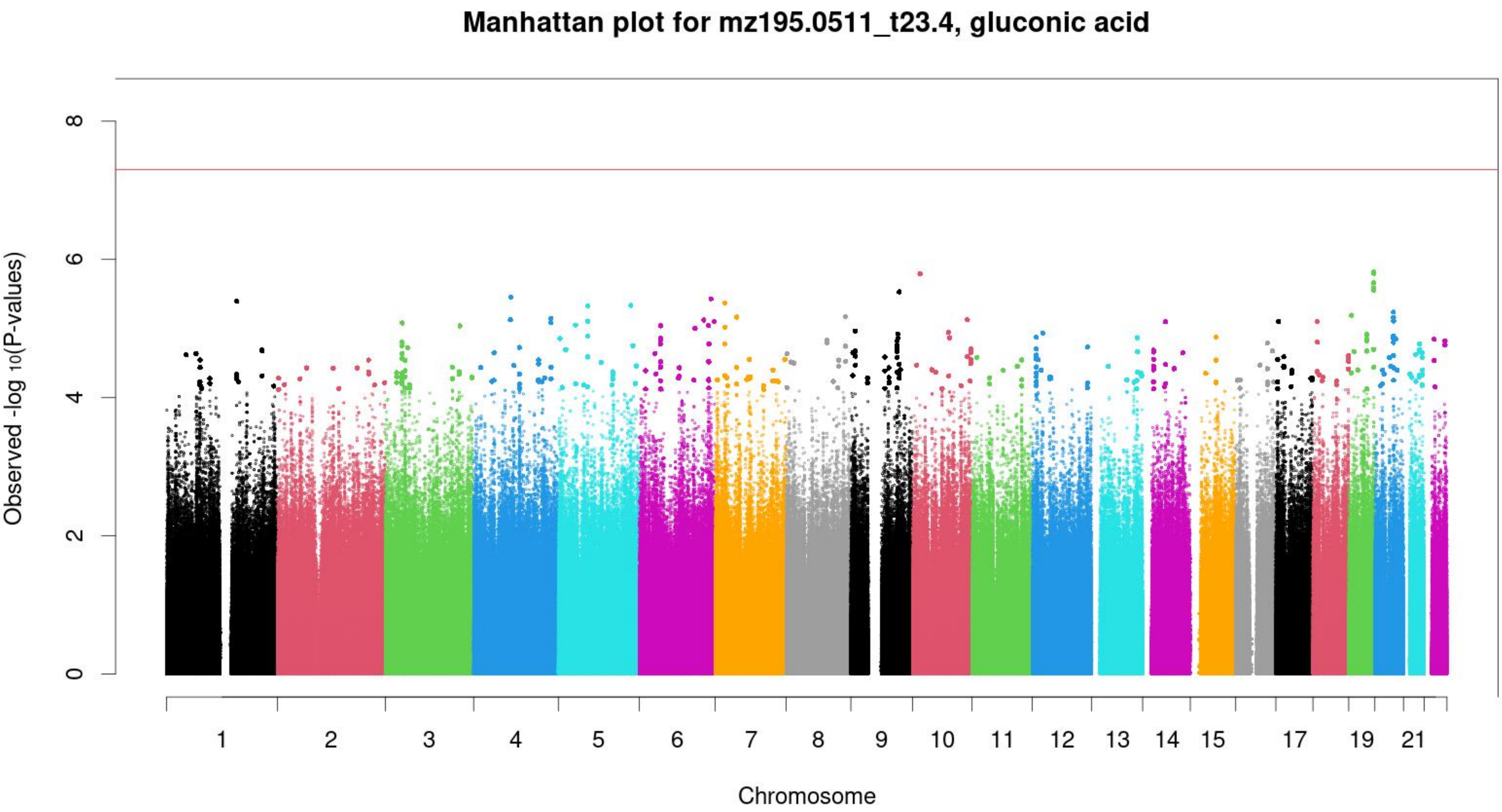

Manhattan plot for mz195.0511_t23.4, gluconic acid
Observed -log 10(P-values)
0
2
4
6
8
1
2
3
4
5
6
7
8
9
10
11
12
13
14
15
17
19
21
Chromosome

Manhattan plot for mz195.0651_t53.4, ferulate

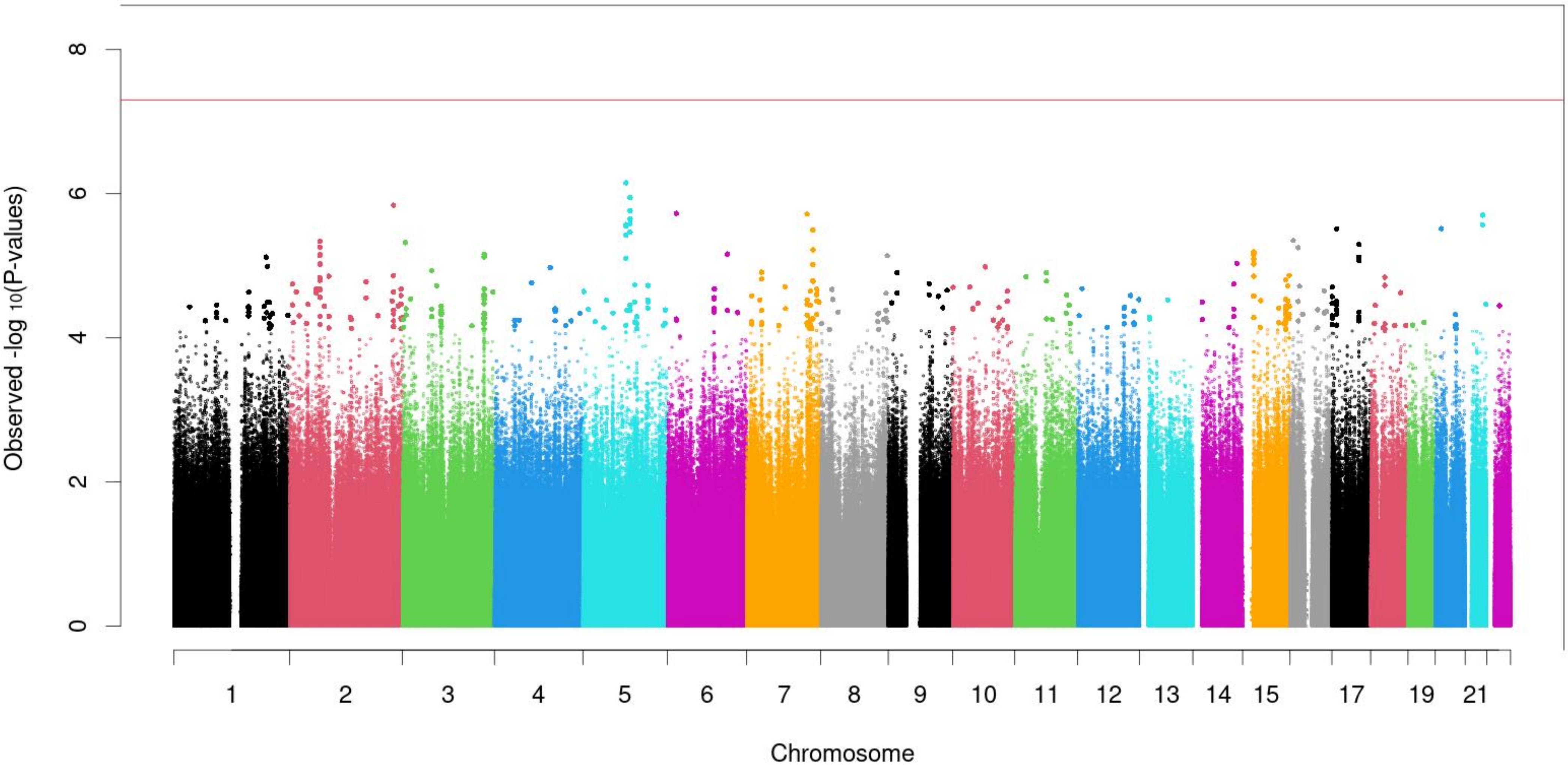

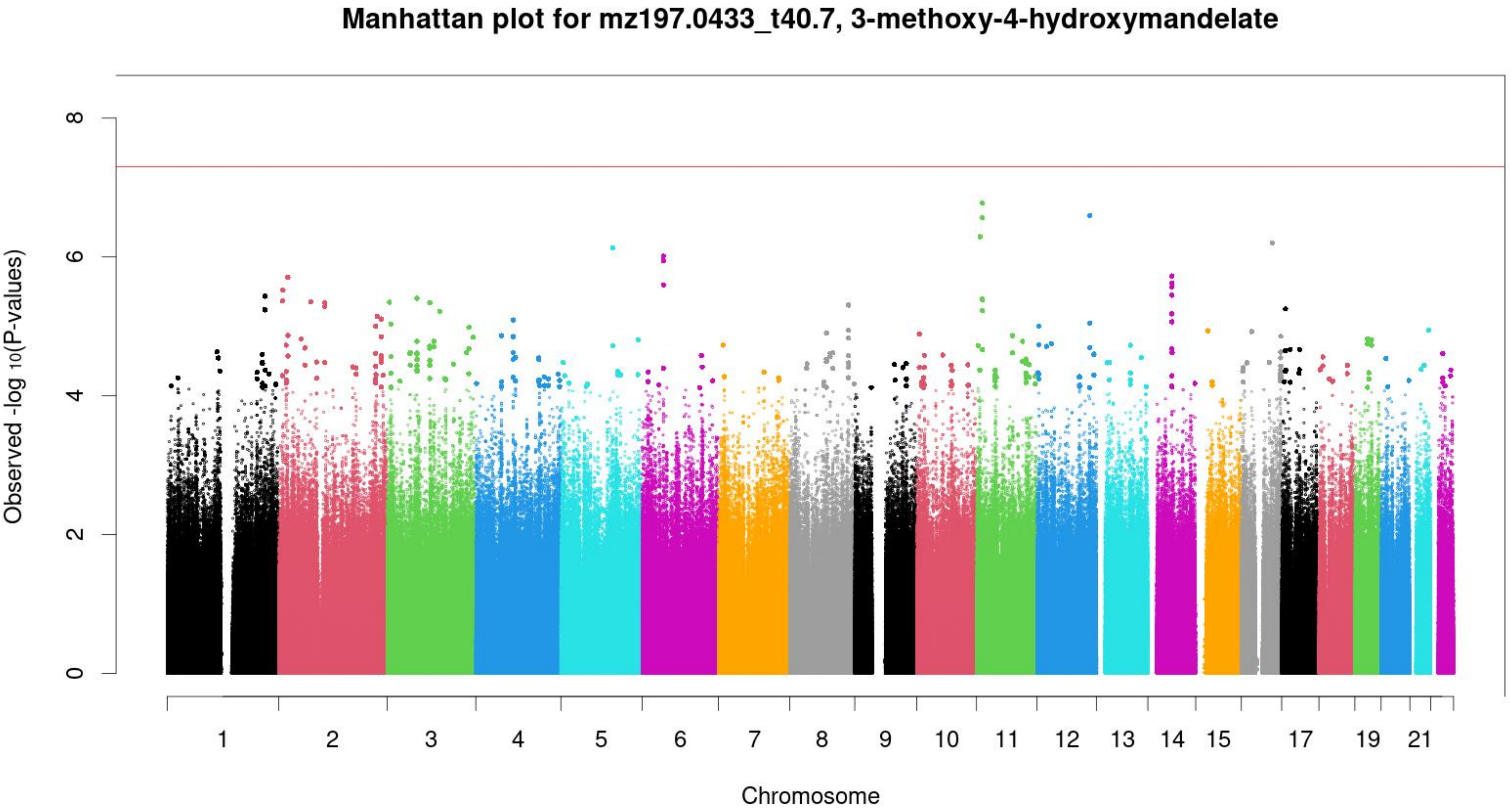

Manhattan plot for mz197.0433_t40.7, 3-methoxy-4-hydroxymandelate
Observed -log 10(P-values)
0
2
4
6
8
1
2
3
4
5
6
7
8
9
10
11
12
13
14
15
17
19
21
Chromosome

Manhattan plot for mz198.076_t50.3, 3,4-dihydroxy-l-phenylalanine (l-dopa)

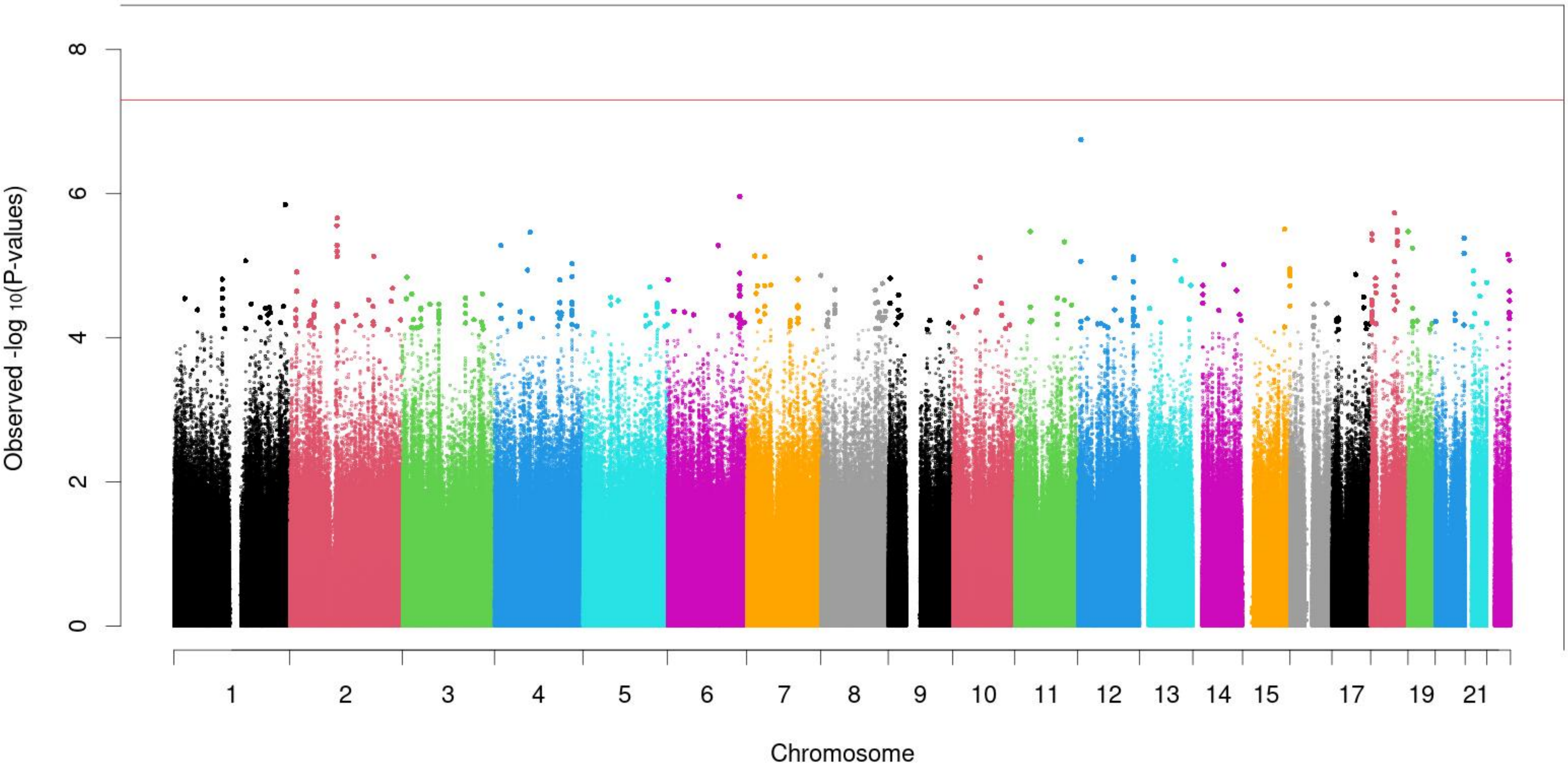

Manhattan plot for mz199.1704_t152.3, lauric acid

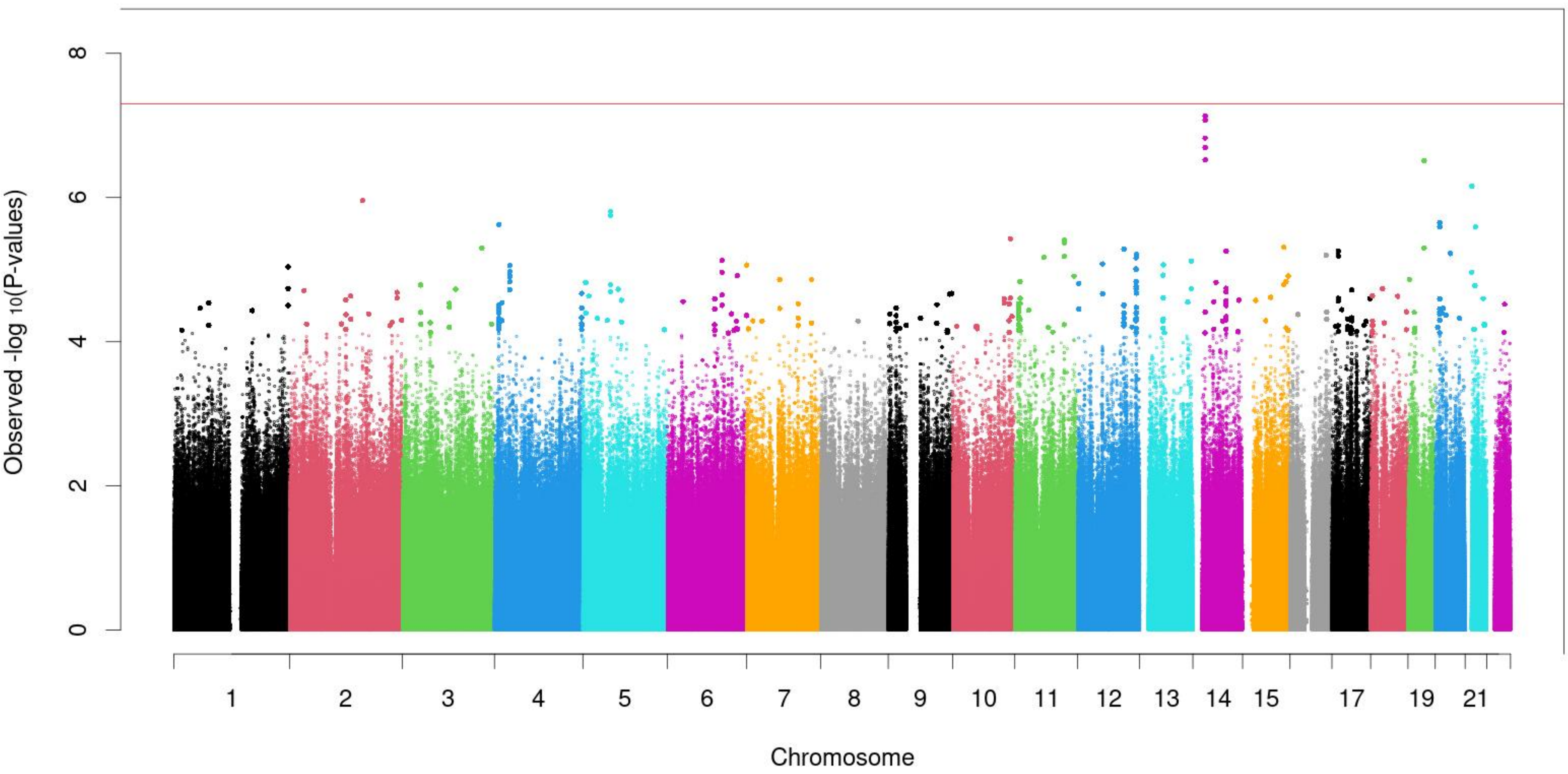

Manhattan plot for mz202.1087_t40.9, n6-(delta2-isopentenyl)-adenine

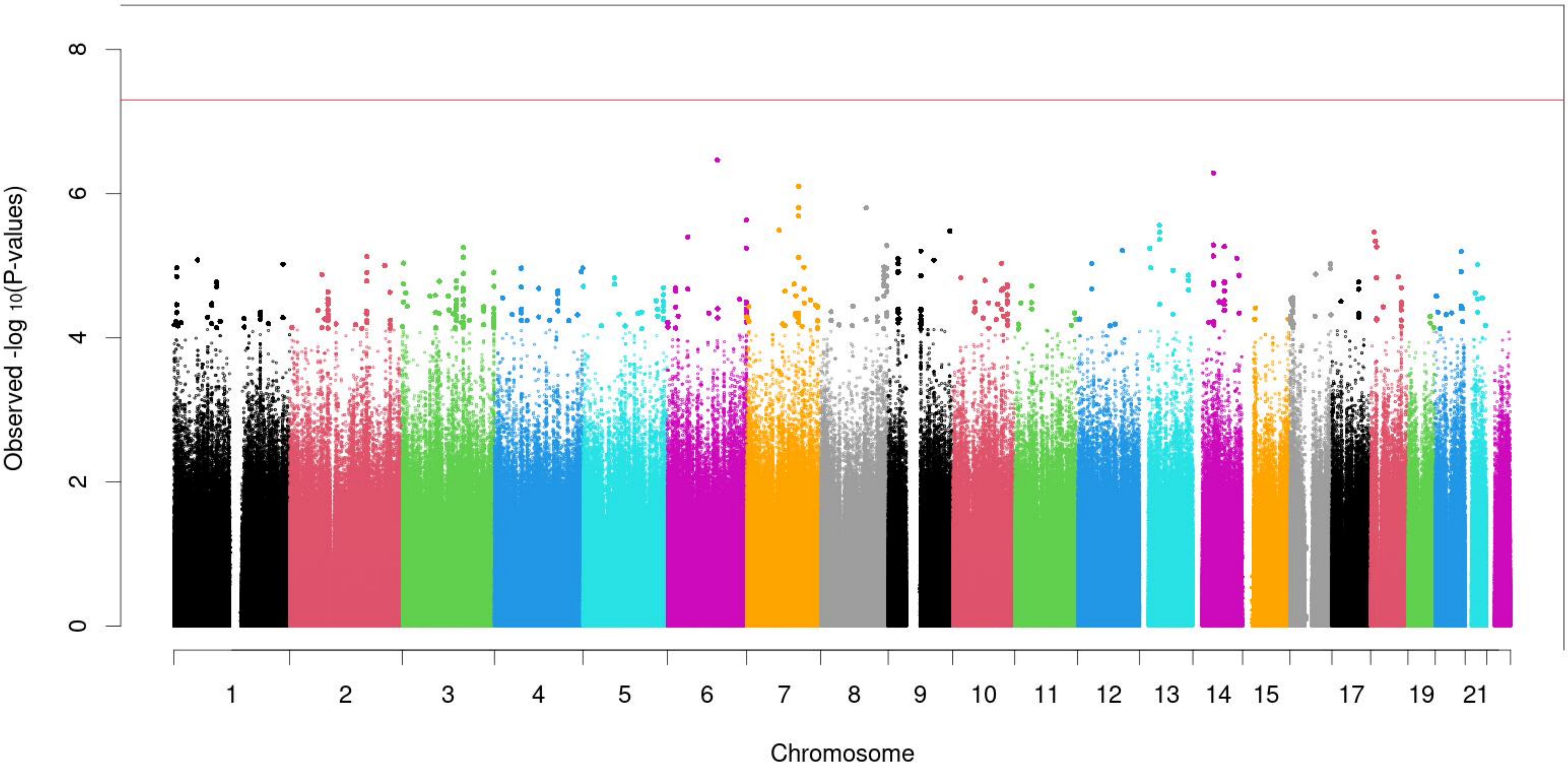

Manhattan plot for mz204.123_t63.1, o-acetyl-l-carnitine

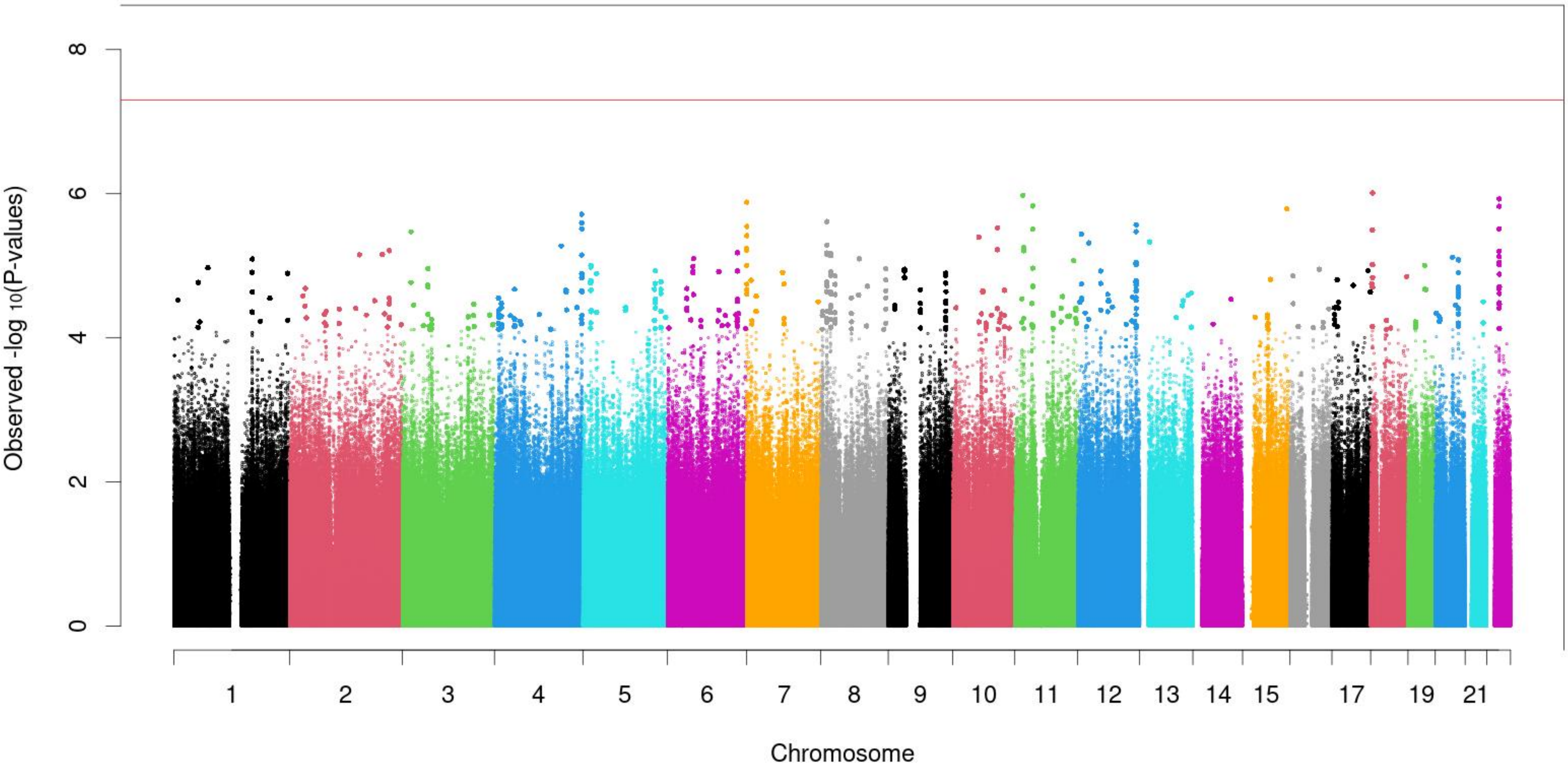

Manhattan plot for mz205.0972_t45.9, tryptophan

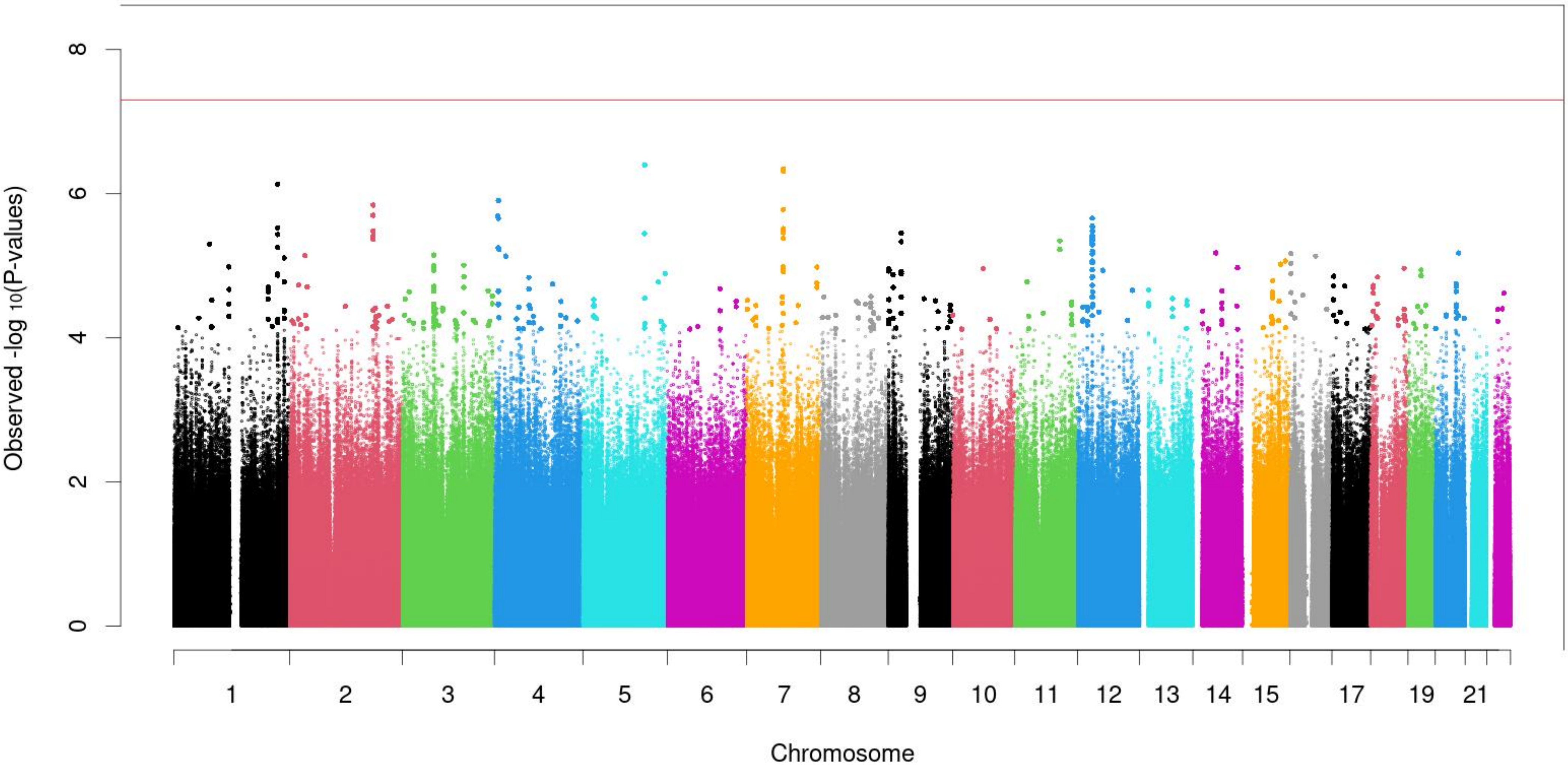

Manhattan plot for mz206.0825_t46.2, n-acetyl-l-phenylalanine

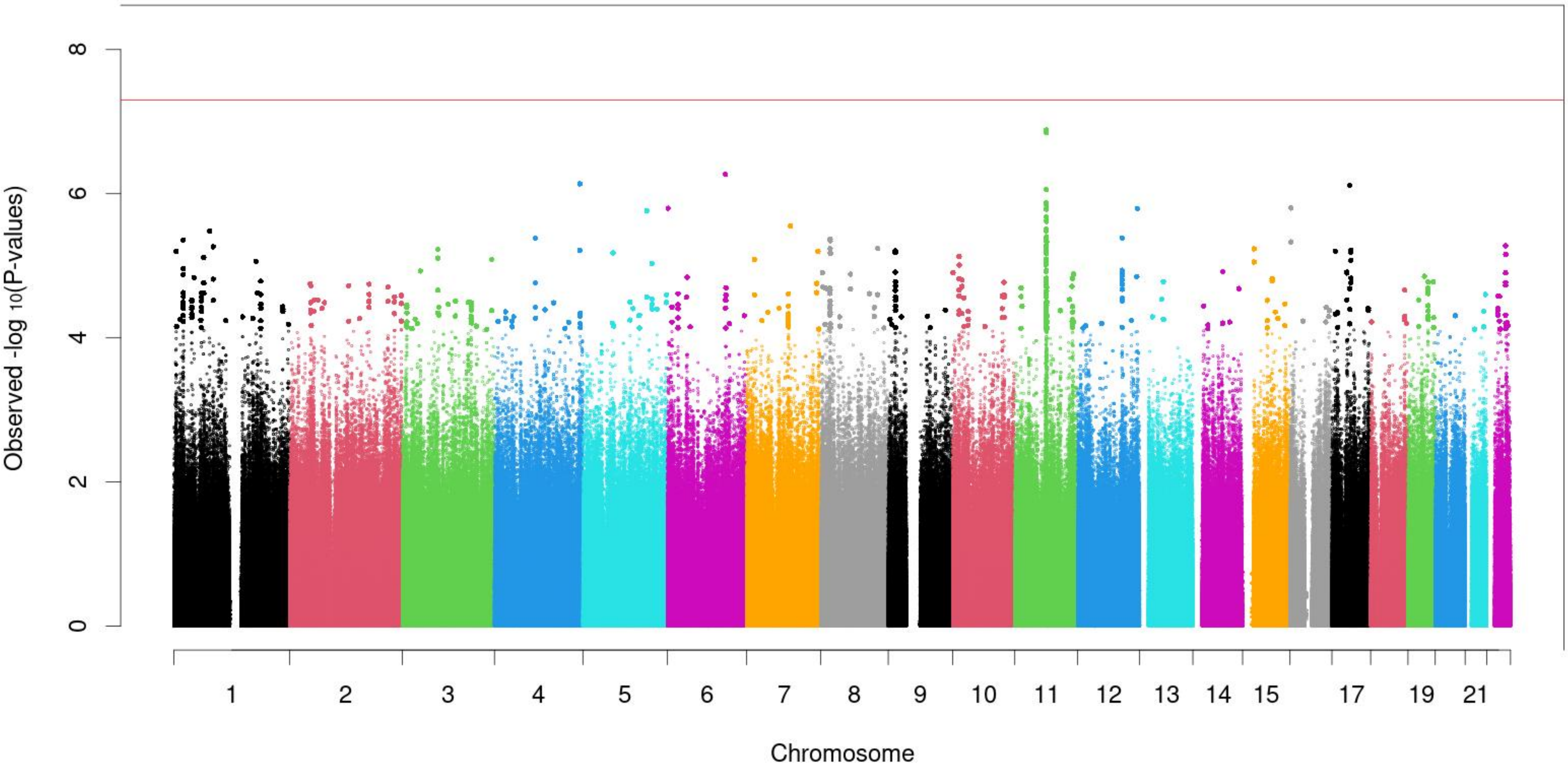

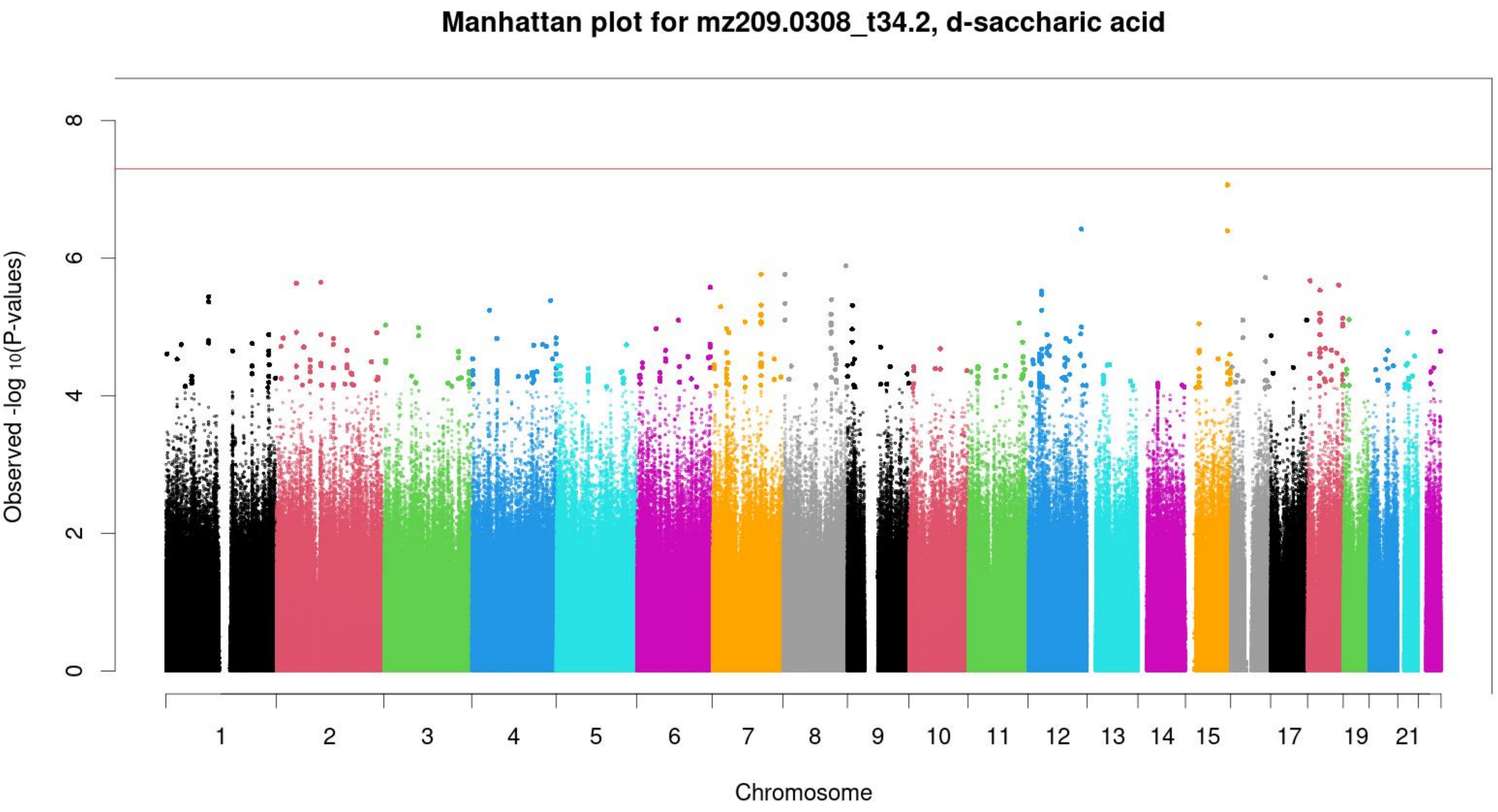

Manhattan plot for mz209.0308_t34.2, d-saccharic acid
Observed -log 10(P-values)
0
2
4
6
8
1
2
3
4
5
6
7
8
9
10
11
12
13
14
15
17
19
21
Chromosome

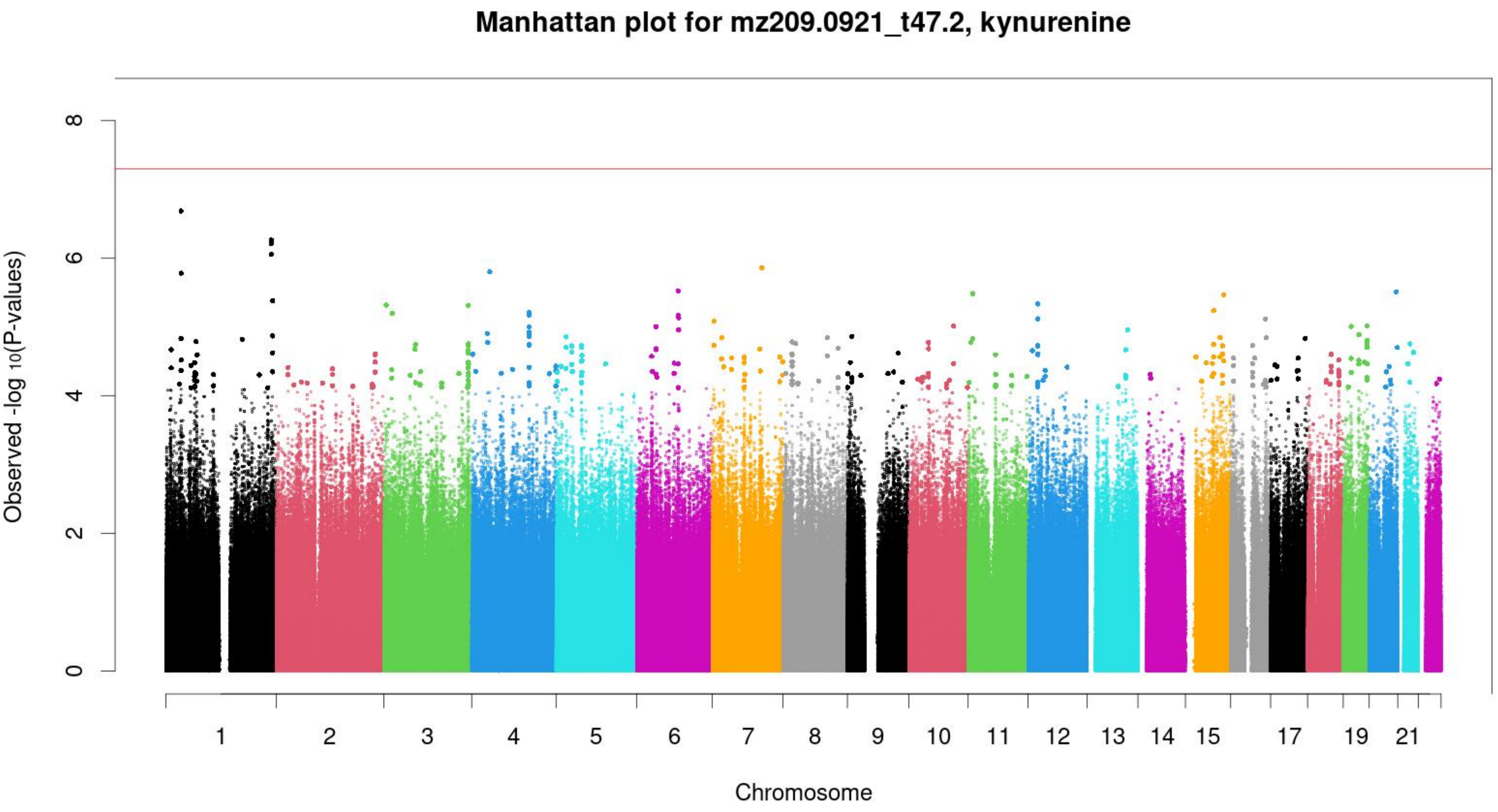

Manhattan plot for mz209.0921_t47.2, kynurenine
Observed -log 10(P-values)
0
2
4
6
8
1
2
3
4
5
6
7
8
9
10
11
12
13
14
15
17
19
21
Chromosome

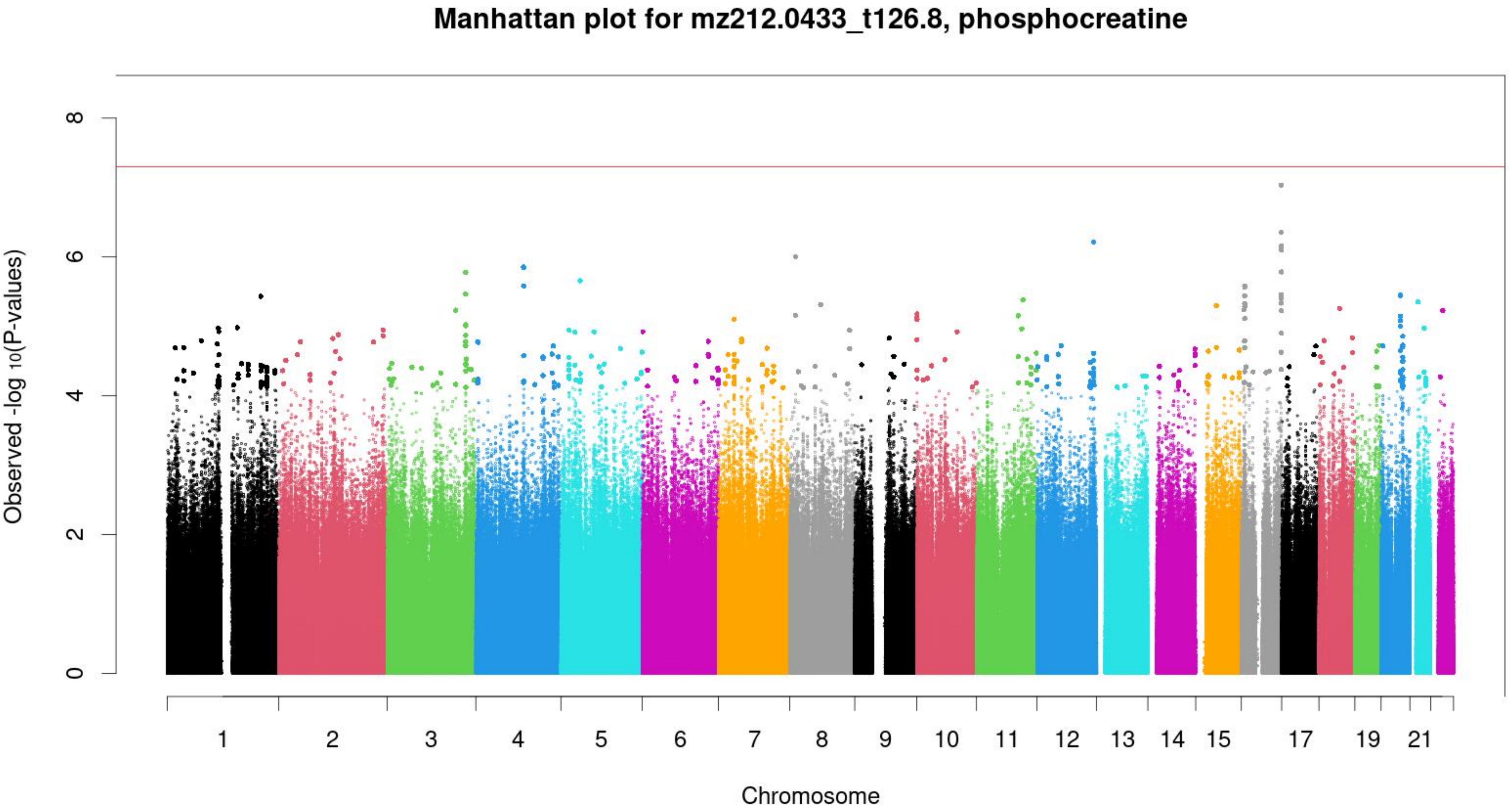

Manhattan plot for mz212.0433_t126.8, phosphocreatine
Observed -log 10(P-values)
0
2
4
6
8
Chromosome
1
2
3
4
5
6
7
8
9
10
11
12
13
14
15
17
19
21

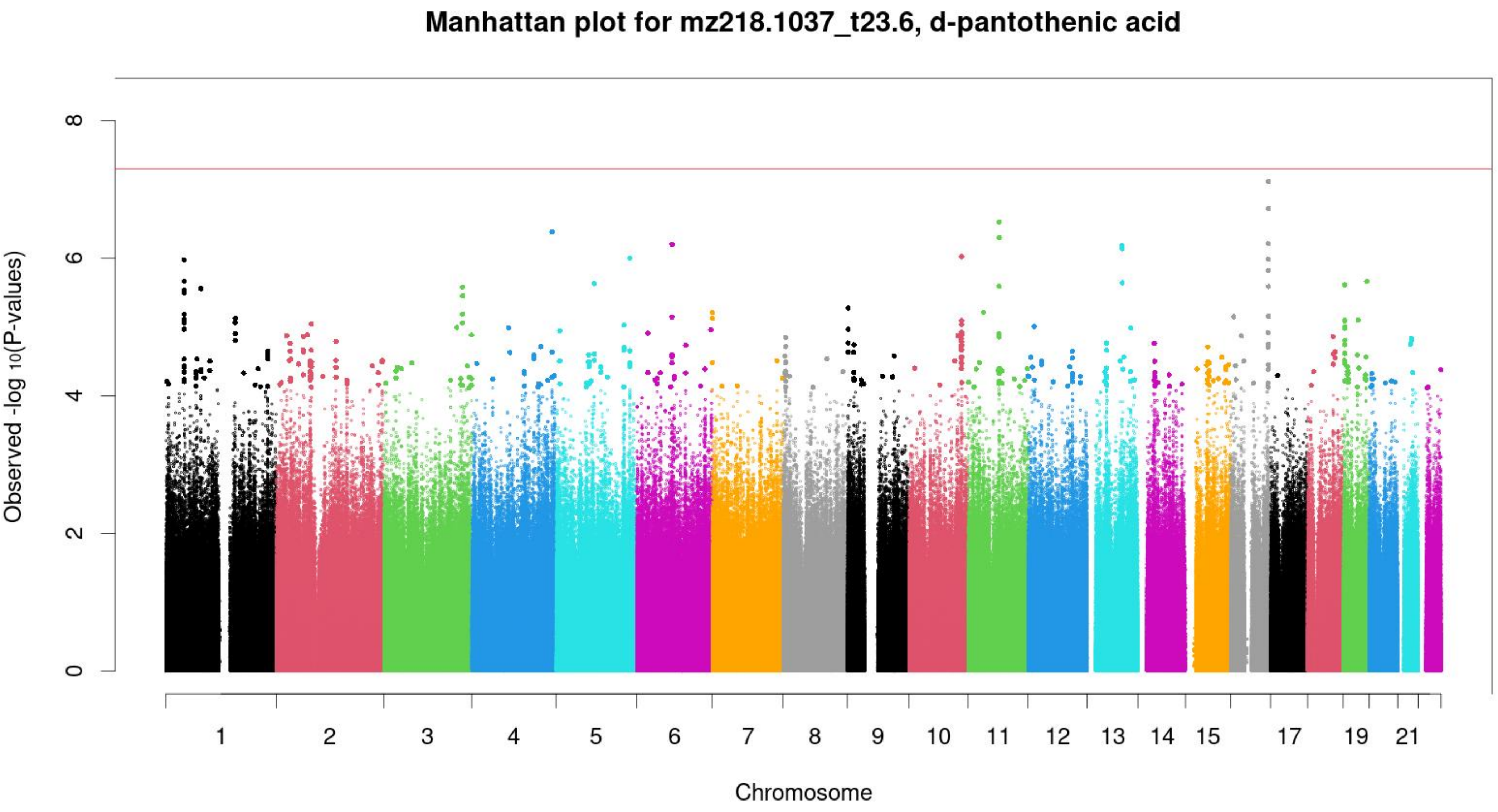

Manhattan plot for mz218.1037_t23.6, d-pantothenic acid
Observed -log 10(P-values)
0
2
4
6
8
1
2
3
4
5
6
7
8
9
10
11
12
13
14
15
17
19
21
Chromosome

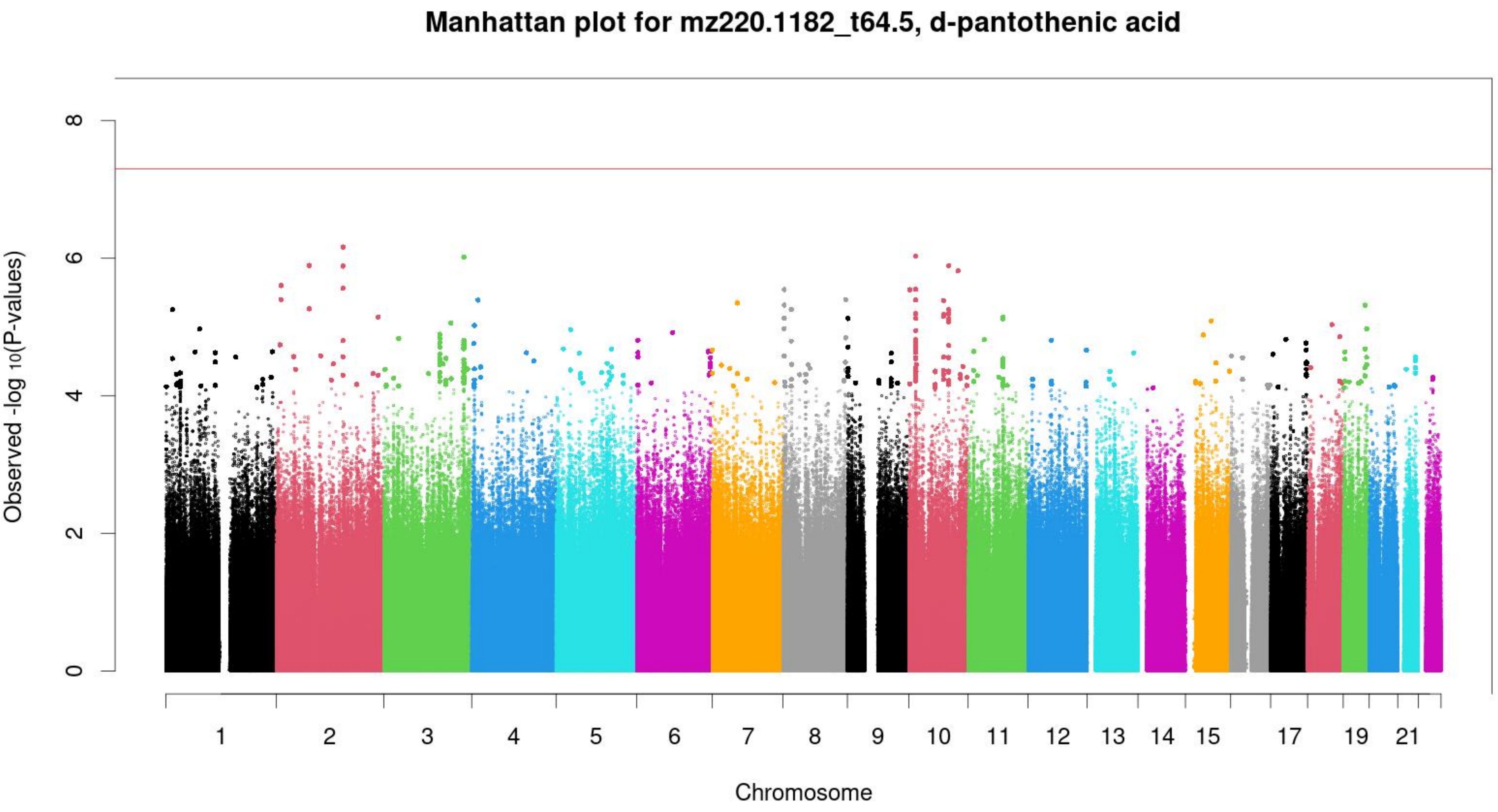

Manhattan plot for mz220.1182_t64.5, d-pantothenic acid
Observed -log 10(P-values)
0
2
4
6
8
1
2
3
4
5
6
7
8
9
10
11
12
13
14
15
17
19
21
Chromosome

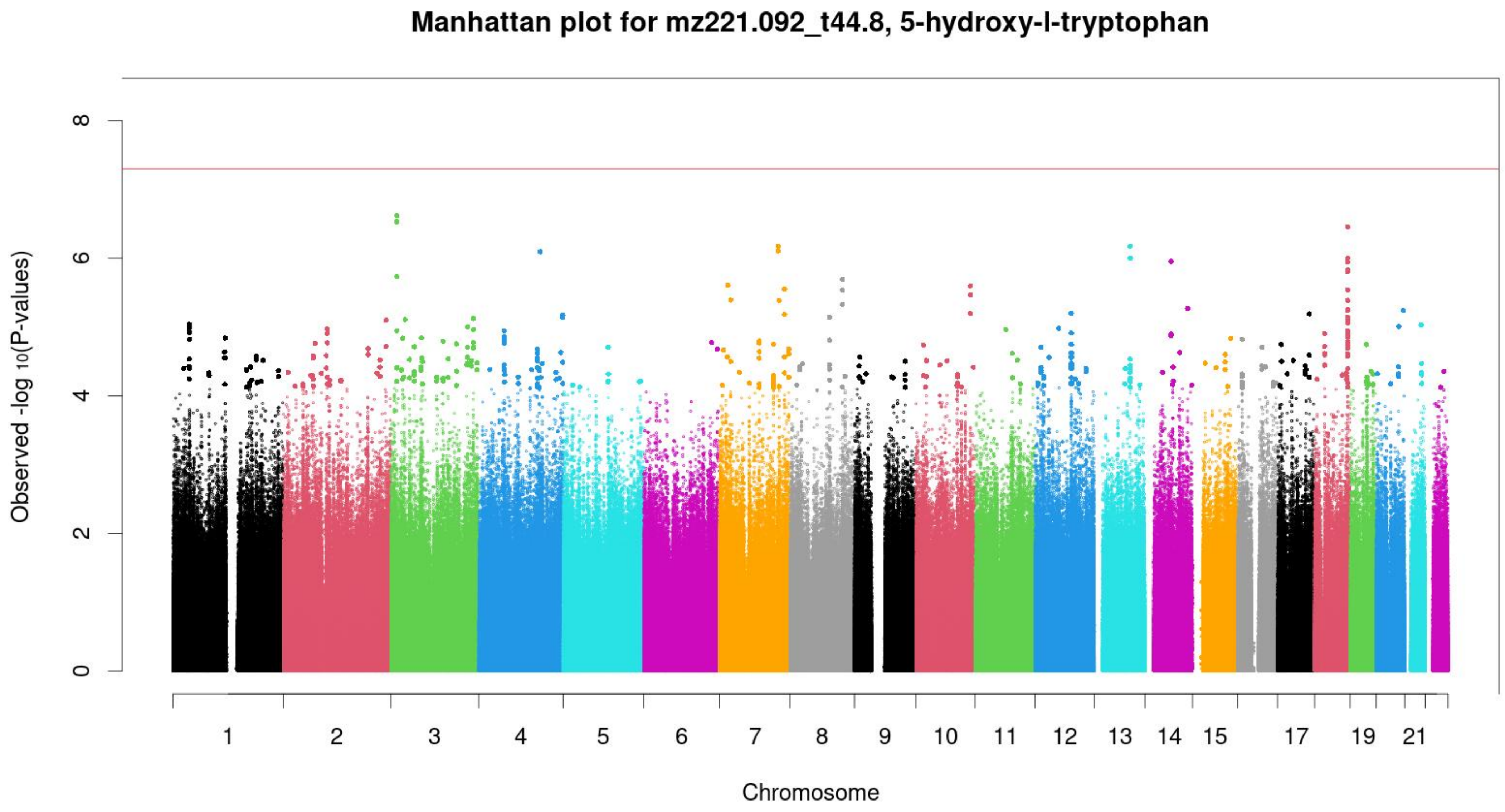
Manhattan plot for mz221.092_t44.8, 5-hydroxy-l-tryptophan
Observed -log 10(P-values)
0
2
4
6
8
Chromosome
1
2
3
4
5
6
7
8
9
10
11
12
13
14
15
17
19
21

Manhattan plot for mz222.0962_t43.6, n-acetyl-d-glucosamine

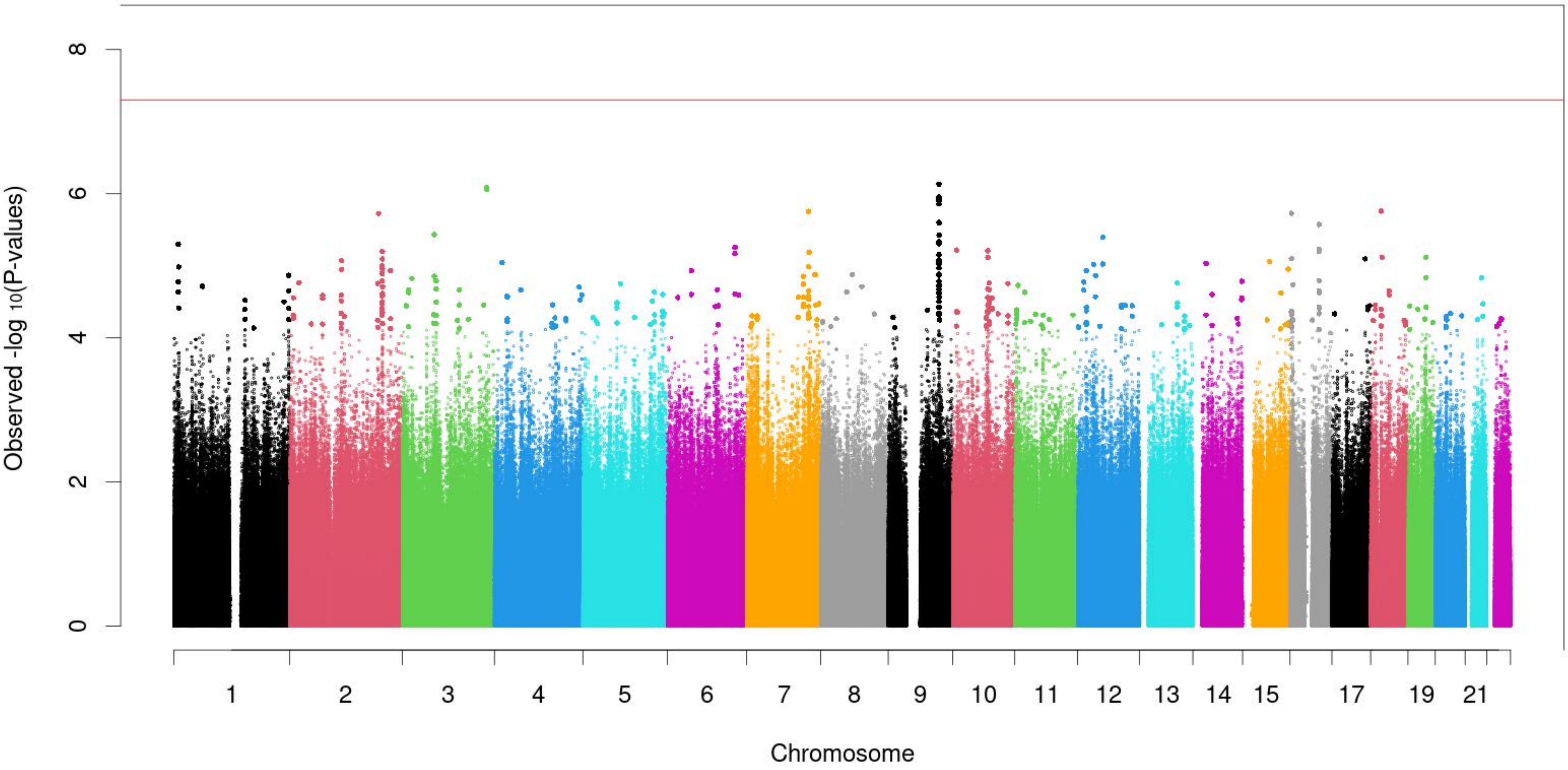

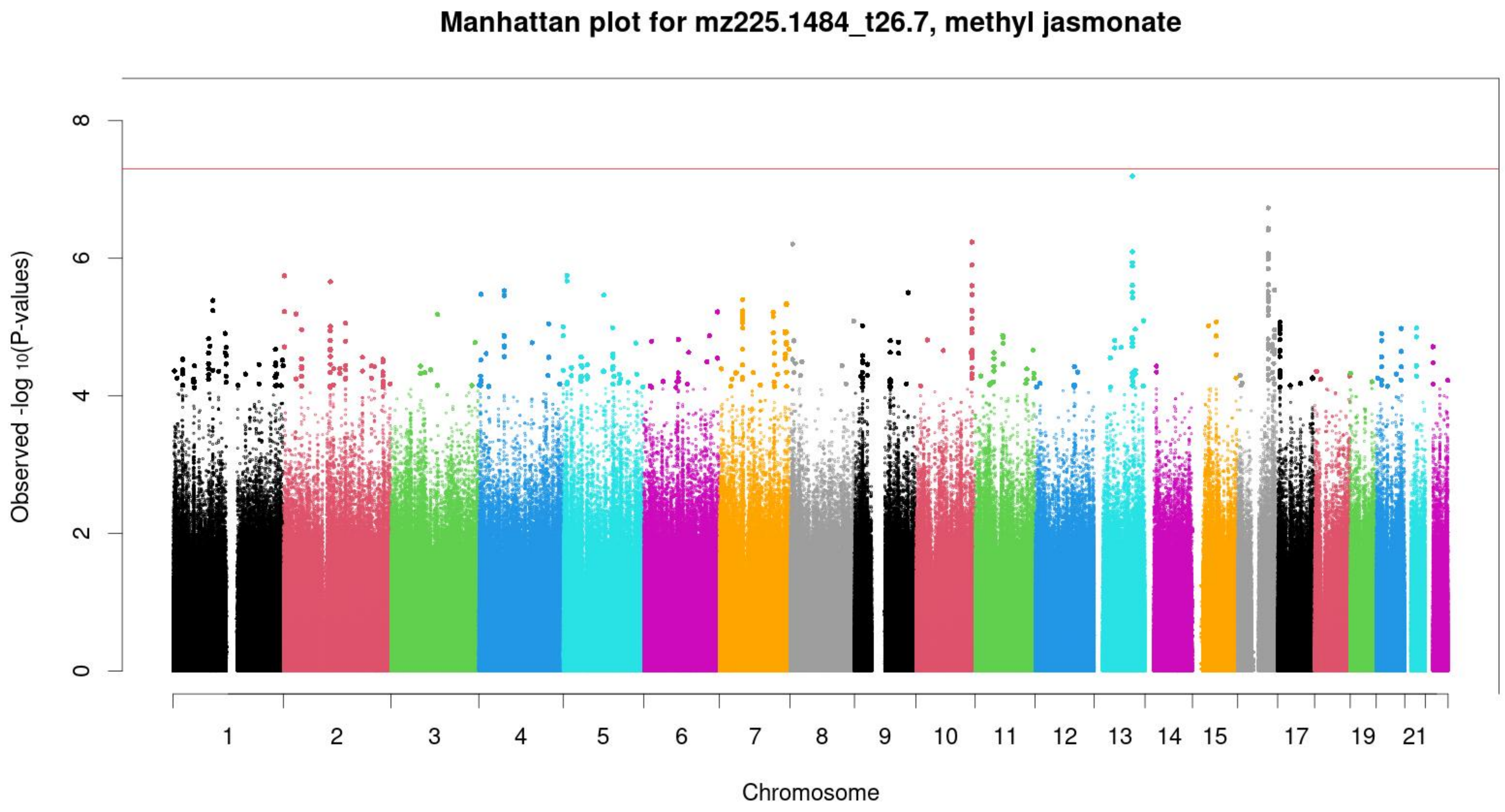
Manhattan plot for mz225.1484_t26.7, methyl jasmonate
Observed -log 10(P-values)
0
2
4
6
8
1
2
3
4
5
6
7
8
9
10
11
12
13
14
15
17
19
21
Chromosome

Manhattan plot for mz237.0362_t73.2, quinate

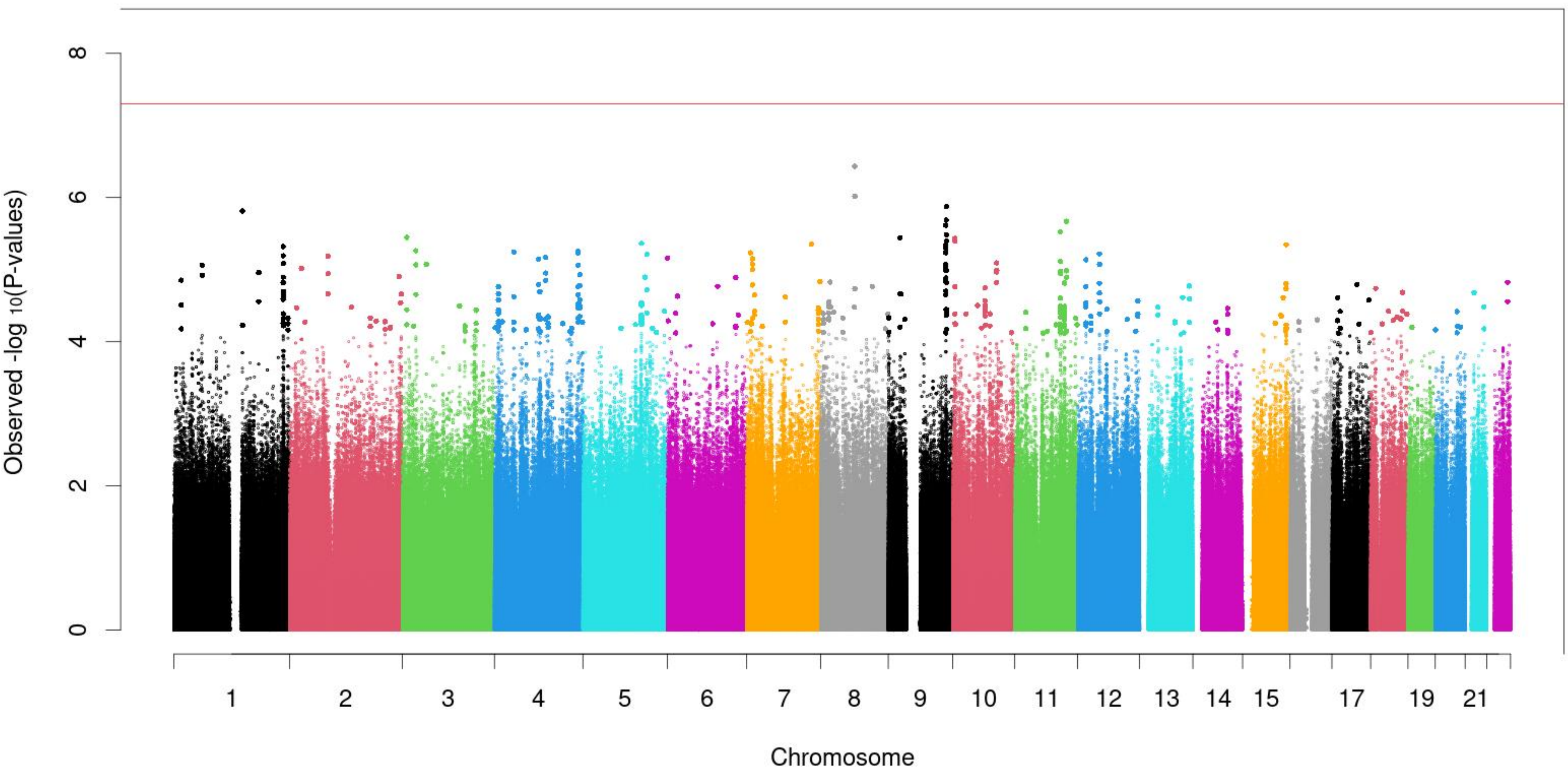

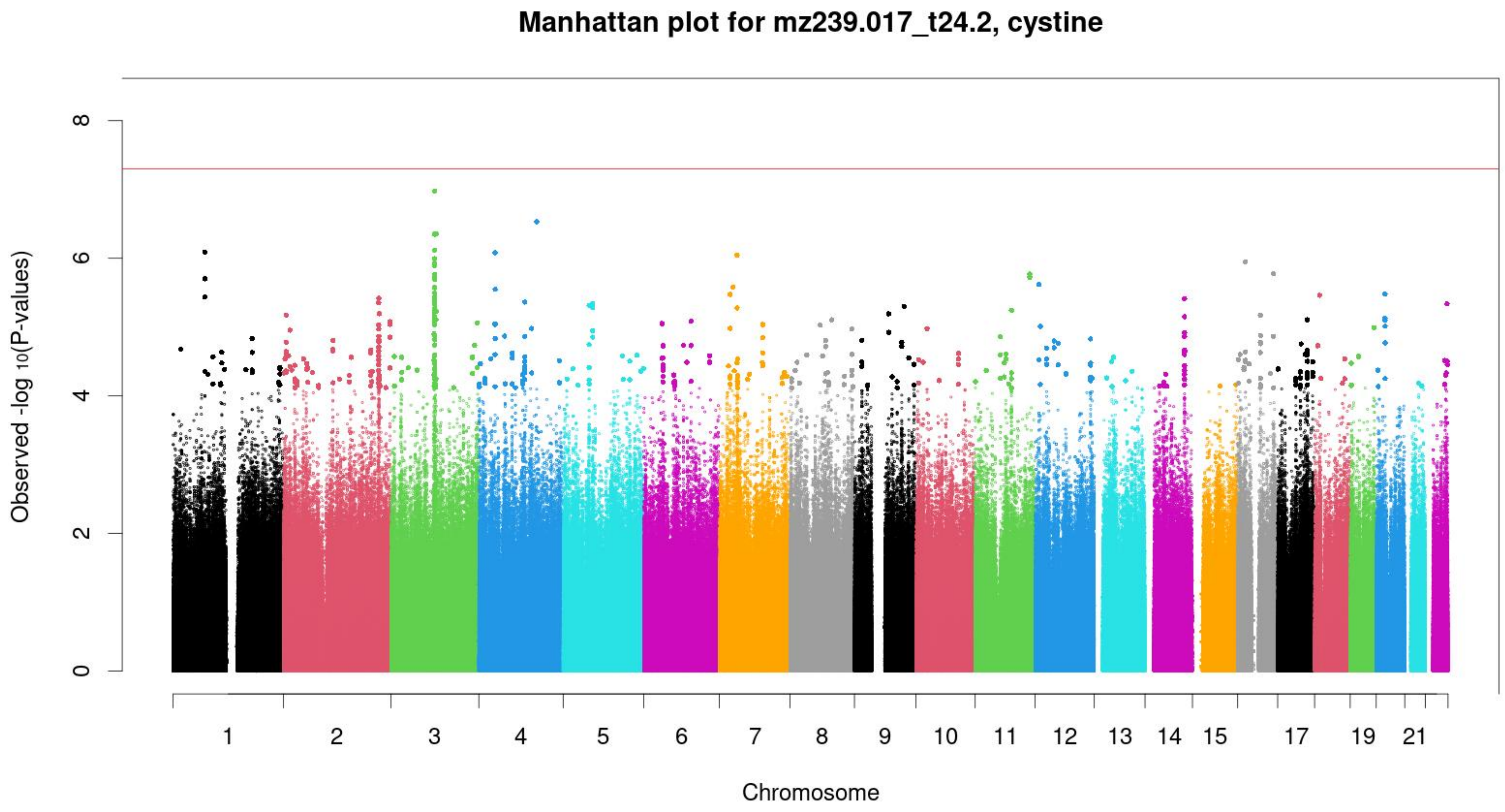
Manhattan plot for mz239.017_t24.2, cystine
Observed -log 10(P-values)
0
2
4
6
8
1
2
3
4
5
6
7
8
9
10
11
12
13
14
15
17
19
21
Chromosome

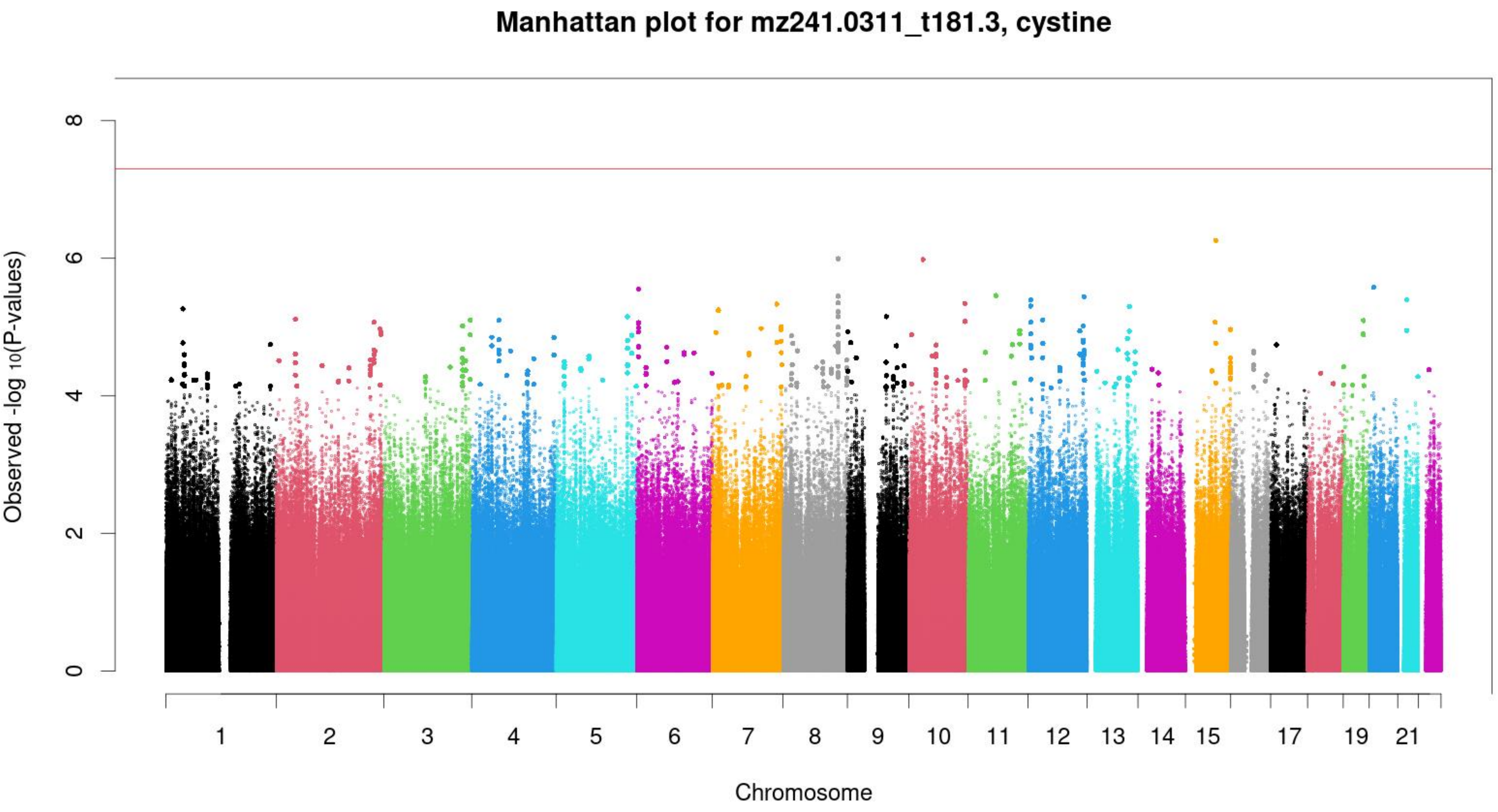
Manhattan plot for mz241.0311_t181.3, cystine
Observed -log 10(P-values)
0
2
4
6
8
Chromosome
1
2
3
4
5
6
7
8
9
10
11
12
13
14
15
17
19
21

Manhattan plot for mz243.0627_t22.4, uridine

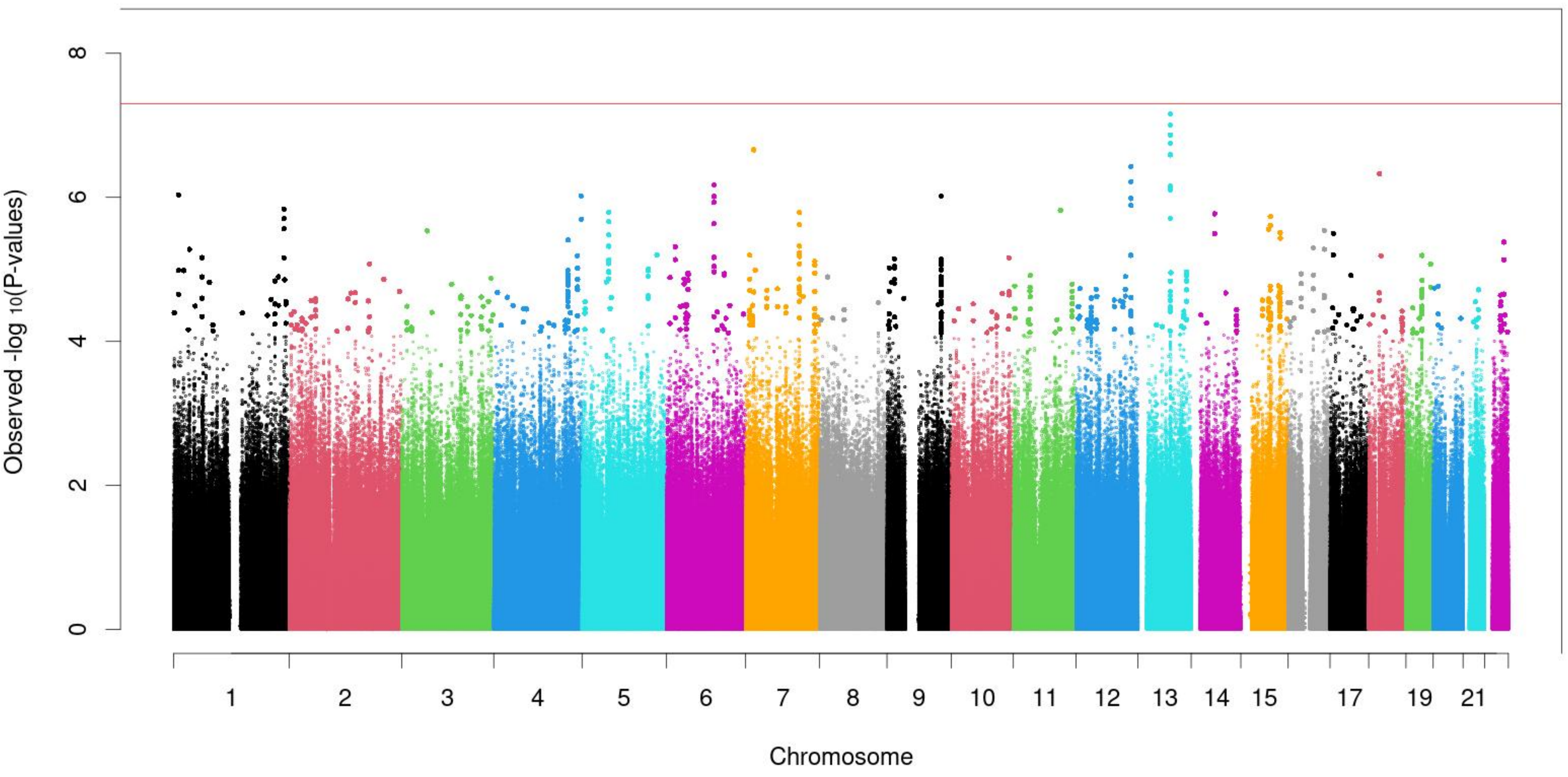

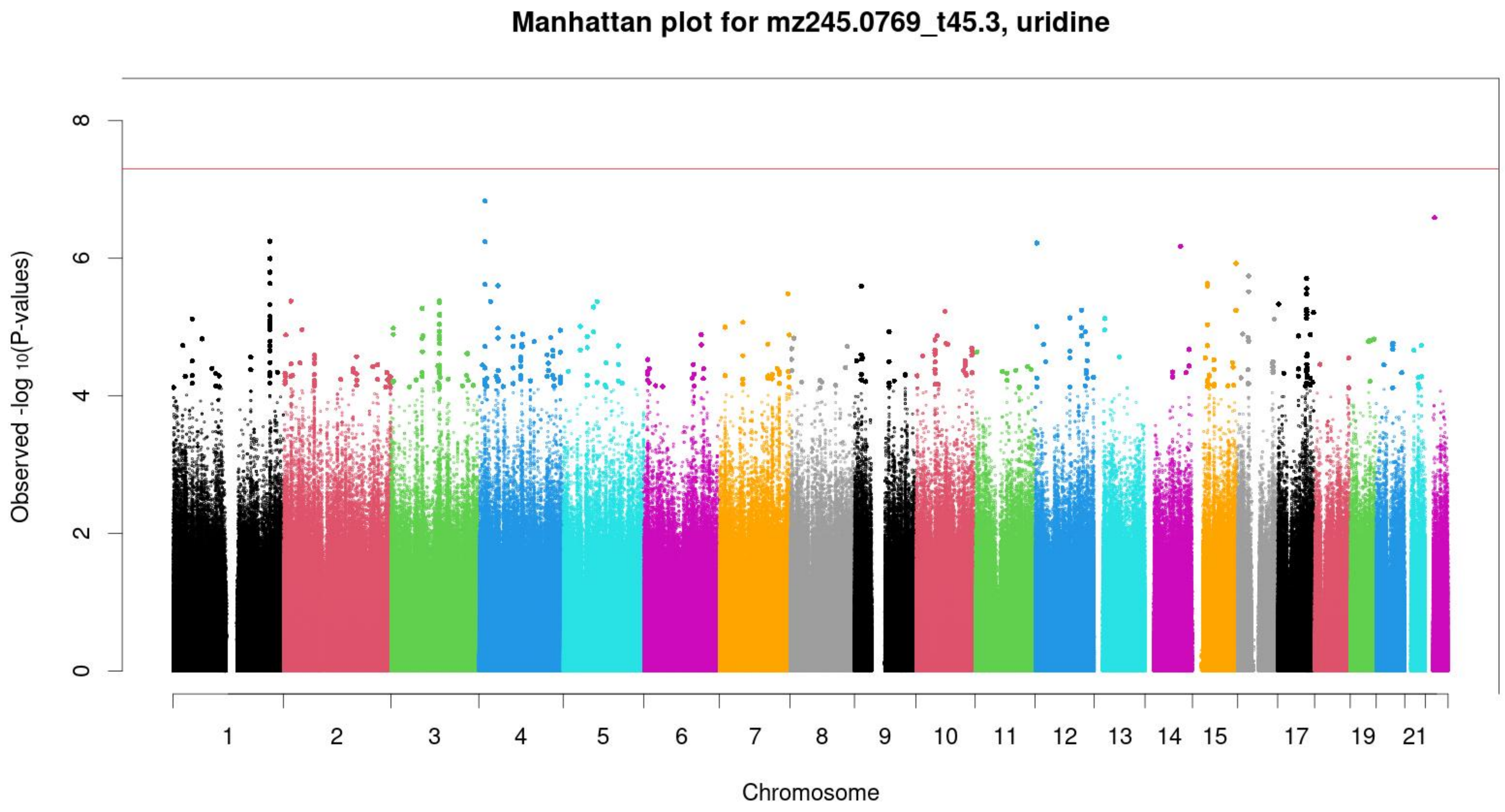

Manhattan plot for mz245.0769_t45.3, uridine
Observed -log 10(P-values)
0
2
4
6
8
1
2
3
4
5
6
7
8
9
10
11
12
13
14
15
17
19
21
Chromosome

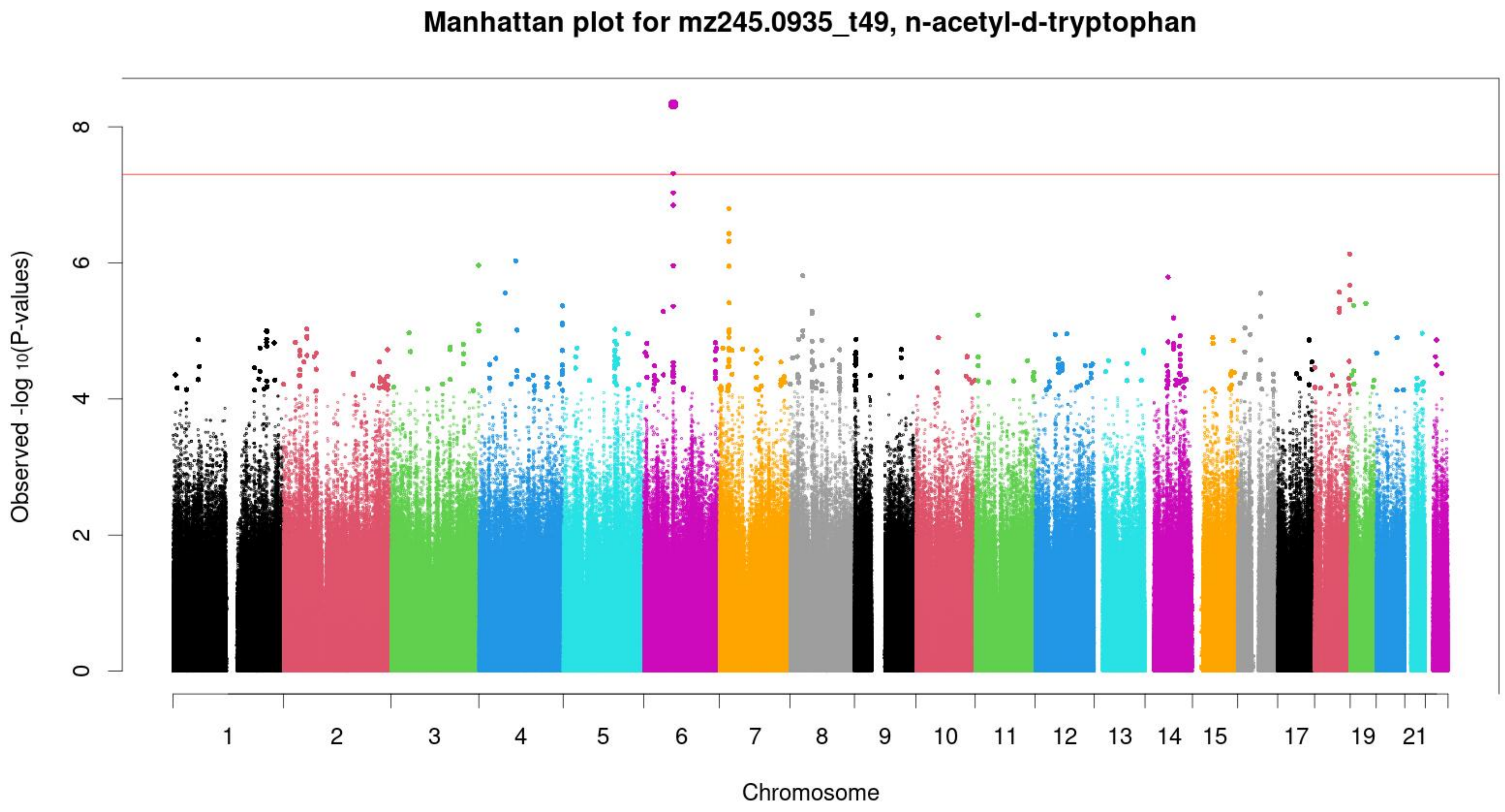
Manhattan plot for mz245.0935_t49, n-acetyl-d-tryptophan
Observed -log 10(P-values)
0
2
4
6
8
1
2
3
4
5
6
7
8
9
10
11
12
13
14
15
17
19
21
Chromosome

Manhattan plot for mz245.0971_t55, biotin

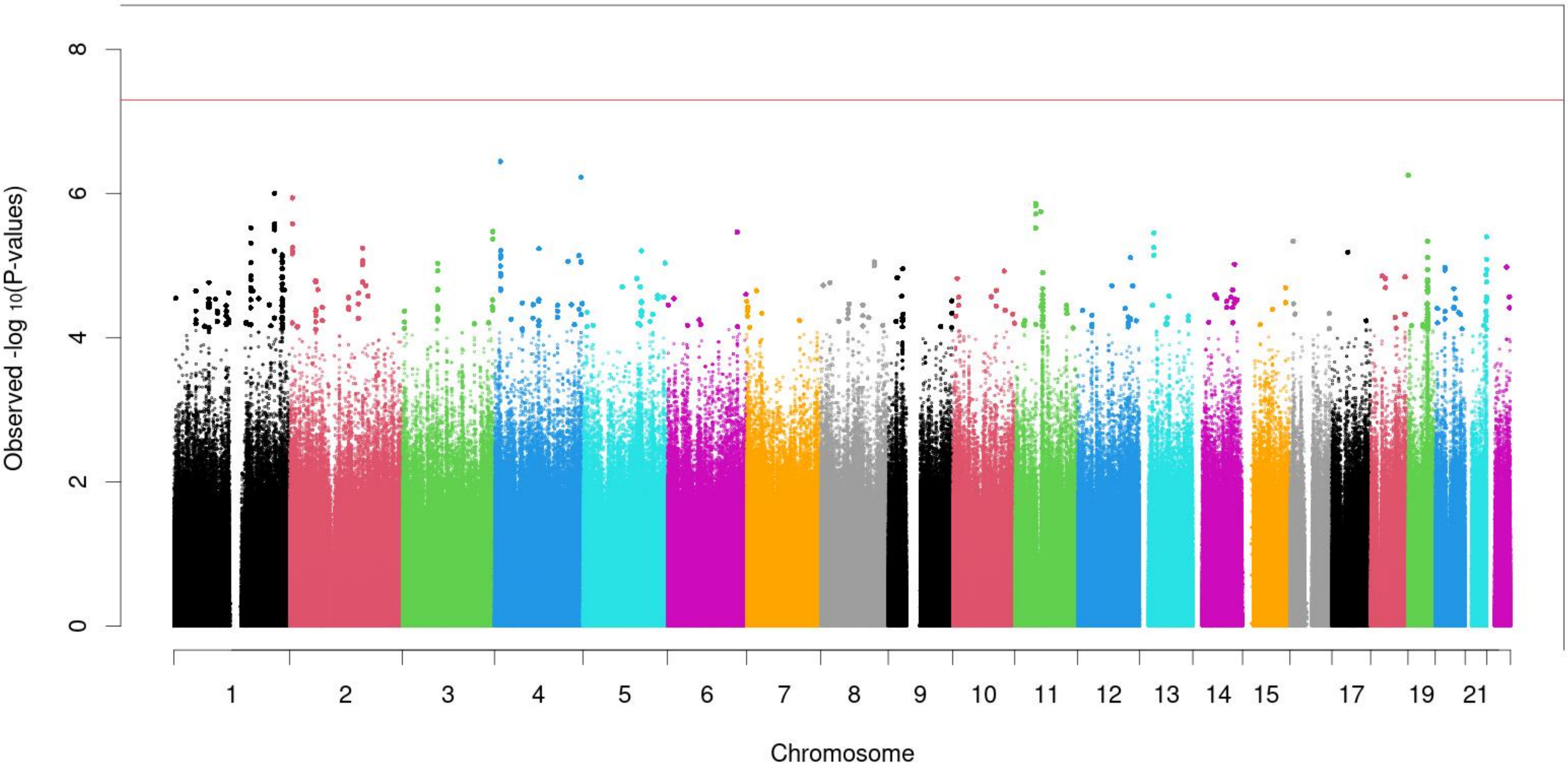

Manhattan plot for mz247.1076_t34.1, n-acetyl-d-tryptophan

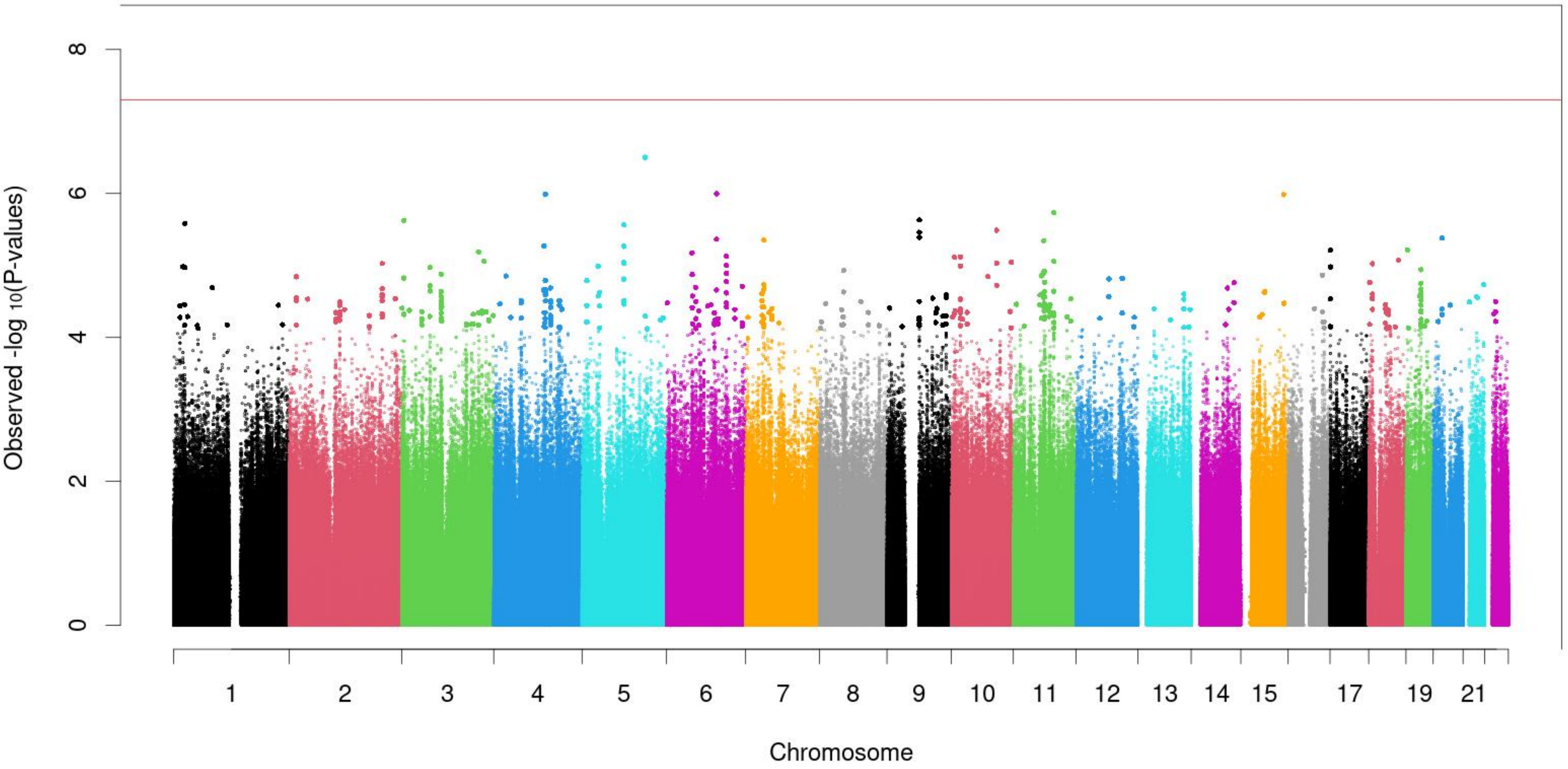

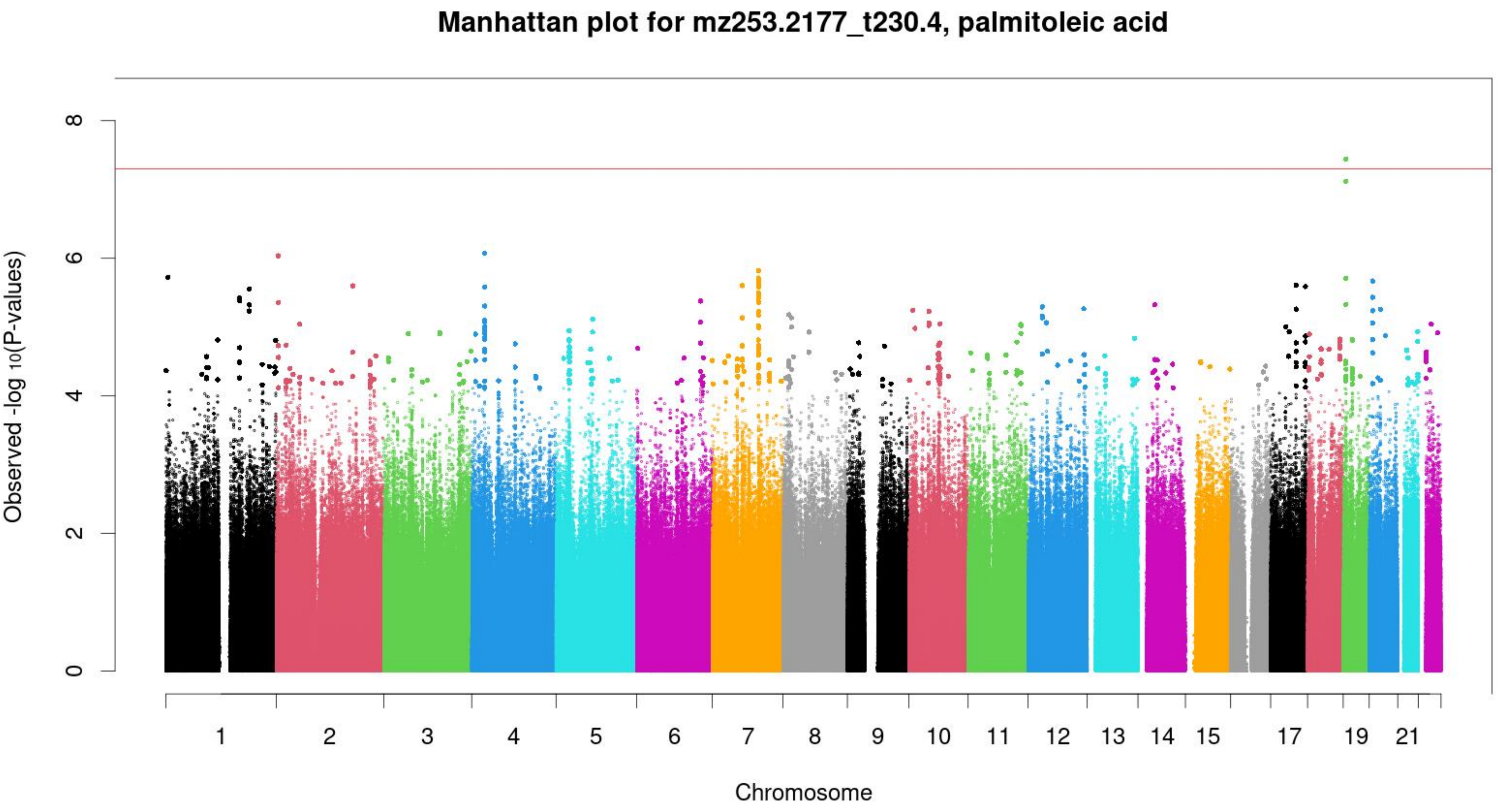

Manhattan plot for mz253.2177_t230.4, palmitoleic acid
Observed -log 10(P-values)
0
2
4
6
8
1
2
3
4
5
6
7
8
9
10
11
12
13
14
15
17
19
21
Chromosome

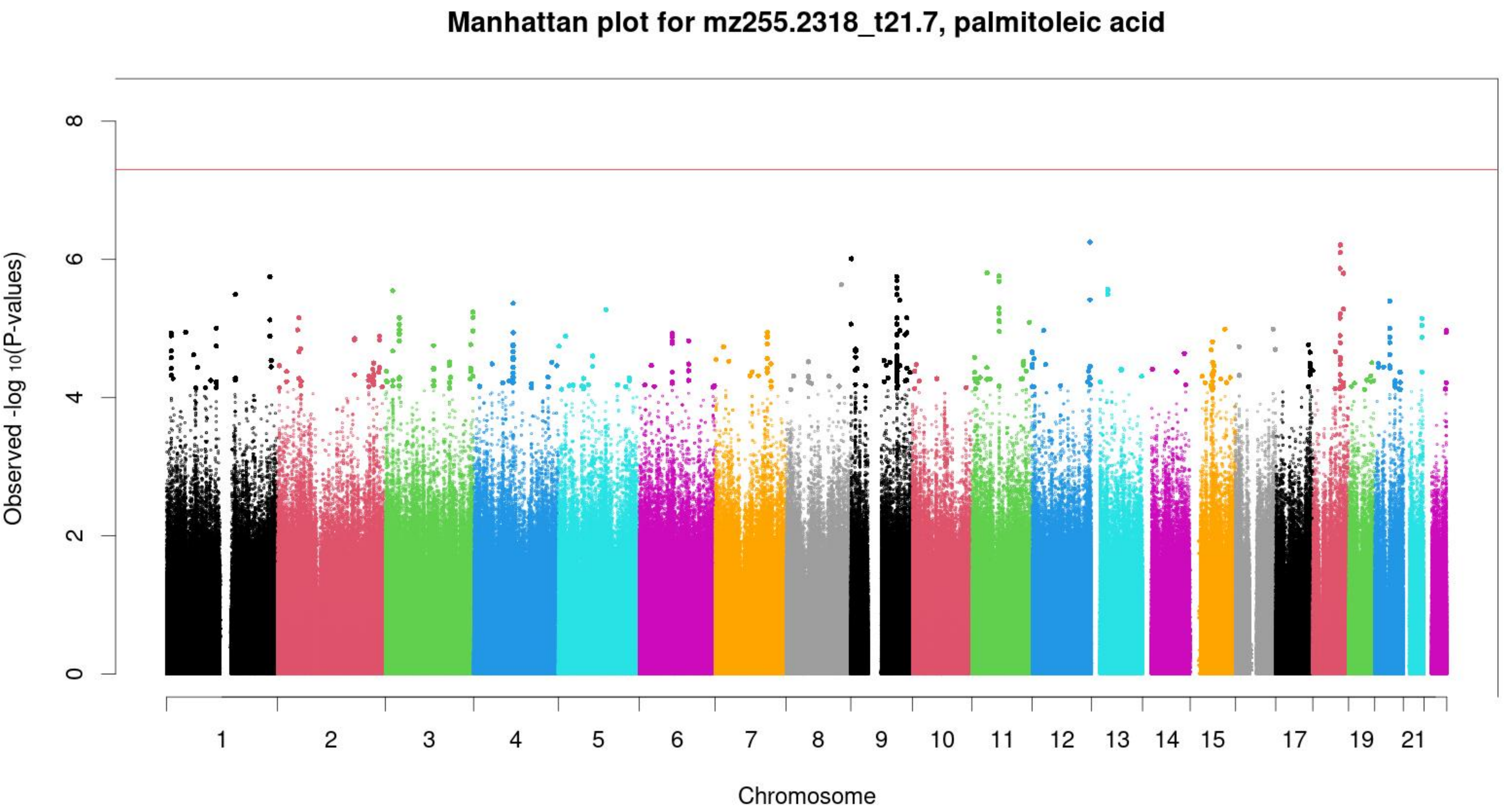
Manhattan plot for mz255.2318_t21.7, palmitoleic acid
Observed -log 10(P-values)
0
2
4
6
8
1
2
3
4
5
6
7
8
9
10
11
12
13
14
15
17
19
21
Chromosome

Manhattan plot for mz258.1102_t114.4, sn-glycero-3-phosphocholine

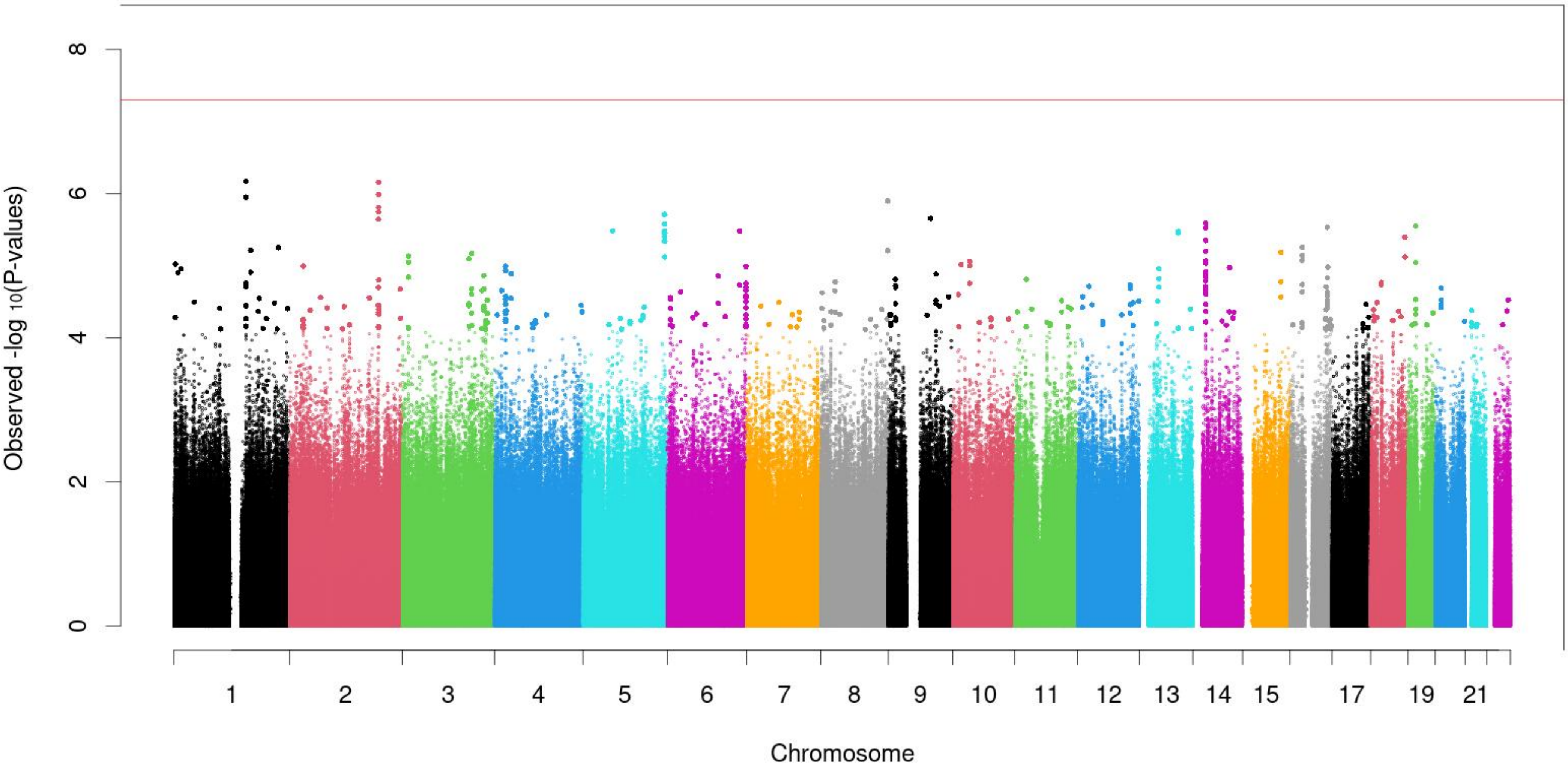

Manhattan plot for mz266.0893_t18.4, adenosine

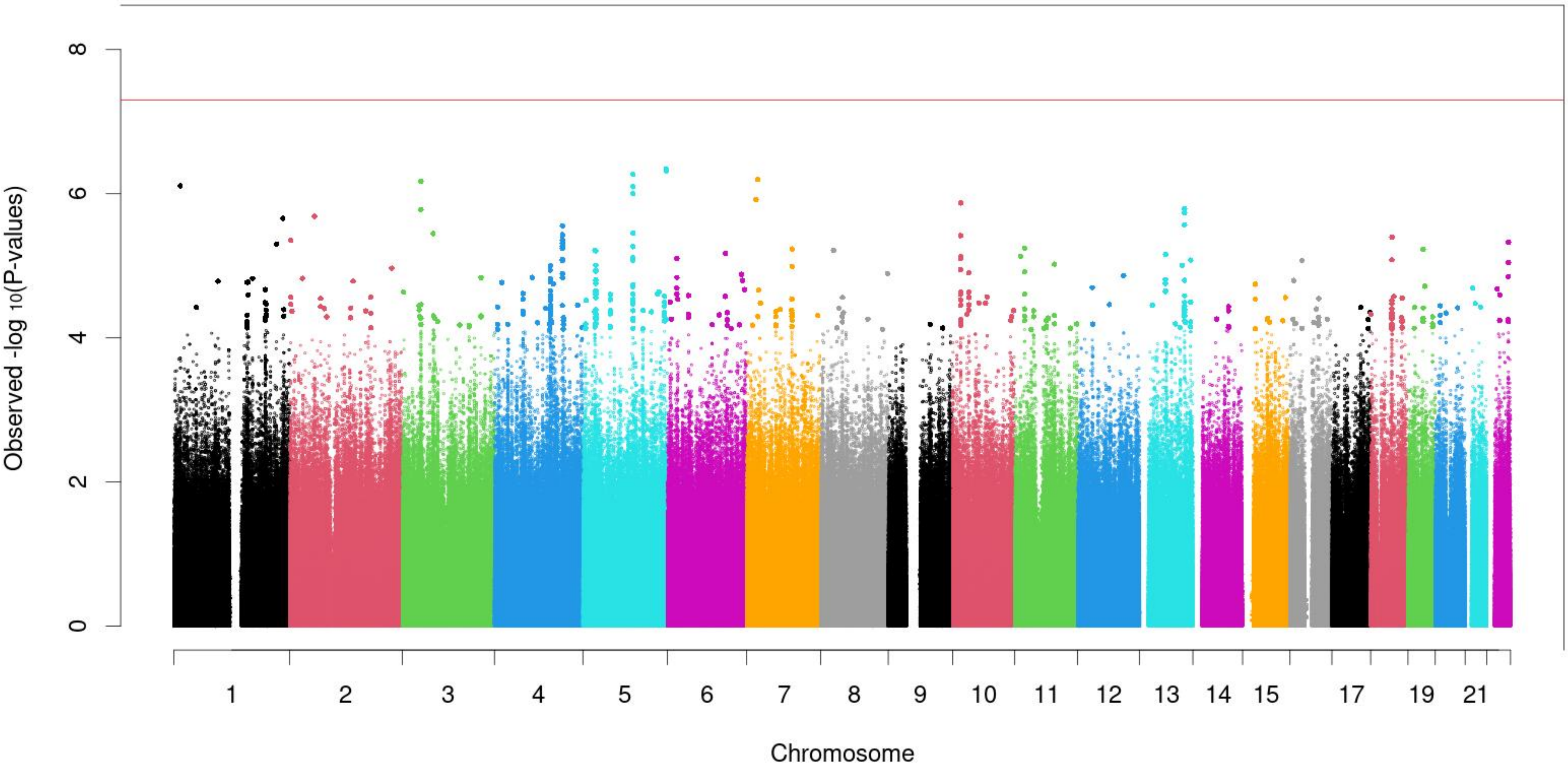

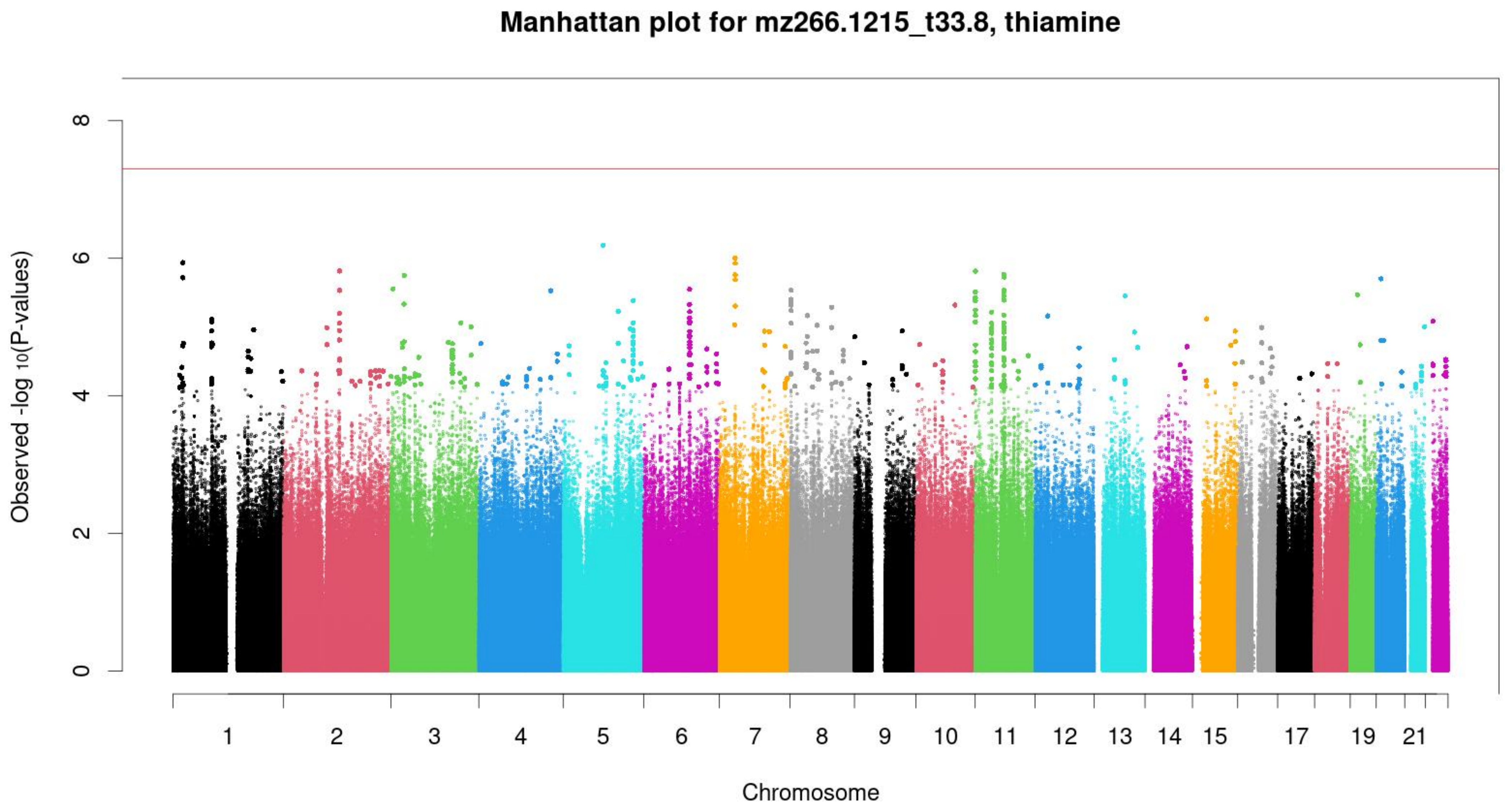
Manhattan plot for mz266.1215_t33.8, thiamine
Observed -log 10(P-values)
0
2
4
6
8
1
2
3
4
5
6
7
8
9
10
11
12
13
14
15
17
19
21
Chromosome

Manhattan plot for mz269.0881_t43.1, inosine

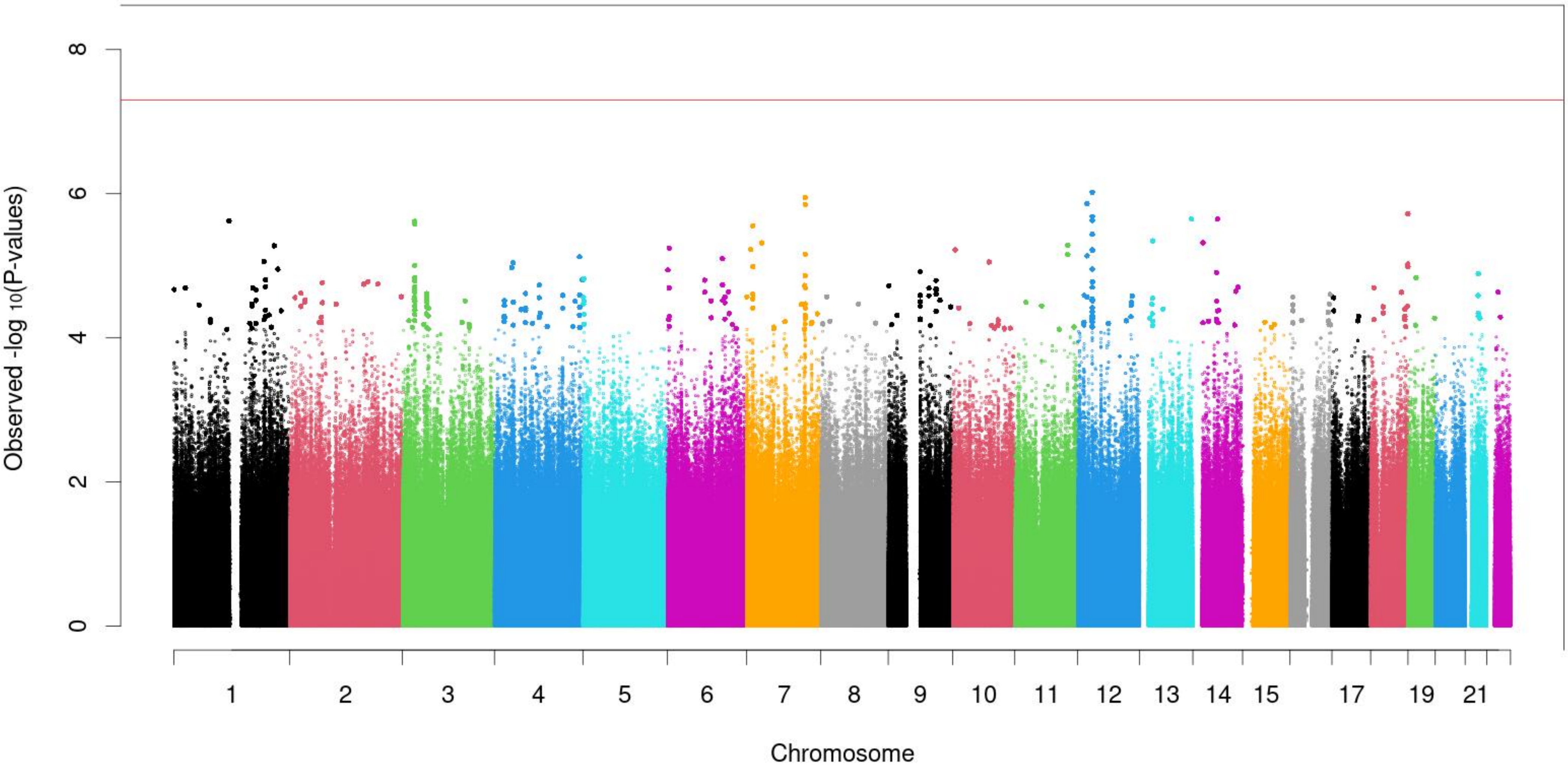

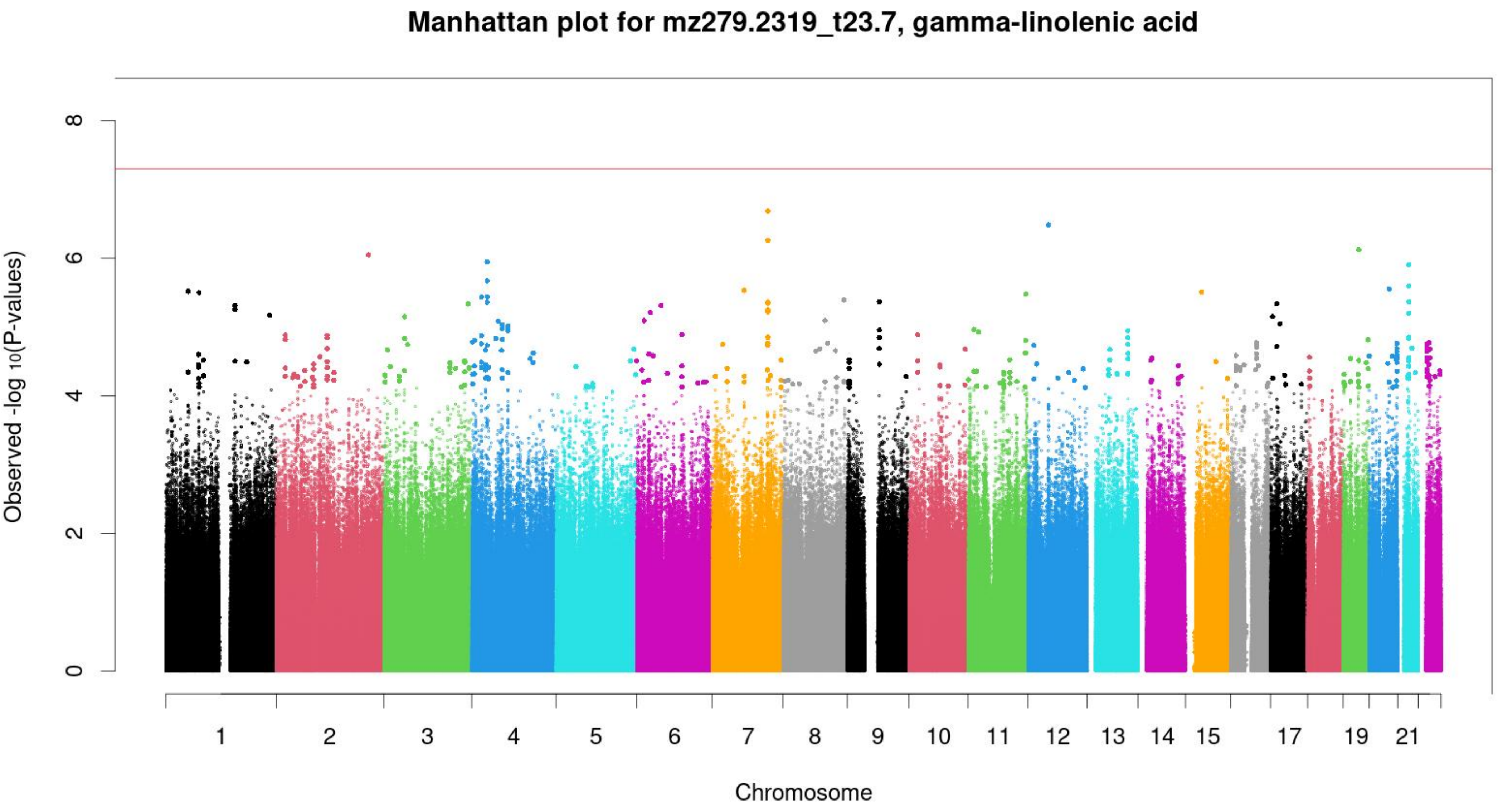

Manhattan plot for mz279.2319_t23.7, gamma-linolenic acid
Observed -log 10(P-values)
0
2
4
6
8
1
2
3
4
5
6
7
8
9
10
11
12
13
14
15
17
19
21
Chromosome

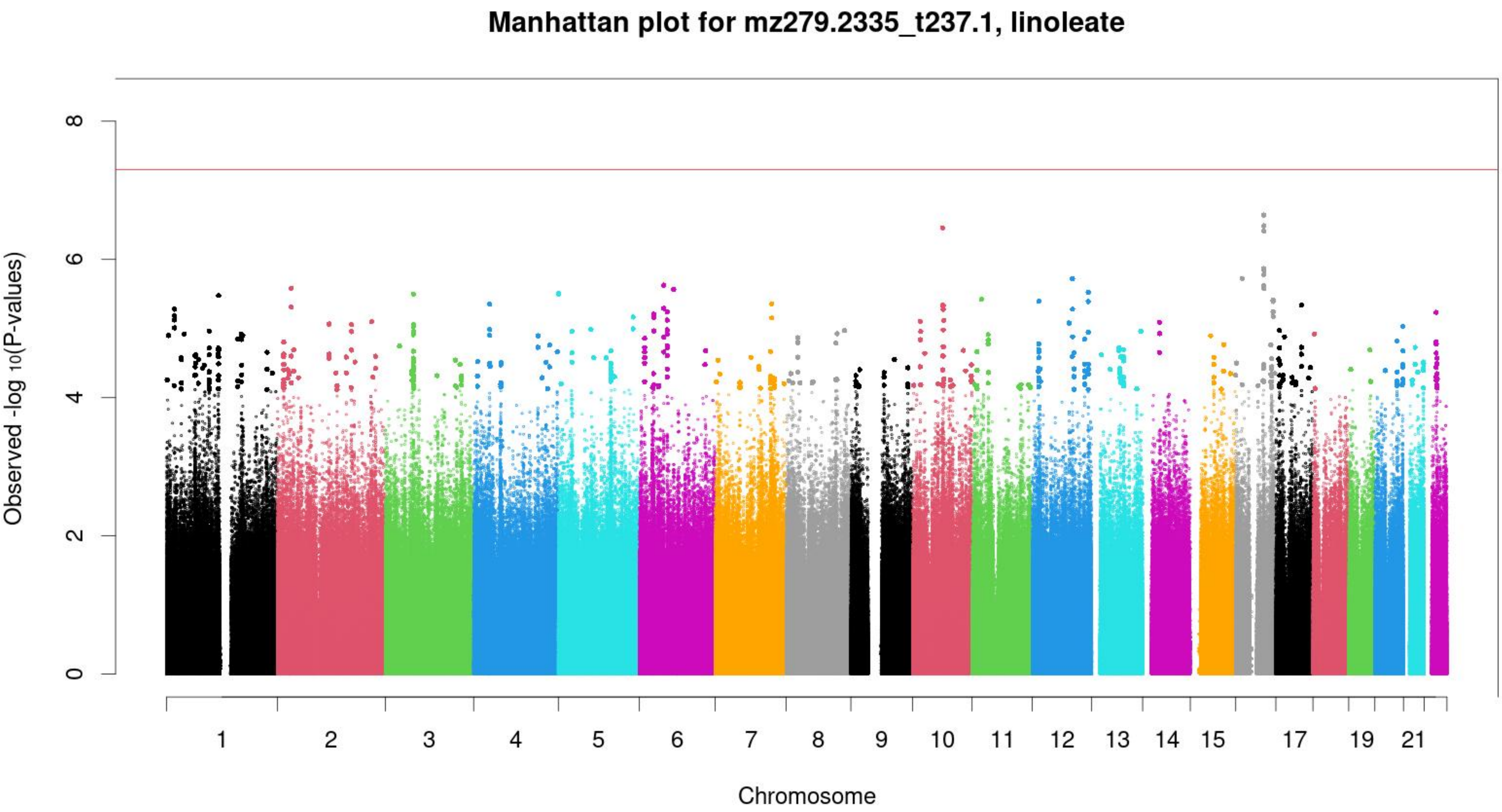

Manhattan plot for mz279.2335_t237.1, linoleate
Observed -log 10(P-values)
0
2
4
6
8
1
2
3
4
5
6
7
8
9
10
11
12
13
14
15
17
19
21
Chromosome

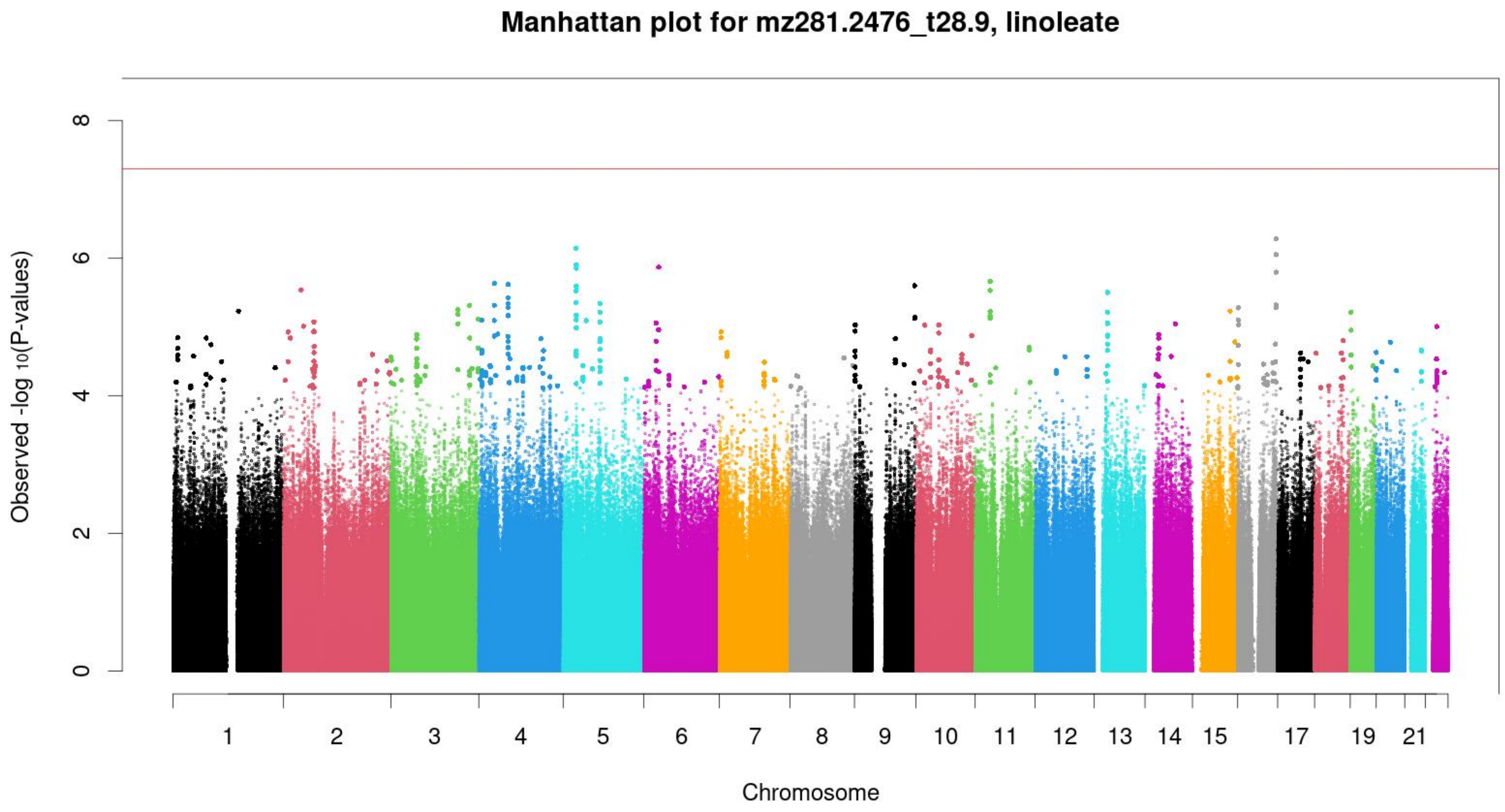
Manhattan plot for mz281.2476_t28.9, linoleate
Observed -log 10(P-values)
0
2
4
6
8
1
2
3
4
5
6
7
8
9
10
11
12
13
14
15
17
19
21
Chromosome

Manhattan plot for mz281.2493_t256.8, elaidic acid

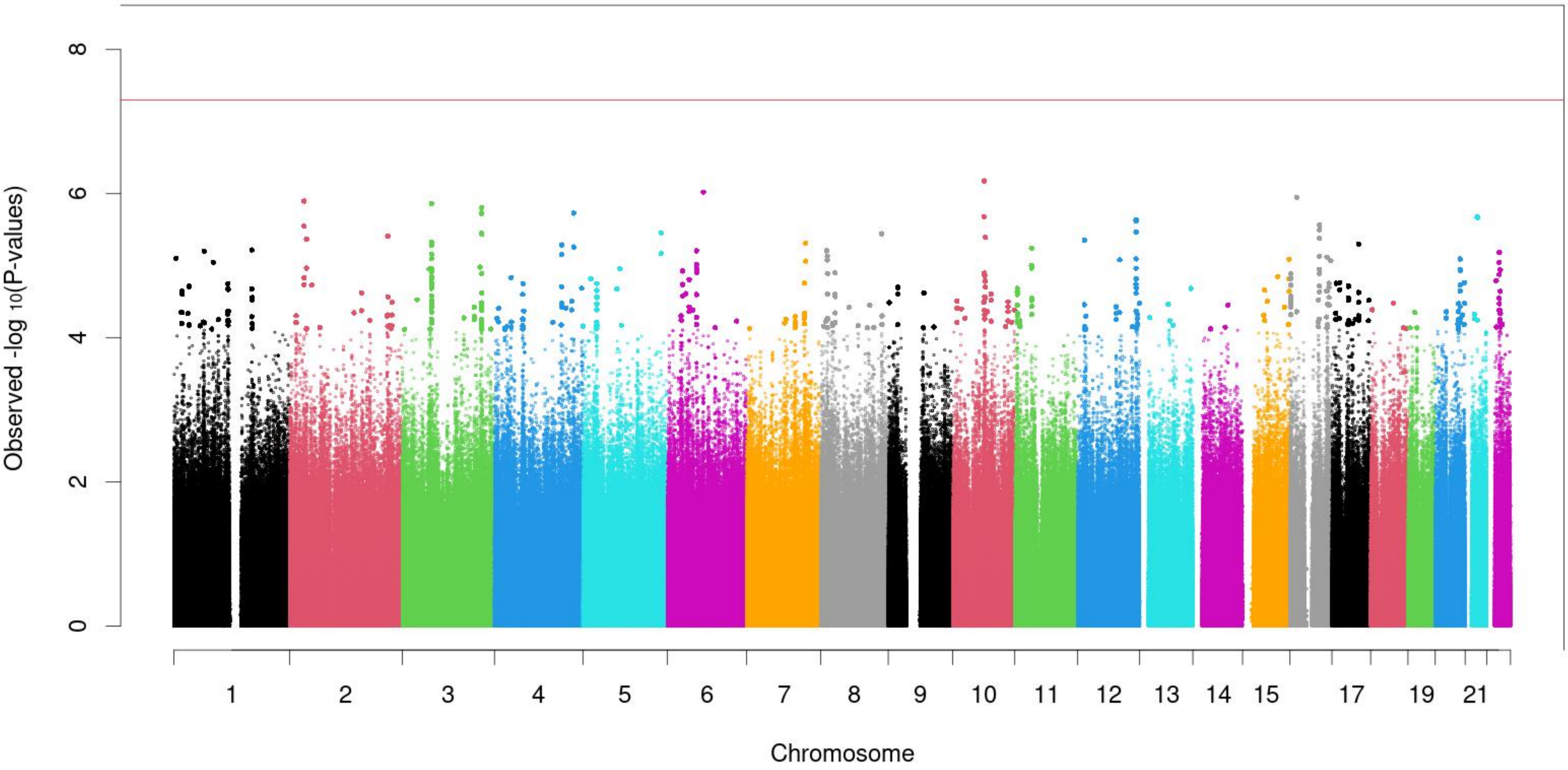

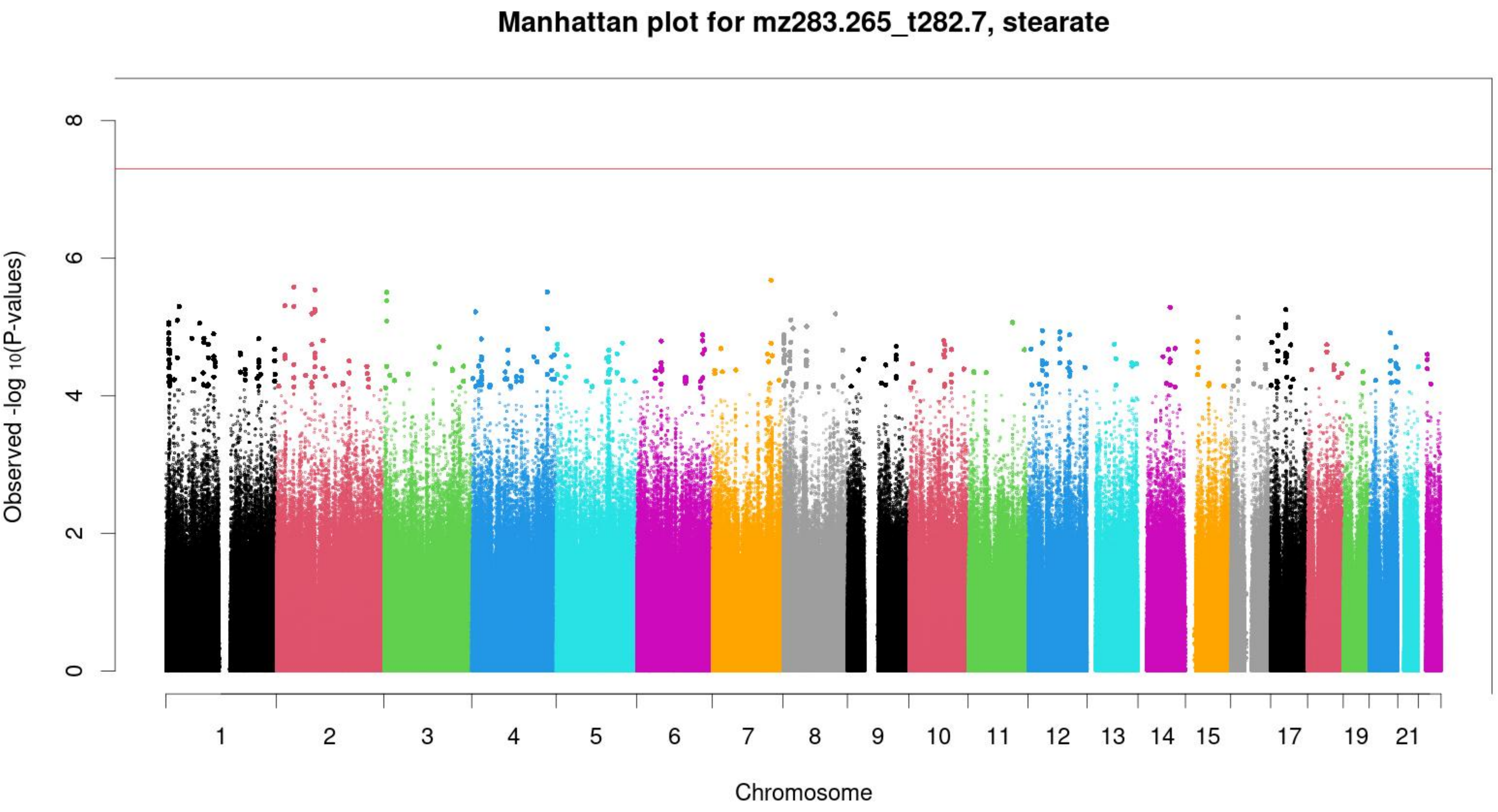

Manhattan plot for mz283.265_t282.7, stearate
Observed -log 10(P-values)
0
2
4
6
8
1
2
3
4
5
6
7
8
9
10
11
12
13
14
15
17
19
21
Chromosome

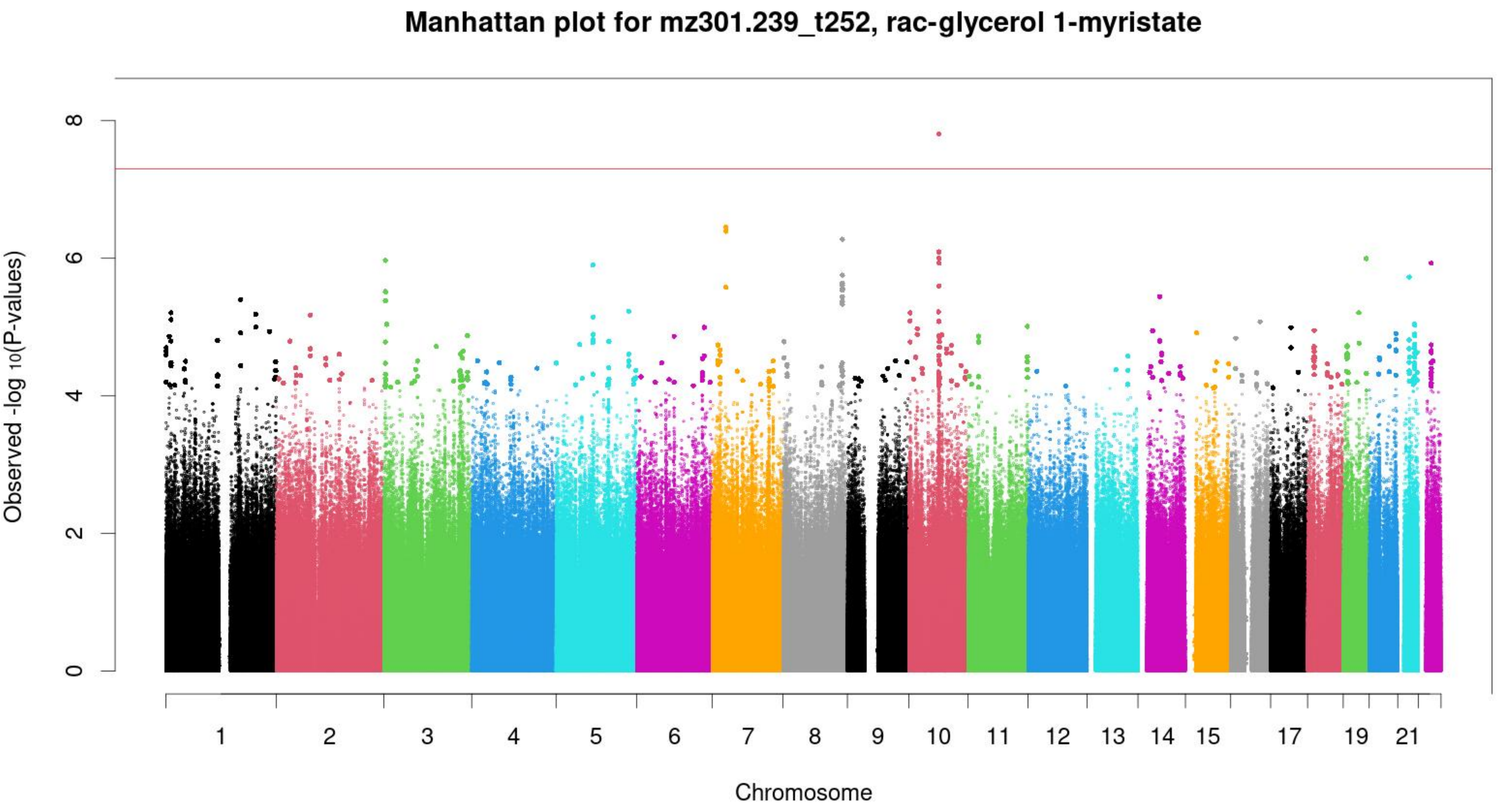

Manhattan plot for mz301.239_t252, rac-glycerol 1-myristate
Observed -log 10(P-values)
0
2
4
6
8
1
2
3
4
5
6
7
8
9
10
11
12
13
14
15
17
19
21
Chromosome

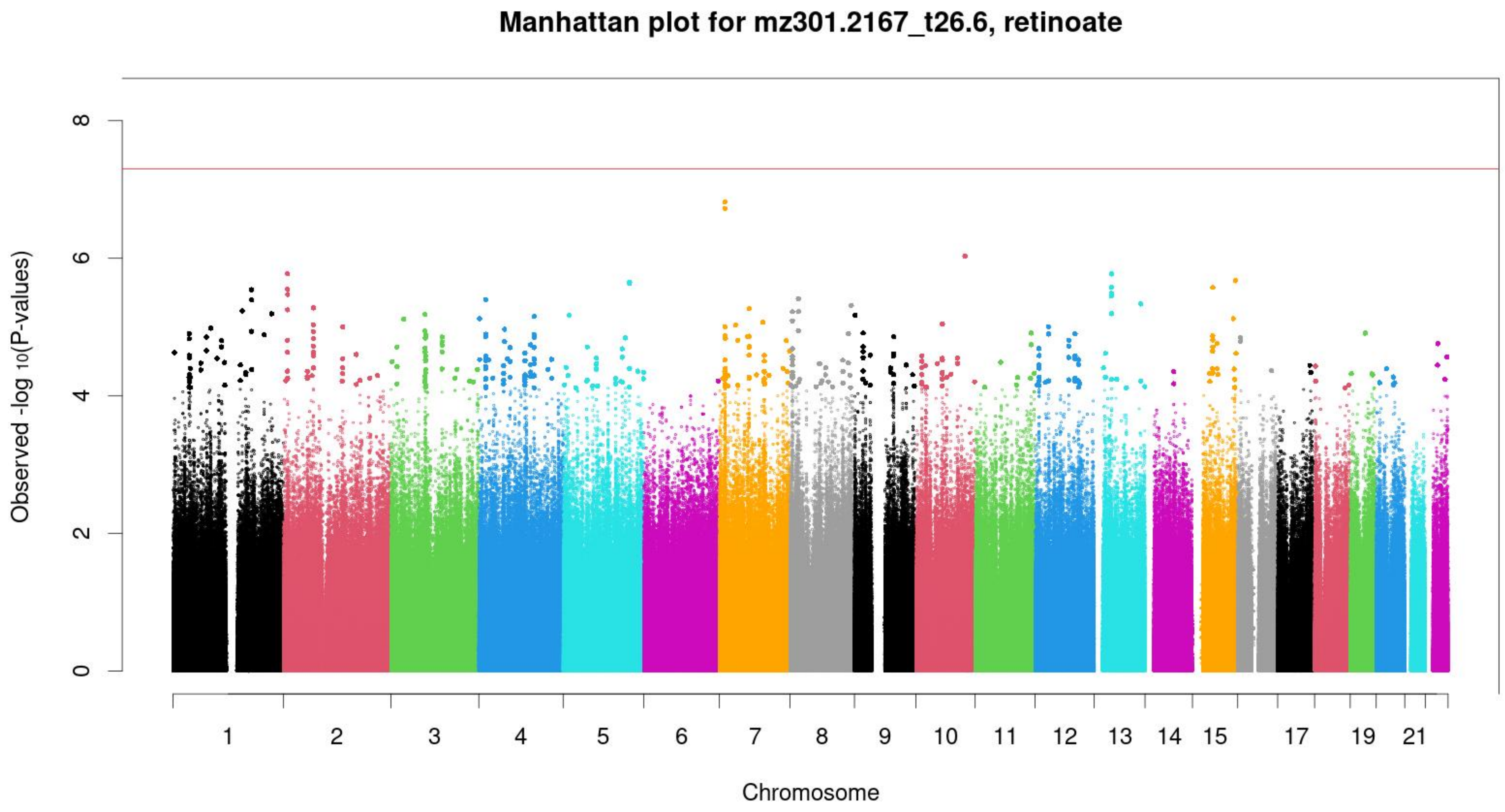

Manhattan plot for mz301.2167_t26.6, retinoate
Observed -log 10(P-values)
0
2
4
6
8
1
2
3
4
5
6
7
8
9
10
11
12
13
14
15
17
19
21
Chromosome

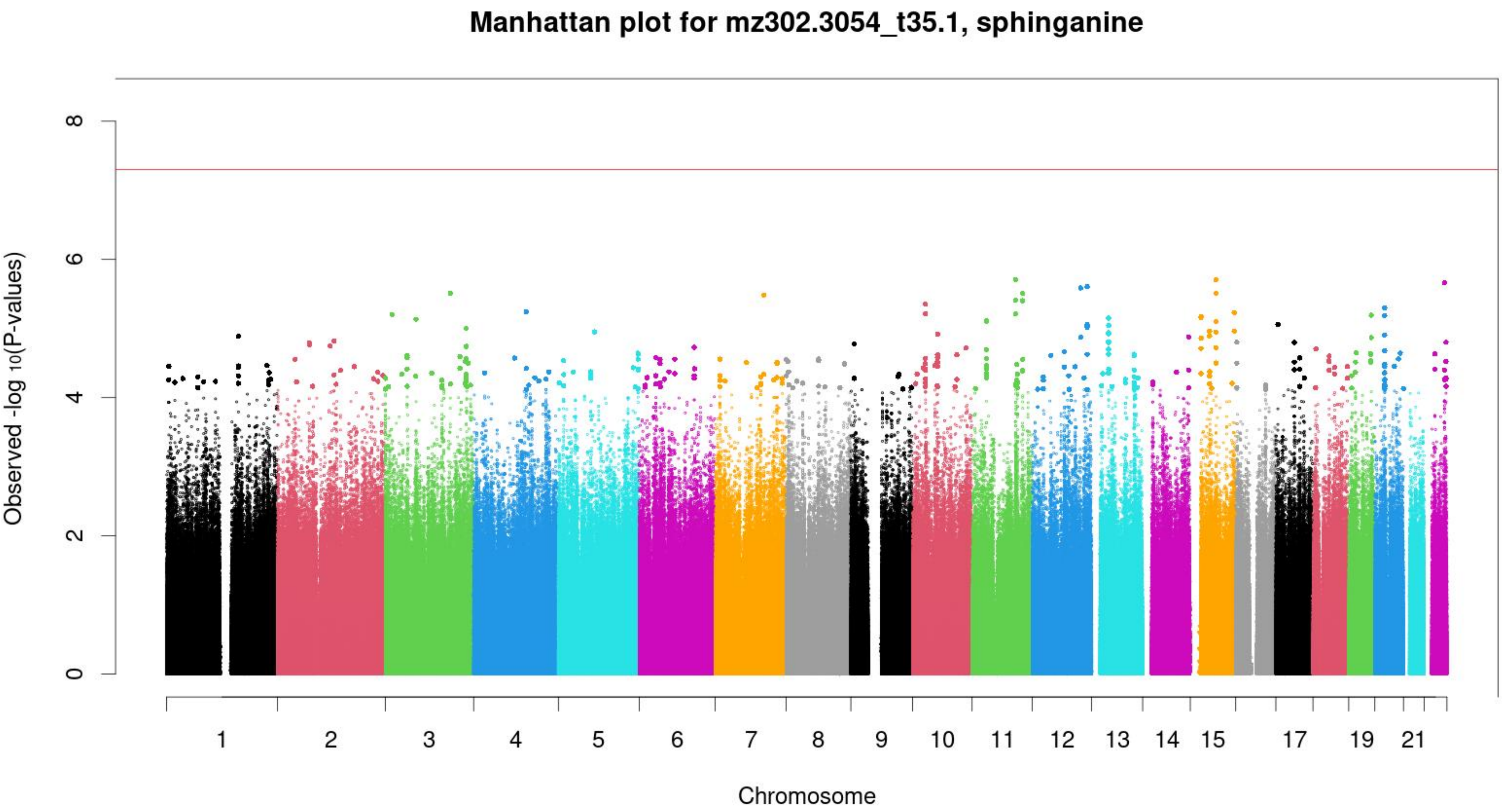

Manhattan plot for mz302.3054_t35.1, sphinganine
Observed -log 10(P-values)
0
2
4
6
8
1
2
3
4
5
6
7
8
9
10
11
12
13
14
15
17
19
21
Chromosome

Manhattan plot for mz307.0316_t28.5, 2'-deoxyuridine 5'-mono-phos-phate

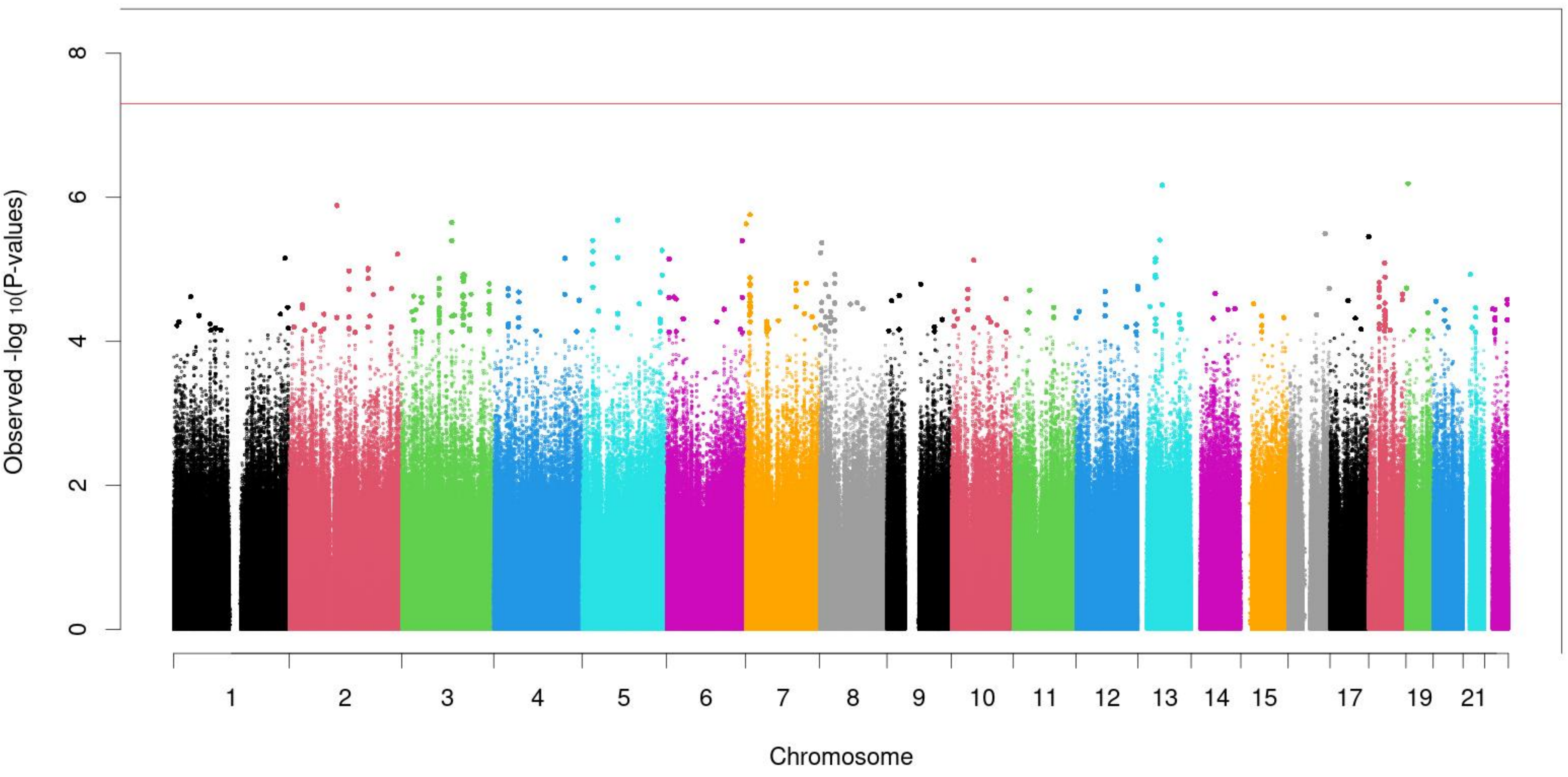

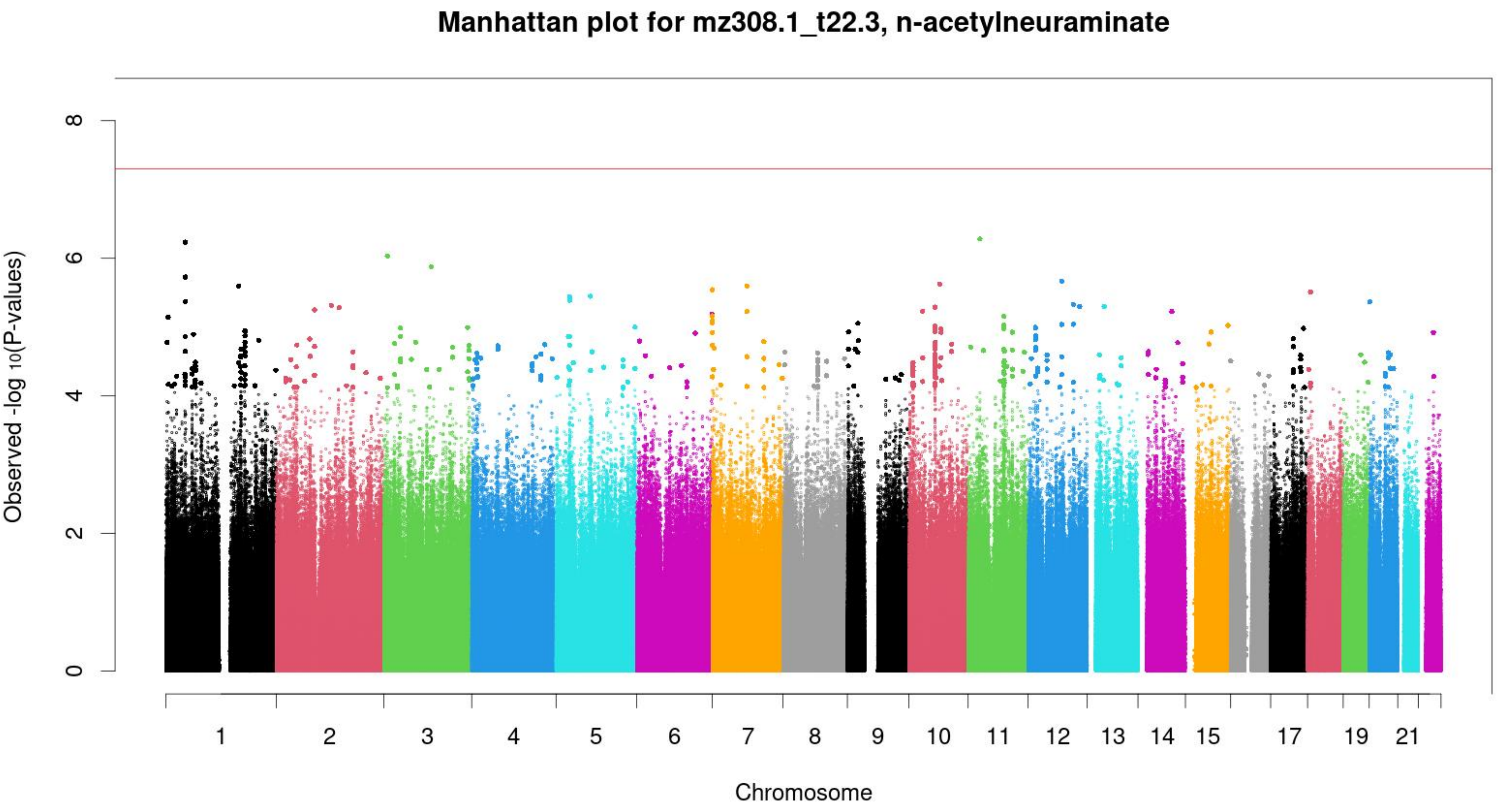

Manhattan plot for mz308.1_t22.3, n-acetylneuraminate
Observed -log 10(P-values)
0
2
4
6
8
1
2
3
4
5
6
7
8
9
10
11
12
13
14
15
17
19
21
Chromosome

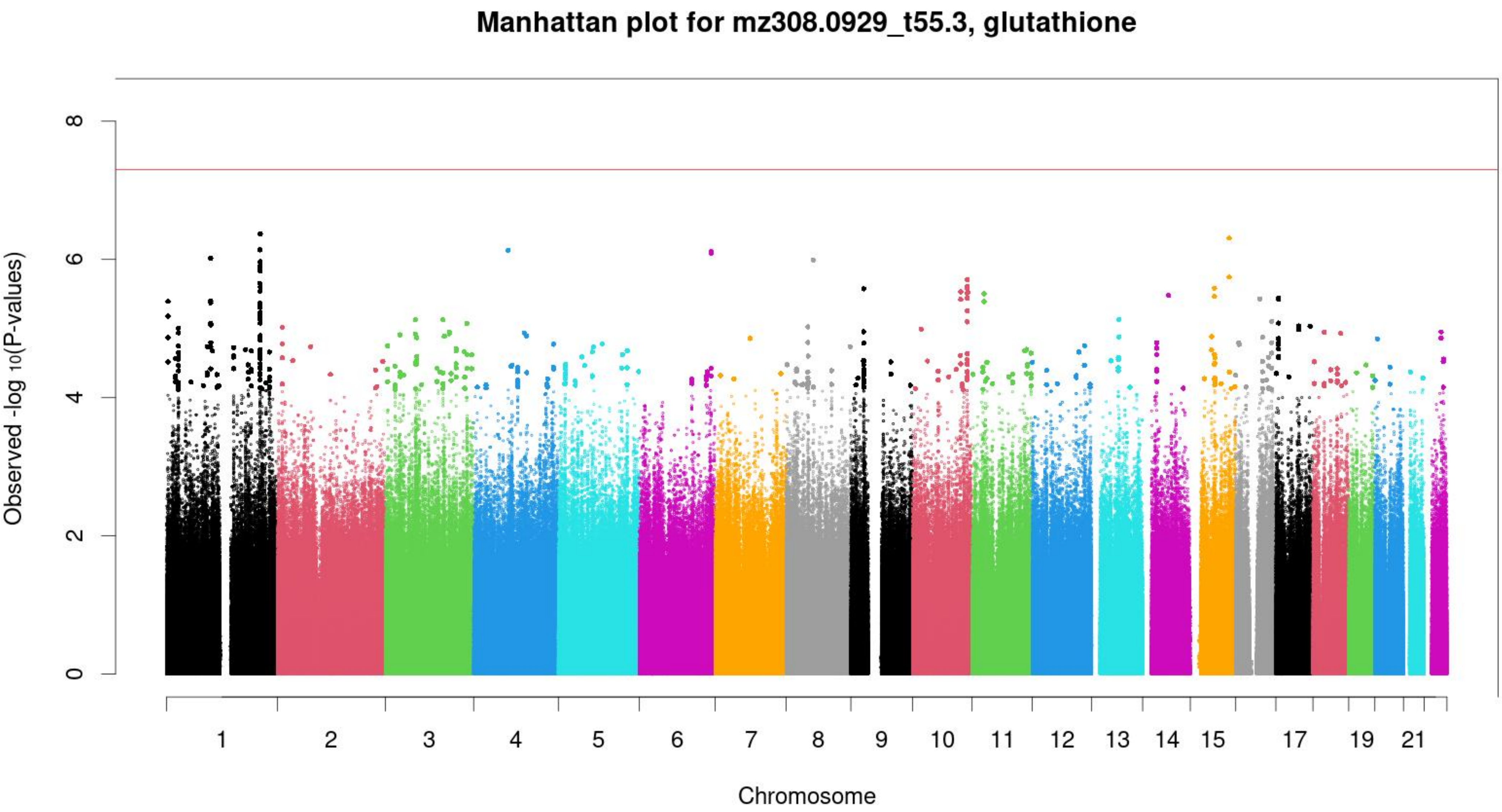
Manhattan plot for mz308.0929_t55.3, glutathione
Observed -log 10(P-values)
0
2
4
6
8
1
2
3
4
5
6
7
8
9
10
11
12
13
14
15
17
19
21
Chromosome

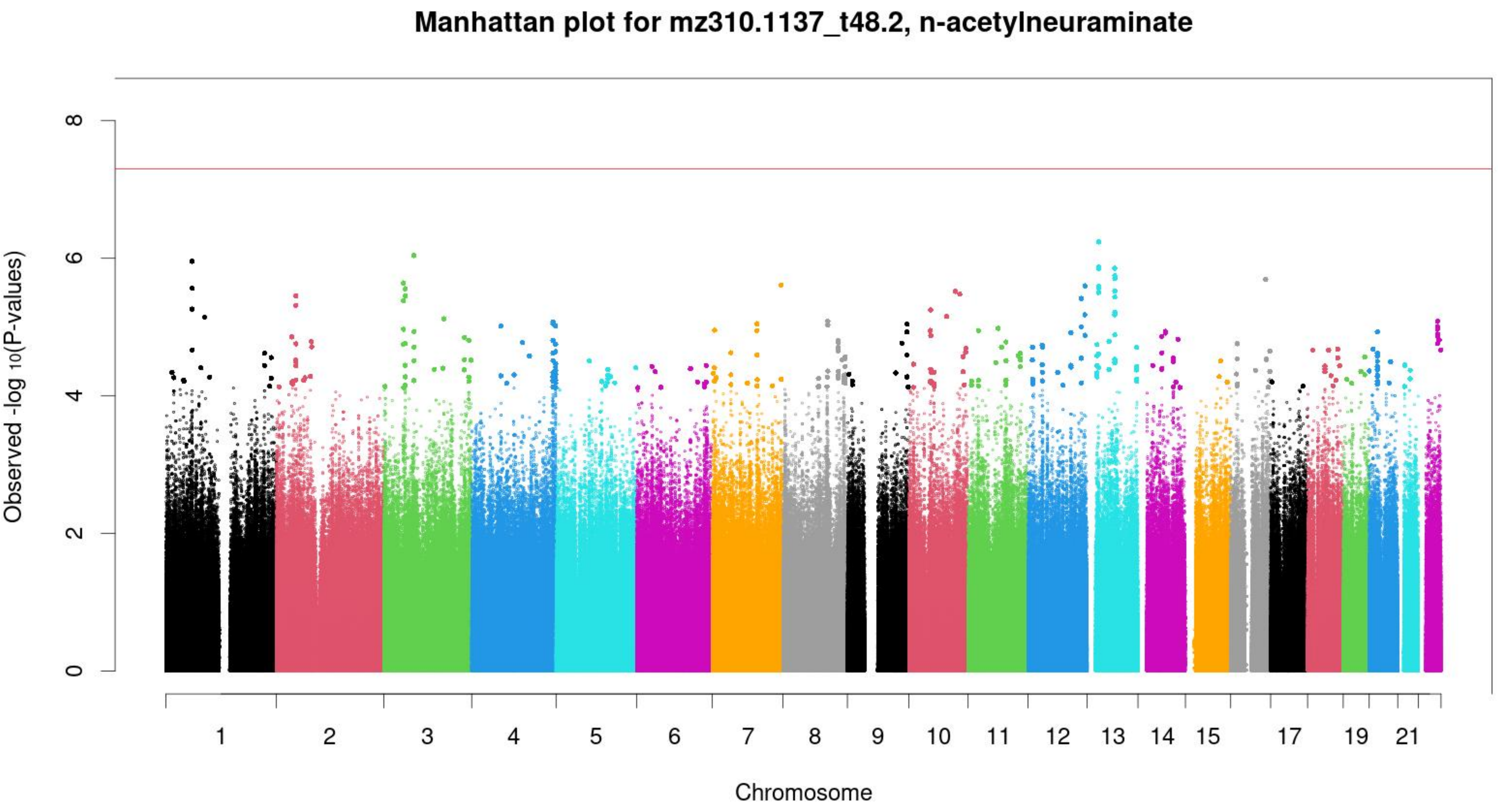

Manhattan plot for mz310.1137_t48.2, n-acetylneuraminate
Observed -log 10(P-values)
0
2
4
6
8
1
2
3
4
5
6
7
8
9
10
11
12
13
14
15
17
19
21
Chromosome

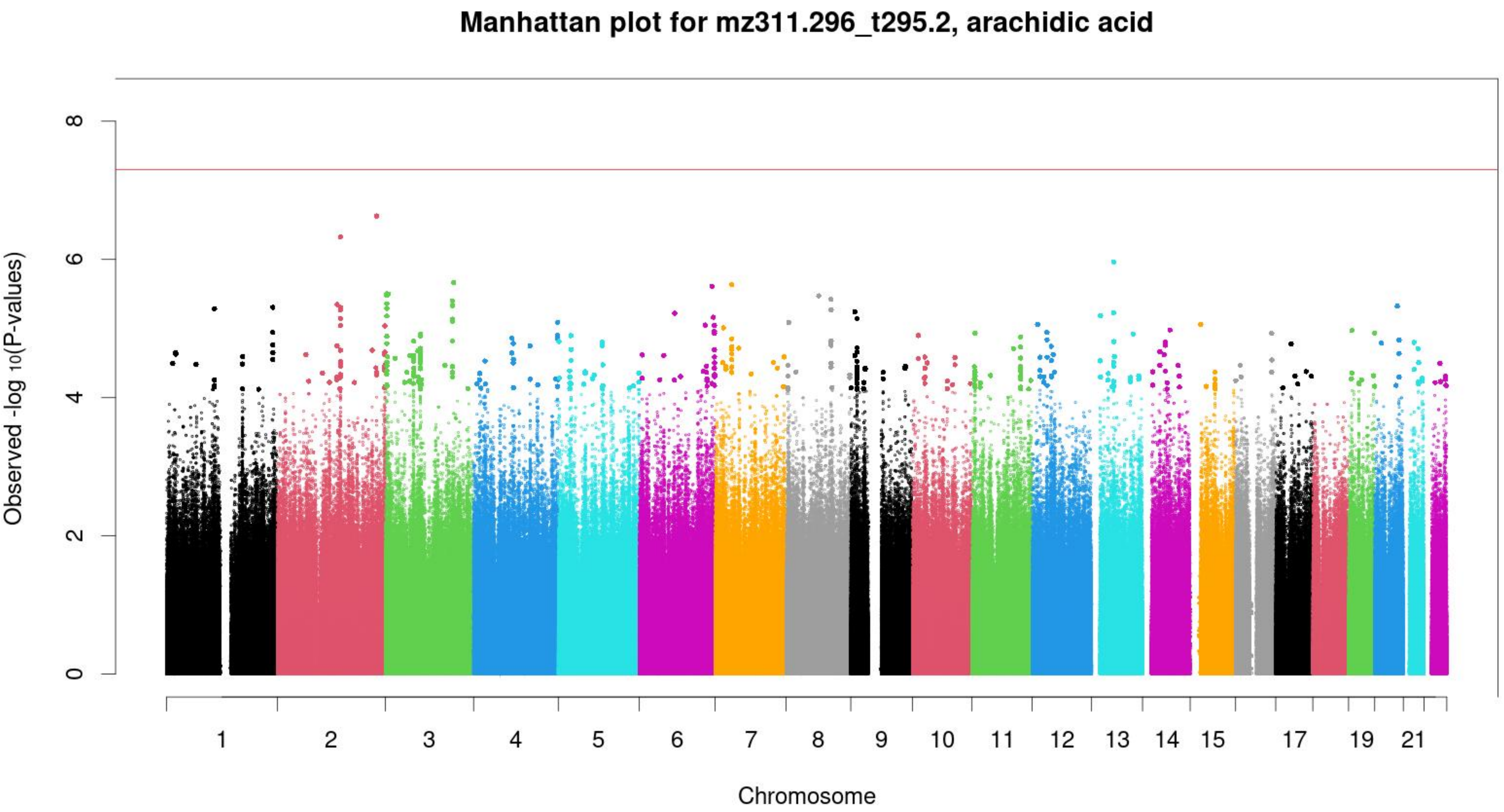
Manhattan plot for mz311.296_t295.2, arachidic acid
Observed -log 10(P-values)
0
2
4
6
8
1
2
3
4
5
6
7
8
9
10
11
12
13
14
15
17
19
21
Chromosome

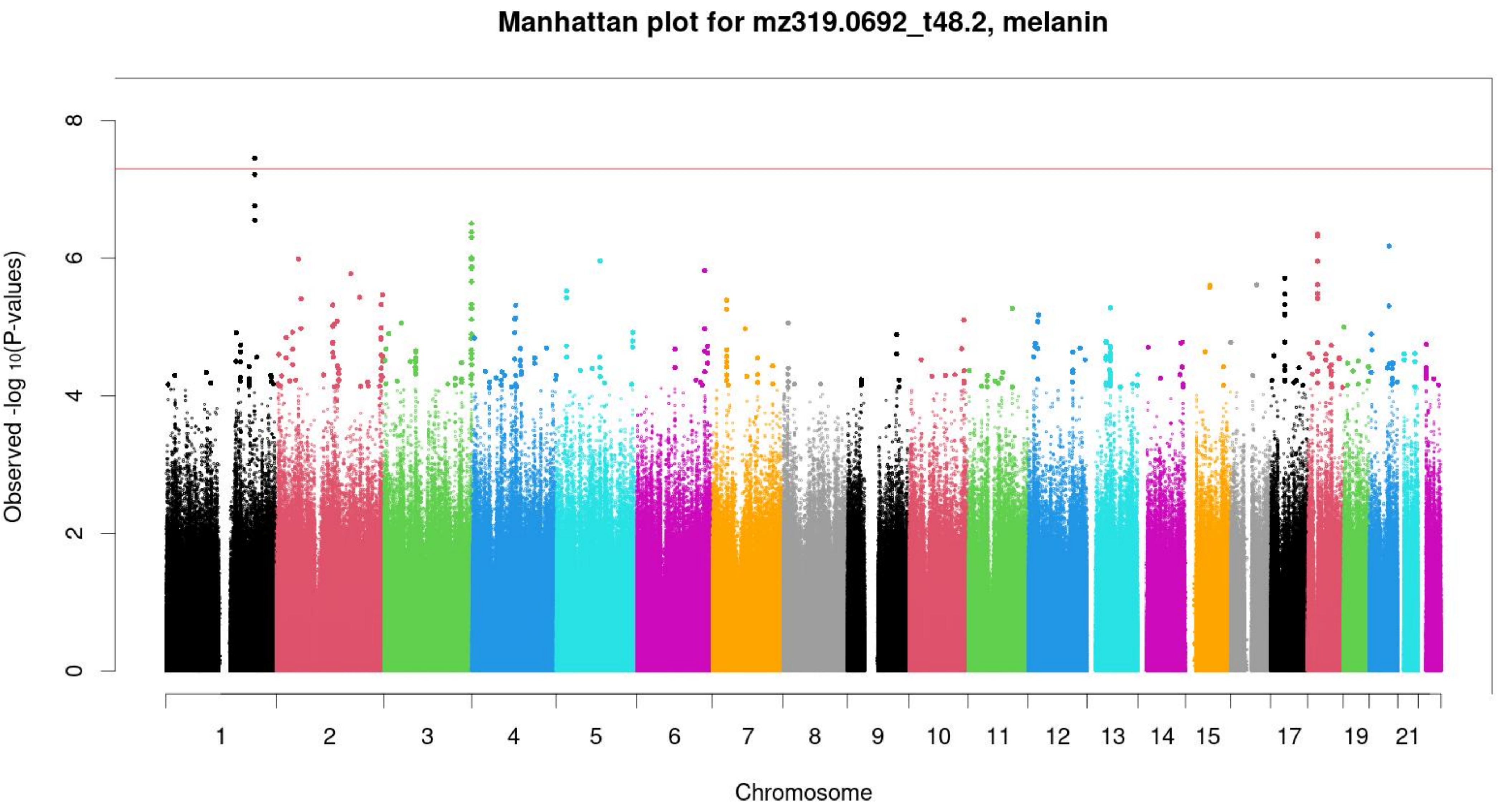

Manhattan plot for mz319.0692_t48.2, melanin
Observed -log 10(P-values)
0
2
4
6
8
1
2
3
4
5
6
7
8
9
10
11
12
13
14
15
17
19
21
Chromosome

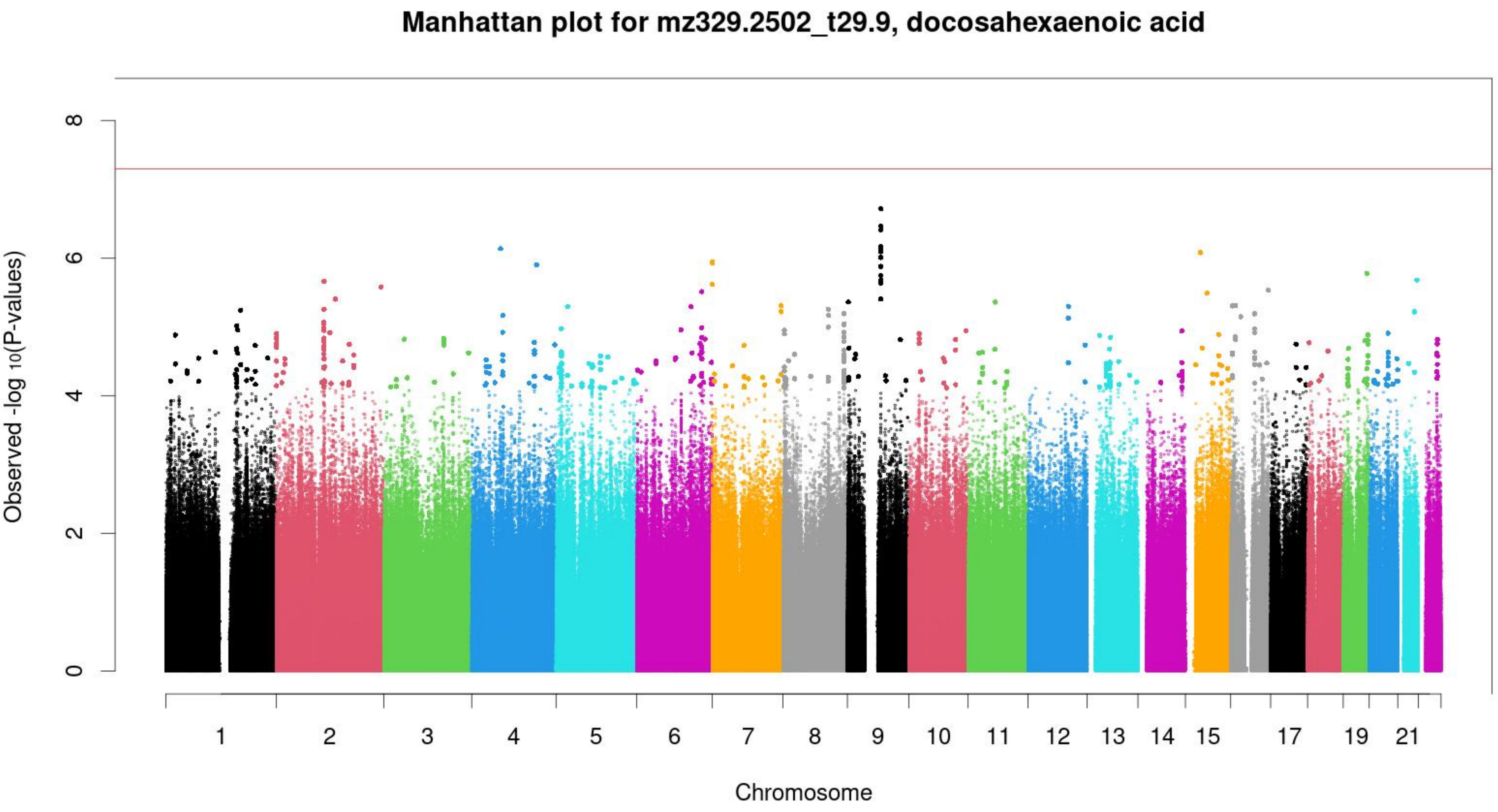
Manhattan plot for mz329.2502_t29.9, docosahexaenoic acid
Observed -log 10(P-values)
0
2
4
6
8
Chromosome
1
2
3
4
5
6
7
8
9
10
11
12
13
14
15
17
19
21

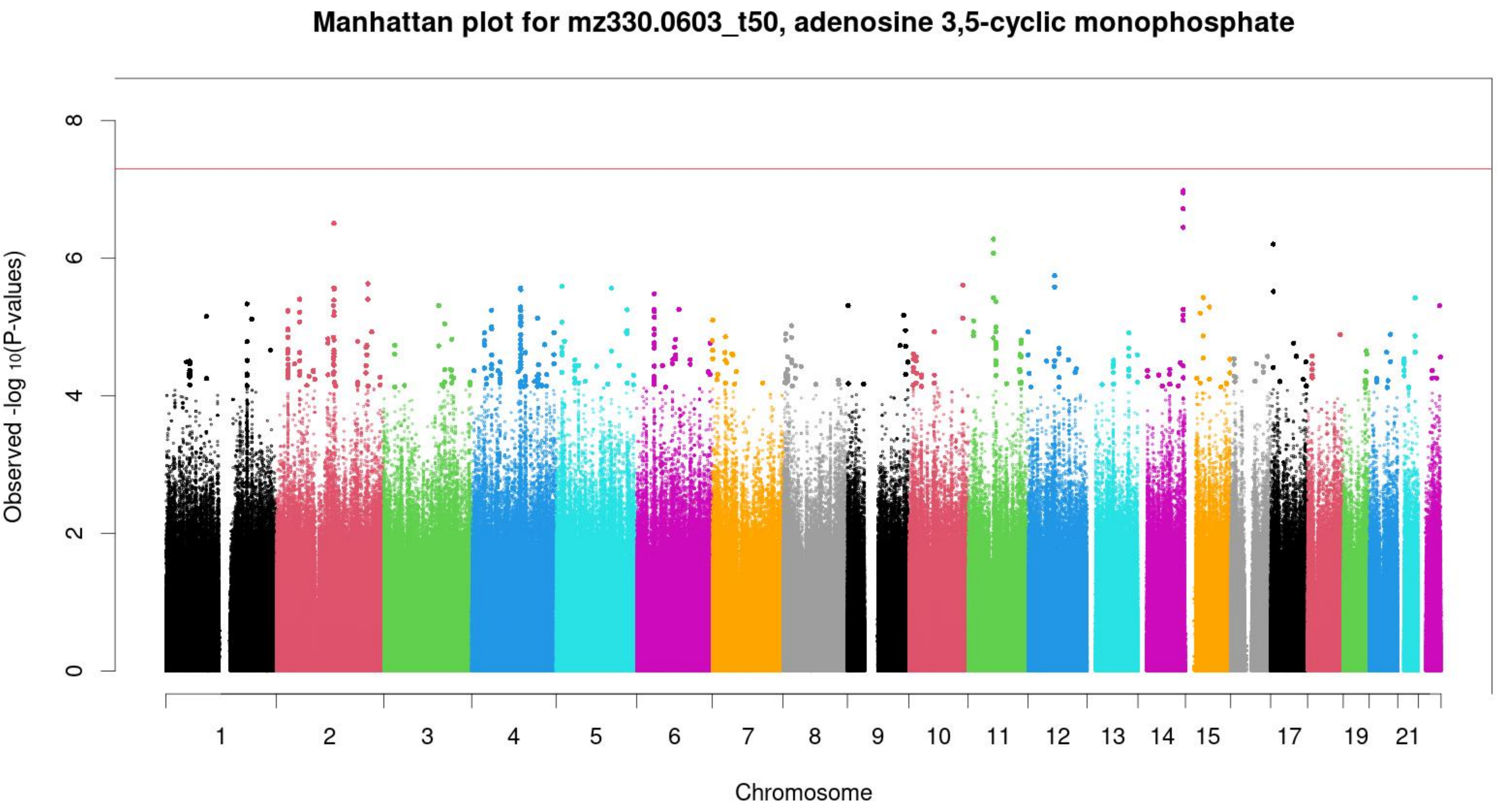
Manhattan plot for mz330.0603_t50, adenosine 3,5-cyclic monophosphate
Observed -log 10(P-values)
0
2
4
6
8
1
2
3
4
5
6
7
8
9
10
11
12
13
14
15
17
19
21
Chromosome

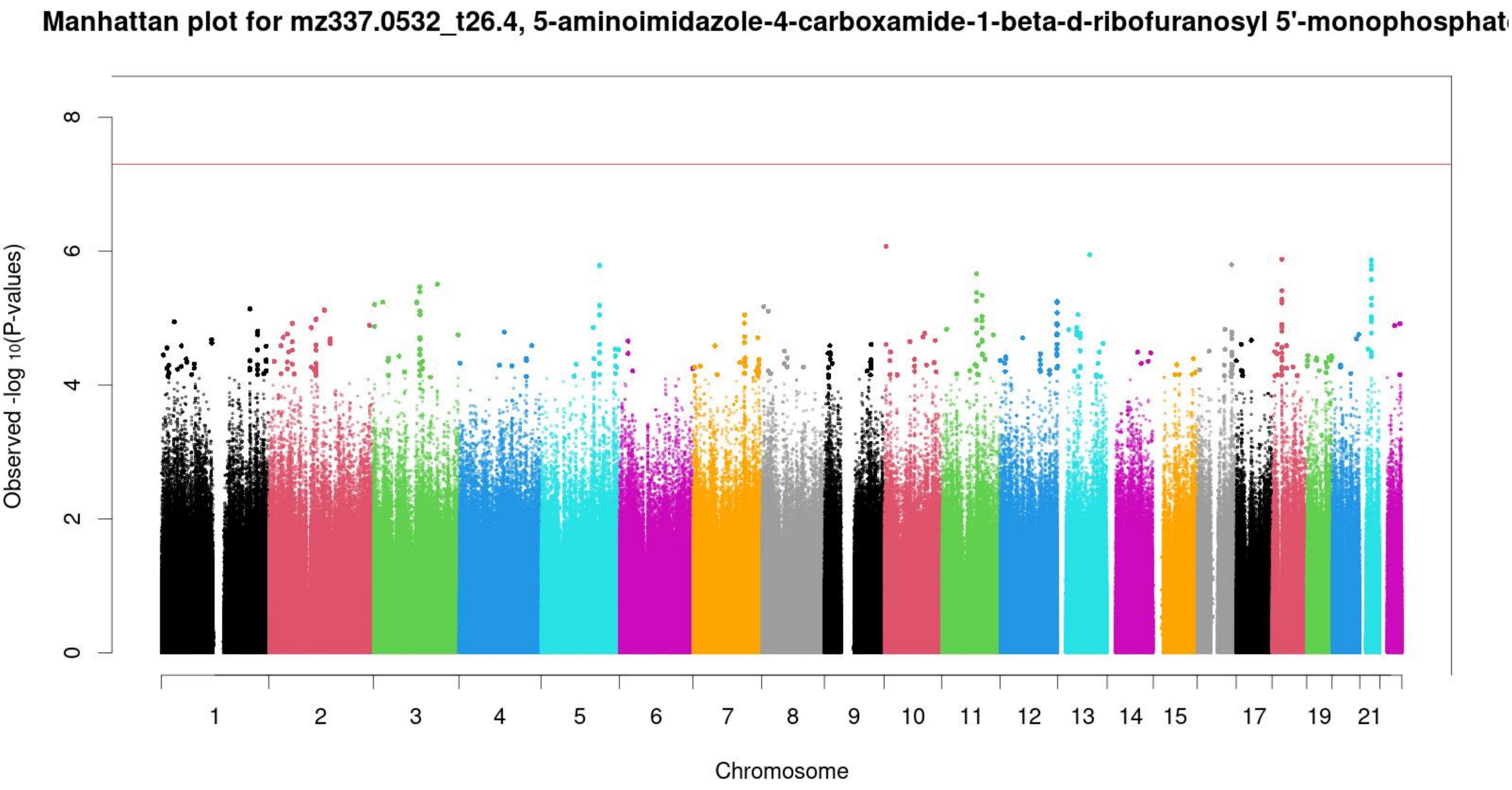
Manhattan plot for mz337.0532_t26.4, 5-aminoimidazole-4-carboxamide-1-beta-d-ribofuranosyl 5'-monophosphat
Observed -log 10(P-values)
0
2
4
6
8
1
2
3
4
5
6
7
8
9
10
11
12
13
14
15
17
19
21
Chromosome

Manhattan plot for mz344.2795_t32.4, lauroylcarnitine

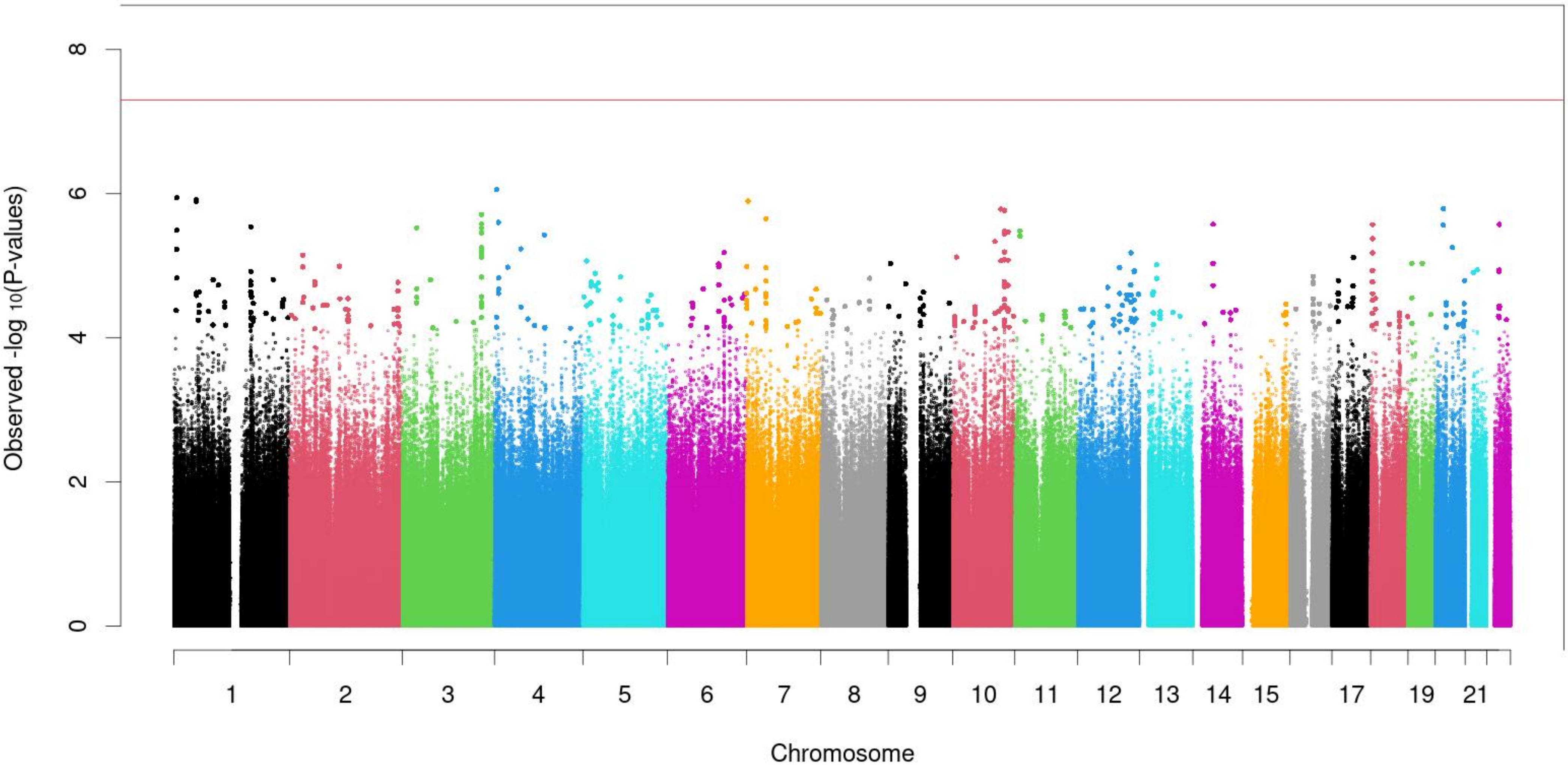

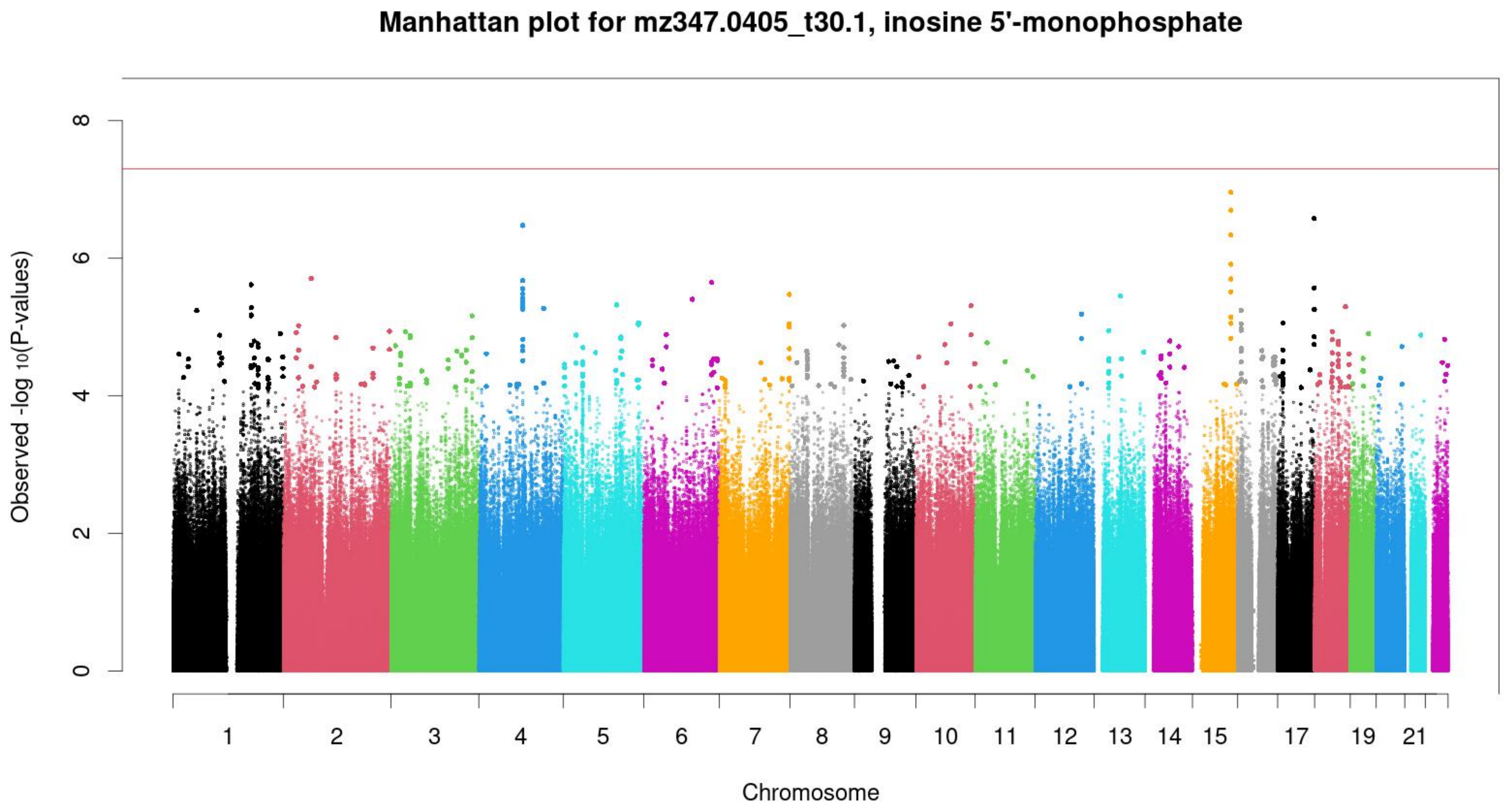
Manhattan plot for mz347.0405_t30.1, inosine 5'-monophosphate
Observed -log 10(P-values)
0
2
4
6
8
1
2
3
4
5
6
7
8
9
10
11
12
13
14
15
17
19
21
Chromosome

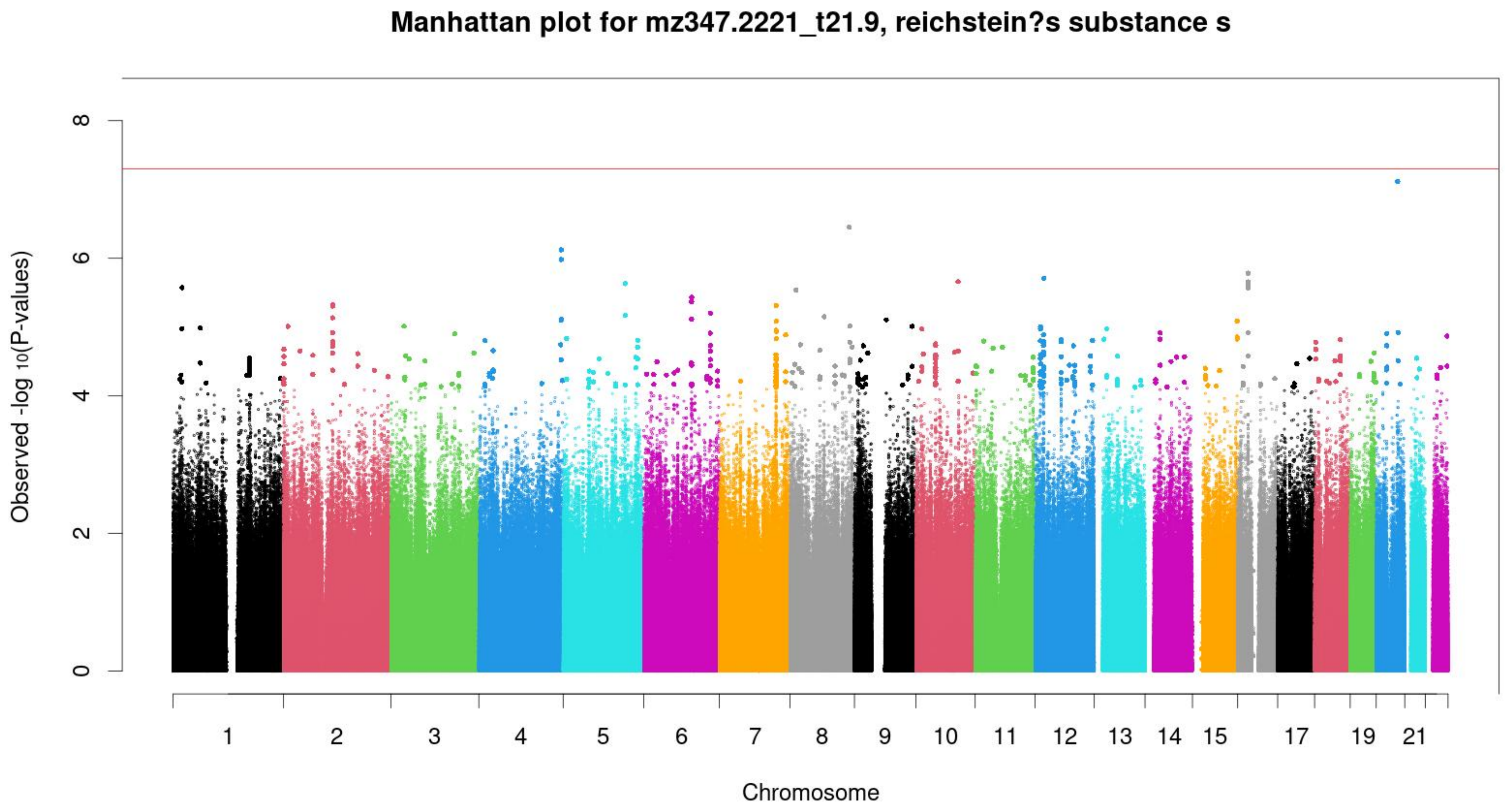
Manhattan plot for mz347.2221_t21.9, reichstein?s substance s
Observed -log 10(P-values)
0
2
4
6
8
1
2
3
4
5
6
7
8
9
10
11
12
13
14
15
17
19
21
Chromosome

Manhattan plot for mz357.2999_t24, 1-oleoyl-rac-glycerol

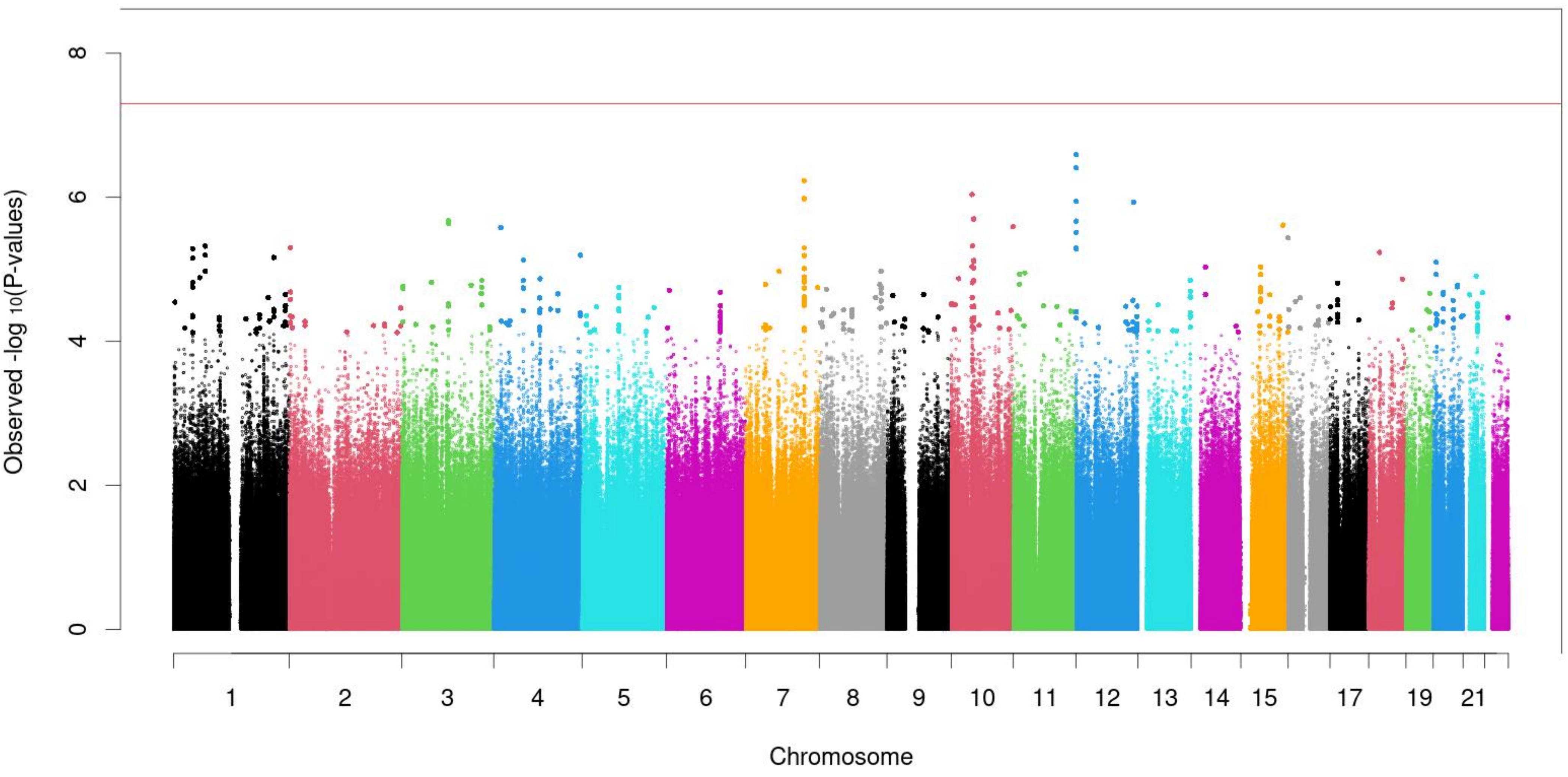

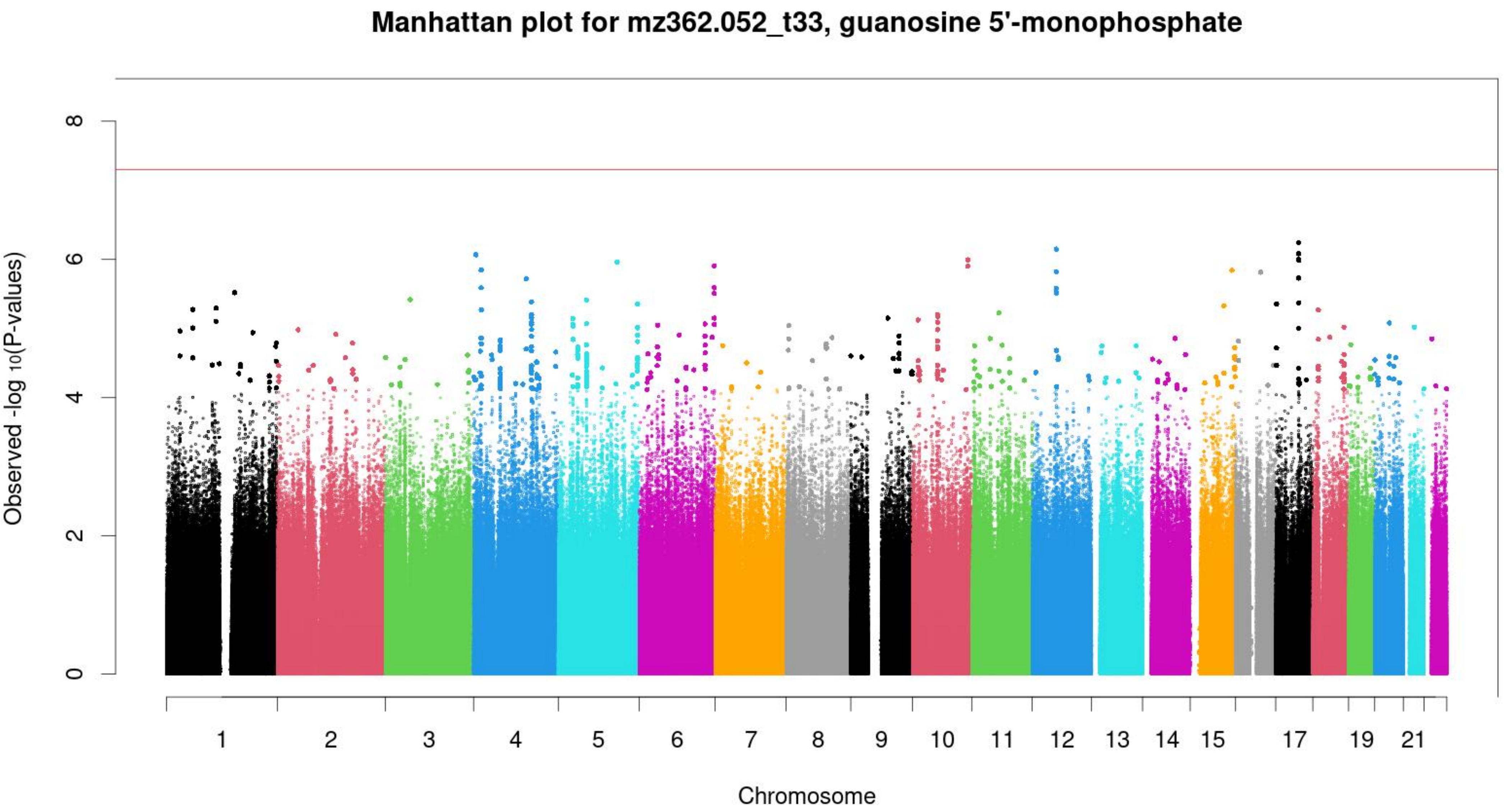
Manhattan plot for mz362.052_t33, guanosine 5'-monophosphate
Observed -log 10(P-values)
0
2
4
6
8
1
2
3
4
5
6
7
8
9
10
11
12
13
14
15
17
19
21
Chromosome

Manhattan plot for mz363.2163_t27.2, cortisol

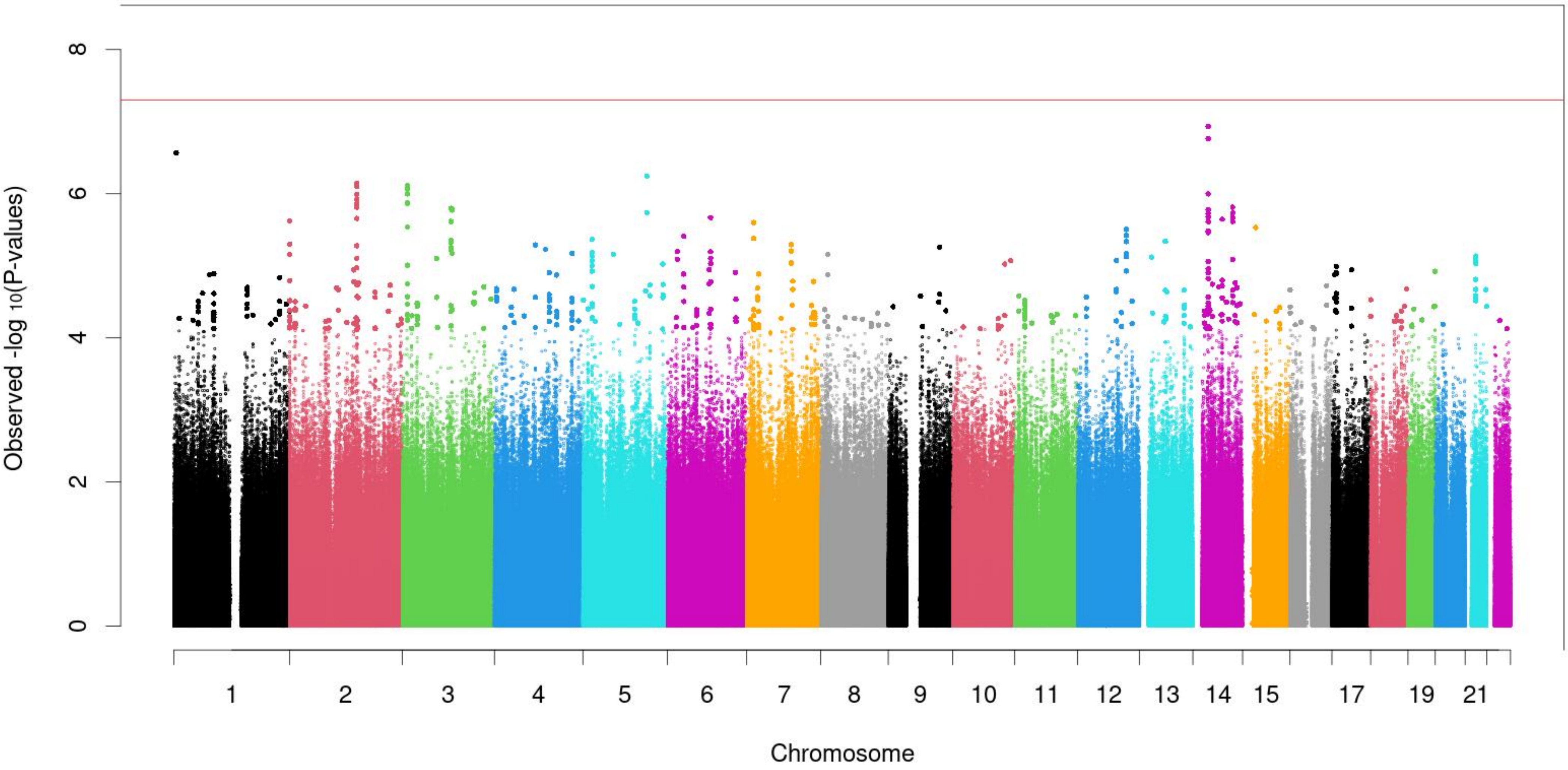

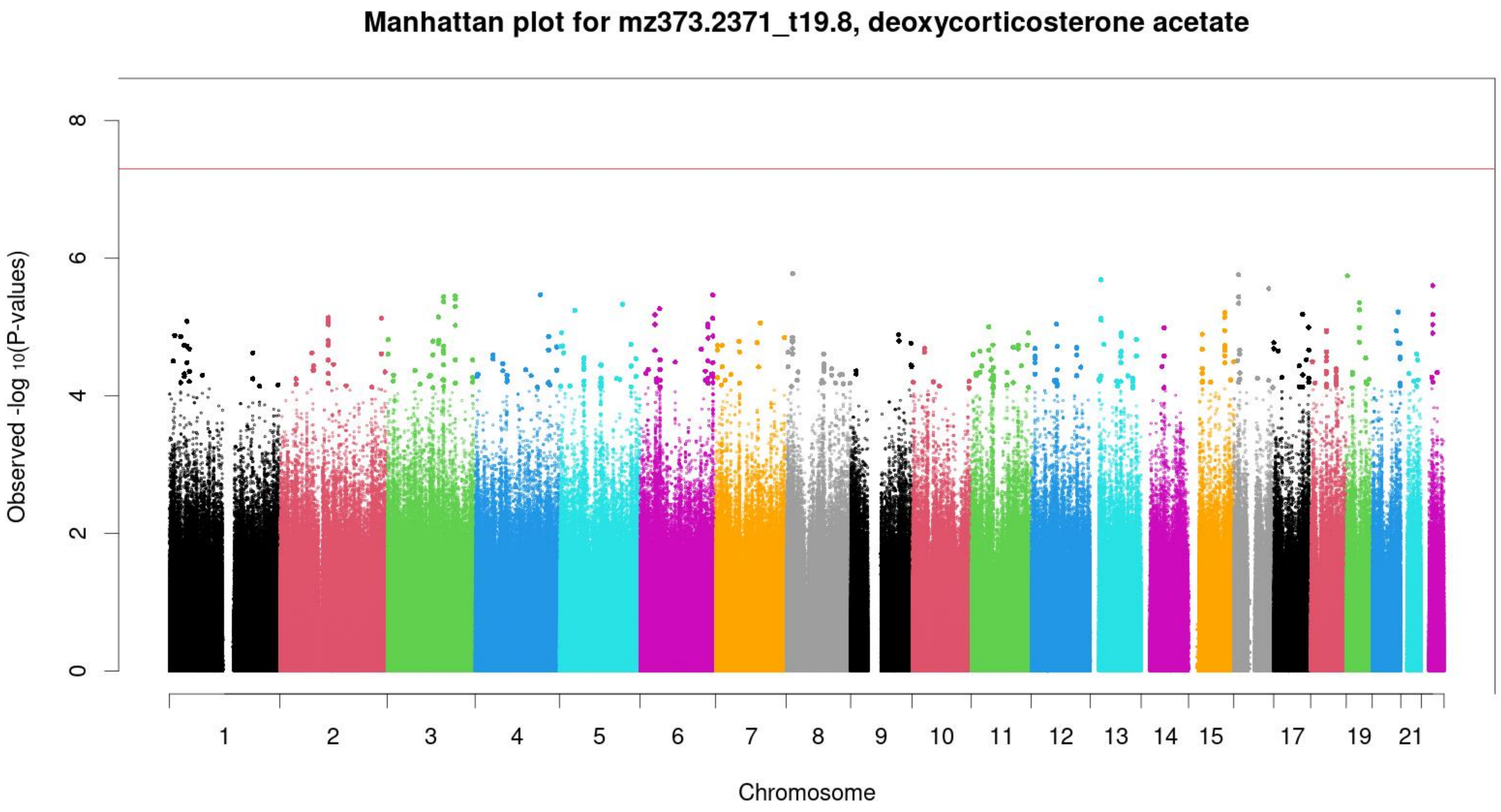

Manhattan plot for mz373.2371_t19.8, deoxycorticosterone acetate
Observed -log 10(P-values)
0
2
4
6
8
1
2
3
4
5
6
7
8
9
10
11
12
13
14
15
17
19
21
Chromosome

Manhattan plot for mz375.2926_t209.2, 3alpha-hydroxy-5beta-cholanate

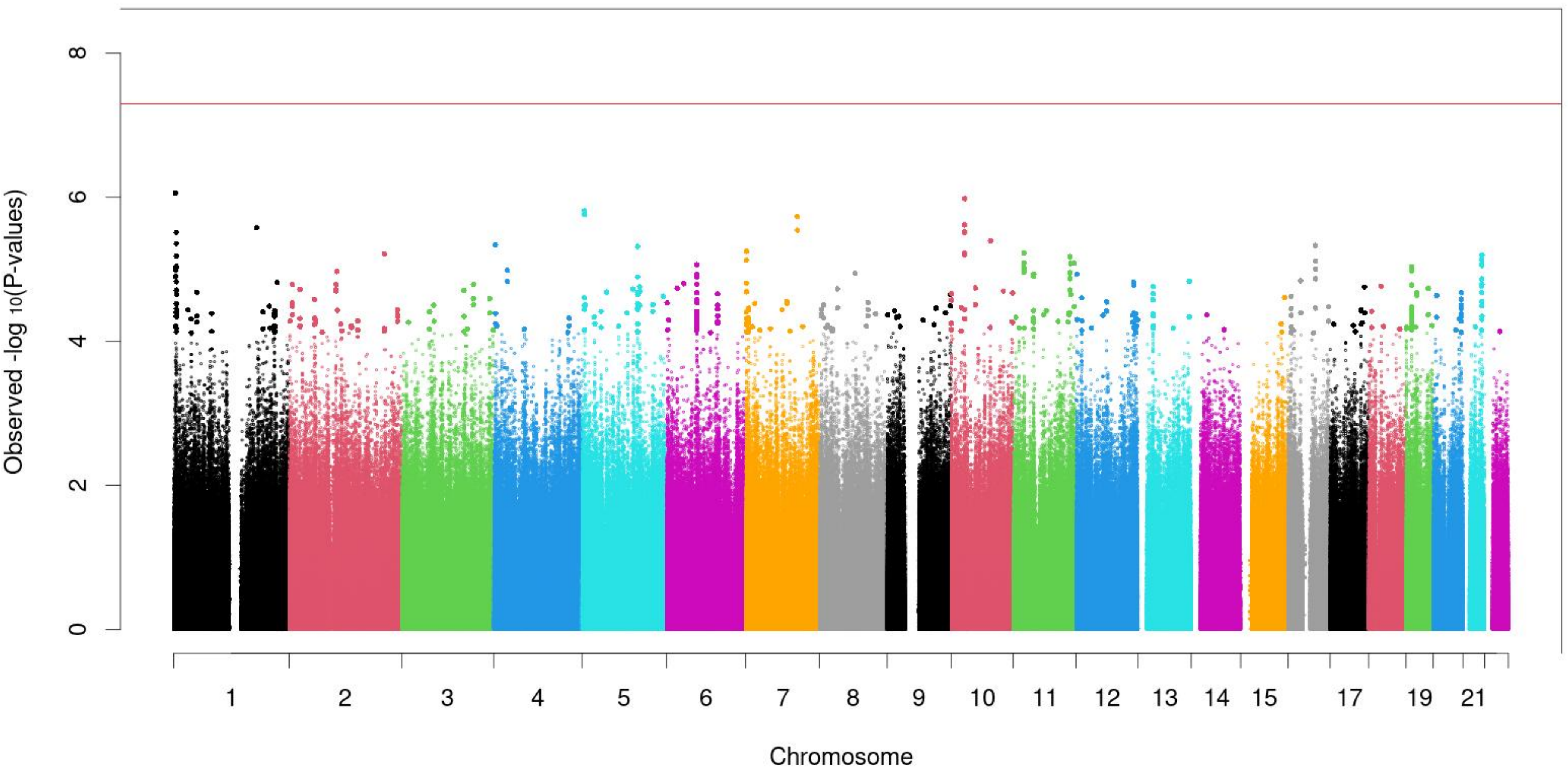

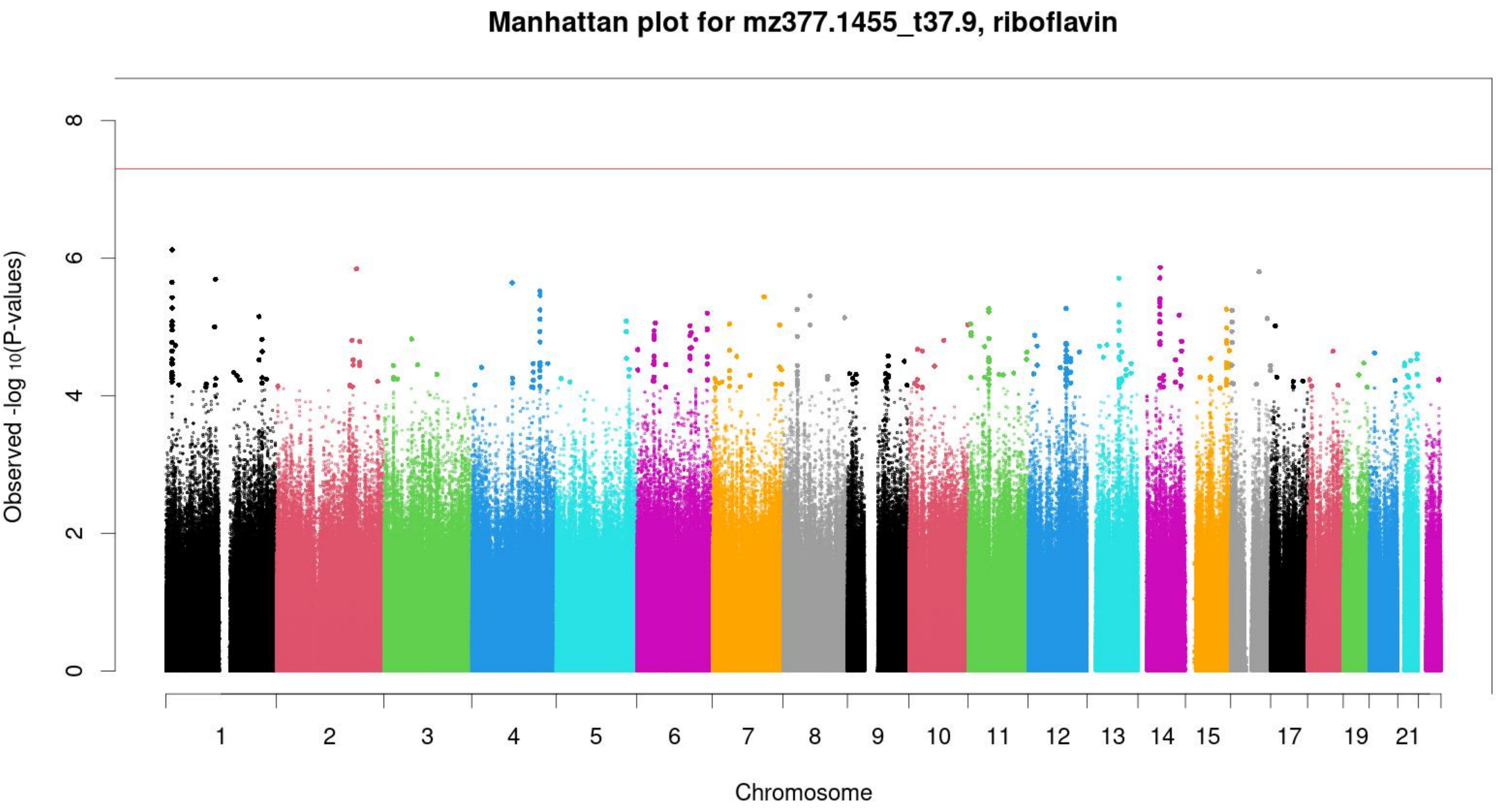
Manhattan plot for mz377.1455_t37.9, riboflavin
Observed -log 10(P-values)
0
2
4
6
8
1
2
3
4
5
6
7
8
9
10
11
12
13
14
15
17
19
21
Chromosome

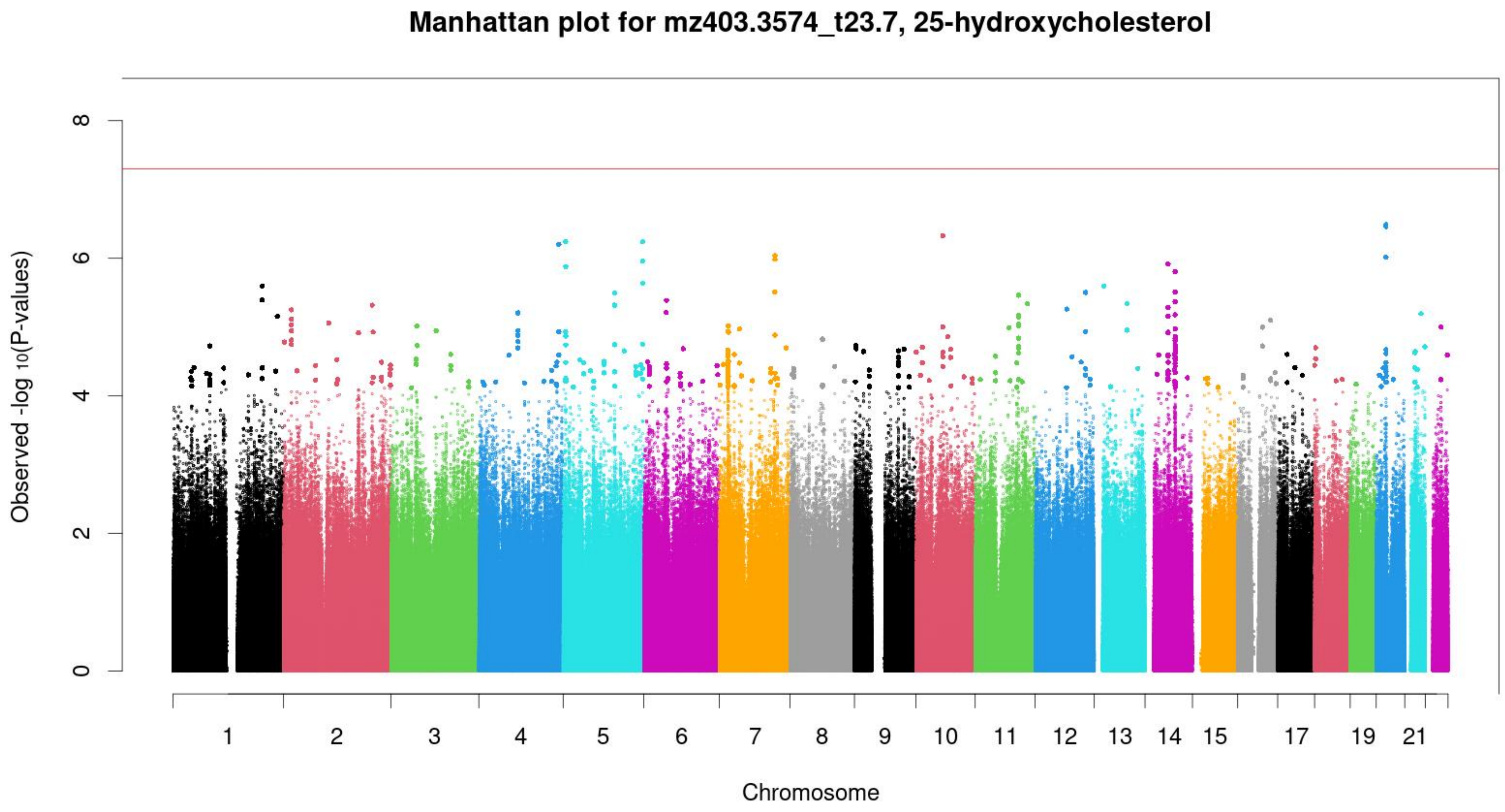
Manhattan plot for mz403.3574_t23.7, 25-hydroxycholesterol
Observed -log 10(P-values)
0
2
4
6
8
1
2
3
4
5
6
7
8
9
10
11
12
13
14
15
17
19
21
Chromosome

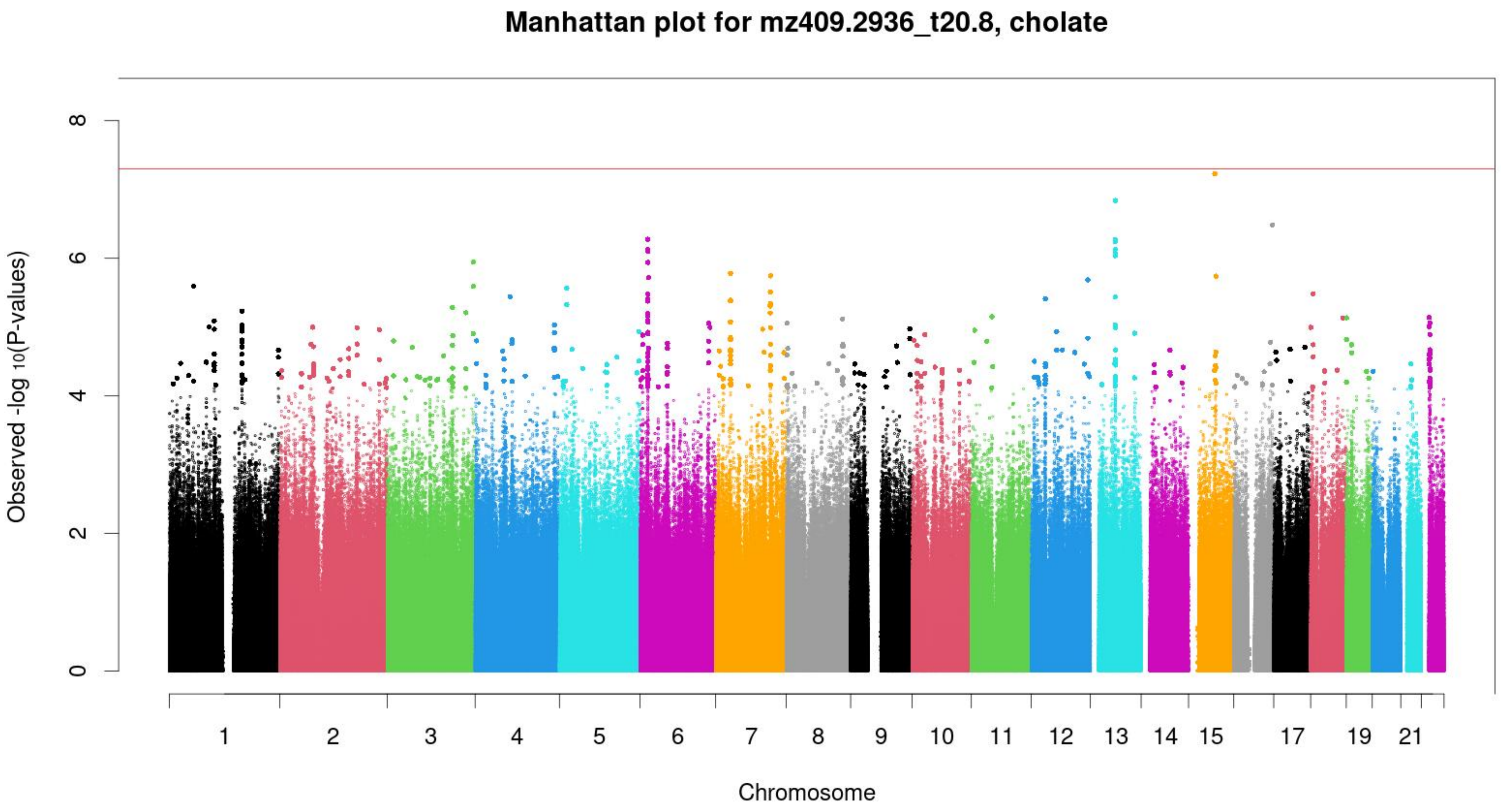
Manhattan plot for mz409.2936_t20.8, cholate
Observed -log 10(P-values)
0
2
4
6
8
1
2
3
4
5
6
7
8
9
10
11
12
13
14
15
17
19
21
Chromosome

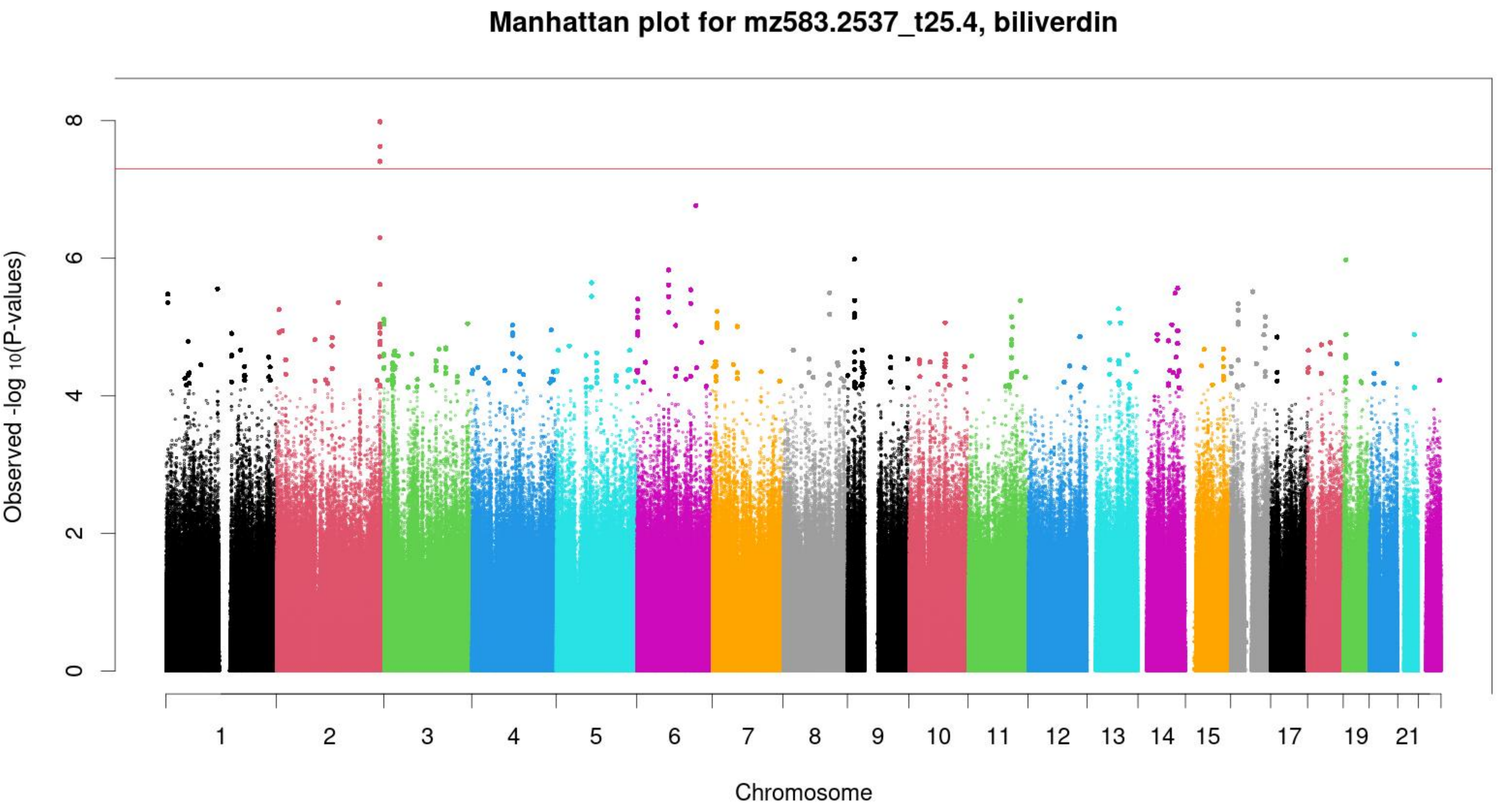
Manhattan plot for mz583.2537_t25.4, biliverdin
Observed -log 10(P-values)
0
2
4
6
8
1
2
3
4
5
6
7
8
9
10
11
12
13
14
15
17
19
21
Chromosome

Manhattan plot for mz585.2707_t23.2, bilirubin

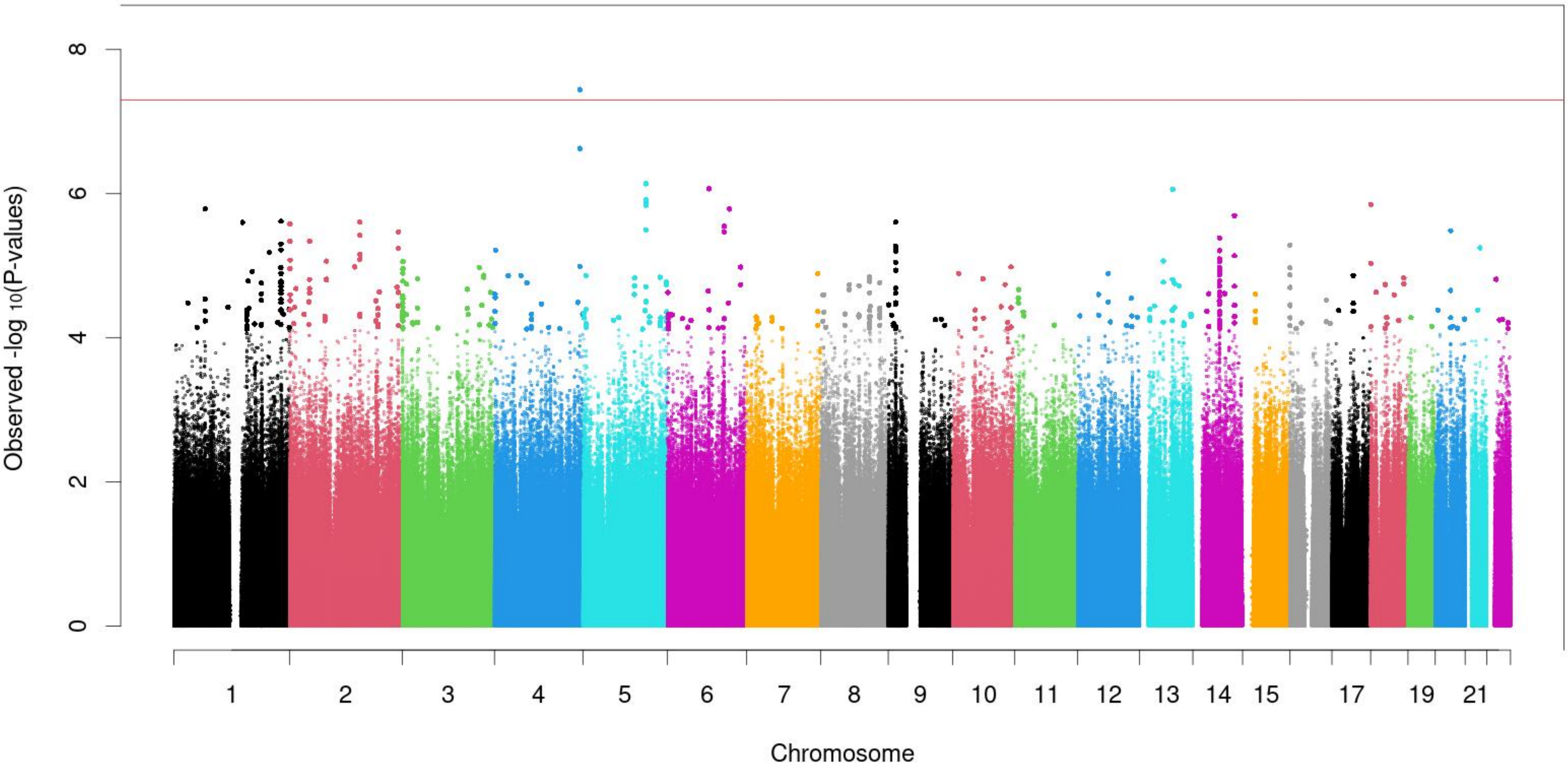

Manhattan plot for mz777.6935_t33.9, thyroxine

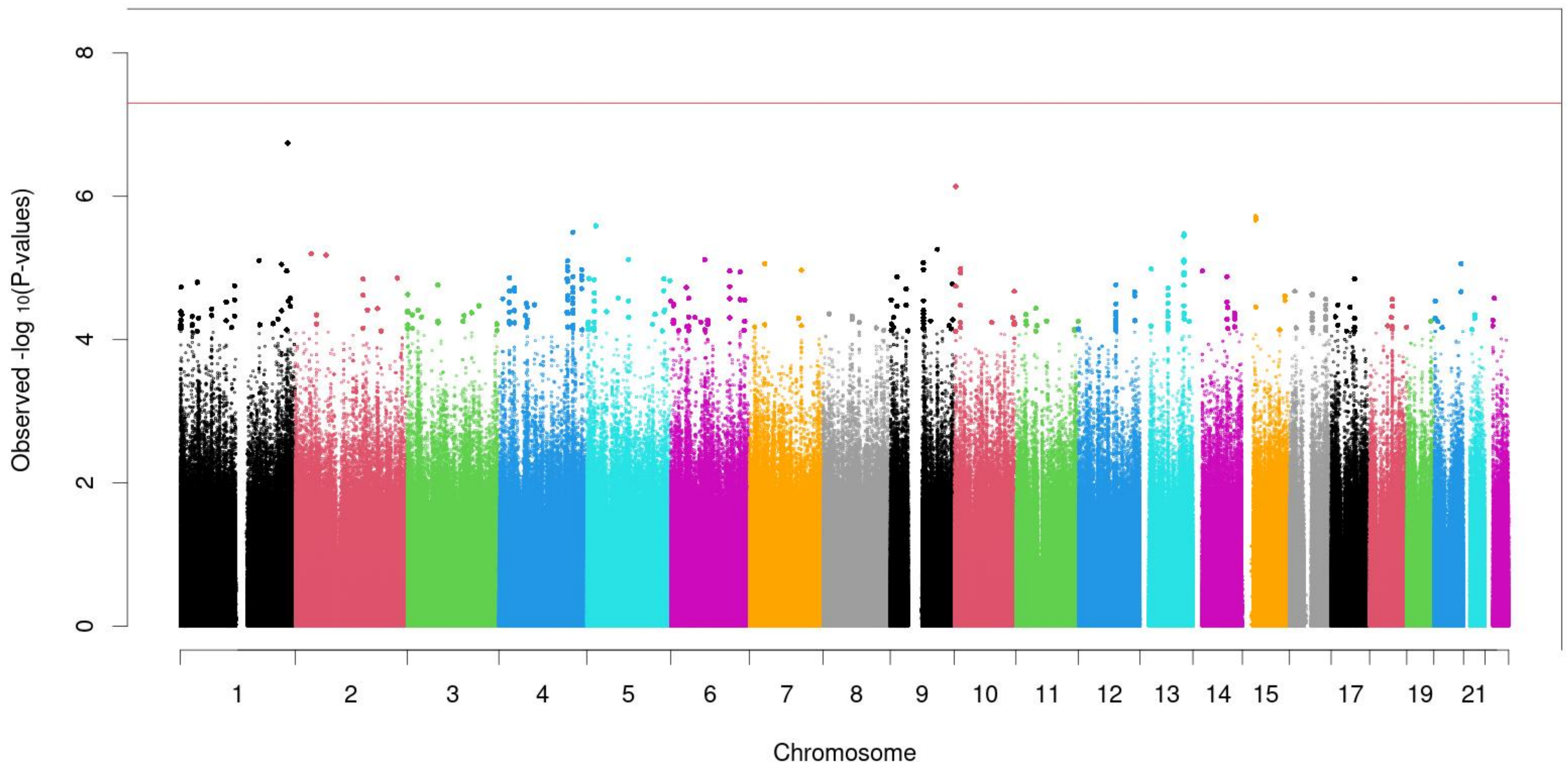

Supplement: Supplementary file 1 [file metabolites-12-00624-s001.zip › SuppFigS2_Man_plots.pdf]
