# Supplementary material for: Crosstalk between Host Genome and Metabolome among People with HIV in South Africa
Source: Metabolites. 2022 Jul 6;12(7):624. doi: 10.3390/metabo12070624 (PMC9316179; doi:10.3390/metabo12070624)
Supplement: Supplementary file 1 [file metabolites-12-00624-s001.zip › SuppTableS1_R1_Tracked.pdf]

**Supplementary Table S1. The 154 metabolites analyzed in GWAS.**

| Metabolic Feature (m/z_Time[s]) | Name | KEGG* ID | Liquid Chromatography | GWAS Inflation Factor |
|---|---|---|---|---|
| mz87.0087_t22.3 | pyruvate | C00022 | C18 negative | 1.00 |
| mz89.0244_t22.1 | (s)-lactate | C01432 | C18 negative | 0.99 |
| mz103.0035_t94 | malonate | C00383 | C18 negative | 1.00 |
| mz104.0351_t26.8 | serine | C00716 | C18 negative | 0.99 |
| mz105.0193_t22.9 | glycerate | C00258 | C18 negative | 1.00 |
| mz112.0516_t24.7 | creatinine | C00791 | C18 negative | 1.01 |
| mz116.0716_t21.8 | 5-aminopentanoate | C00431 | C18 negative | 1.00 |
| mz123.045_t27.4 | 3-hydroxybenzyl alcohol | C03351 | C18 negative | 1.01 |
| mz130.0873_t23 | norleucine | C01933 | C18 negative | 0.99 |
| mz131.0459_t20.7 | 3-ureidopropionate | C02642 | C18 negative | 1.00 |
| mz135.0306_t23.2 | hypoxanthine | C00262 | C18 negative | 1.00 |
| mz140.0118_t36.8 | ethanolamine phosphate | C00346 | C18 negative | 0.99 |
| mz143.1077_t59.1 | caprylic acid | C06423 | C18 negative | 1.00 |
| mz145.0142_t24.5 | alpha-ketoglutaric acid | C00026 | C18 negative | 1.00 |
| mz145.0618_t22.9 | glutamine | C00064 | C18 negative | 1.00 |
| mz145.0982_t37 | lysine | C16440 | C18 negative | 1.00 |
| mz147.0298_t23.1 | citramalate | C00815 | C18 negative | 0.99 |
| mz147.045_t46.9 | trans-cinnamate | C10438 | C18 negative | 0.99 |
| mz147.0661_t24.1 | (rs)-mevalonic acid lithium salt | C00418 | C18 negative | 1.01 |
| mz148.0437_t35.7 | methionine | C00073 | C18 negative | 0.99 |
| mz149.0278_t28.8 | 2-hydroxy-4-(methylthio)butyric acid | CA1484 | C18 negative | 1.02 |
| mz151.026_t22.8 | xanthine | C00385 | C18 negative | 0.99 |
| mz154.0621_t21.1 | histidine | C00135 | C18 negative | 1.01 |
| mz162.056_t60.4 | 3-methyl-2-oxindole | CA1325 | C18 negative | 1.00 |
| mz164.0717_t22.7 | phenylalanine | C00079 | C18 negative | 1.00 |
| mz165.0557_t49.5 | 3-(2-hydroxyphenyl)propanoate | C01198 | C18 negative | 1.00 |
| mz167.0211_t23.9 | urate | C00366 | C18 negative | 1.00 |
| mz173.0091_t25.6 | trans-aconitate | C02341 | C18 negative | 1.00 |
| mz173.0456_t23.2 | shikimate | C00493 | C18 negative | 0.99 |
| mz173.1044_t19.5 | arginine | C00062 | C18 negative | 0.99 |
| mz174.056_t47.1 | indole-3-acetate | C00954 | C18 negative | 1.00 |
| mz174.0884_t21.1 | citrulline | C00327 | C18 negative | 1.01 |
| mz176.0388_t26.6 | formyl-l-methionyl peptide | C03145 | C18 negative | 1.00 |
| mz177.0405_t19.7 | d-gulonic acid gama-lactone | C01040 | C18 negative | 1.00 |
| mz178.051_t30.3 | hippurate | C01586 | C18 negative | 1.00 |
| mz179.035_t25.5 | caffeate | C01481 | C18 negative | 0.99 |
| mz189.088_t31.9 | ll-2,6-diaminoheptanedioate | C00666 | C18 negative | 0.99 |
| mz190.0544_t27.4 | n-acetyl-dl-methionine | CA1210 | C18 negative | 1.00 |
| mz195.0511_t23.4 | gluconic acid | C00257 | C18 negative | 1.00 |

| | | | | |
|---|---|---|---|---|
| mz197.0433_t40.7 | 3-methoxy-4-hydroxymandelate | C05584 | C18 negative | 1.00 |
| mz199.1704_t152.3 | lauric acid | C02679 | C18 negative | 1.00 |
| mz202.1087_t40.9 | n6-(delta2-isopentenyl)-adenine | C04083 | C18 negative | 1.00 |
| mz206.0825_t46.2 | n-acetyl-l-phenylalanine | C03519 | C18 negative | 1.01 |
| mz209.0308_t34.2 | d-saccharic acid | C00818 | C18 negative | 1.01 |
| mz218.1037_t23.6 | d-pantothenic acid | C00864 | C18 negative | 1.00 |
| mz239.017_t24.2 | cystine | C01420 | C18 negative | 1.00 |
| mz243.0627_t22.4 | uridine | C00299 | C18 negative | 1.01 |
| mz245.0935_t49 | n-acetyl-d-tryptophan | C03137 | C18 negative | 1.01 |
| mz253.2177_t230.4 | palmitoleic acid | C08362 | C18 negative | 1.00 |
| mz266.0893_t18.4 | adenosine | C00212 | C18 negative | 1.01 |
| mz279.2335_t237.1 | linoleate | C01595 | C18 negative | 1.01 |
| mz281.2493_t256.8 | elaidic acid | C01712 | C18 negative | 1.01 |
| mz283.265_t282.7 | stearate | C01530 | C18 negative | 1.00 |
| mz301.239_t252 | rac-glycerol 1-myristate | CA1370 | C18 negative | 1.01 |
| mz307.0316_t28.5 | 2'-deoxyuridine 5'-mono-phosphate | C00365 | C18 negative | 1.00 |
| mz308.1_t22.3 | n-acetylneuraminate | C00270 | C18 negative | 0.99 |
| mz311.296_t295.2 | arachidic acid | C06425 | C18 negative | 1.01 |
| mz337.0532_t26.4 | 5-aminoimidazole-4-carboxamide-1-beta-d-ribofuranosyl 5'-monophosphate | C04677 | C18 negative | 1.00 |
| mz347.0405_t30.1 | inosine 5'-monophosphate | C00130 | C18 negative | 1.00 |
| mz362.052_t33 | guanosine 5'-monophosphate | C00144 | C18 negative | 1.00 |
| mz375.2926_t209.2 | 3alpha-hydroxy-5beta-cholanate | C03990 | C18 negative | 1.00 |
| mz90.0551_t71.3 | beta-alanine | C00099 | HILIC positive | 1.00 |
| mz94.0653_t45.9 | aniline | C00292 | HILIC positive | 1.00 |
| mz96.0445_t35.3 | 2-hydroxypyridine | C02502 | HILIC positive | 0.99 |
| mz101.06_t20 | 5-valerolactone | C02240 | HILIC positive | 1.00 |
| mz102.055_t78.3 | 1-aminocyclopropane-1-carboxylate | C01234 | HILIC positive | 1.00 |
| mz103.0391_t80.3 | 2-oxobutanoate | C00109 | HILIC positive | 1.01 |
| mz106.05_t115 | serine | C00716 | HILIC positive | 0.99 |
| mz110.0272_t66.7 | hypotaurine | C00519 | HILIC positive | 1.00 |
| mz113.0347_t39.3 | uracil | C00106 | HILIC positive | 1.00 |
| mz113.0597_t29.2 | sorbate | CA1411 | HILIC positive | 1.00 |
| mz114.0661_t40.7 | creatinine | C00791 | HILIC positive | 1.00 |
| mz116.0343_t85.6 | maleamate | C01596 | HILIC positive | 1.00 |
| mz116.0707_t58 | proline | C16435 | HILIC positive | 0.99 |
| mz118.0612_t73.1 | guanidinoacetate | C00581 | HILIC positive | 1.00 |
| mz123.0441_t56.2 | 3-hydroxybenzaldehyde | C03067 | HILIC positive | 0.99 |
| mz123.0552_t32.2 | nicotinamide | C00153 | HILIC positive | 1.00 |
| mz126.022_t55.6 | taurine | C00245 | HILIC positive | 0.99 |
| mz127.0503_t70.4 | 4-imidazoleacetic acid | C02835 | HILIC positive | 0.98 |

| | | | | |
|---|---|---|---|---|
| mz130.0863_t52.9 | pipecolate | C00408 | HILIC positive | 0.99 |
| mz132.0765_t72.5 | creatine | C00300 | HILIC positive | 1.00 |
| mz133.0647_t24.9 | trans-cinnamaldehyde | C00903 | HILIC positive | 1.00 |
| mz134.06_t21.2 | indoxyl sulfate | C08481 | HILIC positive | 1.00 |
| mz136.0428_t56.3 | homocysteine | C05330 | HILIC positive | 0.99 |
| mz137.0457_t39.7 | hypoxanthine | C00262 | HILIC positive | 1.00 |
| mz138.055_t53.8 | trigonelline | C01004 | HILIC positive | 0.99 |
| mz138.0795_t55.7 | 1-methylnicotinamide | C02918 | HILIC positive | 1.00 |
| mz139.0502_t64.4 | urocanate | C00785 | HILIC positive | 1.00 |
| mz144.0807_t46.2 | 1-naphthylamine | C14790 | HILIC positive | 1.00 |
| mz147.0764_t81.7 | glutamine | C00064 | HILIC positive | 1.00 |
| mz148.0757_t44.7 | 3-methyl-2-oxindole | CA1325 | HILIC positive | 1.00 |
| mz150.0583_t52.3 | methionine | C00073 | HILIC positive | 1.00 |
| mz153.0407_t42 | xanthine | C00385 | HILIC positive | 1.00 |
| mz153.0772_t51.3 | arabitol | CA1387 | HILIC positive | 1.00 |
| mz156.0767_t105 | histidine | C00135 | HILIC positive | 1.00 |
| mz159.0515_t42.1 | allantoin | C01551 | HILIC positive | 1.00 |
| mz162.1124_t80.7 | carnitine | C00318 | HILIC positive | 1.00 |
| mz163.1229_t40.3 | (s)-nicotine | C16150 | HILIC positive | 1.01 |
| mz164.0377_t43.9 | n-acetyl-l-cysteine | C06809 | HILIC positive | 1.00 |
| mz166.0862_t46.5 | phenylalanine | C00079 | HILIC positive | 1.00 |
| mz168.1019_t36 | 3-methoxytyramine | C05587 | HILIC positive | 1.00 |
| mz169.0357_t49 | urate | C00366 | HILIC positive | 1.00 |
| mz169.0971_t67.2 | pyridoxamine | C00534 | HILIC positive | 1.00 |
| mz175.0866_t42.7 | indole-3-acetamide | C02693 | HILIC positive | 1.01 |
| mz176.0663_t49 | n-amidino-l-aspartate | C03139 | HILIC positive | 1.00 |
| mz176.1029_t90.6 | citrulline | C00327 | HILIC positive | 1.00 |
| mz178.0533_t51.9 | formyl-l-methionyl peptide | C03145 | HILIC positive | 1.00 |
| mz180.0329_t57.5 | s-carboxymethyl-l-cysteine | C03727 | HILIC positive | 1.00 |
| mz180.1019_t47.4 | 1-methyl-6,7-dihydroxy-1,2,3,4-tetrahydroisoquinoline | C09642 | HILIC positive | 1.00 |
| mz182.0811_t55.8 | tyrosine | C00082 | HILIC positive | 0.99 |
| mz184.0603_t21 | 4-pyridoxate | C00847 | HILIC positive | 1.00 |
| mz189.1233_t69.3 | nalpha-acetyl-l-lysine | C12989 | HILIC positive | 0.99 |
| mz190.0496_t20 | 4-hydroxy-2-quinolinecarboxylic acid | C01717 | HILIC positive | 1.01 |
| mz192.0655_t46.6 | 5-hydroxyindoleacetate | C05635 | HILIC positive | 1.00 |
| mz195.0505_t48.3 | d-glucuronic acid | C00191 | HILIC positive | 1.01 |
| mz195.0651_t53.4 | ferulate | C01494 | HILIC positive | 1.00 |
| mz198.076_t50.3 | 3,4-dihydroxy-l-phenylalanine (l-dopa) | C00355 | HILIC positive | 0.99 |
| mz204.123_t63.1 | o-acetyl-l-carnitine | C02571 | HILIC positive | 1.00 |
| mz205.0972_t45.9 | tryptophan | C00078 | HILIC positive | 1.00 |
| mz209.0921_t47.2 | kynurenine | C00328 | HILIC positive | 1.00 |

| | | | | |
|---|---|---|---|---|
| mz212.0433_t126.8 | phosphocreatine | C02305 | HILIC positive | 1.00 |
| mz220.1182_t64.5 | d-pantothenic acid | C00864 | HILIC positive | 1.00 |
| mz221.092_t44.8 | 5-hydroxy-l-tryptophan | C01017 | HILIC positive | 1.01 |
| mz222.0962_t43.6 | n-acetyl-d-glucosamine | C00140 | HILIC positive | 1.00 |
| mz225.1484_t26.7 | methyl jasmonate | C11512 | HILIC positive | 1.00 |
| mz237.0362_t73.2 | quinate | C00296 | HILIC positive | 1.00 |
| mz241.0311_t181.3 | cystine | C00491 | HILIC positive | 0.99 |
| mz245.0769_t45.3 | uridine | C00299 | HILIC positive | 0.99 |
| mz245.0971_t55 | biotin | C00120 | HILIC positive | 1.01 |
| mz247.1076_t34.1 | n-acetyl-d-tryptophan | C03137 | HILIC positive | 1.00 |
| mz255.2318_t21.7 | palmitoleic acid | C08362 | HILIC positive | 1.00 |
| mz258.1102_t114.4 | sn-glycero-3-phosphocholine | C00670 | HILIC positive | 0.99 |
| mz266.1215_t33.8 | thiamine | C00378 | HILIC positive | 1.00 |
| mz269.0881_t43.1 | inosine | C00294 | HILIC positive | 1.00 |
| mz279.2319_t23.7 | gamma-linolenic acid | C06426 | HILIC positive | 1.00 |
| mz281.2476_t28.9 | linoleate | C01595 | HILIC positive | 0.99 |
| mz301.2167_t26.6 | retinoate | C00777 | HILIC positive | 0.99 |
| mz302.3054_t35.1 | sphinganine | C00836 | HILIC positive | 1.00 |
| mz308.0929_t55.3 | glutathione | C00051 | HILIC positive | 0.98 |
| mz310.1137_t48.2 | n-acetylneuraminate | C00270 | HILIC positive | 1.00 |
| mz319.0692_t48.2 | melanin | C05606 | HILIC positive | 1.00 |
| mz329.2502_t29.9 | docosahexaenoic acid | C06429 | HILIC positive | 1.01 |
| mz330.0603_t50 | adenosine 3,5-cyclic monophosphate | C00575 | HILIC positive | 0.99 |
| mz344.2795_t32.4 | lauroylcarnitine | CA1406 | HILIC positive | 1.00 |
| mz347.2221_t21.9 | reichstein?s substance s | C05488 | HILIC positive | 1.00 |
| mz357.2999_t24 | 1-oleoyl-rac-glycerol | CA1373 | HILIC positive | 0.99 |
| mz363.2163_t27.2 | cortisol | C00735 | HILIC positive | 1.01 |
| mz373.2371_t19.8 | deoxycorticosterone acetate | C14554 | HILIC positive | 1.00 |
| mz377.1455_t37.9 | riboflavin | C00255 | HILIC positive | 0.99 |
| mz403.3574_t23.7 | 25-hydroxycholesterol | C15519 | HILIC positive | 0.99 |
| mz409.2936_t20.8 | cholate | C00695 | HILIC positive | 0.99 |
| mz583.2537_t25.4 | biliverdin | C00500 | HILIC positive | 0.99 |
| mz585.2707_t23.2 | bilirubin | C00486 | HILIC positive | 0.99 |
| mz777.6935_t33.9 | thyroxine | C01829 | HILIC positive | 1.00 |

*KEGG: Kyoto Encyclopedia of Genes and Genomes database.
